# Tailoring Liquid Crystalline Self‐Assembly in Dialkoxybenzimidazoles: The Role of Charge and Shape

**DOI:** 10.1002/cphc.70588

**Published:** 2026-10-22

**Authors:** Eugen Wuckert, Nikolai Scheuring, Sara Simonovska, Nadia Kapernaum, Wolfgang Frey, Johanna Osterbrink, Andreas Taubert, Frank Gießelmann, Anna Zens, Sabine Laschat

**Affiliations:** ^1^ Institut für Organische Chemie Universität Stuttgart Stuttgart Germany; ^2^ Institut für Physikalische Chemie Universität Stuttgart Stuttgart Germany; ^3^ Department of Chemistry Simon Fraser University Burnaby British Columbia Canada; ^4^ Lehrstuhl für Supramolekulare Chemie Institut für Chemie Universität Potsdam Potsdam Germany

**Keywords:** benzimidazolium ionic liquid crystals, cubic, ionic liquid crystals, nematic, self‐assembly, smectic

## Abstract

Liquid crystalline self‐assembly in neutral and ionic dialkoxybenzimidazoles can be precisely tuned or suppressed by controlling molecular symmetry and charge, revealing key structure–property relationships for mesophase design. To elucidate these effects, a series of neutral (**L‐Bi(n)**, **U‐Bi(n)**) and ionic (**L‐Me**_**2**_ **Bi(n)I**, **U‐Me**_**2**_ **Bi(n)I**) derivatives differing in the substitution pattern of the side chains (*n* = chain length) at the benzimidazole was synthesized and characterized by POM, DSC, and X‐ray diffraction. Nonionic systems exhibit mesomorphism only for rod‐like linear homologues (*n* = 9–14), forming nematic phases, whereas the neutral bent U‐shaped derivatives remain non‐mesomorphic. In contrast, ionic U‐shaped derivatives form smectic phases, including a cubic phase for *n* = 12, while linear derivatives do not form mesophases. The solid‐state structures demonstrate that both the capacity for hydrogen bonding in the neutral derivatives and the presence of π‐stacking interactions in the ionic derivatives significantly influence the molecular packing. Furthermore, distinct differences in the packing arrangements are observed depending on the specific substitution patterns.

## Graphical abstract

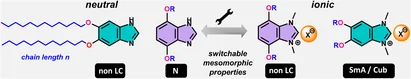

## 1 Introduction

Ionic liquid crystals (ILCs) have attracted much interest due to their beneficial combination of tailored organic salts with low vapor pressure, adjustable polarity, wide electrochemical window typical for ionic liquids, and their long‐range orientational order and anisotropic physical properties typical for thermotropic liquid crystals (LCs) [1, 2, 3, 4, 5, 6, 7, 8, 9]. The joint venture of the two material classes results in unique properties such as 1D electronic or ionic conductivity suitable for broad applications [10, 11, 12]. The molecular building blocks of ILCs consist of an ionic (cationic or anionic) headgroup with or without mesogenic subunit and one or more nonpolar side chains [1, 2, 3, 4, 5].

Benzimidazole is an important building block for both neutral LCs and ILCs. For example, amphiphilic neutral calamitic LCs with benzimidazole headgroup possessed SmC, SmA, and N phases [13, 14, 15, 16] as well as SmB and SmX phases and showed a large birefringence Δ*n* [17]. Neutral LCs with a flexible spacer between calamitic mesogen and benzimidazole headgroup showed SmA, SmC, and N phases and high proton conductivity facilitated by H‐bonding [18, 19]. When the benzimidazole was attached to a discotic mesogen, the resulting neutral LCs showed Col_h_ and Col_r_ phases, strong photoluminescence emission [20, 21, 22, 23, 24], as well as hole and electron conductivity [24]. Moreover, several benzimidazole metal complexes displaying liquid crystalline behavior have been reported, with the benzimidazole unit acting either as *N*‐heterocyclic carbene (NHC) ligand [25, 26, 27, 28, 29] as a Lewis basic monodentate *N*‐donor ligand [30] or as bi(tri)dentate chelate ligand [31, 32, 33, 34, 35, 36], some of which are showing polymorphism including an additional monotropic mesophase depending on the metal ion and number of side chains (for some structurally related non‐mesomorphic benzimidazole complexes, see ref [37, 38]).

On the other hand, benzimidazolium ILCs are much less explored. As shown in Scheme 1, calamitic ILCs **1** with a benzimidazolium headgroup showed SmA phases [39, 40], whereas the flexible tetradentate benzimidazolium ILC **2** possessed both thermotropic Col phases and lyotropic mesophases [41]. As discussed above, several metallomesogens based on benzimidazole NHC or *N*‐donor ligands have been reported such as **4**, **6**, **8** [25, 26, 27, 28, 29, 30, 38, 42], resulting mostly in monotropic mesomorphism and therefore thermodynamically unstable mesophases. However, no general predictions on the resulting mesophase geometry or mesomorphic behavior can be made. For example, benzimidazolium bromide **3** displayed a SmA phase, which disappeared upon complexation with Ag(I) to **4a** [42]. On the one hand, loss of liquid crystalline behavior upon complexation of ligands with metal ions, such as lanthanide ions, was already observed in the literature [32]. On the other hand, non‐mesogenic neutral benzimidazole **5** and benzimidazolium iodide **7** could be converted into LC metal complexes **6** and **8**, respectively [27, 28, 30]. Mesomorphic properties were also reported for Pd complex **4d** as well as for the corresponding benzimidazolium precursor [29]. The effect of preservation of liquid crystalline behavior was already observed for Lu‐containing complexes bearing benzimidazolium‐derived ligands [34].

**SCHEME 1:**
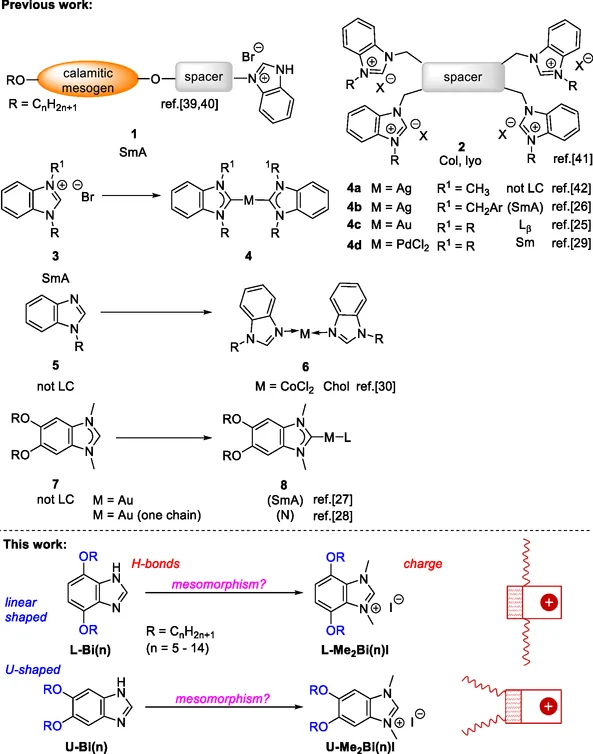
Previous work on liquid crystalline benzimidazolium ILCs and the present study of charge and shape effects on the phase behavior of novel dialkoxybenzimidazoles **L‐Bi(n)**, **U‐Bi(n)** and imidazolium salts **L‐Me**_**2**_ **Bi(n)I**, **U‐Me**_**2**_ **Bi(n)I**. Reported monotropic mesophases are marked by the use of parenthesis for the according phases.

Therefore, in the current work we wanted to get a more detailed insight into structure–property relationships regarding the phase behavior of neutral and ionic benzimidazole derivatives. We chose neutral compounds **L‐Bi(n)**, **U‐Bi(n)** differing in the substitution pattern of the side chains at the benzimidazole and their ionic counterparts **L‐Me**_**2**_ **Bi(n)I**, **U‐Me**_**2**_ **Bi(n)I** in order to study the role of symmetry and charge on the liquid crystalline self‐assembly. We surmised that in particular for the charged species **L‐Me**_**2**_ **Bi(n)I**, **U‐BMe**_**2**_ **Bi(n)I** **,** different conformations should lead to different packing behavior and thus different mesophase geometry. An extended conformation was expected for **U** **‐** **Me**_**2**_ **Bi(n)I**, whereas an amphiphilic‐like conformation was expected for **U‐Me**_**2**_ **Bi(n)I**. Recent molecular dynamic (MD) simulations suggested [43, 44, 45] that ILCs with the positive charge located in the center of the molecule should form preferably N phases, which are very rare among ILCs [2], whereas ILCs with the positive charge at the terminus of the molecule (i.e., amphiphilic shape) should strongly favor the SmA phase [44]. Our results reveal that molecular parameters can indeed be tailored to turn liquid crystalline self‐assembly on or off, depending on the symmetry of side‐chain positions and charge.

## 2 Results and Discussion

### 2.1 Synthesis and Solid‐State Structures

As shown in Scheme 2, the synthesis of linear and U‐shaped benzimidazolium iodides **L‐Me**_**2**_ **Bi(n)I** and **U‐Me**_**2**_ **Bi(n)I** commenced with nitration of the corresponding bis(alkyloxy)benzenes **L‐9(n)** or **U‐9(n)** bearing alkyl chains of varying lengths (*n* = 5–14). Subsequent reduction of the nitro groups and intramolecular cyclocondensation with formic acid afforded benzimidazole intermediates **L‐Bi(n)** and **U‐Bi(n)** in up to 65% yield. Final sequential *N*‐alkylations furnished the target benzimidazolium iodides **L‐Me**_**2**_ **Bi(n)I** and **U‐Me**_**2**_ **Bi(n)I** in yields of up to 91%.

**SCHEME 2:**
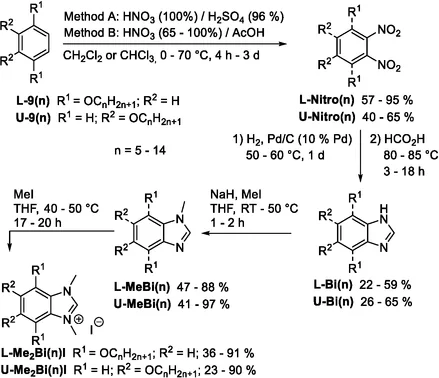
Synthesis of linear and U‐shaped benzimidazolium iodides **L‐Me**_**2**_ **Bi(n)I, U‐Me**_**2**_ **Bi(n)I**.

Suitable single crystals for crystal structure analysis of the compounds **L‐Me**_**2**_ **Bi(9)I**, **U‐Me**_**2**_ **Bi(5)I**, and **U‐Bi(6)** were obtained (Figure S154). Crystals of **L‐Bi(12)** could likewise be obtained. However, the data quality of this measurement was only sufficient to provide an indication of the overall structural features.

The solid‐state structure of neutral **U‐Bi(6)** consists of corrugated molecular layers interconnected by hydrogen bonds (N─N = 2.83 Å, Figure 1). The benzimidazole units involved in hydrogen bonding are inclined by 43.3°, giving rise to a slightly staircase‐like arrangement. Within each layer, neighboring molecules are rotated by 180°, resulting in a head‐to‐head orientation, while the layers pack in a nearly ideal lamellar fashion. In the ionic derivatives, hydrogen bonding is no longer possible because the donor and acceptor positions are methylated.

**FIGURE 1:**
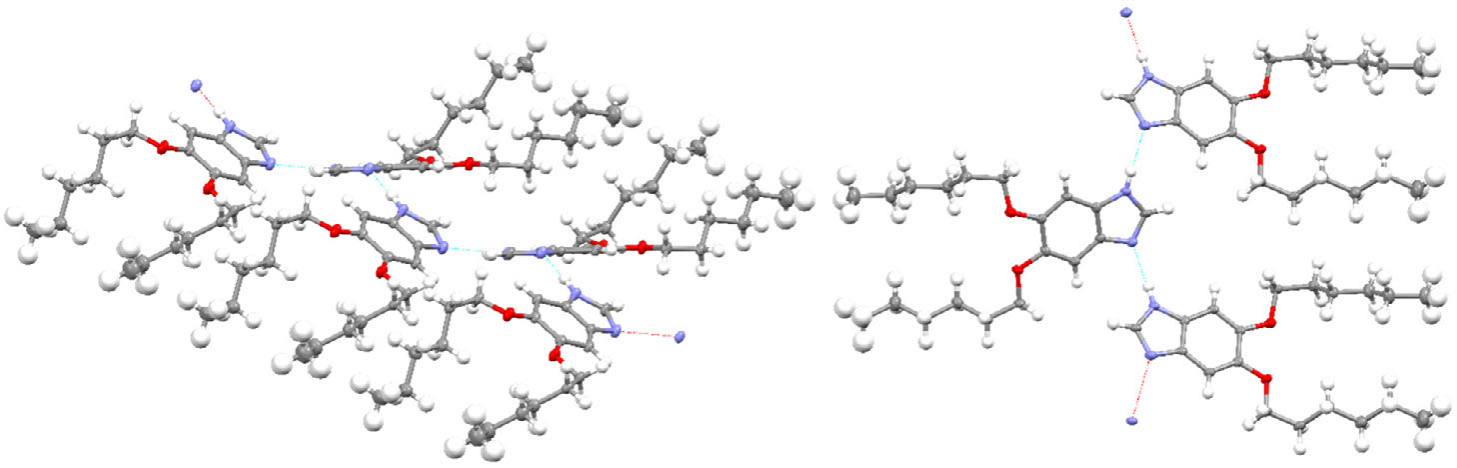
Illustration of the hydrogen bonds (dashed bonds) in a layer of **U‐Bi(6)**. Displacement ellipsoids at 50% probability level; hydrogen atoms omitted; color code: O red, C gray, N blue; H white. Further data are listed in the ESI, Section 5.

Both ionic compounds, **L‐Me**_**2**_ **Bi(9)I** and **U‐Me**_**2**_ **Bi(5)I**, also adopt layered structures (Figure 2). In **L‐Me**_**2**_ **Bi(9)I**, benzimidazole units rotate by 180° between adjacent layers, whereas they retain the same orientation in **U‐Me**_**2**_ **Bi(5)I**. Perpendicular views (Figure 2) reveal that **L‐Me**_**2**_ **Bi(9)I** forms a herringbone‐type arrangement with adjacent layers rotated by about 90° and interdigitating alkoxy side chains. In contrast, **U‐Me**_**2**_ **Bi(5)I** exhibits well‐defined lamellar layers with parallel side chains; these layers either stack directly or are separated by highly disordered iodide ions. Additional structural details are provided in the ESI (Section 5).

**FIGURE 2:**
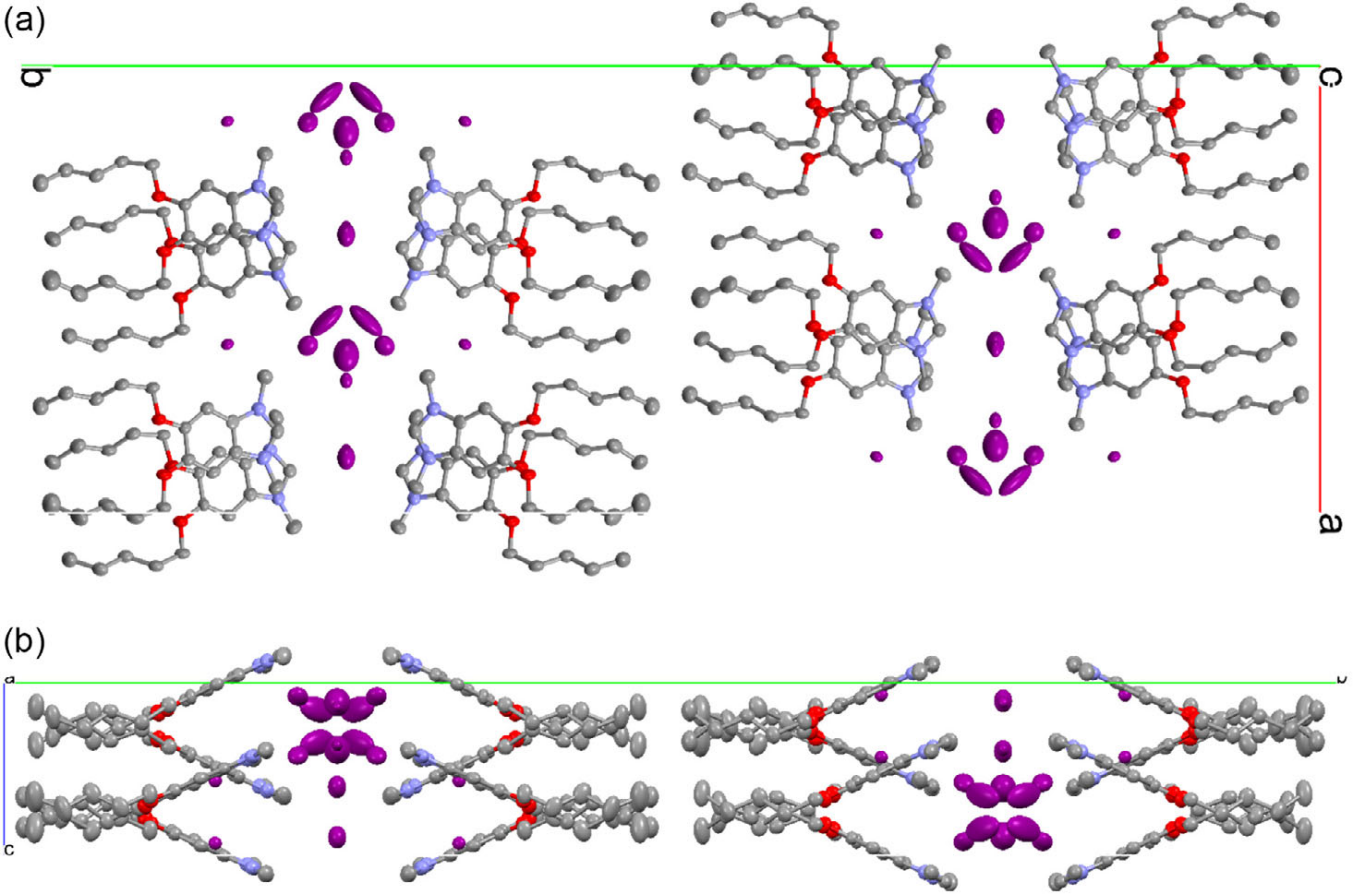
Illustration of the packing of (a) **U‐Me**_**2**_ **Bi(5)I** view along the *c*‐axis and (b) **U‐Me**_**2**_ **Bi(5)I** view along the *a*‐axis. Displacement ellipsoids at 50% probability level; hydrogen atoms omitted; color code: O red, C gray, N blue; I purple. Further data are listed in the ESI, Section 5.

The results demonstrated that a flat aromatic core structure with alkoxy side chains does not necessarily lead to a flat conformation. It should be noted that bent solid‐state structures have been reported for *N*,*N‐*dialkylbenzimidazolium salts [25, 46], whereas flat solid state structures were reported for Ag(I) NHC complexes carrying *N*,*N‐*dialkylbenzimidazolium salts as NHC ligands [29, 42].

### 2.2 Mesomorphic Properties

The phase behavior of benzimidazole derivatives was studied by differential scanning calorimetry (DSC) and polarizing optical microscopy (POM). Typical DSC curves of liquid crystalline derivatives are shown exemplary for the *n* = 12 derivatives in Figure 3. DSC curves of all derivatives are shown in the ESI, Section 3.2. and corresponding data are shown in Tables S1–S4. For the *N*‐methylated **L‐Me**_**2**_ **Bi(n)** and **U‐Me**_**2**_ **Bi(n)**, no mesomorphism could be found. The general trend found was that the neutral linear derivatives showed mesomorphic properties only upon cooling, whereas their ionic counterparts did not display any liquid crystallinity at all. For the U‐shaped derivatives, exactly the opposite behavior was observed; neutral derivatives lacked mesomorphic properties, while ionic derivatives showed formation of a mesophase. For instance, upon second heating the linear derivative **L‐Bi(12)** showed an endothermic Cr → Cr transition at 53 °C and an endothermic melting transition at 59 °C (Figure 3a). Upon subsequent cooling, an exothermic isotropic to mesophase transition at 51 °C followed by an exothermic crystallization at 42 °C was detected. The small enthalpy of 2.2 kJ/mol for the I → M transition already suggested the formation of a monotropic nematic phase, which was later confirmed by POM and X‐ray diffraction (XRD) (see below). The second heating curve of the corresponding U‐shaped derivatives **U‐Bi(n)** showed none to two endothermic crystal‐to‐crystal transitions, followed by a clearing transition (Figures S11 and S12). Upon subsequent cooling, crystallization from the isotropic phase occurred, followed by one or multiple exothermic crystal‐to‐crystal transitions.

**FIGURE 3:**
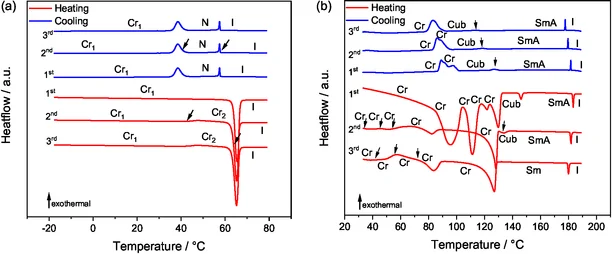
Exemplary DSC thermograms of (a) **L‐Bi(12)** and (b) **U‐Me**_**2**_ **Bi(12)I** heating rate: 5 K/min; cooling rate: 2 K/min. As the different crystalline phases were not investigated in detail within this work and due to several overlapping crystalline phase transitions, no numerical assignment of the crystalline phases was made.

The DSC curves of the linear benzimidazolium salts **L‐Me**_**2**_ **Bi(n)I** revealed only endothermic Cr → Cr transitions and isotropic melting in the first heating cycle (Figures S14 and S15), which appeared in reverse order in the first cooling cycle. Please note that ionic derivatives showed decomposition to a certain extent in corresponding thermograms, which is why the first heating and cooling cycles were considered for samples, where decomposition could hardly be suppressed. In contrast, the U‐shaped benzimidazolium salts **U‐Me**_**2**_ **Bi(n)I** showed a unique polymorphism (Figures S17 and S18). **U‐Me**_**2**_ **Bi(12)I** displayed several crystal‐to‐crystal transitions upon second heating (Figure 3), representative behavior for almost all other derivatives of this series. These transitions are followed by an endothermic melting transition at 124 °C into a mesophase that was later assigned as most probably cubic *Im3m* for **U‐Me**_**2**_ **Bi(12)I** (see below). At 130 °C, a mesophase (cub) to mesophase (SmA) transition was found, followed by an endothermic melting at 181 °C. Upon cooling in the first cooling cycle, an exothermic isotropic to smectic transition at 182 °C and a smectic to cubic transition at 131 °C followed by crystallization at 102 °C as well as a crystal to crystal transition at 96 °C were observed. The other derivatives only revealed one enantiotropic mesophase (SmA) with a transition from crystal to mesophase at around 140 °C for all derivatives with *n* = 9, 10, and 12 (Figure S18). The ionic derivative **U‐Me**_**2**_ **Bi(14)I** revealed a similar pattern in thermograms differing in the determined mesophase, which was identified by POM and XRD to be of hexatic smectic nature (see below).

POM observations of linear benzimidazoles **L‐Bi(n)** with chain lengths *n* ≥ 7 revealed Schlieren textures upon cooling, which are typical for nematic (N) phases. An illustrative example is shown in Figure 4a for **L‐Bi(12)** at 55 °C. In the heating cycle, no textures were visible, further confirming the monotropic nature of the mesophase. In contrast, for the U‐shaped benzimidazoles **U‐Bi(n)** only crystalline non‐shearable textures were observed in both heating and cooling cycles, e.g., for **U‐Bi(14)** at 90 °C (Figure S12).

**FIGURE 4:**
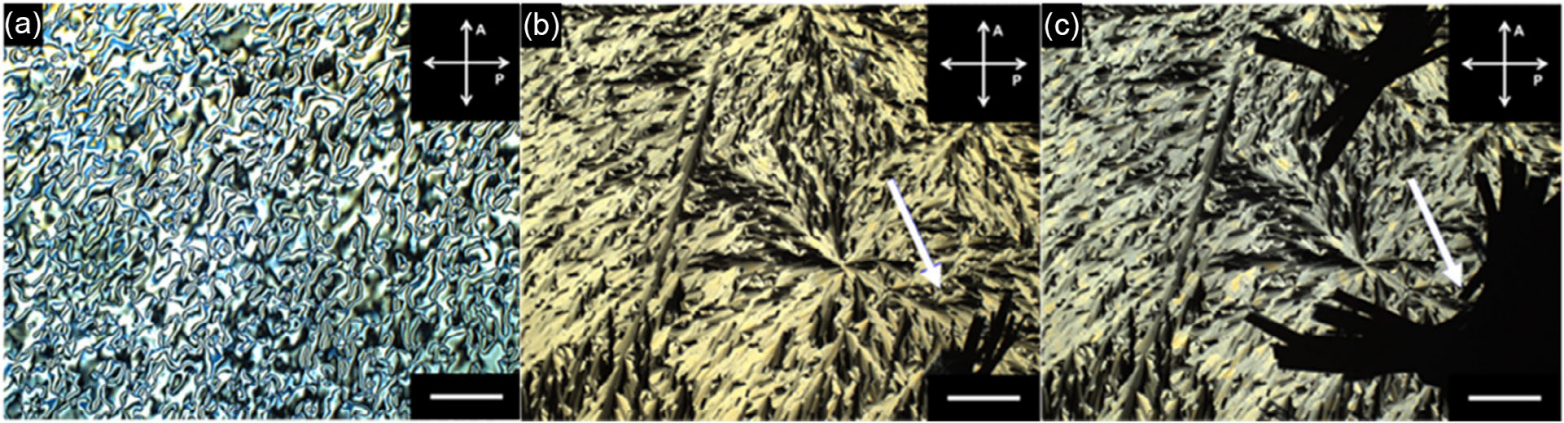
Microscopic pictures of (a) characteristic nematic Schlieren textures of linear benzimidazoles **L‐Bi(12)**, 55.1 °C. (b) Fan‐shaped textures of a smectic phase of U‐shaped benzimidazole‐iodide **U‐Me**_**2**_ **Bi(12)I**, 110.6 °C and (c) 105.0 °C after 135 s. The white arrow serves as orientation for the development of the optically isotropic texture of the cubic phase; all POM textures observed in the cooling cycle. The scale bar is 200 µm.

All members of the linear benzimidazolium iodide series **L‐Me**_**2**_ **Bi(n)I** displayed only crystalline grainy textures in both heating and cooling cycles, confirming the absence of liquid crystalline phases (Figure S3). For members of the U‐shaped benzimidazolium iodide series **U‐Me**_**2**_ **Bi(n)I** with *n* = 6, 7, 8, 9, and 10, textures with homeotropic areas were visible under the POM upon cooling, hinting toward a SmA phase. A unique phase behavior was observed for the derivative with chain length *n* = 12. Upon cooling from the isotropic liquid compound, **U‐Me**_**2**_ **Bi(12)I** displayed a fan‐shaped texture between 170 °C and 110 °C (Figure 4b). Upon further cooling, at 100 °C the texture changed to an optically isotropic texture, which remained stable regardless of the viewing angle and was not altered by mechanical shearing (Figure 4c). This optically isotropic texture is characteristic for a cubic phase [47]. The optically isotropic texture was maintained until crystallization began below 81 °C. The assignment of a cubic phase was further supported by the very small exothermic SmA → Cub transition enthalpy (Δ*H* = −0.6 kJ/mol) detected in the DSC cooling curve (Figure 3b, Table S4). Upon further cooling, the sample crystallized. Derivative **U‐Me**_**2**_ **Bi(14)I** with even longer alkyl chains showed no cubic phase but shearable fan‐shaped textures from 135 °C up to 170 °C upon heating as well (Figure S5). Upon cooling, crystallization was observed below 89 °C. The lack of homeotropic areas and noticeably less fluidity in the mesophase compared to the other SmA phases indicate another kind of smectic phase instead of a SmA phase [47]. POM textures of all derivatives are shown in Section 3.1, ESI.

The investigation of the four series via POM and DSC clearly showed that only two of them displayed mesomorphic behavior: the nonionic **L‐Bi(n)** and the ionic **U‐Me**_**2**_ **Bi(n)I** series. The exact phase transition temperatures and enthalpies for all four series can be found in the ESI, Section 3.2.

The compounds of the two series which showed mesomorphic properties were investigated with small‐angle X‐ray scattering (SAXS). Figure 5 shows the SAXS curves of the nematic and the isotropic phases of mesogen **L‐Bi(10)**. For the three other **L‐Bi(n)** homologues with nematic phases with *n* = 9, 12, and 14, it was not possible to get an X‐ray scattering curve due to the monotropic nature of these phases. **L‐Bi(10)** shows a diffuse scattering peak in the small‐angle regime in the isotropic phase at *q* = 3.7 nm^−1^. This corresponds to a *d*‐value of 17.1 Å. It is clearly shown in Figure 5 that upon cooling from the isotropic phase, the nematic phase is formed with a higher *q*‐value of 4.5 nm^−1^, corresponding to a *d*‐value of 13.7 Å. This nematic phase could only be observed in cooling as it is monotropic.

**FIGURE 5:**
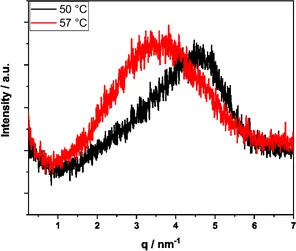
XRD diffractogram of the scattering vector *q* versus the intensity *I* during cooling cycle for **L‐Bi(10)** in the isotropic phase at 57 °C (red) and in the nematic phase at 50 °C (black). For both phases, broad small‐angle peaks are observed. Upon cooling in the nematic phase, the peak becomes slightly narrower and is now found at higher *q*‐values.

The maximum of the broad nematic scattering peak is compared with the molecular lengths of **L‐Bi(10)** (determined with Chem3D [48]) of 26.2 Å. It is obvious that the scattering maximum is much smaller than the molecular length, indicating a significant degree of conformational and orientational molecular disorder.

The other series which was investigated with SAXS was the **U‐Me**_**2**_ **Bi(n)I** series. All ionic samples suffered from slight decomposition, leading to an additional reflection at *q*‐values below the (001) reflection in the recorded diffractograms, and thus, this peak was not considered for the determination of the mesophase geometry. The homologues with *n* = 5 and 6 showed monotropic smectic A phases, while the homologues with *n* = 7–12 showed enantiotropic smectic A phases. Furthermore, a cubic phase could be observed for **U‐Me**_**2**_ **Bi(12)I**, as well as a more ordered hexatic smectic type of phase for the homologue **U‐Me**_**2**_ **Bi(14)I**. Table 1 summarizes the lattice parameters of the observed phases. The layer spacing of the smectic A phase increases with increasing chain length *n*.

**TABLE 1:** XRD data and molecular lengths of the U‐Me_2_Bi(n)I series.

**U‐Me**_**2**_ **Bi(n)I**						
*n*	Mesophase	Lattice parameter, Å	*d*, Å	Miller indices	*L*_calc_, Å	Ratio(*d*/*L*_calc_)
6	SmA at 124 °C	26.3	26.3	(001)	13.1	2.0
			13.2	(002)		
7	SmA at 148 °C	27.3	27.3	(001)	14.2	1.9
			13.6	(002)		
8	SmA at 153 °C	28.4	28.4	(001)	15.5	1.8
9	SmA at 144 °C	30.4	30.4	(001)	16.7	1.8
			15.2	(002)		
10	SmA at 144 °C	31.2	31.2	(001)	18.0	1.7
12	Cub at 116 °C	119.5	84.5 48.4 37.0 34.7	(110) (211) (310) (222)	—	—
12	SmA at 135 °C	33.3	33.3	(001)	20.5	1.6
14	SmX	29.3	29.3	(001)	23.0	1.3
			19.6	(002)		
			9.9	(003)		

For a better understanding of the molecular structure of the mesophases, the molecular lengths (estimated with Chem3D [48], for more details, see Figure S25) of the **U‐Me**_**2**_ **Bi(n)I** series were compared with the layer peaks of the smectic phases (Table 1).

The layer thickness for the observed SmA phases was in the range from 2 (for *n* = 6) to 1.6 (for *n* = 12) times of the molecular lengths. This indicates that the molecules formed double‐ or partial‐double layers. There was a strong nanosegregation between the ionic and the nonionic part of the molecules, forming alternating ionic and nonionic sub‐layers. For the shortest homologue with *n* = 6, there was only a very small amount of interdigitation between the alkyl tails from one layer to the next. This led to the formation of full double layers. With increasing chain length, this amount of interdigitation increased steadily, and in the case of *n* = 12, partial double layers of 1.6 times the molecular length with a higher degree of interdigitation were formed. This nanosegregation and formation of double layers is a quite typical behavior for ionic smectic A phases [1, 2, 3, 49].

Figure 6 shows the SAXS profiles *I*(*q*) of **U‐Me**_**2**_ **Bi(6)I** and **U‐Me**_**2**_ **Bi(12)I**, which are representative for the **U‐Me**_**2**_ **Bi(n)I** series. For further details, see Figure S22, ESI.

**FIGURE 6:**
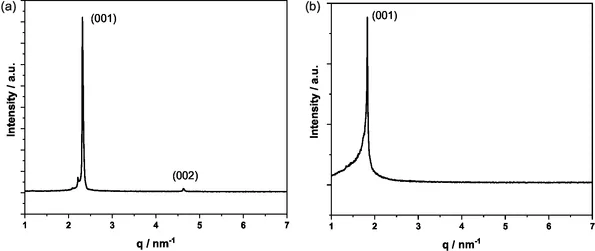
Exemplary XRD diffractograms of the smectic A phase of **U‐Me**_**2**_ **Bi(n)I** with (a) *n* = 6 at 124 °C and (b) *n* = 12 at 135 °C. The measurements show a sharp and strong (001) smectic layer peak; furthermore, for some of the homologues additionally the higher order (002) layer peak was observed. The sharp layer peak together with a diffuse halo (Figure S20) indicates that these mesogens form SmA phases.

All compounds exhibit a (001) layer peak typical for smectic phases. Furthermore, in some of the homologues also the (002) peak of the smectic layers was observed. For **U‐Me**_**2**_ **Bi(12)I**, it was possible to receive a 2D‐X‐ray diffractogram (Figure S20) over the small and wide‐angle regime. It showed a diffuse wide‐angle halo, indicating a fluid smectic phase like a SmA or SmC phase. Together with optical texture observations (see Figure 4b and ESI, Section 3.1), this leads to the conclusion that these **U‐Me**_**2**_ **Bi(n)I** mesogens form SmA phases.

The small‐angle X‐ray diffractogram of **U‐Me**_**2**_ **Bi(12)I** is shown in Figure 7. The diffractogram shows reflections that can be assigned to the (110), (211), (310), and (222) reflection of a cubic phase. This results in a cubic phase with the space group *Im3m* to be the most likely mesophase geometry. The reflections could be indexed according to a cubic lattice with the cubic lattice parameter *a* = 119.5 Å.

**FIGURE 7:**
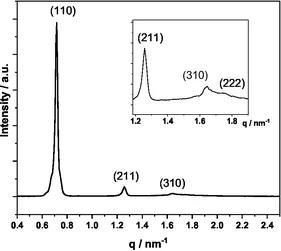
SAXS diffractograms of the cubic phase of **U‐Me**_**2**_ **Bi(12)I** at 116 °C. This phase shows the typical peaks of a cubic phase. This leads to a cubic lattice parameter of *a* = 119.5 Å.

For the **U‐Me**_**2**_ **Bi(14)I** homologue, SAXS and WAXS were recorded. Please note that the corresponding DSC thermogram (Figure S18d) shows pronounced decomposition. Therefore, acquisition times during XRD experiments had to be kept to a minimum in order to achieve interpretable measurements that describe the mesophase observed by POM. Both diffractograms showed a sharp (001) reflection accompanied by less intense (002) and (003) reflections (Figure 8). Additionally, a halo could be observed in the wide‐angle regime. Rather than displaying a purely diffuse halo, two more reflections could be found at *q* = 11.84 nm^−1^ and *q* = 14.32 nm^−1^, marked with an arrow in Figure 8, respectively. The occurrence of such additional reflections in the wide‐angle region is typical for both hexatic smectic phases SmI and SmF [50]. The smectic layer distance was determined to be 29.3 Å for this derivative, leading to a ratio between the layer spacing and the molecular length of only 1.3, which is significantly smaller than for the smectic A phases. This decrease can be explained by the existing tilt within a SmF or SmI phase, leading to a smaller layer distance compared to the SmA phase, which might serve as another experimental evidence for the presence of a hexatic SmX (i.e., either SmI or SmF) phase. Unfortunately, we were not able to get an aligned 2D wide‐angle X‐ray diffractogram. Due to the instability of the sample at higher temperatures, it was not possible to receive an aligned 2D‐X‐ray diffractogram. Therefore, we were not able to assign the two marked reflections in the wide‐angle regime as both possible hexatic phases show reflections in this approximate area. Ultimately, it was not possible to distinguish between SmF and SmI.

**FIGURE 8:**
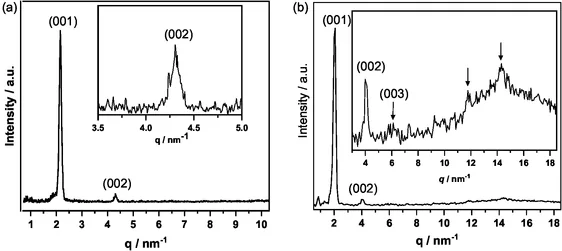
(a) SAXS and (b) WAXS diffractogram of the SmX phase of **U‐Me**_**2**_ **Bi(14)I** at 131 °C. Distinct (001) and additional (002) and (003) reflections can be found in the small‐angle regime, as well as two further reflections in the halo region marked with an arrow. These two reflexes characterize both the SmI and SmF phases.

In the case of the nonionic molecules, mesomorphic behavior was only found for the rod‐shaped linear homologues as the driving force to form nematic phases here is the orientational order of the rod‐shaped molecules. The resulting monotropic mesophase and phase widths for the series are shown in Figure 9a.

**FIGURE 9:**
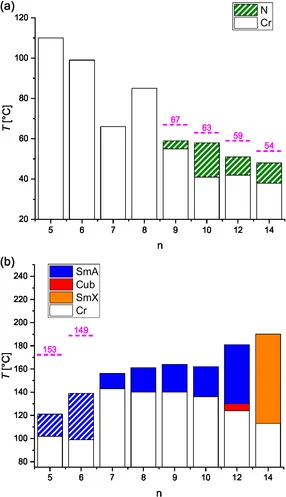
(a) Phase ranges for the **L‐Bi(n)** series and transition temperatures during cooling cycle with a cooling rate of 2 K · min^−1^. Monotropic nematic mesophases were observed for *n* = 9, 10, 12, and 14 and their melting points are given as pink dotted lines were determined from the second heating cycle with a heating rate of 5 K · min^−1^. (b) Phase ranges for the **U‐Me**_**2**_ **Bi(n)I** series and transition temperatures during heating cycle with a heating rate of 5 K · min^−1^. All compounds showed a smectic phase which in the case of *n* = 5 and 6 appeared only on cooling with a cooling rate of 2 K · min^−1^ and was therefore monotropic. Their melting points are given as pink dotted lines and were determined from the first heating cycle with a heating rate of 5 K · min^−1^. The *n* = 12 homologue showed an additional cubic phase, whereas for *n* = 14, a hexatic smectic phase SmX was found. Cr: crystalline phase, N: nematic, SmA: smectic A phase, Cub: cubic phase, SmX: hexatic smectic phase. Monotropic phases are marked with white stripes and were determined from the cooling cycle with a cooling rate of 2 K · min^−1^. Their reported mesophases have been determined from cooling cycles, which lead to monotropic transition temperatures. To display the data with as little loss of information as possible, their thermodynamic transition temperatures from the crystalline to the isotropic phase determined from the heating cycles are given additionally as pink lines.

The bent U‐shaped homologues cannot form mesophases as they cannot orient parallel to each other. In the case of the ionic molecules, the driving force for the formation of mesophases is the positional order emerging from the segregation between ionic and nonionic parts of the molecules. The U‐shaped homologues where the charge is located at the end of the molecules can nanosegregate and form layers and, therefore, build smectic phases. For the linear homologues with the charge in the middle of the molecules, this is not possible and therefore no mesophases are observed. Therefore, the ionic U‐shaped series showed mostly smectic A phases. For the C_12_ homologue additionally, a cubic phase was found, and for benzimidazolium salt **U‐Me**_**2**_ **Bi(14)I**, a hexatic smectic phase SmX like SmF or SmI was assigned due to slightly different fluid behavior and the lack of homeotropic areas during POM observations. The phase widths as well as the observed mesophase geometries are shown in Figure 9b.

## 3 Conclusion

In this work, we investigated structure–property relationships governing the phase behavior of neutral and ionic dialkoxybenzimidazoles, with a focus on the influence of molecular symmetry and charge distribution on liquid crystalline self‐assembly. Therefore, a series of linear and U‐shaped neutral benzimidazoles **L‐Bi(n)** and **U‐Bi(n)** (*n* = 5–14) and their ionic analogues (benzimidazole iodides) **L‐Me**_**2**_ **Bi(n)I** and **U‐Me**_**2**_ **Bi(n)I** (*n* = 5–14) were synthesized to systematically probe these effects. Differences between the neutral and ionic benzimidazoles are already evident in their solid‐state structures. While the neutral benzimidazoles arrange via hydrogen bonding into a perfect lamellar structure of parallel layers, the ionic benzimidazoles form layers through offset π‐stacking. These layers are oriented at a twist angle of approximately 90° relative to each other. However, detailed characterization of the mesophase properties by determined by POM, DSC, and XRD revealed that the solid‐state structures do not allow an easy correlation with the mesomorphic properties. The neutral rod‐like linear benzimidazoles **L‐Bi(n)** with longer alkoxy side chains (*n* = 9–14) form nematic phases driven by oriental order mesomorphism, whereas bent U‐shaped benzimidazoles **U‐Bi(n)** do not show any mesomorphism. In contrast, ionic benzimidazole iodides **L‐Me**_**2**_ **Bi(n)I**, **U‐Me**_**2**_ **Bi(n)I** are governed by positional order: **U‐Me**_**2**_ **Bi(n)I** with terminal charges exhibit nanosegregation and form layered smectic A phases, including a cubic phase for **U‐Me**_**2**_ **Bi(12)I**, while linear benzimidazolium iodides **L‐Me**_**2**_ **Bi(n)I** with centrally located charges do not from mesophases despite their lamellar order in the solid state. Overall, the study highlights how variations on the molecular architecture in dialkoxybenzimidazoles—particularly in symmetry and charge—enable control over mesophase formation. As benzimidazole derivatives are important precursors for metal complexes, future work must demonstrate how changes of symmetry and charge of the benzimidazole ligand might affect the liquid crystalline self assembly of the resulting metallomesogen.

## Author Contributions

E.W. synthesized and characterized all compounds and performed POM and DSC measurements. N.S. and S.S. performed POM and XRD measurements. W.F. and J.O. performed the single‐crystal X‐ray diffraction (SC‐XRD) determinations. N.K. evaluated the XRD data, wrote the XRD section of this manuscript, and provided her expertise regarding LCs. S.S. revised the discussion of the LC characterization. F.G. provided his expertise regarding LCs. A.T. provided his expertise on ILCs. N.S., S.S., E.W., S.L., and A.Z. checked the data and helped with the manuscript preparation. S.L., N.S., S.S., N.K. and A.Z. wrote the manuscript; S.L. coordinated the research.

## Conflicts of Interest

There are no conflicts to declare.

## Supporting information

The supporting information contains the experimental procedures, nuclear magnetic resonance spectra, DSC, POM, XRD, and SC‐XRD data.
