# Supplementary material for: Tailoring Liquid Crystalline Self‐Assembly in Dialkoxybenzimidazoles: The Role of Charge and Shape
Source: Chemphyschem. 2026 Oct 22;27(20):e70588. doi: 10.1002/cphc.70588 (PMC13691409; doi:10.1002/cphc.70588)
Supplement: Supplementary file 1 [file CPHC-27-e70588-s001.pdf]

Electronic Supporting Information (ESI)

Tailoring Liquid Crystalline Self-Assembly in Dialkoxybenzimidazoles: The Role of Charge and Shape

Eugen Wuckert,[a] Nikolai Scheuring,[b,c] Sara Simonovska,[a] Nadia Kapernaum,[b] Wolfgang Frey,[a] Johanna Osterbrink,[a] Andreas Taubert,[d] Frank Gießelmann,[b] Anna Zens,[a] Sabine Laschat*[a]

[a] *Institut für Organische Chemie, Universität Stuttgart, Pfaffenwaldring 55, D-70569 Stuttgart, Germany, sabine.laschat@oc.uni-stuttgart.de*
[b] *Institut für Physikalische Chemie, Universität Stuttgart, Pfaffenwaldring 55, D-70569 Stuttgart, Germany*
[c] *Department of Chemistry, Simon Fraser University, Burnaby, BC V5A 4Y8, Canada*
[d] *Universität Potsdam, Institut für Chemie, Lehrstuhl für Supramolekulare Chemie, Karl-Liebknecht-Straße 24-25, D-14476 Potsdam, Germany*

Table of Contents

1. General Methods 2
   1.1 Synthesis 2
   1.2. Mesomorphic properties 3
2. Synthesis 4
   2.1. General Synthetic Procedures (GP) 4
   2.2. Syntheses of Dinitrodialkoxybenzenes (U-Nitro(n), L-Nitro(n)) 6
   2.3. Syntheses of U-shaped benzimidazoles (U-Bi(n)) 19
   2.4. Syntheses of linear benzimidazoles (L-Bi(n)) 25
   2.5. Syntheses of 5,6-bis(alkoxy)-1-methyl-benzoimidazole (U-MeBi(n)) 33
   2.6. Syntheses of 4,7-bis(alkoxy)-1-methyl-benzoimidazole (L-MeBi(n)) 40
   2.7. Syntheses of 1,3-dimethyl-5,6-bis(alkoxy)-benzoimidazolium iodides (U-Me_2Bi(n)I) 48
   2.8. Syntheses of 1,3-dimethyl-4,7-bis(alkoxy)-benzoimidazolium iodides (L-Me_2Bi(n)I) 52
3. Mesomorphic Properties 59
   3.1. POM Results 59
   3.2. DSC Results 64
   3.3. XRD Results 81
4. NMR spectra 85
5. SC-XRD 149
6. Literature 163

1. General Methods

1.1 Synthesis

Methods and Chemicals:

If not indicated otherwise, all chemicals were commercially available and have been used without further purification. Anhydrous THF was obtained from a MBraun SPS-800 solvent purification system (SPS) after being dried through active alumina columns. Petroleum ether, EtOAc and CH_2Cl_2 were distilled prior to use.

Thin layer chromatography (TLC):

Reaction monitoring and/or column chromatography was performed on TLC plates Alugram® Xtra SIL G/UV_{254} from Macherey-Nagel as stationary phase with the indicated solvent mixture as mobile phase. Substance spots were identified by illuminating the plates with UV light (254 nm and 366 nm) or by immersing the plate in one of the following staining solutions followed by heating to 300 °C:

Cerium molybdate: $Ce(NH_4)_4(NO_3)_6$ (0.5 g) and $(NH_4)_6Mo_7O_{24} \cdot 4\,H_2O$ (12 g) were dissolved in deionized H_2O (200 mL) and acidified with conc. H_2SO_4 (28 mL).

$KMnO_4$: $KMnO_4$ (0.375 g), K_2CO_3 (2.5 g) and NaOH (0.1 g) were dissolved in demineralized H_2O (50 mL).

Nuclear magnetic resonance spectroscopy:

^{1}H NMR spectra were recorded at frequencies of 400/ 500/ 700 MHz, broadband decoupled ^{13}C NMR spectra were recorded at frequencies of 100/ 125/ 176 MHz on Bruker AVANCE 300 / ASCEND 400 / AVANCE 500 / AVANCE 700 spectrometers, respectively. Chemical shifts were given in parts per million (ppm) and referenced to the peak of the deuterated solvents if not indicated otherwise. Multiplicities were given in Hz and abbreviated as following: singlet (s), doublet (d), triplet (t), quartet (q), multiplet (m). COSY, HSQC, HMBC and NOESY spectra were utilized to assign the signals to the corresponding atoms.

Mass spectrometry:

High resolution mass spectra (HRMS) were measured on an Exactive Plus Orbitrap MS Thermo Fisher Scientific System or an Exactive GC Orbitrap MS Thermo Fisher Scientific System

Infrared Spectroscopy:

Infrared spectra were measured in solution on a Vektor 22 spectrometer by Bruker with a MKII Golden Gate Single Reflection Diamant ATR-System. The intensities of the absorption bands were classified as strong (s) and weak (w).

1.2. Mesomorphic properties

Differential Scanning Calorimetry (DSC):

DSC measurements were performed on a DSC822^{e} by Mettler Toledo in standardized 40 µL aluminum crucibles and evaluated with the software STARe 14.0. Phase transition temperatures are given as onsets of the corresponding peaks.

Polarizing Optical Microscopy (POM):

A polarizing optical microscope Olympus BX50 equipped with a variable temperature sample holder LTS350 (control unit: TP39 and LNP, ΔT = ± 1 K) by Linkam Scientific was used to investigate the mesomorphic properties of our compounds. Micrographs were taken with a Zeiss Axiocam 105 color camera module and the software ZEN core. The samples were investigated on regular glass slides and in polyimide coated cells.

Small-angle (SAXS) X-ray diffraction:

Small-angle X-ray scattering (SAXS) experiments were performed at 25 °C with an Anton Paar SAXSess mc2 equipped with a Dectris Mythen 1 K detector. Cu Kα radiation was generated with an ID 3003 X-ray generator (Seifert) operated at 40 kV and 40 mA and line collimated. The sample to detector distance was calibrated using a sample of powdered cholesteryl palmitate.

Small-angle (SAXS) of U-Me$_2$Bi(14)I and Wide-angle (WAXS) X-ray diffraction:

WAXS measurements and SAXS measurement of **U-Me$_2$Bi(14)I** were performed on a Bruker AXS Nanostar C equipped with a Siemens ceramic tube generator (CuK$_{\alpha}$ radiation, λ = 1.5405 Å, 1500 W). A Bruker HI-STAR detector was used and the resulting measurement calibrated against the diffraction pattern of silver behenate at 25°C. Samples were measured in sealed capillaries from Hildenberg GmbH with an external diameter of 0.7 mm. As evaluation software SAXSoffline from Bruker and Origin 2025b from OriginLab were used.

SC-XRD:

Crystals suitable for single crystal X-ray diffraction were taken from the parent solution and immersed instantaneously into perfluorinated polyether oil and fixed on a cryo loop. Full shells of intensity data were collected at low temperature with a Bruker Kappa APEXII Duo or a Rigaku XtaLAB Synergy DW diffractometer. The obtained data was processed using the standard Bruker or Rigaku software package (SAINT, APEX2 or APEX6, CrysalisPro).[1] Multiscan absorption correction was performed with the

SADABS program or Empirical absorption correction using spherical harmonics,implemented in SCALE3 ABSPACK scaling algorithm was used.[2] Structure solving was achieved by intrinsic phasing [2–4] and refinement with the SHELXTL software package (Version 2014/7 or 2019/3).[4–7] The structures were graphically handled with OLEX2 during solution and refinement.[8]All non-hydrogen atoms received anisotropic displacement parameters. Hydrogen atoms were generally placed at calculated positions and refined with a riding model. The visualization of the structures was performed using Mercury 2025.3.3. Displacement ellipsoids are depicted with 50 % probability. Crystallographic data for the structures reported in this paper have been deposited in the Cambridge Crystallographic

Data Centre (Deposition Numbers 2532065 for ***o*-Bi(14)**, 2532063 for ***p*-Bi(6)**, 2532062 for ***o*-Nitro(12)**, 2532061 for ***o*-Me$_2$Bi(9)I** and 2532066 for ***p*-Me$_2$Bi(5)I**. These data can be obtained free of charge from The Cambridge Crystallographic Data Centre via www.ccdc.cam.ac.uk/data_request/cif.

2. Synthesis

2.1. General Synthetic Procedures (GP)

GP1: Syntheses of 1,2-dinitro-4,5-bis(alkyloxy)benzenes (U-Nitro(n) (n = 5 – 10, 12, 14))[9]

Bis(alkyloxy)benzene was dissolved in CH_2Cl_2 and added dropwise to HNO_3 (100 %, cooled to 0°C), for at least 15 min. The reaction mixture was stirred at 0°C for 30 min, then H_2SO_4 (96 %) was slowly added. The ice was removed and stirred for 3 h at room temperature, then the resulting reaction mixture was poured over ice water. The aqueous phase was extracted with CH_2Cl_2 (3 x 100 mL) and the combined organic phases were dried over $MgSO_4$ and concentrated in vacuo. After recrystallization (from EtOH (only for R = C_5H_{11}) or from MeOH), the desired 1,2-dinitro-4,5-bis(alkyloxy)benzenes **U-Nitro(n)** (n = 5 – 10, 12, 14) were obtained as solids. Specific quantities, conditions, and analytical data for each individual compound are provided below.

GP2: Syntheses of of 1,2-dinitro-4,5-bis(alkyloxy)benzenes (L-Nitro(n) (n = 5 – 9))[10]

To a cooled solution (15 °C) of 1,2-bis(alkyloxy)benzene a mixture of conc. AcOH and CH_2Cl_2, HNO_3 (65 %) was added dropwise, maintaining the temperature below 40 °C. The reaction mixture was stirred for 30 min, then cooled again to 15 °C, and fuming nitric acid (100 %) was added slowly. The mixture was stirred at ambient temperature for 72 h. Upon completion, the reaction mixture was poured into ice water (250 mL), and the organic layer was separated and washed successively with water (3 × 150 mL), saturated aqueous sodium bicarbonate (150 mL), and brine (150 mL). The organic phase was dried over anhydrous $MgSO_4$, and the solvent was removed under reduced pressure. After purification, **L-Nitro(n)** (n = 5 – 9) was obtained as solids. Specific quantities, conditions, purification methods and analytical data for each individual compound are provided below.

GP3: Syntheses of 1,4-bis(alkyloxy)-2,3-dinitrobenzene (L-Nitro(n) (n = 10, 12, 14))[11]

To HNO_3 (100 %) cooled to 0 °C, a viscous suspension of 1,4-bis(alkyloxy)benzene in conc. AcOH (50 mL) was added portion wise under stirring.[11] The reaction mixture was stirred for 1 h at 0 °C, followed by 1 h at room temperature, and then heated to 70 °C for an additional hour. After completion, the reaction mixture was poured onto ice, and the resulting precipitate was collected by filtration. The solid was washed sequentially with saturated aqueous sodium bicarbonate and water, then dissolved in a mixture of petroleum ether / ethyl acetate (100 : 1). The organic layer was dried over anhydrous $MgSO_4$, filtered, and concentrated under reduced pressure. After purification **L-Nitro(n)** (n = 10, 12, 14) could be obtained as yellow crystals. Specific quantities, conditions, purification methods and analytical data for each individual compound are provided below

GP4: Syntheses of bis(alkoxy)-benzoimidazoles (U-Bi(n) and L-Bi(n) n = 5-9, 10, 12, 14))[12,13]

1,2-dinitro-4,5-bis(alkyloxy)benzenes **U-Nitro(n) or** 1,4-bis(alkyloxy)-2,3-dinitrobenzene **L-Nitro(n)** was dissolved in tetrahydrofuran (THF), and 10% palladium on carbon was added. The reaction mixture was hydrogenated by introducing H_2 gas over a period of time. The reaction mixture was purged with a N_2 for 10 min, after which formic acid (100 %) was added. The reaction temperature was raised up (see below at each compound), and the mixture was stirred at this temperature. After cooling to rt, the mixture was filtered through a pad of Celite®. The solvent and excess formic acid were removed under reduced pressure. The resulting crude product was suspended in diethyl ether (200 mL), water (100 mL), and 20% aqueous ammonium hydroxide solution (5 mL) and stirred at room temperature for 30 min. The organic phase was separated and washed with water (2 × 100 mL), then dried over $MgSO_4$. After filtration, the solvent was removed under reduced pressure. Final purification steps, specific quantities, conditions, and analytical data for each individual compound are provided below.

GP5: Syntheses of bis(alkoxy)-1-methyl-benzoimidazoles (U-MeBi(n) or L-MeBi(n), n = 5-9, 10, 12, 14))[12,13]

U-Bi(n) or **L-Bi(n)** was dissolved in THF, and NaH (60 % dispersion in mineral oil) was added. Then, MeI was added dropwise, and the reaction mixture was stirred at room temperature for 1 h. After workup and purification, the product **U-MeBi(n)** or **L-MeBi(n)** was obtained as a solid. Specific quantities, conditions, workup and purification methods as well as analytical data for each individual compound are provided below.

GP6: Syntheses of 1,3-dimethyl-5,6-bis(alkoxy)-benzoimidazolium iodides (U-Me$_2$Bi(n)I or L-Me$_2$Bi(n)I, n = 5-9, 10, 12, 14))[12,13]

U-MeBi(n) or **L-MeBi(n)** was dissolved in THF and MeI was added. The reaction mixture stirred at 50 °C overnight. After cooling the reaction mixture to 0 °C, the resulting solid was collected by vacuum filtration, washed with cold THF (3 x 100 mL), and dried under reduced pressure. After recrystallization

(from THF) the desired iodides could be obtained. Specific quantities, conditions, and analytical data for each individual compound are provided below.

2.2. Syntheses of Dinitrodialkoxybenzenes (U-Nitro(n), L-Nitro(n))

1,2-Dinitro-4,5-bis(pentyloxy)benzene (U-Nitro(5)):

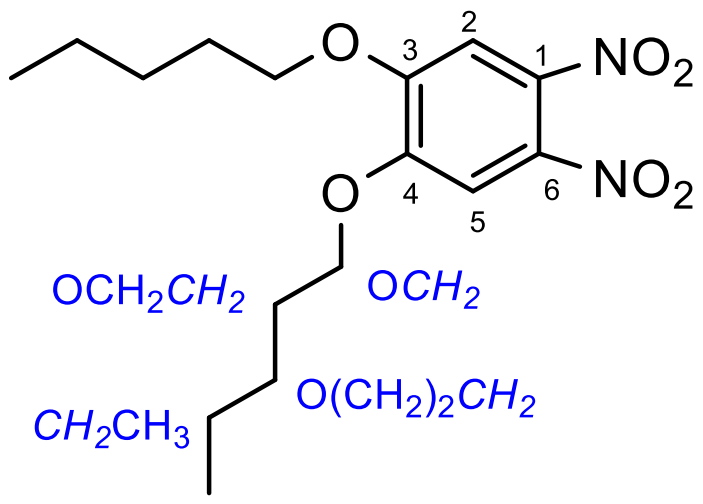

U-Nitro(5)

$C_{16}H_{24}N_2O_6$
(340.16 g mol^{-1})

According to **GP1**, 1,2-bispentyloxybenzene (4.05 g, 16.2 mmol), HNO_3 (100 %, 4.00 mL, 97.0 mmol), H_2SO_4 (96 %, 3.60 mL, 64.7 mmol) in 44 mL CH_2Cl_2; **U-Nitro(5)** as yellow crystals: 5.08 g (14.7 mmol, 91 %); ^{1}H NMR (500 MHz, $CDCl_3$): δ = 0.94 (t, *J* = 7.2 Hz, 6H, 2 x CH_3), 1.37-1.50 (m, 8H, 2 x CH_3*CH_2*CH_2*), 1.85-1.90 (m, 4H, 2 x OCH_2*CH_2*), 4.10 (t, *J* = 6.5 Hz, 4H, 2 x OCH_2), 7.29 (s, 2H, 2-H, 5-H) ppm; ^{13}C NMR (125 MHz, $CDCl_3$): δ = 14.1 (2 x CH_3), 22.5 (2 x CH_3*CH_2*), 28.1 (2 x $O(CH_2)_2$*CH_2*), 28.5 (2 x OCH_2*CH_2*), 70.3 (2 x OCH_2), 108.0 (C-2, C-5), 136.6 (C-1, C-6), 151.9 (C-3, C-4) ppm; DSC: heating up (10 K/min): Cr 115 °C [-29.5 kJ mol^{-1}] I; cooling down (10 K/min): Cr 113 °C [29.6 kJ mol^{-1}] I; CHN-analysis: calcd. (%) for $C_{16}H_{24}N_2O_6$: C 56.46, H 7.11, N 8.23, found: C 56.63, H 7.15, N 8.19. The spectroscopic data are in accordance to literature.[14]

1,2-Dinitro-4,5-bis(hexyloxy)benzene (U-Nitro(6)):

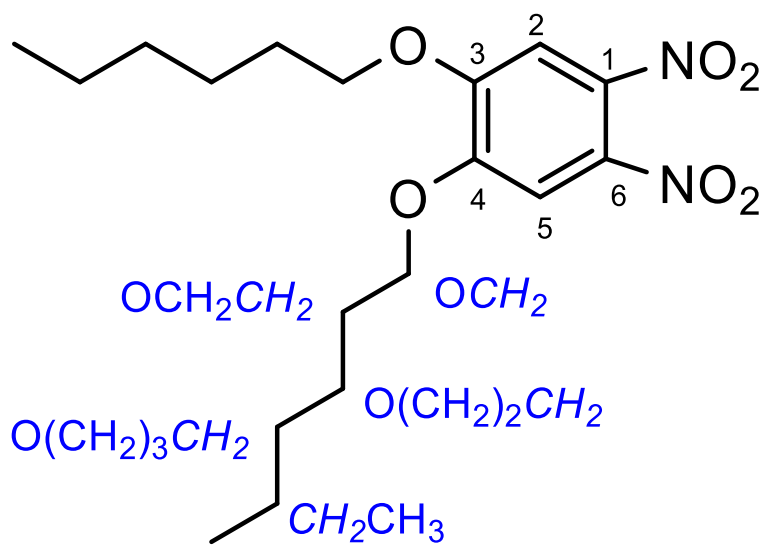

U-Nitro(6)

$C_{18}H_{28}N_2O_6$
(368.19 g mol^{-1})

According to **GP1**, 1,2-bishexyloxybenzene (14.8 g, 53.3 mmol), HNO_3 (100 %, 13.3 mL, 319 mmol), H_2SO_4 (96 %, 11.4 mL, 213 mmol) in 145 mL CH_2Cl_2; **U-Nitro(6)** as yellow crystals: 11.3 g (30.1 mmol, 58 %); ^{1}H NMR (700 MHz, $CDCl_3$): δ = 0.91 (t, *J* = 6.8 Hz, 6H, 2 x CH_3), 1.33-1.38 (m, 8H, 2 x CH_3*CH_2*, 2 x CH_3CH_2*CH_2*), 1.46-1.50 (m, 4H, 2 x $O(CH_2)_2$*CH_2*), 1.85-1.89 (m, 4H, 2 x OCH_2*CH_2*), 4.10 (t, *J* = 6.5 Hz, 4H, 2 x OCH_2), 7.29 (s, 2H, 2-H, 5-H) ppm; ^{13}C NMR (175 MHz, $CDCl_3$): δ = 14.1 (2 x CH_3), 22.7 (2 x CH_3*CH_2*), 25.6 (2 x $O(CH_2)_2$*CH_2*), 28.8 (2 x OCH_2*CH_2*), 31.5 (2

x $CH_3CH_2CH_2$), 70.3 (2 x OCH_2), 108.0 (C-2, C-5), 136.6 (C-1, C-6), 151.9 (C-3, C-4) ppm; DSC: heating up (10 K/min): Cr_1 9 °C [-3.3 kJ mol^{-1}] Cr_2 102 °C [-28.7 kJ mol^{-1}] I; cooling down (10 K/min):] Cr_1 8 °C [1.7 kJ mol^{-1}] Cr_2 100 °C [28.7] kJ mol^{-1} I; CHN-analysis: calcd. (%) for $C_{18}H_{28}N_2O_6$: C 58.68, H 7.66, N 7.60, found: C 58.74, H 7.66, N 7.66. The spectroscopic data are in accordance to literature.[15]

1,2-Dinitro-4,5-bis(octyloxy)benzene (U-Nitro(7)):

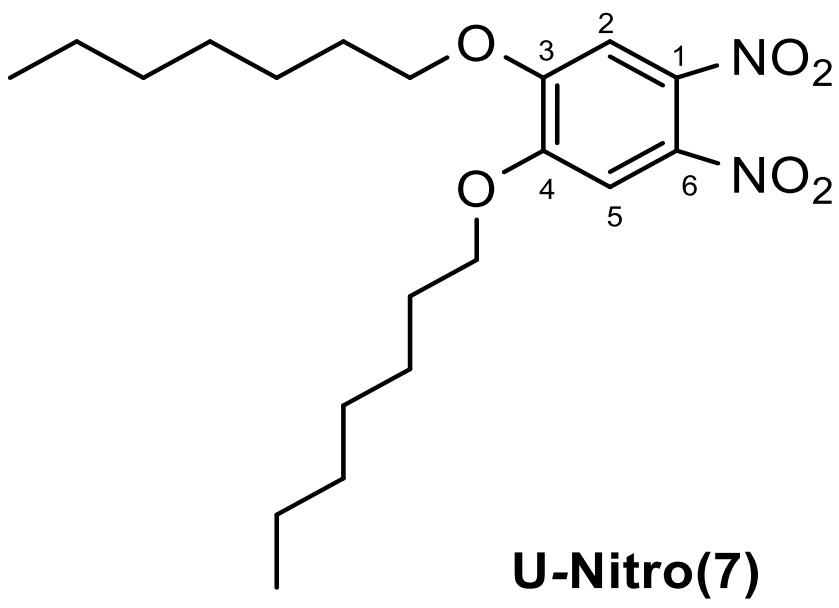

U-Nitro(7)

$C_{20}H_{32}N_2O_6$
(396.48 g mol^{-1})

According to **GP1**, 1,2-bisheptyloxybenzene (15.0 g, 49.0 mmol), HNO_3 (100 %, 12.2 mL, 294 mmol), H_2SO_4 (96 %, 10.5 mL, 196 mmol) in 134 mL CH_2Cl_2; **U-Nitro(7)** as yellow crystals: 12.5 g (31.6 mmol, 65 %); ^{1}H NMR (700 MHz, CDCl3): δ = 0.90 (t, J = 6.8 Hz, 6H, 2 x CH_3), 1.29-1.39 (m, 12H, CH_2), 1.45-1.50 (m, 4H, 2 x $O(CH_2)2CH_2$), 1.85-1.89 (m, 4H, 2 x OCH_2CH_2), 4.10 (t, J = 6.5 Hz, 4H, 2 x OCH_2), 7.29 (s, 2H, 2-H, 5-H) ppm; ^{13}C NMR (175 MHz, $CDCl_3$): δ = 14.2 (2 x CH_3), 22.7 (CH_2), 25.9 (2 x $O(CH_2)_2CH_2$), 28.8 (2 x OCH_2CH_2), 29.0, 31.8 (CH_2), 70.3 (2 x OCH_2), 108.0 (C-2, C-5), 136.6 (C-1, C-6), 151.9 (C-3, C-4) ppm; MS (EI pos.): m/z (%) = 396.2 (41) $[M]^+$, 298.1 (9) $[M-C_7H_{15}+H]^+$, 282.1 (25) $[M-OC_7H_{15}+H]^+$, 200.0 (20) $[M-2 x C_7H_{15}+H]^+$, 184.0 (100) $[M-C_7H_{15}-OC_7H_{15}+2H]^+$; HRMS (EI pos.): calcd. for $C_{20}H_{32}N_2O_6$: 396.2255 found: 396.2252; FT-IR (ATR): $\tilde{\nu}$ /cm^{-1} = 2954 (w), 2929 (w), 2858 (w), 1590 (w), 1533 (s), 1467 (w), 1367 (m), 1335 (m), 1282 (s), 1226 (m), 1040 (m), 906 (vs), 867 (w), 814 (w), 727 (vs), 650 (m) cm^{-1}; DSC: heating up (10 K/min) Cr_1 5 °C [-4.5 kJ mol^{-1}] Cr_2 10 °C [-0.4 kJ mol^{-1}] Cr_3 91 °C [-26.9 kJ mol^{-1}] I; cooling down (10 K/min) Cr_1 5 °C [4.4 kJ mol^{-1}] Cr_2 9 °C [0.5 kJ mol^{-1}] Cr_3 90 °C [27.0 kJ mol^{-1}] I; CHN-analysis: calcd. (%) for $C_{20}H_{32}N_2O_6$: C 60.59, H 8.13, N 7.07, found: C 60.70, H 8.12, N 7.03.

1,2-Dinitro-4,5-bis(octyloxy)benzene (U-Nitro(8)):

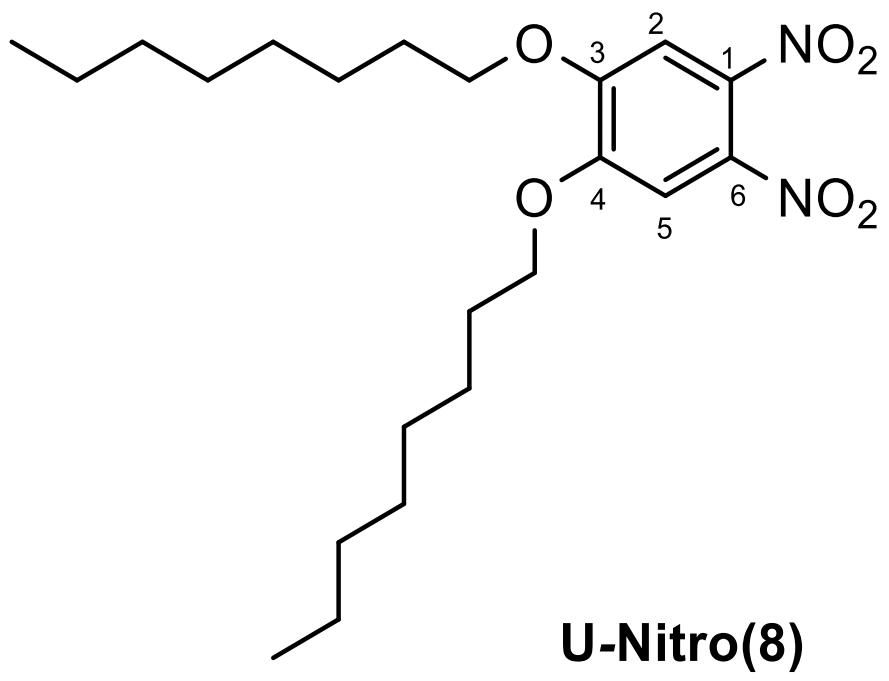

$C_{22}H_{36}N_2O_6$
(424.26 g mol^{-1})

U-Nitro(8)

According to **GP1**, 1,2-bisoctyloxybenzene (1.00 g, 3.00 mmol), HNO_3 (100 %, 0.98 mL, 18 mmol), H_2SO_4 (96 %, 0.64 mL, 12.0 mmol) in 6 mL CH_2Cl_2; **U-Nitro(8)** as yellow crystals: 1.00 g (2.35 mmol, 79 %); ^{1}H NMR (700 MHz, $CDCl_3$): δ = 0.89 (t, *J* = 6.8 Hz, 6H, 2 x CH_3), 1.27-1.38 (m, 16H, CH_2), 1.45-1.50 (m, 4H, 2 x $O(CH_2)_2C\mathit{H_2}$), 1.85-1.89 (m, 4H, 2 x $OCH_2C\mathit{H_2}$), 4.10 (t, *J* = 6.5 Hz, 4H, 2 x OCH_2), 7.29 (s, 2H, 2-H, 5-H) ppm; ^{13}C NMR (175 MHz, $CDCl_3$): δ = 14.2 (2 x CH_3), 22.8 (CH_2), 25.9 (2 x $O(CH_2)_2\mathit{C}H_2$), 28.8 (2 x $OCH_2\mathit{C}H_2$), 29.3, 31.9 (CH_2), 70.3 (2 x OCH_2), 108.0 (C-2, C-5), 136.6 (C-1, C-6), 151.9 (C-3, C-4) ppm; MS (EI pos.): m/z (%) = 424.3 (42) $[M]^+$, 312.1 (9) $[M\text{-}C_8H_{17}\text{+}H]^+$, 296.1 (28) $[M\text{-}OC_8H_{17}\text{+}H]^+$, 200.0 (10) $[M\text{-}2 \times C_8H_{17}\text{+}H]^+$, 184.0 (100) $[M\text{-}C_8H_{17}\text{-}OC_7H_{15}\text{+}2H]^+$; HRMS (EI pos.): calc. for $C_{22}H_{36}N_2O_6$: 424.2568, found: 424.2570; FT-IR (ATR): $\tilde{\nu}$ /cm^{-1} = 2927 (m), 2856 (w), 1590 (w), 1533 (s), 1467 (m), 1335 (m), 1282 (s), 1225 (s), 1041 (m), 906 (vs), 866 (w), 812 (w), 727 (vs), 650 (m) cm^{-1}; DSC: heating up (10 K/min) Cr_1 11 °C [-5.0 kJ mol^{-1}] Cr_2 16 °C [-6.0 kJ mol^{-1}] Cr_3 81 °C [-20.5 kJ mol^{-1}] I; cooling down (10 K/min) Cr_1 16 °C [6.2 kJ mol^{-1}] Cr_2 82 °C [21.2 kJ mol^{-1}] I; CHN-analysis: calcd. (%) for $C_{22}H_{36}N_2O_6$: C 62.24, H 8.55, N 6.60, calcd. (%) for $C_{22}H_{36}N_2O_6$ + 0.06 eq. H_2O: C 62.08, H 8.55, N 6.58, found: C 61.91, H 8.42, N 6.70. The spectroscopic data are in accordance to literature.[15]

1,2-Dinitro-4,5-bis(nonyloxy)benzene (U-Nitro(9)):

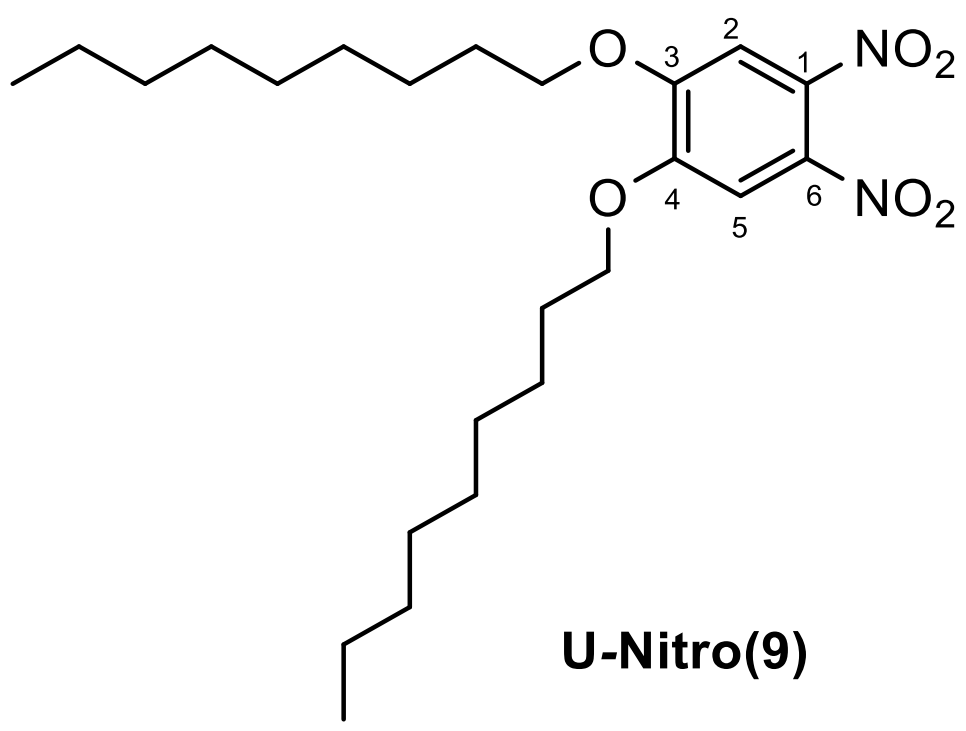

$C_{24}H_{40}N_2O_6$
(452.29 g mol^{-1})

U-Nitro(9)

According to **GP1**, 1,2-bisnonylloxybenzene (16.1 g, 44.0 mmol), HNO_3 (100 %, 9.60 mL, 229 mmol), H_2SO_4 (96 %, 8.20 mL, 153 mmol) in 100 mL CH_2Cl_2; **U-Nitro(9)** as yellow crystals: 18.9 g (41.9 mmol, 95 %); ^{1}H NMR (700 MHz, $CDCl_3$): δ = 0.88 (t, *J* = 7.0 Hz, 6H, 2 x CH_3), 1.26-1.39 (m,

20H, CH_2), 1.45-1.49 (m, 4H, 2 x $O(CH_2)_2CH_2$), 1.84-1.89 (m, 4H, 2 x OCH_2CH_2), 4.10 (t, *J* = 6.5 Hz, 4H, 2 x OCH_2), 7.29 (s, 2H, 2-H, 5-H) ppm; ^{13}C NMR (175 MHz, $CDCl_3$): δ = 14.2 (2 x CH_3), 22.8 (CH_2), 25.9 (2 x $O(CH_2)_2CH_2$), 28.8 (2 x OCH_2CH_2), 29.36, 29.37 (CH_2), 29.6, 32.0 (CH_2), 70.3 (2 x OCH_2), 108.0 (C-2, C-5), 136.6 (C-1, C-6), 151.9 (C-3, C-4) ppm; MS (EI pos.): m/z (%) = 452.3 (42) $[M]^+$, 259.2(13), 241.2 (11), 184.0 (9) $[M-C_9H_{19}-OC_9H_{19}+2H]^+$, 147.1 (34), 129.1 (100); HRMS (EI pos.): calc. for $C_{24}H_{40}N_2O_6$: 452.2881, found: 452.2878; FT-IR (ATR): $\tilde{\nu}$ /cm^{-1} = 2956 (m), 2919 (s), 2850 (m), 1587 (w), 1528 (vs), 1465 (m), 1398 (w), 1370 (s), 1352 (s), 1334 (s), 1285 (vs), 1225 (vs), 1066 (m), 1055 (m), 1037 (m), 1005 (w), 963 (w), 907 (s), 871 (m), 814 (m) cm^{-1}; DSC: heating up (10 K/min) Cr_1 32 °C [-14.5 kJ mol^{-1}] Cr_2 40 °C [-0.9 kJ mol^{-1}] Cr_3 84 °C [-26.8 kJ mol^{-1}] I; cooling down (10 K/min) Cr_1 28 °C [12.2 kJ mol^{-1}] Cr_2 31 °C [3.2 kJ mol^{-1}] Cr_3 81 °C [27.3 kJ mol^{-1}] I; CHN-analysis: calcd. (%) for $C_{24}H_{40}N_2O_6$: C 63.69, H 8.91, N 6.19, found: C 63.60, H 8.93, N 6.15. The spectroscopic data are in accordance to literature.[14]

1,2-Dinitro-4,5-bis(decyloxy)benzene (U-Nitro(10)):

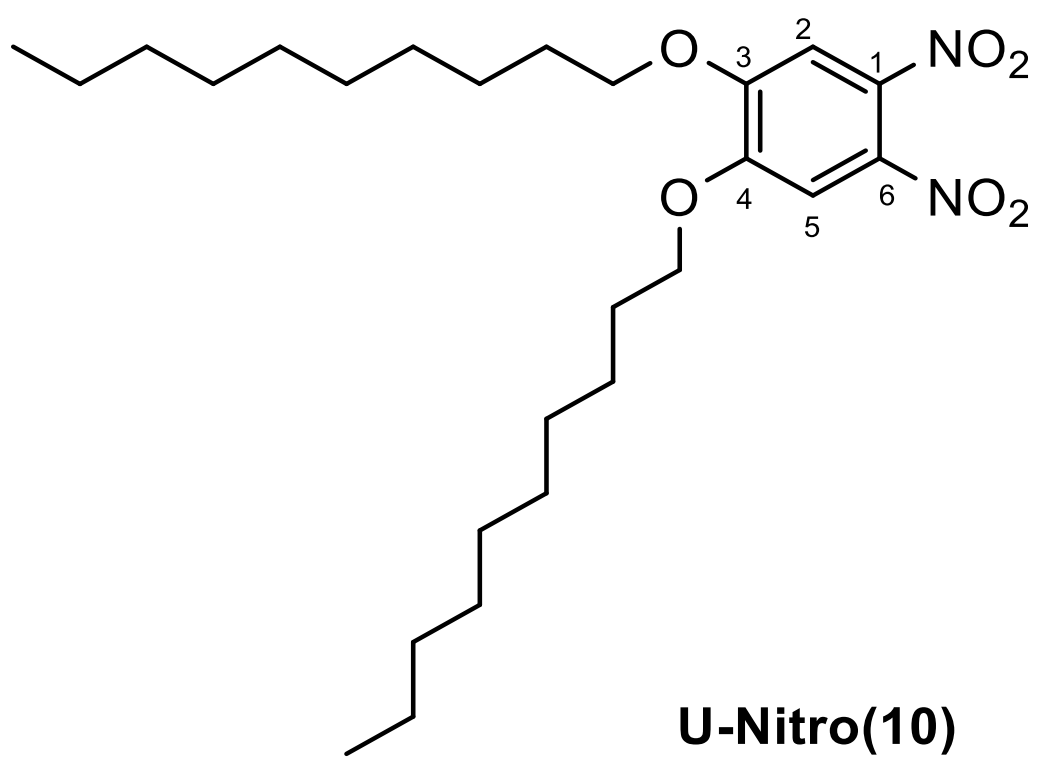

$C_{26}H_{44}N_2O_6$
(480.32 g mol^{-1})

U-Nitro(10)

According to **GP1**, 1,2-bisdecyloxybenzene (1.17 g, 3.00 mmol), HNO_3 (100 %, 0.98 mL, 18.0 mmol), H_2SO_4 (96 %, 0.64 mL, 12.0 mmol) in 6 mL CH_2Cl_2; **U-Nitro(10)** as yellow crystals: 1.30 g (2.70 mmol, 90 %); ^1H NMR (700 MHz, $CDCl_3$): δ = 0.88 (t, *J* = 7.0 Hz, 6H, 2 x CH_3), 1.27-1.38 (m, 24H, CH_2), 1.45-1.49 (m, 4H, 2 x $O(CH_2)_2CH_2$), 1.85-1.89 (m, 4H, 2 x OCH_2CH_2), 4.10 (t, *J* = 6.5 Hz, 4H, 2 x OCH_2), 7.29 (s, 2H, 2-H, 5-H) ppm; ^{13}C NMR (175 MHz, $CDCl_3$): δ = 14.3 (2 x CH_3), 22.8 (CH_2), 25.9 (2 x $O(CH_2)_2CH_2$), 28.8 (2 x OCH_2CH_2), 29.4, 29.5, 29.66, 29.68, 32.0 (CH_2), 70.3 (2 x OCH_2), 108.0 (C-2, C-5), 136.6 (C-1, C-6), 151.9 (C-3, C-4) ppm; DSC: heating up (10 K/min) Cr_1 53 °C [-28.0 kJ mol^{-1}] Cr_2 81 °C [-24.3 kJ mol^{-1}] I; cooling down (10 K/min) Cr_1 34 °C [20.1 kJ mol^{-1}] Cr_2 80 °C [26.7 kJ mol^{-1}] I; CHN-analysis: calcd. (%) for $C_{26}H_{44}N_2O_6$: C 64.97, H 9.23, N 5.83, found: C 65.06, H 9.22, N 5.82. The spectroscopic data are in accordance to literature.[15]

1,2-Dinitro-4,5-bis(dodecyloxy)benzene (U-Nitro(12)):

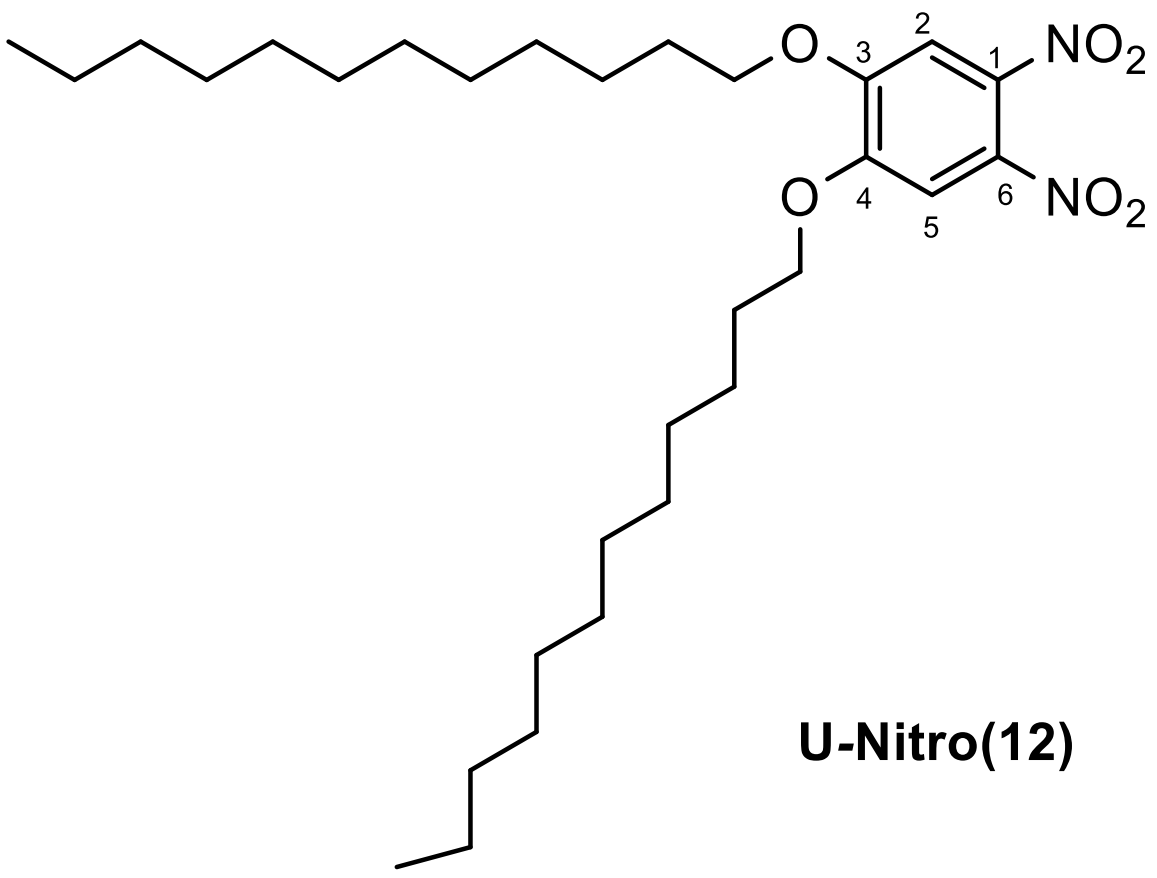

$C_{30}H_{52}N_2O_6$
(536.38 g mol^{-1})

U-Nitro(12)

According to **GP1**, 1,2-bisdodecyloxybenzene (14.5 g, 32.5 mmol), HNO_3 (100 %, 7.1 mL, 170 mmol), H_2SO_4 (96 %, 6.00 mL, 112 mmol) in 80 mL CH_2Cl_2; **U-Nitro(12)** as yellow crystals: 10.0 g (18.6 mmol, 57 %); ^{1}H NMR (700 MHz, $CDCl_3$): δ = 0.88 (t, *J* = 7.1 Hz, 6H, 2 x CH_3), 1.26-1.38 (m, 32H, CH_2), 1.45-1.49 (m, 4H, 2 x $O(CH_2)_2CH_2$), 1.85-1.89 (m, 4H, 2 x OCH_2CH_2), 4.10 (t, *J* = 6.5 Hz, 4H, 2 x OCH_2), 7.29 (s, 2H, 2-H, 5-H) ppm; ^{13}C NMR (175 MHz, $CDCl_3$): δ = 14.3 (2 x CH_3), 22.8 (2 x CH_3CH_2), 26.0 (2 x $O(CH_2)_2CH_2$), 28.8 (2 x OCH_2CH_2), 29.4, 29.5, 29.69, 29.72, 29.79, 29.82 32.1 (CH_2), 70.3 (2 x OCH_2), 108.0 (C-2, C-5), 136.6 (C-1, C-6), 151.9 (C-3, C-4) ppm; DSC: heating up (10 K/min) Cr_1 34 °C [-34.4 kJ mol^{-1}] Cr_2 80 °C [-28.5 kJ mol^{-1}] I; cooling down (10 K/min) Cr_1 58 °C [31.8 kJ mol^{-1}] Cr_2 78 °C [30.5 kJ mol^{-1}] I; CHN-analysis: calcd. (%) for $C_{30}H_{52}N_2O_6$: C 67.13, H 9.76, N 5.22, found: C 67.31, H 9.78, N 5.20. The spectroscopic data are in accordance to literature.[10]

1,2-Dinitro-4,5-bis(tetradecyloxy)benzene (U-Nitro(14)):

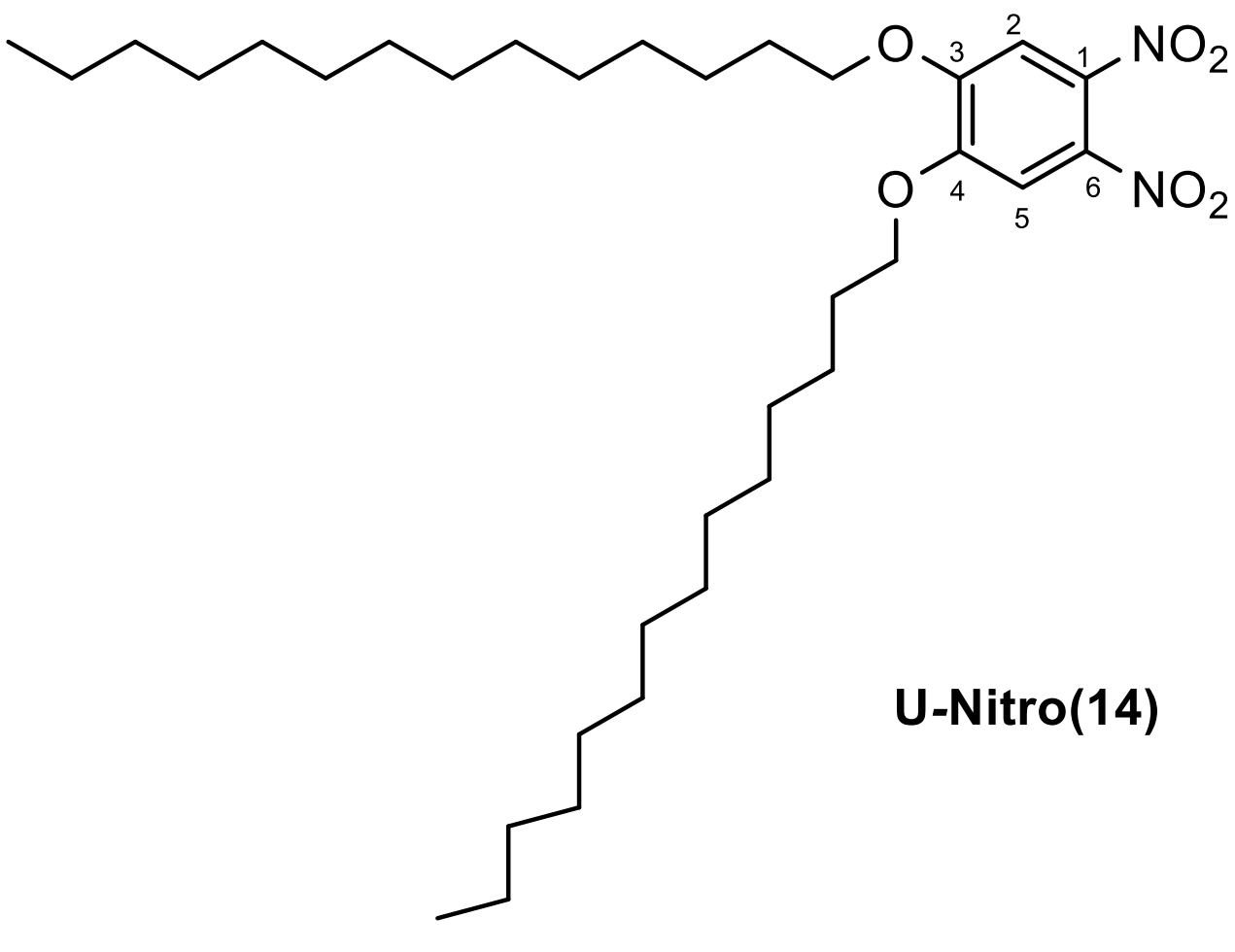

$C_{34}H_{60}N_2O_6$
(592.45 g mol^{-1})

U-Nitro(14)

According to **GP1**, 1,2-bistetradecyloxybenzene (9.5 g, 18.9 mmol), HNO_3 (100%, 4.00 mL, 96 mmol), 100 mL CH_2Cl_2; **U-Nitro(14)** as yellow crystals: 9.20 g (15.5 mmol, 82 %); ^{1}H NMR (700 MHz, $CDCl_3$): δ = 0.87 (t, *J* = 7.1 Hz, 6H, 2 x CH_3), 1.22-1.37 (m, 40H, CH_2), 1.44-1.48 (m, 4H, 2 x $O(CH_2)_2CH_2$), 1.85-1.89 (m, 4H, 2 x OCH_2CH_2), 4.10 (t, *J* = 6.5 Hz, 4H, 2 x OCH_2), 7.29 (s, 2H, 2-H,

5-H) ppm; ^{13}C NMR (175 MHz, $CDCl_3$): δ = 14.3 (2 x CH_3), 22.9 (2 x CH_3*CH_2*), 25.9 (2 x $O(CH_2)_2$*CH_2*), 28.7 (2 x OCH_2*CH_2*), 29.4, 29.5, 29.70, 29.73, 29.82, 29.83, 29.85, 29.86, 32.1 (CH_2), 70.2 (2 x OCH_2), 107.7 (C-2, C-5), 136.5 (C-1, C-6), 151.7 (C-3, C-4) ppm. 29.72, 29.79, 29.82 32.1 (CH_2), 70.3 (2 x OCH_2), 108.0 (C-2, C-5), 136.6 (C-1, C-6), 151.9 (C-3, C-4) ppm; DSC: heating up (10 K/min) Cr 79 °C [-75.4 kJ mol^{-1}] I; cooling down (10 K/min) Cr_1 72 °C [43.9 kJ mol^{-1}] Cr_2 78 °C [31.9 kJ mol^{-1}] I; CHN-analysis: calcd. (%) for $C_{34}H_{60}N_2O_6$: C 68.88, H 10.20, N 4.73, found: C 68.96, H 10.25, N 4.69. The spectroscopic data are in accordance to literature.[16]

2,3-Dinitro-1,4-bis(pentyloxy)benzene (L-Nitro(5)):

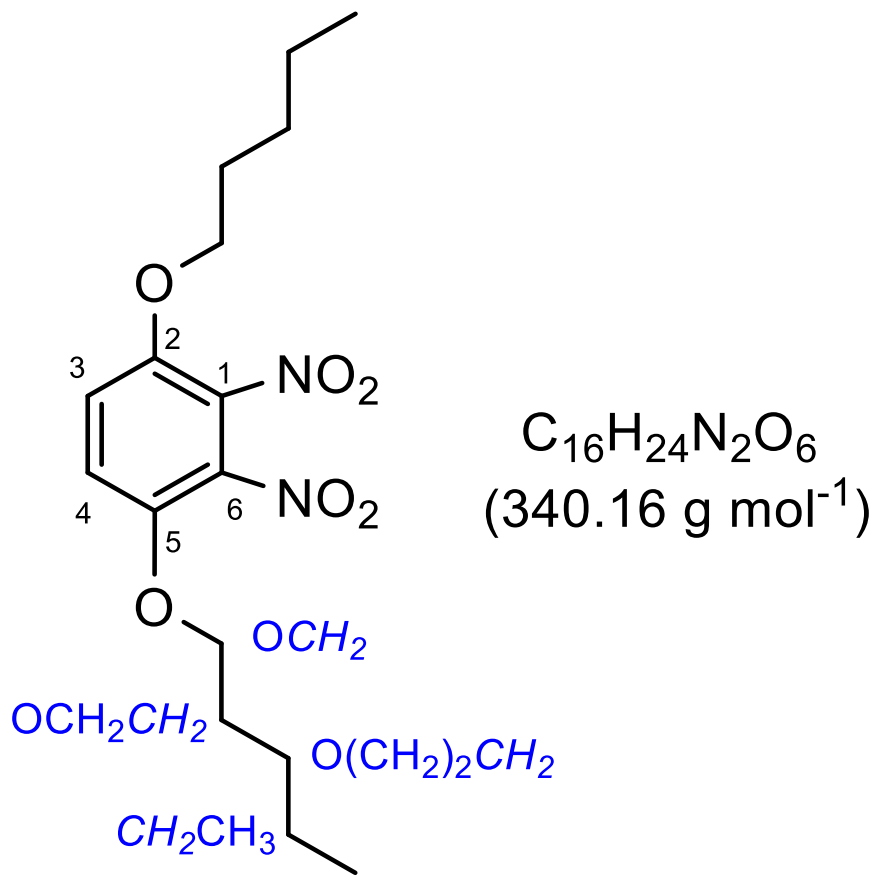

L-Nitro(5)

According to **GP2**, 1,4-bispentyloxybenzene (10.0 g, 40.0 mmol), HNO_3 (65 %, 40 mL), HNO_3 (100 %, 15 mL) in 50 mL CH_2Cl_2 and 50 mL conc. AcOH; **L-Nitro(5)** as yellow crystals: 5.50 g (16.1 mmol, 40 %) without further purification; ^{1}H NMR (500 MHz, $CDCl_3$): δ = 0.91 (t, *J* = 7.1 Hz, 6H, 2 x CH_3), 1.31-1.43 (m, 8H, 2 x C*H_2*CH_3, 2 x $O(CH_2)_2$C*H_2*)), 1.74-1.80 (m, 4H, 2 x OCH_2C*H_2*), 4.05 (t, *J* = 6.4 Hz, 4H, 2 x OCH_2), 7.15 (s, 2H, 3-H, 4-H) ppm; ^{13}C NMR (125 MHz, $CDCl_3$): δ = 14.0 (2 x CH_3), 22.4 (2 x *CH_2*CH_3), 27.9 (2 x $O(CH_2)_2$*CH_2*), 28.7 (2 x OCH_2*CH_2*), 71.2 (2 x OCH_2), 117.9 (C-3, C-4), 134.8 (C-1, C-6), 145.0 (C-2, C-5) ppm; MS (ESI): m/z (%) = 363.15 [M+Na$^+$]; HRMS (ESI): calcd. for $C_{16}H_{24}N_2O_6$+Na$^+$: 363.1527, found: 363.1520; FT-IR (ATR): $\tilde{\nu}$ /cm^{-1} = 2956 (m), 2872 (w), 1547 (vs), 1494 (s), 1467 (s), 1434 (m), 1358 (s), 1271 (vs), 1155 (m), 1038 (s), 948 (m), 907 (s), 886 (m), 811 (s), 801 (s), 729 (vs), 650 (m) cm^{-1}; DSC: heating up (10 K/min) Cr 55 °C [-28.5 kJ mol^{-1}] I; cooling down (10 K/min) Cr_1 55 °C [6.7 kJ mol^{-1}] Cr_2 38 °C [20.9 kJ mol^{-1}] I; CHN-analysis: calcd. (%) for $C_{16}H_{24}N_2O_6$: C 56.46, H 7.11, N 8.23, found: C. 56.51, H 7.14, N 8.11.

2,3-Dinitro-1,4-bis(hexyloxy)benzene (L-Nitro(6)):

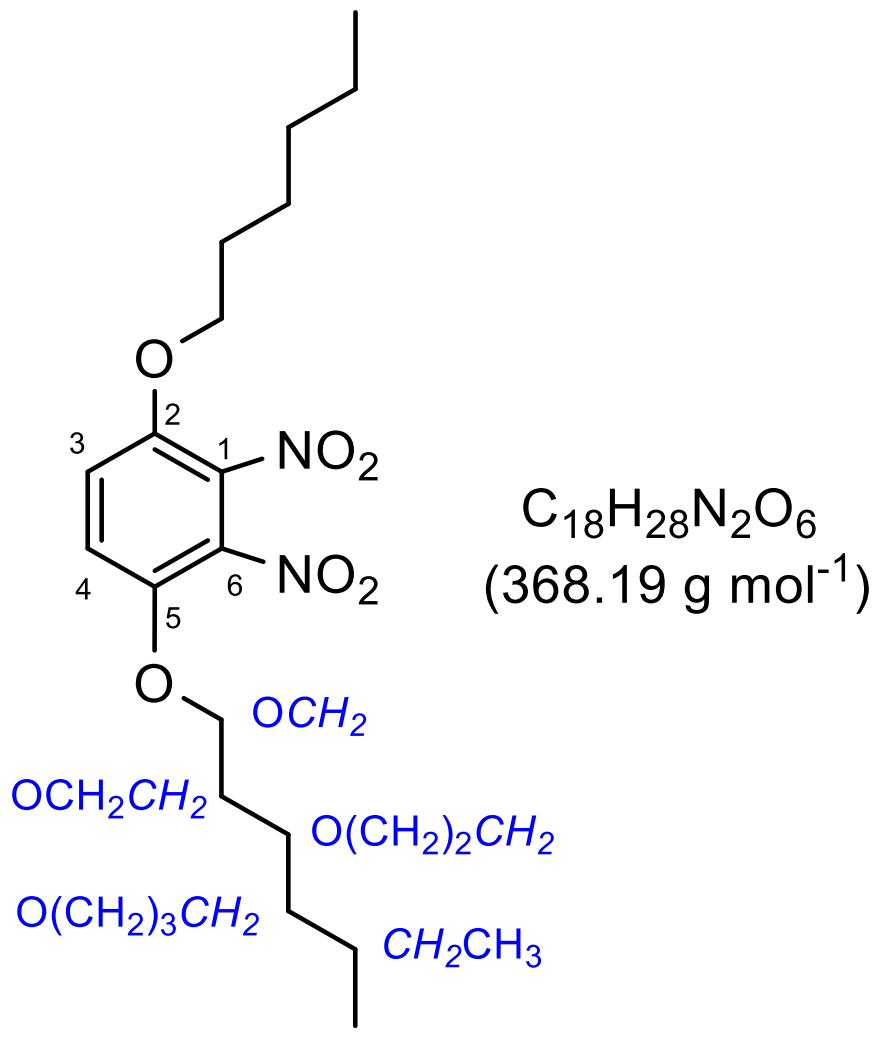

L-Nitro(6)

According to **GP2**, 1,4-bishexyloxybenzene (11.1 g, 40.0 mmol), HNO_3 (65 %, 40 mL), HNO_3 (100 %, 15 mL) in 60 mL CH_2Cl_2 and 50 mL conc. AcOH; **L-Nitro(6)** as yellow crystals: 7.20 g (19.7 mmol, 49 %) without further purification; ^{1}H NMR (700 MHz, $CDCl_3$): δ = 0.89 (t, *J* = 6.9 Hz, 6H, 2 x CH_3), 1.29-1.34 (m, 8H, CH_2), 1.39-1.44 (m, 4H, 2 x $O(CH_2)_2CH_2$), 1.74-1.78 (m, 4H, 2 x OCH_2CH_2), 4.05 (t, *J* = 6.5 Hz, 4H, 2 x OCH_2), 7.15 (s, 2H, 3-H, 4-H) ppm; ^{13}C NMR (175 MHz, $CDCl_3$): δ = 14.1 (2 x CH_3), 22.6 (CH_2), 25.5 (2 x $O(CH_2)_2CH_2$), 29.0 (2 x OCH_2CH_2), 31.5 (CH_2), 71.1 (2 x OCH_2), 117.9 (C-3, C-4), 134.8 (C-1, C-6), 145.0 (C-2, C-5) ppm; MS (ESI): m/z (%) = 391.18 [$M+Na^+$]; HRMS (ESI): calcd. for $C_{18}H_{28}N_2O_6+Na^+$: 391.1840, found: 391.1835; FT-IR (ATR): $\tilde{\nu}$ /cm^{-1} = 2956 (m), 2926 (m), 2857 (m), 1542 (vs), 1495 (m), 1468 (m), 1433 (w), 1359 (s), 1277 (s), 1155 (w), 1046 (m), 812 (m) cm^{-1}; DSC: heating up (10 K/min) Cr 53 °C [-26.6 kJ mol^{-1}] I; cooling down (10 K/min) Cr 47 °C [25.3 kJ mol^{-1}] I; CHN-analysis: calcd. (%) for $C_{18}H_{28}N_2O_6$: C 58.68, H 7.66, N 7.60, found: C 58.71, H 7.69, N 7.55.

2,3-Dinitro-1,4-bis(heptyloxy)benzene (L-Nitro(7)):

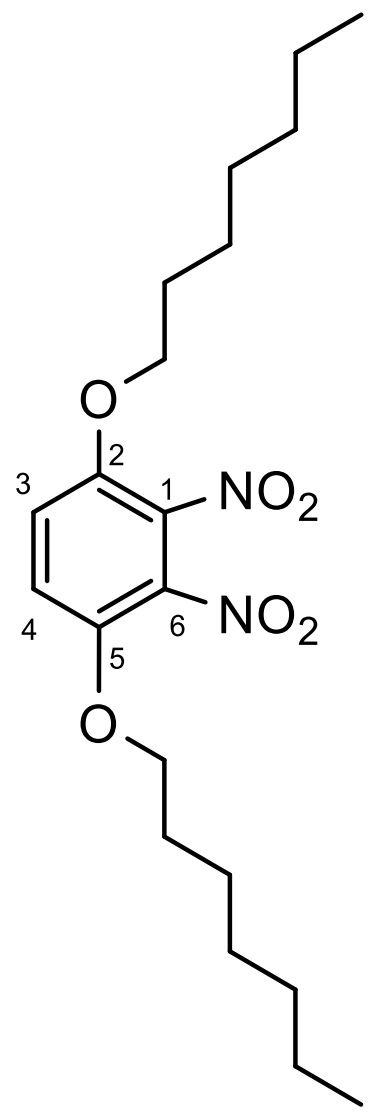

$C_{20}H_{32}N_2O_6$
(396.23 g mol^{-1})

L-Nitro(7)

According to **GP2**, 1,4-bisheptyloxybenzene (12.3 g, 40.0 mmol), HNO_3 (65 %, 40 mL), HNO_3 (100 %, 10 mL) in 100 mL CH_2Cl_2 and 50 mL conc. AcOH; **L-Nitro(7)** as yellow crystals: 10.3 g (25.9 mmol, 65 %) without further purification; ^{1}H NMR (700 MHz, $CDCl_3$): δ = 0.89 (t, *J* = 7.0 Hz, 6H, 2 x CH_3), 1.26-1.34 (m, 12H, CH_2), 1.39-1.43 (m, 4H, 2 x $O(CH_2)_2C\mathit{H}_2$), 1.74-1.78 (m, 4H, 2 x $OCH_2C\mathit{H}_2$), 4.05 (t, *J* = 6.5 Hz, 4H, 2 x OCH_2), 7.15 (s, 2H, 3-H, 4-H) ppm; ^{13}C NMR (175 MHz, $CDCl_3$): δ = 14.2 (2 x CH_3), 22.7 (CH_2), 25.8 (2 x $O(CH_2)_2\mathit{C}H_2$), 29.00, 29.01 (2 x $OCH_2\mathit{C}H_2$, CH_2), 31.8 (CH_2), 71.2 (2 x OCH_2), 117.9 (C-3, C-4), 134.8 (C-1, C-6), 145.0 (C-2, C-5) ppm; MS (ESI): m/z (%) = 419.21 [M+Na$^+$]; HRMS (ESI): calcd. for $C_{20}H_{32}N_2O_6$+Na$^+$: 419.2153, found: 419.2146; FT-IR (ATR): $\tilde{\nu}$ /cm^{-1} = 2953 (m), 2930 (m), 2870 (w), 1546 (vs), 1494 (s), 1465 (s), 1434 (m), 1392 (m), 1358 (s), 1271 (vs), 1231 (w), 1155 (m), 1042 (s), 1021 (m), 927 (m), 908 (s), 833 (w), 812 (s), 732 (vs), 650 (w) cm^{-1}; DSC: heating up (10 K/min) Cr_1 35 °C [-19.6 kJ mol^{-1}] Cr_2 39 °C [-13.3 kJ mol^{-1}] I; cooling down (10 K/min) Cr_1 -2 °C [21.4 kJ mol^{-1}] Cr_2 37 °C [7.7 kJ mol^{-1}] I; CHN-analysis: calcd. (%) for $C_{20}H_{32}N_2O_6$: C 60.59, H 8.14, N 7.07, found: C. 60.88, H 8.18, N 7.03.

2,3-Dinitro-1,4-bis(octyloxy)benzene (L-Nitro(8)):

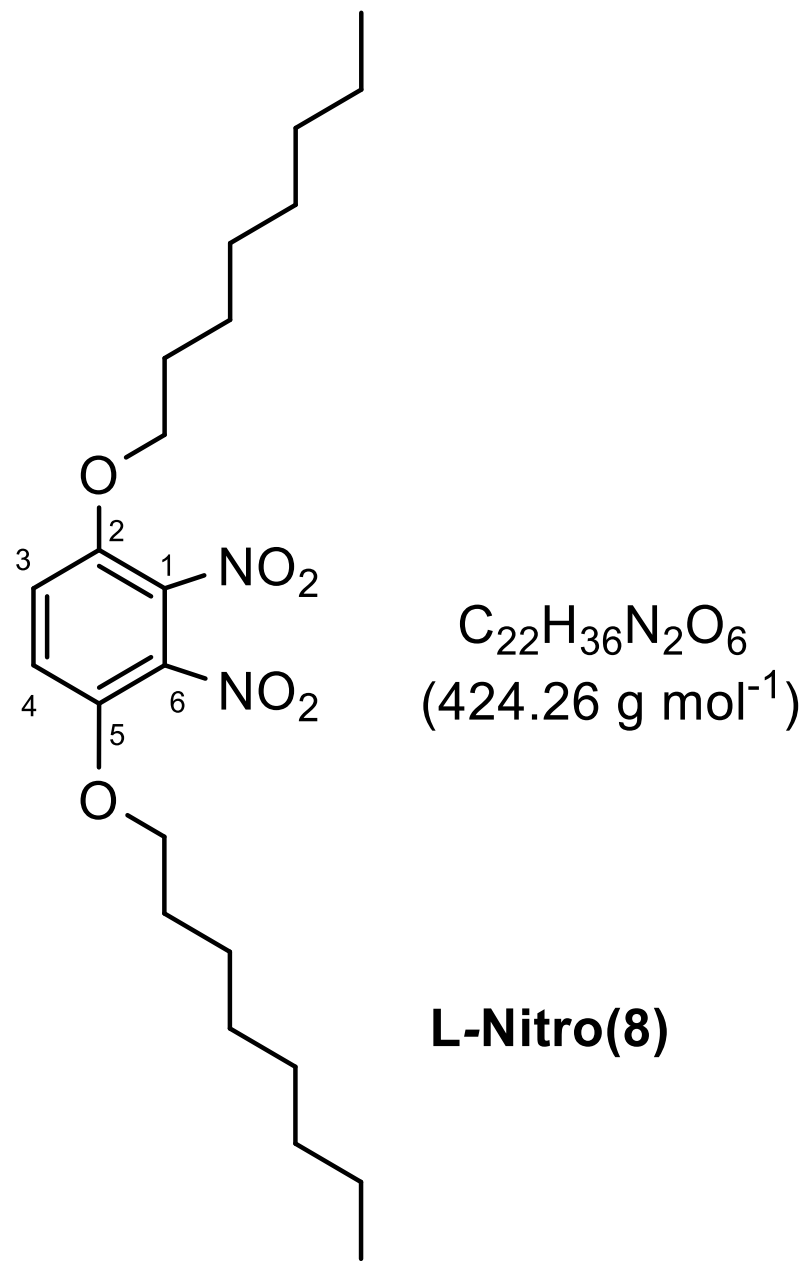

L-Nitro(8)

According to **GP2**, 1,4-bisoctyloxybenzene (10.1 g, 30.2 mmol), HNO_3 (65 %, 30 mL), HNO_3 (100 %, 13 mL) in 110 mL CH_2Cl_2 and 50 mL conc. AcOH; **L-Nitro(8)** as yellow crystals: 10.3 g (25.9 mmol, 65 %) without further purification; ^{1}H NMR (500 MHz, $CDCl_3$): δ = 0.88 (t, *J* = 7.0 Hz, 6H, 2 x CH_3), 1.27-1.30 (m, 16H, CH_2), 1.38-1.44 (m, 4H, 2 x $O(CH_2)_2CH_2$), 1.73-1.78 (m, 4H, 2 x OCH_2CH_2), 4.05 (t, *J* = 6.5 Hz, 4H, 2 x OCH_2), 7.15 (s, 2H, 3-H, 4-H) ppm; ^{13}C NMR (125 MHz, $CDCl_3$): δ = 14.2 (2 x CH_3), 22.8 (CH_2), 25.8 (2 x $O(CH_2)_2CH_2$), 29.0, 29.26, 29.28 (2 x OCH_2CH_2, CH_2), 31.9 (CH_2), 71.2 (2 x OCH_2), 117.9 (C-3, C-4), 134.7 (C-1, C-6), 144.9 (C-2, C-5) ppm; MS (ESI): m/z (%) = 447.25 [M+Na$^+$]; HRMS (ESI): calcd. for $C_{22}H_{36}N_2O_6$+Na$^+$: 447.2466, found: 447.2455; FT-IR (ATR): $\tilde{\nu}$ /cm^{-1} = 2924 (s), 2855 (m), 1543 (vs), 1494 (s), 1467 (s), 1433 (m), 1357 (s), 1274 (vs), 1184 (w), 1154 (m), 1045 (s), 943 (w), 910 (w), 885 (w), 811 (s), 735 (m) cm^{-1}; DSC: heating up (10 K/min) Cr 41 °C [-30.7 kJ mol^{-1}] I; cooling down (10 K/min) Cr_1 11 °C [20.4 kJ mol^{-1}] Cr_2 33 °C [6.4 kJ mol^{-1}] I; CHN-analysis: calcd. (%) for $C_{22}H_{36}N_2O_6$: C 62.24, H 8.55, N 6.60, found: C. 62.54, H 8.62, N 6.53. The spectroscopic data are in accordance to literature.[17]

2,3-Dinitro-1,4-bis(nonyloxy)benzene (L-Nitro(9)):

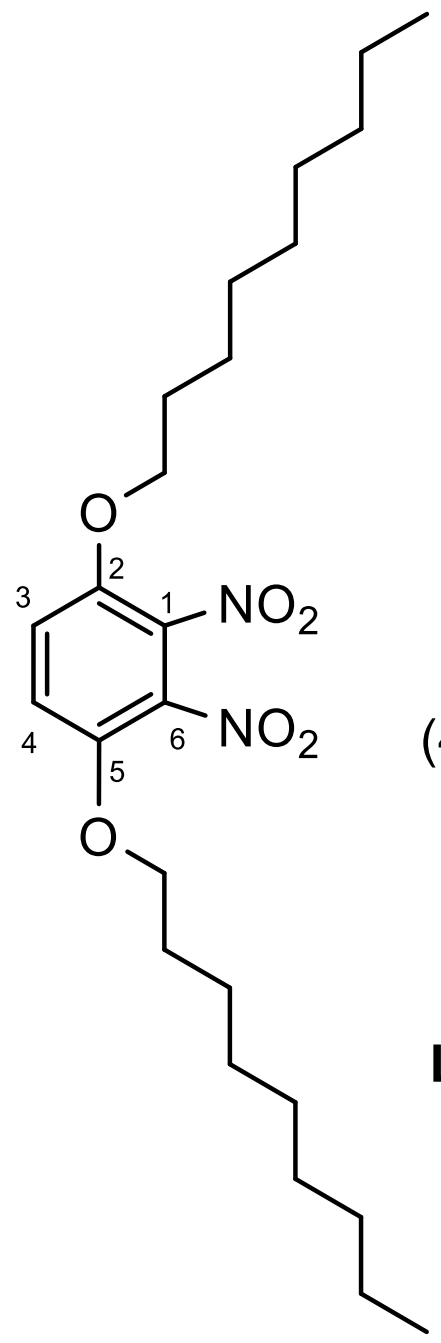

$C_{24}H_{40}N_2O_6$
(452.29 g mol^{-1})

L-Nitro(9)

According to **GP2**, 1,4-bisnonyloxybenzene (10.9 g, 30.0 mmol), HNO_3 (65 %, 30 mL), HNO_3 (100 %, 15 mL) in 100 mL CH_2Cl_2 and 50 mL conc. AcOH, recrystallization from EtOH; **L-Nitro(9)** as yellow crystals: 6.10 g (13.6 mmol, 45 %); ^{1}H NMR (700 MHz, $CDCl_3$): δ = 0.88 (t, *J* = 7.1 Hz, 6H, 2 x CH_3), 1.26-1.30 (m, 20H, CH_2), 1.38-1.43 (m, 4H, 2 x $O(CH_2)_2C\mathit{H_2}$), 1.74-1.78 (m, 4H, 2 x $OCH_2C\mathit{H_2}$), 4.05 (t, *J* = 6.4 Hz, 4H, 2 x OCH_2), 7.15 (s, 2H, 3-H, 4-H) ppm; ^{13}C NMR (175 MHz, $CDCl_3$): δ = 14.2 (2 x CH_3), 22.8 (CH_2), 25.8 (2 x $O(CH_2)_2\mathit{C}H_2$), 29.0, 29.34, 29.34, 29.56 (2 x $OCH_2\mathit{C}H_2$, CH_2), 32.0 (CH_2), 71.2 (2 x OCH_2), 117.9 (C-3, C-4), 134.8 (C-1, C-6), 144.9 (C-2, C-5) ppm; MS (EI pos.): m/z (%) = 452.3 (21) $[M]^+$, 326.1(13), 200.0 (29), 184.0 (12) $[M\text{-}C_9H_{19}\text{-}OC_9H_{19}\text{+}2H]^+$, 126.1 (48), 97.1 (100); HRMS (EI pos.): calcd. for $C_{24}H_{40}N_2O_6$: 452.2881 found: 452.2878; FT-IR (ATR): $\tilde{\nu}$ /cm^{-1} = 2955 (m), 2916 (s), 2850 (s), 1535 (vs), 1494 (s), 1467 (s), 1431 (m), 1395 (m), 1357 (s), 1280 (vs), 1185 (m), 1155 (m), 1071 (s), 1037 (m), 1025 (m), 955 (w), 897 (m), 847 (w), 814 (s), 772 (w), 757 (w), 738 (w), 721 (m) cm^{-1}; DSC: heating up (10 K/min) Cr 49 °C [-33.7 kJ mol^{-1}] I; cooling down (10 K/min) Cr_1 23 °C [19.9 kJ mol^{-1}] Cr_2 35 °C [6.5 kJ mol^{-1}] I; CHN-analysis: calcd. (%) for $C_{24}H_{40}N_2O_6$: C 63.69, H 8.91, N 6.19, found: C. 63.95, H 8.98, N 6.12.

2,3-Dinitro-1,4-bis(decyloxy)benzene (L-Nitro(10)):

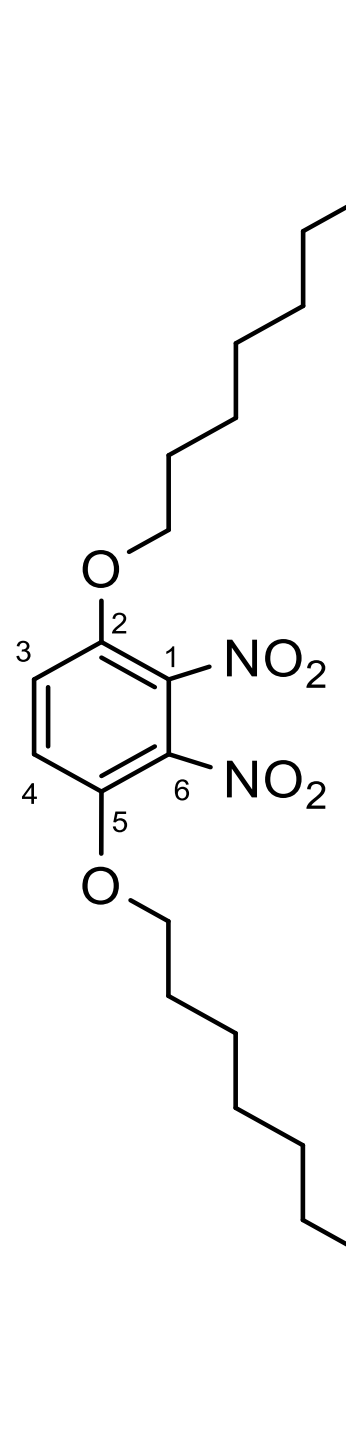

$C_{26}H_{44}N_2O_6$
(480.329 g mol^{-1})

L-Nitro(10)

According to **GP3**, 1,4-bisdecyloxybenzene (8.4 g, 22.0 mmol), HNO_3 (100 %, 5.0 mL, 120 mmol) in 90 mL CH_2Cl_2 and 6 mL conc. AcOH; stirred for 1 h at 0 °C, followed by 1 h at room temperature (not heated up to 70°C); recrystallization from MeOH; **L-Nitro(10)** as yellow crystals: 6.67 g (9.70 mmol, 45 %); ^{1}H NMR (700 MHz, $CDCl_3$): δ = 0.88 (t, J = 7.1 Hz, 6H, 2 x CH_3), 1.26-1.30 (m, 24H, CH_2), 1.38-1.43 (m, 4H, 2 x $O(CH_2)_2CH_2$), 1.74-1.78 (m, 4H, 2 x OCH_2CH_2), 4.05 (t, J = 6.4 Hz, 4H, 2 x OCH_2), 7.15 (s, 2H, 3-H, 4-H) ppm; ^{13}C NMR (175 MHz, $CDCl_3$): δ = 14.3 (CH_3), 22.8 (2 x CH_3CH_2), 25.8 ($O(CH_2)_2CH_2$), 29.0, 29.3, 29.41, 29.43, 29.61, 29.65 (OCH_2CH_2, CH_2), 32.0 (CH_2), 71.2 (OCH_2), 117.9 (C-3, C-4), 134.8 (C-1, C-6), 145.0 (C-2, C-5) ppm; MS (EI pos.): m/z (%) = 480.3 (6) $[M]^+$, 200.0 (13), 149.0 (10), 111.1 (87), 97.1 (100); HRMS (DEP, EI pos.): calcd. for $C_{26}H_{44}N_2O_6$: 480.3194, found: 480.3189.; FT-IR (ATR): $\tilde{\nu}$ /cm^{-1} = 2955 (m), 2916 (s), 2873 (m), 2850 (s), 1536 (vs), 1495 (m), 1468 (s), 1432 (m), 1395 (w), 1358 (m), 1282 (vs), 1186 (w), 1156 (w), 1072 (m), 1050 (w), 1028 (w), 816 (m), 721 (w) cm^{-1}; DSC: heating up (10 K/min) Cr 53 °C [-46.2 kJ mol^{-1}] I; cooling down (10 K/min) Cr_1 36 °C [27.2 kJ mol^{-1}] Cr_2 39 °C [8.8 kJ mol^{-1}] I; CHN-analysis: calcd. (%) for $C_{26}H_{44}N_2O_6$: C 64.97, H 9.23, N 5.83, found: C 65.24, H 9.26, N 5.77.

2,3-Dinitro-1,4-bis(dodecyloxy)benzene (L-Nitro(12)):

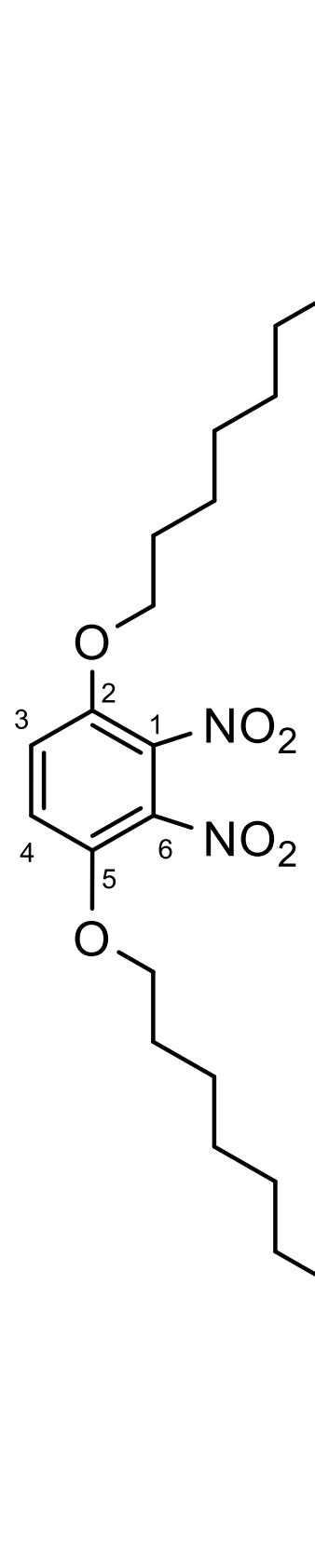

$C_{30}H_{52}N_2O_6$
(536.38 g mol^{-1})

L-Nitro(12)

According to **GP3**, 1,4-bisdodecyloxybenzene (11.3 g, 24.6 mmol), HNO_3 (100 %, 5.2 ml, 125 mmol) in 80 mL $CHCl_3$ and 5 mL conc. AcOH; stirred for 1 h at 0 °C, followed by 1 h at room temperature (not heated up to 70°C); recrystallization from MeOH; **L-Nitro(12)** as yellow crystals: 7.18 g (13.4 mmol, 54 %); ^{1}H NMR (400 MHz, $(CD_3)_2CO$): δ = 0.87 (t, *J* = 6.8 Hz, 6H, 2 x CH_3), 1.28-1.47 (m, 36H, CH_2), 1.73-1.80 (m, 4H, 2 x $OCH_2C\mathit{H_2}$), 4.22 (t, *J* = 6.4 Hz, 4H, 2 x OCH_2), 7.59 (s, 2H, 3-H, 4-H) ppm; ^{13}C NMR (175 MHz, $CDCl_3$): δ = 14.3 (CH_3), 23.3 (2 x $CH_3\mathit{C}H_2$), 26.4 ($O(CH_2)_2\mathit{C}H_2$), 29.3, 29.6, 30.3, 30.4 ($OCH_2\mathit{C}H_2$, CH_2), 32.6 (CH_2), 71.6 (OCH_2), 120.0 (C-3, C-4), 134.9 (C-1, C-6), 145.5 (C-2, C-5) ppm; HRMS (APCI pos.): calcd. for $C_{30}H_{52}N_2O_6$: 536.3820 found: 536.3811. MS (APCI neg.): m/z (%) = 571.35 (100) $[M+Cl]^-$. HRMS (APCI neg.): calcd. for $C_{30}H_{52}N_2O_6Cl$: 571.3519 found: 571.3511. FT-IR (ATR): $\tilde{\nu}$ /cm^{-1} = 2955 (m), 2915 (vs), 2872 (m), 2849 (s), 1533 (vs), 1493 (s), 1467 (s), 1431 (m), 1395 (m), 1354 (s), 1276 (vs), 1188 (m), 1156 (m), 1072 (s), 1044 (m), 1034 (m), 1005 (m), 942 (w), 894 (m), 856 (w), 820 (s), 793 (w), 773 (w), 753 (w), 720 (m) cm^{-1}. DSC: heating up (10 K/min) Cr 62 °C [-48.7 kJ mol^{-1}] I; cooling down (10 K/min) Cr 45 °C [43.4 kJ mol^{-1}] I; CHN-analysis: calcd. (%) for $C_{30}H_{52}N_2O_6$: C 67.13, H 9.77, N 5.22, found: C 67.26, H 9.88, N 5.17.

2,3-Dinitro-1,4-bis(tetradecyloxy)benzene (L-Nitro(14)):

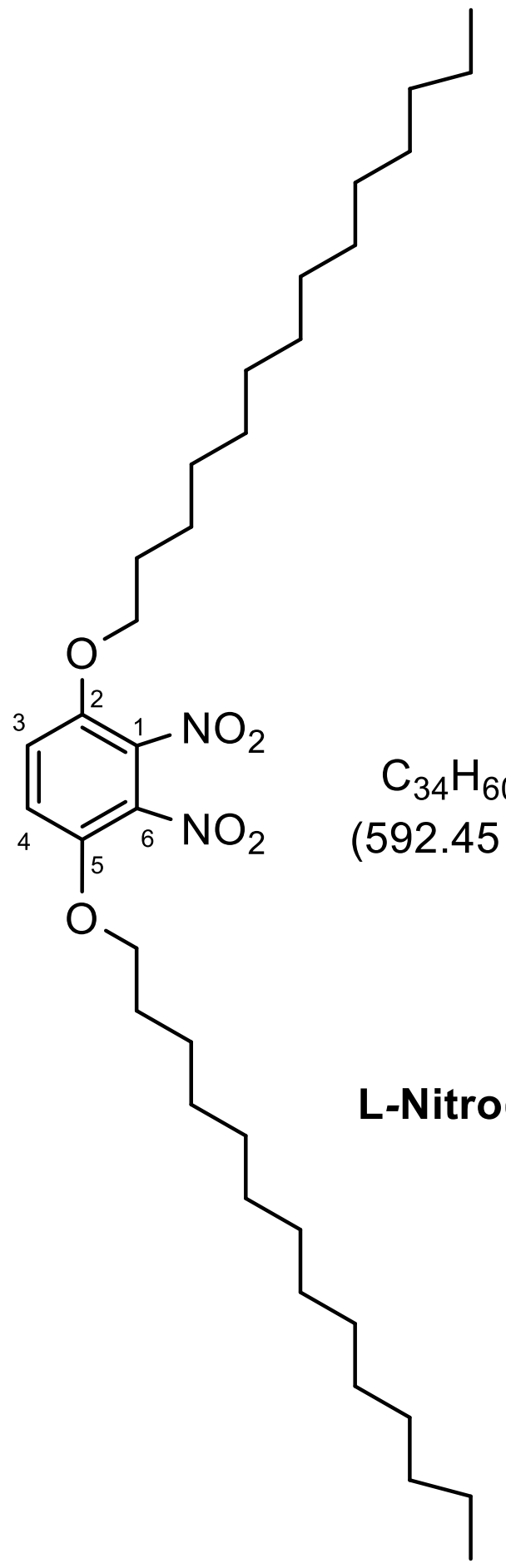

$C_{34}H_{60}N_2O_6$
(592.45 g mol^{-1})

L-Nitro(14)

According to **GP3**, 1,4-bisdodecyloxybenzene (5.00 g, 9.90 mmol), HNO_3 (100 %, 100 mL), 50 mL conc. AcOH; **L-Nitro(14)** (1.77 g 3.00 mmol, 30 %) as yellow crystals; ^{1}H NMR (700 MHz, $CDCl_3$): δ = 0.88 (t, J = 7.1 Hz, 6H, 2 x CH_3), 1.25-1.31 (m, 40H, CH_2), 1.38-1.43 (m, 4H, 2 x $O(CH_2)_2C\mathit{H}_2$), 1.74-1.78 (m, 4H, 2 x $OCH_2C\mathit{H}_2$), 4.05 (t, J = 6.5 Hz, 4H, 2 x OCH_2), 7.14 (s, 2H, 3-H, 4-H) ppm; ^{13}C NMR (175 MHz, $CDCl_3$): δ = 14.3 (CH_3), 22.8 (2 x $CH_3\mathit{C}H_2$), 25.8 ($O(CH_2)_2\mathit{C}H_2$), 29.0, 29.4, 29.5, 29.6, 29.7, 29.78, 29.79, 29.81, 29.83 ($OCH_2\mathit{C}H_2$, CH_2), 32.1 (CH_2), 71.2 (OCH_2), 117.9 (C-3, C-4), 134.8 (C-1, C-6), 145.0 (C-2, C-5) ppm; HRMS (APCI pos.): calcd. for $C_{34}H_{60}N_2O_6$: 592.4446; found: 592.4438; FT-IR (ATR): $\tilde{\nu}$ /cm^{-1} = 2913 (vs), 2850 (s), 1535 (vs), 1492 (m), 1472 (s), 1464 (s), 1428 (w), 1395 (w), 1348 (m), 1278 (s), 1184 (w), 1154 (m), 1072 (m), 1054 (w), 1036 (m), 1022 (m), 1012 (m), 992 (w), 924 (w), 887 (w), 846 (w), 813 (s), 791 (w), 754 (w), 713 (m) cm^{-1}; DSC: heating up (10 K/min) Cr_1 63 °C [-33.4 kJ mol^{-1}] Cr_2 70 °C [-68.0 kJ mol^{-1}] I; cooling down (10 K/min) Cr 57 °C [59.7 kJ mol^{-1}] I; CHN-analysis: calcd. (%) for $C_{34}H_{60}N_2O_6$: C 68.88, H 10.20, N 4.73, found: C 69.06, H 10.32, N 4.66.

2.3. Syntheses of U-shaped benzimidazoles (U-Bi(n))

5,6-Bis(pentyloxy)-benzoimidazole (U-Bi(5)):

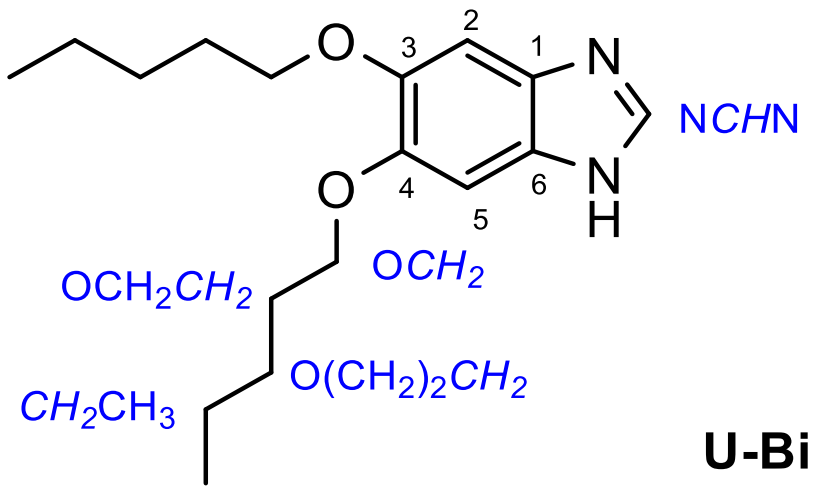

$C_{17}H_{26}N_2O_2$
(280.20 g mol^{-1})

U-Bi(5)

According to **GP4**, **U-Nitro(5)** (3.85 g, 11.2 mmol), Pd / C (10 % Pd, 2.0 g, 1.9 mmol Pd), 300 mL THF; introducing H_2 over 6 h at 60 °C, then stirring at 50 °C over night; 10 mL formic acid (90 %), 3 d 80 °C; purification: flash chromatography, initially using ethyl acetate, followed by a gradient elution with dichloromethane/methanol (40 : 1 → 20 : 1) and final recrystallization from acetone; **U-Bi(5)** (1.50 g, 5.17 mmol, 46 %) as light brown crystals; ^{1}H NMR (700 MHz, $CDCl_3$): δ = 0.91 (t, *J* = 7.3 Hz, 6H, 2 x CH_3), 1.35-1.40 (m, 4H, 2 x CH_2CH_3), 1.43-1.48 (m, 4H, 2 x $O(CH_2)_2CH_2$), 1.81-1.95 (m, 4H, 2 x OCH_2CH_2), 3.98 (t, *J* = 6.6 Hz, 4H, 2 x OCH_2), 7.12 (s, 2H, 2-H, 5-H), 7.92 (s, 1H, NCHN) ppm; ^{13}C NMR (175 MHz, $CDCl_3$): δ = 14.2 (2 x CH_3), 22.6 (2 x CH_3CH_2), 28.4 (2 x $O(CH_2)_2CH_2$), 29.1 (2 x OCH_2CH_2), 70.0 (2 x OCH_2), 100.3 (br, C-2, C-5), 131.7 (br, C-1, C-6), 139.4 (NCHN), 147.4 (C-3, C-4) ppm; MS (ESI): m/z (%) = 291.21 [$M+H^+$]; HRMS (ESI): calcd. for $C_{17}H_{26}N_2O_2+H^+$: 291.2067, found: 291.2060; FT-IR (ATR): $\tilde{\nu}$ /cm^{-1} = 3060 (w), 2956 (s), 2929 (s), 2858 (s), 2805 (m), 2759 (m), 2666 (m), 1490 (s), 1466 (vs), 1433 (m), 1420 (m), 1399 (w), 1379 (w), 1324 (vs), 1267 (m), 1237 (m), 1213 (vs), 1181 (vs), 1113 (m), 1051 (m), 1027 (w), 984 (m), 961 (m), 925 (m), 872 (m), 823 (m) cm^{-1}; CHN-analysis: calcd. (%) for $C_{17}H_{26}N_2O_2$: C 70.31, H 9.02, N 9.65, found: C 70.38, H 9.06, N 9.57.

5,6-Bis(hexyloxy)-benzoimidazole (U-Bi(6)):

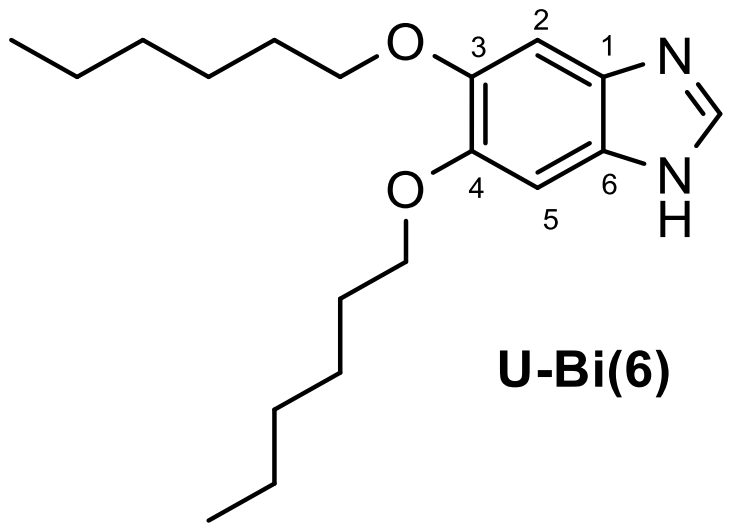

$C_{19}H_{30}N_2O_2$
(319,230 g mol^{-1})

U-Bi(6)

According to **GP4**, **U-Nitro(6)** (4.37 g, 11.9 mmol), Pd / C (10 % Pd, 1.50 g, 1.40 mmol Pd), 250 mL THF; introducing H_2 over 5 h at 50 °C, then stirring at 50 °C over night; 20 mL formic acid (90 %), 3 h 80 °C; purification: flash chromatography, initially using ethyl acetate, followed by a gradient elution with dichloromethane/methanol (20 : 1 → 10 : 1) and final recrystallization from acetone; **U-Bi(6)** (2.05 g, 6.44 mmol, 54 %) as light brown crystals; ^{1}H NMR (700 MHz, $CDCl_3$): δ = 0.89 (t, *J* = 6.6 Hz, 6H, 2 x CH_3), 1.30-1.35 (m, 8H, CH_2), 1.45-1.49 (m, 4H, 2 x $O(CH_2)_2CH_2$), 1.79-1.84 (m, 4H, 2 x

OCH$_2$C*H$_2$*), 4.00 (t, *J* = 6.6 Hz, 4H, 2 x OCH$_2$), 7.12 (s, 2H, 2-H, 5-H), 7.92 (s, 1H, NCHN) ppm; ^{13}C NMR (175 MHz, CDCl$_3$): □ = 14.2 (2 x CH$_3$), 22.7 (2 x CH$_3$*C*H$_2$), 25.9 (O(CH$_2$)$_2$*C*H$_2$), 29.4 (2 x OCH$_2$*C*H$_2$, CH$_2$), 31.8 (CH$_2$), 70.0 (2 x OCH$_2$), 100.4 (br, C-2, C-5), 131.7 (br, C-1, C-6), 139.4 (NCHN), 147.4 (C-3, C-4) ppm; MS (ESI): m/z (%) = 319.24 [M+H$^+$]; HRMS (ESI): calcd. for $C_{19}H_{31}N_2O_2$+H$^+$: 319.2380, found: 319.2374; FT-IR (ATR): $\tilde{\nu}$ /cm^{-1} = 3056 (w), 2955 (s), 2924 (s), 2857 (s), 2652 (m), 1490 (s), 1465 (vs), 1433 (m), 1419 (m), 1399 (w), 1379 (w), 1324 (vs), 1265 (m), 1236 (m), 1212 (vs), 1174 (vs), 1112 (s), 1069 (w), 1047 (m), 996 (m), 961 (s), 943 (m), 929 (m), 889 (m), 823 (s), 807 (m), 727 (w) cm^{-1}; CHN-analysis: calcd. (%) for $C_{19}H_{30}N_2O_2$: C 71.66, H 9.50, N 8.80, calcd. (%) for $C_{22}H_{36}N_2O_2$ + 0.1 Äquiv. H_2O: C 71.26, H 9.50, N 8.75, found: C 71.18, H 9.50, N 8.72.

5,6-Bis(heptyloxy)-benzoimidazole (U-Bi(7)):

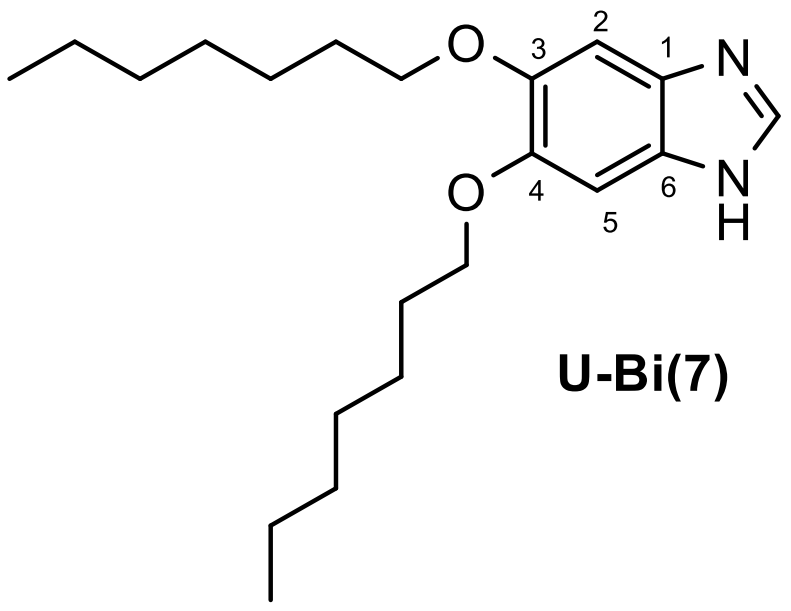

U-Bi(7)

$C_{21}H_{34}N_2O_2$
(346.26 g mol^{-1})

According to **GP4**, **U-Nitro(7)** (5.25 g, 13.24 mmol), Pd / C (10 % Pd, 2.0 g, 1.9 mmol Pd), 200 mL THF; introducing H$_2$ over 5 h at 50 °C, then stirring at 50 °C over night; 20 mL formic acid (90 %), 6 h 80 °C; purification: flash chromatography, initially using ethyl acetate, followed by a gradient elution with dichloromethane/methanol (20 : 1) and final recrystallization first from petrol ether and from MeCN; **U-Bi(7)** 1.19 g (3.43 mmol, 26 %) as light brown crystals; ^{1}H NMR (700 MHz, CDCl$_3$): δ = 0.88 (t, *J* = 6.9 Hz, 6H, 2 x CH$_3$), 1.67-1.36 (m, 12H, CH$_2$), 1.44-1.49 (m, 4H, 2 x O(CH$_2$)$_2$C*H$_2$*), 1.80-1.84 (m, 4H, 2 x OCH$_2$C*H$_2$*), 3.98 (t, *J* = 6.6 Hz, 4H, 2 x OCH$_2$), 7.12 (s, 2H, 2-H, 5-H), 7.92 (s, 1H, NCHN) ppm; ^{13}C NMR (175 MHz, CDCl$_3$): δ = 14.2 (2 x CH$_3$), 22.7 (2 x CH$_3$*C*H$_2$), 26.2 (O(CH$_2$)$_2$*C*H$_2$), 29.3, 29.5 (2 x OCH$_2$*C*H$_2$, CH$_2$), 31.8 (CH$_2$), 70.0 (2 x OCH$_2$), 100.4 (br, C-2, C-5), 132.1 (br, C-1, C-6), 139.4 (NCHN), 147.4 (C-3, C-4) ppm; MS (ESI): m/z (%) = 347.27 [M+H$^+$]; HRMS (ESI): calcd. for $C_{21}H_{34}N_2O_2$+H$^+$: 347.2693, found: 347.2684; FT-IR (ATR): $\tilde{\nu}$ /cm^{-1} = 3060 (w), 2956 (s), 2920 (s), 2851 (s), 2648 (w), 1465 (vs), 1432 (m), 1399 (w), 1379 (w), 1324 (vs), 1266 (m), 1235 (m), 1212 (vs), 1179 (vs), 1113 (s), 1064 (m), 1041 (w), 1006 (m), 961 (s), 901 (m), 823 (s), 808 (m), 724 (m), 619 (m) cm^{-1}; CHN-analysis: calcd. (%) for $C_{21}H_{34}N_2O_2$: C 72.79, H 9.89, N 8.08, found: C 73.05, H 9.92, N 8.01.

5,6-Bis(octyloxy)-benzoimidazole (U-Bi(8)):

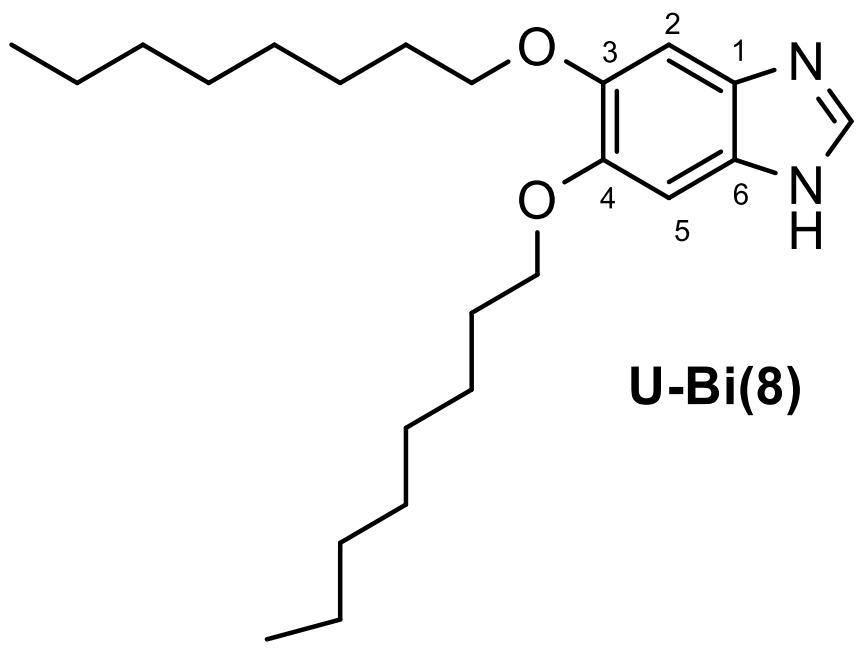

U-Bi(8)

$C_{23}H_{38}N_2O_2$
(374.29 g mol^{-1})

According to **GP4**, **U-Nitro(8)** (4.7 g, 10.9 mmol), Pd / C (10 % Pd, 1.0 g, 0.9 mmol Pd), 250 mL THF; introducing H_2 over 5 h at 60 °C, then stirring at 60 °C over night; 20 mL formic acid (90 %), 5 h 85 °C; purification: flash chromatography, initially using ethyl acetate, followed by a gradient elution with dichloromethane/methanol (20 : 1 → 10 : 1) and final recrystallization, first from heptane, then from MeCN; **U-Bi(8)** 0.90 g (2.40 mmol, 22 %) as light brown crystals; ^{1}H NMR (700 MHz, $CDCl_3$): δ = 0.88 (t, *J* = 7.0 Hz, 6H, 2 x CH_3), 1.25-1.37 (m, 16H, CH_2), 1.45-1.50 (m, 4H, 2 x $O(CH_2)_2C\mathit{H_2}$), 1.81-1.85 (m, 4H, 2 x $OCH_2C\mathit{H_2}$), 3.99 (t, *J* = 6.6 Hz, 4H, 2 x OCH_2), 7.13 (s, 2H, 2-H, 5-H), 7.91 (s, 1H, NCHN) ppm; ^{13}C NMR (175 MHz, $CDCl_3$): δ = 14.2 (2 x CH_3), 22.8 (2 x $CH_3\mathit{C}H_2$), 26.2 ($O(CH_2)_2\mathit{C}H_2$), 29.43, 29.47, 29.6 (2 x $OCH_2\mathit{C}H_2$, CH_2), 32.0 (CH_2), 70.1 (2 x OCH_2), 100.4 (br, C-2, C-5), 132.2 (br, C-1, C-6), 139.3 (NCHN), 147.4 (C-3, C-4) ppm; MS (ESI): m/z (%) = 375.30 [M+H$^+$]; HRMS (ESI): calcd. for $C_{23}H_{39}N_2O_2+H^+$: 375.3006, found: 375.3001; FT-IR (ATR): $\tilde{\nu}$ /cm^{-1} = 2955 (m), 2920 (s), 2852 (m), 1487 (m), 1465 (s), 1429 (m), 1323 (s), 1266 (m), 1241 (w), 1214 (m), 1180 (s), 1114 (m), 1062 (w), 1015 (w), 959 (m), 905 (vs), 874 (m), 839 (m), 810 (m), 725 (vs), 649 (m), 624 (m) cm^{-1}; CHN-analysis: calcd. (%) for $C_{23}H_{38}N_2O_2$: C 73.75, H 10.23, N 7.48, found: C 73.91, H 10.38, N 7.41.

5,6-Bis(nonyloxy)-benzoimidazole (U-Bi(9)):

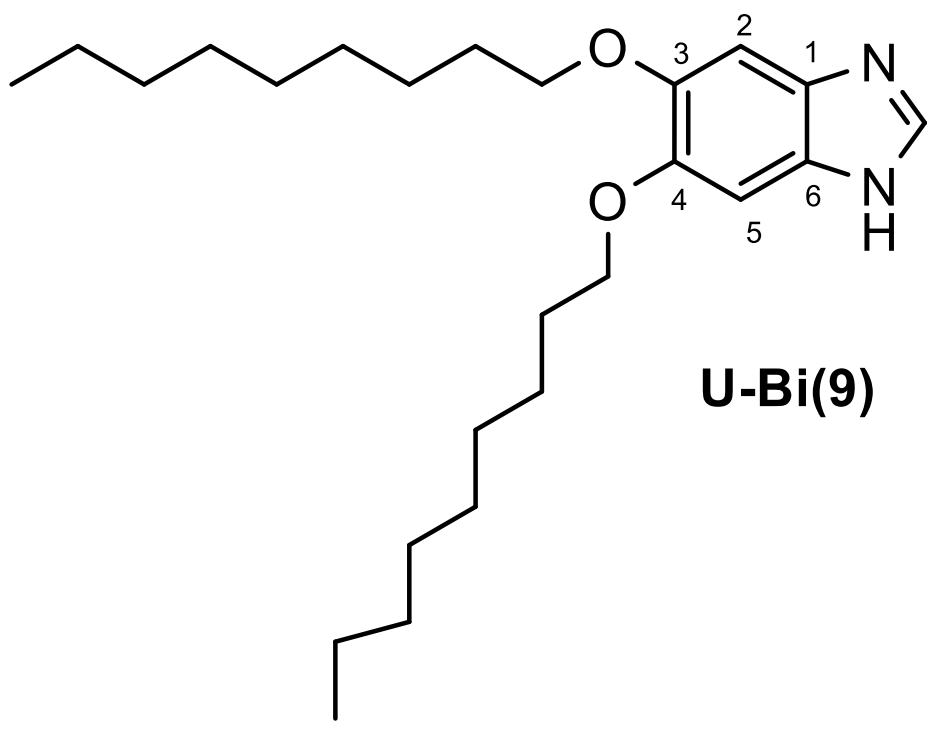

U-Bi(9)

$C_{25}H_{42}N_2O_2$
(402.32 g mol^{-1})

According to **GP4**, **U-Nitro(9)** (4.4 g, 9.72 mmol), Pd / C (10 % Pd, 2.0 g, 1.9 mmol Pd), 200 mL THF; introducing H_2 over 5 h at 50 °C, then stirring at 50 °C over night; 20 mL formic acid (90 %), 5 d at 80 °C; purification: recrystallization from acetone; **U-Bi(9)** (1.50 g, 3.73 mmol, 38 %) as light brown crystals; ^{1}H NMR (700 MHz, $CDCl_3$): δ = 0.87 (t, *J* = 7.1 Hz, 6H, 2 x CH_3), 1.25-1.37 (m, 20H, CH_2),

1.45-1.49 (m, 4H, 2 x O(CH$_2$)$_2$C*H*$_2$), 1.80-1.85 (m, 4H, 2 x OCH$_2$C*H*$_2$), 3.99 (t, *J* = 6.6 Hz, 4H, 2 x OCH$_2$), 7.12 (s, 2H, 2-H, 5-H), 7.91 (s, 1H, NCHN) ppm; ^{13}C NMR (175 MHz, CDCl$_3$): δ = 14.2 (2 x CH$_3$), 22.8 (2 x CH$_3$*C*H$_2$), 26.2 (O(CH$_2$)$_2$*C*H$_2$), 29.43, 29.47, 29.6 (2 x OCH$_2$*C*H$_2$, CH$_2$), 32.0 (CH$_2$), 70.1 (2 x OCH$_2$), 100.4 (br, C-2, C-5), 132.2 (br, C-1, C-6), 139.3 (NCHN), 147.4 (C-3, C-4) ppm; MS (ESI): m/z (%) = 403.33 [M+H$^+$]; HRMS (ESI): calcd. for $C_{25}H_{42}N_2O_2$+H$^+$: 403.3319, found: 403.3311; FT-IR (ATR): $\tilde{\nu}$ /cm^{-1} = 3055 (w), 2956 (m), 2919 (vs), 2849 (vs), 2678 (w), 1465 (vs), 1432 (m), 1420 (m), 1378 (w), 1324 (vs), 1264 (m), 1236 (m), 1212 (vs), 1180 (vs), 1112 (s), 1067 (m), 1011 (w), 960 (s), 928 (m), 916 (m), 886 (m), 824 (vs), 807 (s), 735 (m), 722 (m), 618 (m) cm^{-1}; CHN-analysis: calcd. (%) for $C_{25}H_{42}N_2O_2$: C 74.58, H 10.52, N 6.96, found: C 74.74, H 10.54, N 6.90.

5,6-Bis(decyloxy)-benzoimidazole (U-Bi(10)):

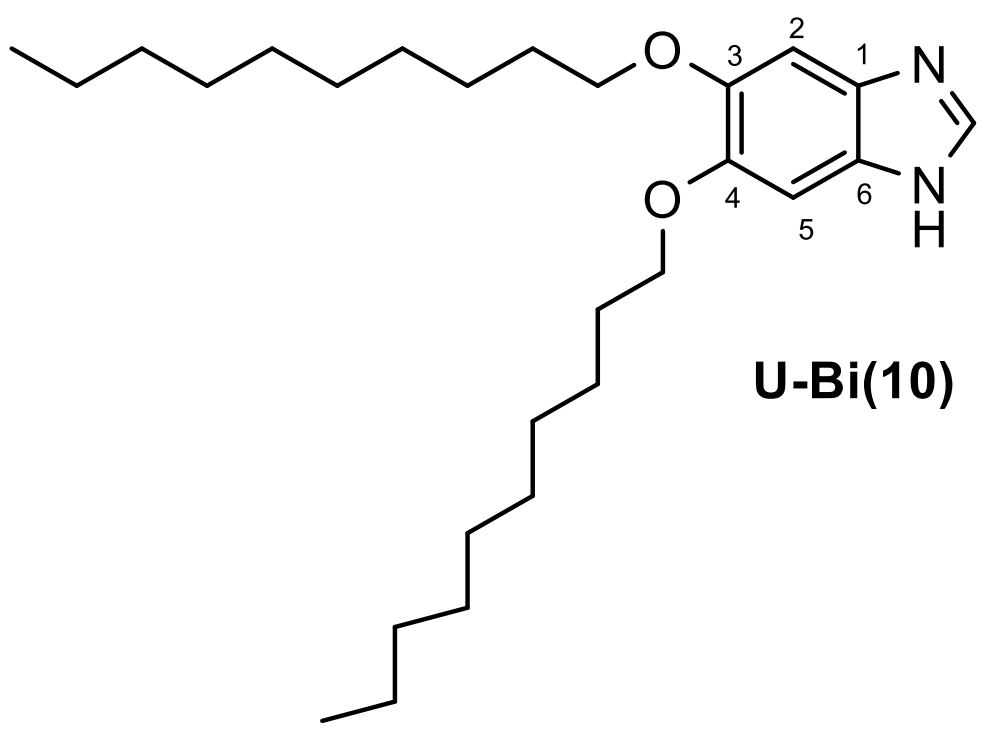

$C_{27}H_{46}N_2O_2$
(430.36 g mol^{-1})

According to **GP4**, **U-Nitro(10)** (11.0 g, 22.9 mmol), Pd / C (10 % Pd, 1.4 g, 1.3 mmol Pd), 300 mL THF; introducing H$_2$ over 5 h at 60 °C, then stirring 17 h at 60 °C; 20 mL formic acid (90 %), 5 h at 80 °C; purification: recrystallization from MeCN; **U-Bi(10)** (3.11 g, 7.22 mmol, 32 %) as light brown crystals; ^{1}H NMR (700 MHz, CDCl$_3$): δ = 0.87 (t, *J* = 7.1 Hz, 6H, 2 x CH$_3$), 1.26-1.36 (m, 24H, CH$_2$), 1.45-1.49 (m, 4H, 2 x O(CH$_2$)$_2$C*H*$_2$), 1.80-1.84 (m, 4H, 2 x OCH$_2$C*H*$_2$), 4.00 (t, *J* = 6.6 Hz, 4H, 2 x OCH$_2$), 7.13 (s, 2H, 2-H, 5-H), 7.91 (s, 1H, NCHN) ppm; ^{13}C NMR (175 MHz, CDCl$_3$): δ = 14.3 (2 x CH$_3$), 22.8 (2 x CH$_3$*C*H$_2$), 26.3 (O(CH$_2$)$_2$*C*H$_2$), 29.48, 29.50, 29.6, 29.74, 29.79 (2 x OCH$_2$*C*H$_2$, CH$_2$), 32.1 (CH$_2$), 70.1 (2 x OCH$_2$), 100.4 (br, C-2, C-5), 131.7 (br, C-1, C-6), 139.3 (NCHN), 147.5 (C-3, C-4) ppm; MS (ESI): m/z (%) = 431.36 [M+H$^+$]; HRMS (ESI): calcd. for $C_{27}H_{46}N_2O_2$+H$^+$: 431.3632, found: 431.3625.; FT-IR (ATR): $\tilde{\nu}$ /cm^{-1} = 2955 (m), 2918 (vs), 2849 (vs), 1504 (m), 1486 (m), 1466 (vs), 1411 (m), 1389 (w), 1324 (vs), 1266 (m), 1236 (m), 1218 (s), 1182 (vs), 1152 (m), 1117 (m), 1066 (w), 1047 (w), 1022 (w), 981 (m), 960 (m) cm^{-1}; CHN-analysis: calcd. (%) for $C_{27}H_{46}N_2O_2$: C 75.30, H 10.77, N 6.50, found: C 75.15, H 10.90, N 6.42.

5,6-Bis(dodecyloxy)-benzoimidazole (U-Bi(12)):

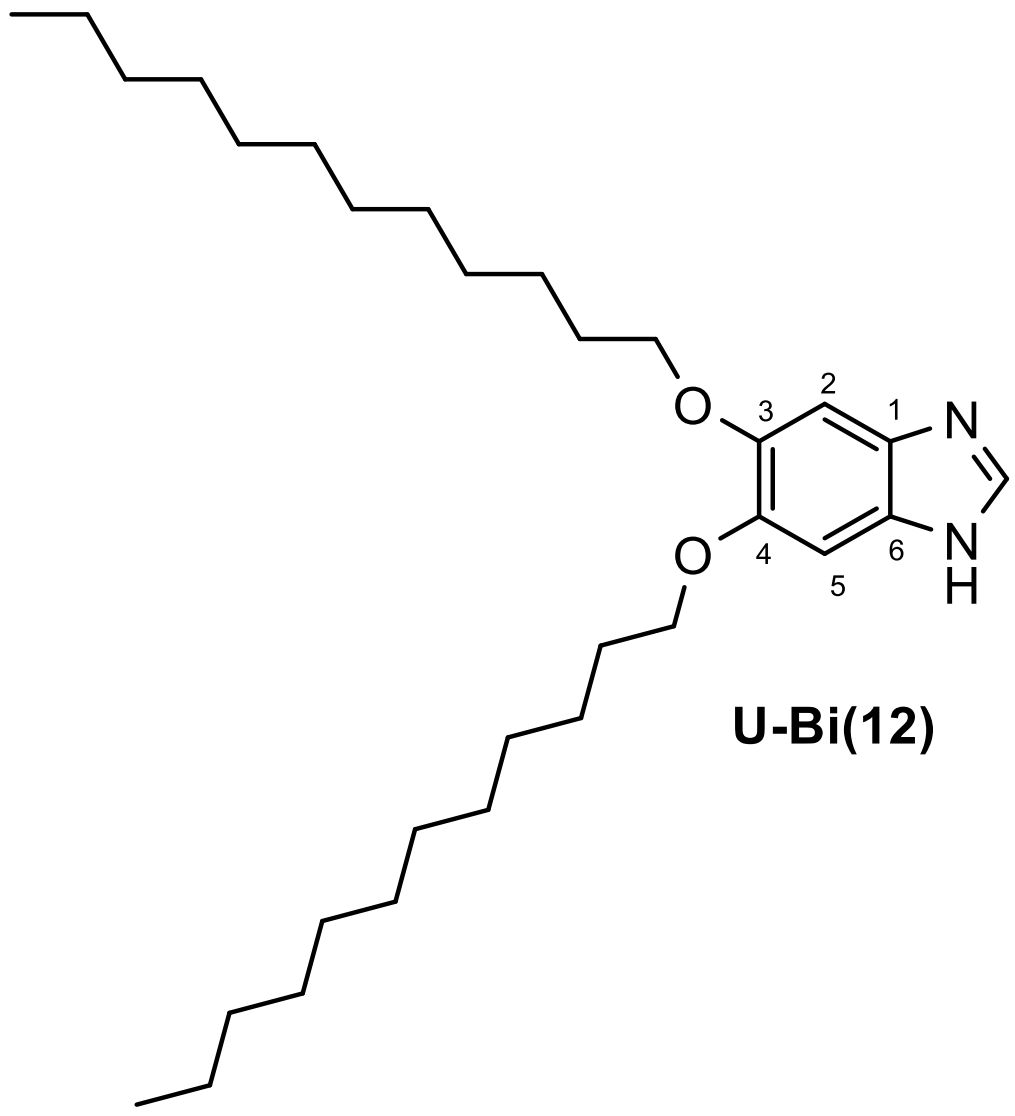

U-Bi(12)

$C_{31}H_{54}N_2O_2$
(486.42 g mol^{-1})

According to **GP4**, **U-Nitro(12)** (4.10 g, 7.64 mmol), Pd / C (10 % Pd, 1.4 g, 1.3 mmol Pd), 250 mL THF; introducing H_2 over 5 h at 50 °C, then stirring 24 h at 50 °C; 20 mL formic acid (90 %), 3 h at 80 °C; purification: recrystallization from EtOH; **U-Bi(12)** (2.20 g, 4.52 mmol, 59 %) as colorless solid; ^{1}H NMR (700 MHz, $CDCl_3$): δ = 0.88-0.90 (m, 6H, 2 x CH_3), 1.28-1.39 (m, 32H, CH_2), 1.47-1.51 (m, 4H, 2 x $O(CH_2)_2C\mathit{H_2}$), 1.81-1.85 (m, 4H, 2 x $OCH_2C\mathit{H_2}$), 4.01 (t, J = 6.6 Hz, 4H, 2 x OCH_2), 7.13 (s, 2H, 2-H, 5-H), 7.89, 7.89 (s, 1H, NCHN) ppm; ^{13}C NMR (175 MHz, $CDCl_3$): δ = 14.2 (2 x CH_3), 22.8 (2 x $CH_3\mathit{C}H_2$), 26.3 ($O(CH_2)_2\mathit{C}H_2$), 29.50, 29.63, 29.64, 29.80, 29.81, 29.85 (2 x $OCH_2\mathit{C}H_2$, CH_2), 32.1 (CH_2), 70.4 (2 x OCH_2), 101.4 (br, C-2, C-5), 132.0 (br, C-1, C-6), 139.2 (NCHN), 147.9 (C-3, C-4) ppm; MS (ASAP pos.): m/z (%) = 487.43 (100) [M+H$^+$], 319.24 (62) $[M-C_{12}H_{25}+2H]^+$, 151.05 (14) $[M-2C_{12}H_{25}+3H]^+$; HRMS (ASAP pos.): calcd. for $C_{31}H_{54}N_2O_2+H^+$: 487.4258, found: 487.4262; FT-IR (ATR): $\tilde{\nu}$ /cm^{-1} = 2955 (m), 2917 (vs), 2872 (m), 2849 (vs), 1503 (s), 1485 (s), 1464 (vs), 1410 (s), 1396 (m), 1322 (vs) cm^{-1}; CHN-analysis: calcd. (%) for $C_{31}H_{54}N_2O_2$: C 76.49, H 11.18, N 5.75, found: C 76.37, H 11.14, N 5.70.

5,6-Bis(tetradecyloxy)-benzoimidazole (U-Bi(14)):

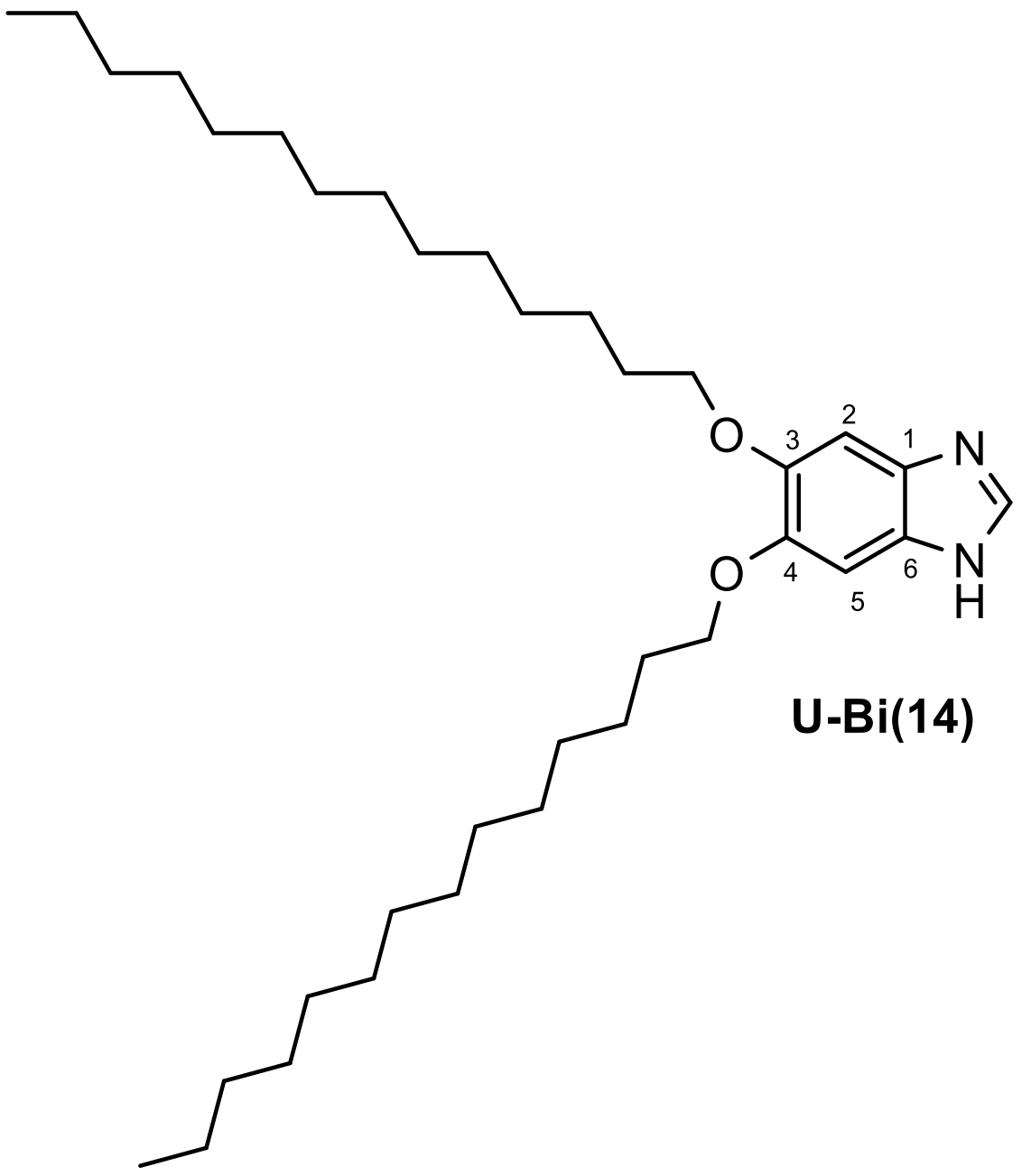

$C_{35}H_{62}N_2O_2$
(542.48 g mol^{-1})

U-Bi(14)

According to **GP4**, **U-Nitro(14)** (4.70 g, 7.93 mmol), Pd / C (10 % Pd, 1.4 g, 1.3 mmol Pd), 250 mL THF; introducing H_2 over 5 h at 60 °C, then stirring 15 h at 60 °C; 20 mL formic acid (90 %), 18 h at 85 °C; after cooling down, reaction mixture was poured into ice water and extracted with hot toluene (3 x 100 mL). Precipitation drops out during cooling. The residue was filtered off and washed with THF (2 x 100 mL); 2.13 g (3.92 mmol, 49 %) **U-Bi(14)** was obtained; ^{1}H NMR (700 MHz, $CDCl_3$): δ = 0.89 (t, *J* = 7.0 Hz, 6H, 2 x CH_3), 1.27-1.39 (m, 40H, CH_2), 1.47-1.52 (m, 4H, 2 x $O(CH_2)_2C\mathit{H}_2$), 1.81-1.85 (m, 4H, 2 x $OCH_2C\mathit{H}_2$), 4.01 (t, *J* = 6.6 Hz, 4H, 2 x OCH_2), 7.14 (s, 2H, 2-H, 5-H), 7.88 (s, 1H, NCHN) ppm; ^{13}C NMR (175 MHz, $CDCl_3$): δ = 14.2 (2 x CH_3), 22.8 (2 x $CH_3\mathit{C}H_2$), 26.3 ($O(CH_2)_2\mathit{C}H_2$),, 29.5, 29.6, 29.81, 29.86 (2 x $OCH_2\mathit{C}H_2$, CH_2), 32.1 (CH_2), 70.4 (2 x OCH_2), 101.0 (br, C-2, C-5), 131.9 (br, C-1, C-6), 139.1 (NCHN), 147.9 (C-3, C-4) ppm; MS (ESI): m/z (%) = 543.49 [M+H$^+$]; HRMS (ESI): calcd. for $C_{35}H_{62}N_2O_2$+H$^+$: 543.4884, found: 543.4876; FT-IR (ATR): $\tilde{\nu}$ /cm^{-1} = 2955 (m), 2917 (vs), 2872 (m), 2849 (vs), 1504 (m), 1486 (m), 1465 (vs), 1411 (m), 1396 (w), 1324 (vs), 1266 (m), 1236 (m), 1219 (s), 1182 (vs), 1152 (m), 1117 (s) cm^{-1}; CHN-analysis: calcd. (%) for $C_{35}H_{62}N_2O_2$: C 77.43, H 11.51, N 5.16, found: C 77.46, H 11.47, N 5.02.

2.4. Syntheses of linear benzimidazoles (L-Bi(n))

4,7-Bis(pentyloxy)-benzoimidazole (L-Bi(5)):

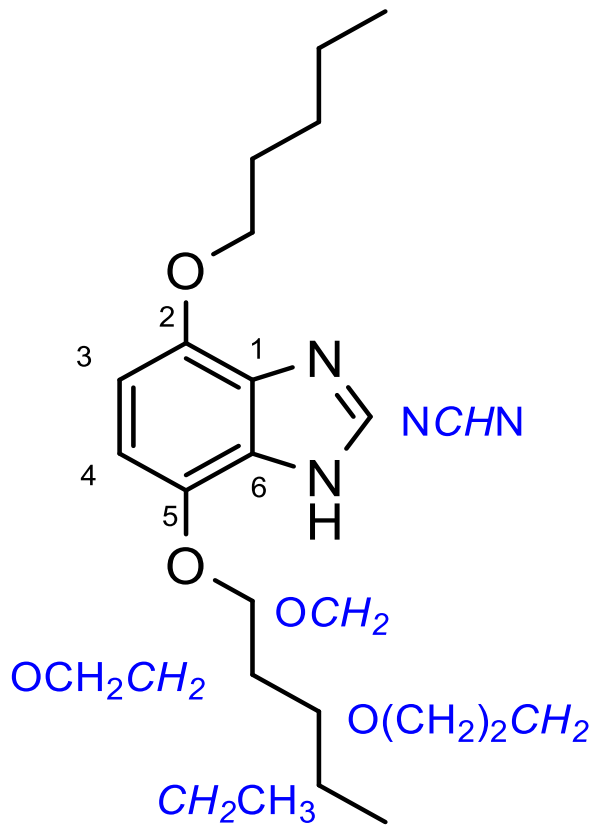

$C_{17}H_{26}N_2O_2$
(290.20 g mol^{-1})

L-Bi(5)

According to **GP4**, **L-Nitro(5)** (5.0 g, 14.7 mmol), Pd / C (10 % Pd, 2.5 g, 2.3 mmol Pd), 350 mL THF; introducing H_2 over 6 h at 50 °C, then stirring over night at 50 °C; 20 mL formic acid (90 %), 18 h at 85 °C; column chromatography with PE / EtOAc 2 : 1 → 1 : 1); recrystallization from EtOAc; 1.5 g (5.1 mmol, 35 %) **L-Bi(5)** as light brown solid; ^{1}H NMR (700 MHz, $CDCl_3$): δ = 0.86-0.90 (m, 6H, 2 x CH_3), 1.31-1.43 (m, 4H, 2 x $C\textit{H}_2CH_3$), 1.40-1.44 (m, 4H, 2 x $O(CH_2)_2C\textit{H}_2$), 1.76-1.86 (m, 4H, 2 x $OCH_2C\textit{H}_2$), 4.05 (t, J = 6.1 Hz, 2H, OCH_2), 4.14 (t, J = 6.2 Hz, 2H, OCH_2), 6.56 (s, 1H, 3-H or 4-H), 6.57 (s, 1H, 4-H or 3-H), 8.00 (s, 1H, NCHN), 10.57 (s, 1H, NH) ppm; ^{13}C NMR (175 MHz, $CDCl_3$): δ = 14.1 (2 x CH_3), 22.6 (2 x $\textit{C}H_2CH_3$), 28.4 (2 x $O(CH_2)_2\textit{C}H_2$), 29.2 (2 x $OCH_2\textit{C}H_2$), 68.8, 69.2 (OCH_2), 103.6 (C-3 or C-4), 104.2 (C-4 or C-3), 125.2 (C-Ar), 134.8 (C-Ar), 139.0 (NCHN), 140.2 (C-Ar), 145.6 (C-Ar) ppm; MS (ESI): m/z (%) = 291.21 [M+H$^+$]; HRMS (ESI): calcd. for $C_{17}H_{26}N_2O_2$+H$^+$: 291.2067, found: 291.2060; FT-IR (ATR): $\tilde{\nu}$ /cm^{-1} = 2953 (m), 2922 (s), 2862 (s), 2772 (m), 2597 (m), 1526 (vs), 1454 (vs), 1409 (m), 1389 (m), 1303 (s), 1285 (s), 1262 (vs), 1194 (m), 1163 (w), 1125 (w), 1097 (s), 1083 (vs), 1065 (s), 984 (m), 949 (s), 922 (m), 894 (m), 869 (m) cm^{-1}; CHN-analysis: calcd. (%) for $C_{17}H_{26}N_2O_2$: C 70.31, H 9.02, N 9.65, found: C 70.59, H 9.09, N 9.71.

4,7-Bis(hexyloxy)-benzoimidazole (L-Bi(6)):

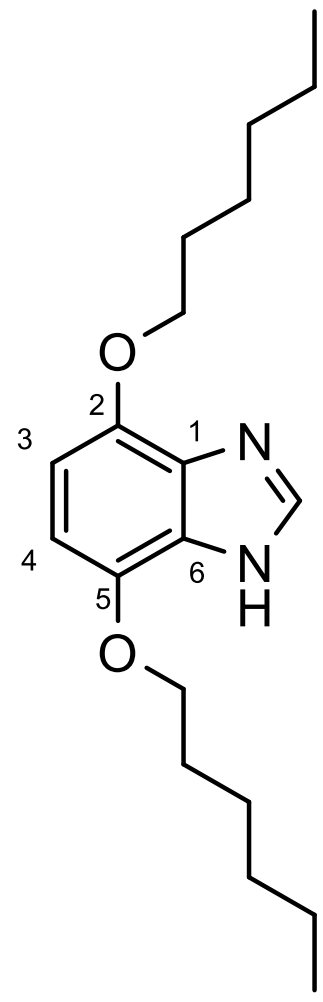

$C_{19}H_{30}N_2O_2$
(318.23 g mol^{-1})

L-Bi(6)

According to **GP4**, **L-Nitro(6)** (1.4 g, 3.9 mmol), Pd / C (10 % Pd, 1.5 g, 1.4 mmol Pd), 400 mL THF; introducing H_2 over 6 h at 50 °C, then stirring over night at 50 °C; 20 mL formic acid (90 %), 8 h at 85 °C; column chromatography with PE / EtOAc 2 : 1 → 1 : 1); recrystallization from CH_2Cl_2; .3 g (1.0 mmol, 26 %) **L-Bi(6)** as light brown solid; ^{1}H NMR (700 MHz, $(CD_3)_2SO$): δ = 0.87 (t, *J* = 6.8 Hz, 6H, 2 x CH_3), 1.27-1.33 (m, 8H, CH_2), 1.41-1.47 (m, 4H, 2 x $O(CH_2)_2C\mathit{H_2}$), 1.70-1.77 (m, 4H, 2 x $OCH_2C\mathit{H_2}$), 4.04 (t, *J* = 6.5 Hz, 2H, OCH_2), 4.11 (t, *J* = 6.5 Hz, 2H, OCH_2), 6.50 (d, *J* = 8.4 Hz, 1H, 3-H or 4-H), 6.58 (d, *J* = 8.4 Hz, 1H, 4-H or 3-H), 7.99 (s, 1H, NCHN), 12.59 (s, 1H, NH) ppm; ^{13}C NMR (175 MHz, $(CD_3)_2SO$)): δ = 14.0 (2 x CH_3), 22.1 (2 x CH_2, $\mathit{C}H_2CH_3$), 25.2, 25.3 ($O(CH_2)_2\mathit{C}H_2$), 28.9, 29.1 ($OCH_2\mathit{C}H_2$), 31.10, 31.12 (CH_2), 68.1, 68.5 (OCH_2), 103.9 (C-3, C-4), 125.2 (C-Ar), 135.0 (C-Ar), 139.96 (C-Ar), 140.01 (NCHN), 144.7 (C-Ar) ppm.; MS (ESI): m/z (%) = 319.24 [M+H$^+$]; HRMS (ESI): calcd. for $C_{19}H_{30}N_2O_2+H^+$: 319.2380, found: 319.2374; FT-IR (ATR): $\tilde{\nu}$ /cm^{-1} = 2924 (s), 2857 (m), 2771 (m), 2593 (m), 1526 (vs), 1458 (s), 1409 (w), 1390 (m), 1305 (s), 1285 (m), 1262 (vs), 1195 (w), 1126 (w), 1085 (vs), 972 (w), 949 (m), 921 (w), 776 (m), 649 (m) cm^{-1}; CHN-analysis: calcd. (%) for $C_{19}H_{30}N_2O_2$: C 71.66, H 9.49, N 8.80, found: C 71.49, H 9.43, N 8.71.

4,7-Bis(heptyloxy)-benzoimidazole (L-Bi(7)):

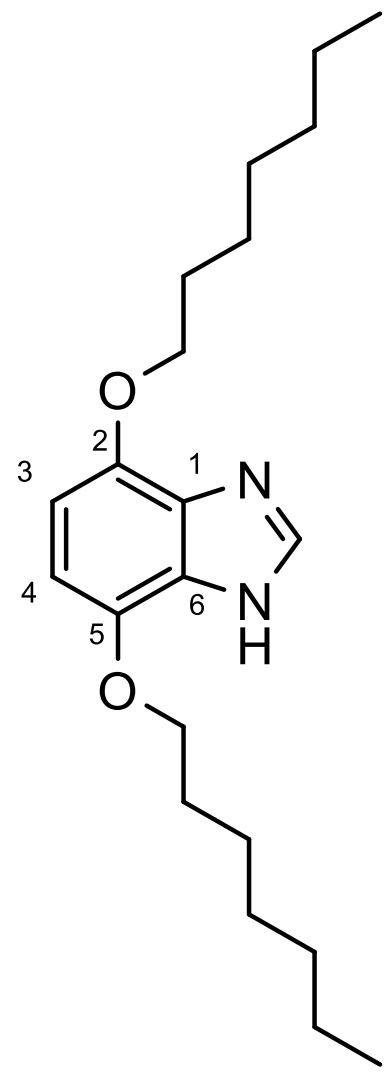

$C_{21}H_{34}N_2O_2$
(346.26 g mol^{-1})

L-Bi(7)

According to **GP4**, **L-Nitro(7)** (6.4 g, 16.1 mmol), Pd / C (10 % Pd, 2.3 g, 2.2 mmol Pd), 600 mL THF; introducing H_2 over 6 h at 60 °C, then stirring over night at 60 °C; 10 mL formic acid (90 %), 4 h at 85 °C; column chromatography with PE / EtOAc (2 : 1 → 1 : 1)); recrystallization from heptane and MeOH; 1.7 g (5.0 mmol, 31 %) **L-Bi(7)** as light brown solid; ^{1}H NMR (700 MHz, $(CD_3)_2SO$): δ = 0.86 (t, *J* = 6.9 Hz, 6H, 2 x CH_3), 1.27-1.35 (m, 12H, CH_2), 1.41-1.46 (m, 4H, 2 x $O(CH_2)_2C\mathit{H_2}$), 1.70-1.76 (m, 4H, 2 x $OCH_2C\mathit{H_2}$), 4.04 (t, *J* = 6.5 Hz, 2H, OCH_2), 4.11 (t, *J* = 6.5 Hz, 2H, OCH_2), 6.50 (d, *J* = 8.4 Hz, 1H, 3-H or 4-H), 6.58 (d, *J* = 8.4 Hz, 1H, 4-H or 3-H), 7.99 (s, 1H, NCHN), 12.59 (s, 1H, NH) ppm; ^{13}C NMR (175 MHz, , $(CD_3)_2SO$)): δ = 14.0 (2 x CH_3), 22.1 (2 x CH_2), 25.5, 25.6 ($O(CH_2)_2\mathit{C}H_2$), 28.55, 28.56 (CH_2), 28.9, 29.1 ($OCH_2\mathit{C}H_2$), 31.3 (2 x CH_2), 68.1, 68.5 (OCH_2), 103.9 (C-3, C-4), 125.2 (C-Ar), 135.0 (C-Ar), 139.96 (C-Ar), 140.01 (NCHN), 144.7 (C-Ar) ppm; MS (ESI): m/z (%) = 347.27 [M+H^{+}]; HRMS (ESI): calcd. for $C_{21}H_{34}N_2O_2$+H^{+}: 347.2693, found: 347.2687; FT-IR (ATR): $\tilde{\nu}$ /cm^{-1} = 2924 (s), 2854 (s), 2594 (w), 1525 (vs), 1457 (s), 1389 (m), 1304 (s), 1261 (vs), 1196 (m), 1083 (vs), 950 (m), 865 (m), 776 (m), 725 (w), 672 (w), 648 (m) cm^{-1}; CHN-analysis: calcd. (%) for $C_{21}H_{34}N_2O_2$: C 72.79, H 9.89, N 8.08, found: C 72.79, H 9.91, N 7.99.

4,7-Bis(octyloxy)-benzoimidazole (L-Bi(8)):

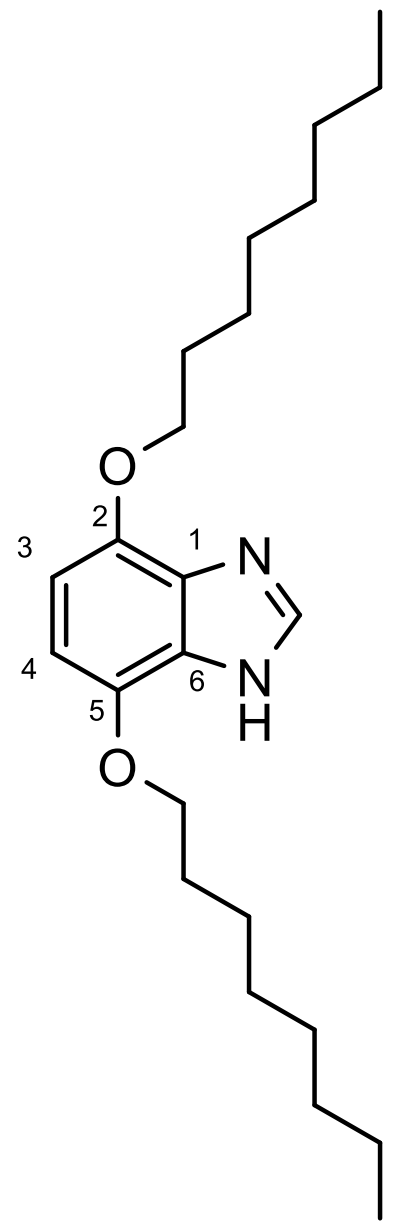

$C_{23}H_{38}N_2O_2$
(374.29 g mol^{-1})

L-Bi(8)

According to **GP4**, **L-Nitro(8)** (5.0 g, 11.9 mmol), Pd / C (10 % Pd, 2.1 g, 2.0 mmol Pd), 500 mL THF; introducing H_2 over 6 h at 60 °C, then stirring over night at 60 °C; 20 mL formic acid (90 %), 4 h at 85 °C; column chromatography with PE / EtOAc (2 : 1 → 1 : 1)); recrystallization from acetone; 1.5 g (4.0 mmol, 33 %) **L-Bi(8)** as light brown solid; ^{1}H NMR (700 MHz, $(CD_3)_2SO$): δ = 0.86 (t, *J* = 6.9 Hz, 6H, 2 x CH_3), 1.24-1.34 (m, 16H, CH_2), 1.42-1.46 (m, 4H, 2 x $O(CH_2)_2C\mathit{H_2}$), 1.71-1.76 (m, 4H, 2 x $OCH_2C\mathit{H_2}$), 4.04 (t, *J* = 6.1 Hz, 2H, OCH_2), 4.11 (t, *J* = 6.2 Hz, 2H, OCH_2), 6.51 (d, *J* = 8.3 Hz, 1H, 3-H or 4-H), 6.58 (d, *J* = 8.3 Hz, 1H, 4-H or 3-H), 7.99 (s, 1H, NCHN), 12.57 (s, 1H, NH) ppm; ^{13}C NMR (175 MHz, , $(CD_3)_2SO$)): δ = 14.0 (2 x CH_3), 22.1 (2 x CH_2), 25.5, 25.6 ($O(CH_2)_2\mathit{C}H_2$), 28.7, 28.8 (CH_2), 28.9, 29.1 ($OCH_2\mathit{C}H_2$), 31.3 (2 x CH_2), 68.1, 68.5 (OCH_2), 103.9 (C-3, C-4), 125.2 (C-Ar), 135.0 (C-Ar), 139.95 (C-Ar), 140.00 (NCHN), 144.7 (C-Ar) ppm; MS (ESI): m/z (%) = 375.30 [M+H$^+$]; HRMS (ESI): calcd. for $C_{23}H_{38}N_2O_2$+H$^+$: 375.3006, found: 375.2996; FT-IR (ATR): $\tilde{\nu}$ /cm^{-1} = 2921 (s), 2853 (s), 2590 (m), 1525 (vs), 1457 (s), 1408 (w), 1389 (m), 1304 (s), 1285 (m), 1262 (vs), 1195 (w), 1162 (w), 1125 (w), 1083 (vs), 976 (w), 947 (m), 866 (w), 776 (m), 763 (w), 724 (w), 649 (m) cm^{-1}; CHN-analysis: calcd. (%) for $C_{23}H_{38}N_2O_2$: C 73.75, H 10.23, N 7.48, found: C 73.99, H 10.33, N 7.40.

4,7-Bis(nonyloxy)-benzoimidazole (L-Bi(9)):

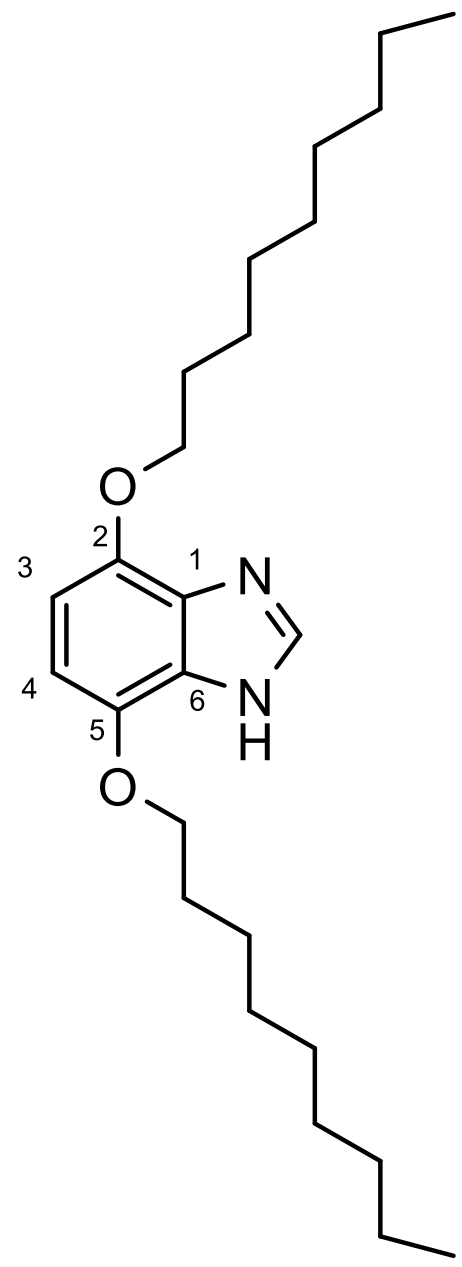

$C_{25}H_{42}N_2O_2$
(402.32 g mol^{-1})

L-Bi(9)

According to **GP4**, **L-Nitro(9)** (4.4 g, 9.7 mmol), Pd / C (10 % Pd, 2.1 g, 2.0 mmol Pd), 250 mL THF; introducing H_2 over 5 h at 60 °C, then stirring over night at 60 °C; 20 mL formic acid (90 %), 3 h at 85 °C; column chromatography with PE / EtOAc (5 : 1 → 1 : 1)); recrystallization from acetone; 12.5 g (6.3 mmol, 65 %) **L-Bi(9)** as light brown solid; ^{1}H NMR (500 MHz, $(CD_3)_2SO$): δ = 0.84 (t, *J* = 6.8 Hz, 6H, 2 x CH_3), 1.24-1.32 (m, 20H, CH_2), 1.39-1.42 (m, 4H, 2 x $O(CH_2)_2C\mathit{H_2}$), 1.69-1.77 (m, 4H, 2 x $OCH_2C\mathit{H_2}$), 4.02 (t, *J* = 6.4 Hz, 2H, OCH_2), 4.11 (t, *J* = 6.4 Hz, 2H, OCH_2), 6.49 (d, *J* = 8.4 Hz, 1H, 3-H or 4-H), 6.57 (d, *J* = 8.4 Hz, 1H, 4-H or 3-H), 7.99 (s, 1H, NCHN), 12.60 (s, 1H, NH) ppm; ^{13}C NMR (125 MHz, , $(CD_3)_2SO$)): δ = 13.9 (2 x CH_3), 22.1 (2 x CH_2), 25.5, 25.6 ($O(CH_2)_2\mathit{C}H_2$), 28.7, 28.9 (CH_2), 29.0, 29.1 ($OCH_2\mathit{C}H_2$), 31.3 (2 x CH_2), 68.1, 68.5 (OCH_2), 103.9 (C-3, C-4), 125.2 (C-Ar), 135.0 (C-Ar), 140.0 (NCHN), 144.7 (C-Ar) ppm; MS (ESI): m/z (%) = 403.33 [M+H^{+}]; HRMS (ESI): calcd. for $C_{25}H_{42}N_2O_2+H^+$: 403.3319, found: 403.3317; FT-IR (ATR): $\tilde{\nu}$ /cm^{-1} = 2920 (s), 2851 (s), 2660 (w), 2591 (w), 1525 (vs), 1453 (s), 1408 (w), 1389 (m), 1304 (s), 1282 (s), 1262 (vs), 1197 (w), 1082 (vs), 948 (m), 911 (w), 861 (m), 775 (m), 724 (w), 648 (m) cm^{-1}; CHN-analysis: calcd. (%) for $C_{25}H_{42}N_2O_2$: C 74.58, H 10.51, N 6.89, found: C 74.72, H 10.63, N 6.89.

4,7-Bis(decyloxy)-benzoimidazole (L-Bi(10)):

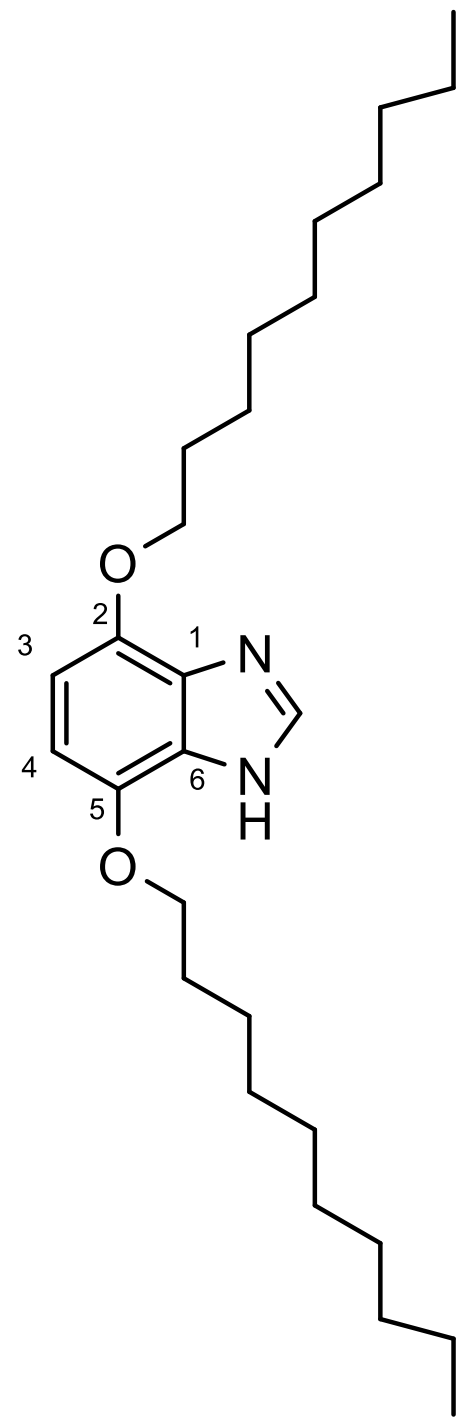

$C_{27}H_{46}N_2O_2$
(430.68 g mol^{-1})

L-Bi(10)

According to **GP4**, **L-Nitro(10)** (3.50 g, 7.28 mmol), Pd / C (10 % Pd, 1.0 g, 0.9 mmol Pd), 250 mL THF; introducing H_2 over 5 h at 60 °C, then stirring over night at 60 °C; 20 mL formic acid (90 %), 3 h at 85 °C; recrystallization from heptane; 1.39 g (3.23 mmol, 44 %) **L-Bi(10)** as light brown solid; ^{1}H NMR (700 MHz, $(CD_3)_2CO$): δ = 0.86-0.88 (m, 6H, 2 x CH_3), 1.29-1.36 (m, 24H, CH_2), 1.46-1.52 (m, 4H, 2 x $O(CH_2)_2CH_2$), 1.76-1.81 (m, 4H, 2 x OCH_2CH_2), 4.08 (t, *J* = 6.4 Hz, 2H, OCH_2), 4.24 (t, *J* = 6.5 Hz, 2H, OCH_2), 6.56 (d, *J* = 8.4 Hz, 1H, 3-H or 4-H), 6.62 (d, *J* = 8.4 Hz, 1H, 4-H or 3-H), 8.01 (s, 1H, NCHN), 11.93 (s, 1H, NH) ppm; ^{13}C NMR (175 MHz, $(CD_3)_2CO$)): δ = 14.4 (2 x CH_3), 23.3 (2 x CH_2), 26.8, 25.9 ($O(CH_2)_2CH_2$), 30.1, 30.19, 30.20, 30.23, 30.3, 30.4, 30.5 (2 x OCH_2CH_2, CH_2), 32.6 (2 x CH_2), 69.2, 70.0 (OCH_2), 104.8, 105.6 (C-3, C-4 or C-4, C-3), 126.5 (C-Ar), 136.5 (C-Ar), 140.2 (NCHN), 141.4 (C-Ar), 146.4 (C-Ar) ppm; MS (ESI): m/z (%) = 431.36 [M+H$^+$]; HRMS (ESI): calcd. for $C_{27}H_{46}N_2O_2+H^+$: 431.3632, found: 431.3627; FT-IR (ATR): $\tilde{\nu}$ /cm^{-1} = 2920 (s), 2850 (s), 2595 (w), 1525 (s), 1456 (s), 1408 (w), 1389 (m), 1304 (s), 1284 (m), 1260 (vs), 1195 (m), 1161 (w), 1079 (vs), 981 (w), 948 (m), 865 (m), 776 (s), 763 (m), 722 (m), 673 (w), 648 (s), 562 (w) cm^{-1}; CHN-analysis: calcd. (%) for $C_{27}H_{46}N_2O_2$: C 75.30, H 10.77, N 6.50, found: C 75.39, H 10.83, N 6.47.

4,7-Bis(dodecyloxy)-benzoimidazole (L-Bi(12)):

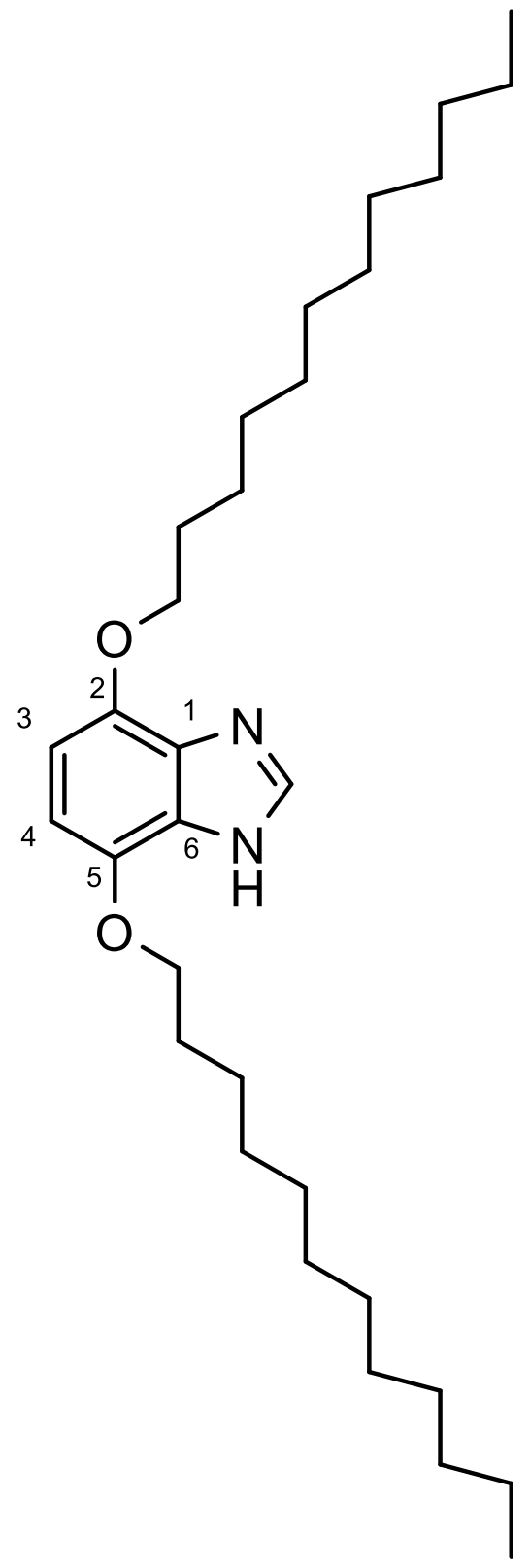

$C_{31}H_{54}N_2O_2$
(486.42 g mol^{-1})

L-Bi(12)

According to **GP4**, **L-Nitro(12)** (4.50 g, 8.38 mmol), Pd / C (10 % Pd, 1.7 g, 1.6 mmol Pd), 250 mL THF; introducing H_2 over 5 h at 50 °C, then stirring over night at 50 °C; 20 mL formic acid (90 %), 5 h at 80 °C; recrystallization from acetone; 2.22 g (4.55 mmol, 54 %) **L-Bi(12)** as light brown solid; ^{1}H NMR (700 MHz, $CDCl_3$): δ = 0.88 (t, *J* = 7.1 Hz, 6H, 2 x CH_3), 1.25-1.33 (m, 32H, CH_2), 1.43-1.47 (m, 4H, 2 x $O(CH_2)_2CH_2$), 1.80-1.84 (m, 4H, 2 x OCH_2CH_2), 4.10 (t, *J* = 6.7 Hz, 4H, 2 x OCH_2), 6.57 (s, 2H, 3-H, 4-H), 7.98 (s, 1H, NCHN) ppm; ^{13}C NMR (175 MHz, $CDCl_3$): δ = 14.3 (2 x CH_3), 22.8 (2 x CH_3CH_2), 26.2 ($O(CH_2)_2CH_2$), 29.50, 29.53, 29.6, 29.74, 29.77, 29.78, 29.80, 29.81 (2 x OCH_2CH_2, CH_2), 32.1 (CH_2), 69.1 (2 x OCH_2), 104.1 (C-3, C-4), 129.6 (br, C-1, C-6), 138.7 (NCHN), 142.8 (br, C-2, C-5) ppm; MS (ESI): m/z (%) = 487.43 [M+H$^+$]; HRMS (ESI): calcd. for $C_{31}H_{54}N_2O_2+H^+$: 487.4258, found: 487.4251; FT-IR (ATR): $\tilde{\nu}$ /cm^{-1} = 2919 (vs), 2850 (s), 2592 (w), 1525 (s), 1466 (s), 1456 (s), 1408 (w), 1389 (m), 1303 (s), 1284 (m), 1260 (vs), 1195 (w), 1160 (w), 1080 (vs), 970 (w), 946 (m), 869 (m), 776 (m), 761 (m), 722 (m), 673 (w), 648 (s), 561 (w) cm^{-1}; CHN-analysis: calcd. (%) for $C_{31}H_{54}N_2O_2$: C 76.49, H 11.18, N 5.75, found: C 76.51, H 11.19, N 5.76.

4,7-Bis(tetradecyloxy)-benzoimidazole (L-Bi(14)):

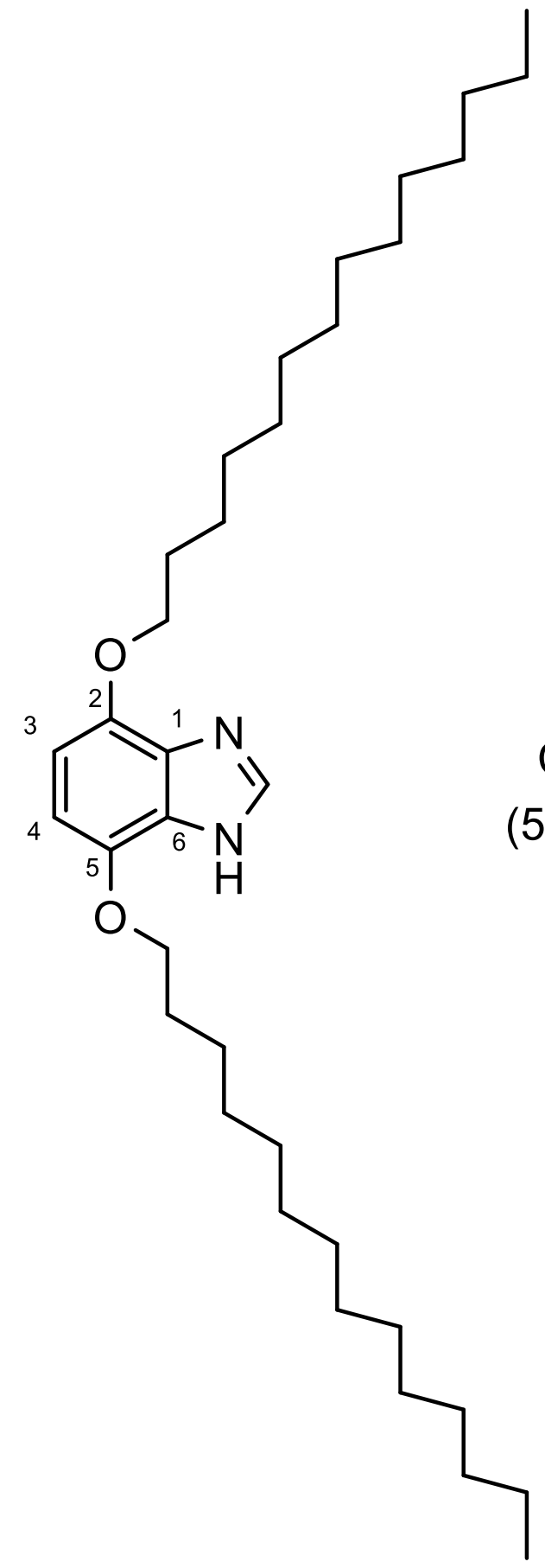

$C_{35}H_{62}N_2O_2$
(542.48 g mol^{-1})

L-Bi(14)

According to **GP4**, **L-Nitro(14)** (3.43 g, 5.79 mmol), Pd / C (10 % Pd, 0.8 g, 0.8 mmol Pd), 200 mL THF; introducing H_2 over 5 h at 50 °C, then stirring over night at 50 °C; 20 mL formic acid (90 %), 3 h at 85 °C; column chromatography with CH_2Cl_2 / MeOH (40 : 1 → 20 : 1); recrystallization from acetone; 1.35 g (2.49 mmol, 43 %) **L-Bi(14)** as light brown solid; ^{1}H

NMR (700 MHz, $CDCl_3$): δ = 0.88 (t, J = 7.1 Hz, 6H, 2 x CH_3), 1.25-1.34 (m, 40H, CH_2), 1.44-1.48 (m, 4H, 2 x $O(CH_2)_2\mathit{CH_2}$), 1.81-1.85 (m, 4H, 2 x $OCH_2\mathit{CH_2}$), 4.10 (t, J = 6.7 Hz, 4H, 2 x OCH_2), 6.57 (s, 2H, 3-H, 4-H), 7.96 (s, 1H, NCHN) ppm; ^{13}C NMR (175 MHz, $CDCl_3$): δ = 14.3 (2 x CH_3), 22.8 (2 x $CH_3\mathit{C}H_2$), 26.3 ($O(CH_2)_2\mathit{C}H_2$), 29.51, 29.54, 29.6, 29.75, 29.77, 29.80, 29.82, 29.83, 29.84 (2 x $OCH_2\mathit{C}H_2$, CH_2), 32.1 (CH_2), 69.1 (2 x OCH_2), 104.0 (C-3, C-4), 129.8 (br, C-1, C-6), 138.7 (NCHN), 142.9 (br, C-2, C-5) ppm; MS (ESI): m/z (%) = 543.49 [M+H$^+$]; HRMS (ESI): calcd. for $C_{35}H_{62}N_2O_2$+H$^+$: 543.4884, found: 543.4881; FT-IR (ATR): $\tilde{\nu}$ /cm^{-1} = 2918 (vs), 2848 (vs), 2599 (w), 1525 (s), 1467 (s), 1409 (w), 1388 (m), 1303 (s), 1285 (m), 1261 (vs), 1222 (w), 1194 (m), 1085 (vs), 948 (m), 932 (m), 865 (m), 777 (s), 745 (w), 721 (s), 673 (w), 648 (s), 561 (w) cm^{-1}; CHN-analysis: calcd. (%) for $C_{35}H_{62}N_2O_2$: C 77.43, H 11.51, N 5.16, found: C 77.62, H 11.61, N 4.99.

2.5. Syntheses of 5,6-bis(alkoxy)-1-methyl-benzoimidazole (U-MeBi(n))

5,6-Bis(pentyloxy)-1-methyl-benzoimidazole (U-MeBi(5)):

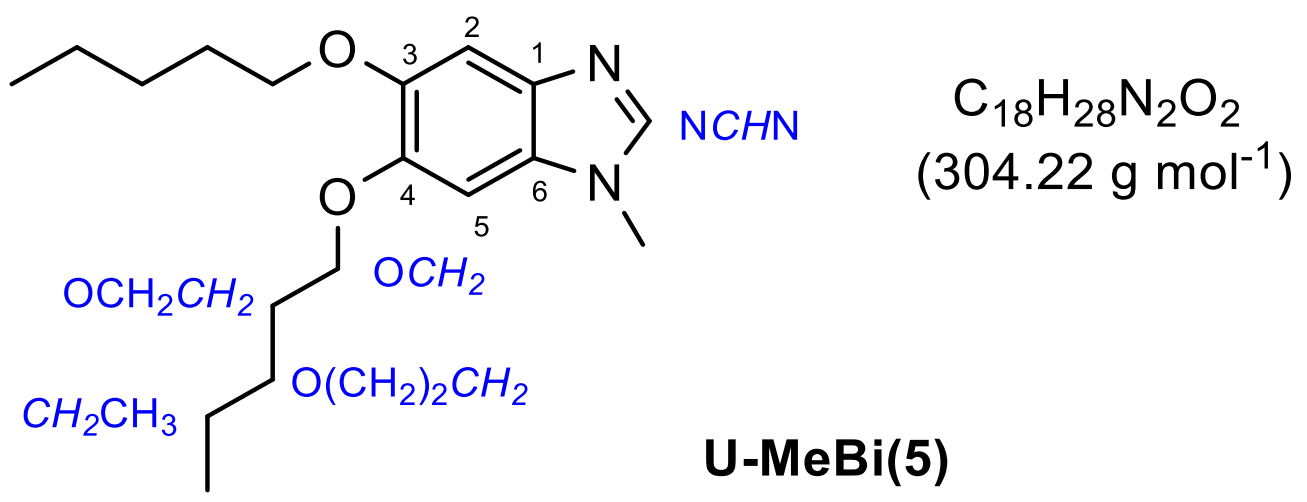

U-MeBi(5)

According to **GP5**, **U-Bi(5)** (1.01 g, 3.48 mmol), 20 mL THF; NaH (60 %, 0.15 g, 3.75 mmol); MeI (0.26 ml, 4.13 mmol), 1 h at room temperature; The mixture was applied directly to a silica gel column and eluted successively with diethyl ether, ethyl acetate, and CH_2Cl_2/MeOH (40 : 1); **U-MeBi(5)** (0.55 g, 1.81 mmol, 53 %); ^{1}H NMR (700 MHz, $CDCl_3$): δ = 0.92-0.95 (m, 6H, 2 x CH_3), 1.37-1.43 (m, 4H, 2 x C*H*$_2$CH$_3$), 1.46-1.51 (m, 4H, 2 x O(CH$_2$)$_2$C*H*$_2$), 1.83-1.89 (m, 4H, 2 x OCH$_2$C*H*$_2$), 3.76 (s, NCH_3), 4.01-4.04 (m, 4H, 2 x OCH_2), 6.83 (s, 1H, 5-H), 7.27 (s, 1H, 2-H), 7.69 (s, 1H, NCHN) ppm; ^{13}C NMR (175 MHz, $CDCl_3$): δ = 14.19, 14.20 (2 x CH_3), 22.63, 22.65 (2 x CH$_3$*C*H$_2$), 28.40, 28.42 (2 x O(CH$_2$)$_2$*C*H$_2$), 29.13, 29.19 (2 x OCH$_2$*C*H$_2$), 31.2 (NCH_3), 69.9, 70.2 (2 x OCH_2), 94.8 (C-5), 104.8 (C-2), 128.7 (Ar C, C-6 or C-1), 137.7 (Ar C, C-1 or C-6), 142.2 (NCHN), 146.9 (Ar C, C-4 or C-3), 147.7 (Ar C, C-3 or C-4) ppm; MS (ESI): m/z (%) = 305.22 [M+H$^+$]; HRMS (ESI): calcd. for $C_{18}H_{28}N_2O_2+H^+$: 305.2224, found: 305.2219; FT-IR (ATR): $\tilde{\nu}$ /cm^{-1} = 2954 (s), 2931 (s), 2861 (m), 1503 (vs), 1491 (s), 1468 (vs), 1452 (s), 1422 (m), 1367 (m), 1331 (m), 1271 (vs), 1228 (vs), 1204 (vs), 1148 (s), 1089 (vs), 1048 (m), 986 (m), 916 (m), 871 (m), 837 (m), 805 (m), 730 (w), 620 (m) cm^{-1}; DSC: heating up (5 K/min) Cr 63 °C [-24.4 kJ mol^{-1}] I; cooling down (5 K/min) Cr$_1$ 43 °C [10.4 kJ mol^{-1}] Cr$_2$ 43.8 °C [12.0 kJ mol^{-1}] Cr$_3$ 44.0 °C [4.4 kJ mol^{-1}] I; CHN-analysis: calcd. (%) for $C_{18}H_{28}N_2O_2$: C 71.02, H 9.27, N 9.20, found: C 70.91, H 9.26, N 8.99.

5,6-Bis(hexyloxy)-1-methyl-benzoimidazole (U-MeBi(6)):

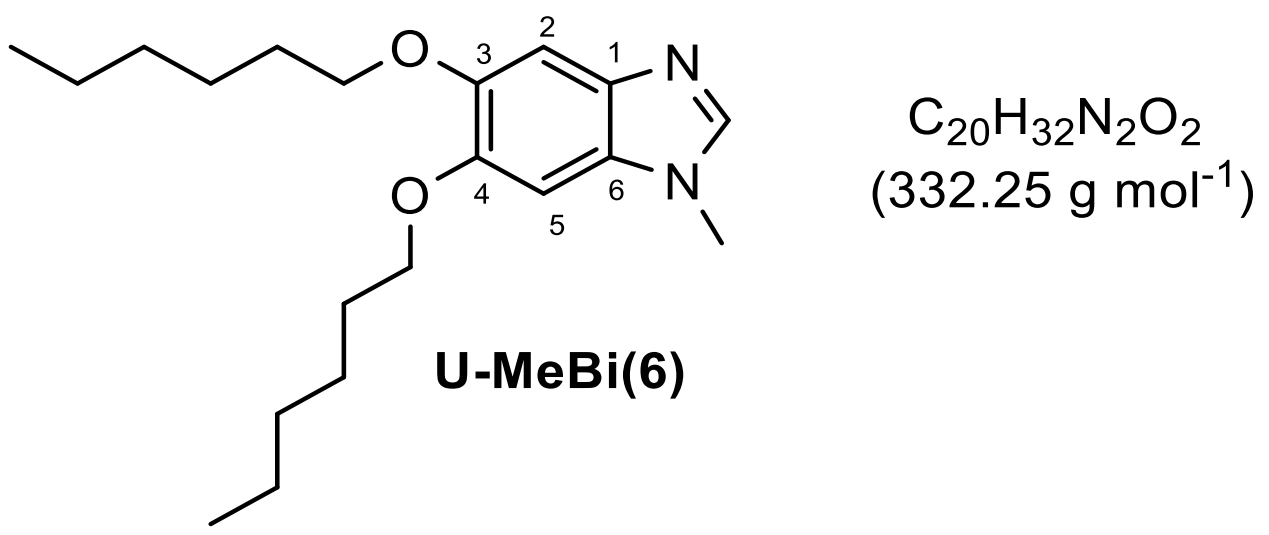

U-MeBi(6)

According to **GP5**, **U-Bi(6)** (2.04 g, 6.41 mmol), 20 mL THF; NaH (60 %, 0.31 g, 7.68 mmol); MeI (0.50 ml, 8.03 mmol), 1 h at room temperature; The mixture was applied directly to a silica gel column and purified by column chromatography using petroleum ether/ethyl acetate (5 : 1 → 2:1) followed by CH_2Cl_2/MeOH (40 : 1) as eluents. The purified compound **U-MeBi(6)** was recrystallised from MeOH

to afford the product as a solid (1.66 g, 4.99 mmol, 78 %); ^{1}H NMR (700 MHz, $CDCl_3$): δ = 0.89-0.92 (m, 6H, 2 x CH_3), 1.34-1.38 (m, 8H, 2 x $CH_2CH_2CH_3$), 1.49-1.53 (m, 4H, 2 x $O(CH_2)_2CH_2$), 1.82-1.88 (m, 4H, 2 x OCH_2CH_2), 3.76 (s, NCH_3), 4.02-4.04 (m, 4H, 2 x OCH_2), 6.83 (s, 1H, 5-H), 7.27 (s, 1H, 2-H), 7.69 (s, 1H, NCHN) ppm; ^{13}C NMR (175 MHz, $CDCl_3$): δ = 14.2 (2 x CH_3), 22.8 (2 x CH_3CH_2), 25.90, 25.91 (2 x $O(CH_2)_2CH_2$), 29.41, 29.48 (2 x CH_2), 31.2 (NCH_3), 31.76, 31.78 (2 x CH_2), 69.9, 70.3 (2 x OCH_2), 94.9 (C-5), 104.8 (C-2), 128.7 (Ar C, C-6 or C-1), 137.7 (Ar C, C-1 or C-6), 142.2 (NCHN), 146.9 (Ar C, C-4 or C-3), 147.7 (Ar C, C-3 or C-4) ppm; MS (ESI): m/z (%) = 333.25 [$M+H^+$]; HRMS (ESI): calcd. for $C_{20}H_{32}N_2O_2+H^+$: 333.2537, found: 333.2530; FT-IR (ATR): $\tilde{\nu}$ /cm^{-1} = 2929 (s), 2858 (m), 1503 (s), 1490 (s), 1468 (s), 1452 (s), 1422 (m), 1367 (m), 1331 (m), 1271 (vs), 1228 (s), 1204 (s), 1148 (s), 1088 (s), 1043 (w), 1008 (m), 927 (m), 908 (m), 882 (m), 838 (m), 805 (m), 728 (vs), 642 (m), 619 (m) cm^{-1}; DSC: heating up (10 K/min) Cr 61 °C [-34.0 kJ mol^{-1}] I; cooling down (10 K/min) Cr_1 48 °C [br, 31.0 kJ mol^{-1}] Cr_2 43.8 °C [12.0 kJ mol^{-1}] Cr_3 44.0 °C [4.4 kJ mol^{-1}] I; CHN-analysis: calcd. (%) for $C_{20}H_{32}N_2O_2$: C 72.25, H 9.70, N 8.43, found: C 72.28, H 9.69, N 8.35.

5,6-Bis(heptyloxy)-1-methyl-benzoimidazole (U-MeBi(7)):

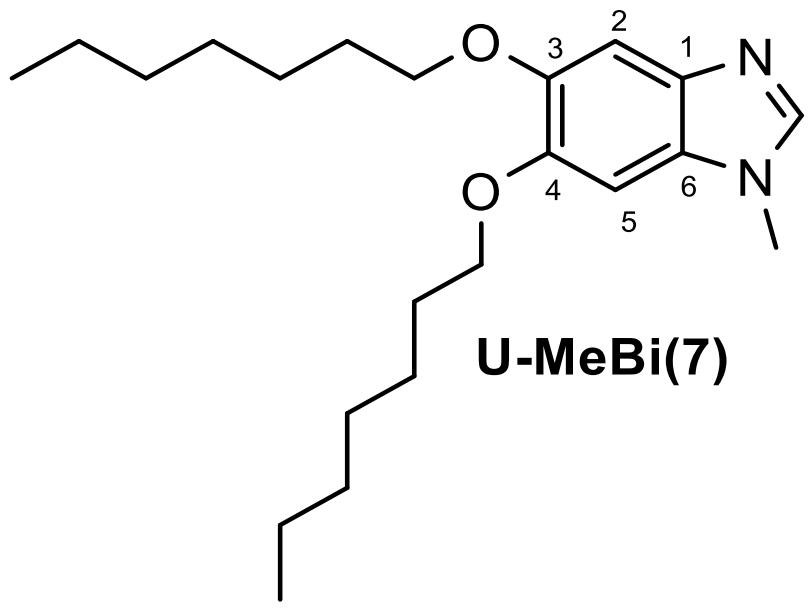

U-MeBi(7)

$C_{22}H_{36}N_2O_2$
(360.28 g mol^{-1})

According to **GP5**, **U-Bi(7)** (1.12 g, 3.23 mmol), 20 mL THF; NaH (60 %, 0.14 g, 3.50 mmol); MeI (0.24 ml, 3.86 mmol), 1 h at room temperature; The mixture was applied directly to a silica gel column and eluted successively with diethyl ether, ethyl acetate, and CH_2Cl_2/MeOH (40 : 1); purified by recrystallization from MeCN yielded **U-MeBi(7)** (0.55 g, 1.81 mmol, 53 %); ^{1}H NMR (700 MHz, $CDCl_3$): δ = 0.86-0.90 (m, 6H, 2 x CH_3), 1.28-1.40 (m, 12H, CH_2), 1.47-1.52 (m, 4H, 2 x $O(CH_2)_2CH_2$), 1.82-1.88 (m, 4H, 2 x OCH_2CH_2), 3.77 (s, NCH_3), 4.01-4.04 (m, 4H, 2 x OCH_2), 6.83 (s, 1H, 5-H), 7.27 (s, 1H, 2-H), 7.70 (s, 1H, NCHN) ppm; ^{13}C NMR (175 MHz, $CDCl_3$): δ = 14.2 (2 x CH_3), 22.8 (2 x CH_3CH_2), 26.19, 26.20 (2 x $O(CH_2)_2CH_2$), 29.26, 29.27, 29.46, 29.54 (2 x OCH_2CH_2, CH_2), 31.2 (NCH_3), 32.0 (CH_2), 69.9, 70.3 (2 x OCH_2), 94.9 (C-5), 104.8 (C-2), 128.7 (Ar C, C-6 or C-1), 137.7 (Ar C, C-1 or C-6), 142.2 (NCHN), 147.0 (Ar C, C-4 or C-3), 147.7 (Ar C, C-3 or C-4) ppm; MS (ESI): m/z (%) = 361.28 [$M+H^+$]; HRMS (ESI): calcd. for $C_{22}H_{36}N_2O_2+H^+$: 361.2850, found: 361.2841; FT-IR (ATR): $\tilde{\nu}$ /cm^{-1}= 3298 (w), 3103 (w), 2955 (s), 2924 (vs), 2856 (s), 1506 (vs), 1491 (vs), 1468 (vs), 1453 (s), 1422 (m), 1367 (m), 1333 (m), 1273 (vs), 1231 (vs), 1206 (vs), 1150 (s), 1091 (vs), 1010 (m), 967 (w), 948 (w), 836 (m), 804 (m), 724 (m) cm^{-1}; DSC: heating up (10 K/min) Cr_1 52 °C [-2.3 kJ mol^{-1}]

Cr_2 65 °C [-29.5 kJ mol^{-1}] I; cooling down (10 K/min) Cr_1 45 °C [2.4 kJ mol^{-1}] Cr_2 54 °C [29.3 kJ mol^{-1}] I; CHN-analysis: calcd. (%) for $C_{22}H_{36}N_2O_2$: C 73.29, H 10.06, N 7.77, found: C 73.36, H 10.05, N 7.70.

5,6-Bis(octyloxy)-1-methyl-benzoimidazole (U-MeBi(8)):

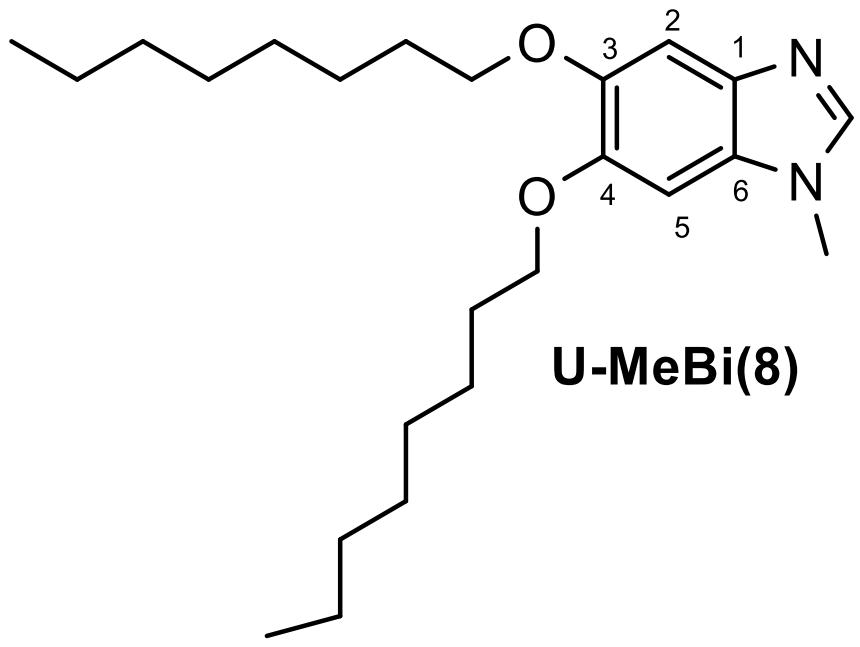

$C_{24}H_{40}N_2O_2$
(388,31 g mol^{-1})

U-MeBi(8)

According to **GP5**, **U-Bi(8)** (0.20 g, 0.53 mmol), 10 mL THF; NaH (60 %, 0.04 g, 1.08 mmol); MeI (1.06 mmol), 1 h at room temperature; The mixture was applied directly to a silica gel column and eluted successively with diethyl ether/ethyl acetate (5 : 1), and CH_2Cl_2/MeOH (40 : 1); **U-MeBi(8)** (0.55 g, 1.81 mmol, 53 %); ^{1}H NMR (700 MHz, $CDCl_3$): δ = 0.87-0.89 (m, 6H, 2 x CH_3), 1.27-1.39 (m, 16H, CH_2), 1.47-1.52 (m, 4H, 2 x $O(CH_2)_2C\mathit{H_2}$), 1.82-1.88 (m, 4H, 2 x $OCH_2C\mathit{H_2}$), 3.76 (s, NCH_3), 4.01-4.04 (m, 4H, 2 x OCH_2), 6.83 (s, 1H, 5-H), 7.27 (s, 1H, 2-H), 7.70 (s, 1H, NCHN) ppm; ^{13}C NMR (175 MHz, $CDCl_3$): δ = 14.2 (2 x CH_3), 22.8 (2 x $CH_3\mathit{C}H_2$), 26.24, 26.25 (2 x $O(CH_2)_2\mathit{C}H_2$), 29.43, 29.46, 29.53, 29.56, 29.57 (2 x $OCH_2\mathit{C}H_2$, CH_2), 31.2 (NCH_3), 32.0 (CH_2), 69.9, 70.3 (2 x OCH_2), 94.9 (C-5), 104.8 (C-2), 128.7 (Ar C, C-6 or C-1), 137.7 (Ar C, C-1 or C-6), 142.2 (NCHN), 147.0 (Ar C, C-4 or C-3), 147.7 (Ar C, C-3 or C-4) ppm; MS (EI pos.): m/z (%) = 388.3 (58) $[M]^+$, 276.2 (24) $[M-C_8H_{17}+H]^+$, 164.1 (100) $[M-2C_8H_{17}+2H]^+$; HRMS (EI pos.): calcd. for $C_{24}H_{40}N_2O_2$: 388.3084, found: 388.3086; FT-IR (ATR): $\tilde{\nu}$ /cm^{-1} = 3359 (w), 3100 (w), 2954 (s), 2923 (vs), 2855 (s), 1506 (s), 1492 (s), 1468 (s), 1454 (m), 1422 (m), 1368 (m), 1333 (m), 1274 (s), 1231 (s), 1206 (s), 1151 (m), 1092 (m), 1017 (w), 965 (w) cm^{-1}; DSC: heating up (10 K/min) Cr_1 11 °C [-4.2 kJ mol^{-1}] Cr_2 68 °C [-37.8 kJ mol^{-1}] I; cooling down (10 K/min) Cr_1 4 °C [3.2 kJ mol^{-1}] Cr_2 47 °C [37.0 kJ mol^{-1}] I; CHN-analysis: calcd. (%) for $C_{24}H_{40}N_2O_2$: C 74.18, H 10.38, N 7.21, found: C 74.00, H 10.41, N 7.09.

5,6-Bis(nonyloxy)-1-methyl-benzoimidazole (U-MeBi(9)):

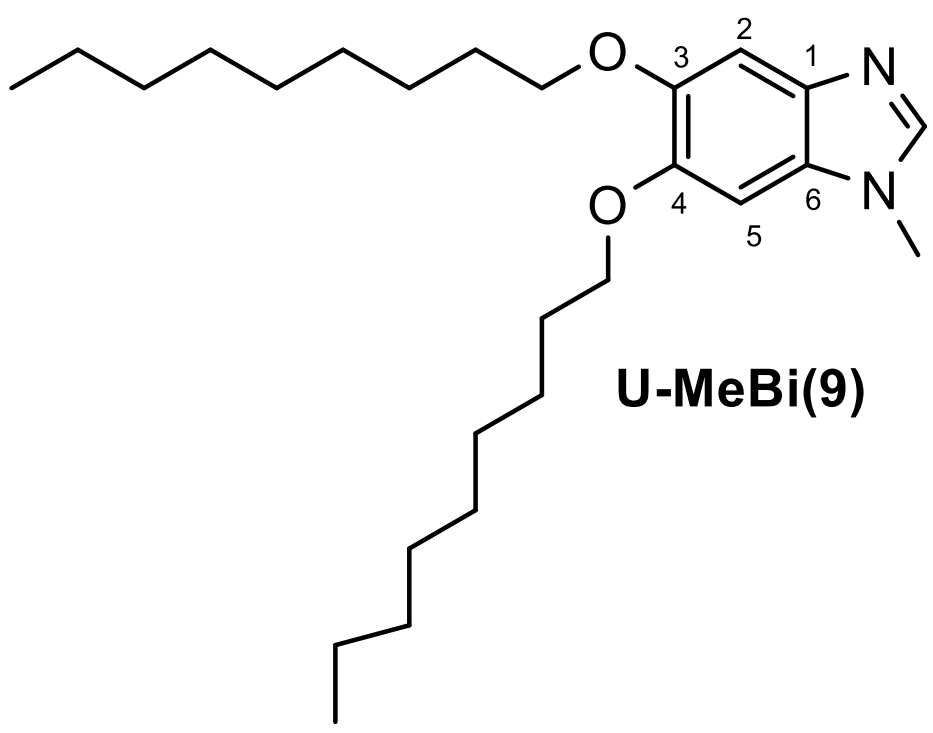

U-MeBi(9)

$C_{26}H_{44}N_2O_2$
(416.34 g mol^{-1})

U-Bi(9) (0.43 g, 1.06 mmol) (40 mL), and NaH (60 %, 0.06 g, 1.40 mmol) was added. MeI (0.12 mL, 1.99 mmol) was added dropwise, and the mixture was stirred at room temperature for 1 h. The reaction mixture was concentrated under reduced pressure, dissolved in diethyl ether, and washed with water (3 ×100 mL). The organic phase was dried over $MgSO_4$, filtered, and concentrated. The residue was purified by column chromatography on silica gel, eluting first with CH_2Cl_2, followed by CH_2Cl_2/MeOH (40:1. Recrystallisation from methanol afforded the **U-MeBi(9)** (0.39 g, 0.94 mmol, 88%); ^{1}H NMR (700 MHz, $CDCl_3$): δ = 0.87-0.89 (m, 6H, 2 x CH_3), 1.25-1.39 (m, 20H, CH_2), 1.47-1.52 (m, 4H, 2 x $O(CH_2)_2C\mathit{H_2}$), 1.82-1.88 (m, 4H, 2 x $OCH_2C\mathit{H_2}$), 3.76 (s, NCH_3), 4.01-4.04 (m, 4H, 2 x OCH_2), 6.83 (s, 1H, 5-H), 7.27 (s, 1H, 2-H), 7.69 (s, 1H, NCHN) ppm; ^{13}C NMR (175 MHz, $CDCl_3$): δ = 14.3 (2 x CH_3), 22.8 (2 x $CH_3\mathit{C}H_2$), 26.24, 26.25 (2 x $O(CH_2)_2\mathit{C}H_2$), 29.44, 29.47, 29.54, 29.61, 29.62, 29.7 (2 x $OCH_2\mathit{C}H_2$, CH_2), 31.2 (NCH_3), 32.0 (CH_2), 69.9, 70.3 (2 x OCH_2), 94.9 (C-5), 104.8 (C-2), 128.7 (Ar C, C-6 or C-1), 137.7 (Ar C, C-1 or C-6), 142.2 (NCHN), 147.0 (Ar C, C-4 or C-3), 147.7 (Ar C, C-3 or C-4) ppm; MS (ESI): m/z (%) = 417.35 [M+H$^+$]; HRMS (ESI): calcd. for $C_{26}H_{44}N_2O_2+H^+$: 417.3476, found: 417.3461; FT-IR (ATR): $\tilde{\nu}$ /cm^{-1} = 3088 (w), 2954 (s), 2918 (vs), 2848 (vs), 2217 (w), 1505 (s), 1491 (s), 1465 (vs), 1422 (m), 1401 (w), 1374 (m), 1346 (m), 1274 (vs), 1236 (vs), 1203 (vs), 1151 (s), 1090 (vs), 1011 (m), 970 (m), 929 (m), 915 (m), 887 (m), 831 (s) cm^{-1}; DSC: heating up (10 K/min) Cr_1 52 °C [-4.3 kJ mol^{-1}] Cr_2 72 °C [-40.1 kJ mol^{-1}] I; cooling down (10 K/min) Cr_1 39 °C [5.0 kJ mol^{-1}] Cr_2 63 °C [42.0 kJ mol^{-1}] I; CHN-analysis: calcd. (%) for $C_{26}H_{44}N_2O_2$: C 74.95, H 10.64, N 6.72, found: C 75.07, H 10.66, N 6.68.

5,6-Bis(decyloxy)-1-methyl-benzoimidazole (U-MeBi(10)):

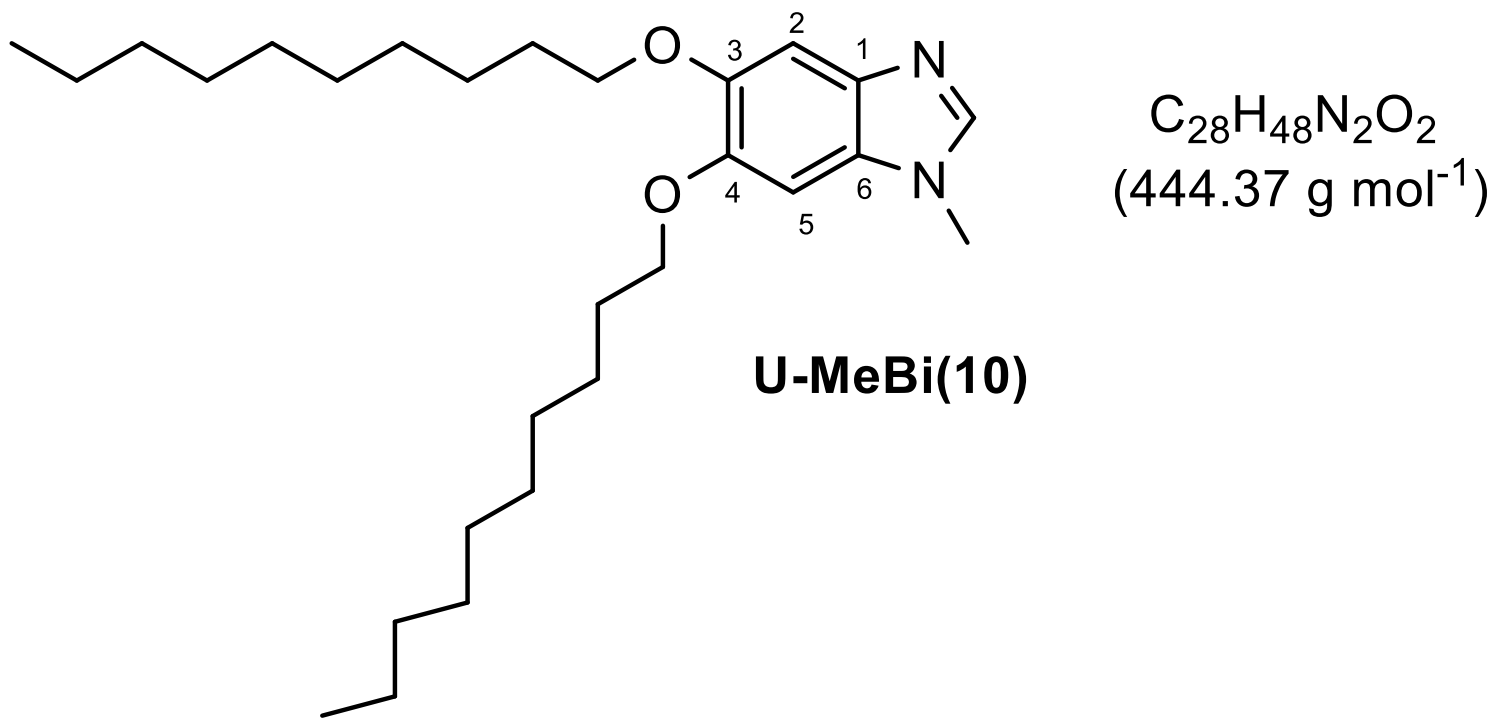

U-MeBi(10)

According to **GP5**, **U-Bi(10)** (1.50 g, 3.48 mmo), 20 mL THF; NaH (60 %, 0.14 g, 3.66 mmol); MeI (0.22 ml, 3.49 mmol), 1.5 h at room temperature; the mixture was applied directly to a silica gel column and eluted successively with petroleum ether/ethyl acetate (5 : 1), and CH_2Cl_2/MeOH (40 : 1); recrystallization from MeOH yielded pure **U-MeBi(10)** (0.91 g, 2.05 mmol, 59 %); ^{1}H NMR (700 MHz, $CDCl_3$): δ = 0.88 (t, *J* = 7.0 Hz, 6H, 2 x CH_3), 1.27-1.39 (m, 24H, CH_2), 1.47-1.52 (m, 4H, 2 x $O(CH_2)_2C\mathit{H}_2$), 1.82-1.89 (m, 4H, 2 x $OCH_2C\mathit{H}_2$), 3.76 (s, NCH_3), 4.01-4.04 (m, 4H, 2 x OCH_2), 6.83 (s, 1H, 5-H), 7.27 (s, 1H, 2-H), 7.70 (s, 1H, NCHN) ppm; ^{13}C NMR (175 MHz, $CDCl_3$): δ = 14.2 (2 x CH_3), 22.8 (2 x $CH_3\mathit{C}H_2$), 26.24, 26.25 (2 x $O(CH_2)_2\mathit{C}H_2$), 29.46, 29.49, 29.53, 29.60, 29.61, 29.7, 29.8 (2 x $OCH_2\mathit{C}H_2$), CH_2), 31.2 (NCH_3), 32.0 (CH_2), 69.9, 70.3 (2 x OCH_2), 94.9 (C-5), 104.8 (C-2), 128.7 (Ar C, C-6 or C-1), 137.6 (Ar C, C-1 or C-6), 142.1 (NCHN), 147.0 (Ar C, C-4 or C-3), 147.7 (Ar C, C-3 or C-4) ppm; MS (ESI): m/z (%) = 445.38 [$M+H^+$]; HRMS (ESI): calcd. for $C_{28}H_{48}N_2O_2+H^+$: 445.3789, found: 445.3782; FT-IR (ATR): $\tilde{\nu}$ /cm^{-1} = 2955 (m), 2916 (vs), 2871 (m), 2848 (vs), 1507 (s), 1491 (s), 1467 (s), 1422 (m), 1400 (w), 1374 (m), 1346 (m), 1274 (s), 1252 (s), 1236 (vs), 1203 (vs), 1153 (s), 1090 (vs), 1048 (w), 1019 (m), 984 (m), 939 (m), 906 (m), 864 (w), 833 (s), 795 (m), 724 (s) cm^{-1}; DSC: heating up (10 K/min) Cr_1 41 °C [-7.9 kJ mol^{-1}] Cr_2 77 °C [-49.1 kJ mol^{-1}] I; cooling down (10 K/min) Cr_1 36 °C [6.7 kJ mol^{-1}] Cr_2 59 °C [21.6 kJ mol^{-1}] I; CHN-analysis: calcd. (%) for $C_{28}H_{48}N_2O_2$: C 75.63, H 10.88, N 6.30, found: C 75.86, H 11.08, N 6.32.

5,6-Bis(dodecyloxy)-1-methyl-benzoimidazole (U-MeBi(12)):

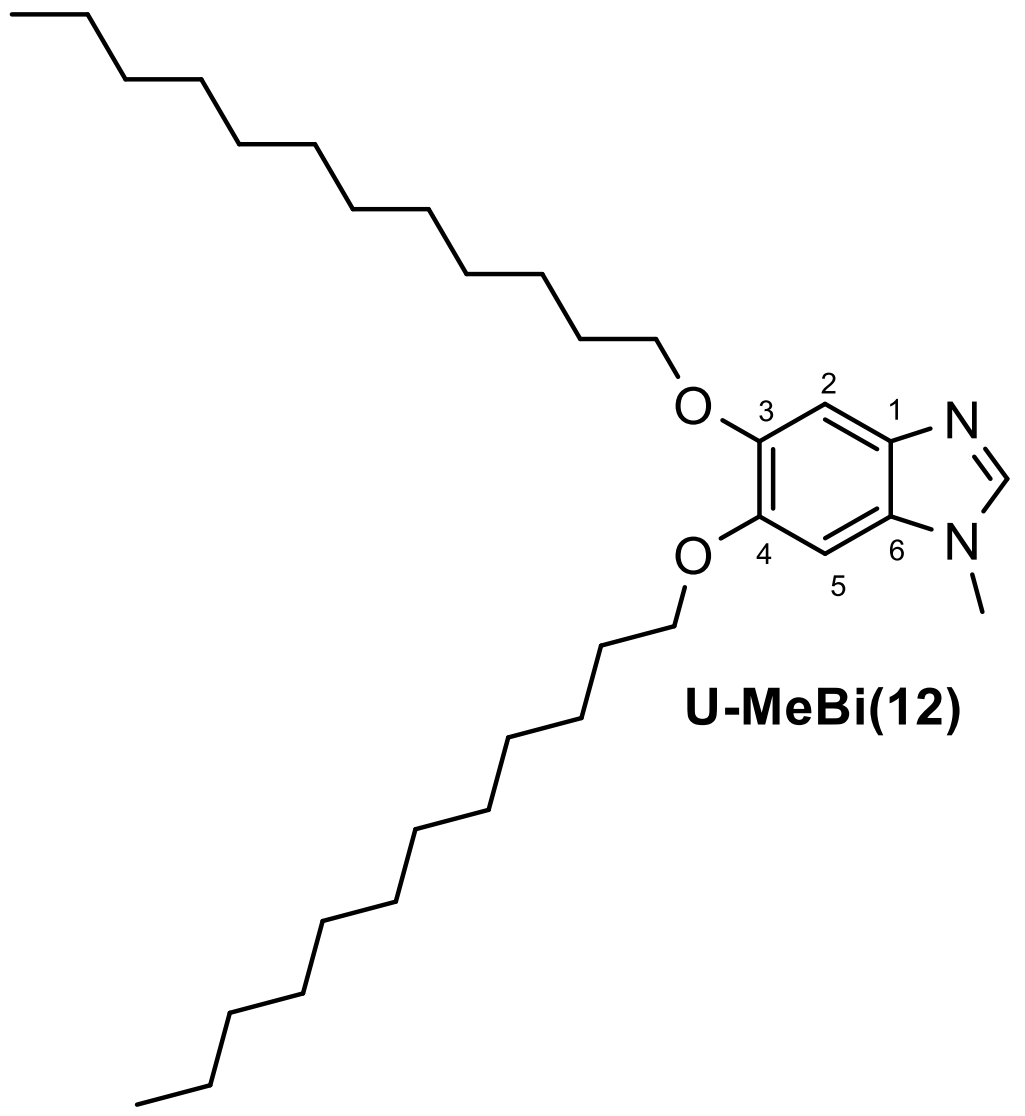

U-MeBi(12)

$C_{32}H_{56}N_2O_2$
(500.43 g mol^{-1})

According to **GP5**, **U-Bi(12)** (0.39 g, 0.81 mmol), 10 mL THF; NaH (60 %, 0.05 g, 1.13 mmol); MeI (0.07 ml, 1.13 mmol), 1.5 h at room temperature; the mixture was applied directly to a silica gel column and eluted successively with petroleum ether/ethyl acetate (5 : 1), and CH_2Cl_2/MeOH (40 : 1); recrystallization from MeOH yielded pure **U-MeBi(12)** (0.29 g, 0.58 mmol, 72 %); ^{1}H NMR (700 MHz, $CDCl_3$): δ = 0.87 (t, *J* = 7.0 Hz, 6H, 2 x CH_3), 1.26-1.39 (m, 32H, CH_2), 1.47-1.52 (m, 4H, 2 x $O(CH_2)_2C\mathit{H_2}$), 1.82-1.88 (m, 4H, 2 x $OCH_2C\mathit{H_2}$), 3.77 (s, NCH_3), 4.01-4.04 (m, 4H, 2 x OCH_2), 6.83 (s, 1H, 5-H), 7.27 (s, 1H, 2-H), 7.70 (s, 1H, NCHN) ppm; ^{13}C NMR (175 MHz, $CDCl_3$): δ = 14.3 (2 x CH_3), 22.8 (2 x $CH_3\mathit{C}H_2$), 26.25, 26.26 (2 x $O(CH_2)_2\mathit{C}H_2$), 29.47, 29.51, 29.54, 29.62, 29.79, 29.80, 29.83, 29.84 (2 x $OCH_2\mathit{C}H_2$), CH_2), 31.2 (NCH_3), 32.1 (CH_2), 69.9, 70.3 (2 x OCH_2), 94.9 (C-5), 104.8 (C-2), 128.7 (Ar C, C-6 or C-1), 137.6 (Ar C, C-1 or C-6), 142.1 (NCHN), 147.0 (Ar C, C-4 or C-3), 147.8 (Ar C, C-3 or C-4) ppm; MS (ESI): m/z (%) = 501.44 [M+H$^+$]; HRMS (ESI): calcd. for $C_{32}H_{56}N_2O_2$+H$^+$: 501.4415, found: 501.4403; FT-IR (ATR): $\tilde{\nu}$ /cm^{-1} = 2954 (m), 2914 (vs), 2872 (m), 2847 (vs), 1505 (m), 1495 (s), 1467 (s), 1422 (w), 1375 (w), 1347 (m), 1275 (s), 1232 (m), 1207 (s), 1151 (m), 1092 (s), 994 (w), 955 (w), 924 (w), 888 (w), 831 (m), 802 (w), 727 (s) cm^{-1}; DSC: heating up (10 K/min) Cr_1 42 °C [-8.2 kJ mol^{-1}] Cr_2 83 °C [-54.7 kJ mol^{-1}] I; cooling down (10 K/min) Cr_1 36 °C [7.7 kJ mol^{-1}] Cr_2 61 °C [53.6 kJ mol^{-1}] I; CHN-analysis: calcd. (%) for $C_{32}H_{56}N_2O_2$: C 76.75, H 11.27, N 5.59, found: C 77.05, H 11.50, N 5.58.

5,6-Bis(tetradecyloxy)-1-methyl-benzoimidazole (U-MeBi(14)):

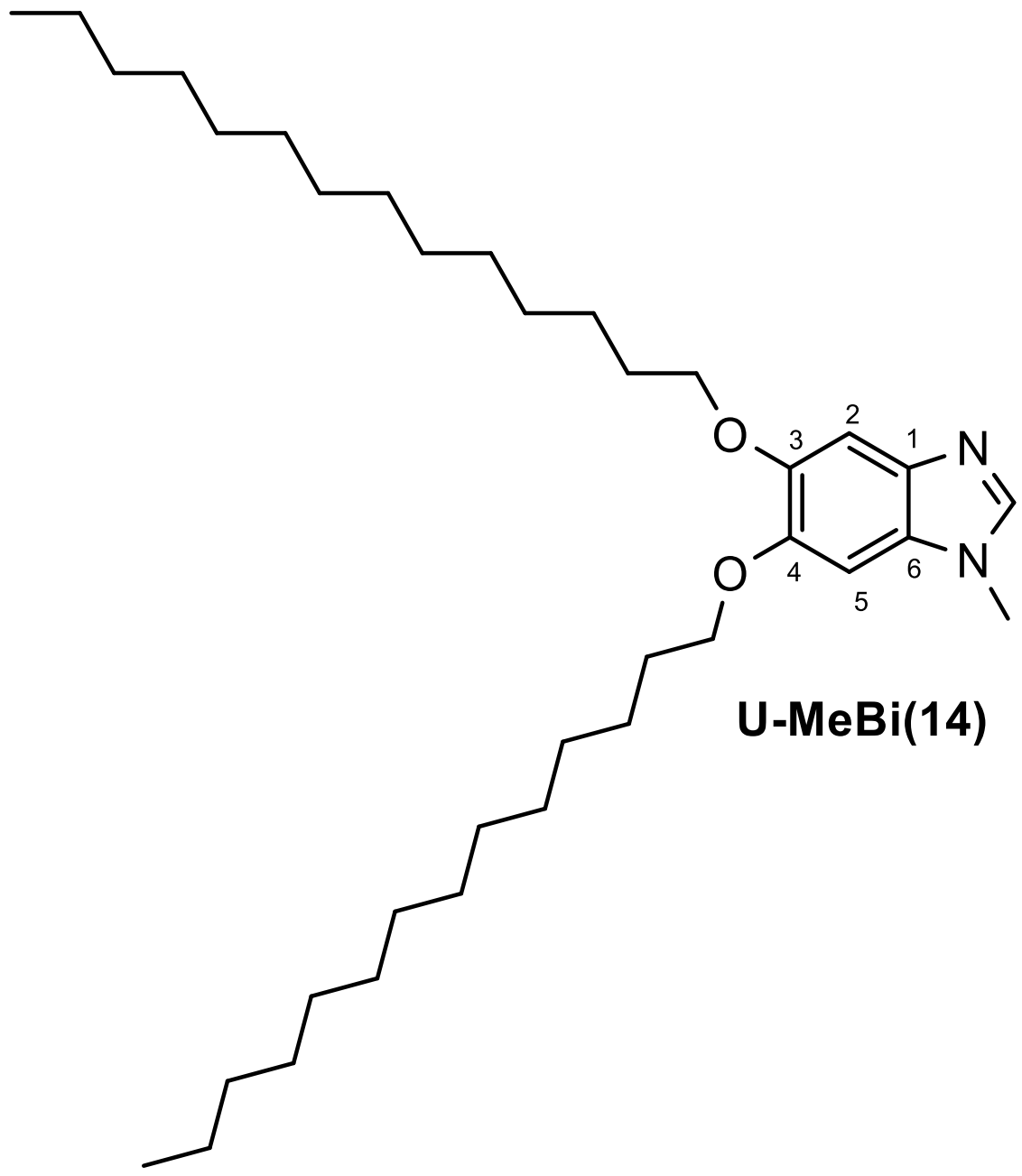

U-MeBi(14)

$C_{36}H_{64}N_2O_2$
(556.50 g mol^{-1})

According to **GP5**, **U-Bi(14)** (0.61 g, 1.12 mmol), 10 mL THF; NaH (60 %, 0.05 g, 1.13 mmol); MeI (0.07 ml, 1.75 mmol), 1.5 h at room temperature; the mixture was applied directly to a silica gel column and eluted successively with petroleum ether/ethyl acetate (5 : 1), and CH_2Cl_2/MeOH (40 : 1); recrystallization from MeOH yielded pure **U-MeBi(14)** (0.47 g, 0.85 mmol, 76 %); ^{1}H NMR (700 MHz, $CDCl_3$): δ = 0.87 (t, J = 7.0 Hz, 6H, 2 x CH_3), 1.25-1.39 (m, 40H, CH_2), 1.47-1.52 (m, 4H, 2 x $O(CH_2)_2C\mathit{H_2}$), 1.82-1.88 (m, 4H, 2 x $OCH_2C\mathit{H_2}$), 3.76 (s, NCH_3), 4.01-4.04 (m, 4H, 2 x OCH_2), 6.83 (s, 1H, 5-H), 7.27 (s, 1H, 2-H), 7.70 (s, 1H, NCHN) ppm; ^{13}C NMR (175 MHz, $CDCl_3$): δ = 14.3 (2 x CH_3), 22.8 (2 x $CH_3\mathit{C}H_2$), 26.25, 26.26 (2 x $O(CH_2)_2\mathit{C}H_2$),.47, 29.51, 29.54, 29.61, 29.62, 29.80, 29.85 (CH_2), 31.2 (NCH_3), 32.1 (CH_2), 69.9, 70.3 (2 x OCH_2), 94.9 (C-5), 104.8 (C-2), 128.7 (Ar C, C-6 or C-1), 137.6 (Ar C, C-1 or C-6), 142.1 (NCHN), 147.0 (Ar C, C-4 or C-3), 147.7 (Ar C, C-3 or C-4) ppm; MS (ESI): m/z (%) = 557.50 [M+H$^+$]; HRMS (ESI): calcd. for $C_{36}H_{64}N_2O_2$+H$^+$: 557.5041, found: 557.5031; FT-IR (ATR): $\tilde{\nu}$ /cm^{-1} = 2955 (w), 2914 (vs), 2874 (m), 2847 (vs), 1495 (s), 1467 (s), 1421 (w), 1400 (w), 1376 (w), 1348 (m), 1330 (w), 1273 (s), 1230 (m), 1207 (s), 1149 (m), 1092 (m), 1050 (w), 1005 (w), 971 (w), 933 (w), 915 (m), 879 (w), 829 (m) cm^{-1}; DSC: heating up (10 K/min) Cr_1 19 °C [-5.2 kJ mol^{-1}] Cr_2 86 °C [-65.1 kJ mol^{-1}] I; cooling down (10 K/min) Cr_1 18 °C [6.0 kJ mol^{-1}] Cr_2 75 °C [62.8 kJ mol^{-1}] I; CHN-analysis: calcd. (%) for $C_{36}H_{64}N_2O_2$: C 77.84, H 11.58, N 5.75, found: C 77.84, H 11.84, N 5.04.

2.6. Syntheses of 4,7-bis(alkoxy)-1-methyl-benzoimidazole (L-MeBi(n))

4,7-Bis(pentyloxy)-1-methyl-benzoimidazole (L-MeBi(5)):

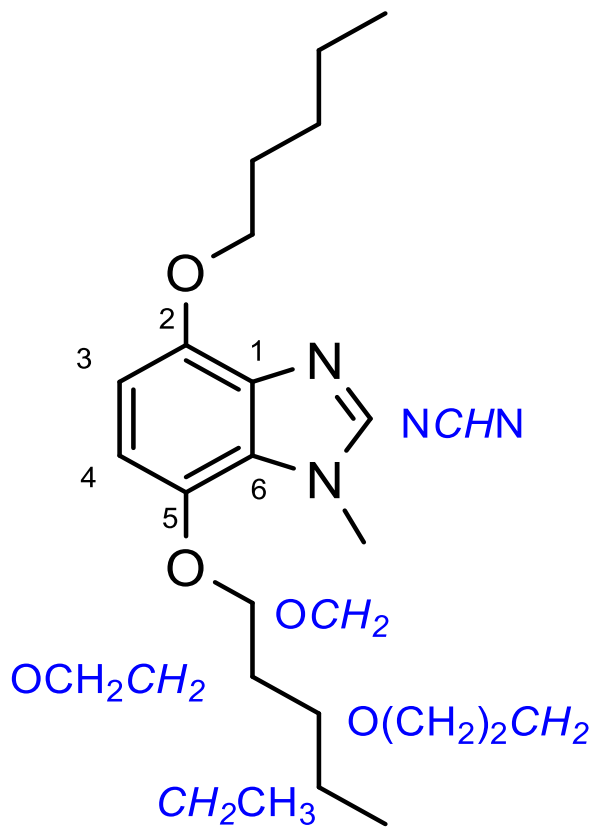

$C_{18}H_{28}N_2O_2$
(304.22 g mol^{-1})

L-MeBi(5)

According to **GP5**, **L-Bi(5)** (0.50 g, 1.7 mmol), 10 mL THF; NaH (60 %, 0.08 g, 2.1 mmol); MeI (0.12 ml, 1.9 mmol), 1 h at room temperature; the mixture was applied directly to a silica gel column and eluted successively with petroleum ether/ethyl acetate (40 : 1), followed by CH_2Cl_2/MeOH (40 : 1); **L-MeBi(5)** (0.50 g, 1.3 mmol, 77 %). ^{1}H NMR (700 MHz, $CDCl_3$): δ = 0.91-0.95 (m, 6H, 2 x CH_3), 1.35-1.43 (m, 4H, C*H*$_2$$CH_3$), 1.45-1.51 (m, 4H, 2 x $O(CH_2)_2$C*H*$_2$), 1.82-1.91 (m, 4H, 2´-H, 2´´-H [2 x OCH_2C*H*$_2$]), 4.02 (t, *J* = 6.5 Hz, 2H, 1´-H [OCH_2]), 4.03 (s, 3H, NCH_3), 4.12 (t, *J* = 6.7 Hz, 2H, 1´´-H [OCH_2]), 6.50-6.53 (m, 2H, 3-H, 4-H), 7.66 (s, 1H, NCHN) ppm; ^{13}C NMR (175 MHz, $CDCl_3$): δ = 14.18, 14.19 (2 x CH_3), 22.6, 22.7 (*C*H_2CH_3), 28.4, 28.5 (C-3´, C-3´´ [$O(CH_2)_2$*C*H_2]), 29.17, 29.24 (C-2´, C-2´´ [2 x OCH_2*C*H_2]), 33.9 (NCH_3), 68.7 (C-1´´[OCH_2]), 69.2 (C-1´, [OCH_2]), 103.4 (Ar C, C-4 or C-3), 104.0 (Ar C, C-3 or C-4), 125.9 (Ar C, C-6 or C-1), 136.2 (Ar C, C-1 or C-6), 141.5 (Ar C, C-5 or C-2), 142.8 (NCHN), 145.6 (Ar C, C-2 or C-5) ppm; MS (ESI): m/z (%) = 305.22 [M+H$^+$]; HRMS (ESI): calcd. for $C_{18}H_{28}N_2O_2$+H$^+$: 305.2224, found: 305.2218; FT-IR (ATR): $\tilde{\nu}$ /cm^{-1} = 2954 (m), 2930 (s), 2869 (m), 1520 (vs), 1501 (s), 1469 (s), 1433 (m), 1379 (w), 1338 (m), 1262 (vs), 1206 (w), 1155 (m), 1124 (w), 1090 (s), 1056 (s), 982 (w), 783 (m), 722 (m), 641 (m) cm^{-1}; DSC: heating up (10 K/min) Cr 44 °C [-24.0 kJ mol^{-1}] I; cooling down (10 K/min) Cr 22 °C [16.2 kJ mol^{-1}] I; CHN-analysis: calcd. (%) for $C_{18}H_{28}N_2O_2$: C 71.02, H 9.27, N 9.20, calcd. (%) for $C_{19}H_{30}N_2O_2$ + 0.1 eq. H_2O: C 70.60, H 9.28, N 9.15, found: C 70.53, H 9.17, N 9.01.

4,7-Bis(hexyloxy)-1-methyl-benzoimidazole (L-MeBi(6)):

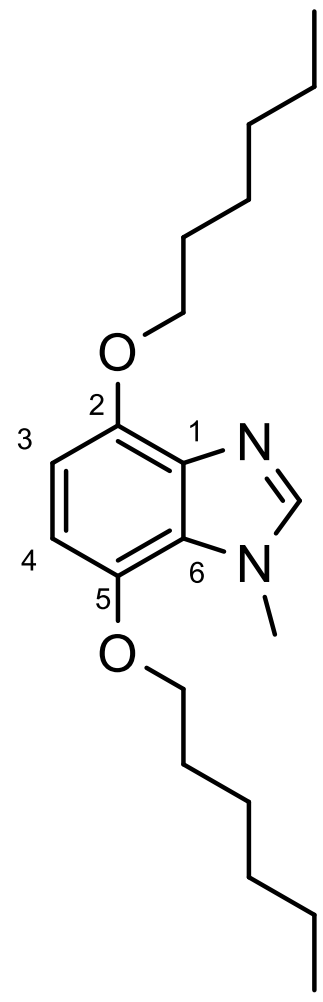

$C_{20}H_{32}N_2O_2$
(332.25 g mol^{-1})

L-MeBi(6)

According to **GP5**, **L-Bi(6)** (0.24 g, 0.7 mmol), 10 mL THF; NaH (60 %, 0.02 g, 0.7 mmol); MeI (0.03 ml, 0.5 mmol), 1 h at room temperature; the mixture was applied directly to a silica gel column and eluted successively with CH_2Cl_2/MeOH (40 : 1); **L-MeBi(6)** (0.23 g, 0.7 mmol, 91 %); ^{1}H NMR (700 MHz, $CDCl_3$): δ = 0.88-0.92 (m, 6H, 2 x CH_3), 1.30-1.40 (m, 8H, CH_2), 1.47-1.52 (m, 4H, 2 x $O(CH_2)_2CH_2$), 1.80-1.90 (m, 4H, 2´-H, 2´´-H [2 x OCH_2CH_2]), 4.01 (t, *J* = 6.4 Hz, 2H, 1´-H [OCH_2]), 4.03 (s, 3H, NCH_3), 4.12 (t, *J* = 6.8 Hz, 2H, 1´´-H [OCH_2]), 6.50-6.53 (m, 2H, 3-H, 4-H), 7.65 (s, 1H, NCHN) ppm; ^{13}C NMR (175 MHz, $CDCl_3$): δ = 14.15, 14.22 (2 x CH_3), 22.73, 22.75 (CH_2), 25.9, 26.0 (C-3´, C-3´´ [$O(CH_2)_2CH_2$]), 29.4, 29.5 (C-2´, C-2´´ [2 x OCH_2CH_2]), 31.7, 31.9 (CH_2), 33.9 (NCH_3), 68.7 (C-1´´[OCH_2]), 69.2 (C-1´, [OCH_2]), 103.4 (Ar C, C-4 o. C-3), 104.0 (Ar C, C-3 o. C-4), 125.8 (Ar C, C-6 or C-1), 136.3 (Ar C, C-1 or C-6), 141.5 (Ar C, C-5 or C-2), 142.8 (NCHN), 145.5 (Ar C, C-2 or C-5) ppm; MS (ESI): m/z (%) = 333.25 [$M+H^+$]; HRMS (ESI): calcd. for $C_{20}H_{32}N_2O_2+H^+$: 333.2537, found: 333.2530; FT-IR (ATR): $\tilde{\nu}$ /cm^{-1} = 2954 (s), 2929 (s), 2859 (m), 1521 (vs), 1502 (s), 1469 (m), 1434 (w), 1338 (m), 1263 (vs), 1155 (m), 1092 (s), 1054 (m), 779 (w), 723 (w), 642 (w) cm^{-1}; DSC: heating up (10 K/min) Cr 53 °C [-26.5 kJ mol^{-1}] I; cooling down (10 K/min) Cr 7 °C [13.4 kJ mol^{-1}] I; CHN-analysis: calcd. (%) for $C_{20}H_{32}N_2O_2$: C 72.25, H 9.70, N 8.43, calcd. (%) for $C_{20}H_{32}N_2O_2$ + 0.2 eq. H_2O: C 71.47, H 9.72, N 8.34, found: C 71.48, H 9.60, N 8.27.

4,7-Bis(heptyloxy)-1-methyl-benzoimidazole (L-MeBi(7)):

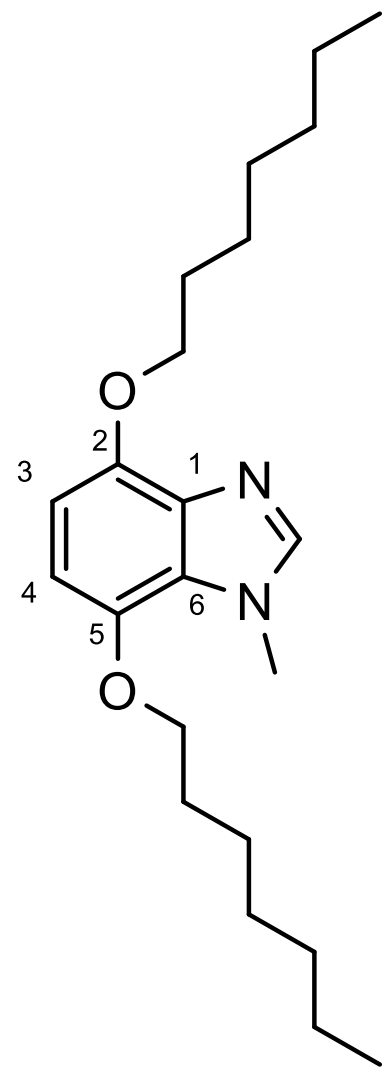

$C_{22}H_{36}N_2O_2$
(360.28 g mol^{-1})

L-MeBi(7)

According to **GP5**, **L-Bi(7)** (0.54 g, 1.6 mmol), 20 mL THF; NaH (60 %, 0.08 g, 1.9 mmol); MeI (0.11 ml, 1.7 mmol), 1 h at room temperature; the mixture was applied directly to a silica gel column and eluted successively with petroleum ether/ethyl acetate (40 : 1), followed by CH_2Cl_2/MeOH (40 : 1); **L-MeBi(7)** (0.50 g, 1.4 mmol, 88 %); ^{1}H NMR (500 MHz, $CDCl_3$): δ = 0.86-0.91 (m, 6H, 2 x CH_3), 1.30-1.40 (m, 12H, CH_2), 1.45-1.52 (m, 4H, 2 x $O(CH_2)_2C\mathit{H_2}$), 1.80-1.94 (m, 4H, 2´-H, 2´´-H [2 x $OCH_2C\mathit{H_2}$]), 4.01-4.03 (m, 2H, 1´-H [OCH_2], 3H, NCH_3), 4.13 (t, *J* = 6.7 Hz, 2H, 1´´-H [OCH_2]), 6.49-6.53 (m, 2H, 3-H, 4-H), 7.64 (s, 1H, NCHN) ppm; ^{13}C NMR (125 MHz, $CDCl_3$): δ = 14.21, 14.23 (2 x CH_3), 22.73, 22.77 (CH_2), 26.2, 26.3 (C-3´, C-3´´ [$O(CH_2)_2C\mathit{H_2}$]), 29.2, 29.3, 29.5, 29.6 (C-2´, C-2´´ [2 x $OCH_2C\mathit{H_2}$], CH_2), 31.92, 31.94 (CH_2), 33.9 (NCH_3), 68.7 (C-1´´[OCH_2]), 69.2 (C-1´, [OCH_2]), 103.4 (Ar C, C-4 o. C-3), 104.0 (Ar C, C-3 o. C-4), 125.9 (Ar C, C-6 or C-1), 136.4 (Ar C, C-1 or C-6), 141.5 (Ar C, C-5 or C-2), 142.8 (NCHN), 145.6 (Ar C, C-2 or C-5) ppm; MS (ESI): m/z (%) = 361.28 [M+H$^+$]; HRMS (ESI): calcd. for $C_{22}H_{36}N_2O_2$+H$^+$: 361.2850, found: 361.2842; FT-IR (ATR): $\tilde{\nu}$ /cm^{-1} = 2922 (m), 2851 (w), 1669 (vs), 1527 (m), 1464 (m), 1433 (w), 1396 (w), 1338 (s), 1269 (w), 1244 (w), 1222 (m), 1117 (w), 1065 (w), 998 (m), 950 (w), 797 (w) cm^{-1}; DSC: heating up (10 K/min) Cr 47 °C [-23.2 kJ mol^{-1}] I; cooling down (10 K/min) Cr -7 °C [9.1 kJ mol^{-1}] I; CHN-analysis: calcd. (%) for $C_{22}H_{36}N_2O_2$: C 73.29, H 10.06, N 7.77, found: C 73.20, H 10.03, N 7.72.

4,7-Bis(octyloxy)-1-methyl-benzoimidazole (L-MeBi(8)):

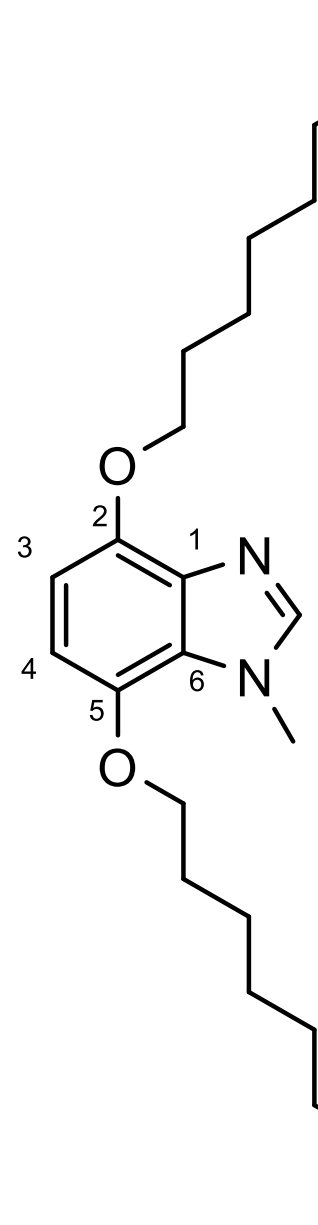

$C_{24}H_{40}N_2O_2$
(388.31 g mol^{-1})

L-MeBi(8)

According to **GP5**, **L-Bi(8)** (0.55 g, 1.5 mmol), 20 mL MeCN and 10 mL THF; NaH (60 %, 0.07 g, 1.80 mmol); MeI (0.10 ml, 1.60 mmol), 1 h at room temperature; the mixture was applied directly to a silica gel column and eluted successively with petroleum ether/ethyl acetate (40 : 1), followed by column chromatography with CH_2Cl_2/MeOH (40 : 1 → 10 : 1); **L-MeBi(8)** (0.56 g, 1.4 mmol, 97 %). ^{1}H NMR (700 MHz, $CDCl_3$): δ = 0.86-0.90 (m, 6H, 2 x CH_3), 1.25-1.39 (m, 16H, CH_2), 1.46-1.52 (m, 4H, 2 x $O(CH_2)_2C\mathit{H_2}$), 1.81-1.91 (m, 4H, 2´-H, 2´´-H [2 x $OCH_2C\mathit{H_2}$]), 4.02 (t, *J* = 6.4 Hz, 2H, 1´-H [OCH_2]), 4.03 (s, 3H, NCH_3), 4.13 (t, *J* = 6.8 Hz, 2H, 1´´-H [OCH_2]), 6.49-6.53 (m, 2H, 3-H, 4-H), 7.64 (s, 1H, NCHN) ppm; ^{13}C NMR (175 MHz, $CDCl_3$): δ = 14.3, (2 x CH_3), 22.79, 22.80 (CH_2), 26.27, 26.35 (C-3´, C-3´´ [$O(CH_2)_2C\mathit{H_2}$]), 29.39, 29.46, 29.48, 29.55 (C-2´, C-2´´ [2 x $OCH_2\mathit{CH_2}$]), 29.62 (CH_2), 31.94, 31.99 (CH_2), 33.9 (NCH_3), 68.7 (C-1´´[OCH_2]), 69.2 (C-1´, [OCH_2]), 103.4 (Ar C, C-4 o. C-3), 104.0 (Ar C, C-3 o. C-4), 125.9 (Ar C, C-6 or C-1), 136.4 (Ar C, C-1 or C-6), 141.5 (Ar C, C-5 or C-2), 142.8 (NCHN), 145.6 (Ar C, C-2 or C-5) ppm; MS (ESI): m/z (%) = 389.32 [$M+H^+$]; HRMS (ESI): calcd. for $C_{24}H_{40}N_2O_2+H^+$: 389.3163, found: 389.3165; FT-IR (ATR): $\tilde{\nu}$ /cm^{-1} = 2922 (s), 2852 (s), 1519 (vs), 1500 (s), 1468 (s), 1434 (w), 1391 (w), 1334 (m), 1279 (s), 1260 (vs), 1207 (w), 1152 (m), 1091 (s), 1055 (s), 908 (w), 776 (m), 724 (s), 679 (w), 641 (s) cm^{-1}; DSC: heating up (10 K/min) Cr 57 °C [-29.0 kJ mol^{-1}] I; cooling down (10 K/min) Cr_1 28 °C [13.2 kJ mol^{-1}] Cr_2 32 °C [13.6 kJ mol^{-1}] I; CHN-analysis: calcd. (%) for $C_{24}H_{40}N_2O_2$: C 72.18, H 10.38, N 7.21, calcd. (%) for $C_{24}H_{40}N_2O_2$ + 0.4 eq. H_2O: C 72.83, H 10.39, N 7.08, found: C 72.89, H 10.26, N 6.97.

4,7-Bis(nonyloxy)-1-methyl-benzoimidazole (L-MeBi(9)):

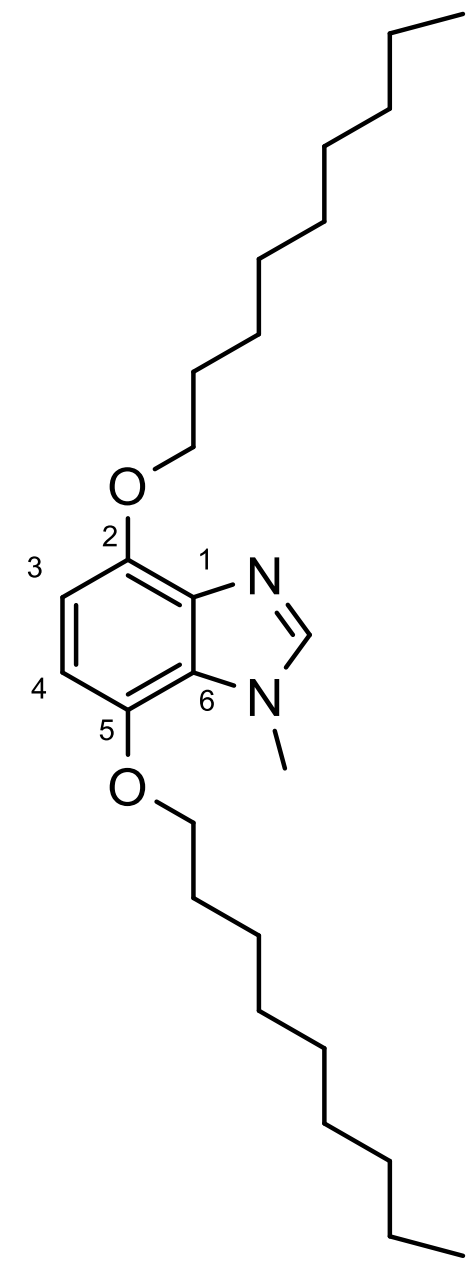

$C_{26}H_{44}N_2O_2$
(416.34 g mol^{-1})

L-MeBi(9)

According to **GP5**, **L-Bi(9)** (1.1 g, 2.7 mmol), 30 mL THF; NaH (60 %, .13 g, 3.3 mmol); MeI (0.19 ml, 3.0 mmol), 1 h at room temperature; the mixture was applied directly to a silica gel column and eluted successively with petroleum ether/ethyl acetate (40 : 1), followed by column chromatography with CH_2Cl_2/MeOH (40 : 1 → 10 : 1); **L-MeBi(9)** (1.1 g, 2.5 mmol, 93 %); ^{1}H NMR (700 MHz, $CDCl_3$): δ = 0.86-0.89 (m, 6H, 2 x CH_3), 1.24-1.39 (m, 20H, CH_2), 1.46-1.51 (m, 4H, 2 x $O(CH_2)_2C\mathit{H_2}$), 1.81-1.90 (m, 4H, 2´-H, 2´´-H [2 x $OCH_2C\mathit{H_2}$]), 4.02 (t, J = 6.4 Hz, 2H, 1´-H [OCH_2]), 4.03 (s, 3H, NCH_3), 4.13 (t, J = 6.8 Hz, 2H, 1´´-H [OCH_2]), 6.49-6.53 (m, 2H, 3-H, 4-H), 7.64 (s, 1H, NCHN) ppm; ^{13}C NMR (175 MHz, $CDCl_3$): δ = 14.25, 14.26 (2 x CH_3), 22.8 (CH_2), 26.3, 26.4 (C-3´, C-3´´ [$O(CH_2)_2\mathit{C}H_2$]), 29.39, 29.44, 29.48, 29.50, 29.55, 29.67, 29.69, 29.69 (2´, 2´´ [2 x $OCH_2\mathit{C}H_2$], CH_2), 32.00, 32.03 (CH_2), 33.9 (NCH_3), 68.7 (C-1´´[OCH_2]), 69.2 (C-1´, [OCH_2]), 103.4 (Ar C, C-4 o. C-3), 104.0 (Ar C, C-3 o. C-4), 125.9 (Ar C, C-6 or C-1), 136.4 (Ar C, C-1 or C-6), 141.5 (Ar C, C-5 or C-2), 142.8 (NCHN), 145.6 (Ar C, C-2 or C-5) ppm; MS (ESI): m/z (%) = 417.35 [$M+H^+$]; HRMS (ESI): calcd. for $C_{26}H_{44}N_2O_2+H^+$: 417.3476, found: 417.3471; FT-IR (ATR): $\tilde{\nu}$ /cm^{-1} = 2924 (vs), 2854 (s), 1521 (vs), 1501 (m), 1468 (m), 1434 (w), 1338 (m), 1277 (s), 1263 (vs), 1154 (m), 1092 (m), 1056 (m), 778 (w), 722 (w), 641 (w) cm^{-1}; DSC: heating up (10 K/min) Cr 37 °C [-37.0 kJ mol^{-1}] I; cooling down (10 K/min) Cr -1 °C [11.4 kJ mol^{-1}] I; CHN-analysis: calcd. (%) for $C_{26}H_{44}N_2O_2$: C 74.95, H 10.64, N 6.72, found: C 75.08, H 10.76, N 6.62.

4,7-Bis(decyloxy)-1-methyl-benzoimidazole (L-MeBi(10)):

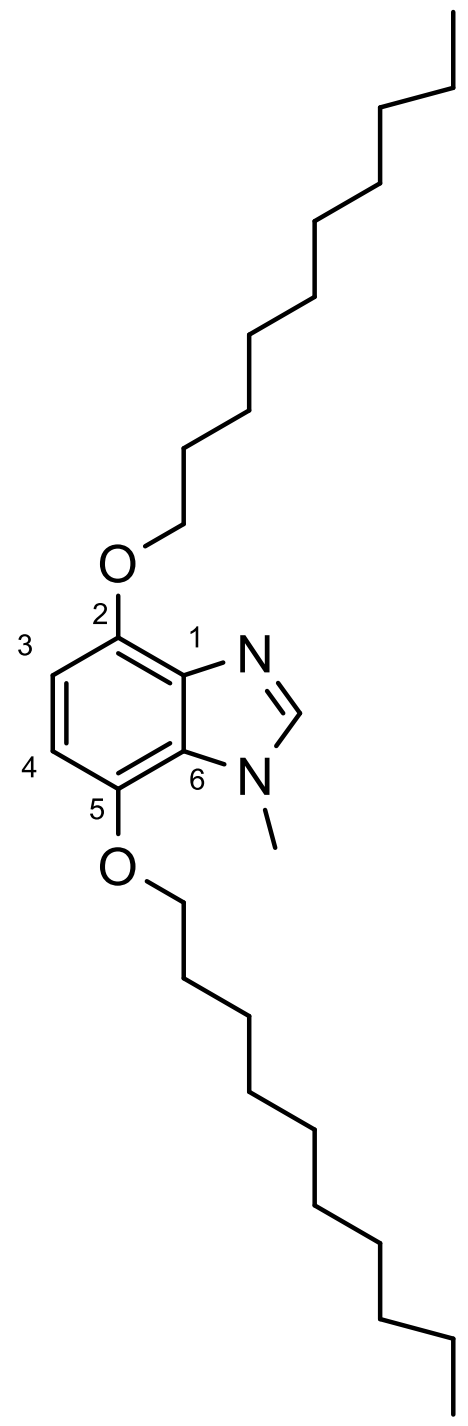

$C_{28}H_{48}N_2O_2$
(444.37 g mol^{-1})

L-MeBi(10)

According to **GP5**, **L-Bi(10)** (0.10 g, 0.24 mmol), 10 mL THF; NaH (60 %, 0.014 g, 0.35 mmol); MeI (0.022 ml, 0.35 mmol), 1 h at room temperature; the mixture was applied directly to a silica gel column and eluted successively with petroleum ether/ethyl acetate (5 : 1), followed by column chromatography with CH_2Cl_2/MeOH (40 : 1); **L-MeBi(10)** (0.062 g, 0.14 mmol, 59 %); ^{1}H NMR (400 MHz, $CDCl_3$): δ = 0.86-0.90 (m, 6H, 2 x CH_3), 1.27-1.36 (m, 24H, CH_2), 1.45-1.53 (m, 4H, 2 x $O(CH_2)_2C\mathit{H_2}$), 1.79-1.92 (m, 4H, 2´-H, 2´´-H [2 x $OCH_2C\mathit{H_2}$]), 4.02 (t, *J* = 6.3 Hz, 2H, 1´-H [OCH_2]), 4.03 (s, 3H, NCH_3), 4.13 (t, *J* = 6.8 Hz, 2H, 1´´-H [OCH_2]), 6.49-6.54 (m, 2H, 3-H, 4-H), 7.65 (s, 1H, NCHN) ppm; ^{13}C NMR (100 MHz, $CDCl_3$): δ = 14.2 (2 x CH_3), (2 x $CH_3\mathit{C}H_2$), 26.3, 26.4 (C-3´, C-3´´ [$O(CH_2)_2\mathit{C}H_2$]), 29.45, 29.46, 29.50, 29.6, 29.65, 29.69, 29.70 (2´, 2´´ [2 x $OCH_2\mathit{C}H_2$], CH_2), 32.0 (CH_2), 33.9 (NCH_3), 68.7 (C-1´´[OCH_2]), 69.3 (C-1´, [OCH_2]), 103.5 (Ar C, C-4 o. C-3), 104.1 (Ar C, C-3 o. C-4), 125.9 (Ar C, C-6 or C-1), 136.3 (Ar C, C-1 or C-6), 141.5 (Ar C, C-5 or C-2), 142.8 (NCHN), 145.6 (Ar C, C-2 or C-5) ppm; MS (ESI): m/z (%) = 445.38 [$M+H^+$]; HRMS (ESI): calcd. for $C_{28}H_{48}N_2O_2+H^+$: 445.3789, found: 445.3781; FT-IR (ATR): $\tilde{\nu}$ /cm^{-1} = 2922 (vs), 2853 (s), 1520 (vs), 1502 (m), 1468 (m), 1434 (w), 1338 (m), 1263 (vs), 1154 (m), 1092 (s), 1056 (m), 779 (w), 722 (m), 641 (w) cm^{-1}; DSC: heating up (10 K/min) Cr 39 °C [-40.3 kJ mol^{-1}] I; cooling down (10 K/min) Cr 14 °C [22.1 kJ mol^{-1}] I; CHN-analysis: calcd. (%) for $C_{28}H_{48}N_2O_2$: C 75.63, H 10.88, N 6.30, found: C 75.92, H 11.13, N 6.42.

4,7-Bis(dodecyloxy)-1-methyl-benzoimidazole (L-MeBi(12)):

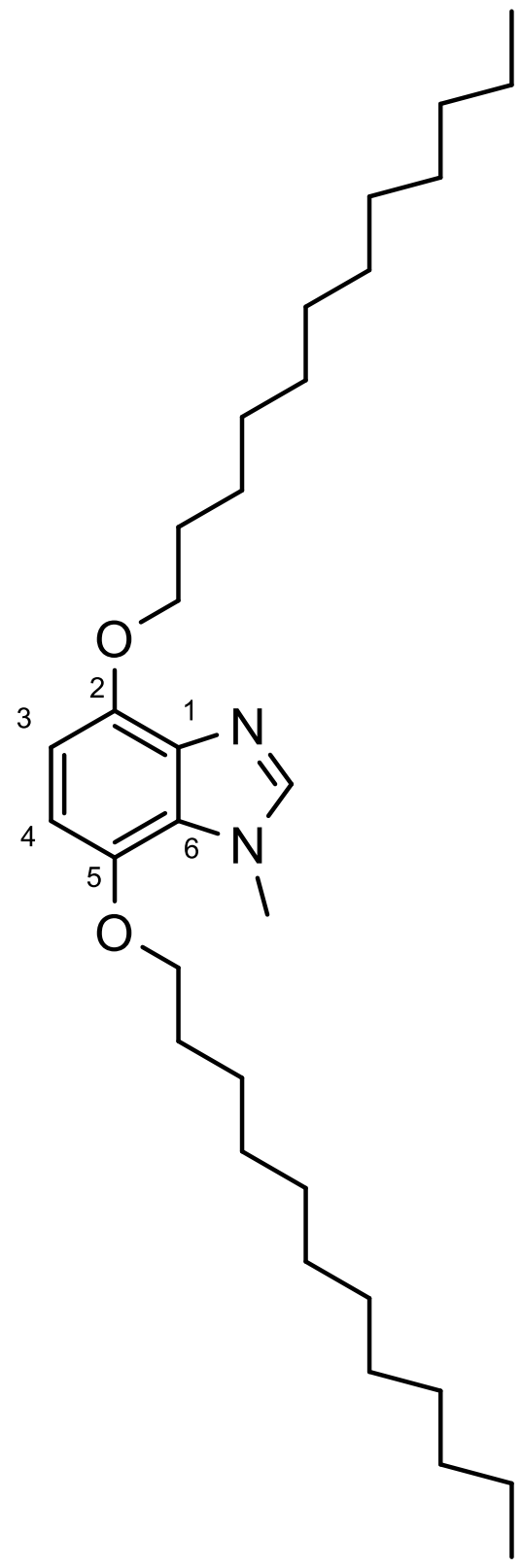

$C_{32}H_{56}N_2O_2$
(500.43 g mol^{-1})

L-MeBi(12)

L-Bi(12) (0.98 g, 2.01 mmol) was dissolved in THF (15 mL), and NaH (60%, 0.097 g, 2.43 mmol) was added. The suspension was stirred at room temperature for 15 min. MeI (0.13 mL, 2.01 mmol) was added dropwise, and the mixture was stirred at the same temperature for 1 h. Silica gel was added directly to the reaction mixture, and the solvent was removed under reduced pressure. The crude product was purified by column chromatography with CH2Cl_2/MeOH (40 : 1) as eluents. The product **L-MeBi(12)** was obtained as a solid (0.91 g, 1.82 mmol, 91%); ^{1}H NMR (500 MHz, $CDCl_3$): δ = 0.86-0.89 (m, 6H, 2 x CH_3), 1.25-1.37 (m, 32H, CH_2), 1.45-1.52 (m, 4H, 2 x $O(CH_2)_2C\mathit{H_2}$), 1.80-1.91 (m, 4H, 2´-H, 2´´-H [2 x $OCH_2C\mathit{H_2}$]), 4.017 (t, *J* = 6.3 Hz, 2H, 1´-H [OCH_2]), 4.024 (s, 3H, NCH_3), 4.13 (t, *J* = 6.8 Hz, 2H, 1´´-H [OCH_2]), 6.49-6.53 (m, 2H, 3-H, 4-H), 7.63 (s, 1H, NCHN) ppm; ^{13}C NMR (125 MHz, $CDCl_3$): δ = 14.3 (2 x CH_3), 22.8 (2 x $CH_3\mathit{C}H_2$), 26.2, 26.3 (C-3´, C-3´´ [$O(CH_2)_2\mathit{C}H_2$]), 29.5, 29.6, 29.65, 29.73, 29.76, 29.79 (2´, 2´´ [2 x $OCH_2\mathit{C}H_2$], CH_2), 32.0 (CH_2), 33.8 (NCH_3), 68.7 (C-1´´[OCH_2]), 69.2 (C-1´, [OCH_2]), 103.4 (Ar C, C-4 o. C-3), 104.0 (Ar C, C-3 o. C-4), 125.9 (Ar C, C-6 or C-1), 136.4 (Ar C, C-1 or C-6), 141.5 (Ar C, C-5 or C-2), 142.8 (NCHN), 145.6 (Ar C, C-2 or C-5) ppm; MS (ESI): m/z (%) = 501.44 [M+H$^+$]; HRMS (ESI): calcd. for $C_{32}H_{56}N_2O_2$+H$^+$: 501.4415, found: 501.4403; FT-IR (ATR): $\tilde{\nu}$ /cm^{-1} = 2920 (vs), 2852 (vs), 1520 (vs), 1501 (s), 1467 (s), 1434 (m), 1378 (w), 1338 (m), 1262 (vs), 1205 (w), 1153 (m), 1090 (s), 1056 (m), 778 (m), 722 (m), 641 (m) cm^{-1}; DSC: heating up (10 K/min) Cr_1 13 °C [-4.4 kJ mol^{-1}] Cr_2 52 °C [-57.8 kJ mol^{-1}] I; cooling down (10 K/min) Cr 26 °C [32.2 kJ mol^{-1}] I; CHN-analysis: calcd. (%) for $C_{32}H_{56}N_2O_2$: C 76.75, H 11.27, N 5.59, found: C 76.96, H 11.51, N 5.59.

4,7-Bis(tetradecyloxy)-1-methyl-benzoimidazole (L-MeBi(14)):

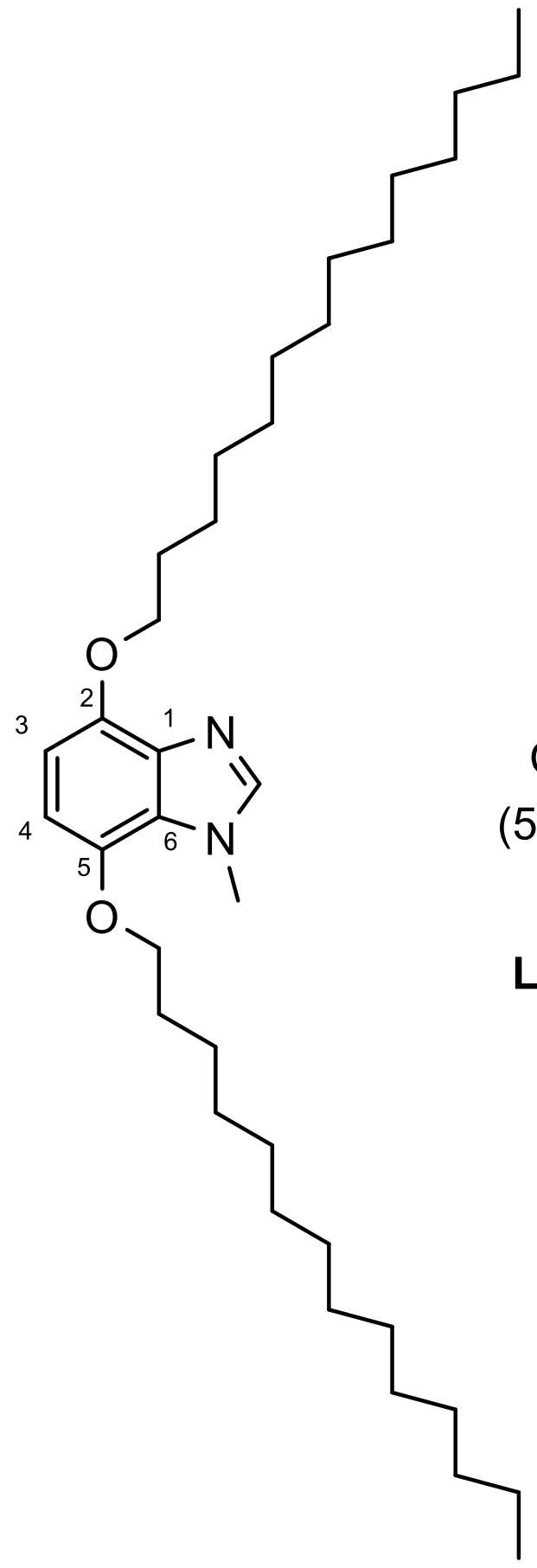

$C_{36}H_{64}N_2O_2$
(556.50 g mol^{-1})

L-MeBi(14)

According to **GP5**, **L-Bi(14)** 0.35 g, 0.64), 10 mL THF; NaH (60 %, 0.05 g, 1.20 mmol); MeI (0.06 ml, 0.96 mmol), 1 h at room temperature; the mixture was applied directly to a silica gel column and eluted successively with petroleum ether/ethyl acetate (5 : 1), followed by column chromatography with CH_2Cl_2/MeOH (20 : 1); recrystallization from EtOH yielded **L-MeBi(14)** (0.15 g, 0.26 mmol, 41 %); ^{1}H NMR (500 MHz, $CDCl_3$): δ = 0.8 (t, *J* = 7.1 Hz, 6H, 2 x CH_3), 1.25-1.38 (m, 40H, CH_2), 1.46-1.51 (m, 4H, 2 x $O(CH_2)_2C\mathit{H_2}$), 1.81-1.85 (m, 2H, 2´-H, [$OCH_2C\mathit{H_2}$]), 1.86-1.90 (m, 2H, 2´´-H [$OCH_2C\mathit{H_2}$]), 4.02 (t, *J* = 6.5 Hz, 2H, 1´-H [OCH_2]), 4.03 (s, 3H, NCH_3), 4.12 (t, *J* = 6.7 Hz, 2H, 1´´-H [OCH_2]), 6.50-6.53 (m, 2H, 3-H, 4-H), 7.66 (s, 1H, NCHN) ppm; ^{13}C NMR (125 MHz, $CDCl_3$): δ = 14.3 (2 x CH_3), 22.8 (2 x $CH_3\mathit{C}H_2$), 26.3, 26.4 (C-3´,C-3´´ [$O(CH_2)_2\mathit{C}H_2$]), 29.48, 29.51, 29.54, 29.66, 29.74, 29.78, 29.79, 29, 80, 29.83 (2´, 2´´ [2 x $OCH_2\mathit{C}H_2$], CH_2), 32.1 (CH_2), 33.9 (NCH_3), 68.7 (C-1´´[OCH_2]), 69.2 (C-1´, [OCH_2]), 103.4 (Ar C, C-4 o. C-3), 104.0 (Ar C, C-3 o. C-4), 125.8 (Ar C, C-6 or C-1), 136.2 (Ar C, C-1 or C-6), 141.5 (Ar C, C-5 or C-2), 142.7 (NCHN), 145.5 (Ar C, C-2 or C-5) ppm; MS (ESI): m/z (%) = 557.50 [M+H$^+$]; HRMS (ESI): calcd. for $C_{36}H_{64}N_2O_2+H^+$: 557.5041, found: 557.5030; FT-IR (ATR): $\tilde{\nu}$ /cm^{-1} = 2919 (vs), 2851 (vs), 1521 (vs), 1502 (m), 1468 (s), 1434 (w), 1392 (w), 1338 (m), 1262 (vs), 1154 (m), 1091 (s), 1056 (m), 779 (m), 722 (m), 641 (m) cm^{-1}; DSC: heating up (10 K/min) Cr 32 °C [-36.6 kJ mol^{-1}] I; cooling down (10 K/min) Cr 25 °C [31.9 kJ mol^{-1}] I; CHN-analysis: calcd. (%) for $C_{36}H_{64}N_2O_2$: C 77.64, H 11.58, N 5.03, found: C 77.89, H 11.86, N 5.05.

2.7. Syntheses of 1,3-dimethyl-5,6-bis(alkoxy)-benzoimidazolium iodides ($U-Me_2Bi(n)I$)

1,3-Dimethyl-5,6-bis(pentyloxy)-benzoimidazolium iodide (U-Me$_2$Bi(5)I):

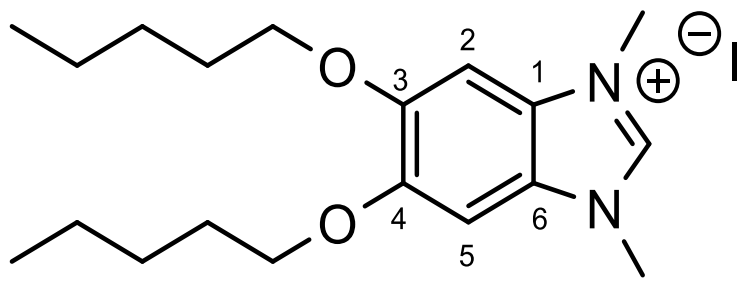

$C_{19}H_{31}IN_2O_2$
(446.14 g mol^{-1})

U-Me$_2$Bi(5)I

According to **GP6**, **U-MeBi(5)** (0.33 g, 1.08 mmol), 20 mL THF; MeI (0.70 ml, 11.24 mmol); **U-Me$_2$Bi(5)I** (0.37 g, 0.83 mmol, 76 %); ^{1}H NMR (700 MHz, $CDCl_3$): δ = 0.93 (t, *J* = 7.3 Hz, 6H, 2 x CH_2CH_3), 1.39-1.43 (m, 4H, 2 x CH_2CH_3), 1.46-1.51 (m, 4H, 2 x $O(CH_2)_2CH_2$), 1.86-1.90 (m, 4H, 2 x OCH_2CH_2), 4.08 (t, *J* = 6.5 Hz, 4H, 2 x OCH_2), 4.18 (s, 6H, 2 x NCH_3), 7.04 (s, 2H, 2 x 2-H), 10.45 (s, 1H, NCHN) ppm; ^{13}C NMR (175 MHz, $CDCl_3$): δ = 14.1 (2 x CH_3), 22.5 (2 x CH_3CH_2), 28.3 ($O(CH_2)_2CH_2$), 28.7 (CH_2), 34.3 (NCH_3), 70.2 (OCH_2), 95.3 (C-2, C-5), 125.8 (C-1, C-6), 139.6 (NCN), 150.8 (C-3, C-4) ppm.; MS (ESI): m/z (%) = 319.24 [M]$^+$; HRMS (ESI): calcd.for $C_{19}H_{31}N_2O_2^+$: 319.2380, found: 319.2376; MS (ESI$_{neg.}$): m/z (%) = 126.90 [M]$^-$; HRMS (ESI$_{neg.}$): calcd.for I^-: 126.9050, found: 126.9047; FT-IR (ATR): $\tilde{\nu}$ /cm^{-1} = 3025 (w), 2957 (s), 2933 (s), 2872 (m), 2857 (m), 1626 (w), 1561 (m), 1503 (s), 1463 (s), 1443 (s), 1418 (w), 1401 (m), 1337 (s), 1313 (w), 1264 (vs), 1231 (vs), 1139 (m), 1098 (s), 1071 (m), 1049 (s), 1025 (m), 992 (s) cm^{-1}.; CHN-Analysis: calcd. (%) for $C_{19}H_{31}IN_2O_2$: C 51.13, H 7.00, N 6.28, found: C 51.28, H 7.04, N 6.22.

1,3-Dimethyl-5,6-bis(hexyloxy)-benzoimidazolium iodide (U-Me$_2$Bi(6)I):

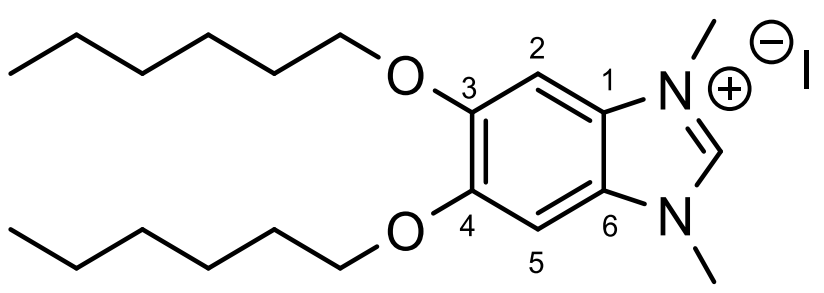

$C_{21}H_{35}IN_2O_2$
(474.17 g mol^{-1})

U-Me$_2$Bi(6)I

According to **GP6**, **U-MeBi(6)** (0.56 g, 1.68 mmol), 10 mL THF; MeI (0.52 ml, 8.42 mmol); **U-Me$_2$Bi(6)I** (42 g, 0.89 mmol, 53 %); ^{1}H NMR (700 MHz, $CDCl_3$): δ = 0.89-0.91 (m, 6H, 2 x CH_3), 1.32-1.35 (m, 8H, 2 x $(CH_2)_2CH_3$), 1.48-1.52 (m, 4H, 2 x $O(CH_2)_2CH_2$), 1.85-1.89 (m, 4H, 2 x OCH_2CH_2), 4.08 (t, *J* = 6.4 Hz, 4H, 2 x OCH_2), 4.18 (s, 6H, 2 x NCH_3), 7.03 (s, 1H, 2-H or 5-H), 7.03 (s, 1H, 5-H or 2-H), 10.45 (s, 1H, NCHN) ppm; ^{13}C NMR (175 MHz, $CDCl_3$): δ = 14.1 (2 x CH_3), 22.7 (2 x CH_3CH_2), 25.8 ($O(CH_2)_2CH_2$), 29.0 (OCH_2CH_2), 31.6 (CH_2), 34.2 (NCH_3), 70.2 (OCH_2), 95.3 (C-2, C-5), 125.8 (C-1, C-6), 139.7 (NCN), 150.8 (C-3, C-4) ppm; MS (ESI): m/z (%) = 347.27 [M]$^+$; HRMS (ESI): calcd. for $C_{21}H_{35}N_2O_2^+$: 347.2693, found: 347.2685; FT-IR (ATR): $\tilde{\nu}$ /cm^{-1} = 2954 (w),

2931 (m), 2858 (w), 1572 (w), 1502 (m), 1465 (m), 1445 (m), 1337 (w), 1262 (s), 1231 (m), 1099 (w), 1010 (w), 906 (vs), 822 (w), 723 (vs), 643 (m), 598 (w) cm^{-1}; CHN-Analysis: calcd. (%) for $C_{21}H_{35}IN_2O_2$: C 53.17, H 7.44, N 5.90, found: C 53.24, H 7.38, N 5.82.

1,3-Dimethyl-5,6-bis(heptyloxy)-benzoimidazolium iodide (U-Me$_2$Bi(7)I):

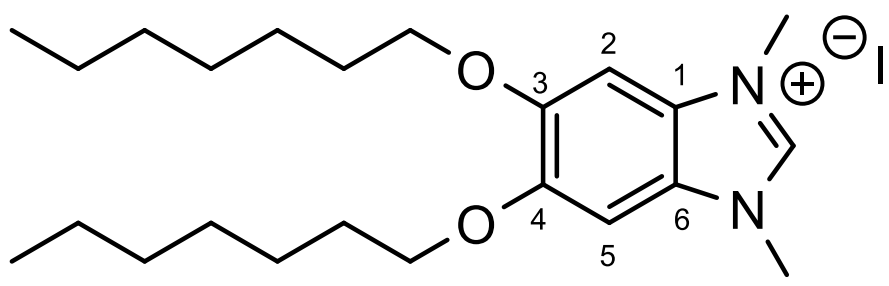

$C_{23}H_{39}IN_2O_2$
(502.21 g mol^{-1})

U-Me$_2$Bi(7)I

According to **GP6**, **U-MeBi(7)** (0.40 g, 1.15 mmol), 8 mL THF; MeI (0.36 ml, 5.78 mmol); **U-Me$_2$Bi(7)I** (0.39 g, 1.04 mmol, 91 %); ^{1}H NMR (700 MHz, $CDCl_3$): δ = 0.89 (t, *J* = 7.0 Hz, 6H, 2 x CH_3), 1.29-1.34 (m, 8H, CH_2), 1.36-1.40 (m, 4H, 2 x $O(CH_2)_2C\mathit{H_2}$), 1.87-1.91 (m, 4H, 2 x $OCH_2C\mathit{H_2}$), 4.08 (t, *J* = 6.5 Hz, 4H, 2 x OCH_2), 4.18 (s, 6H, 2 x NCH_3), 7.01 (s, 2H, 2 x 2-H), 10.54 (s, 1H, NCHN) ppm; ^{13}C NMR (175 MHz, $CDCl_3$): δ = 14.2 (2 x CH_3), 22.7 (2 x $CH_3\mathit{C}H_2$), 26.0 ($O(CH_2)_2\mathit{C}H_2$), 29.05, 29.10 ($OCH_2\mathit{C}H_2$, CH_2), 531.8 (CH_2), 34.3 (NCH_3), 70.2 (OCH_2), 95.4 (C-2, C-5), 125.8 (C-1, C-6), 139.6 (NCN), 150.8 (C-3, C-4) ppm; MS (ESI): m/z (%) = 375.30 $[M]^+$; HRMS (ESI): calcd. for $C_{23}H_{39}N_2O_2^+$: 375.3006, found: 375.2994; FT-IR (ATR): $\tilde{\nu}$ /cm^{-1} = 2956 (w), 2930 (w), 1503 (m), 1466 (m), 1446 (w), 1338 (w), 1263 (m), 1232 (m), 1099 (w), 1010 (w), 905 (vs), 724 (vs), 643 (m) cm^{-1}; CHN-Analysis: calcd. (%) for $C_{23}H_{39}IN_2O_2$: C 54.98, H 7.82, N 5.58, calcd. (%) for $C_{23}H_{39}IN_2O_2$ + 1.0 eq. H_2O: C 53.07, H 7.94, N 5.38, found: C 52.95, H 7.85, N 5.30.

1,3-Dimethyl-5,6-bis(octyloxy)-benzoimidazolium iodide (U-Me$_2$Bi(8)I):

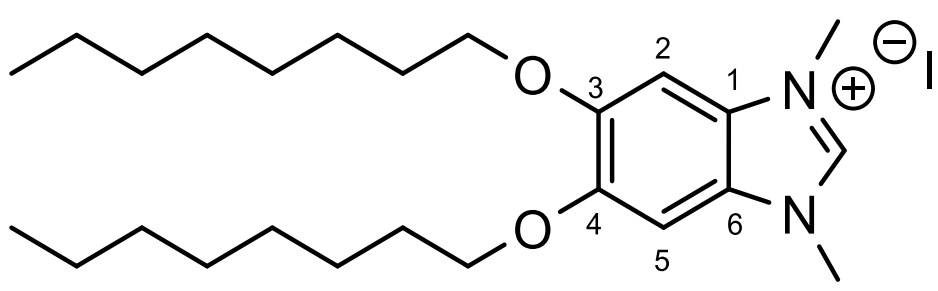

$C_{25}H_{43}IN_2O_2$
(530.24 g mol^{-1})

U-Me$_2$Bi(8)I

According to **GP6**, **U-MeBi(8)** (0.14 g, 0.37 mmol), 10 mL THF; MeI (0.13 ml, 2.01 mmol); **U-Me$_2$Bi(8)I** (0.18 g, 0.33 mmol, 91 %); ^{1}H NMR (700 MHz, $CDCl_3$): δ = 0.87 (t, *J* = 6.8 Hz, 6H, 2 x CH_3), 1.27-1.39 (m, 16H, CH_2), 1.48-1.52 (m, 4H, 2 x $O(CH_2)_2C\mathit{H_2}$), 1.86-1.90 (m, 4H, 2 x $OCH_2C\mathit{H_2}$), 4.08 (t, *J* = 6.4 Hz, 4H, 2 x OCH_2), 4.18 (s, 6H, 2x NCH_3), 7.02 (s, 2H, 2x 2-H), 10.51 (s, 1H, NCHN) ppm; ^{13}C NMR (175 MHz, $CDCl_3$): δ = 14.2 ($CH_2\mathit{C}H_3$), 22.8 (2 x $CH_3\mathit{C}H_2$), 26.1 ($O(CH_2)_2\mathit{C}H_2$), 29.08, 29.35, 29.42, 31.9 (CH_2), 34.2 (NCH_3), 70.2 (OCH_2), 95.3 (C-2, C-5), 125.8 (C-1, C-6), 139.7 (NCN), 150.9 (C-3, C-4) ppm; MS (ESI): m/z (%) = 403.33 $[M]^+$; HRMS (ESI): calcd. for $C_{25}H_{43}N_2O_2^+$: 403.3319, found: 403.3313; MS (ESI$_{neg.}$) m/z (%) = 126.90 $[M]^-$; HRMS (ESI$_{neg.}$): calcd. for I^-: 126.9050,

found: 126.9048; FT-IR (ATR): $\tilde{\nu}$ /cm^{-1} = 3029 (w), 2955 (m), 2917 (s), 2872 (m), 2849 (s), 1627 (w), 1561 (m), 1503 (s), 1464 (vs), 1444 (s), 1402 (m), 1338 (s), 1313 (w), 1265 (vs), 1231 (vs), 1143 (w), 1099 (m), 1063 (m), 1014 (m), 999 (m), 981 (m), 963 (w), 921 (m) cm^{-1}. CHN-Analysis: calcd. (%) for $C_{25}H_{43}IN_2O_2$: C 56.60, H 8.17, N 5.28, calcd. (%) for $C_{25}H_{43}IN_2O_2$ + 1.1 eq. H_2O: C 54.56, H 8.28, N 5.09, found: C 54.48, H 8.09, N 5.00.

1,3-Dimethyl-5,6-bis(nonyloxy)-benzoimidazolium iodide (U-Me$_2$Bi(9)I):

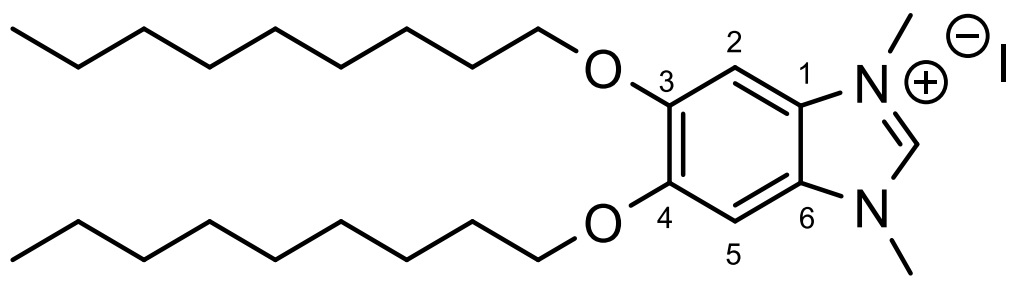

$C_{27}H_{47}IN_2O_2$
(558.27 g mol^{-1})

U-Me$_2$Bi(9)I

According to **GP6**, **U-MeBi(9)** (0.50 g, 1.19 mmol), 8 mL THF; MeI (0.37 ml, 5.94 mmol); **U-Me$_2$Bi(9)I** (0.33 g, 0.58 mmol, 49 %); ^{1}H NMR (700 MHz, $CDCl_3$): δ = 0.87 (t, *J* = 6.5 Hz, 6H, 2 x CH_3), 1.26-1.39 (m, 20H, CH_2), 1.48-1.52 (m, 4H, 2 x O(CH_2)$_2$C*H$_2$*), 1.86-1.90 (m, 4H, 2 x OCH_2C*H$_2$*), 4.08 (t, *J* = 6.5 Hz, 4H, 2 x OCH_2), 4.18 (s, 6H, 2 x NCH_3), 7.02 (s, 2H, 2 x 2-H), 10.52 (s, 1H, NCHN) ppm;

^{13}C NMR (175 MHz, $CDCl_3$): δ = 14.2 (CH_2*C*H_3), 22.8 (2 x CH_3*C*H_2), 26.1 (O(CH_2)$_2$*C*H_2), 29.08, 29.38, 29.48, 29.66, 32.0 (CH_2), 34.2 (NCH_3), 70.2 (OCH_2), 95.3 (C-2, C-5), 125.8 (C-1, C-6), 139.7 (NCN), 150.9 (C-3, C-4) ppm; MS (ESI): m/z (%) = 431.36 [M]$^+$; HRMS (ESI): calcd. for $C_{27}H_{47}N_2O_2^+$: 431.3632, found: 431.3611; MS (ESI$_{neg.}$): m/z (%) = 126.90 [M]$^-$; FT-IR (ATR): $\tilde{\nu}$ /cm^{-1} = 3026 (w), 2956 (m), 2916 (vs), 2873 (m), 2848 (s), 1626 (w), 1561 (m), 1503 (s), 1464 (vs), 1443 (s), 1418 (w), 1402 (m), 1338 (s), 1312 (w), 1266 (vs), 1233 (vs), 1144 (w), 1099 (s), 1067 (m), 1033 (w) cm^{-1}; CHN-Analysis: calcd. (%) for $C_{27}H_{47}IN_2O_2$: C 58.06, H 8.48, N 5.02; calcd. (%) for for $C_{27}H_{47}IN_2O_2$+ 0.7 eq. H_2O: C 56.77, H 8.54, N 4.90, found: C 56.67, H 8.57, N 4.82.

1,3-dimethyl-5,6-bis(decyloxy)-benzoimidazolium iodide (U-Me$_2$Bi(10)I):

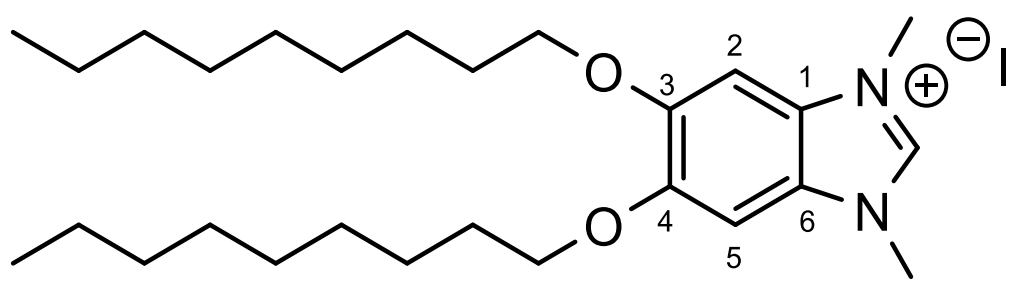

$C_{27}H_{47}IN_2O_2$
(558.27 g mol^{-1})

U-Me$_2$Bi(10)I

According to **GP6**, **U-MeBi(10)** (0.07 g, 0.16 mmol), 6 mL THF; MeI (0.07 ml, 1.12 mmol); **U-Me$_2$Bi(10)I** (0.06 g, 0.11 mmol, 69 %); ^{1}H NMR (700 MHz, $CDCl_3$): δ = 0.86 (t, *J* = 7.1 Hz, 6H, 2 x CH_3), 1.25-1.39 (m, 24H, CH_2), 1.48-1.52 (m, 4H, 2 x O(CH_2)$_2$C*H$_2$*), 1.85-1.90 (m, 4H, 2 x OCH_2C*H$_2$*), 4.08 (t, *J* = 6.5 Hz, 4H, 2x OCH_2), 4.18 (s, 6H, 2x NCH_3), 7.02 (s, 2H, 2x 2-H), 10.49 (s, 1H, NCHN)

ppm; ^{13}C NMR (175 MHz, $CDCl_3$): δ = 14.2 ($CH_2\mathit{C}H_3$), 22.8 (2 x $CH_3\mathit{C}H_2$), 26.1 ($O(CH_2)_2\mathit{C}H_2$), 29.08, 29.45, 29.47, 29.67, 29.71, 32.0 (CH_2), 34.2 (NCH_3), 70.2 (OCH_2), 95.3 (C-2, C-5), 125.8 (C-1, C-6), 139.7 (NCN), 150.9 (C-3, C-4) ppm; MS (ESI): m/z (%) = 459.39 $[M]^+$; HRMS (ESI): calcd. for $C_{29}H_{51}N_2O_2^+$: 459.3945, found: 459.3935; FT-IR (ATR): $\tilde{\nu}$ /cm^{-1} = 2955 (m), 2916 (vs), 2873 (m), 2848 (vs), 1628 (w), 1561 (m), 1504 (s), 1466 (s), 1445 (m), 1418 (w), 1404 (w), 1340 (m), 1312 (w), 1267 (vs), 1232 (s) cm^{-1}; CHN-Analysis: calcd. (%) for $C_{29}H_{51}IN_2O_2$: C 59.37, H 8.76, N 4.78, calcd. (%) for $C_{29}H_{51}IN_2O_2$ + 0.2 eq. H_2O: C 59.06, H 8.79, N 4.72, found: C 59.06, H 8.91, N 4.56.

1,3-Dimethyl-5,6-bis(dodecyloxy)-benzoimidazolium iodide (U-Me₂Bi(12)I):

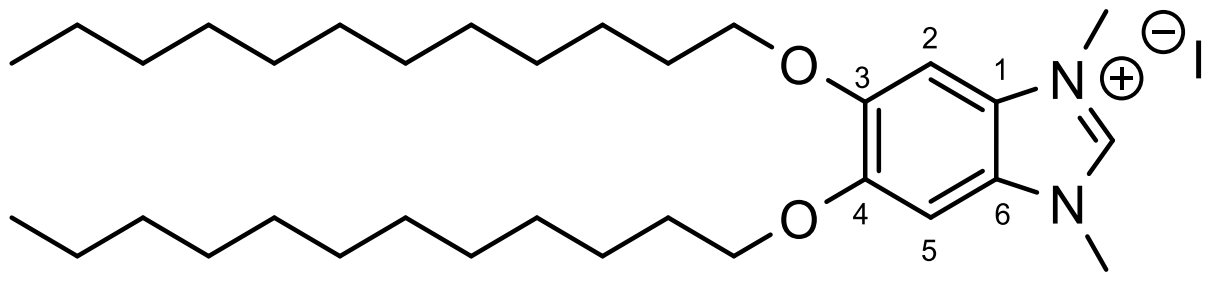

$C_{33}H_{59}IN_2O_2$
(642.36 g mol^{-1})

U-Me$_2$Bi(12)I

According to **GP6**, **U-Bi(12)** (1.00 g, 2.05 mmol), 50 mL THF; NaH: 0.13 g (60 %, 3.20 mmol), MeI (1.2 ml, 19.27 mmol); **U-Me$_2$Bi(12)I** (0.18 g, 0.33 mmol, 91 %); ^{1}H NMR (700 MHz, $CDCl_3$): δ = 0.86 (t, *J* = 7.0 Hz, 6H, 2 x CH_3), 1.25-1.38 (m, 32H, CH_2), 1.47-1.52 (m, 4H, 2 x $O(CH_2)_2C\mathit{H_2}$), 1.85-1.89 (m, 4H, 2 x $OCH_2C\mathit{H_2}$), 4.07 (t, *J* = 6.5 Hz, 4H, 2x OCH_2), 4.18 (s, 6H, 2x NCH_3), 7.02 (s, 2H, 2 x 2-H), 10.49 (s, 1H, NCHN) ppm; ^{13}C NMR (175 MHz, $CDCl_3$): δ = 14.2 ($CH_2\mathit{C}H_3$), 22.8 (2 x $CH_3\mathit{C}H_2$), 26.1 ($O(CH_2)_2\mathit{C}H_2$), 29.08, 29.47, 29.49, 29.71, 29.72, 29.76, 29.81, 32.0 (CH_2), 34.2 (NCH_3), 70.2 (OCH_2), 95.3 (C-2, C-5), 125.8 (C-1, C-6), 139.7; (NCN), 150.8 (C-3, C-4) ppm; MS (ESI): m/z (%) = 515.46 $[M]^+$; HRMS (ESI): calcd. for $C_{33}H_{59}N_2O_2^+$: 515.4571, found: 515.4574; FT-IR (ATR): $\tilde{\nu}$ /cm^{-1} = 2956 (m), 2916 (vs), 2874 (m), 2848 (vs), 1562 (w), 1504 (m), 1466 (s), 1445 (m), 1404 (w), 1340 (m), 1268 (s), 1233 (s), 1099 (m), 1069 (w), 1031 (w), 997 (m), 925 (w) cm^{-1}; CHN-Analysis: calcd. (%) for $C_{33}H_{59}IN_2O_2$: C 61.67, H 9.25, N 4.36, calcd. (%) for $C_{33}H_{59}IN_2O_2$ + 0.3 eq. H_2O: C 61.15, H 9.27, N 4.32, found: C 60.91, H 9.21, N 4.28.

1,3-Dimethyl-5,6-bis(tetradecyloxy)-benzoimidazolium iodide (U-Me₂Bi(14)I):

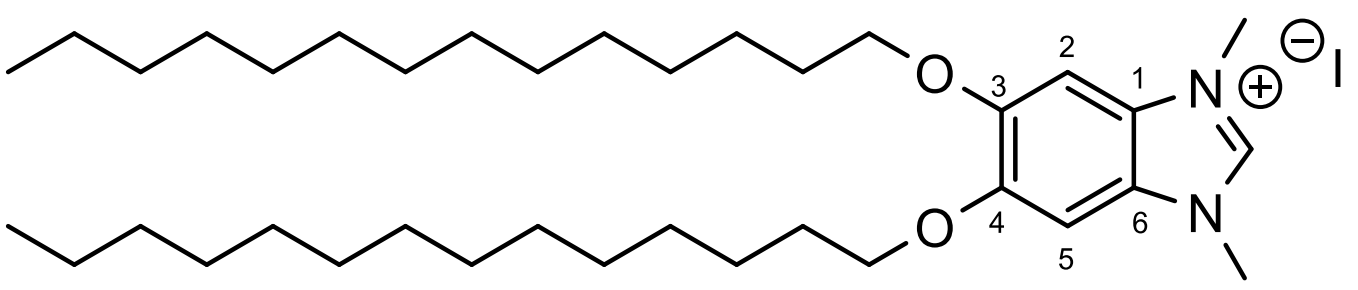

$C_{37}H_{67}IN_2O_2$
(698.42 g mol^{-1})

U-Me$_2$Bi(14)I

According to **GP6**, **U-MeBi(14)** (0.36 g, 0.64 mmol), 9 mL THF; MeI (0.10 ml, 1.61 mmol); **U-Me$_2$Bi(14)I** (0.37 g, 0.53 mmol, 83 %); ^{1}H NMR (700 MHz, $CDCl_3$): δ = 0.87 (t, *J* = 7.1 Hz, 6H, 2 x CH_3), 1.26-1.40 (m, 40H, CH_2), 1.48-1.53 (m, 4H, 2 x $O(CH_2)_2C\mathit{H_2}$), 1.85-1.89 (m, 4H, 2 x $OCH_2C\mathit{H_2}$),

4.09 (t, J = 6.5 Hz, 4H, 2 x OCH_2), 4.18 (s, 6H, 2 x NCH_3), 7.04 (s, 2H, 2 x 2-H), 10.52 (s, 1H, NCHN) ppm; ^{13}C NMR (175 MHz, $CDCl_3$): δ = 14.3 ($CH_2\mathit{C}H_3$), 22.8 (2 x $CH_3\mathit{C}H_2$), 26.1 ($O(CH_2)_2\mathit{C}H_2$), 29.1, 29.5, 29.75, 29.76, 29.80, 29.85, 32.1 (CH_2), 34.0 (NCH_3), 70.2 (OCH_2), 95.2 (C-2, C-5), 125.8 (C-1, C-6), 139.9 (NCN), 150.9 (C-3, C-4) ppm; MS (ESI): m/z (%) = 571.52 $[M]^+$; HRMS (ESI): calcd. for $C_{37}H_{67}N_2O_2^+$: 571.5197, found: 571.5187; FT-IR (ATR): $\tilde{\nu}$ /cm^{-1} = 3027 (w), 2956 (m), 2915 (vs), 2874 (m), 2848 (vs), 1564 (m), 1504 (s), 1465 (s), 1443 (m), 1404 (m), 1340 (m), 1267 (vs), 1232 (s), 1142 (w), 1096 (m), 1073 (m), 1053 (w), 1017 (m), 1006 (m), 987 (m) cm^{-1}; CHN-Analysis: calcd. (%) for $C_{37}H_{67}IN_2O_2$: C 63.59, H 9.66, N 4.01, calcd. (%) for $C_{37}H_{67}IN_2O_2$ + 0.8 eq. H_2O: C 62.31, H 9.69, N 3.93, found: C 62.22, H 9.63, N 3.85.

2.8. Syntheses of 1,3-dimethyl-4,7-bis(alkoxy)-benzoimidazolium iodides (L-Me$_2$Bi(n)I)

1,3-Dimethyl-4,7-bis(pentyloxy)-benzoimidazolium iodide (L-Me$_2$Bi(5)I):

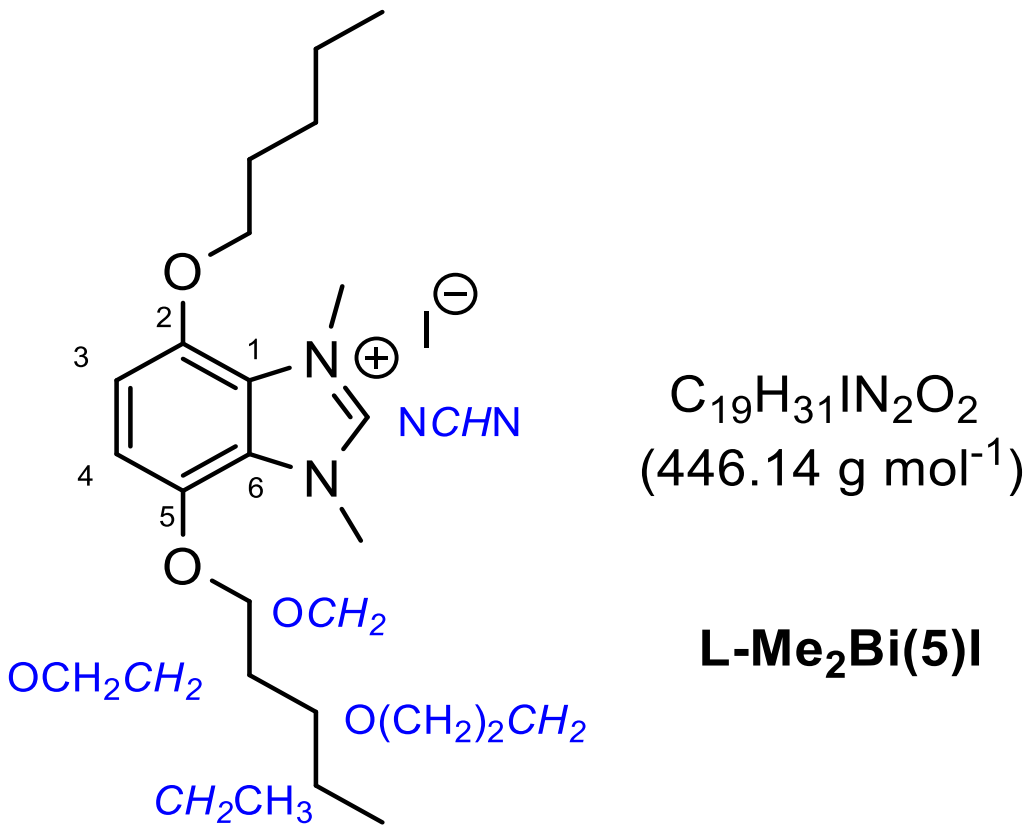

According to **GP6**, **L-MeBi(5)** (0.31 g, 1.0 mmol), 10 mL THF; MeI (0.53 ml, 8.6 mmol); **L-Me$_2$Bi(5)I** (0.35 g, 0.79 mmol, 77 %); ^{1}H NMR (700 MHz, $CDCl_3$): δ = 0.93 (t, J = 7.1 Hz, 6H, 2 x $CH_2\mathit{C}\mathit{H}_3$), 1.37-1.47 (m, 8H, 2 x $C\mathit{H}_2CH_3$, 2 x $O(CH_2)_2C\mathit{H}_2$), 1.83-1.87 (m, 4H, 2 x $OCH_2C\mathit{H}_2$), 4.06 (t, J = 6.5 Hz, 4H, 2 x OCH_2), 4.34 (s, 6H, 2 x NCH_3), 6.77 (s, 2H, 3-H, 4-H), 10.75 (s, 1H, NCHN) ppm; ^{13}C NMR (175 MHz, $CDCl_3$): δ = 14.0 (2 x $CH_2\mathit{C}H_3$), 22.4 (2 x $\mathit{C}H_2CH_3$), 28.3 (2 x $O(CH_2)_2\mathit{C}H_2$), 28.8 (2 x $OCH_2\mathit{C}H_2$), 37.0 (NCH_3), 69.6 (2 x OCH_2), 108.3 (C-3, C-4), 123.6 (C-1, C-6), 142.0 (C-2, C-5), 142.6 (NCN) ppm; MS (ESI): m/z (%) = 319.24 $[M]^+$; HRMS (ESI): calcd. for $C_{19}H_{31}N_2O_2^+$: 319.2380, found: 319.2374; FT-IR (ATR): $\tilde{\nu}$ /cm^{-1} = 2959 (m), 2936 (m), 2874 (w), 2859 (w), 2203 (w), 1524 (s), 1460 (m), 1340 (m), 1278 (s), 1152 (m), 1075 (w), 1016 (w), 917 (s), 815 (w), 729 (vs), 643 (w), 622 (w), 577 (w) cm^{-1}; CHN-Analysis: calcd. (%) for $C_{19}H_{31}IN_2O_2$: C 51.12, H 7.00, N 6.28, found: C 51.02, H 6.97, N 6.22.

1,3-Dimethyl-4,7-bis(hexyloxy)-benzoimidazolium iodide (L-Me_2Bi(6)I):

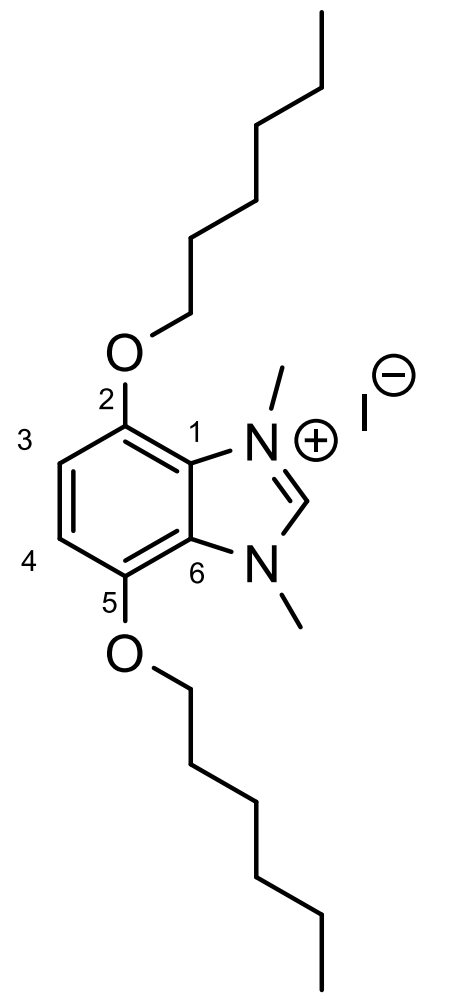

$C_{21}H_{35}IN_2O_2$
(474.17 g mol^{-1})

L-Me_2Bi(6)I

According to **GP6**, **L-MeBi(6)** (0.10 g, 0.29 mmol), 5 mL THF; MeI (0.09 ml, 1.4 mmol); **L-Me_2Bi(6)** (0.09 g, 0.19 mmol, 65 %); ^{1}H NMR (700 MHz, $CDCl_3$): δ = 0.90 (t, *J* = 6.5 Hz, 6H, 2 x $CH_2C\mathit{H_3}$), 1.31-1.37 (m, 8H, CH_2), 1.44-1.48 (m, 4H, 2 x $O(CH_2)_2C\mathit{H_2}$), 1.82-1.86 (m, 4H, 2 x $OCH_2C\mathit{H_2}$), 4.06 (t, *J* = 6.5 Hz, 4H, 2 x OCH_2), 4.33 (s, 6H, 2 x NCH_3), 6.77 (s, 2H, 3-H, 4-H), 10.75 (s, 1H, NCHN) ppm; ^{13}C NMR (175 MHz, $CDCl_3$): δ = 14.1 (2 x $CH_2\mathit{C}H_3$), 22.6 (2 x CH_2), 25.8 (2 x $O(CH_2)_2\mathit{C}H_2$), 29.0, 31.5 (2 x 2 CH_2), 36.9 (NCH_3), 69.5 (2 x OCH_2), 108.1 (C-3, C-4), 123.5 (C-1, C-6), 141.9 (C-2, C-5), 141.9, 142.4 (NCN) ppm; MS (ESI): m/z (%) = 347.27 $[M]^+$; HRMS (ESI): calcd. for $C_{21}H_{35}N_2O_2^+$: 347.2693, found: 347.2686; FT-IR (ATR): $\tilde{\nu}$ /cm^{-1} = 3498 (m), 3032 (w), 2939 (s), 2857 (m), 1529 (vs), 1487 (w), 1463 (m), 1357 (w), 1343 (m), 1282 (vs), 1254 (w) cm^{-1}; CHN-Analysis: calcd. (%) for $C_{21}H_{35}IN_2O_2$: C 53.17, H 7.44, N 5.90, found: C 53.26, H 7.47, N 5.84.

1,3-Dimethyl-4,7-bis(heptyloxy)-benzoimidazolium iodide (L-Me_2Bi(7)I):

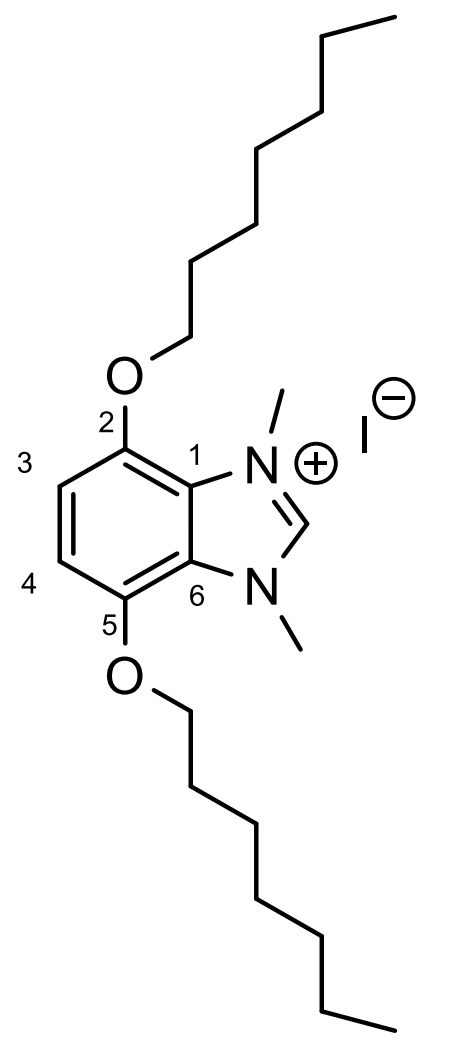

$C_{23}H_{39}IN_2O_2$
(502.21 g mol^{-1})

L-Me_2Bi(7)I

According to **GP6**, **L-MeBi(7)** (0.30 g, 0.80 mmol), 9 mL THF; MeI (0.26 ml, 4.0 mmol); **L-Me$_2$Bi(7)** (0.10 g, 0.19 mmol, 23 %); ^{1}H NMR (700 MHz, CDCl$_3$): δ = 0.89 (t, *J* = 6.8 Hz, 6H, 2 x CH$_2$C*H$_3$*), 1.28-1.38 (m, 12H, CH$_2$), 1.43-1.48 (m, 4H, 2 x O(CH$_2$)$_2$C*H$_2$*), 1.82-1.87 (m, 4H, 2 x OCH$_2$C*H$_2$*), 4.06 (t, *J* = 6.5 Hz, 4H, 2 x OCH$_2$), 4.33 (s, 6H, 2 x NCH$_3$), 6.77 (s, 2H, 3-H, 4-H), 10.77 (s, 1H, NCHN) ppm; ^{13}C NMR (175 MHz, CDCl$_3$): δ = 14.2 (2 x CH$_2$*C*H$_3$), 22.7 (2 x CH$_2$), 26.1 (2 x O(CH$_2$)$_2$*C*H$_2$), 29.04, 29.08, 31.8 (2 x 3 CH$_2$), 36.9 (NCH$_3$), 69.5 (2 x OCH$_2$), 108.1 (C-3, C-4), 123.5 (C-1, C-6), 141.9 (C-2, C-5), 142.36, 142.39 (NCN) ppm; MS (ESI): m/z (%) = 375.30 [M]$^+$; HRMS (ESI): calcd. for $C_{23}H_{39}N_2O_2^+$: 375.3006, found: 375.2998; FT-IR (ATR): $\tilde{\nu}$ /cm^{-1} = 2956 (w), 2927 (m), 2856 (w), 2202 (w), 1525 (s), 1461 (w), 1341 (m), 1277 (s), 1152 (m), 1074 (w), 909 (s), 815 (w), 729 (vs), 643 (w), 621 (w) cm^{-1}; CHN-Analysis: calcd. (%) for $C_{23}H_{39}IN_2O_2$: C 54.98, H 7.82, N 5.58, found: C 55.11, H 7.84, N 5.55.

1,3-Dimethyl-4,7-bis(octyloxy)-benzoimidazolium iodide (L-Me$_2$Bi(8)I):

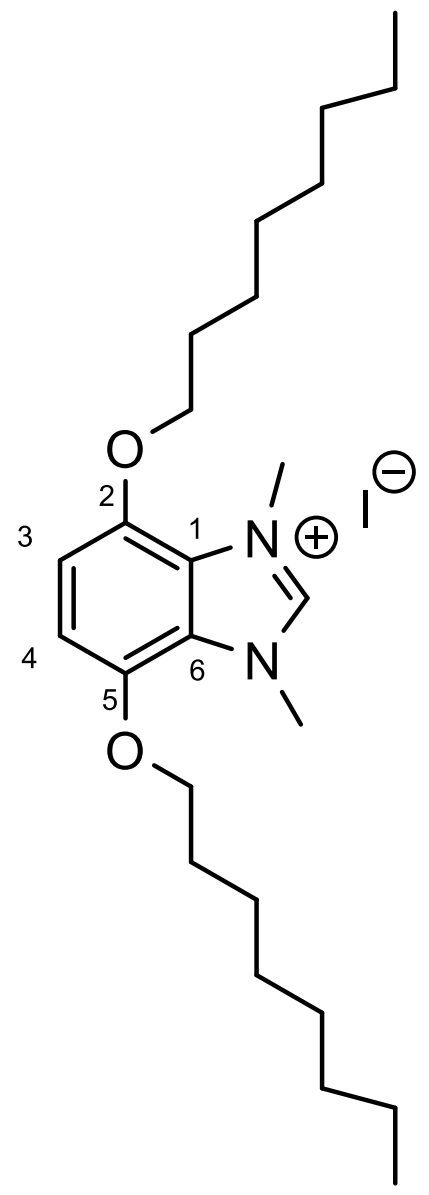

$C_{25}H_{43}IN_2O_2$
(530.24 g mol^{-1})

L-Me$_2$Bi(8)I

According to **GP6**, **L-MeBi(8)** (0.46 g, 1.2 mmol), 9 mL THF; MeI (0.50 ml, 8.0 mmol); **L-Me$_2$Bi(8)** (0.45 g, 0.84 mmol, 70 %); ^{1}H NMR (700 MHz, CDCl$_3$): δ = 0.87 (t, *J* = 7.1 Hz, 6H, 2 x CH$_2$C*H$_3$*), 1.24-1.37 (m, 16H, CH$_2$), 1.43-1.47 (m, 4H, 2 x O(CH$_2$)$_2$C*H$_2$*), 1.81-1.85 (m, 4H, 2 x OCH$_2$C*H$_2$*), 4.05 (t, *J* = 6.5 Hz, 4H, 2 x OCH$_2$), 4.33 (s, 6H, 2 x NCH$_3$), 6.76 (s, 2H, 3-H, 4-H), 10.74 (s, 1H, NCHN) ppm; ^{13}C NMR (175 MHz, CDCl$_3$): δ = 14.2 (2 x CH$_2$*C*H$_3$), 22.7 (2 x CH$_2$), 26.2 (2 x O(CH$_2$)$_2$*C*H$_2$), 29.1, 29.26, 29.32, 31.8 (CH$_2$), 36.9 (NCH$_3$), 69.5 (2 x OCH$_2$), 108.1 (C-3, C-4), 123.5 (C-1, C-6), 141.9 (C-2, C-5), 142.40 (NCN) ppm; MS (ESI): m/z (%) = 403.33 [M]$^+$; HRMS (ESI): calcd. for $C_{25}H_{43}N_2O_2^+$: 403.3319, found: 403.15; FT-IR (ATR): $\tilde{\nu}$ /cm^{-1} = 2928 (w), 1526 (m), 1466 (w), 1341 (w), 1275 (m), 1151 (w), 906 (vs), 726 (vs), 643 (m), 621 (w) cm^{-1}.; CHN-Analysis: calcd. (%) for $C_{25}H_{43}IN_2O_2$: C 56.60, H 8.17, N 5.28, found: C 56.54, H 8.20, N 5.19.

1,3-Dimethyl-4,7-bis(nonyloxy)-benzoimidazolium iodide (L-Me$_2$Bi(9)I):

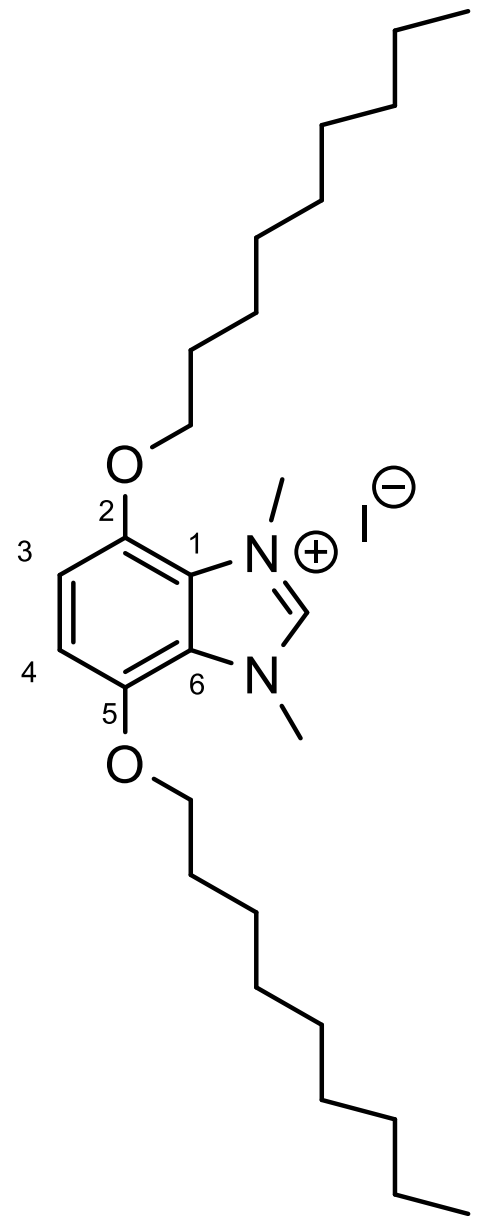

$C_{27}H_{47}IN_2O_2$
(558.27 g mol^{-1})

L-Me$_2$Bi(9)I

According to **GP6**, **L-MeBi(9)** (0.72 g, 1.7 mmol), 9 mL THF; MeI (0.31 ml, 5.0 mmo); **L-Me$_2$Bi(9)** (0.46 g, 0.83 mmol, 49 %); ^{1}H NMR (700 MHz, CDCl$_3$): δ = 0.87 (t, *J* = 6.9 Hz, 6H, 2 x CH$_2$C*H$_3$*), 1.26-1.38 (m, 20H, CH$_2$), 1.43-1.48 (m, 4H, 2 x O(CH$_2$)$_2$C*H$_2$*), 1.82-1.86 (m, 4H, 2 x OCH$_2$C*H$_2$*), 4.06 (t, *J* = 6.4 Hz, 4H, 2 x OCH$_2$), 4.33 (s, 6H, 2 x NCH$_3$), 6.77 (s, 2H, 3-H, 4-H), 10.79 (s, 1H, NCHN) ppm; ^{13}C NMR (175 MHz, CDCl$_3$): δ = 14.2 (2 x CH$_2$*C*H$_3$), 22.8 (2 x CH$_2$), 26.2 (2 x O(CH$_2$)$_2$*C*H$_2$), 29.1, 29.3, 29.4, 29.6, 31.9 (10 x CH$_2$), 36.9 (NCH$_3$), 69.5 (2 x OCH$_2$), 108.1 (C-3, C-4), 123.5 (C-1, C-6), 141.9 (C-2, C-5), 142.40 (NCN) ppm; MS (ESI): m/z (%) = 431.36 [M]$^+$; HRMS (ESI): calcd. for $C_{27}H_{47}N_2O_2^+$: 431.3632, found: 431.3633; FT-IR (ATR): $\tilde{\nu}$ /cm^{-1} = 2927 (w), 1526 (m), 1467 (w), 1342 (w), 1275 (m), 1151 (w), 905 (vs), 723 (vs), 643 (m), 622 (w) cm^{-1}; CHN-Analysis: calcd. (%) for $C_{27}H_{47}IN_2O_2$: C 58.06, H 8.48, N 5.02, found: C 58.14, H 8.49, N 4.93.

1,3-Dimethyl-4,7-bis(decyloxy)-benzoimidazolium iodide (L-Me_2Bi(10)I):

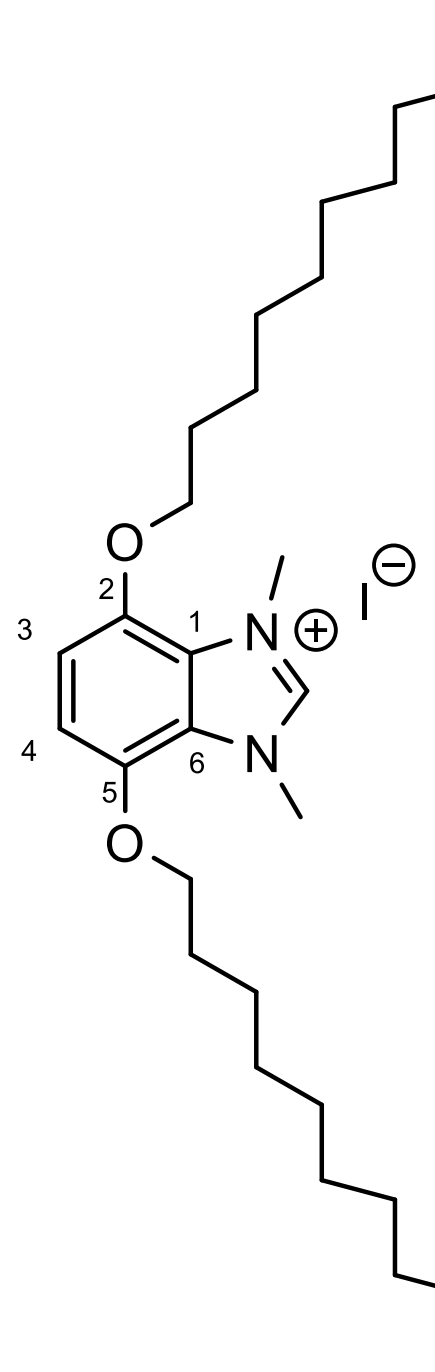

$C_{29}H_{51}IN_2O_2$
(558.27 g mol^{-1})

L-Me_2Bi(10)I

According to **GP6**, **L-MeBi(10)** (0.12 g, 0.26 mmol), 8 mL THF; MeI (0.08 ml, 0.13 mmol); **L-Me_2Bi(10)** (0.08 g, 0.13 mmol, 49 %); ^{1}H NMR (700 MHz, $CDCl_3$): δ = 0.87 (t, J = 13.9 Hz, 6H, 2 x CH_3), 1.24-1.37 (m, 24H, CH_2), 1.43-1.47 (m, 4H, 2 x $O(CH_2)_2C\mathit{H_2}$), 1.82-1.86 (m, 4H, 2 x $OCH_2C\mathit{H_2}$), 4.05 (t, J = 6.5 Hz, 4H, 2 x OCH2), 4.33 (s, 6H, 2 x NCH3), 6.77 (s, 2H, 3-H, 4-H), 10.72 (s, 1H, NCHN) ppm; ^{13}C NMR (175 MHz, $CDCl_3$): δ = 14.2 ($CH_2\mathit{C}H_3$), 22.8 (2 x $CH_3\mathit{C}H_2$), 26.2 (2 x $O(CH_2)_2\mathit{C}H_2$), 29.07, 29.38, 29.63, 32.0 (CH_2), 37.0 (NCH_3), 69.5 (OCH_2), 108.1 (C-3, C-4), 123.5 (C-1, C-6), 141.9 (C-2, C-5), 142.39, 142.40 (NCN) ppm; MS (ESI): m/z (%) = 459.39 $[M]^+$; HRMS (ESI): calcd. for $C_{29}H_{51}N_2O_2^+$: 459.3945, found: 459.3934; FT-IR (ATR): $\tilde{\nu}$ /cm^{-1} = 2917 (vs), 2849 (s), 1524 (vs), 1462 (s), 1398 (w), 1341 (s), 1277 (vs), 1229 (m), 1146 (s), 1093 (m), 1073 (s), 1026 (m), 1009 (m), 992 (m), 824 (w), 800 (m), 738 (s) cm^{-1}; CHN-Analysis: calcd. (%) for $C_{29}H_{51}IN_2O_2$: C 59.37, H 8.76, N 4.78, found: C 59.22, H 8.92, N 4.79.

1,3-Dimethyl-4,7-bis(dodecyloxy)-benzoimidazolium iodide (L-Me_2Bi(12)I):

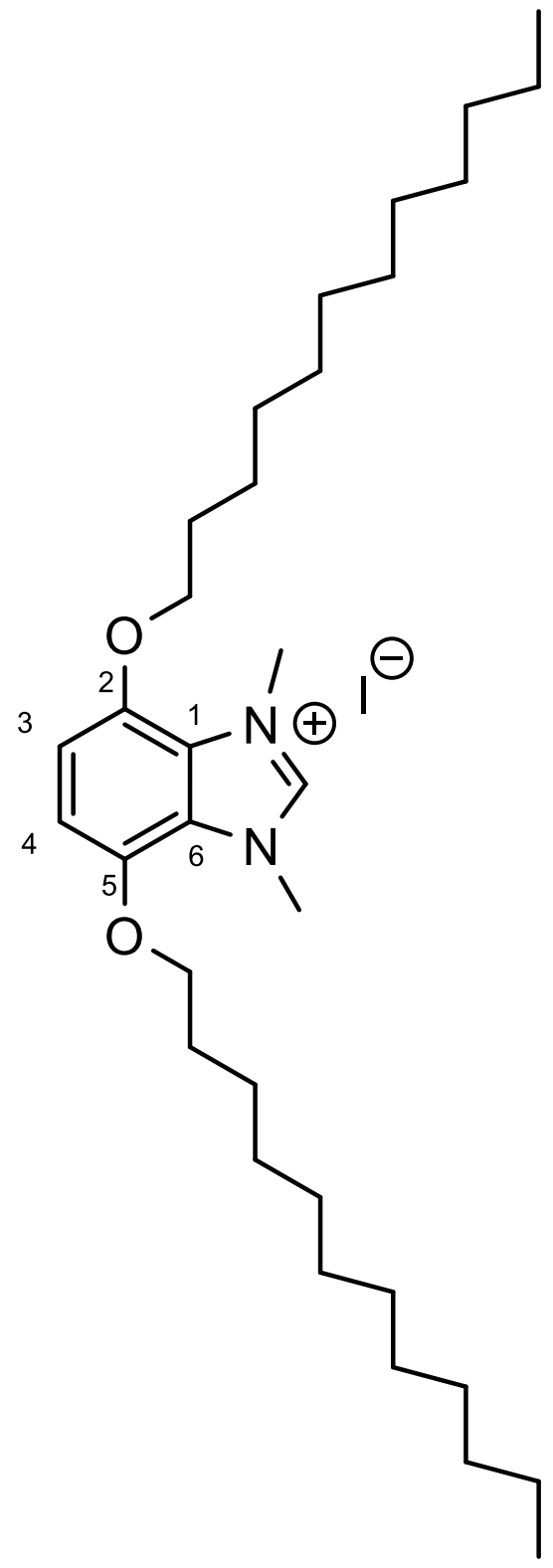

$C_{33}H_{59}IN_2O_2$
(642.36 g mol^{-1})

L-Me_2Bi(12)I

According to **GP6**, **L-MeBi(12)** (0.47 g, 0.94 mmol), 10 mL THF; MeI (0.29 ml, 4.69 mmol); **L-Me_2Bi(12)** (0.54 g, 0.84 mmol, 90 %); ^{1}H NMR (700 MHz, $CDCl_3$): δ = 0.87 (t, *J* = 7.0 Hz, 6H, 2 x CH_3), 1.25-1.36 (m, 32H, CH_2), 1.43-1.47 (m, 4H, 2 x $O(CH_2)_2CH_2$), 1.82-1.86 (m, 4H, 2 x OCH_2CH_2), 4.05 (t, *J* = 6.4 Hz, 4H, 2 x OCH_2), 4.33 (s, 6H, 2 x NCH_3), 6.77 (s, 2H, 3-H, 4-H), 10.76 (s, 1H, NCHN) ppm; ^{13}C NMR (175 MHz, $CDCl_3$): δ = 14.2 (CH_2CH_3), 22.8 (2 x CH_3CH_2), 26.2 (2 x $O(CH_2)_2CH_2$), 29.07, 29.39, 29.45, 29.64, 29.69, 29.73, 29.74, 32.0 (CH_2), 36.9 (NCH_3), 69.5 (OCH_2), 108.1 (C-3, C-4), 123.5 (C-1, C-6), 141.9 (C-2, C-5), 142.4 (br, NCN) ppm; MS (ESI): m/z (%) = 515.46 $[M]^+$; HRMS (ESI): calcd. for $C_{33}H_{59}N_2O_2^+$: 515.4571, found: 515.4572; FT-IR (ATR): $\tilde{\nu}$ /cm^{-1} = 2916 (vs), 2849 (s), 1525 (s), 1463 (m), 1342 (m), 1278 (s), 1150 (m), 1072 (w), 910 (w), 800 (w), 737 (m), 688 (w), 619 (w) cm^{-1}; CHN-Analysis: calcd. (%) for $C_{33}H_{59}IN_2O_2$: C 61.67, H 9.25, N 4.36, found: C 61.92, H 9.29, N 4.34.

1,3-Dimethyl-4,7-bis(tetradecyloxy)-benzoimidazolium iodide (L-Me_2Bi(14)I):

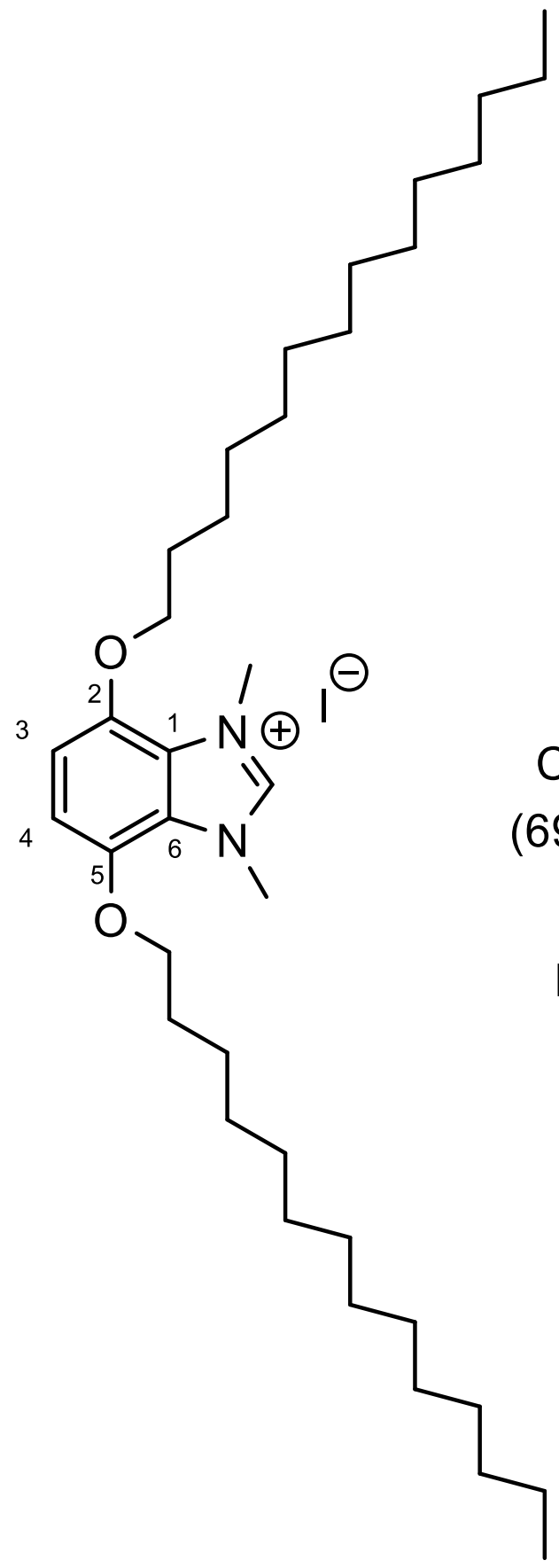

$C_{37}H_{67}IN_2O_2$
(698.42 g mol^{-1})

L-Me_2Bi(14)I

According to **GP6**, **L-MeBi(14)** (0.15 g, 0.27 mmol), 10 mL THF; MeI (0.08 ml, 1.29 mmol); **L-Me_2Bi(12)** (0.09 g, 0.13 mmol, 47 %); ^{1}H NMR (700 MHz, $CDCl_3$): δ = 0.86 (t, *J* = 7.1 Hz, 6H, 2 x CH_3), 1.25-1.37 (m, 40H, CH_2), 1.43-1.47 (m, 4H, 2 x $O(CH_2)_2C\mathit{H_2}$), 1.82-1.86 (m, 4H, 2 x $OCH_2C\mathit{H_2}$), 4.06 (t, *J* = 6.5 Hz, 4H, 2 x OCH_2), 4.33 (s, 6H, 2 x NCH_3), 6.77 (s, 2H, 3-H, 4-H), 10.77 (s, 1H, NCHN) ppm; ^{13}C NMR (175 MHz, $CDCl_3$): δ = 14.2 ($CH_2\mathit{C}H_3$), 22.8 (2 x $CH_3\mathit{C}H_2$), 26.2 (2 x $O(CH_2)_2\mathit{C}H_2$), 29.08, 29.40, 29.47, 29.65, 29.71, 29.75, 29.78, 29.80, 32.0 (CH_2), 36.9 (NCH_3), 69.5 (OCH_2), 108.1 (C-3, C-4), 123.5 (C-1, C-6), 141.9 (C-2, C-5), 142.38, 142.42 (NCN) ppm; MS (ESI): m/z (%) = 571.52 $[M]^+$; HRMS (ESI): calcd. for $C_{37}H_{67}N_2O_2^+$: 571.5197, found: 571.5187; FT-IR (ATR): $\tilde{\nu}$ /cm^{-1} = 2915 (vs), 2849 (vs), 1526 (vs), 1462 (s), 1399 (w), 1341 (m), 1278 (vs), 1223 (w), 1149 (s), 1072 (m), 1037 (w), 1010 (m), 992 (m), 930 (w), 804 (m), 738 (m) cm^{-1}; CHN-Analysis: calcd. (%) for $C_{37}H_{67}IN_2O_2$: C 63.59, H 9.66, N 4.01, found: C 63.50, H 9.69, N 3.84.

3. Mesomorphic Properties

3.1. POM Results

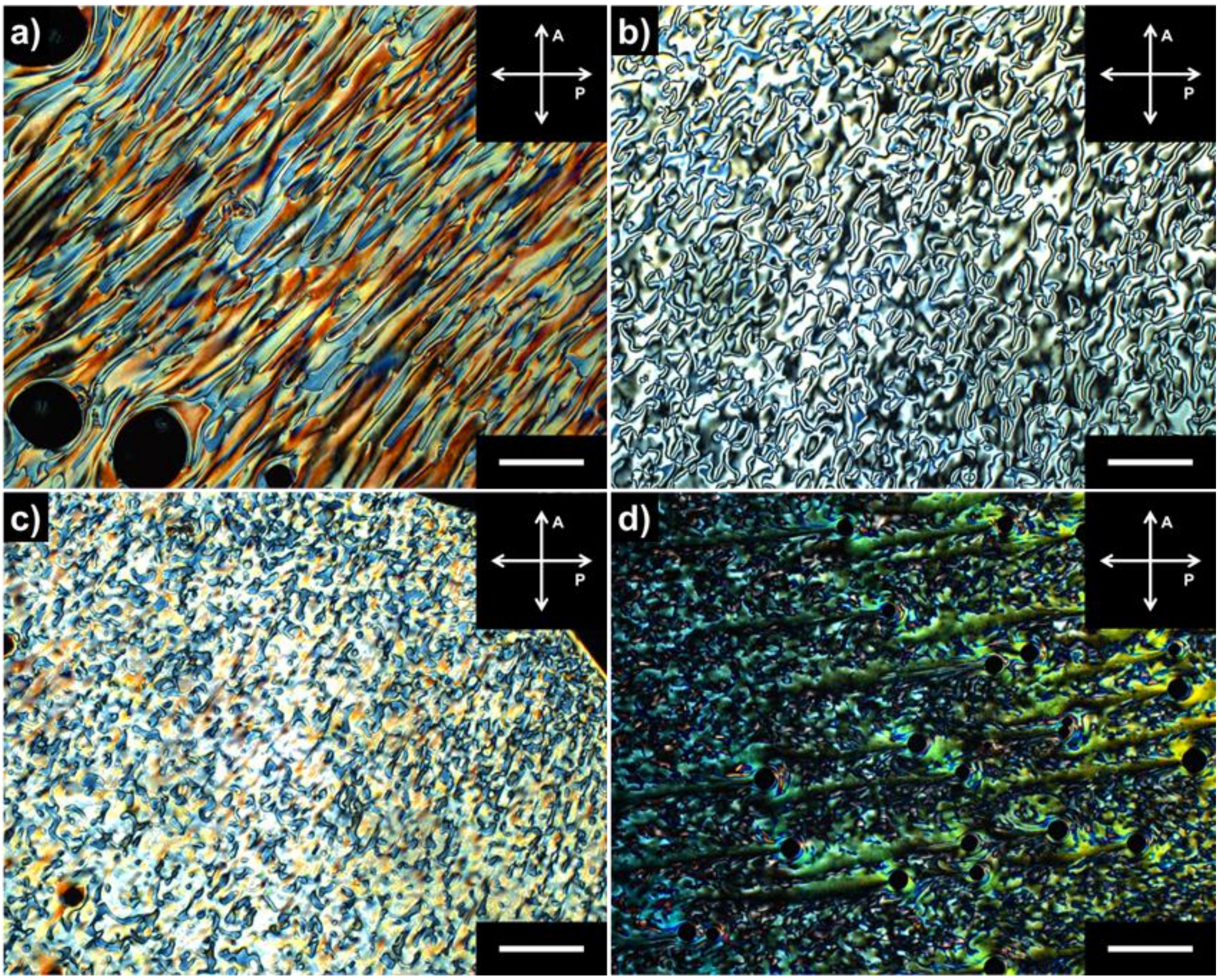

Figure S1. Characteristic nematic schlieren textures obtained on the POM (crossed polarizers, sample sandwiched between two glass slides) upon cooling of: a) **L-Bi(10)** (52.3 °C, cooling rate: 0.1 K min^{-1}); b) **L-Bi(12)** (55.1 °C, cooling rate: 0.1 K min^{-1}); c) **L-Bi(12)** (49.0 °C, cooling rate: 0.1 K min^{-1}); d) **L-Bi(14)** (45.7 °C, 1 K min^{-1}). The scale bar is 200 µm.

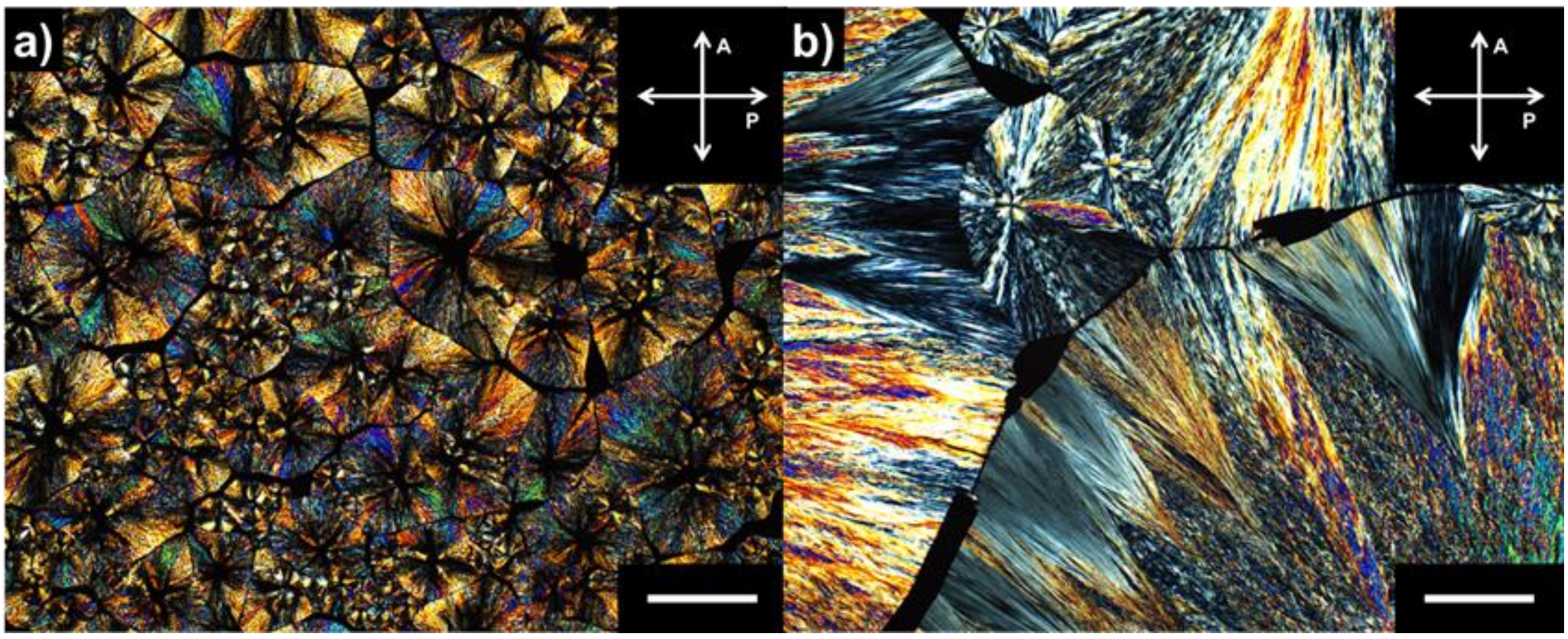

Figure S2. Crystalline Textures obtained on the POM (crossed polarizers, sample sandwiched between two glass slides) of: a) **U-Bi(14)** (59.0 °C, heating rate: 1 K min^{-1}); b) **U-Bi(14)** (90.0 °C, cooling rate: 1 K min^{-1}); The scale bar is 200 μm.

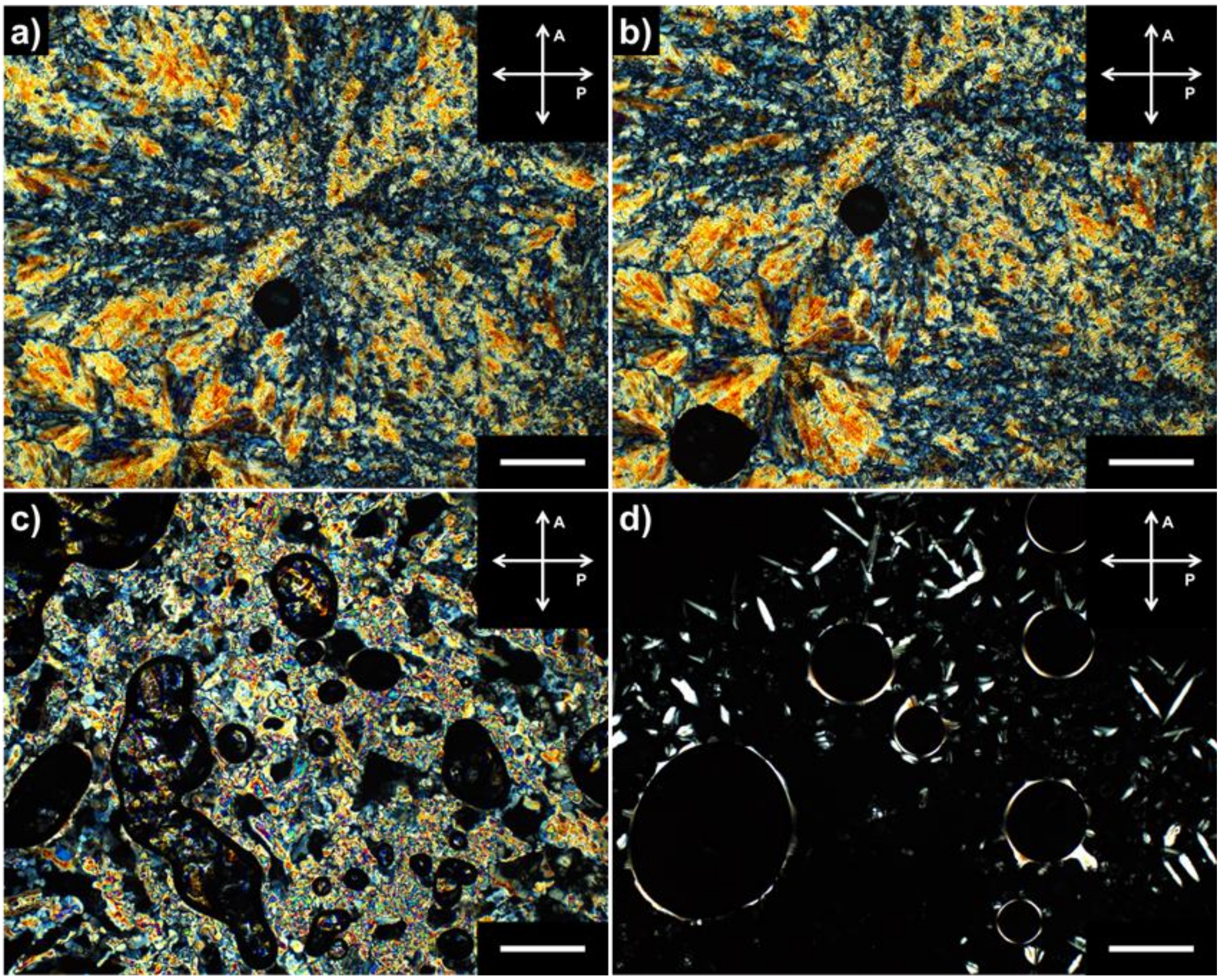

Figure S3. Textures obtained on the POM (crossed polarizers, sample sandwiched between two glass slides) of: a) **L-Me$_2$Bi(12)I** (75.0 °C, heating rate: 2 K min^{-1}); b) **L-Me$_2$Bi(12)I** (60.0 °C, cooling rate: 2 K min^{-1}); c) **L-Me$_2$Bi(14) I** (157.1 °C, heating rate: 0.5 K min^{-1}); d) **L-Me$_2$Bi(14) I** (141.0 °C, cooling rate: 0.5 K min^{-1}); The scale bar is 200 μm.

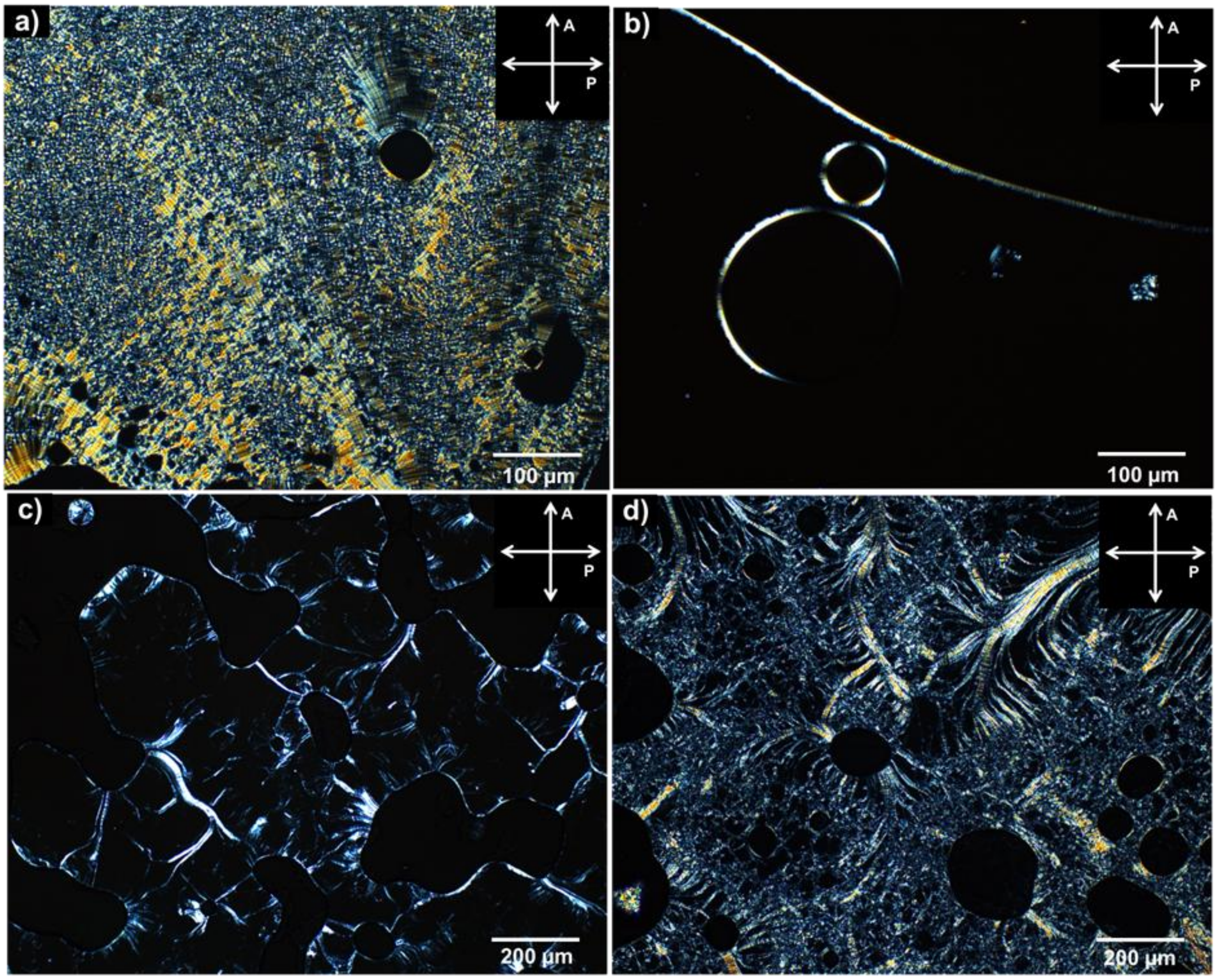

Figure S4. Textures obtained on the POM (crossed polarizers, sample sandwiched between two glass slides) of: a) **U-Me$_2$Bi(5)I** (121.0 °C, cooling rate: 2 K min^{-1}); b) **U-Me$_2$Bi(6)I** (140.6 °C, cooling rate: 2 K min^{-1}); c) **U-Me$_2$Bi(7)I** (147.0 °C, heating rate: 2 K min^{-1}); d) **U-Me$_2$Bi(8)I** (154.0 °C, heating rate: 2 K min^{-1}).

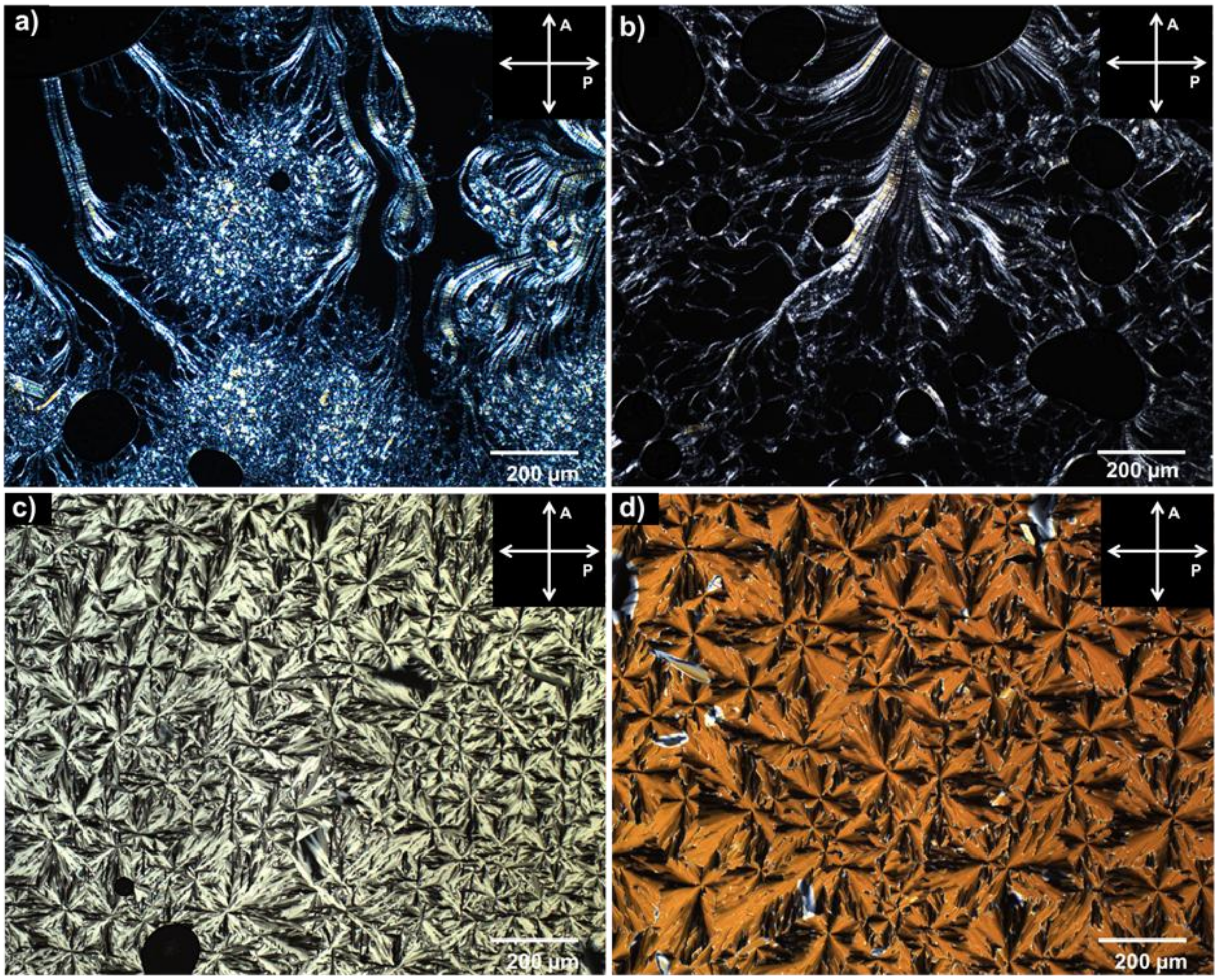

Figure S5. Textures obtained on the POM (crossed polarizers, sample sandwiched between two glass slides) of: a) **U-Me$_2$Bi(9)I** (164.0 °C, heating rate: 2 K min^{-1}); b) **U-Me$_2$Bi(10)I** (141.0 °C, heating rate: 2 K min^{-1}); c) **U-Me$_2$Bi(12)I** (150.0 °C, cooling rate: 2 K min^{-1}); d) **U-Me$_2$Bi(14)I** (170.0 °C, cooling rate: 2 K min^{-1}).

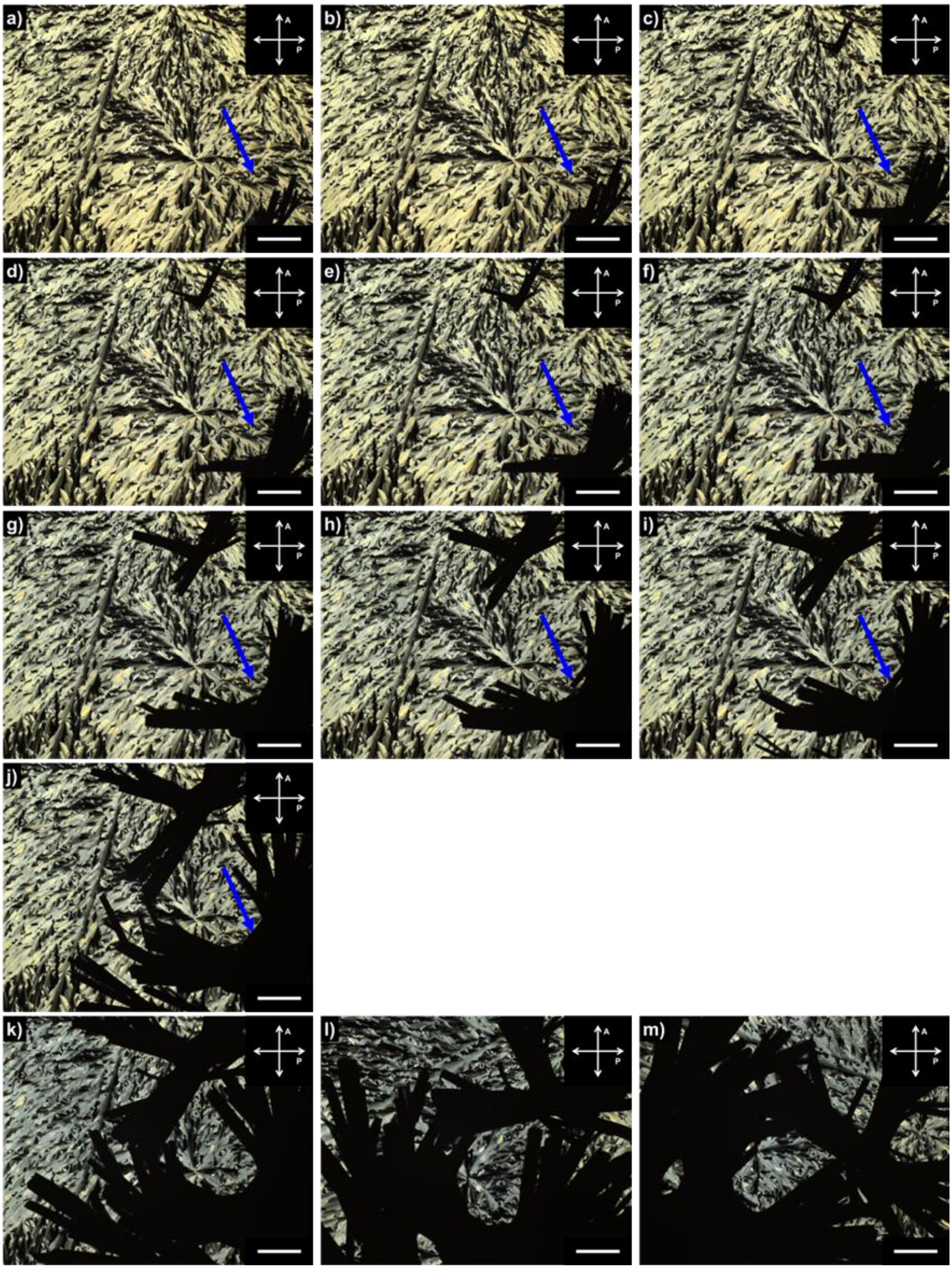

Figure S6. Microscopic pictures of characteristic fan-shaped textures of a smectic phase of **U-Me$_2$Bi(12)I**, all in cooling cycle. The blue arrow serves as orientation for the development of the pseudo-isotropic texture. a) 110.6 °C; b) 109.5 °C; c) 108.0 °C; d) 107.0 °C; e) 106.0 °C; f) 105.0 °C after 10 sec; g) 105.0 °C after 90 sec; h) 105.0 °C after 135 sec; i) 105.0 °C after 165 sec; j) 105.0 °C after 140 sec. The angle-independent pseudo-isotropic texture at 100 °C for k) 0 °; l) 45 ° and 90 ° regarding to initial position of the sample. The scale bar is 200 µm.

3.2. DSC Results

As the different crystalline phases were not investigated in detail within this work and due to several overlapping crystalline phase transitions no numerical assignment of the crystalline phases was made.

Table S1. Phase transition temperatures *T* (onset) in °C (-enthalpies ΔH in $kJ \cdot mol^{-1}$, if available) of linear benzimidazoles **L-Bi(n)**. Cr: Crystalline; N: nematic; I: isotropic liquid; • observed in DSC; – not observed. Values from DSC with cooling rates of $2\ K \cdot min^{-1}$, heating rates of $5\ K \cdot min^{-1}$.

n	Cr			Cr			N			I	
5	•	115	(18.6)	–			–			•	2nd H
	•	110	(–18.1)	–			–			•	1st C
6	•	113	(27.2)	–			–			•	2nd H
	•	99	(–24.2)	–			–			•	2nd C
7	•[a]	–5	–	•	85	(13.7)	–			•	2nd H
	•	63	(–3.7)	•	66	(–7.9)	–			•	2nd C
8	•	94	(26.0)	–			–			•	2nd H
	•	85	(–24.4)	–			–			•	1st C
9	•	67	(18.7)	–			–			•	2nd H
	•	55	(–10.9)	–			•	(59	(–2.4))	•	1st C
10	•	43	(–1.8)	•	63	(30.5)	–			•	2nd H
	•	41	(–16.1)	–			•	(58	(–2.6))	•	1st C
12	•	53	(–1.0)	•	59	(36.6)	–			•	2nd H
	•	42	(–21.5)	–			•	(51	(–2.2))	•	1st C
14	•	26	(–1.4)	•	54	(32.4)	–			•	2nd H
	•	38	(–16.0)	–			•	(48	(–1.9))	•	1st C

[a]: glass

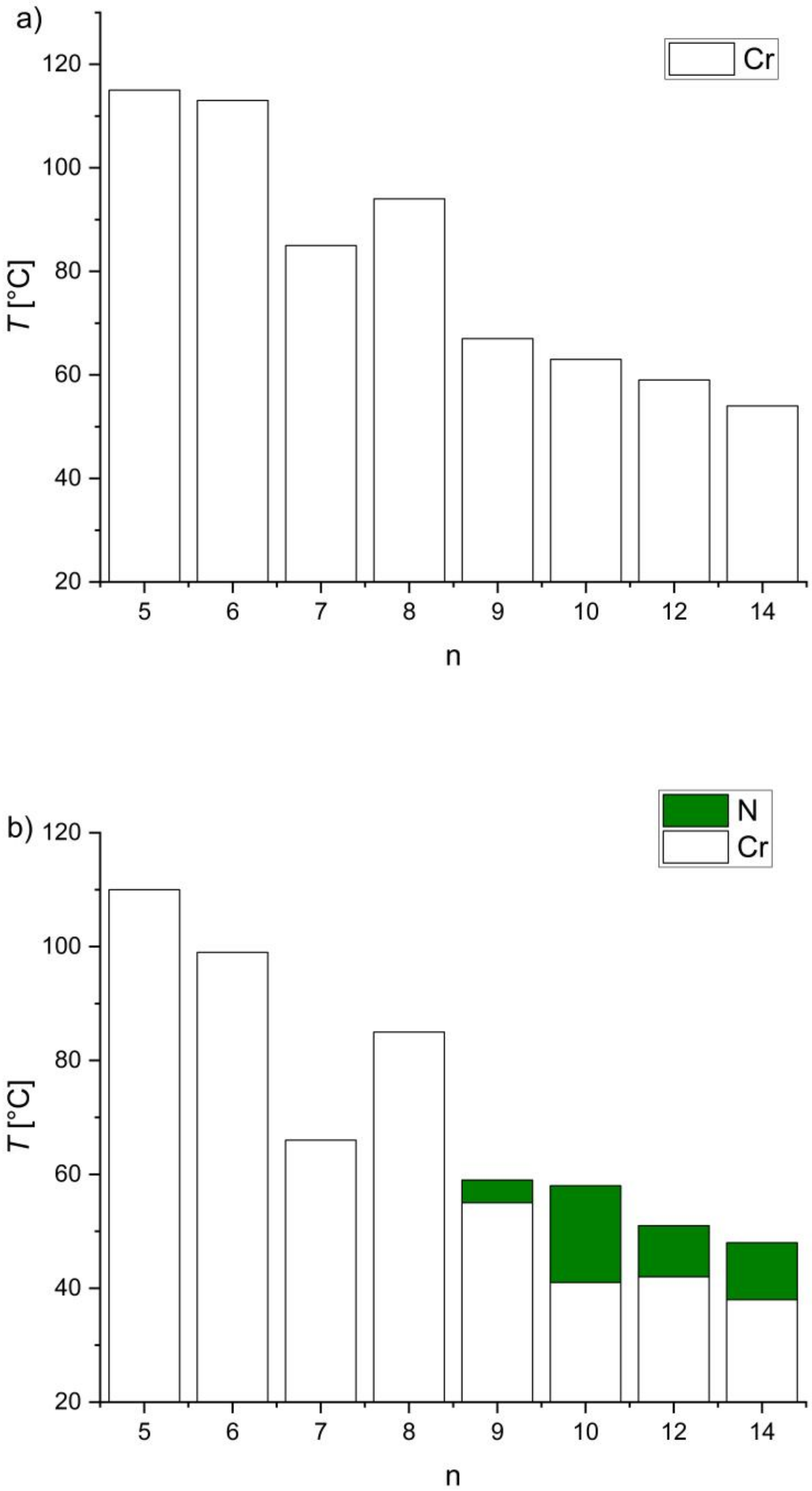

Figure S7. Phase ranges of **L-Bi(n)**. a) Transition temperatures during heating cycle for crystalline to isotropic phase according to Table S1; b) Transition temperatures during cooling cycle for isotropic to crystalline phases according to Table S1.

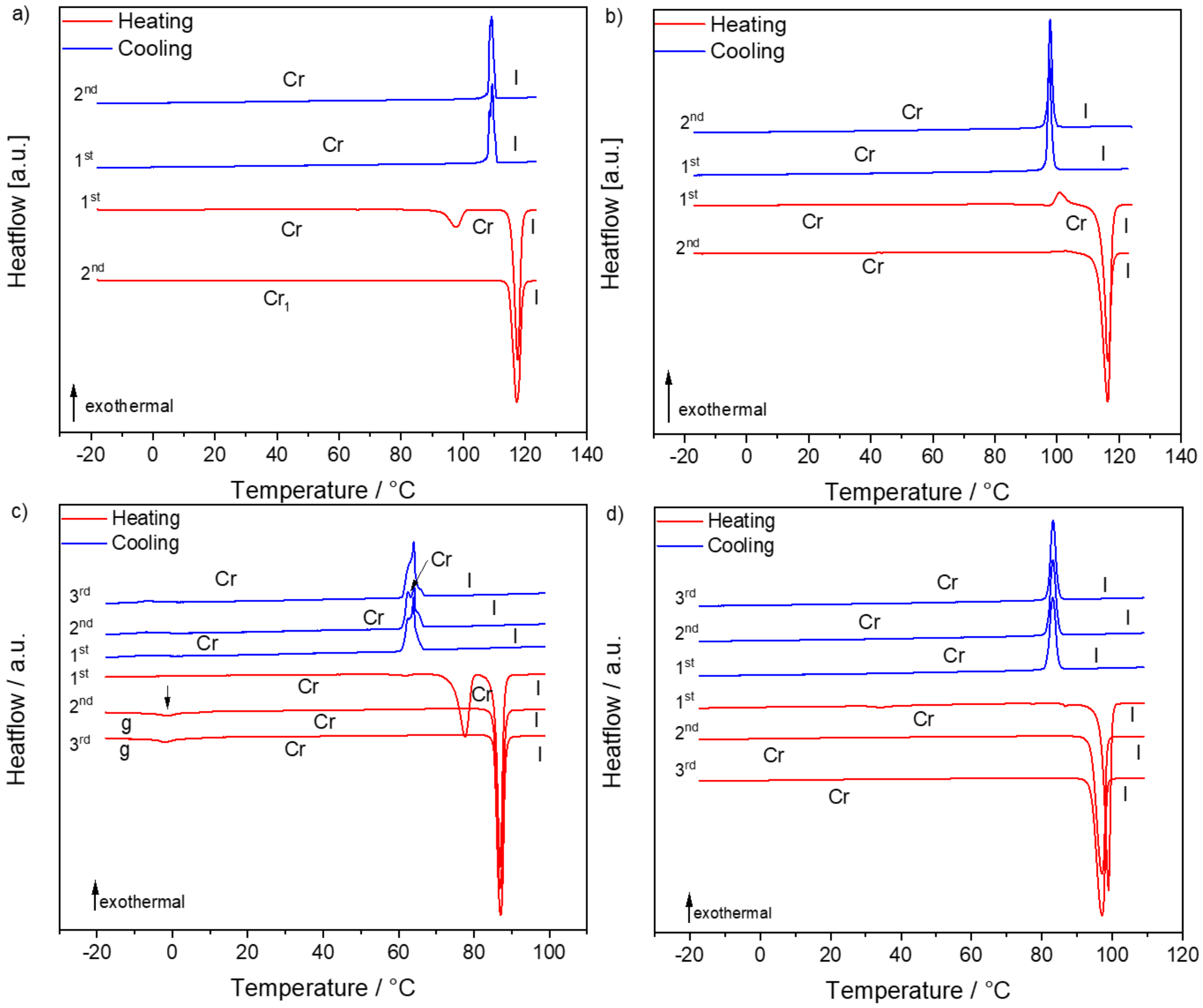

Figure S8. DSC curves of a) **L-Bi(5)**, b) **L-Bi(6)**, c) **L-Bi(7)**, d) **L-Bi(8)**, Cr: crystalline; g: glass; N: nematic; I: isotropic; Cooling rates of 2 K · min^{-1}, heating rates of 5 K · min^{-1}.

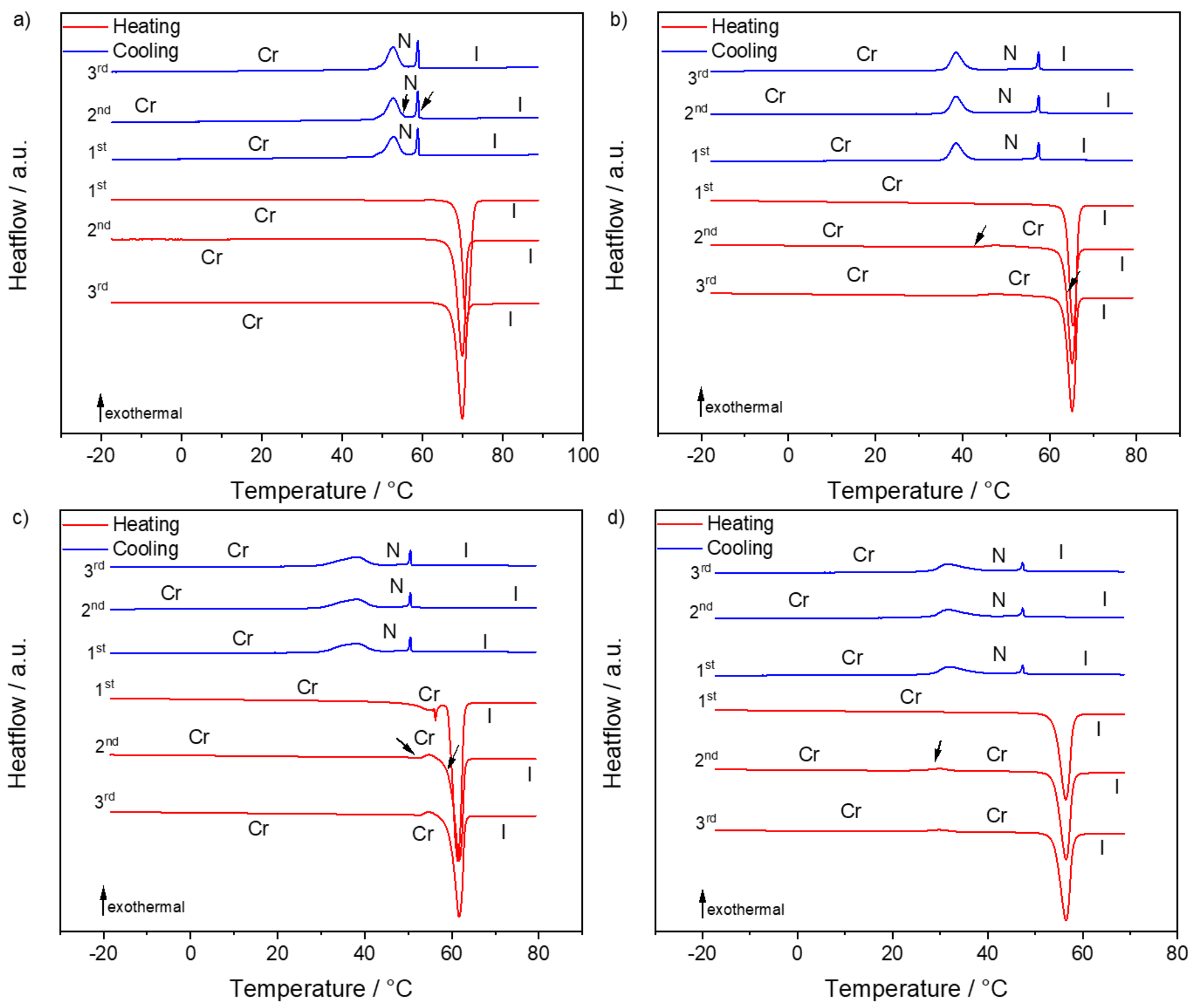

Figure S9. DSC curves of a) **L-Bi(9)**, b) **L-Bi(10)**, c) **L-Bi(12)**, d) **L-Bi(14)**, Cr: crystalline; N: nematic; I: isotropic; Cooling rates of 2 K · min^{-1}, heating rates of 5 K · min^{-1}.

Table S2. Phase transition temperatures *T* (onset) in °C (-enthalpies Δ*H* in kJ · mol^{-1}, if available) of U-shaped benzimidazoles **U-Bi(n)**. Cr: Crystalline; I: isotropic liquid; • observed in DSC; – not observed. Values from DSC with cooling rates of 2 K · min^{-1}, heating rates of 5 K · min^{-1}.

n	Cr			Cr			Cr			I	
5	•	98	(24.0)	–			–			•	1st H
	•	86	(–24.0)	–			–			•	1st C
6	•	101	(21.5)	–			–			•	1st H
	•	69	(–0.3)	•	90	(–9.9)	•	94	(–11.5)	•	1st C
7	•	101	(43.8)	–			–			•	1st H
	•	89.6	(–12.1)	•	89.8	(–22.7)	•	92	(–9.3)	•	1st C
8	•	104	(38.3)	–			–			•	1st H
	•	96	(–27.7)	•	99	(–9.9)	–			•	1st C
9	•	104	(36.8)	–			–			•	2nd H
	•	80	(–0.5)	•	90	(–29.7)	•	96	(–7.6)	•	1st C
10	•	85	(11.1)	•	106	(43.8)	–			•	1st H
	•	35	(–11.8)	•	95	(–36.6)	•	97	(–7.1)	•	1st C
12	•	71	(9.8)	•	93	(4.4)	•	107	(48.3)	•	2nd H
	•	56	(–17.5)	•	94	(–48.9)	–			•	1st C
14	•	79	(12.4)	•	96	(8.5)	•	106	(51.1)	•	2nd H
	•	70	(–20.5)	•	97	(–54.4)	–			•	1st C

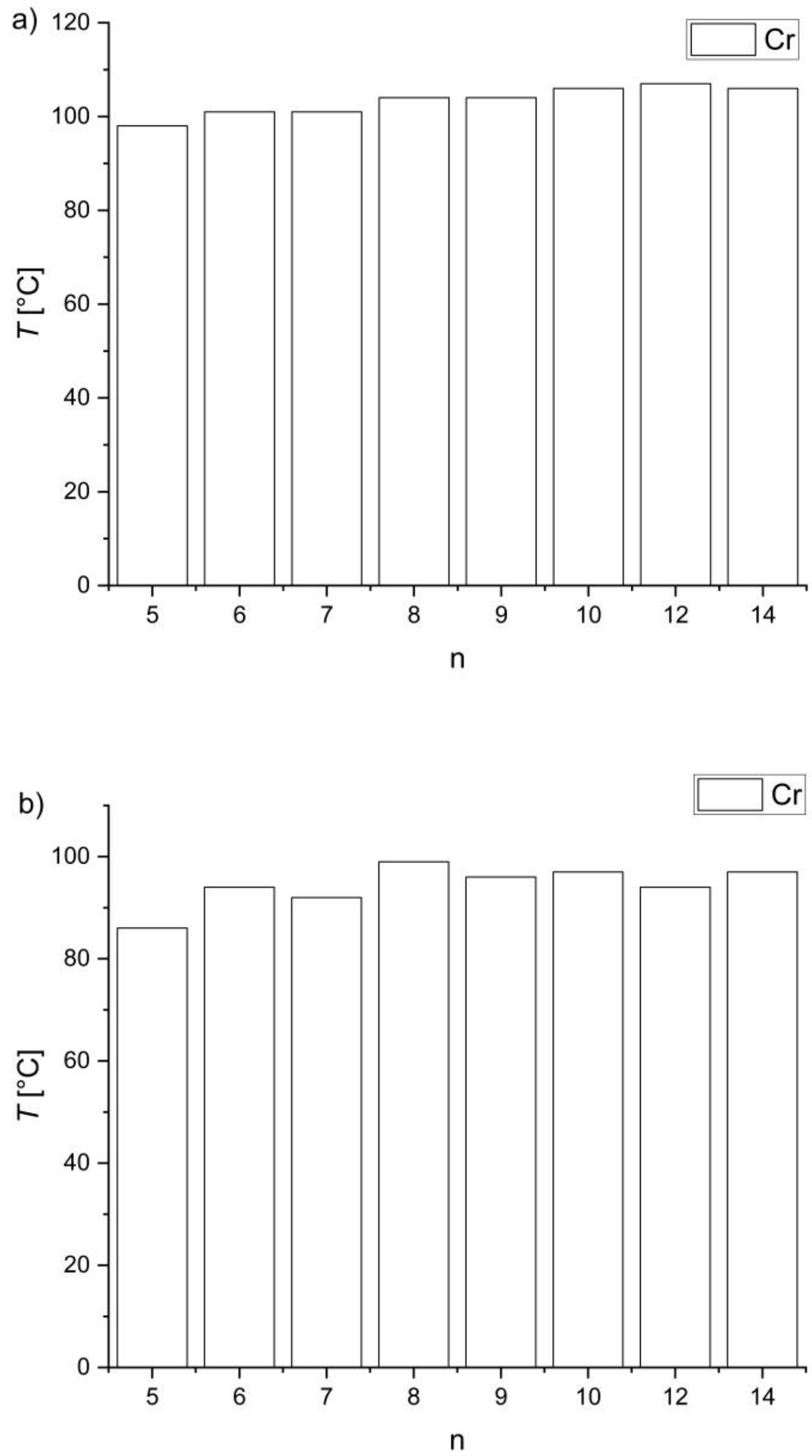

Figure S10. Phase ranges of **U-Bi(n)**. a) Transition temperatures during heating cycle for crystalline to isotropic phase according to Table S2; b) Transition temperatures during cooling cycle for isotropic to crystalline phases according to Table S2.

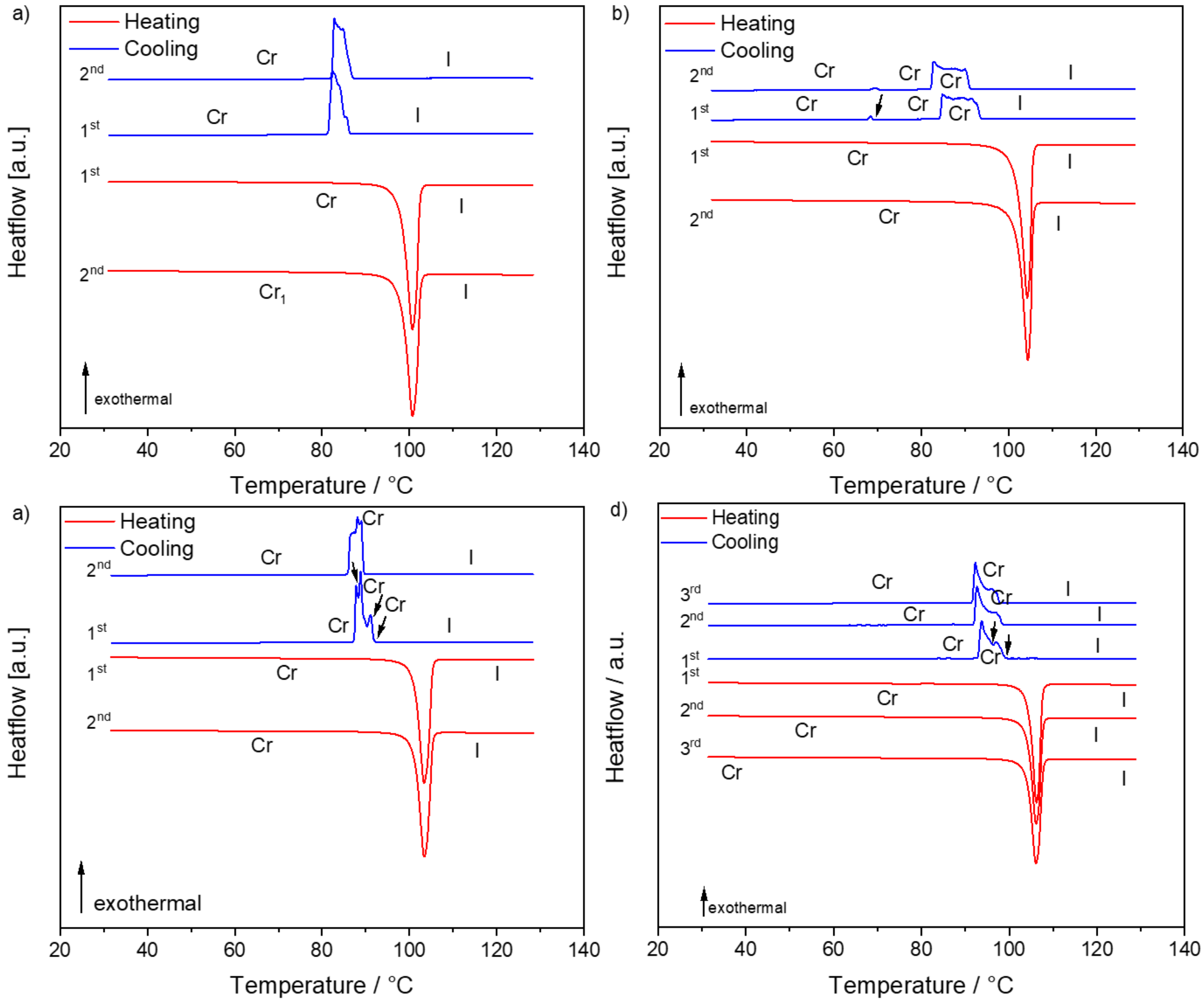

Figure S11. DSC curves of a) **U-Bi(5)**, b) **U-Bi(6)**, c) **U-Bi(7)**, d) **U-Bi(8)**, Cr: crystalline; I: isotropic. Cooling rates of 2 K · min^{-1}, heating rates of 5 K · min^{-1}.

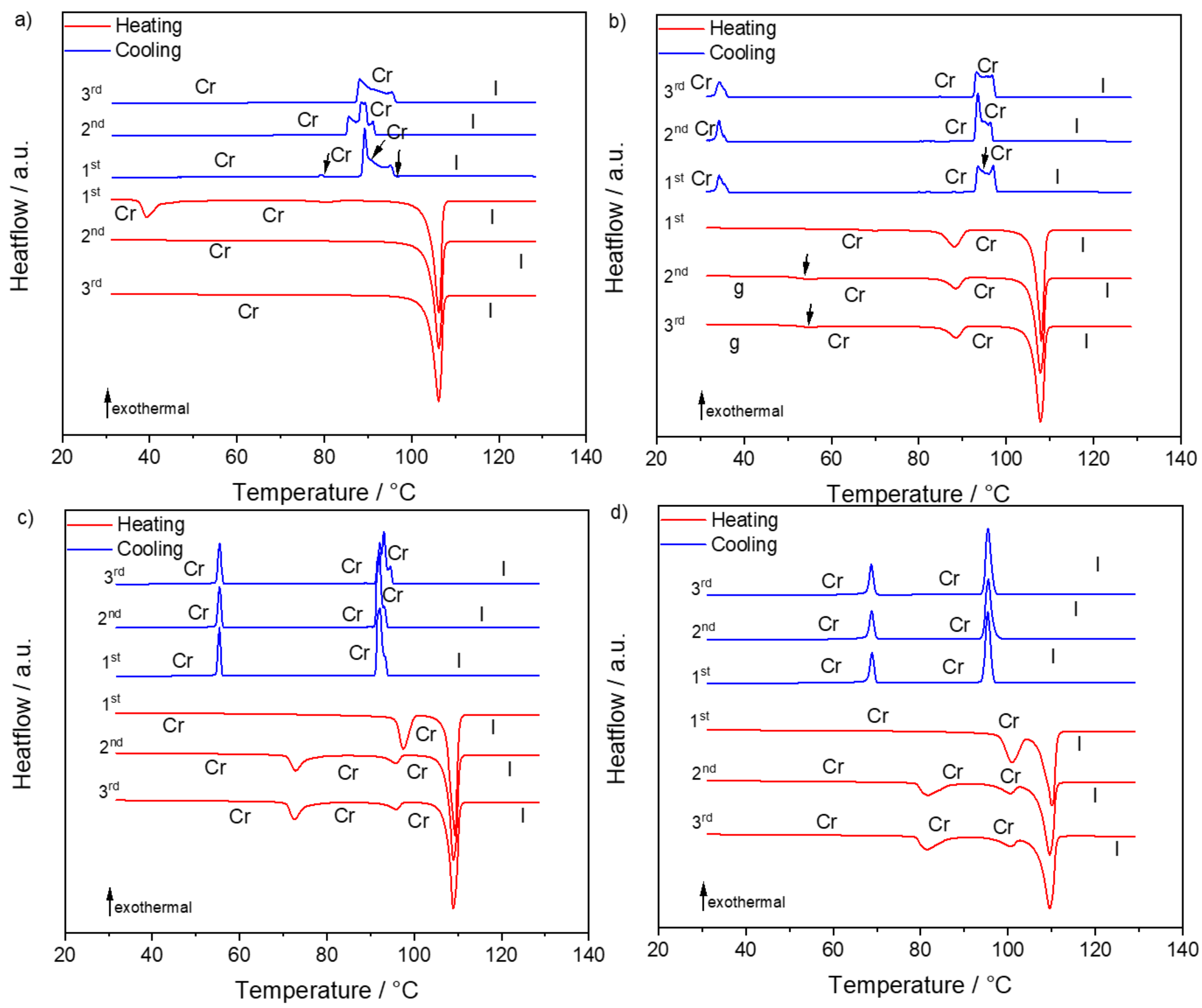

Figure S12. DSC curves of a) **U-Bi(9)**, b) **U-Bi(10)**, c) **U-Bi(12)**, d) **U-Bi(14)**, Cr: crystalline; I: isotropic. Cooling rates of 2 K · min^{-1}, heating rates of 5 K · min^{-1}.

Table S3. Phase transition temperatures T (onset) in °C (-enthalpies ΔH in $kJ \cdot mol^{-1}$, if available) of linear benzimidazole iodides **L-Me₂Bi(n)I**. Cr: Crystalline; I: isotropic liquid; • observed in DSC; – not observed. Values from DSC with cooling rate of $2\ K \cdot min^{-1}$ and heating rate of $5\ K \cdot min^{-1}$.

n	Cr			Cr			Cr			Cr			I	
5	•	167	(34.2)	–			–			–			•	1st H
	•	138	(–25.6)	–			–			–			•	1st C
6	•	160	(32.6)	–			–			–			•	1st H
	•	130	(–24.3)	–			–			–			•	1st C
7	•	167	(31.1)	–			–			–			•	1st H
	•	139	(–18.3)	–			–			–			•	1st C
8	•	36	(1.6)	•	56	(0.7)	•	148	(15.9)	•	172	(20.2)	•	1st H
	•	112	(–8.4)	•	159	(–7.0)	–			–			•	1st C
9	•	140	(20.2)	•	182	(24.0)	–			–			•	1st H
	•	75	(–7.0)	•	154	(–4.3)	–			–			•	1st C
10	•	136	(19.7)	•	174	(19.2)	–			–			•	1st H
	•	95	(–10.2)	•	162	(–6.6)	–			–			•	1st C
12	•	148	(29.9)	•	164	(21.1)	–			–			•	1st H
	•	139	(–22.3)	•	155	(–8.8)	–			–			•	1st C
14	•	142	(30.3)	•	158	(14.8)	•	162	(4.5)	–			•	1st H
	•	128	(–20.7)	•	150	(–5.9)	–			–			•	1st C

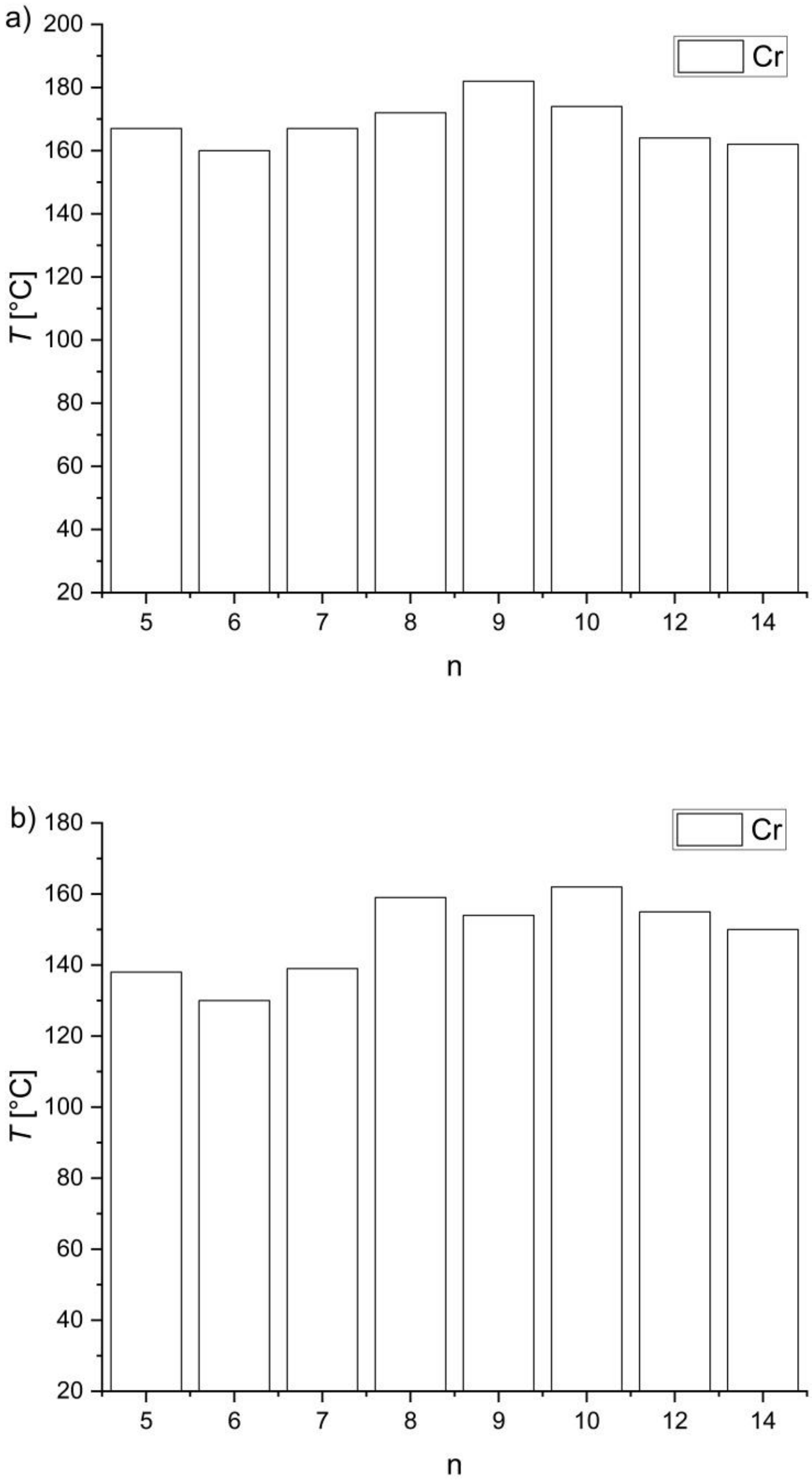

Figure S13. Phase ranges of **L-Me$_2$Bi(n)I**. a) Transition temperatures during heating cycle for crystalline to isotropic phase according to Table S3; b) Transition temperatures during cooling cycle for isotropic to crystalline phases according to Table S3.

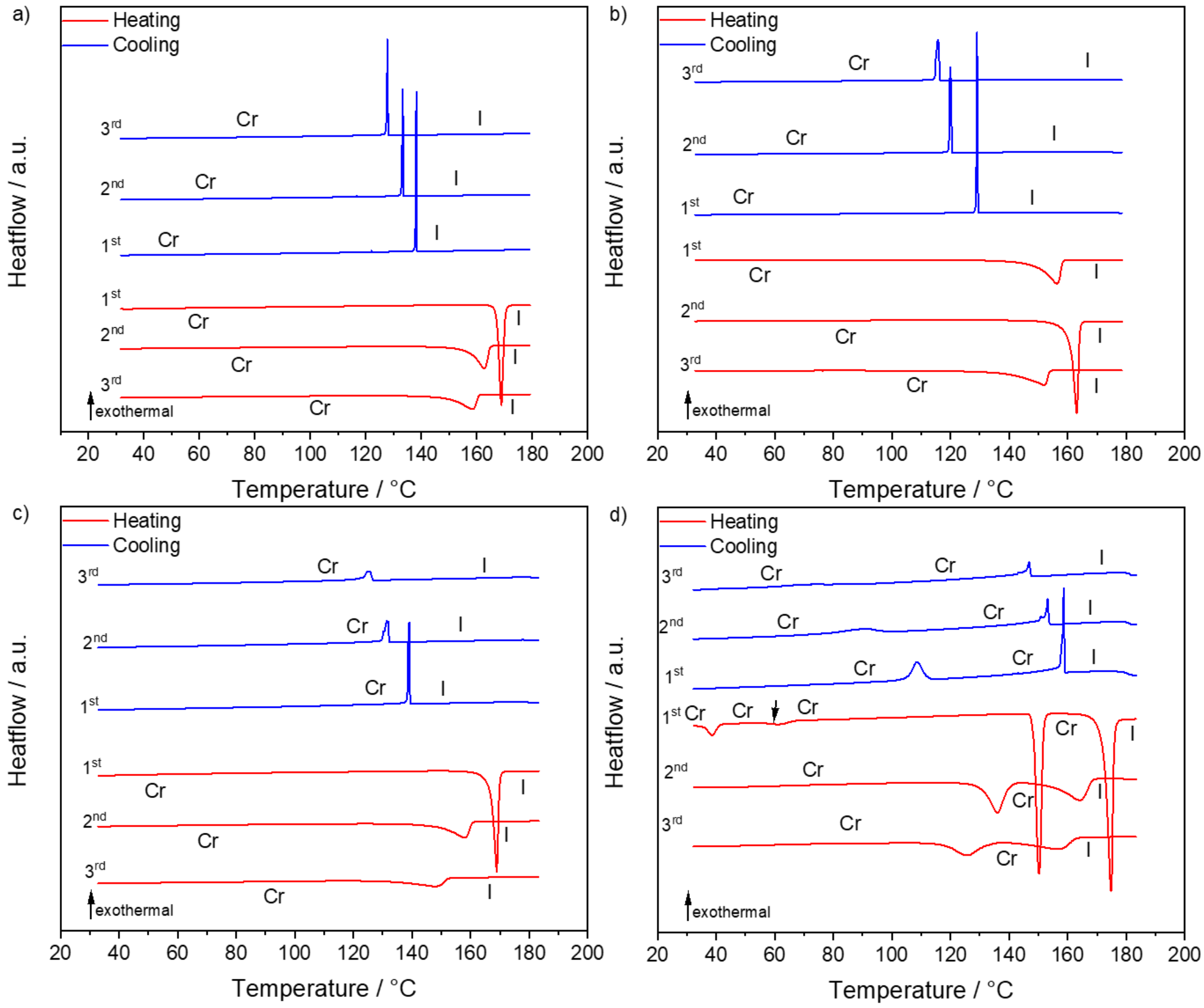

Figure S14. DSC curves of a) **L-Me$_2$Bi(5)I**, b) **L-Me$_2$Bi(6)I**, c) **L-Me$_2$Bi(7)I**, d) **L-Me$_2$Bi(8)I**, Cr: crystalline; I: isotropic.; Cooling rate of 2 K · min^{-1} and heating rate of 5 K · min^{-1}.

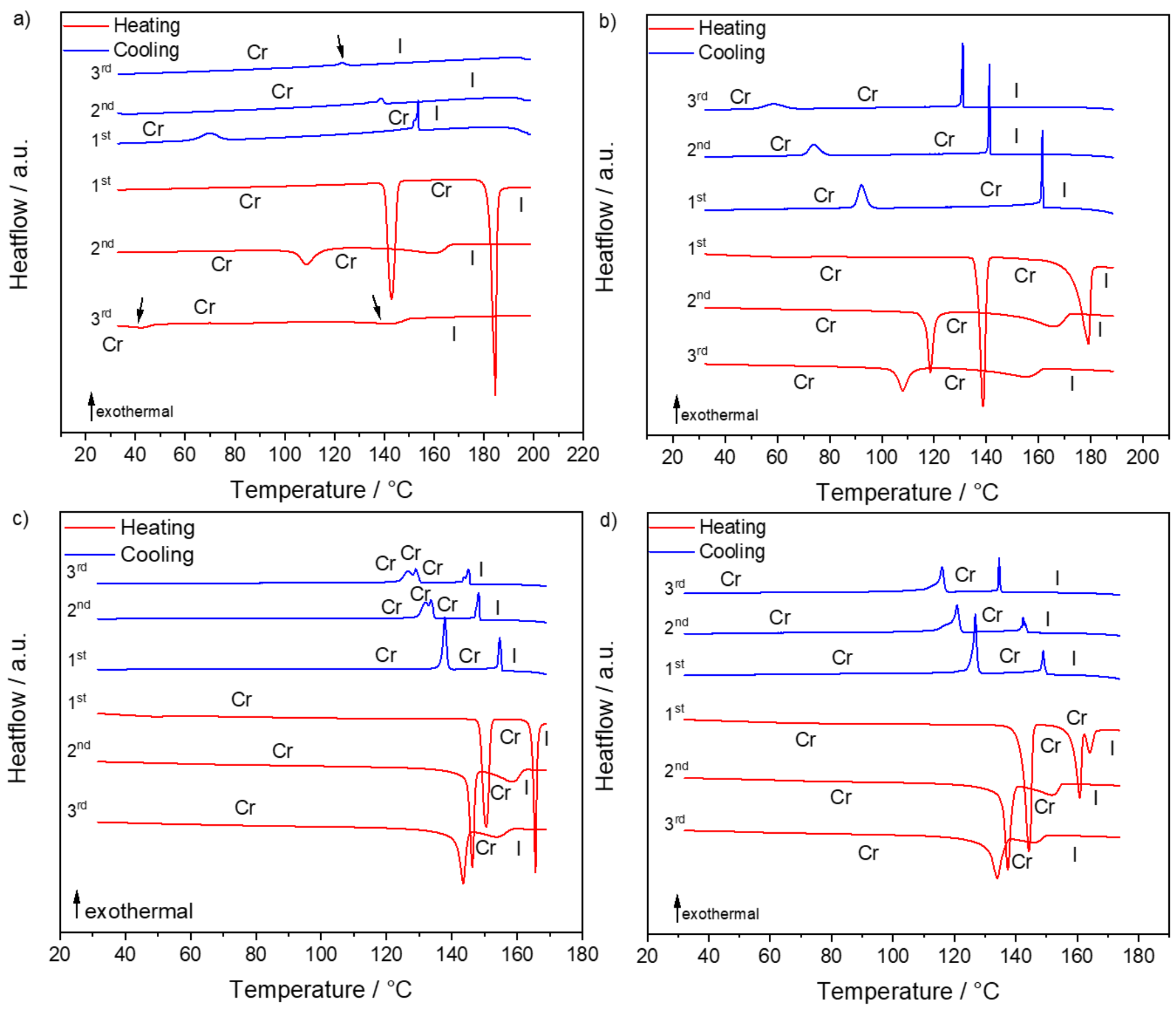

Figure S15. DSC curves of a) **L-Me$_2$Bi(9)I**, b) **L-Me$_2$Bi(10)I**, c) **L-Me$_2$Bi(12)I**, d) **L-Me$_2$Bi(14)I**, Cr: crystalline; Sm: semectic; I: isotropic; cooling rate of 2 $K\cdot min^{-1}$ and heating rate of 5 $K\cdot min^{-1}$.

Table S4. Phase transition temperatures *T* (onset) in °C (-enthalpies ΔH in kJ · mol^{-1}, if available) of U-shaped benzimidazole iodides **U-Me$_2$Bi(n)I**. Cr: Crystalline; Sm: smectic; Cub: cubic; I: isotropic liquid; • observed in DSC; – not observed. Values from DSC with cooling rate of 2 K · min^{-1} and heating rate of 5 K · min^{-1}.

n	Cr		Cr		Cr		Cr		Cr		Cr		Cub		Sm		I
5	•	47 (0.8)	•	72 (0.5)	•	96 (0.8)	•	153 (19.9)	–		–		–		–		• 1st H
	•	100 (–6.7)	•	102 (–4.8)	–		–		–		–		–		•[a]	(121 (–1.9))	• 1st C
6	•	149 (21.7)	–		–		–		–		–		–		–		• 1st H
	•	99 (–17.6)	–		–		–		–		–		–		•[a]	(139 (–1.4))	• 1st C
7	•	143 (19.9)	–		–		–		–		–		–		•[a]	156 (1.2)	• 2nd H
	•	112 (–7.9)	•	113 (–11.2)	–		–		–		–		–		•[a]	156 (–1.2)	• 1st C
8	•	–3 (3.7)	•	39 (3.7)	•	140 (16.3)	–		–		–		–		•[a]	161 (0.8)	• 2nd H
	•	–4 (–1.1)	•	23 (–2.7)	•	118 (–15.4)	–		–		–		–		•[a]	162 (–0.9)	• 1st C
9	•	140 (16.8)	–		–		–		–		–		–		•[a]	164 (0.7)	• 2nd H
	•	117 (–16.1)	–		–		–		–		–		–		•[a]	165 (–0.7)	• 1st C
10	•	136 (14.3)	–		–		–		–		–		–		•[a]	162 (0.5)	• 2nd H
	•	118 (–15.1)	–		–		–		–		–		–		•[a]	163 (–0.5)	• 1st C
12	•	34 (0.2)	•	47 (0.2)	•	56 (–1.9)	•	77 (1.2)	•	124 (10.8)	–		•	130 (0.3)	•[a]	181 (0.5)	• 2nd H
	•	96 (–7.3)	•	102 (–3.5)	–		–		–		–		•	131 (−0.6)	•[a]	182 (–0.5)	• 1st C
14	•	21 (1.3)	•	48 (2.7)	•	57 (–8.0)	•	85 (1.9)	•	100 (11.5)	•	122 (–5.0)	–		•[b]	190 (0.6)	• 2nd H
	•	13 (–2.0)	•	58 (–5.0)	•	88 (–9.2)	–		–		–		–		•[b]	193 (–0.7)	• 1st C

[a]: Smectic A phase observed. [b]: Smectic X phase observed.

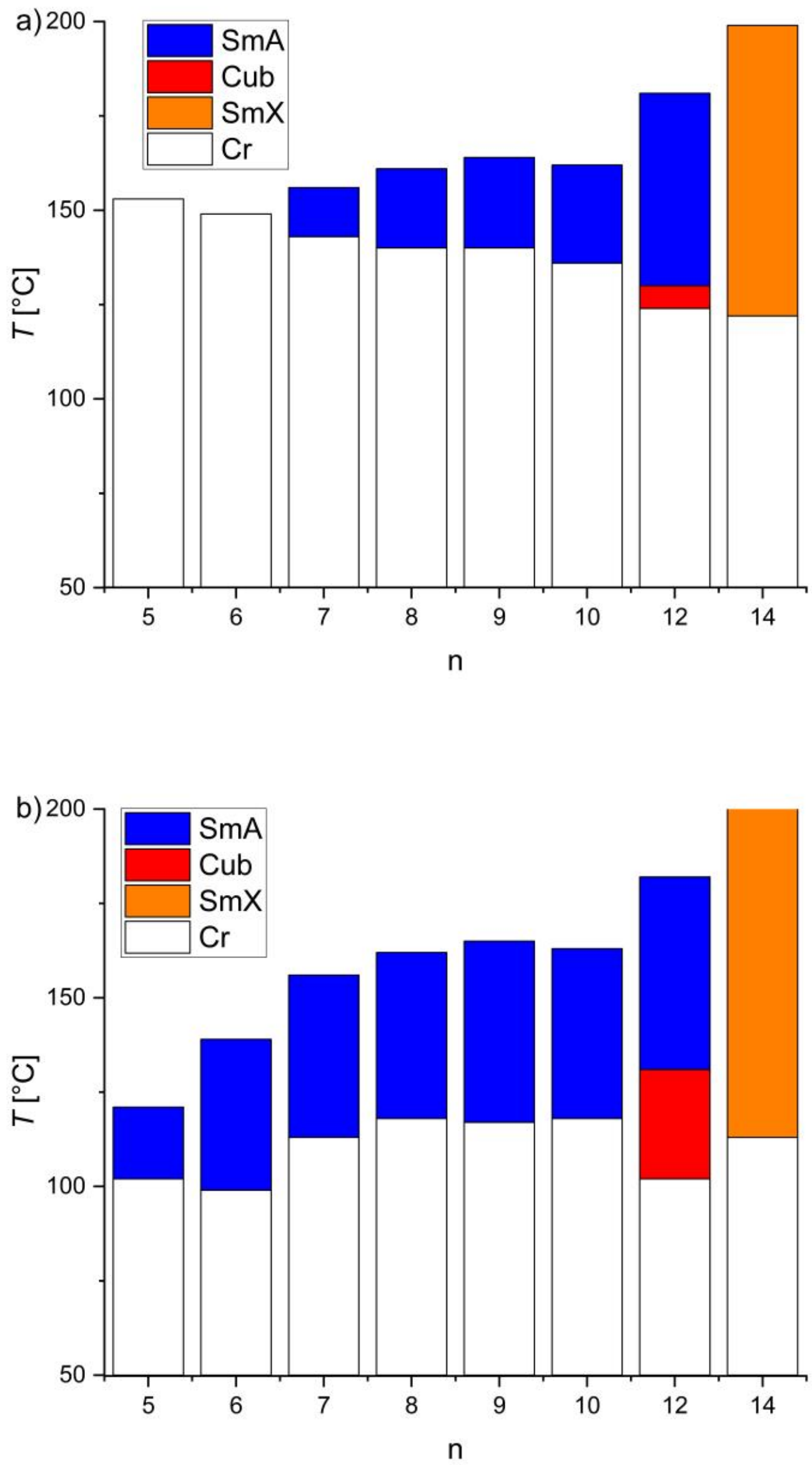

Figure S16. Phase ranges of **U-Me$_2$Bi(n)I**. a) Transition temperatures during heating cycle for crystalline to isotropic phase according to Table S4; b) Transition temperatures during cooling cycle for isotropic to crystalline phases according to Table S4.

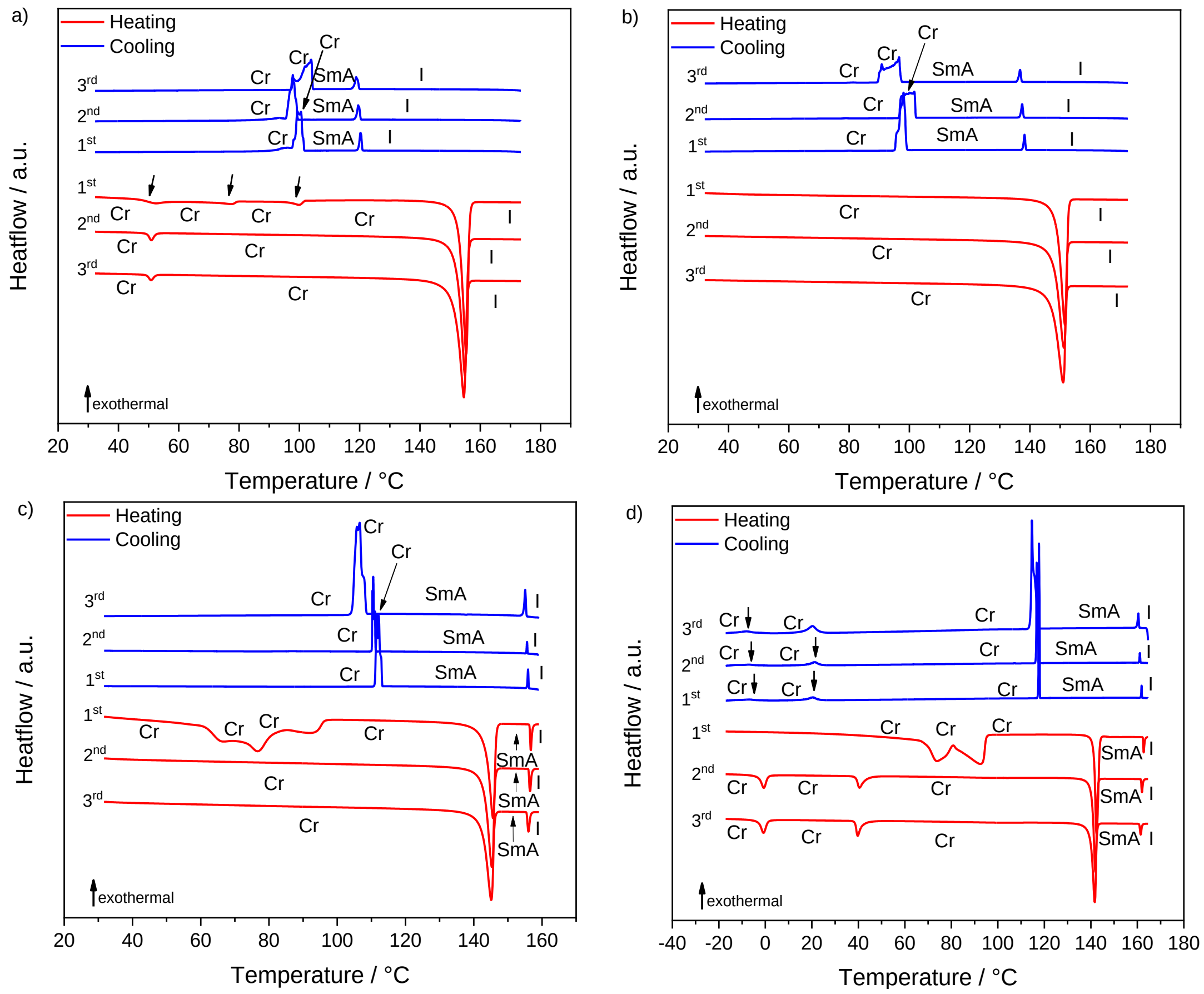

Figure S17. DSC curves of a) **U-Me$_2$Bi(5)I**, b) **U-Me$_2$Bi(6)I**, c) **U-Me$_2$Bi(7)I**, d) **U-Me$_2$Bi(8)I**, Cr: crystalline; Sm: smectic; I: isotropic. Cooling rates of 2 K · min^{-1}, heating rates of 5 K · min^{-1}.

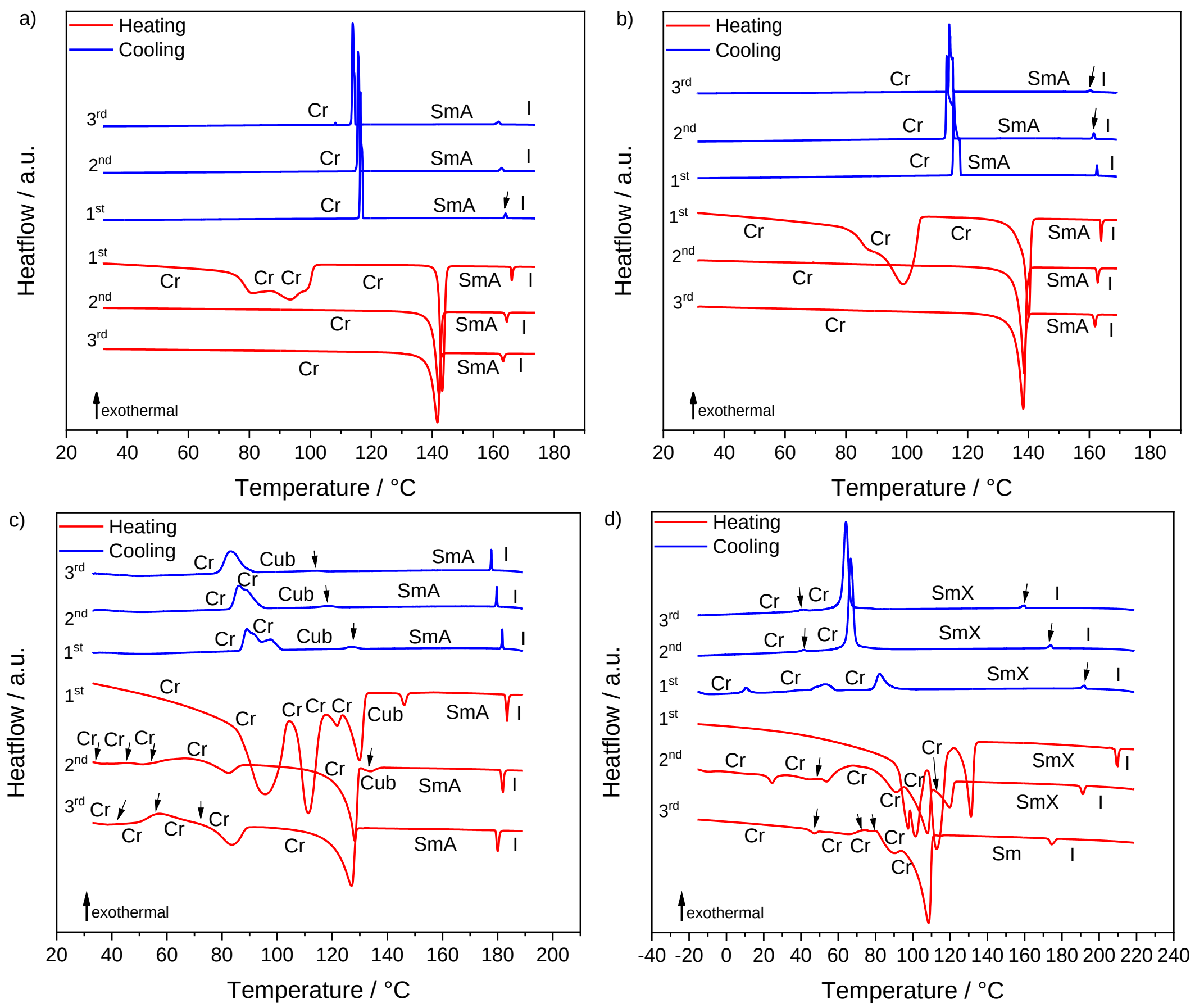

Figure S18. DSC curves of a) **U-Me$_2$Bi(9)I**, b) **U-Me$_2$Bi(10)I**, c) **U-Me$_2$Bi(12)I**, d) **U-Me$_2$Bi(14)I**, Cr: crystalline; Sm: smectic; Cub: cubic; I: isotropic.; Cooling rates of 2 K · min^{-1}, heating rates of 5 K · min^{-1}.

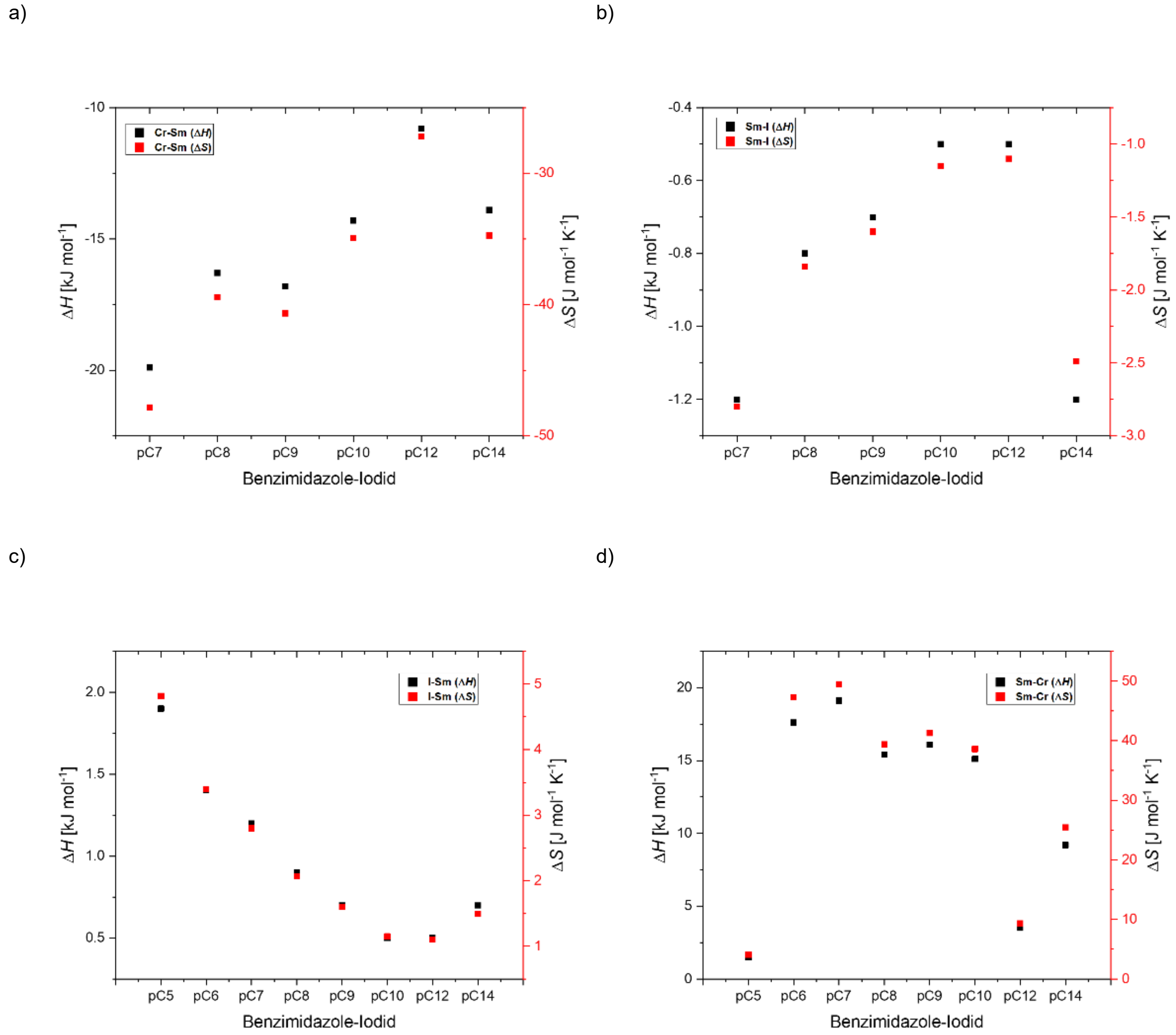

Figure S19. ΔH and ΔS for **U-Me_2Bi(n)I** (n = 7, 8, 9, 10, 12, 14). a) ΔH and ΔS of phase transition Cr-Sm during heating cycles; b) ΔH and ΔS of phase transition Sm-I during cooling cycles; c) ΔH and ΔS of phase transition I-Sm during cooling cycles; d) ΔH and ΔS of phase transition Sm-Cr during cooling cycles. For n = 12 ΔH and ΔS are shown for Cub-Cr.

3.3. XRD Results

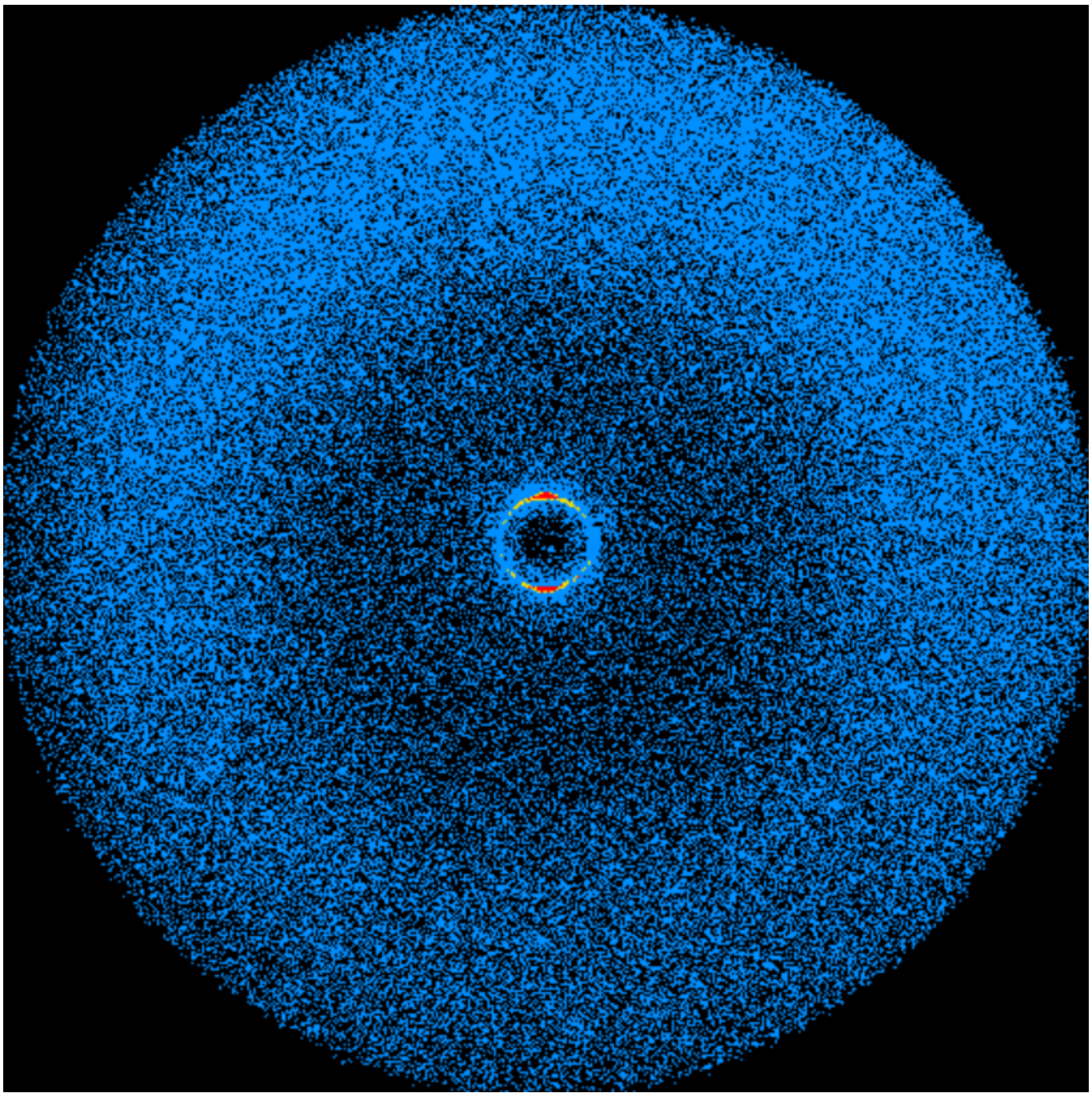

Figure S20. 2D-Xray Diffractogram of **U-Me$_2$Bi(12)I** at 145°C in the SmA phase. A sharp layer peak as well as a diffuse wide angle halo are observed.

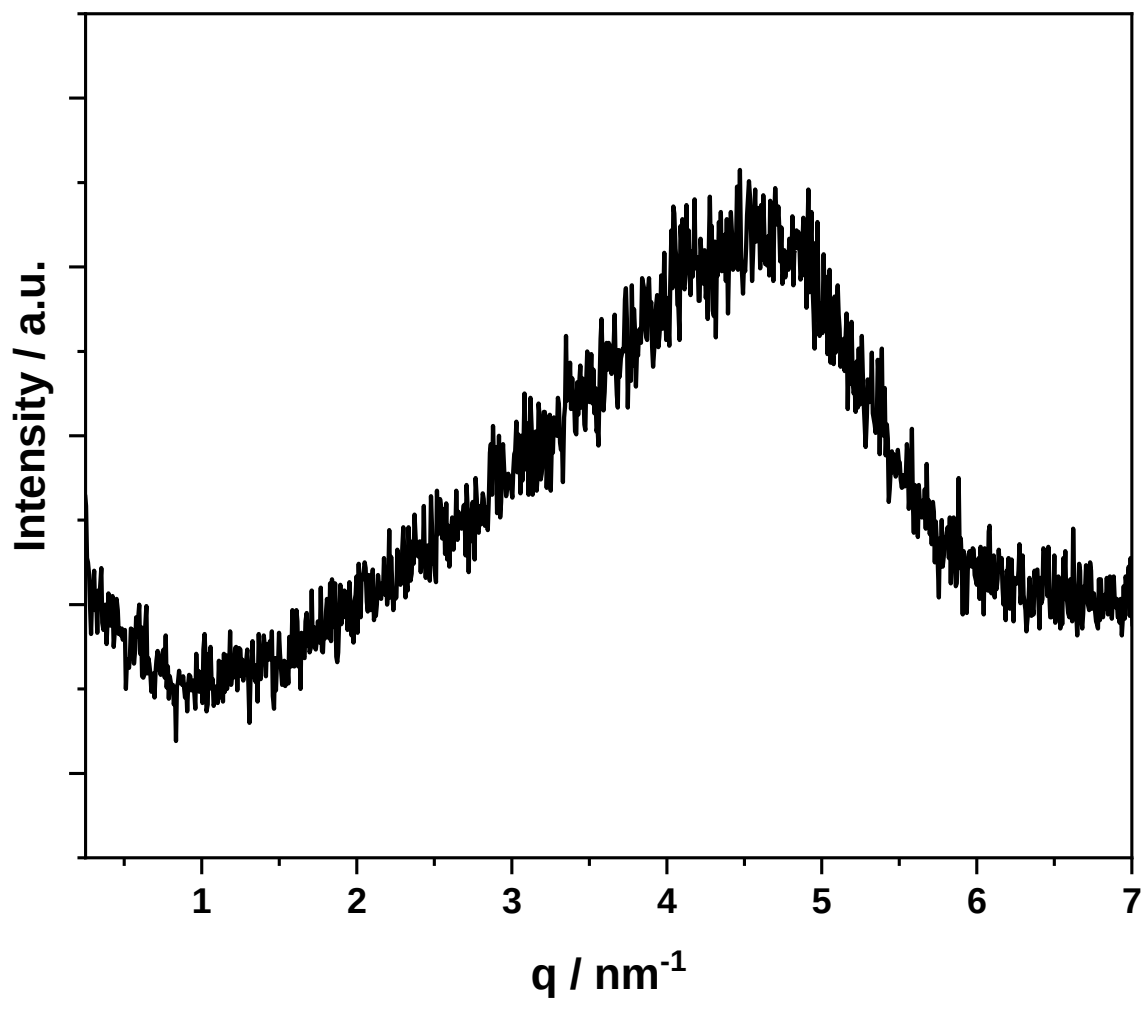

Figure S21. XRD diffractogram of the scattering vector *q* vs. the intensity *I* during cooling cycle in the nematic phase for **L-Bi(10)** at 50 °C.

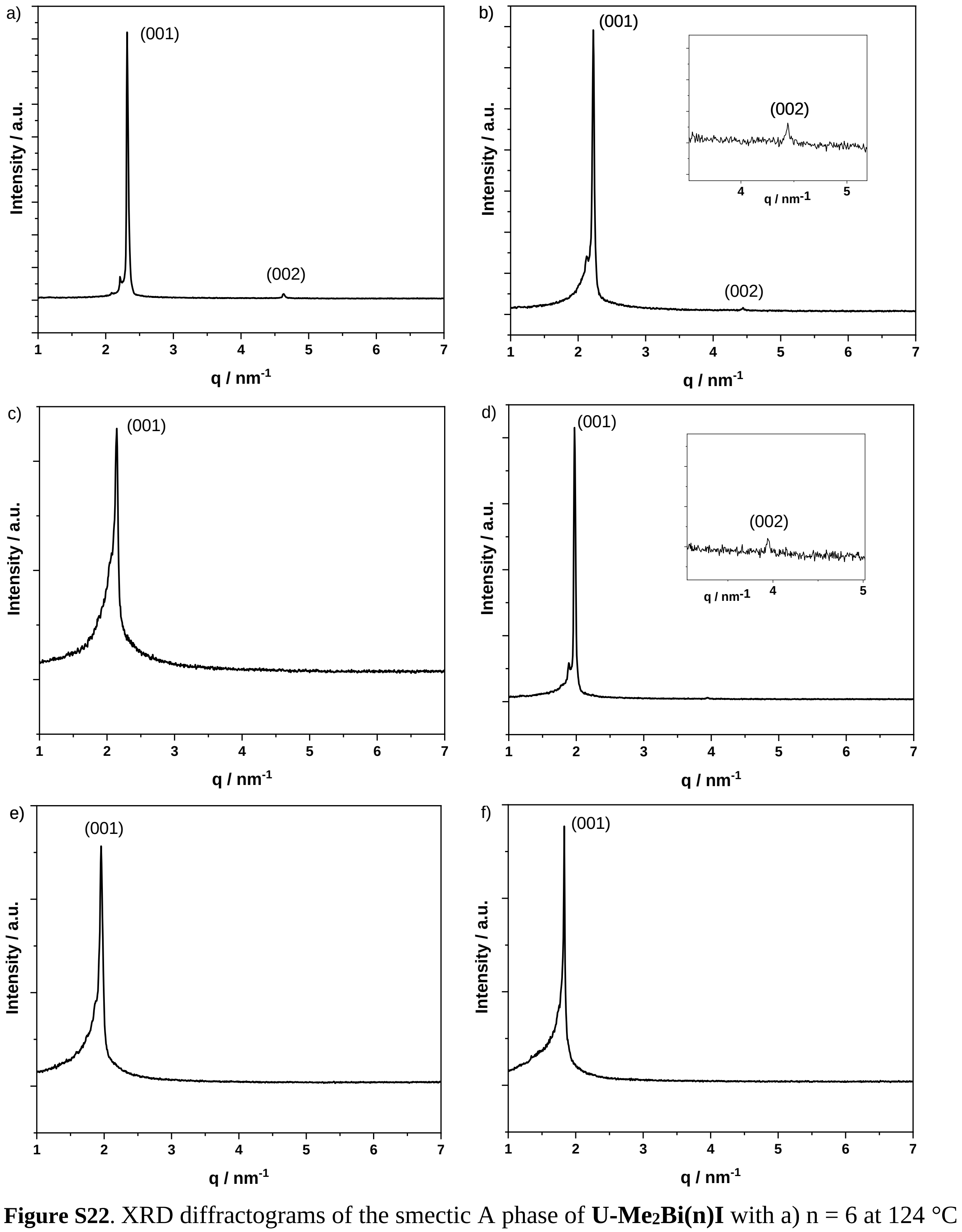

Figure S22. XRD diffractograms of the smectic A phase of **U-Me$_2$Bi(n)I** with a) n = 6 at 124 °C b) n= 7 at 139 °C, c) n = 8 at 153 °C, d) n = 9 at 126 °C, e) n = 10 at 144 °C and f) n = 12 at 135 °C.

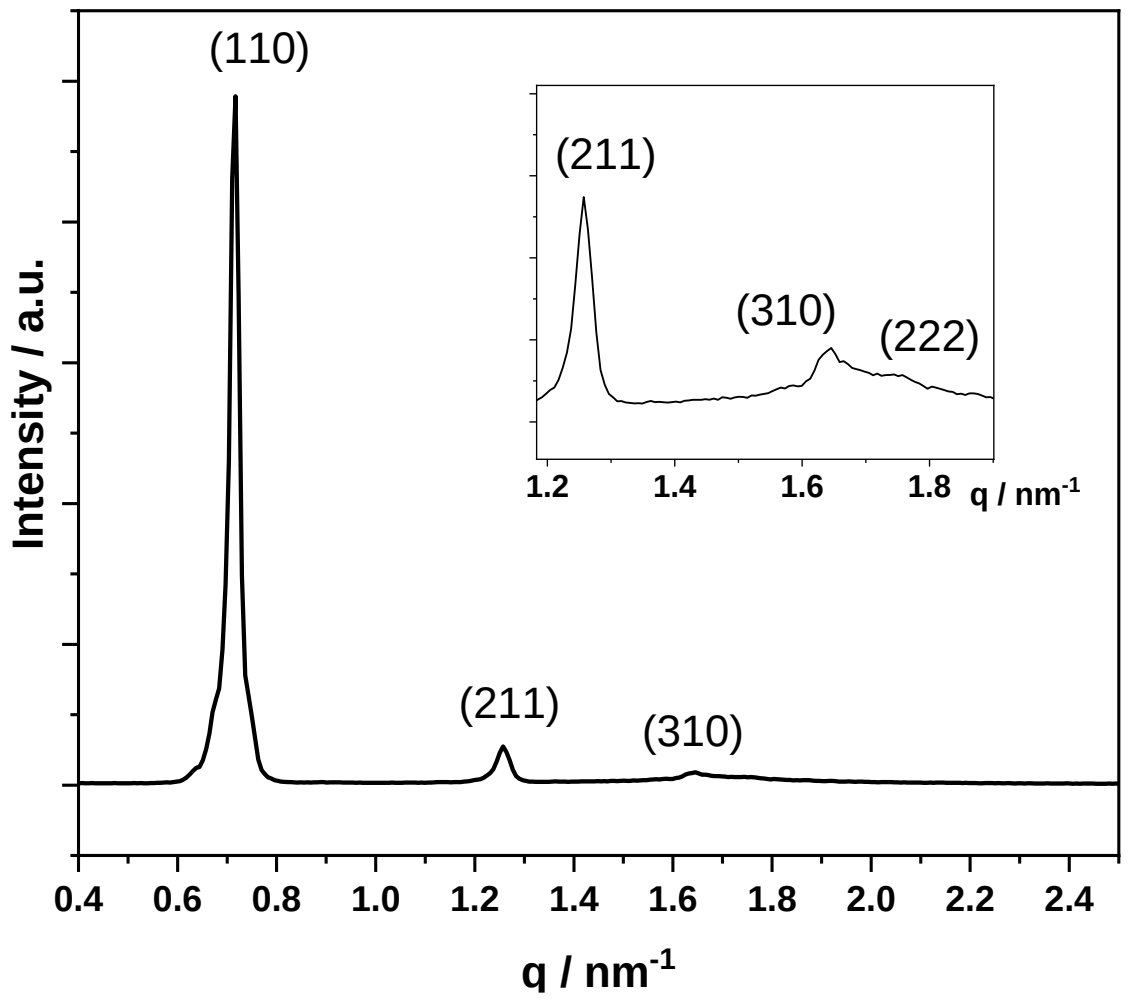

Figure S23. SAXS-diffractograms of the cubic phase of **U-Me$_2$Bi(12)I** at 116 °C. This phase shows the typical peaks of a cubic phase. This leads to a cubic lattice parameter of a = 119.5 Å.

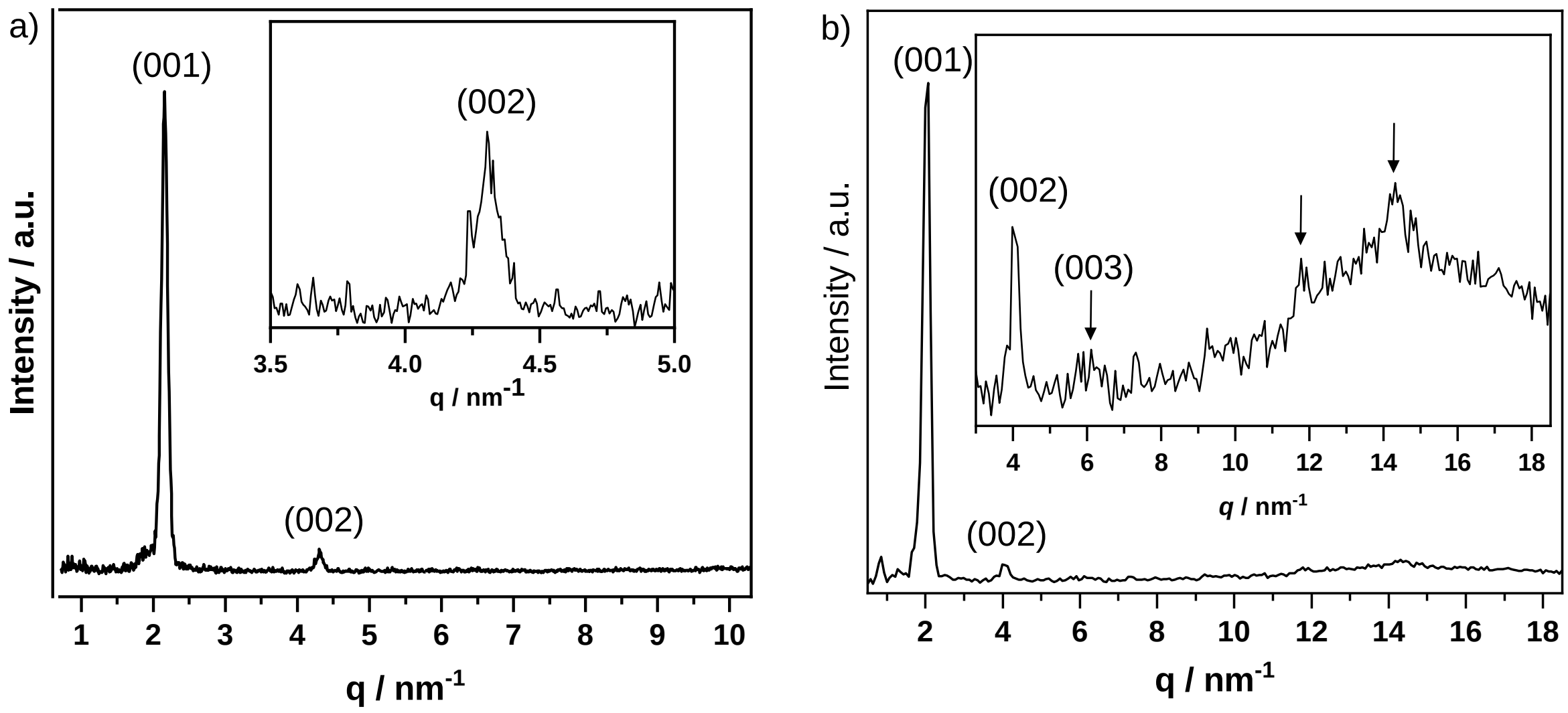

Figure S24. a) SAXS and b) WAXS diffractogram of the SmX phase of **U-Me$_2$Bi(14)I** at 131 °C.

To gain a better understanding of the molecular structure and to determine if double layers are possible, molecular lengths were obtained using Chem3D.[18] For ionic compounds **U-Me$_2$Bi(n)I**, the anion is not considered. Molecular lengths were always determined along the longest possible molecular axis between C atoms furthest from each other. An example can be seen in Figure S25.

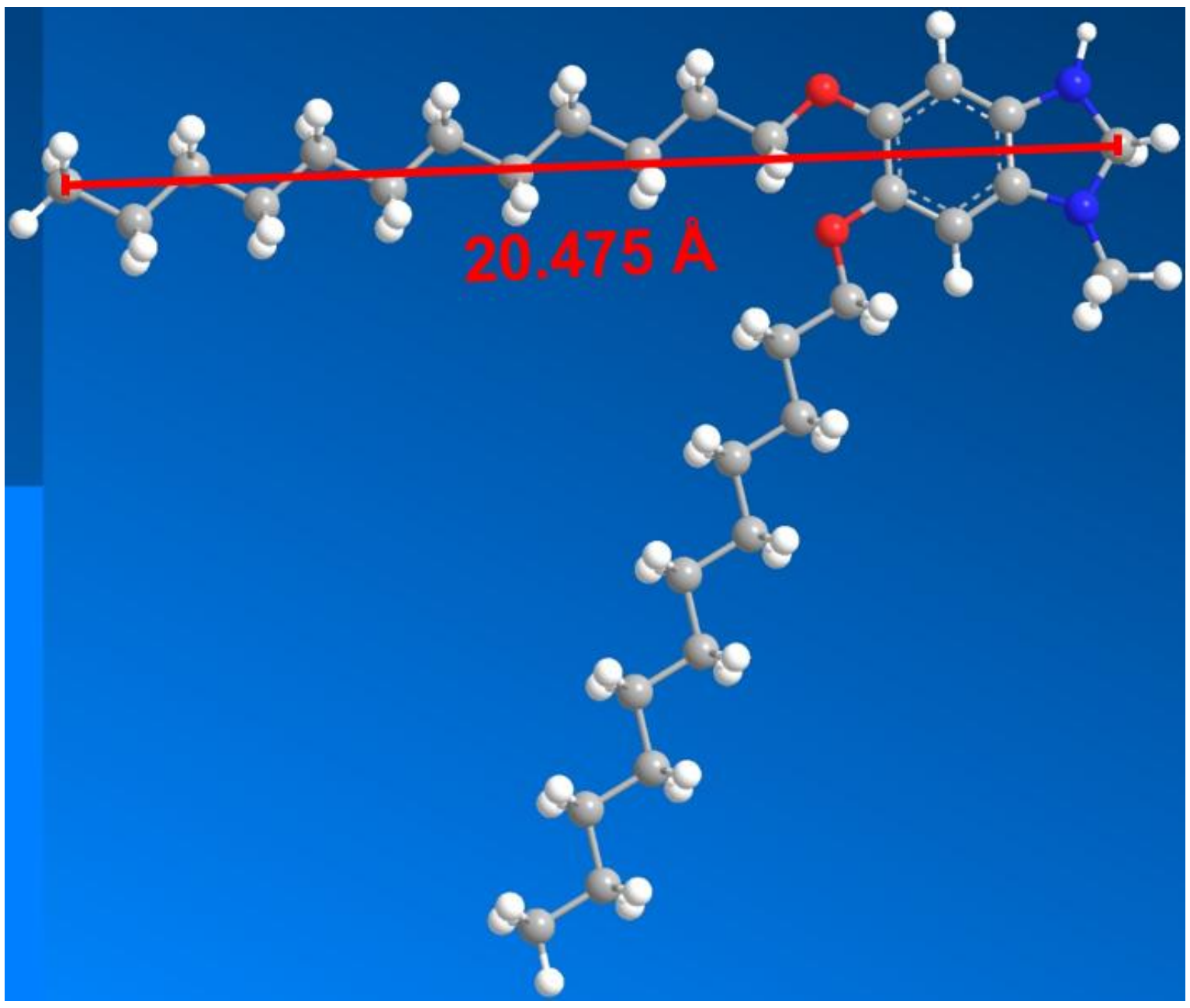

Figure S25. Exemplary depiction of the determined molecular length of **U-Bi(12)**.

4. NMR spectra

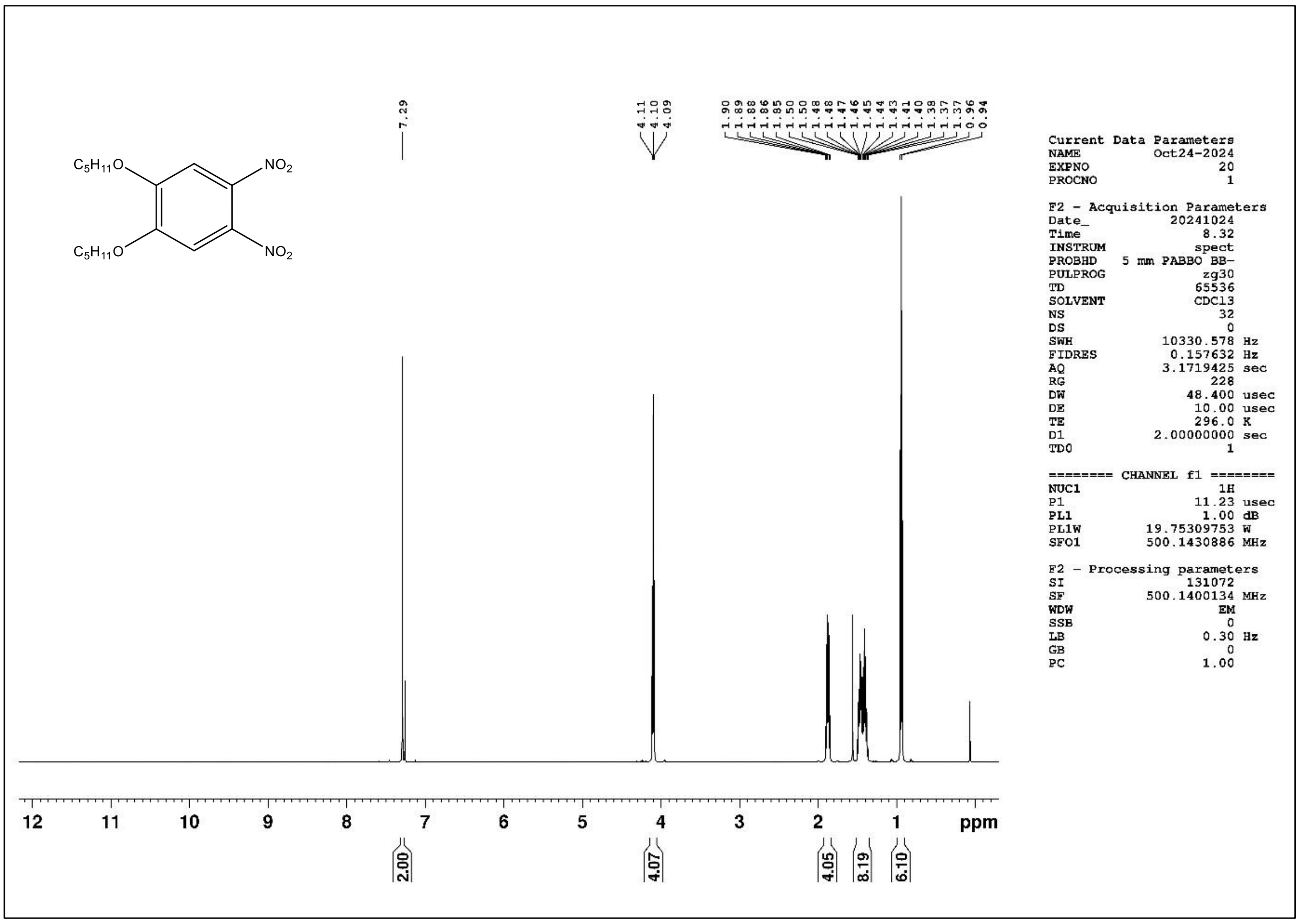

Figure S26. ^{1}H NMR of **U-Nitro(5)**.

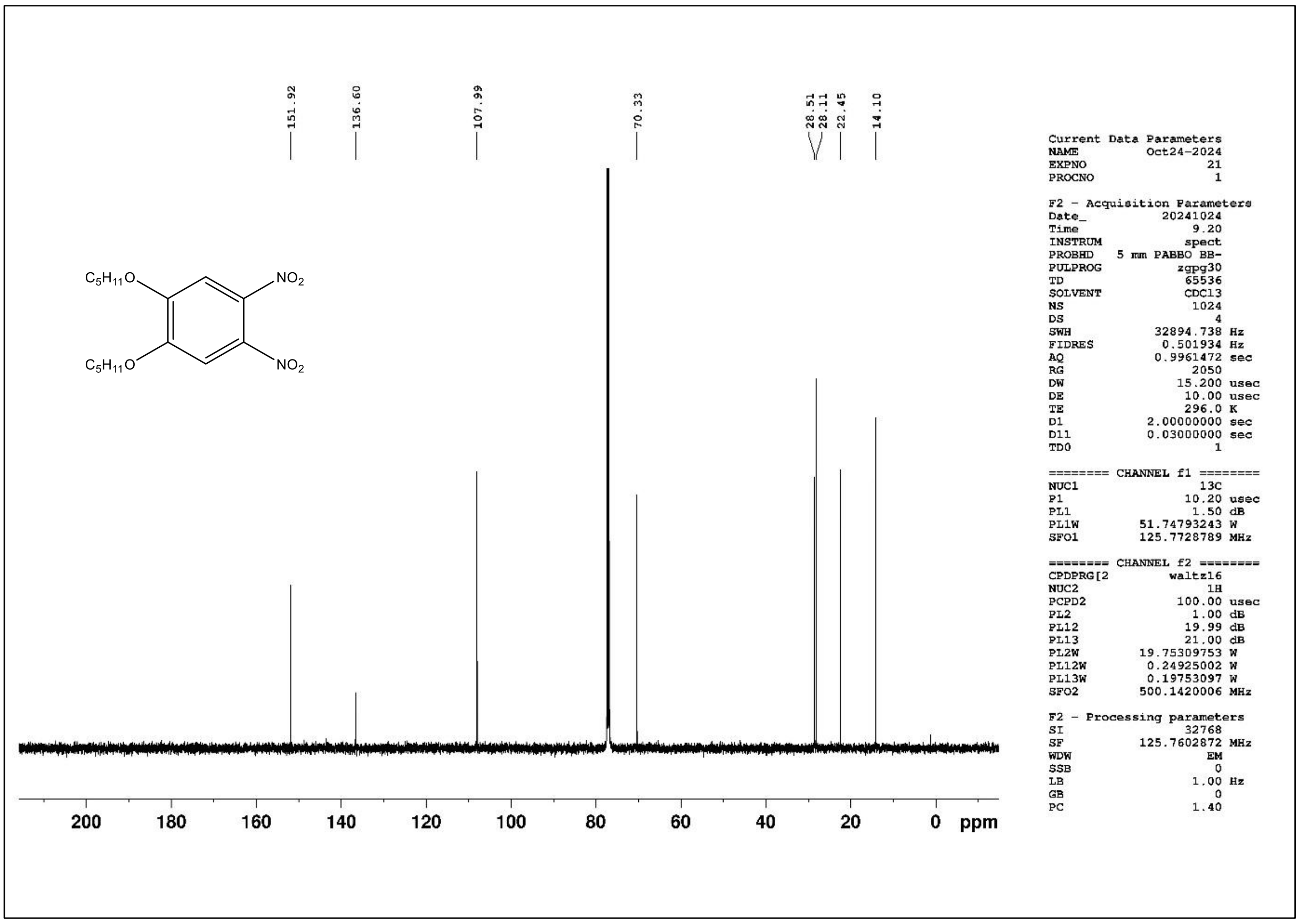

Figure S27. ^{13}C NMR of **U-Nitro(5)**.

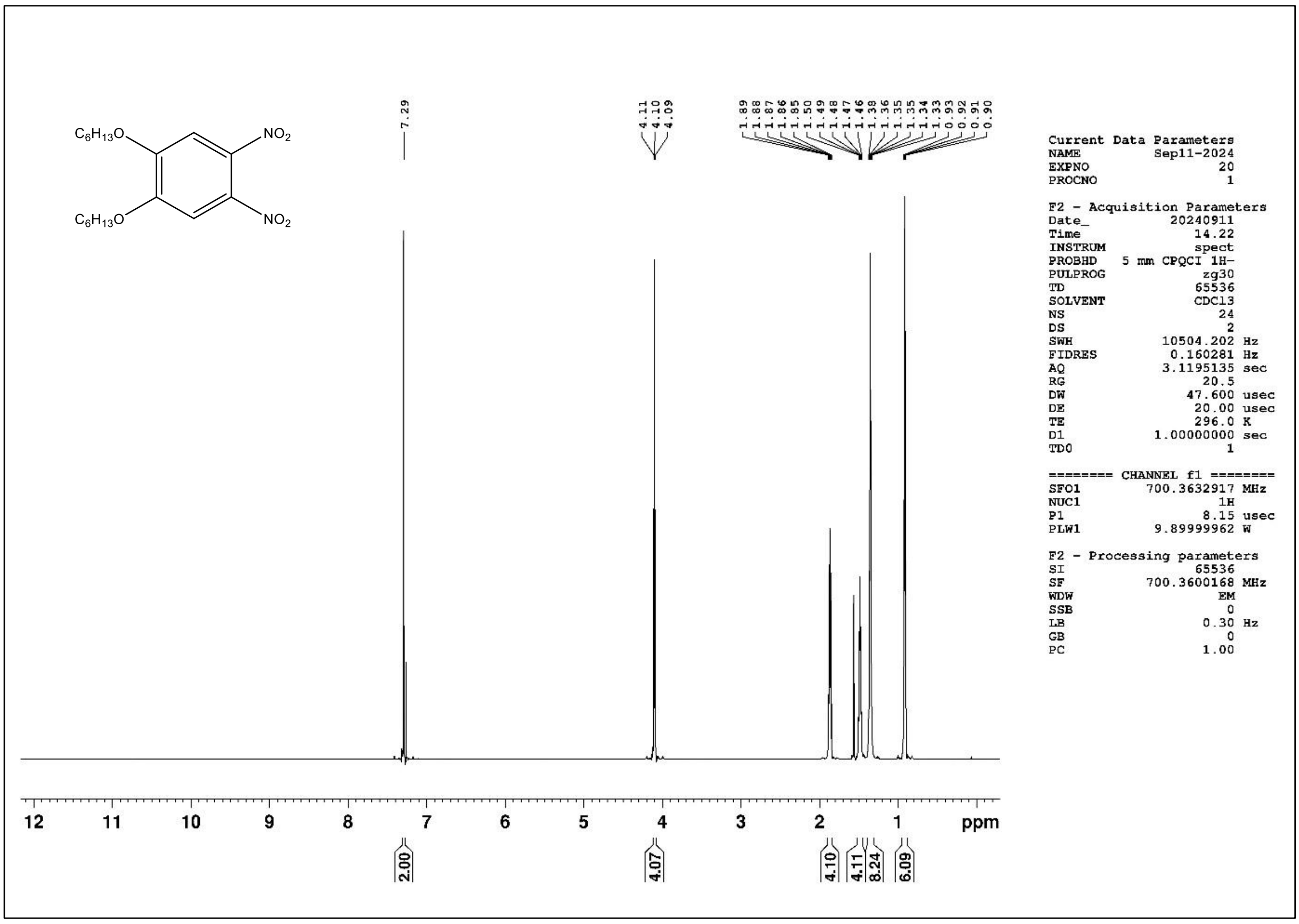

Figure S28. ^{1}H NMR of **U-Nitro(6)**.

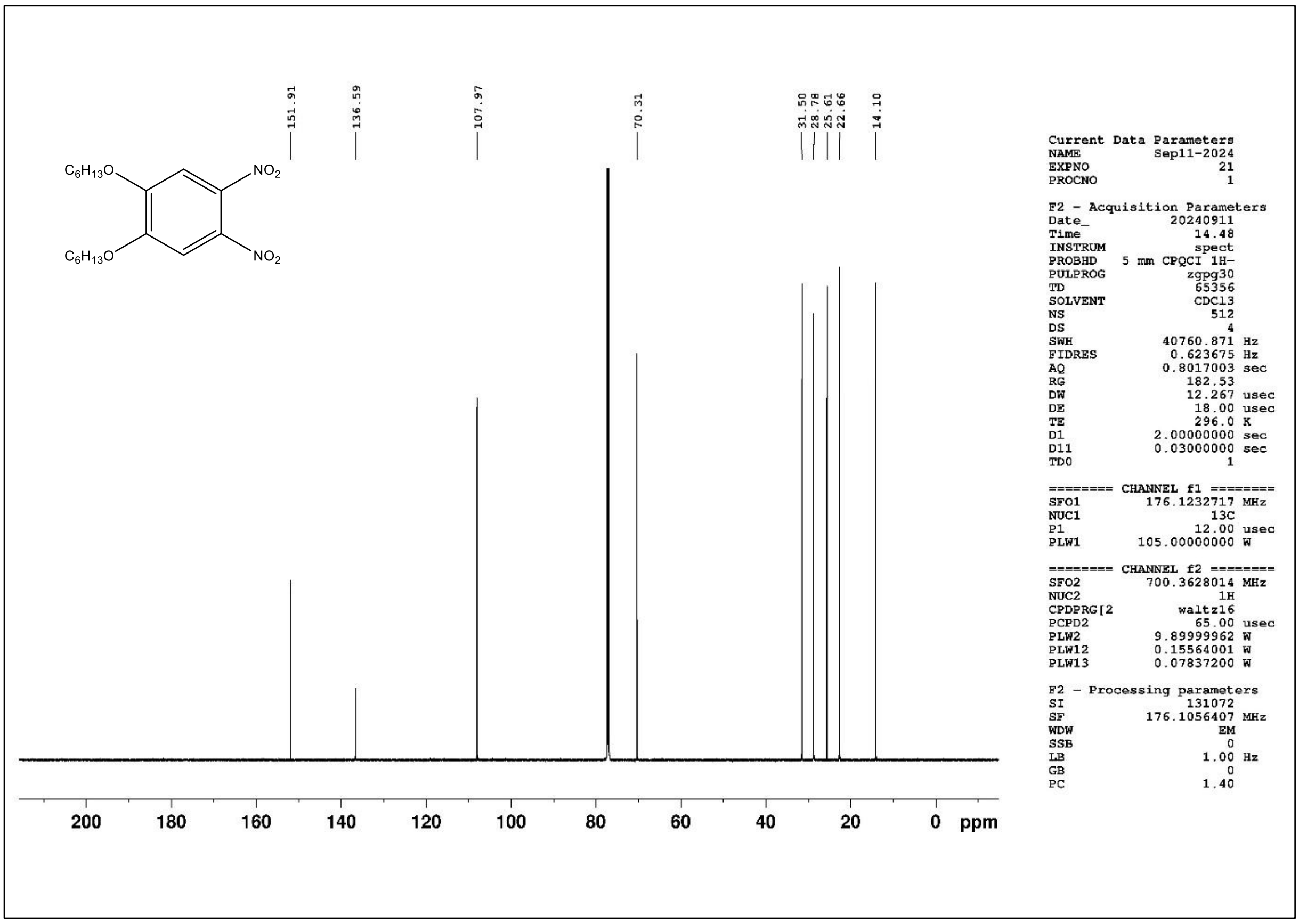

Figure S29. ^{13}C NMR of **U-Nitro(6)**.

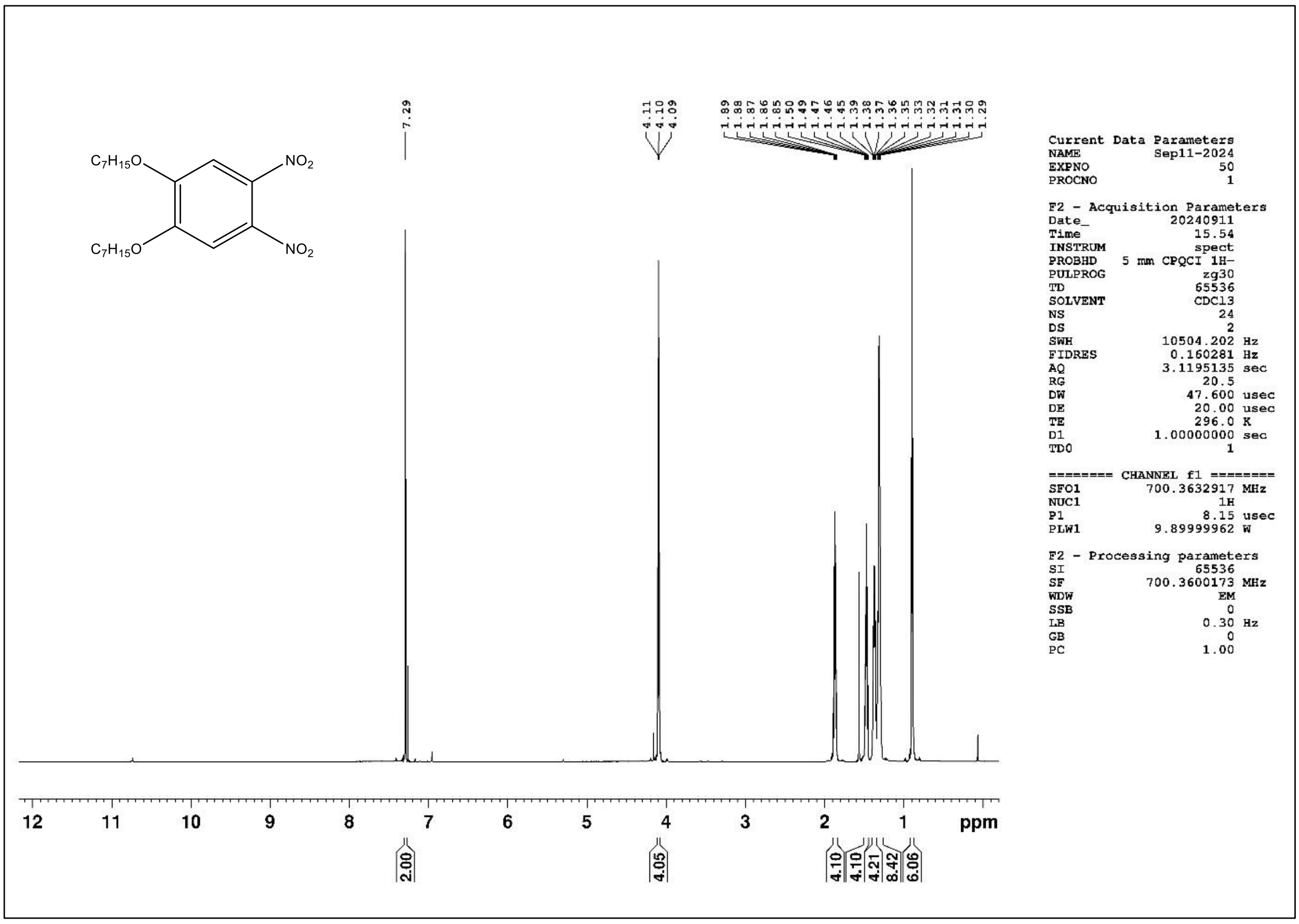

Figure S30. ^{1}H NMR of **U-Nitro(7)**.

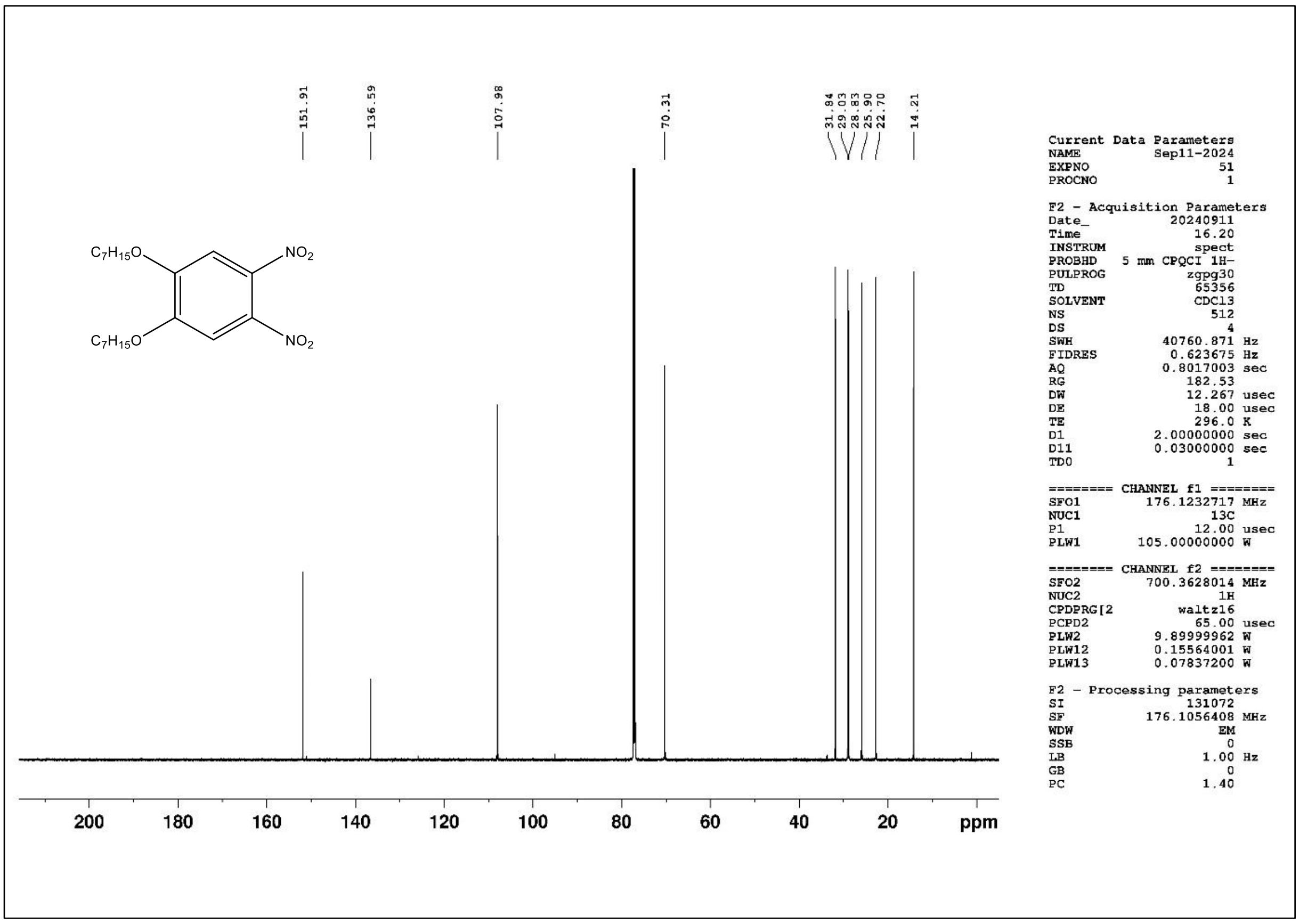

Figure S31. ^{13}C NMR of **U-Nitro(7)**.

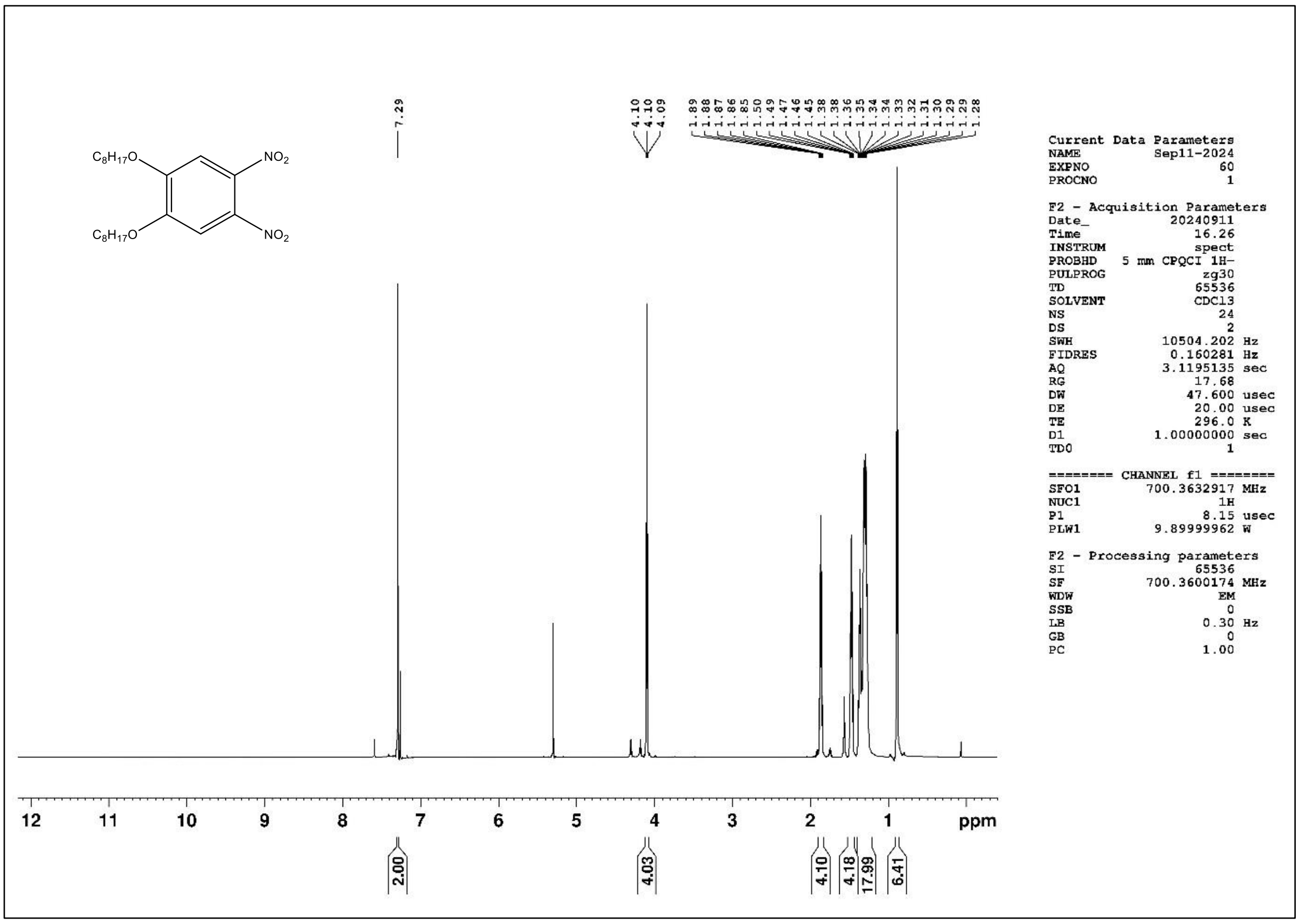

Figure S32. ^{1}H NMR of **U-Nitro(8)**.

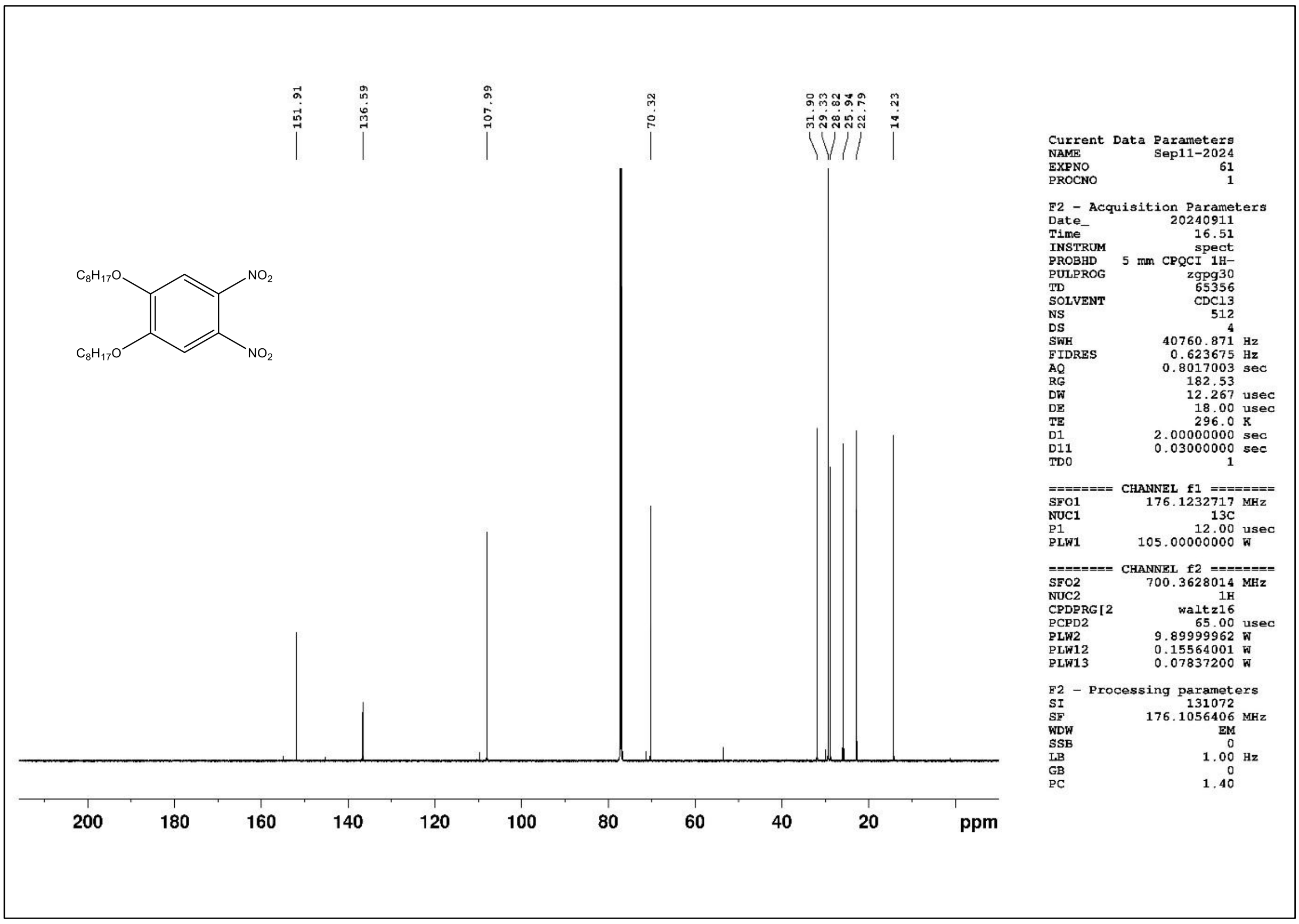

Figure S33. ^{13}C NMR of **U-Nitro(8)**.

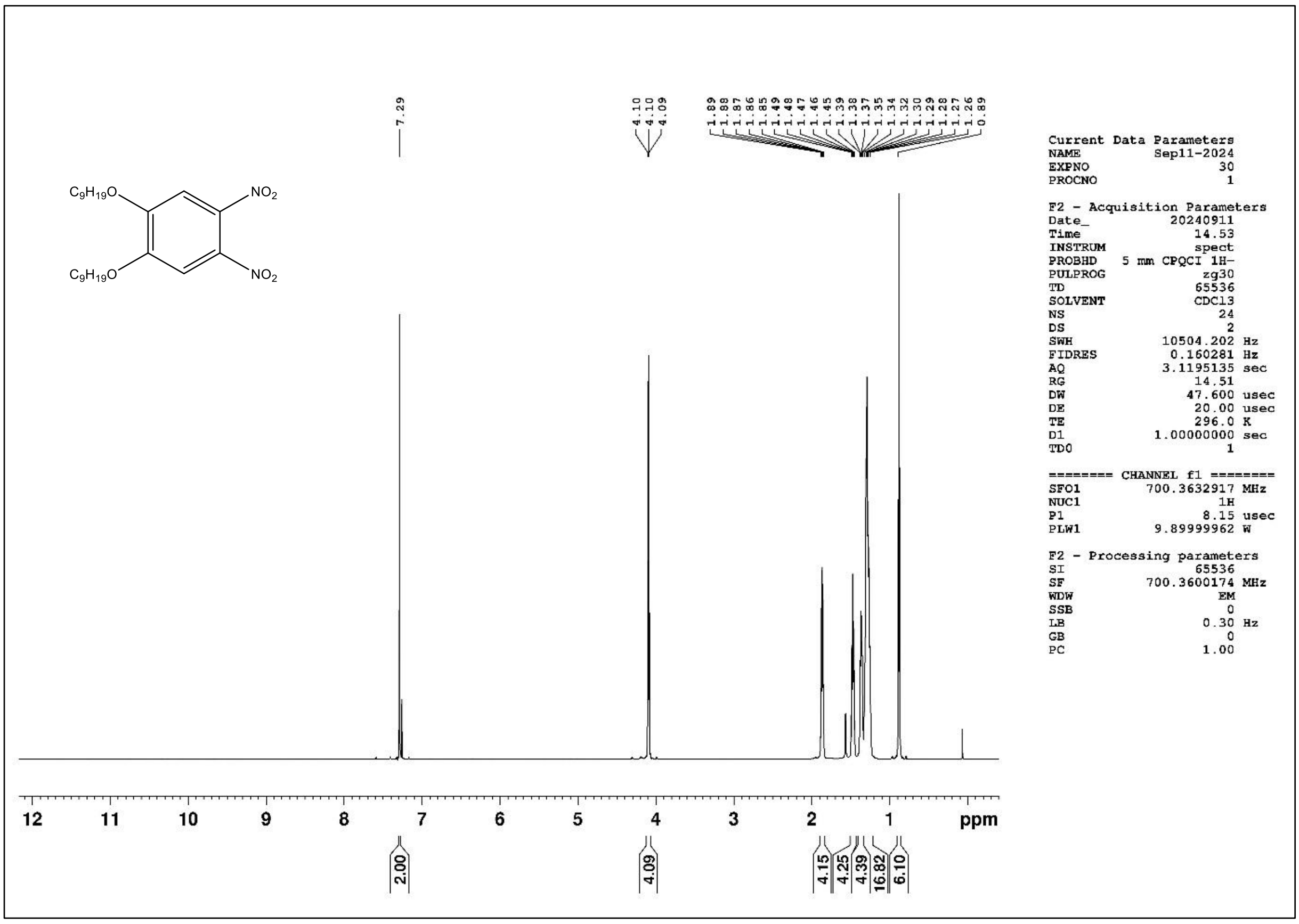

Figure S34. ^{1}H NMR of **U-Nitro(9)**.

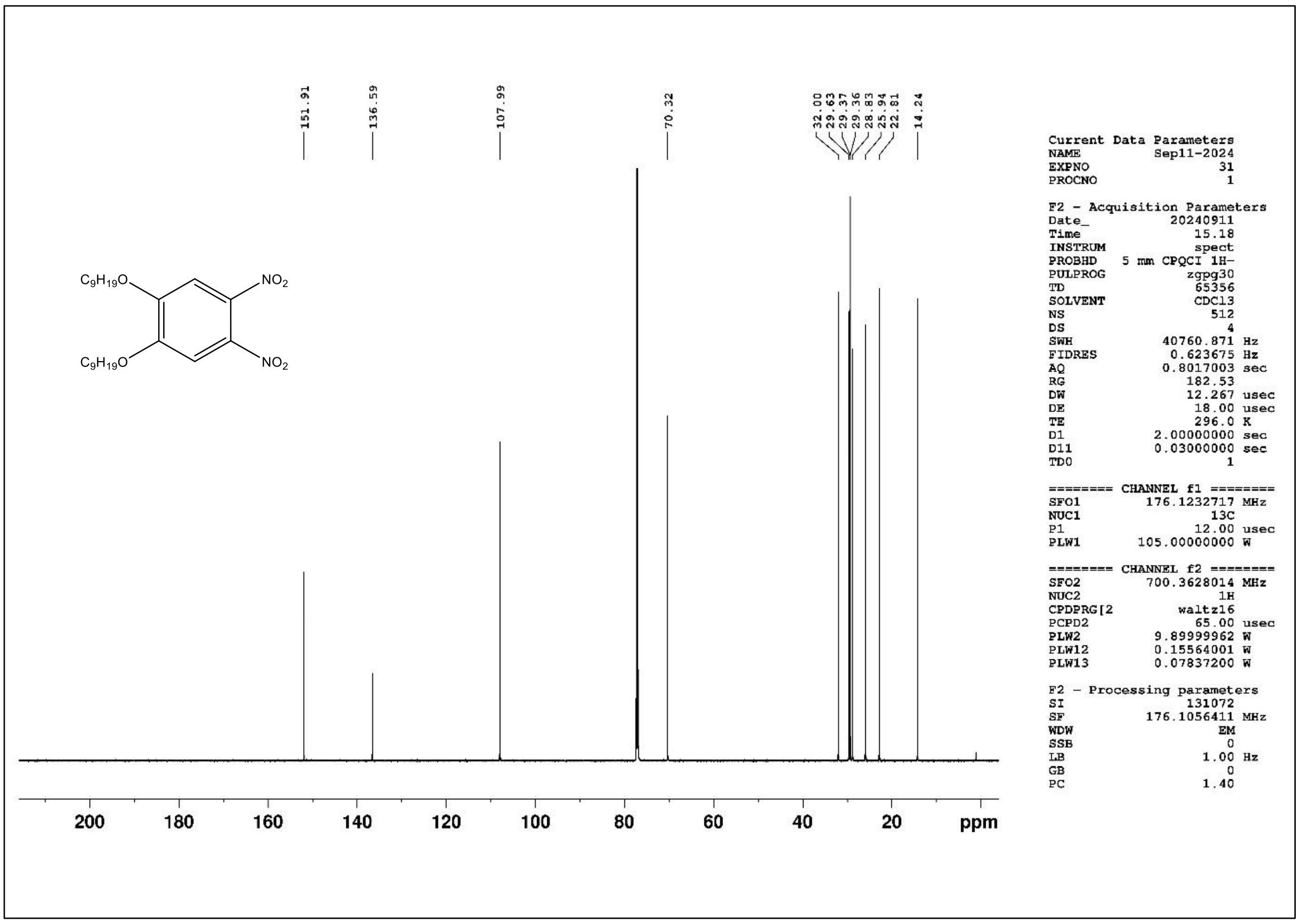

Figure S35. ^{13}C NMR of **U-Nitro(9)**.

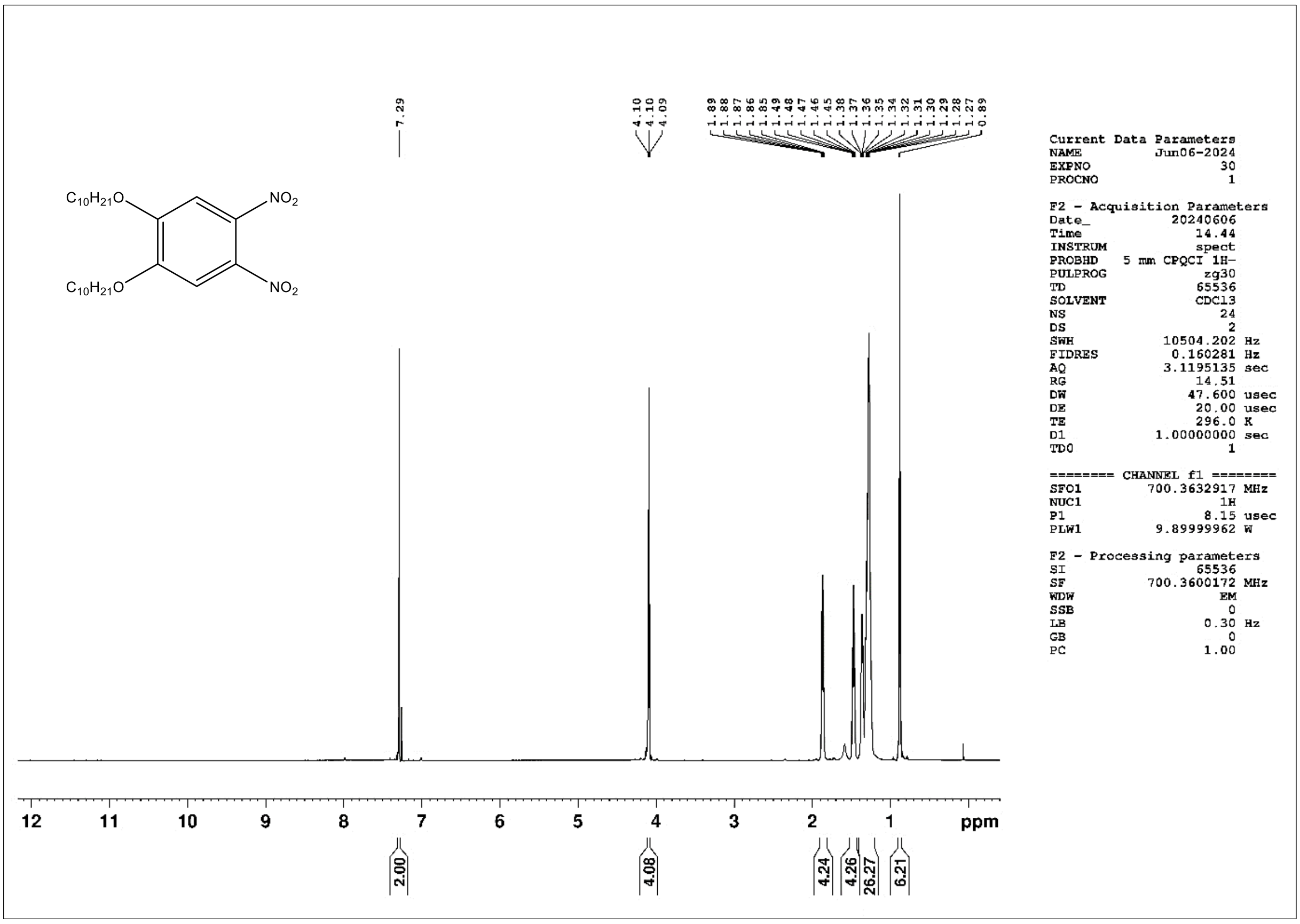

Figure S36. ^{1}H NMR of **U-Nitro(10)**.

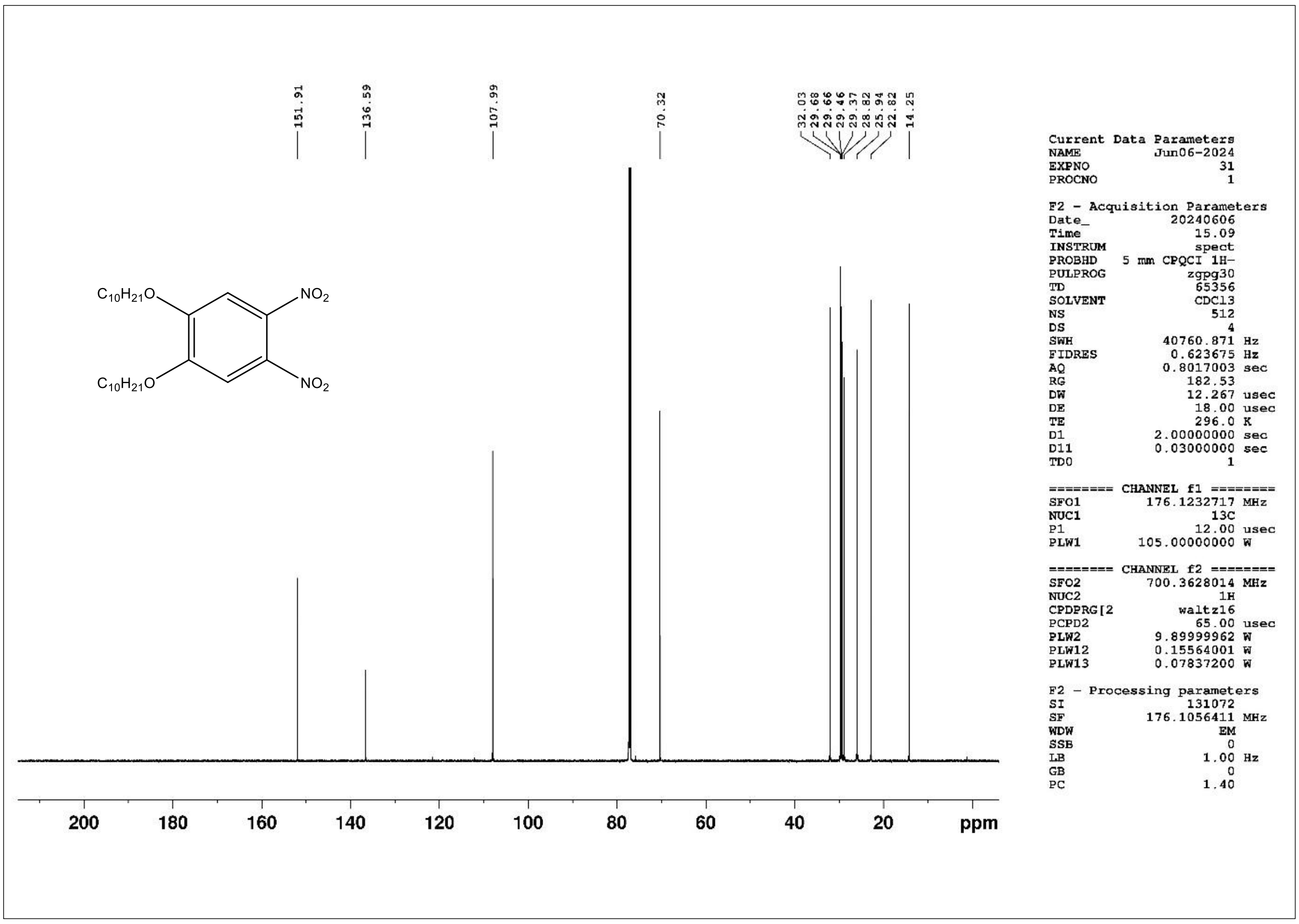

Figure S37. ^{13}C NMR of **U-Nitro(10)**.

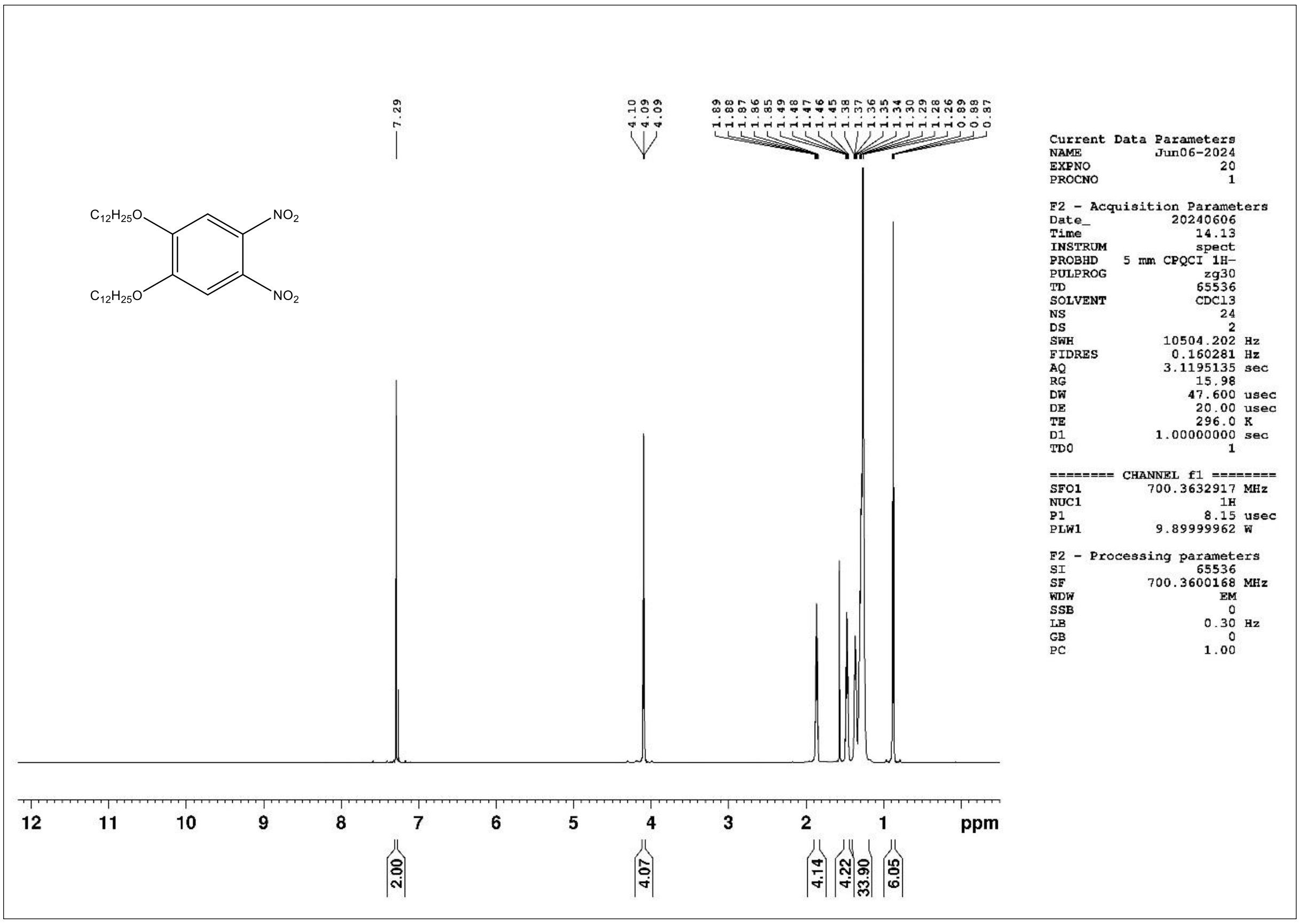

Figure S38. ^{1}H NMR of **U-Nitro(12)**.

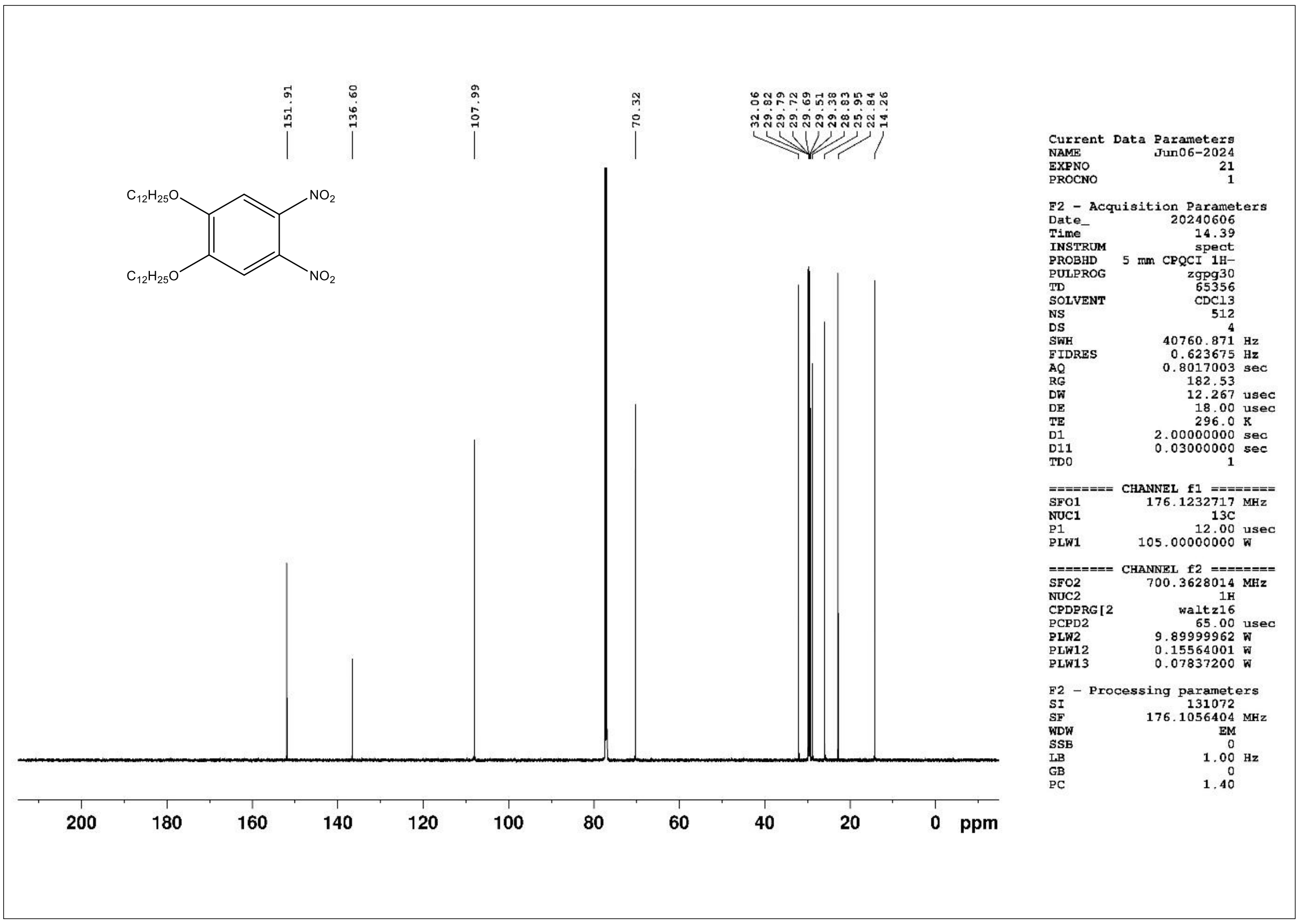

Figure S39. ^{13}C NMR of **U-Nitro(12)**.

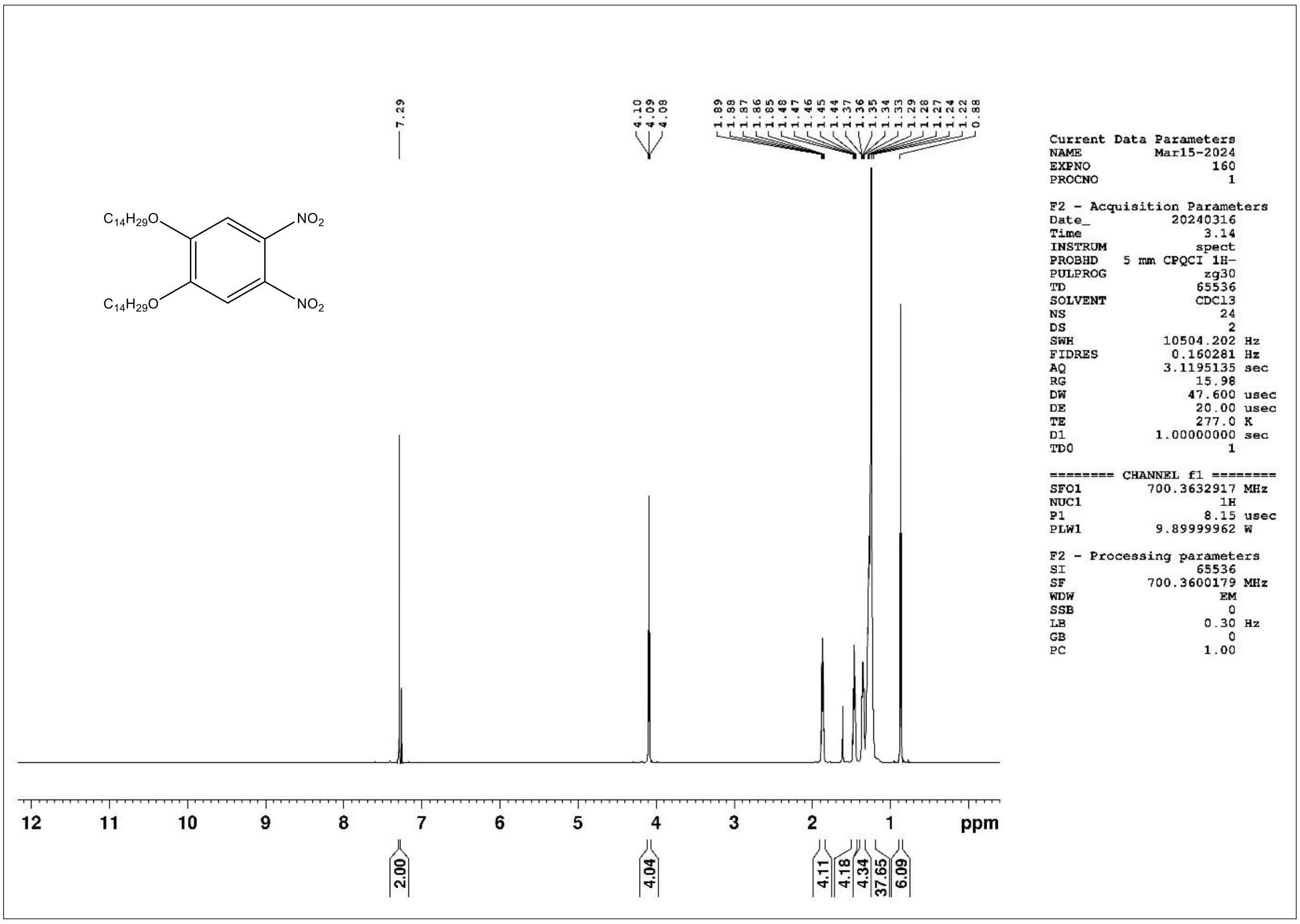

Figure S40. ^{1}H NMR of **U-Nitro(14)**.

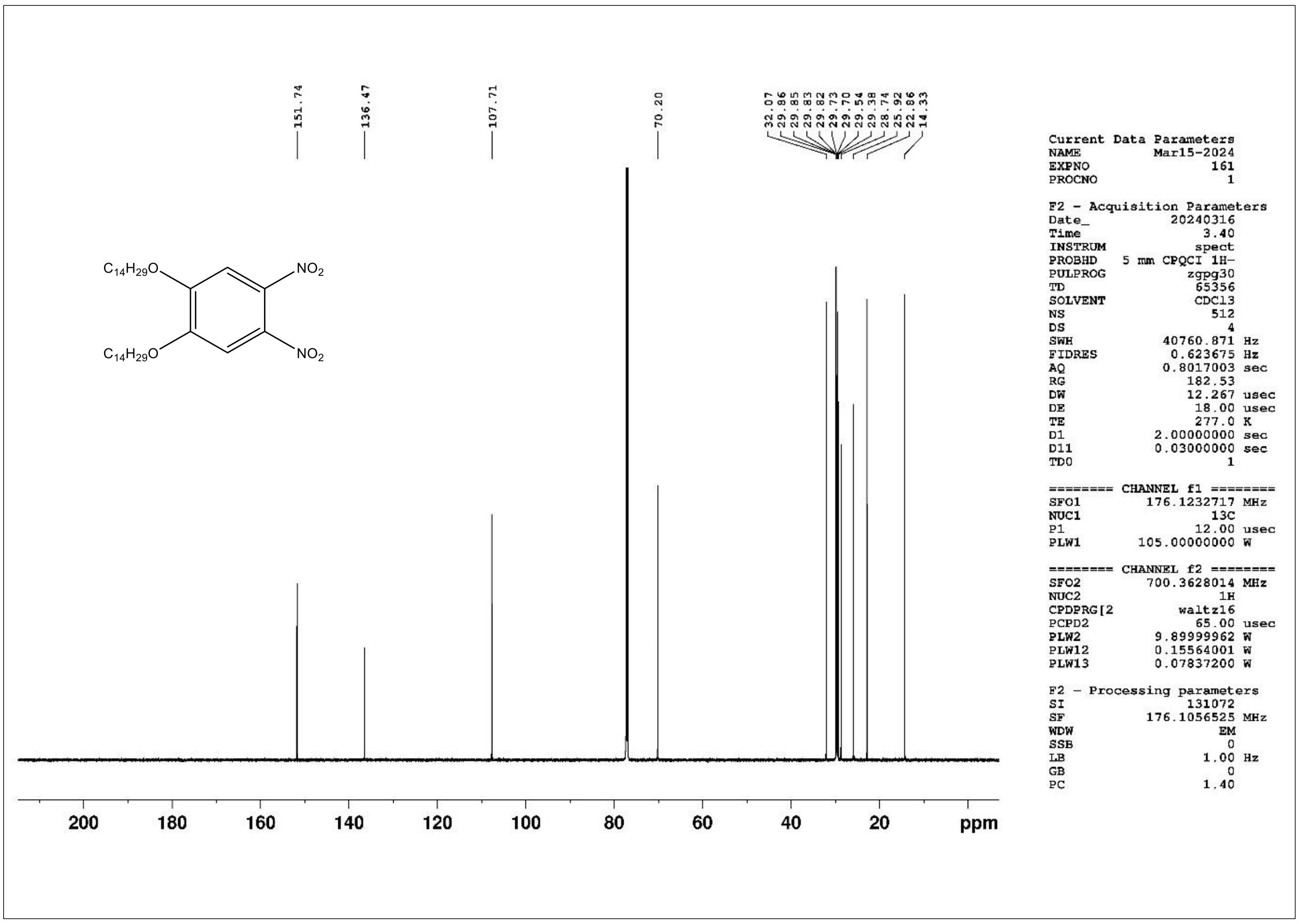

Figure S41. ^{13}C NMR of **U-Nitro(14)**.

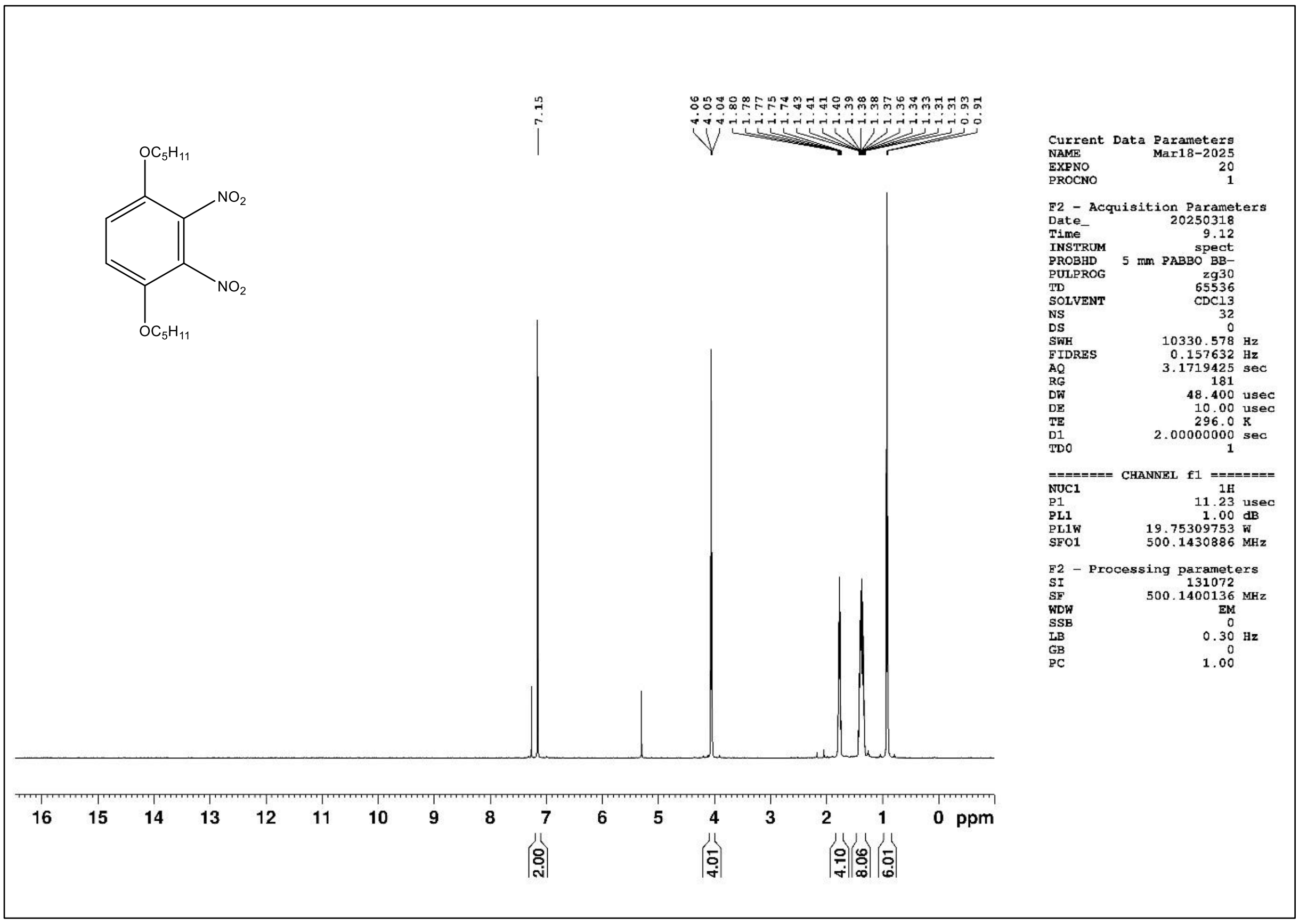

Figure S42. ^{1}H NMR of **L-Nitro(5)**.

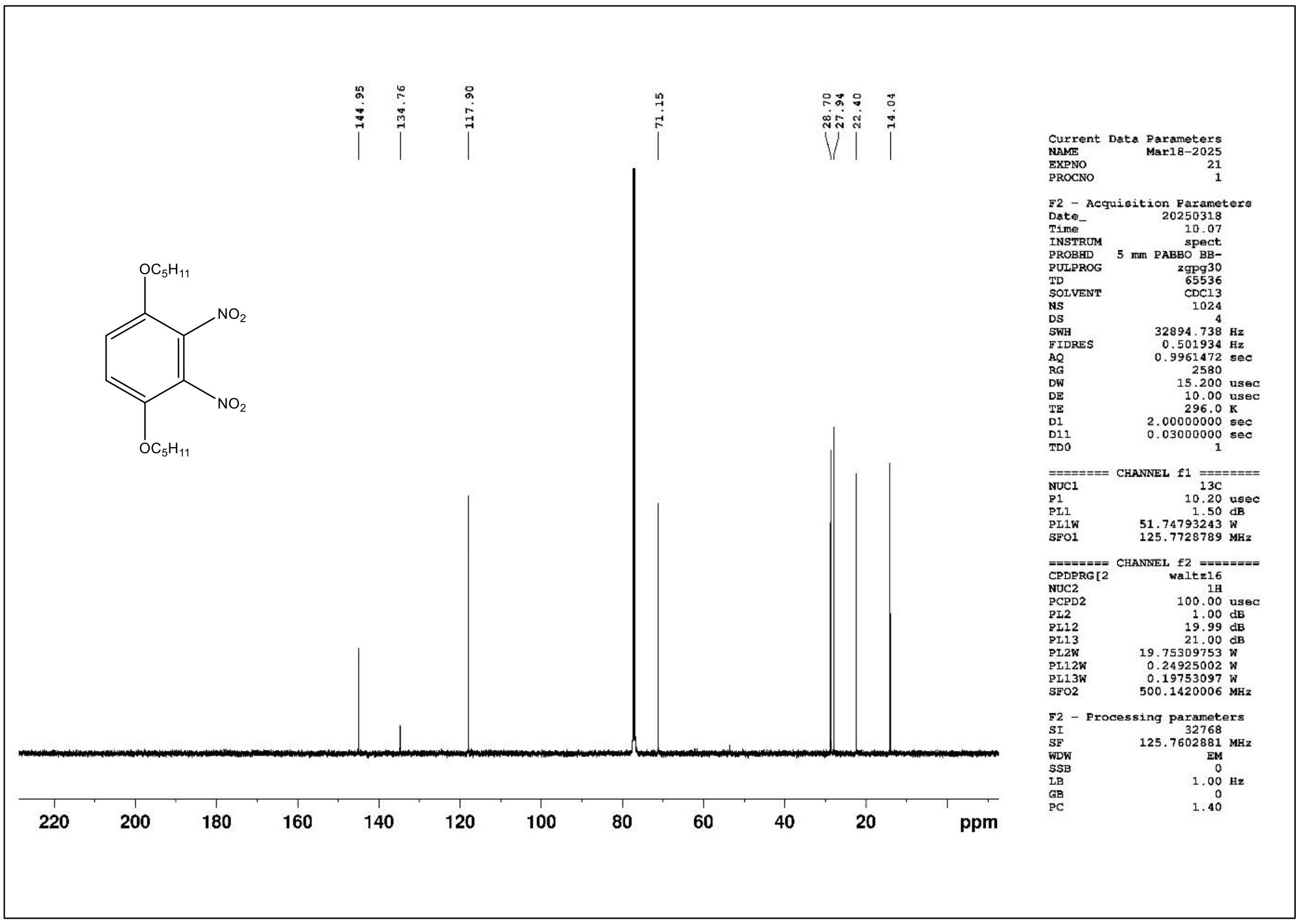

Figure S43. ^{13}C NMR of **L-Nitro(5)**.

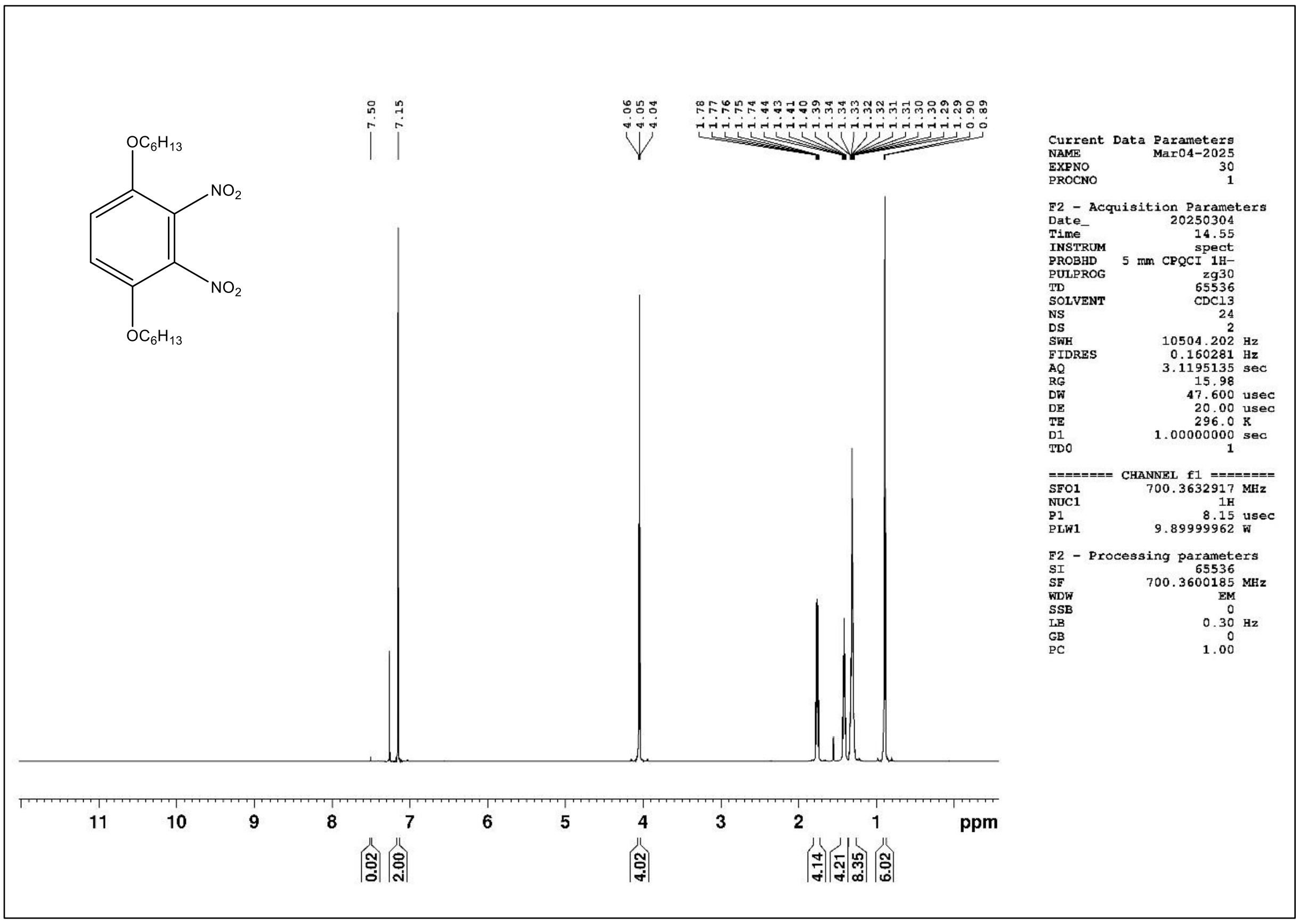

Figure S44. ^{1}H NMR of **L-Nitro(6)**.

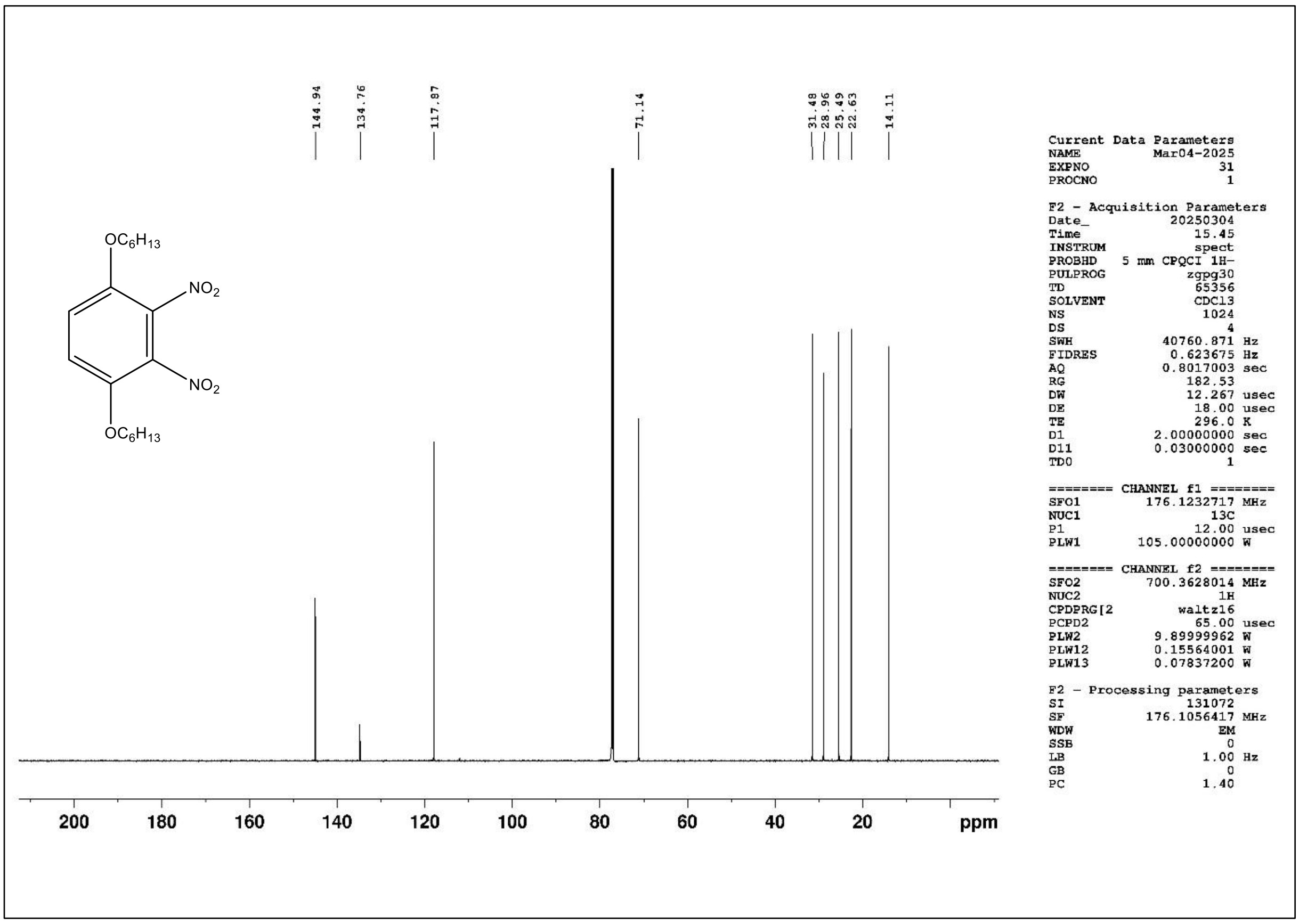

Figure S45. ^{13}C NMR of **L-Nitro(6)**.

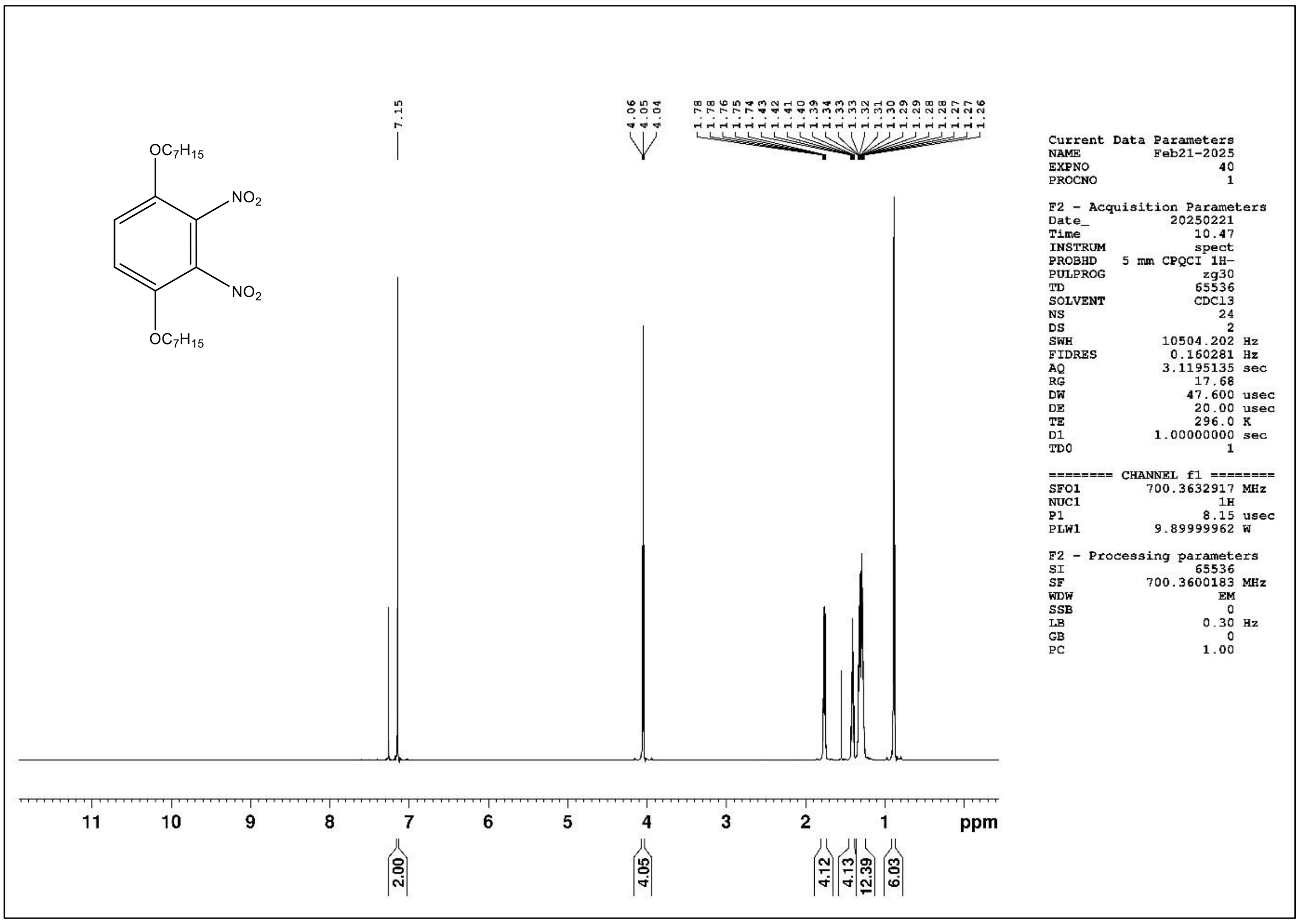

Figure S46. ^{1}H NMR of **L-Nitro(7)**.

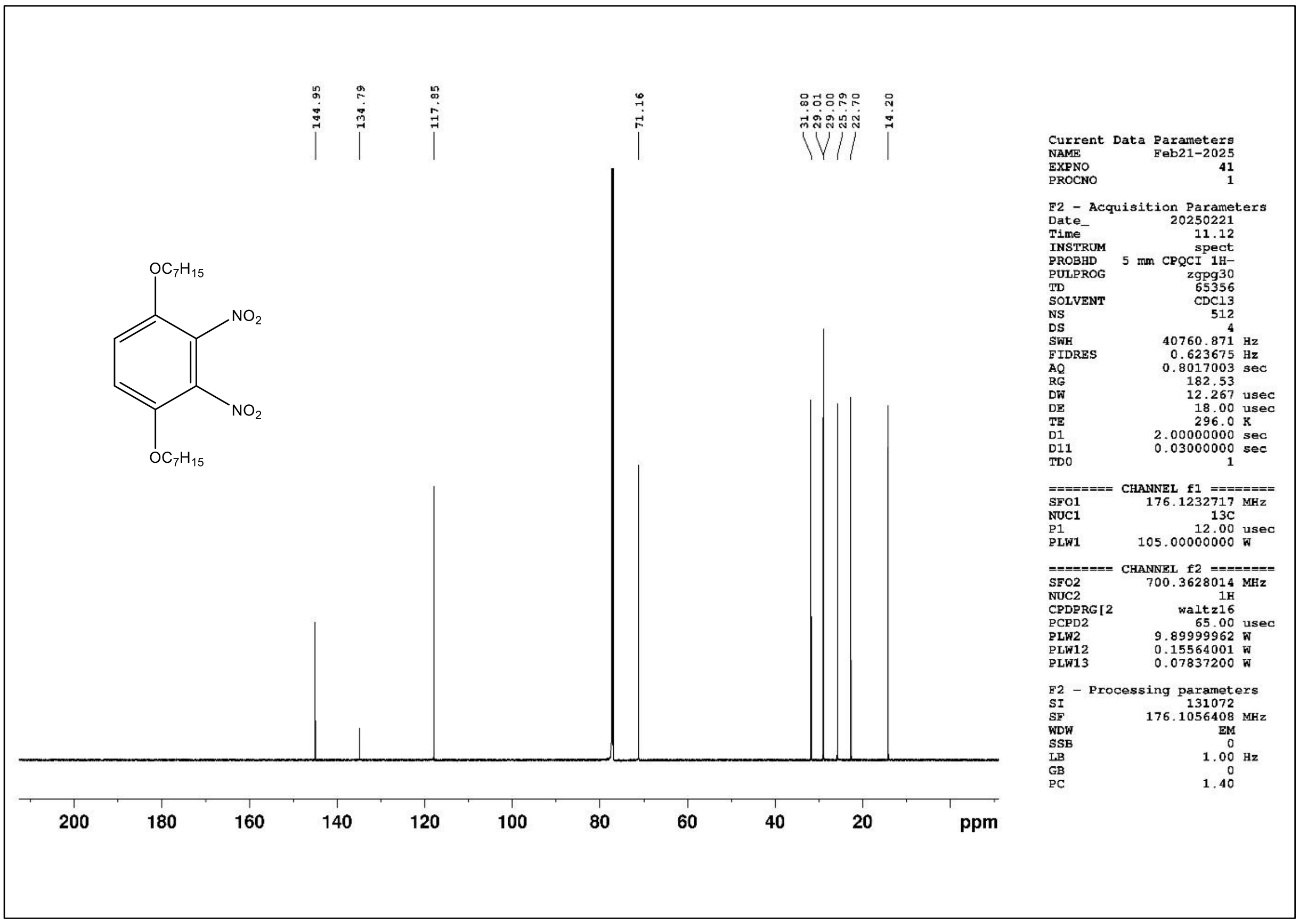

Figure S47. ^{13}C NMR of **L-Nitro(7)**.

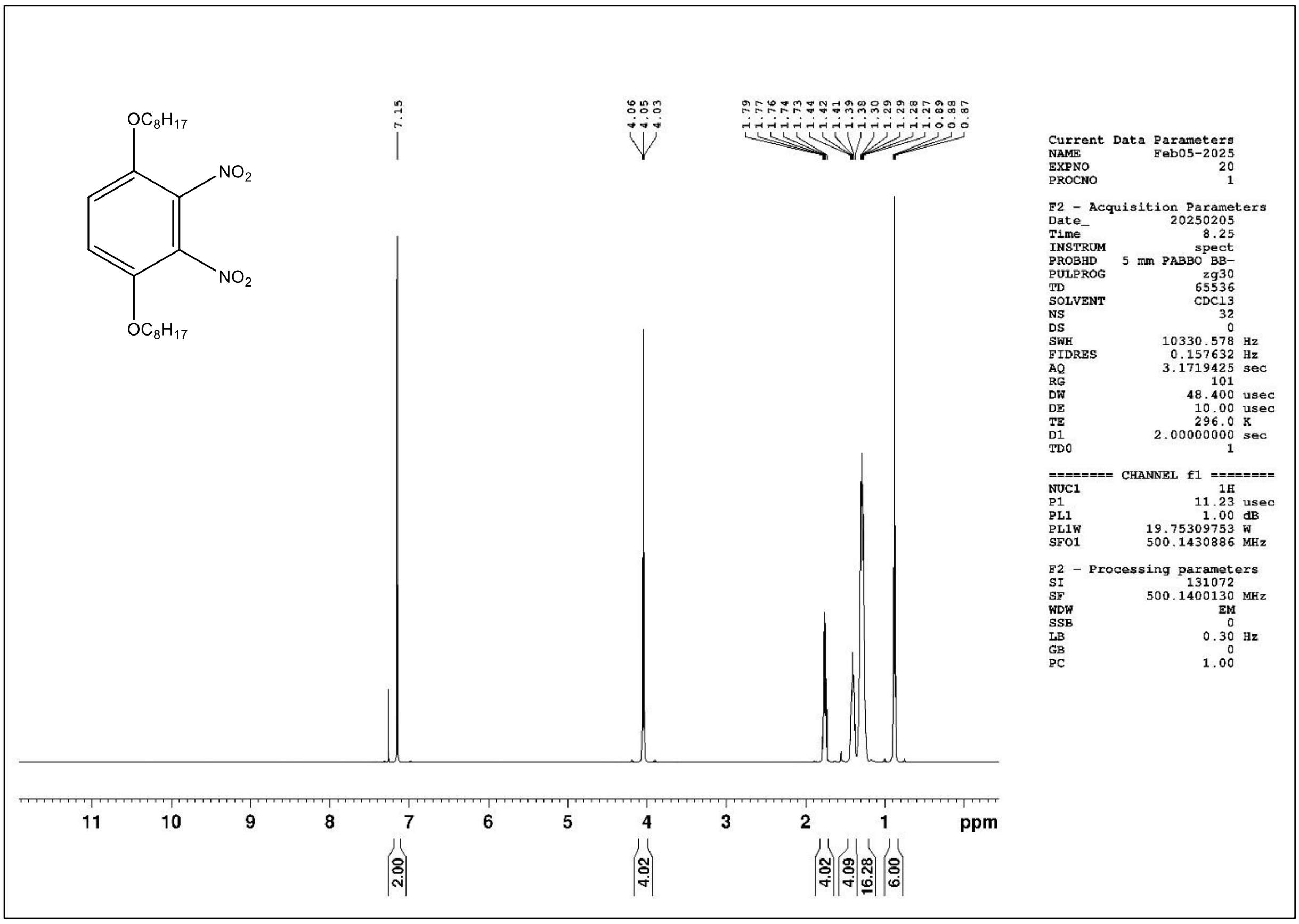

Figure S48. ^{1}H NMR of **L-Nitro(8)**.

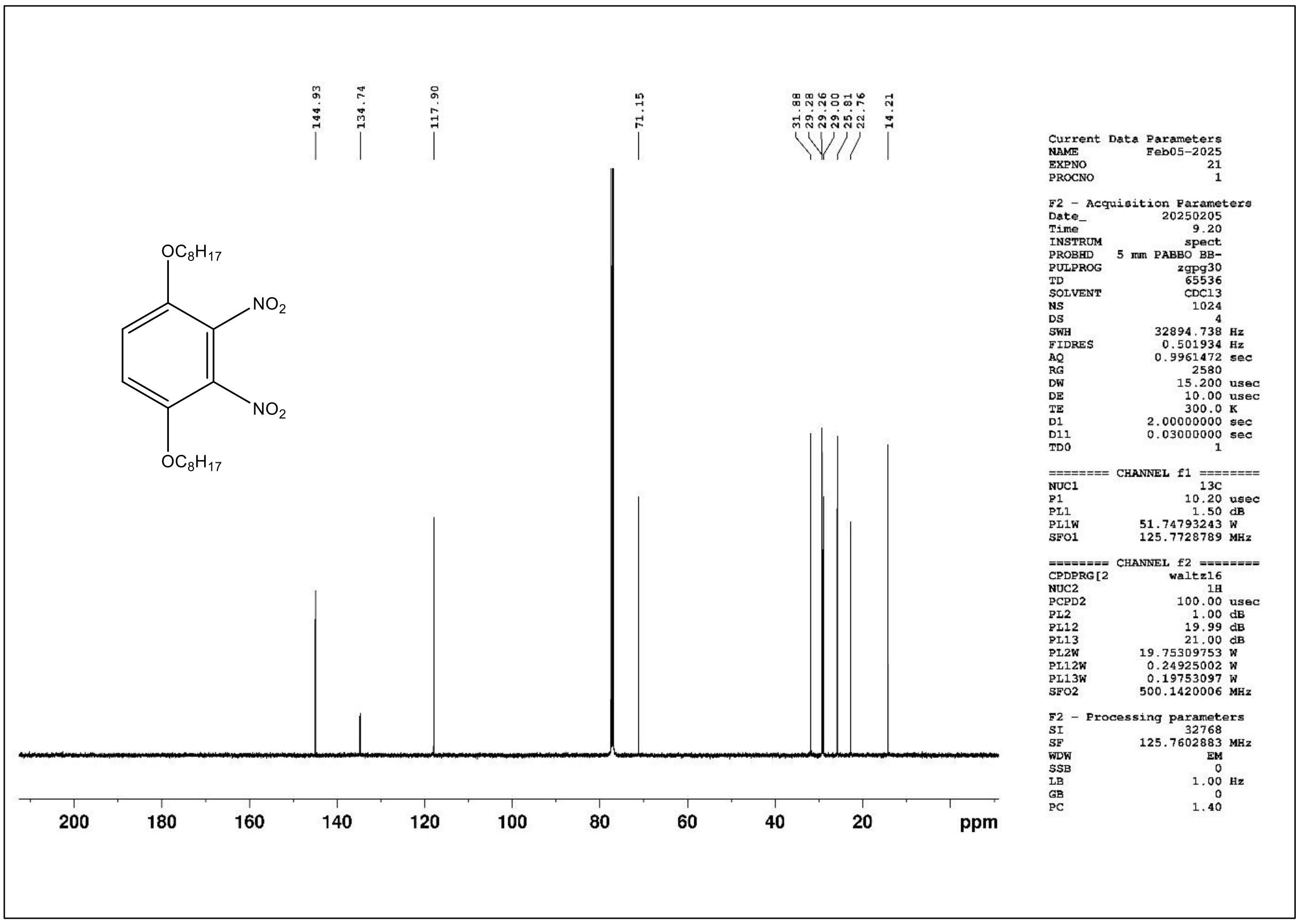

Figure S49. ^{13}C NMR of **L-Nitro(8)**.

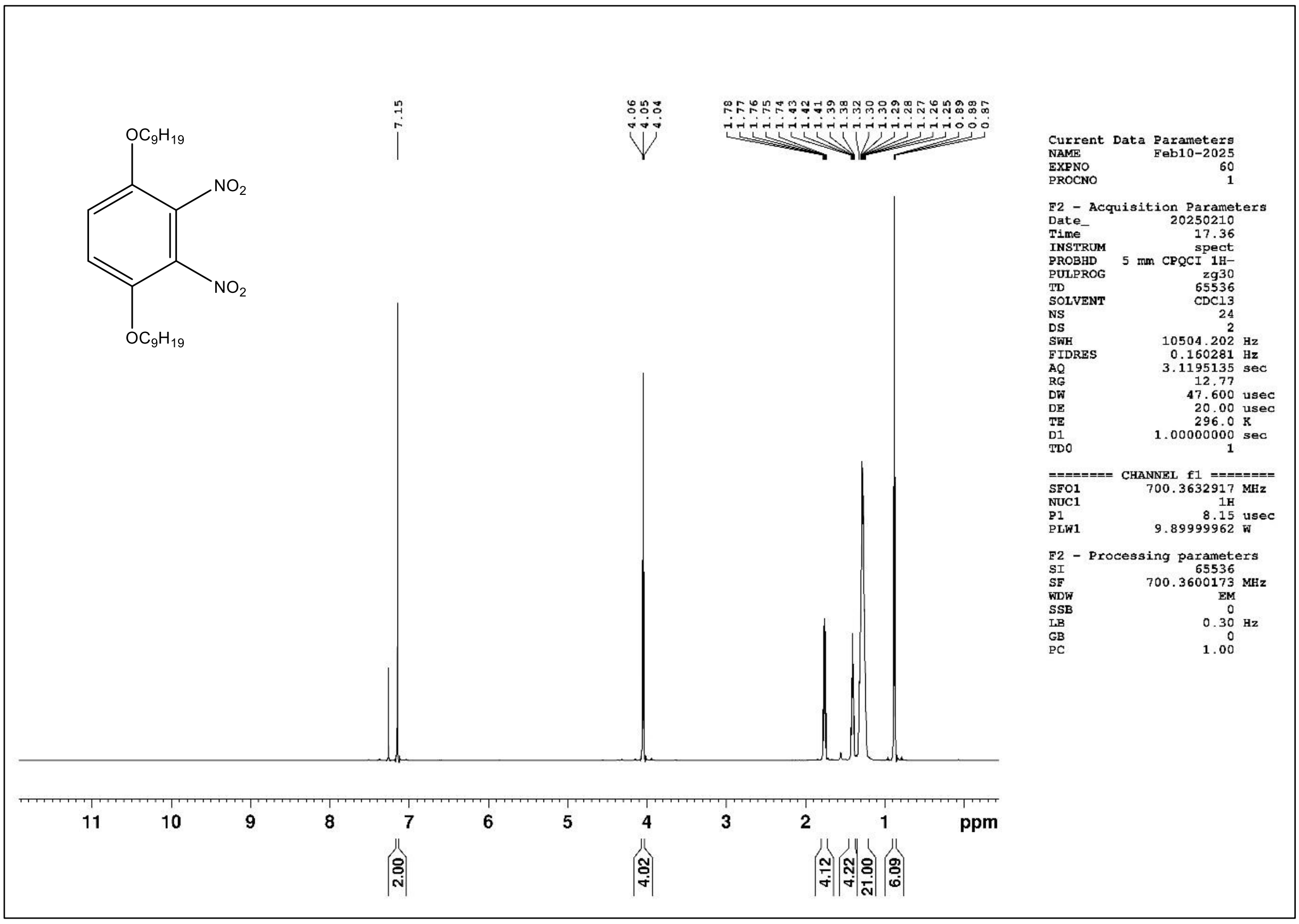

Figure S50. ^{1}H NMR of **L-Nitro(9)**.

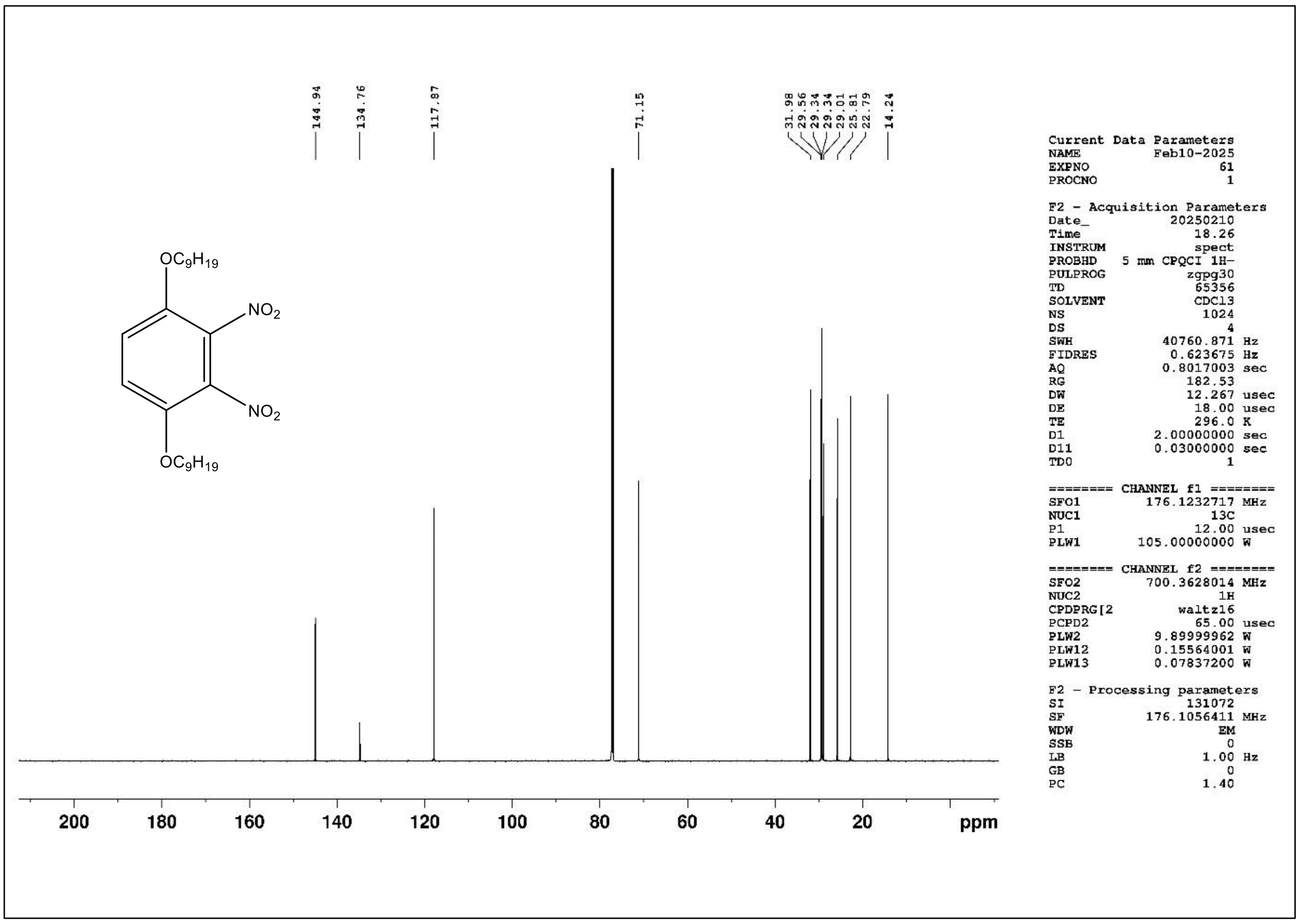

Figure S51. ^{13}C NMR of **L-Nitro(9)**.

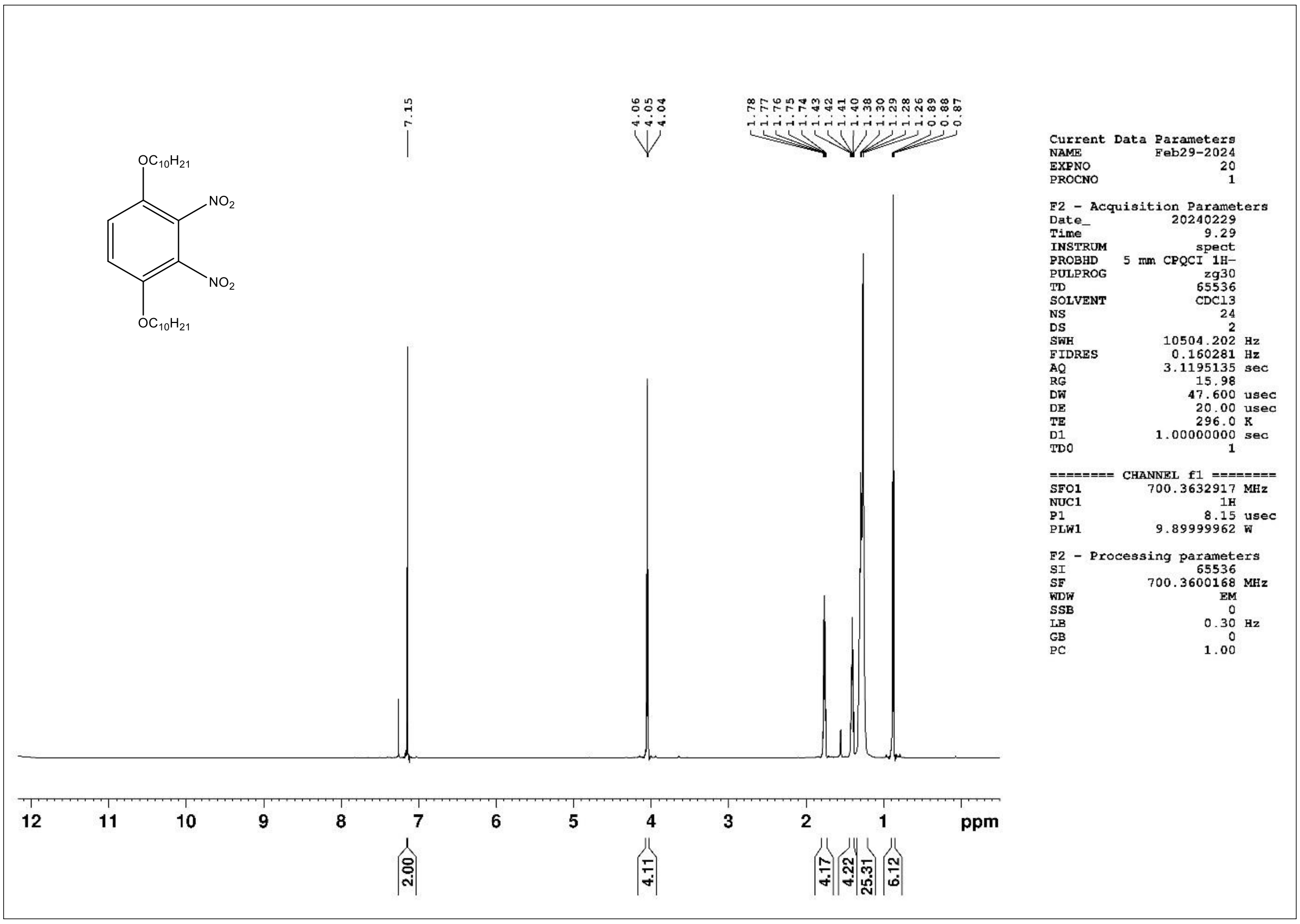

Figure S52. ^{1}H NMR of **L-Nitro(10)**.

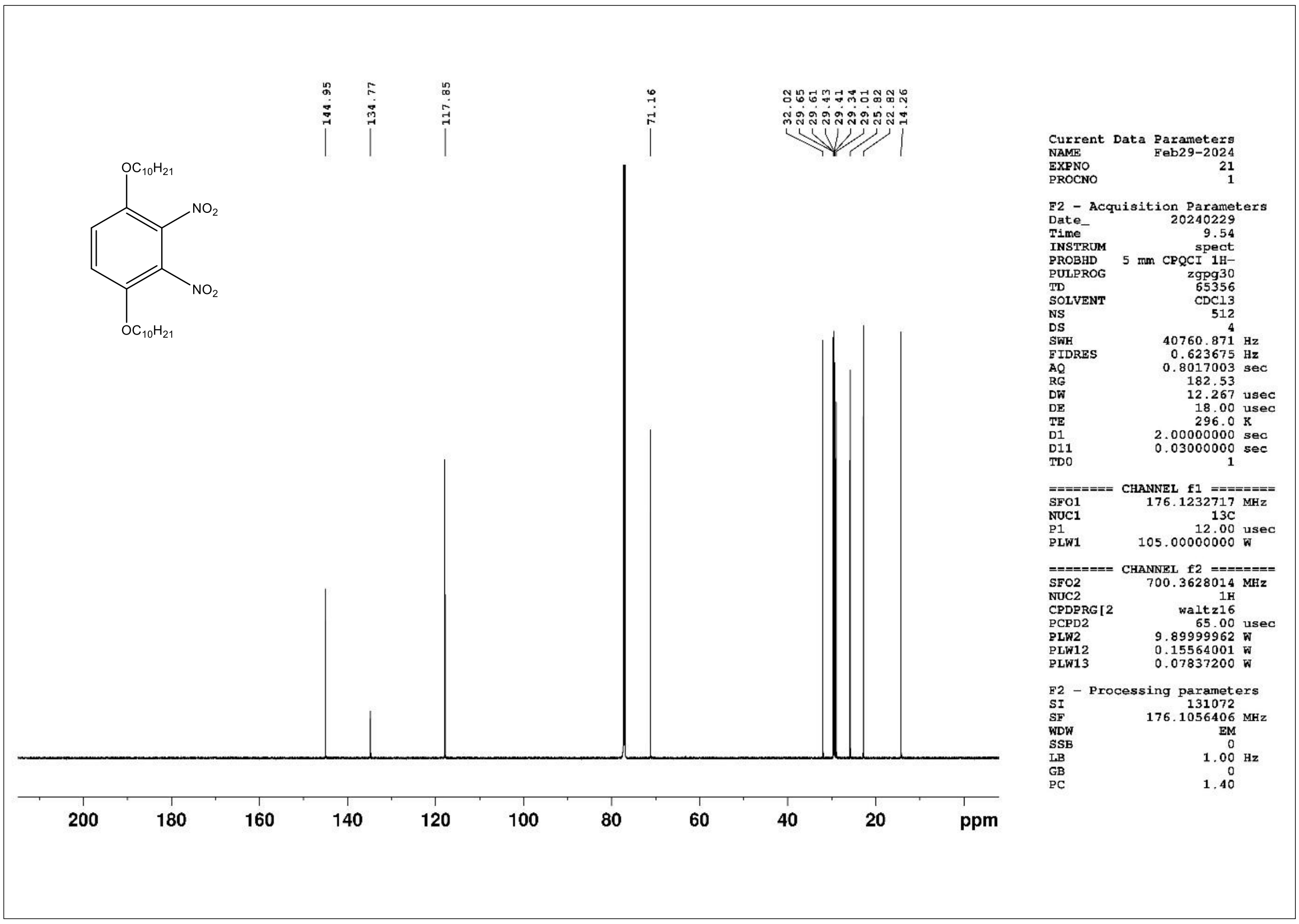

Figure S53. ^{13}C NMR of **L-Nitro(10)**.

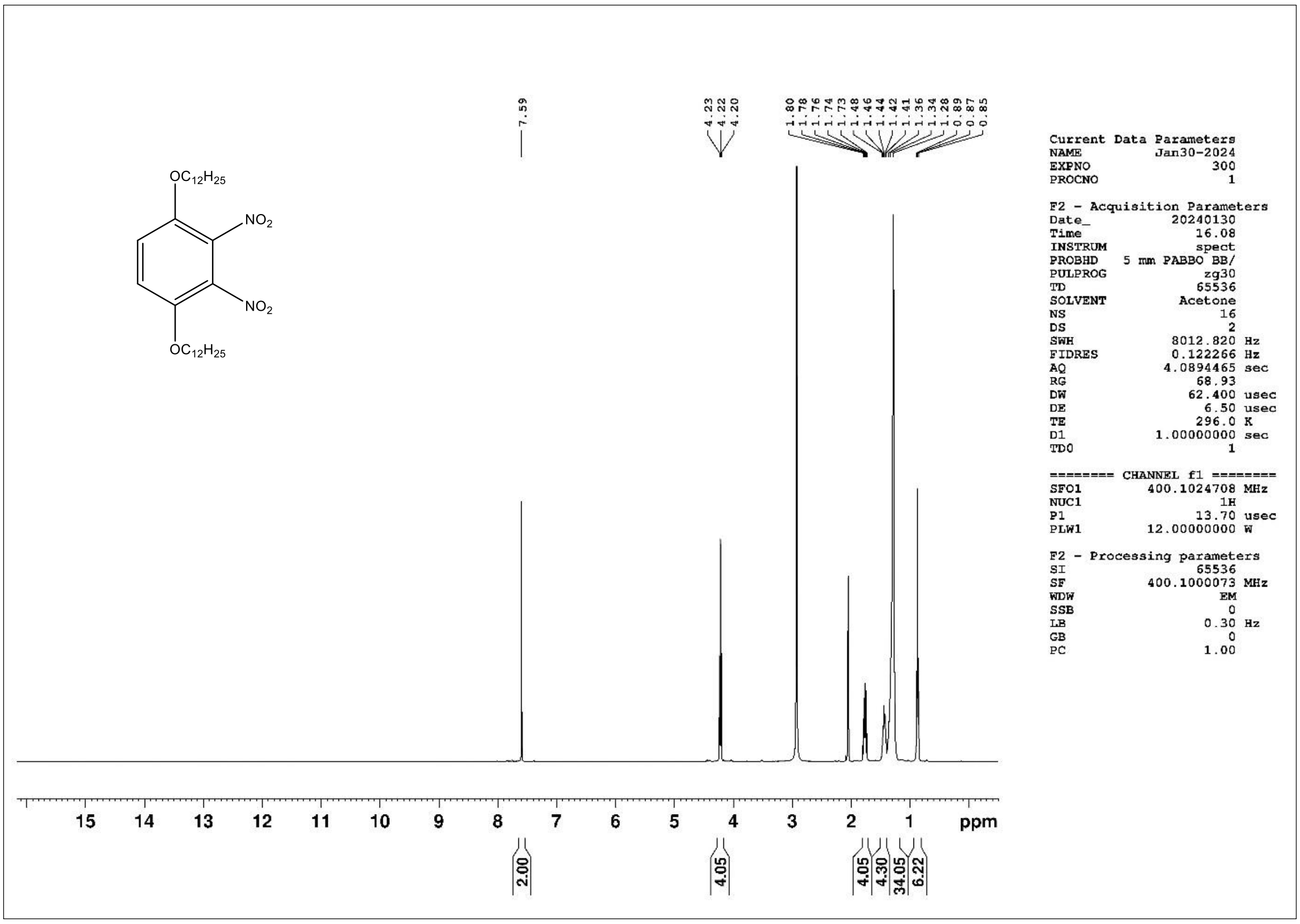

Figure S54. ^{1}H NMR of **L-Nitro(12)**.

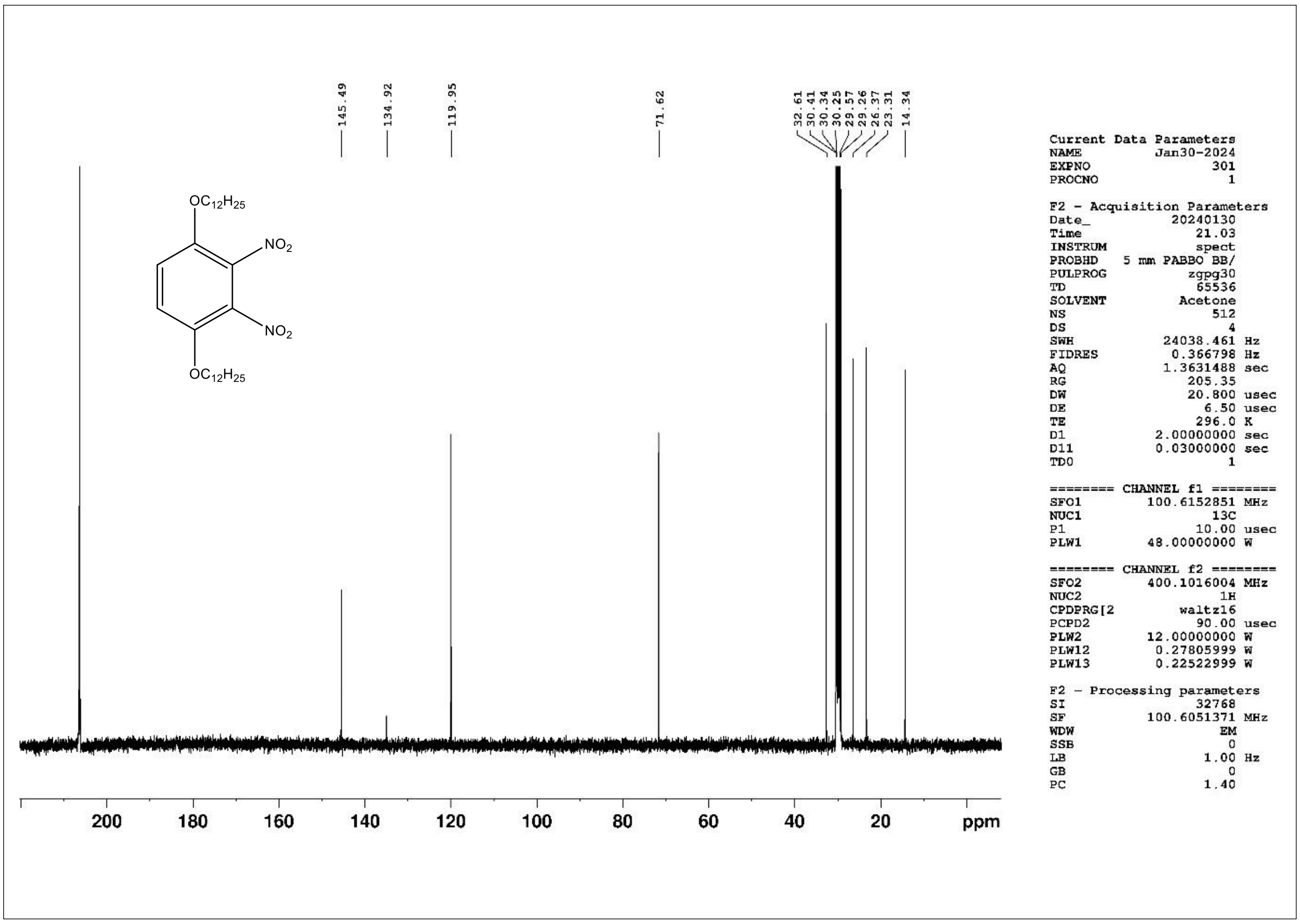

Figure S55. ^{13}C NMR of **L-Nitro(12)**.

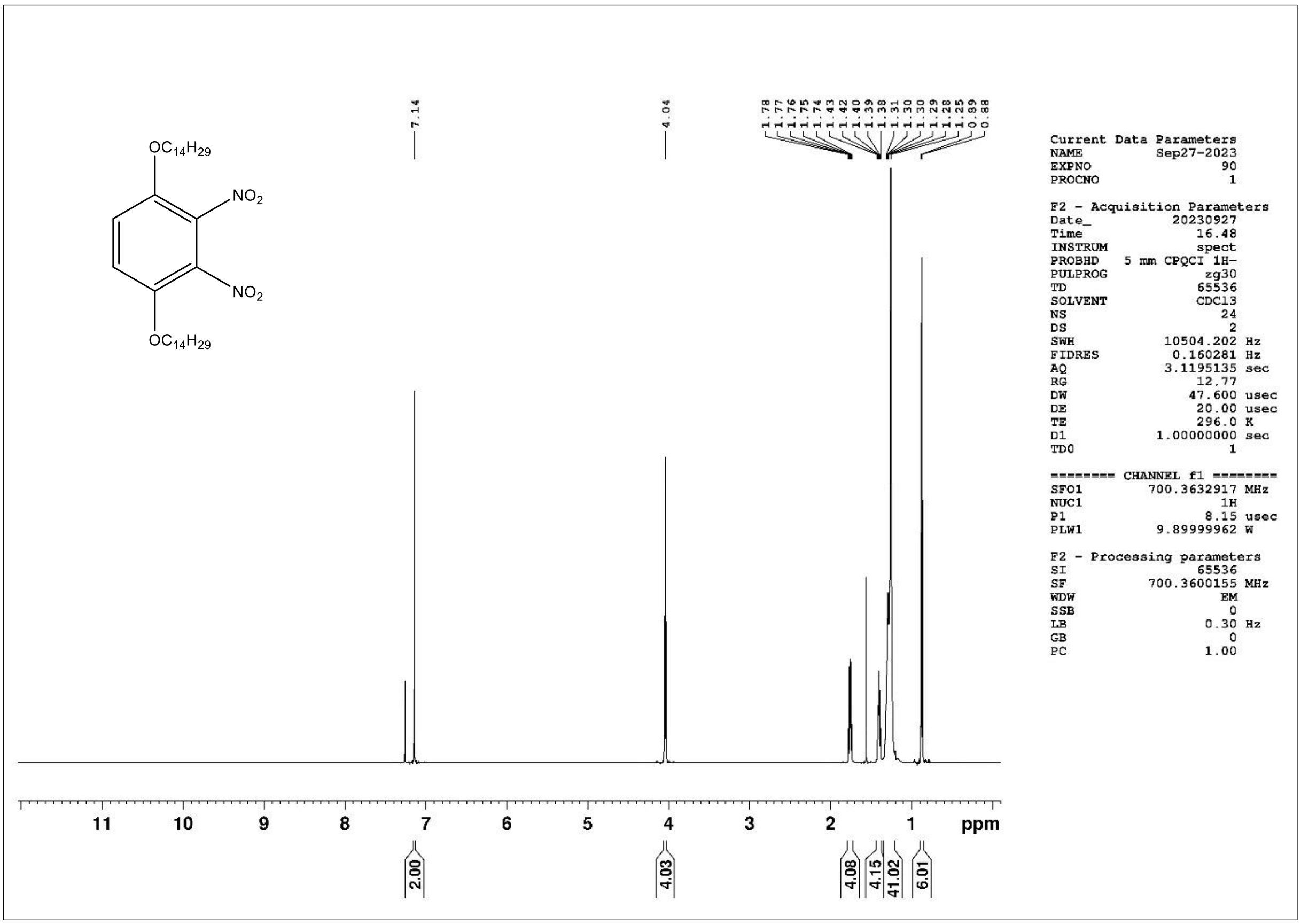

Figure S56. ^{1}H NMR of **L-Nitro(14)**.

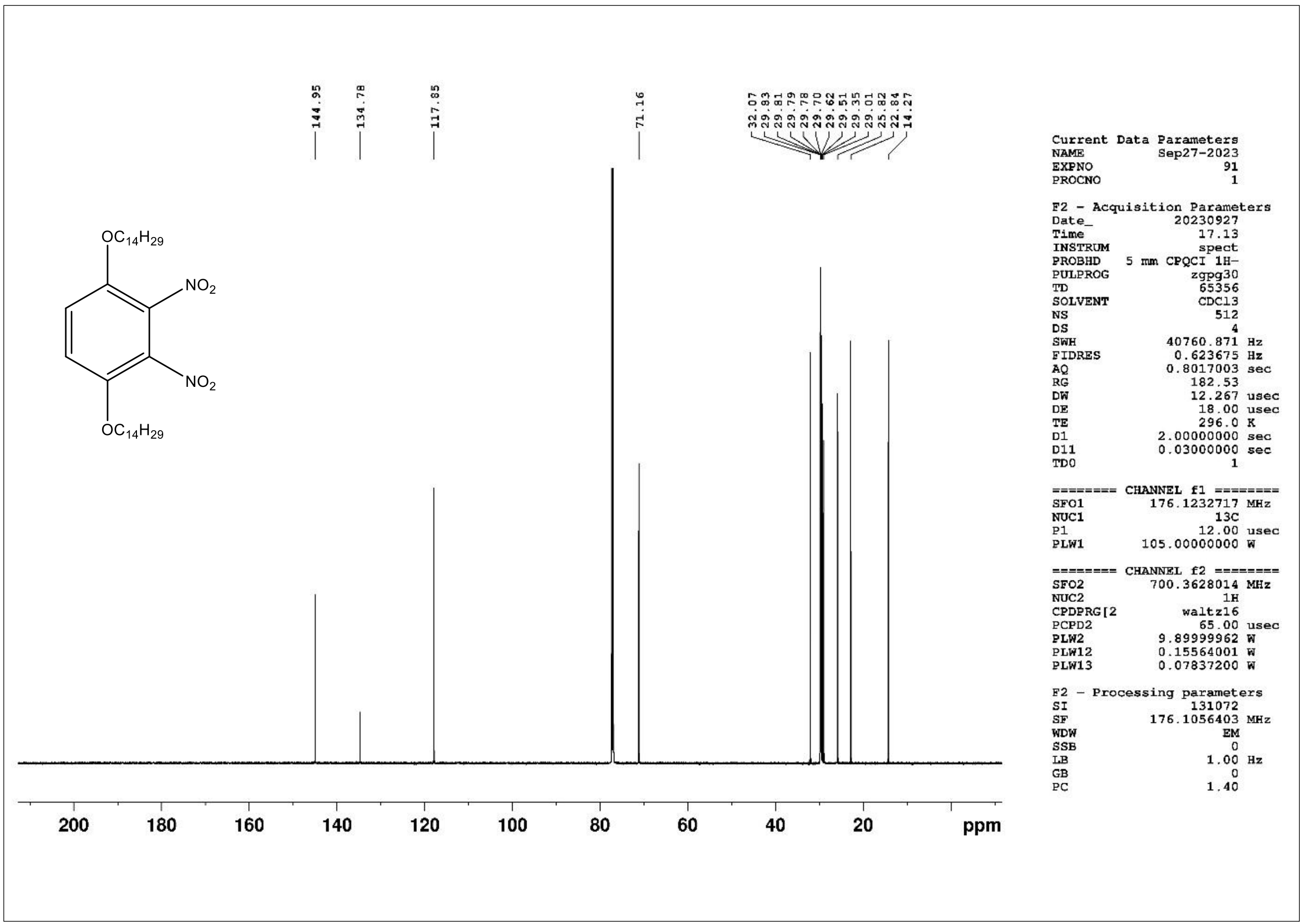

Figure S57. ^{13}C NMR of **L-Nitro(14)**.

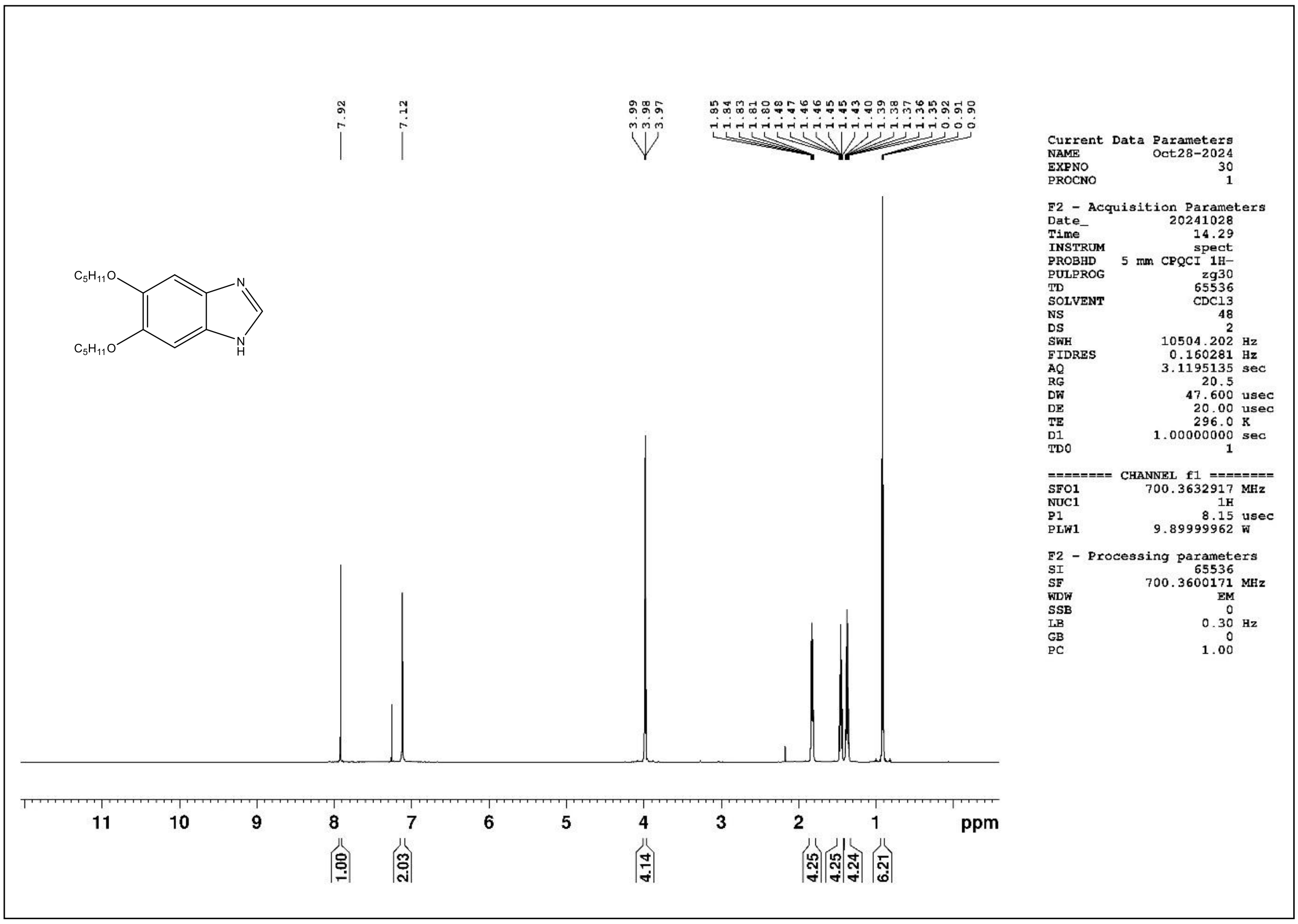

Figure S58. ^{1}H NMR of **U-Bi(5)**.

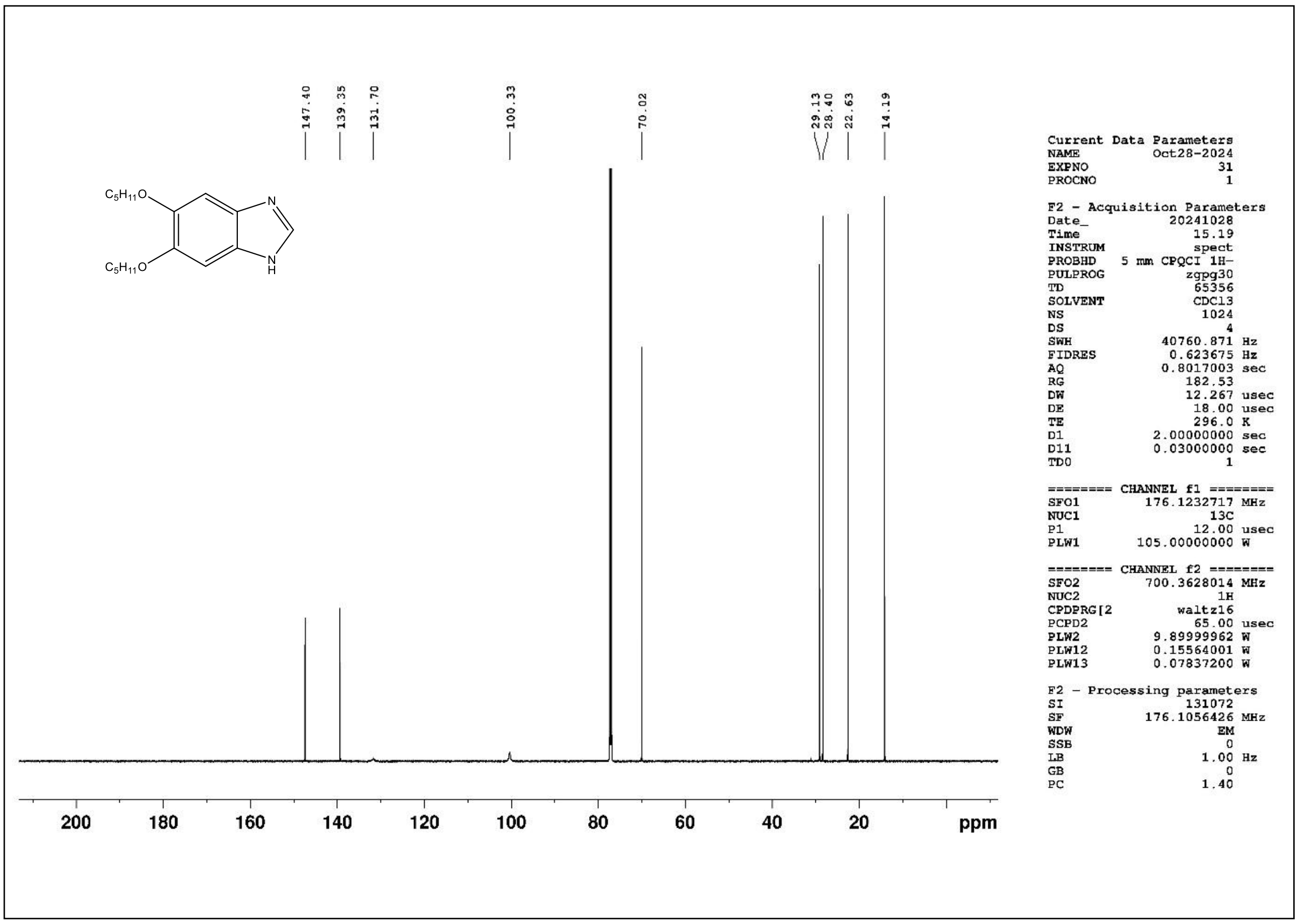

Figure S59. ^{13}C NMR of **U-Bi(5)**.

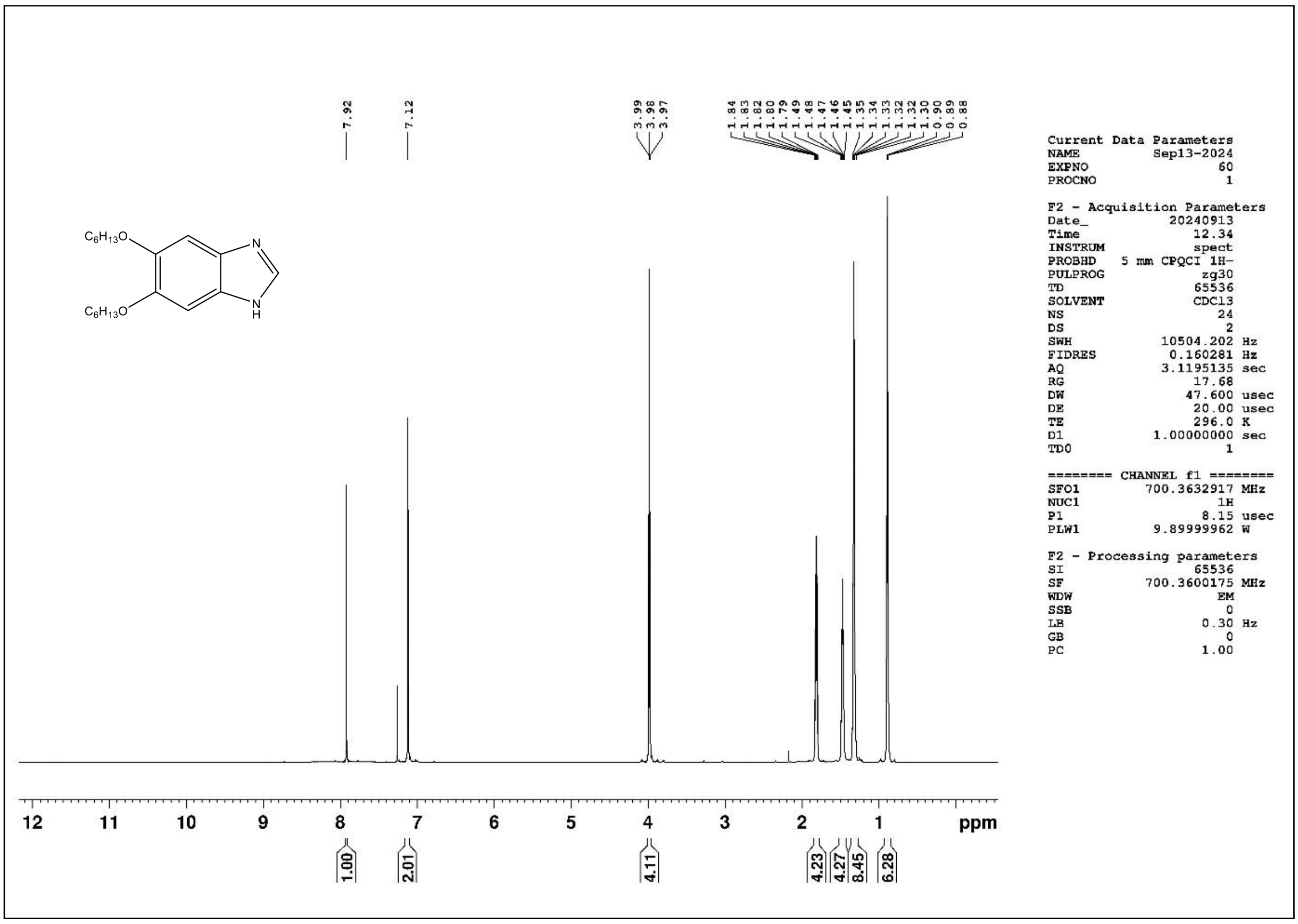

Figure S60. ^{1}H NMR of **U-Bi(6)**.

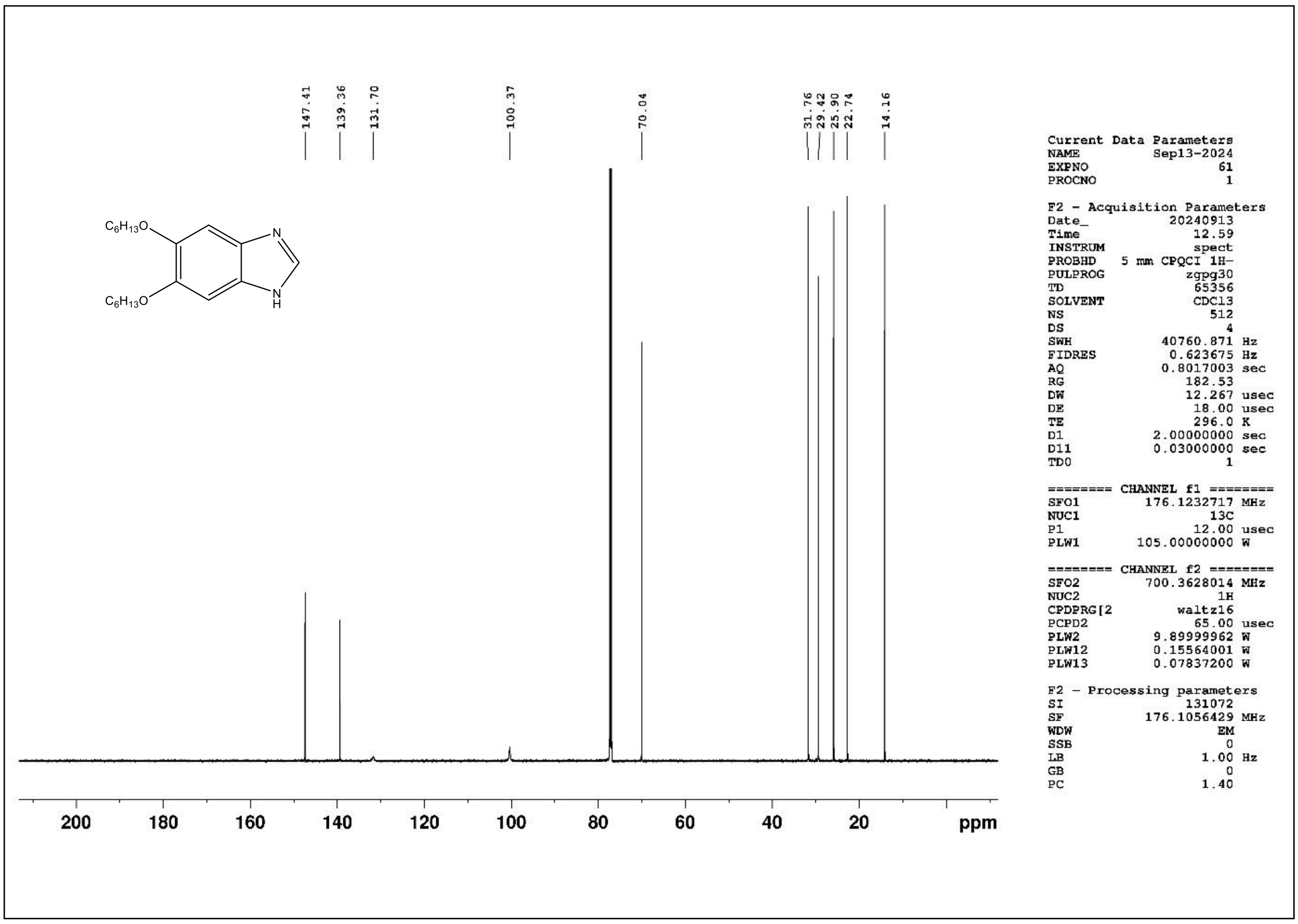

Figure S61. ^{13}C NMR of **U-Bi(6)**.

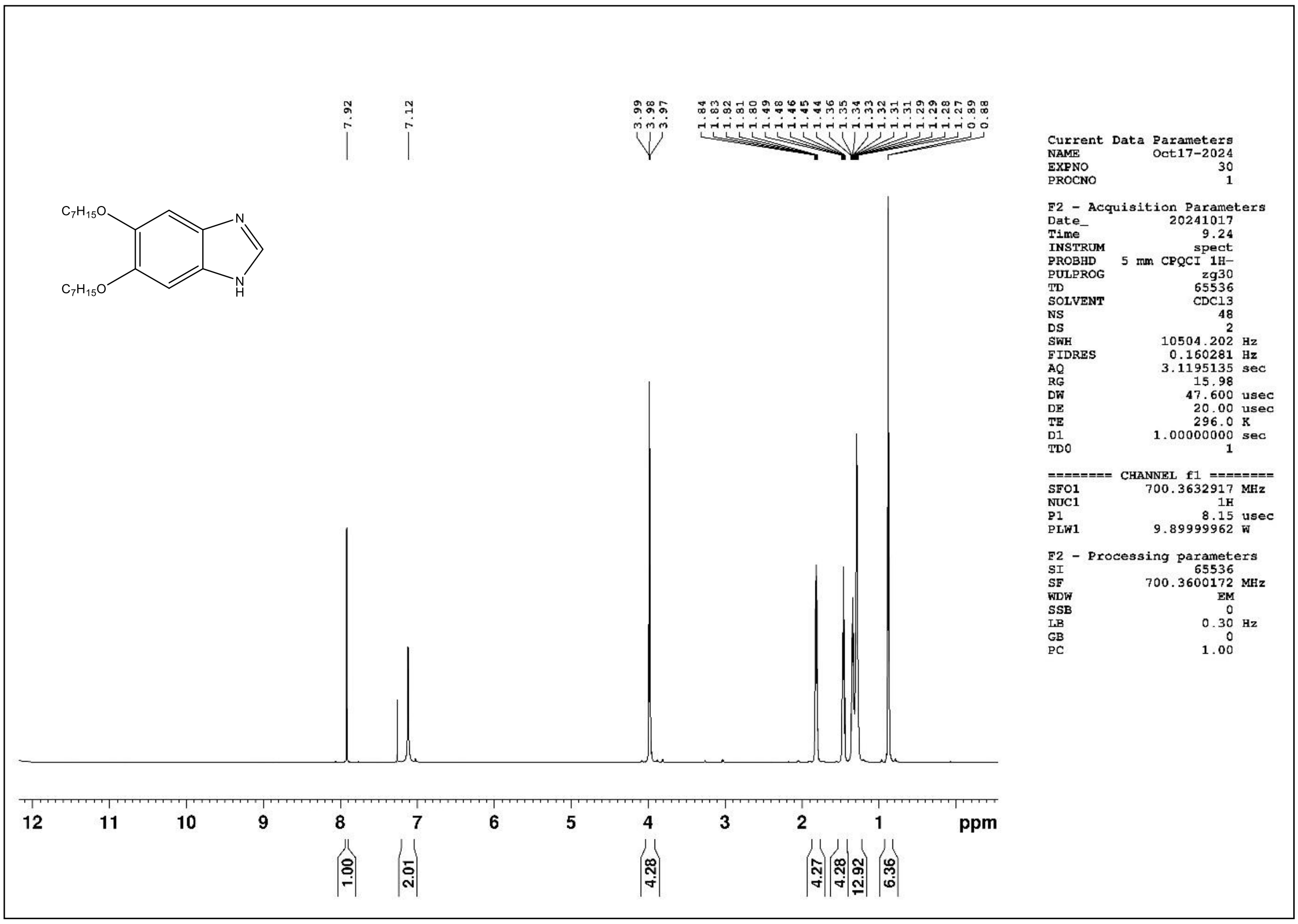

Figure S62. ^{1}H NMR of **U-Bi(7)**.

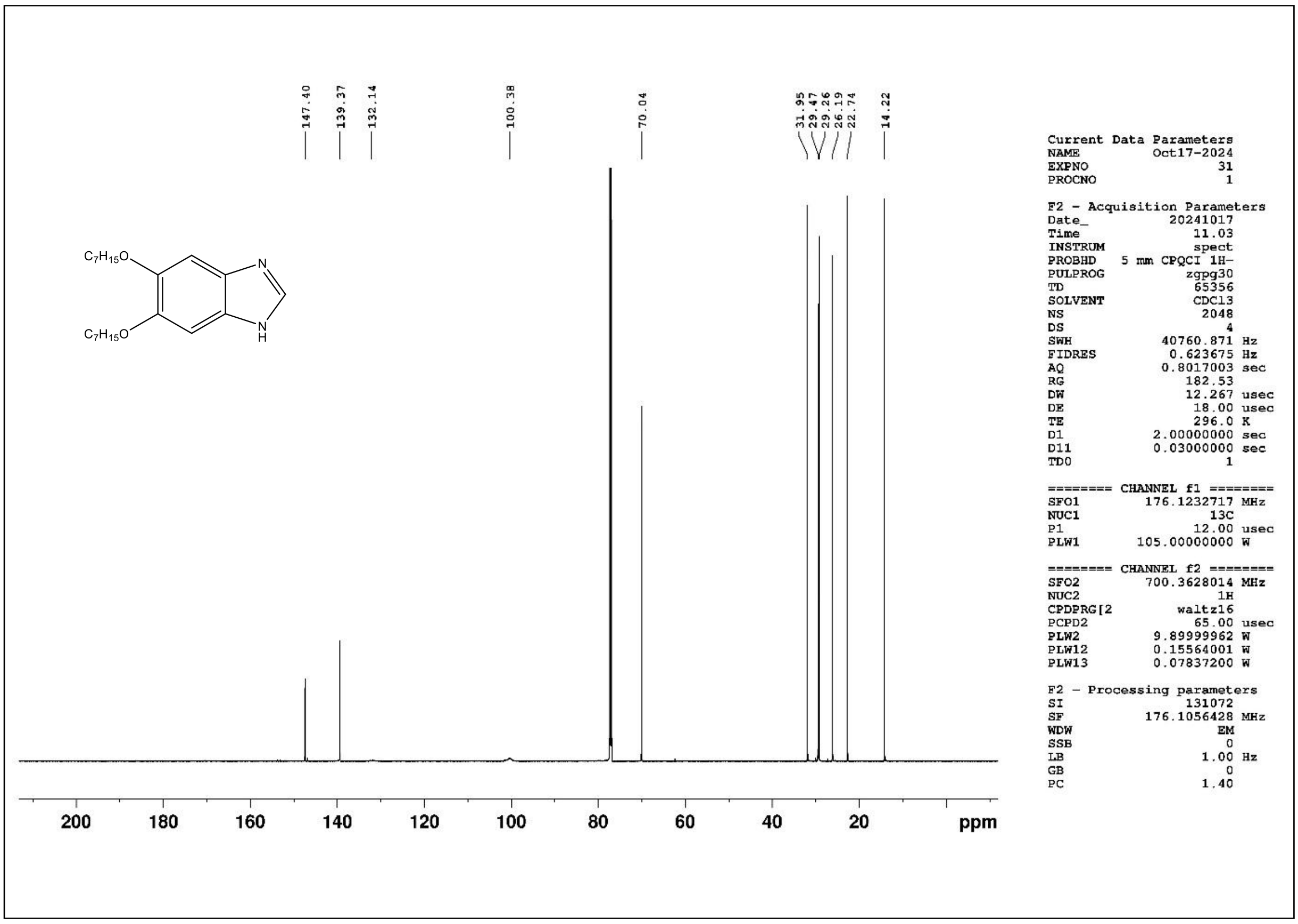

Figure S63. ^{13}C NMR of **U-Bi(7)**.

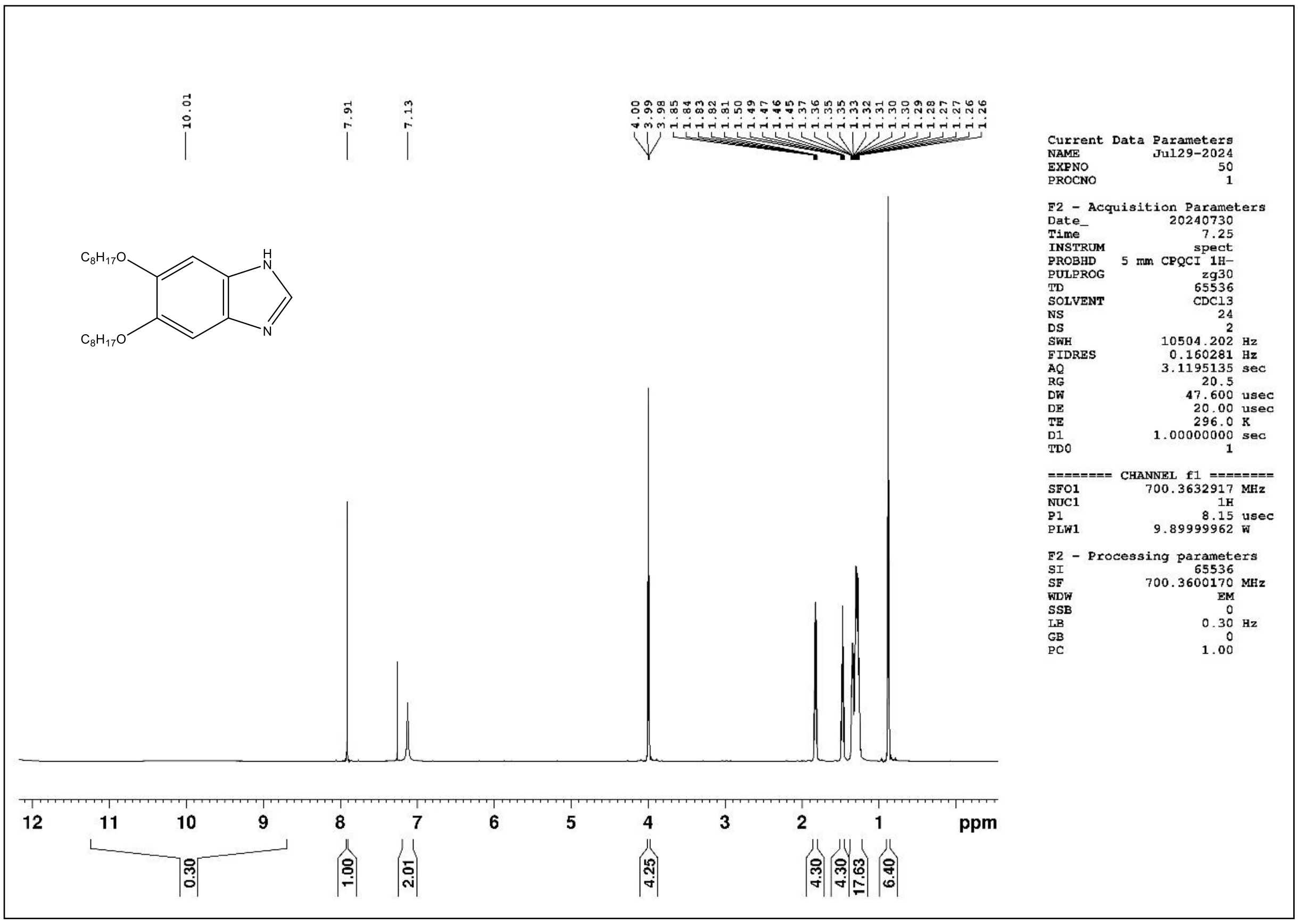

Figure S64. ^{1}H NMR of **U-Bi(8)**.

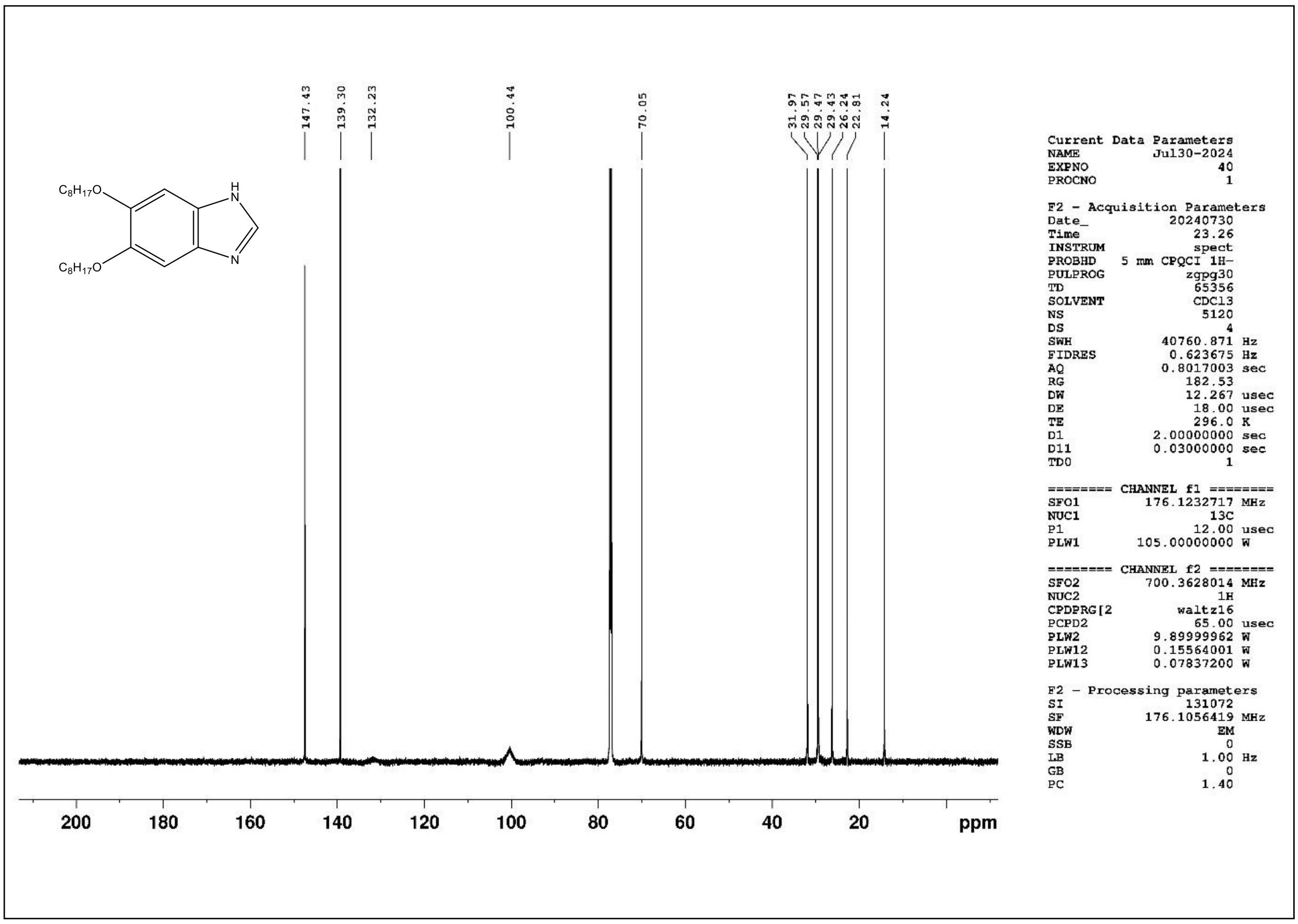

Figure S65. ^{13}C NMR of **U-Bi(8)**.

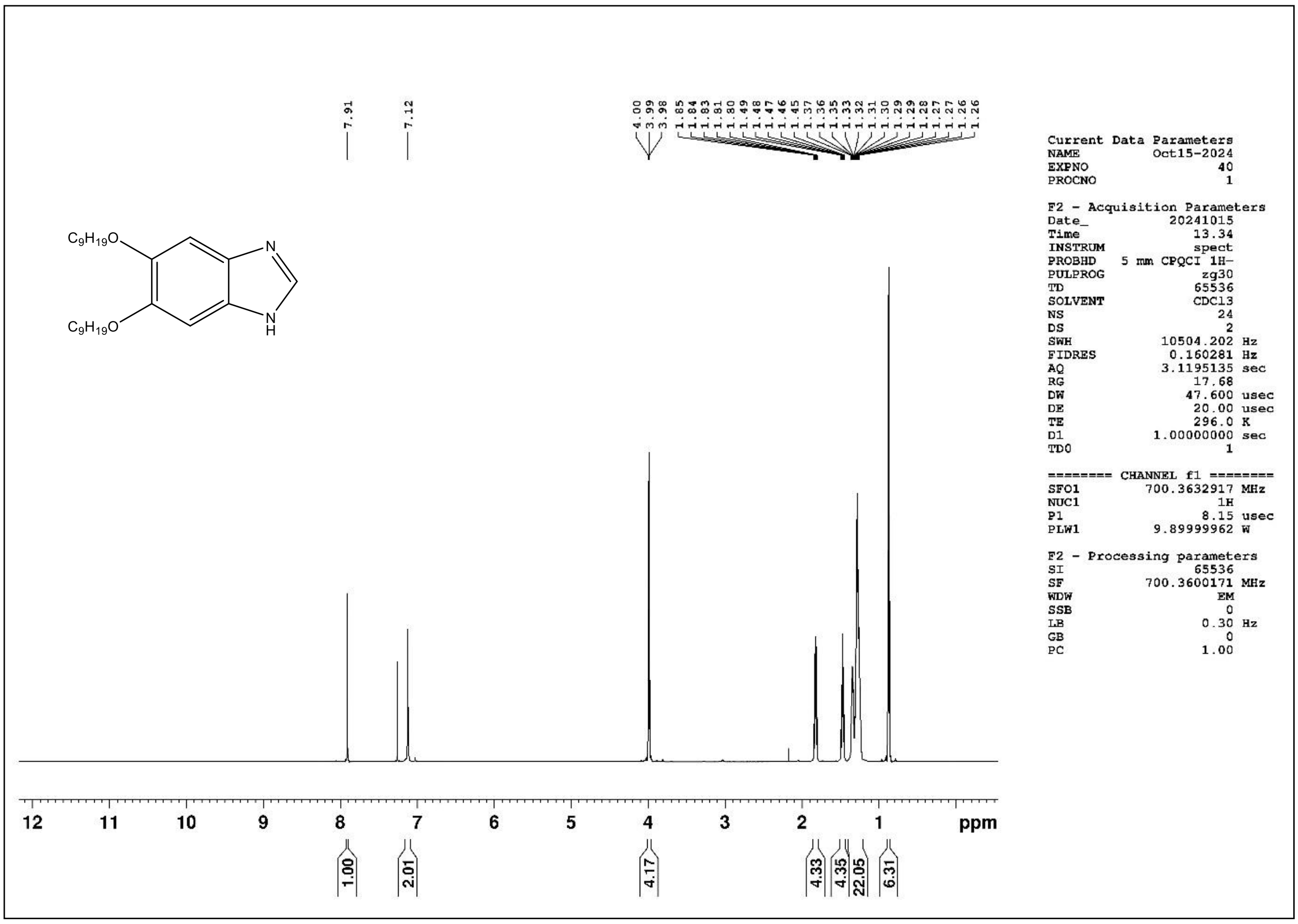

Figure S66. ^{1}H NMR of **U-Bi(9)**.

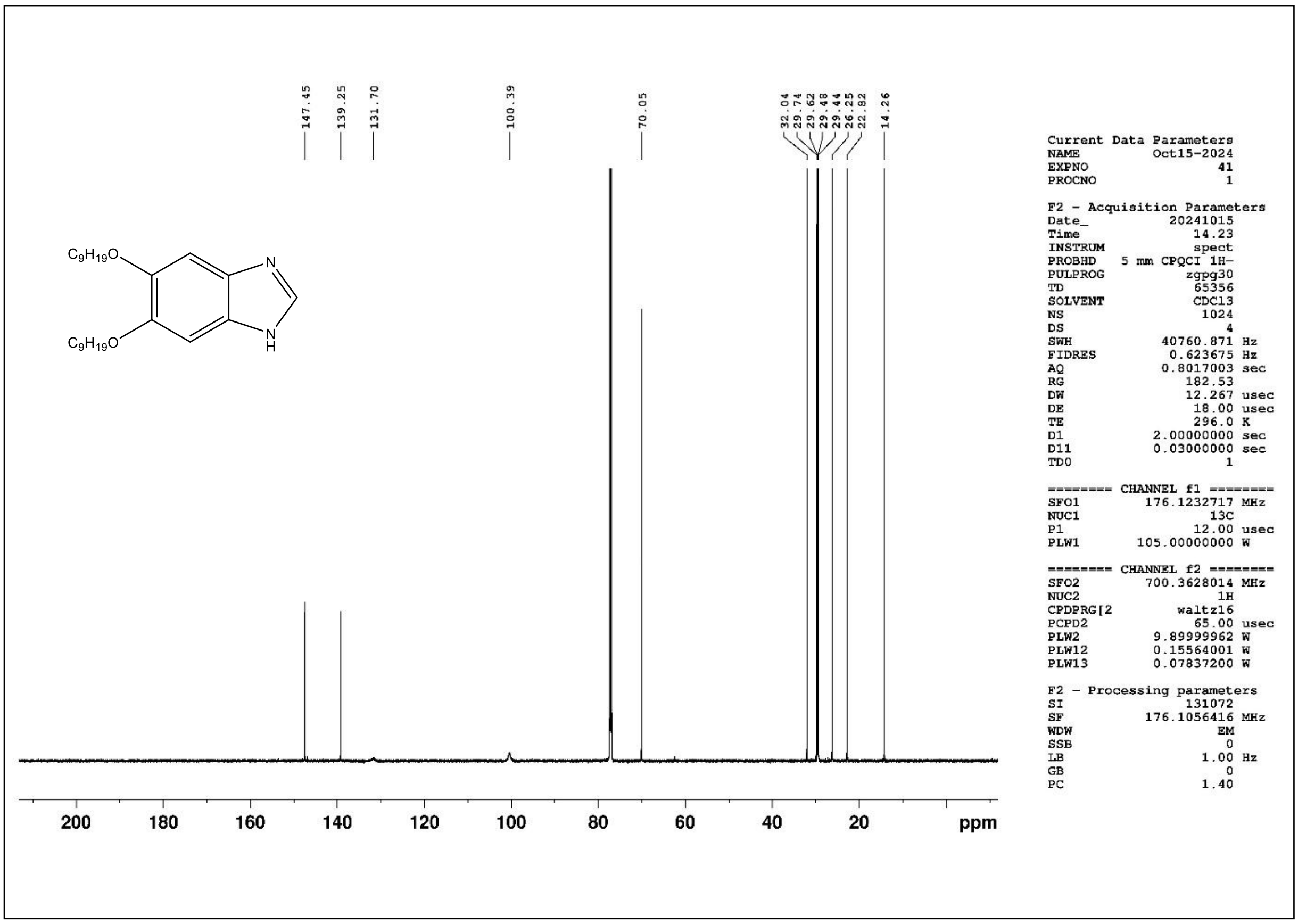

Figure S67. ^{13}C NMR of **U-Bi(9)**.

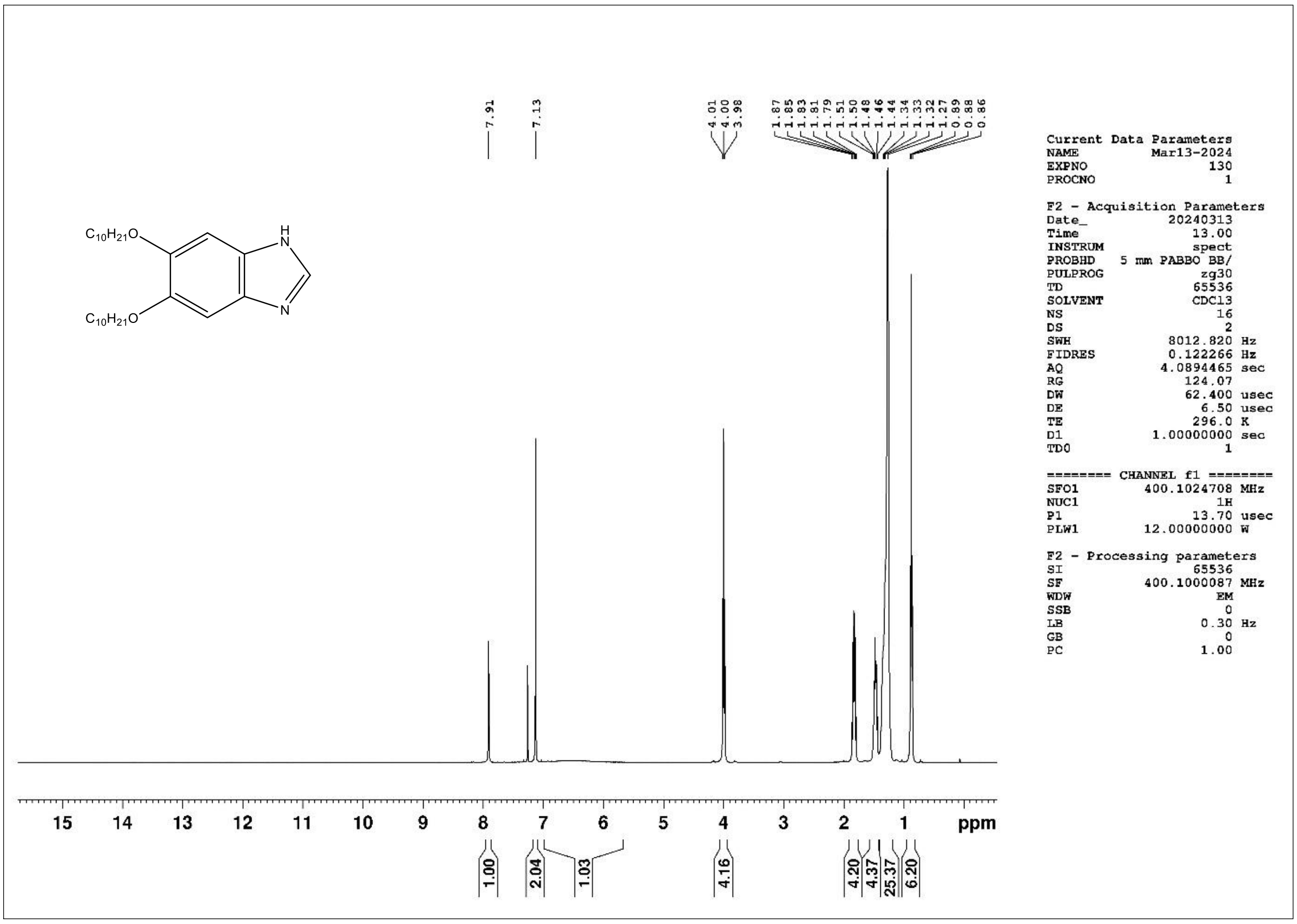

Figure S68. ^{1}H NMR of **U-Bi(10)**.

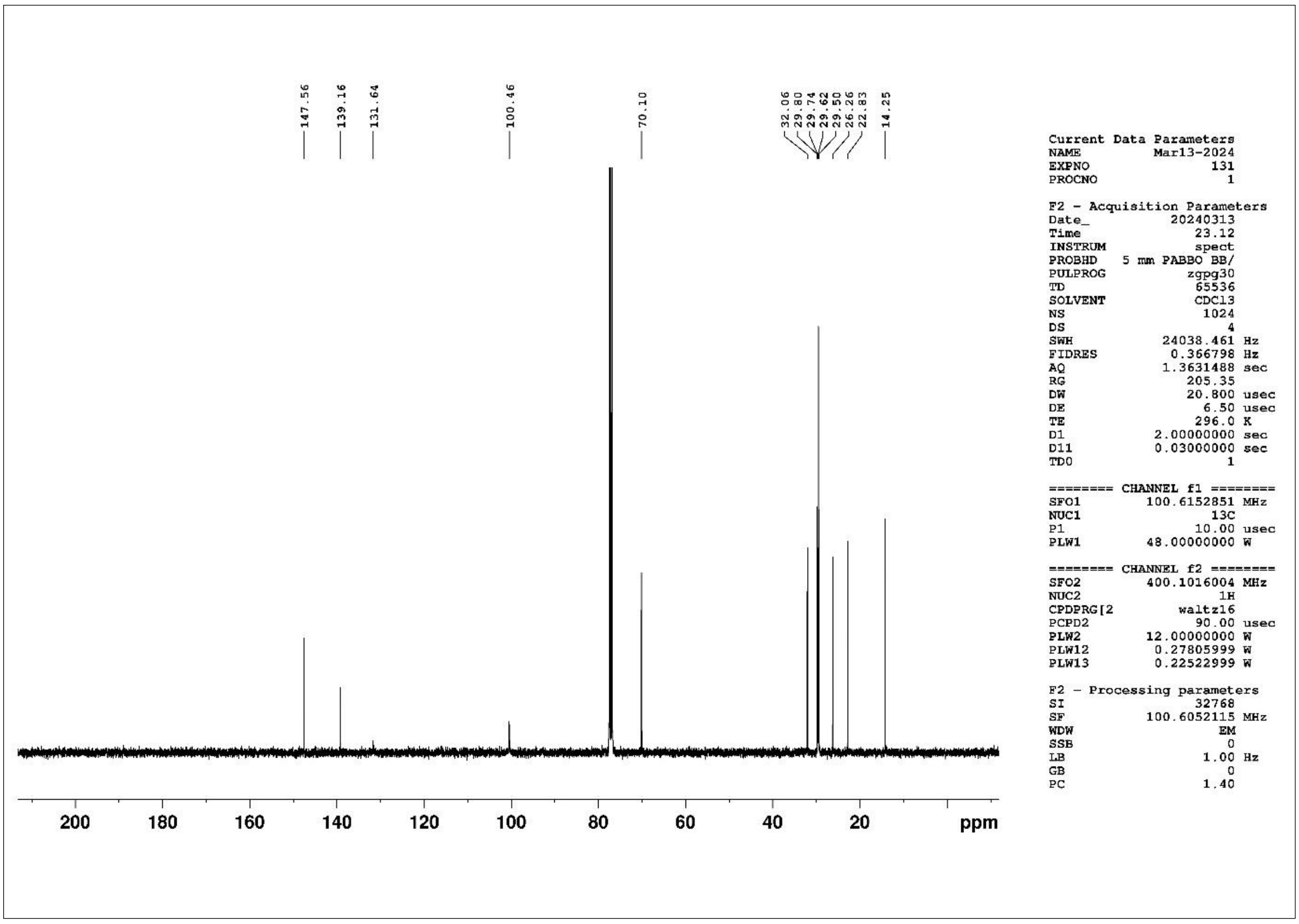

Figure S69. ^{13}C NMR of **U-Bi(10)**.

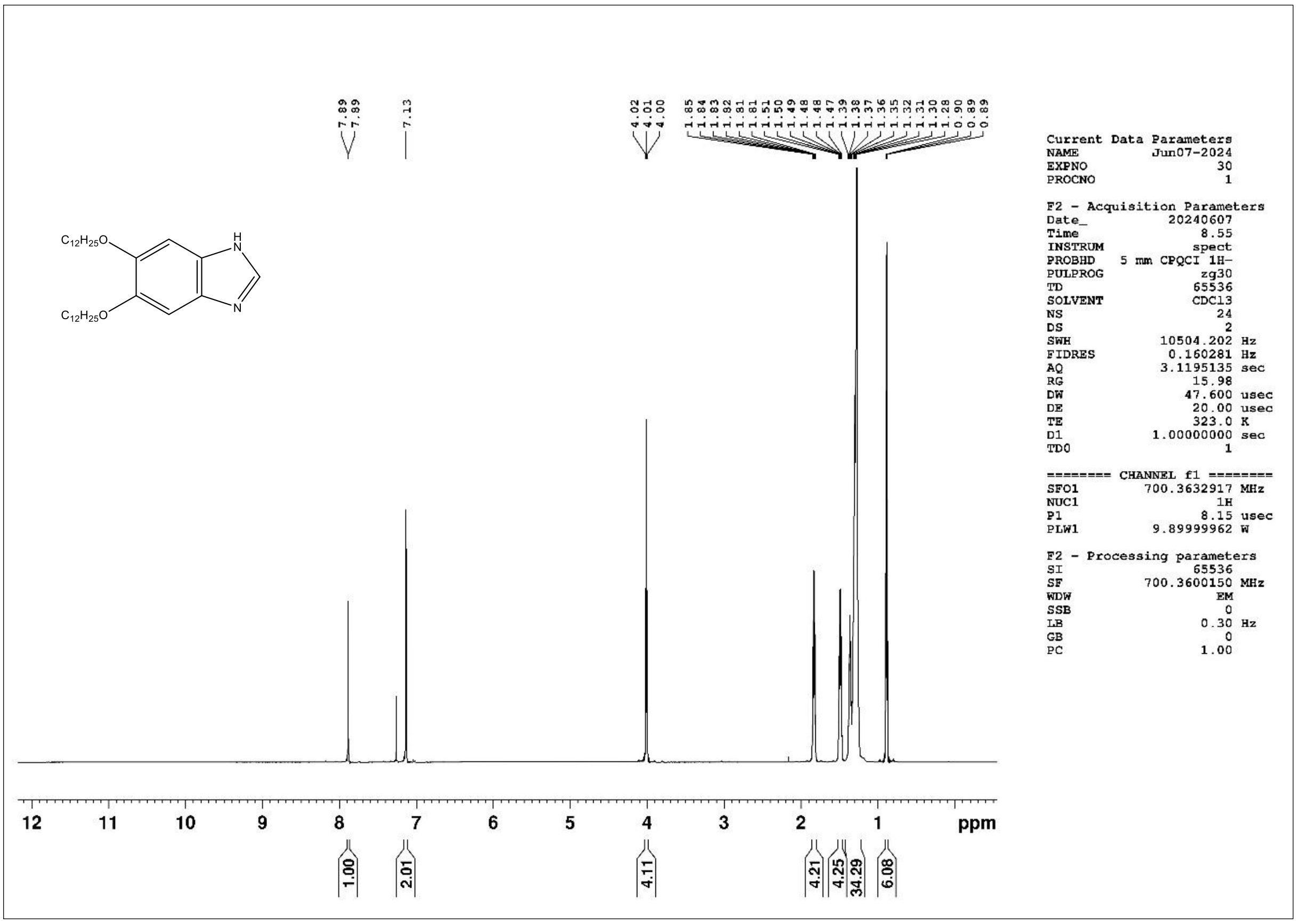

Figure S70. ^{1}H NMR of **U-Bi(12)**.

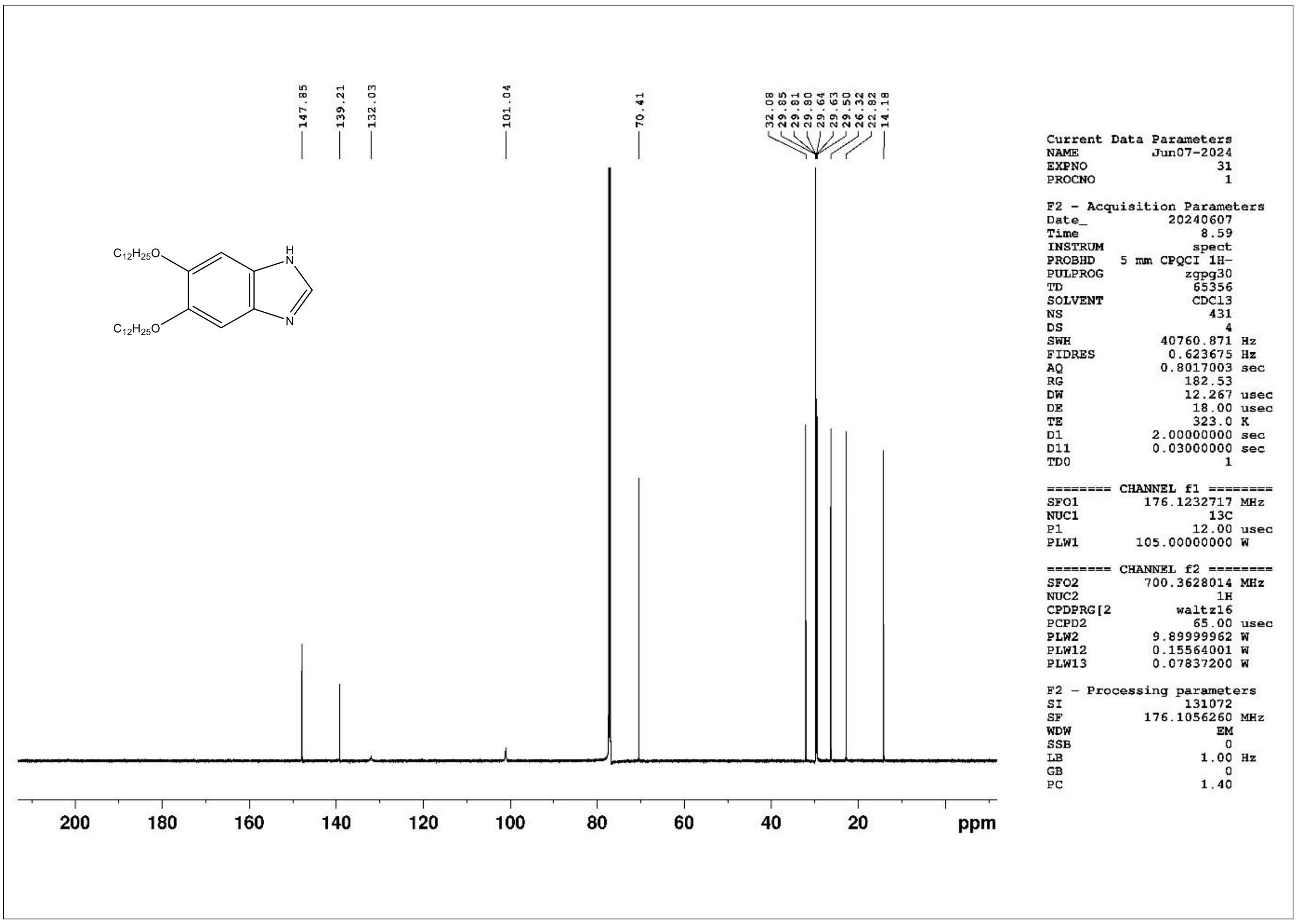

Figure S71. ^{13}C NMR of **U-Bi(12)**.

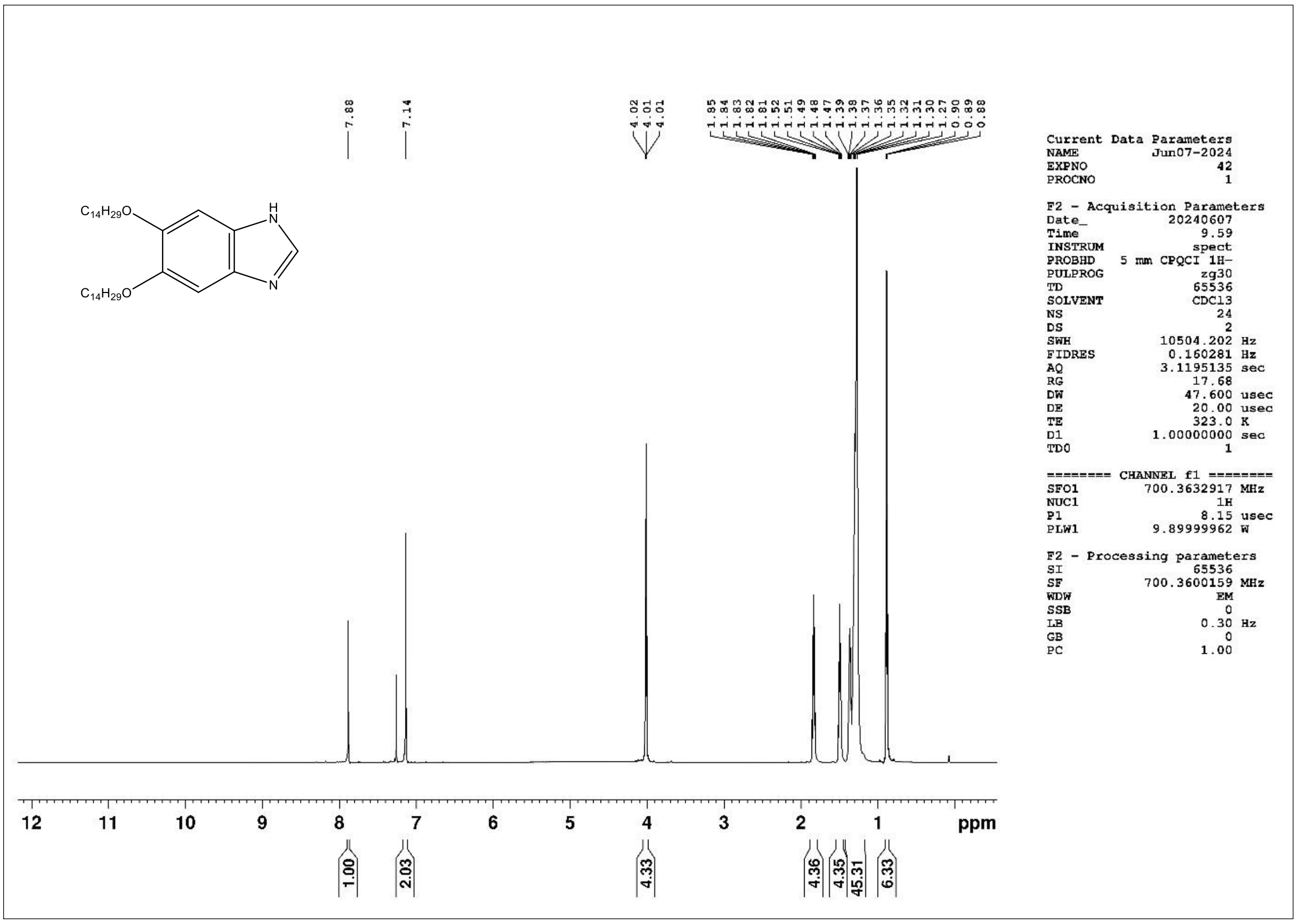

Figure S72. ^{1}H NMR of **U-Bi(14)**.

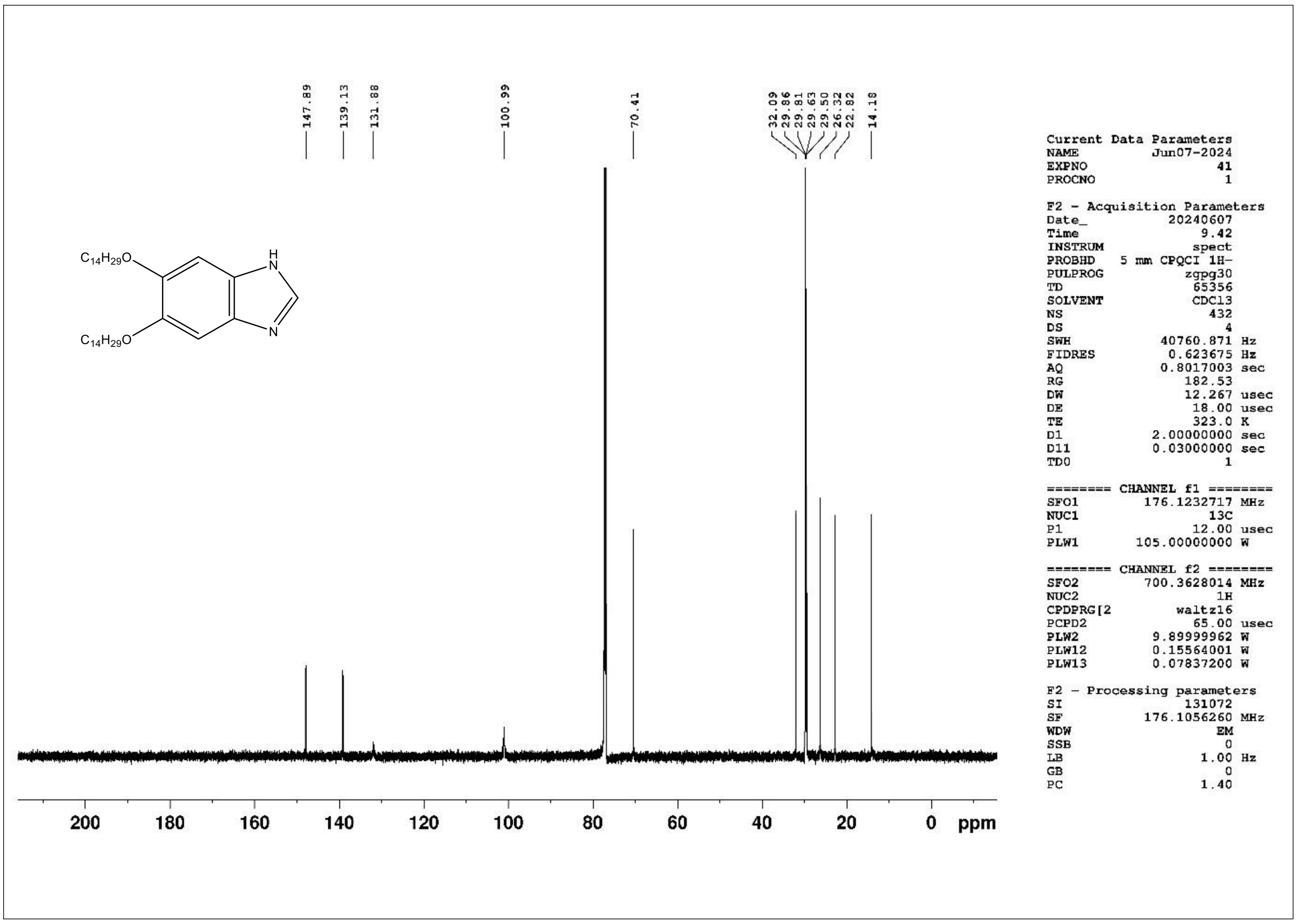

Figure S73. ^{13}C NMR of **U-Bi(14)**.

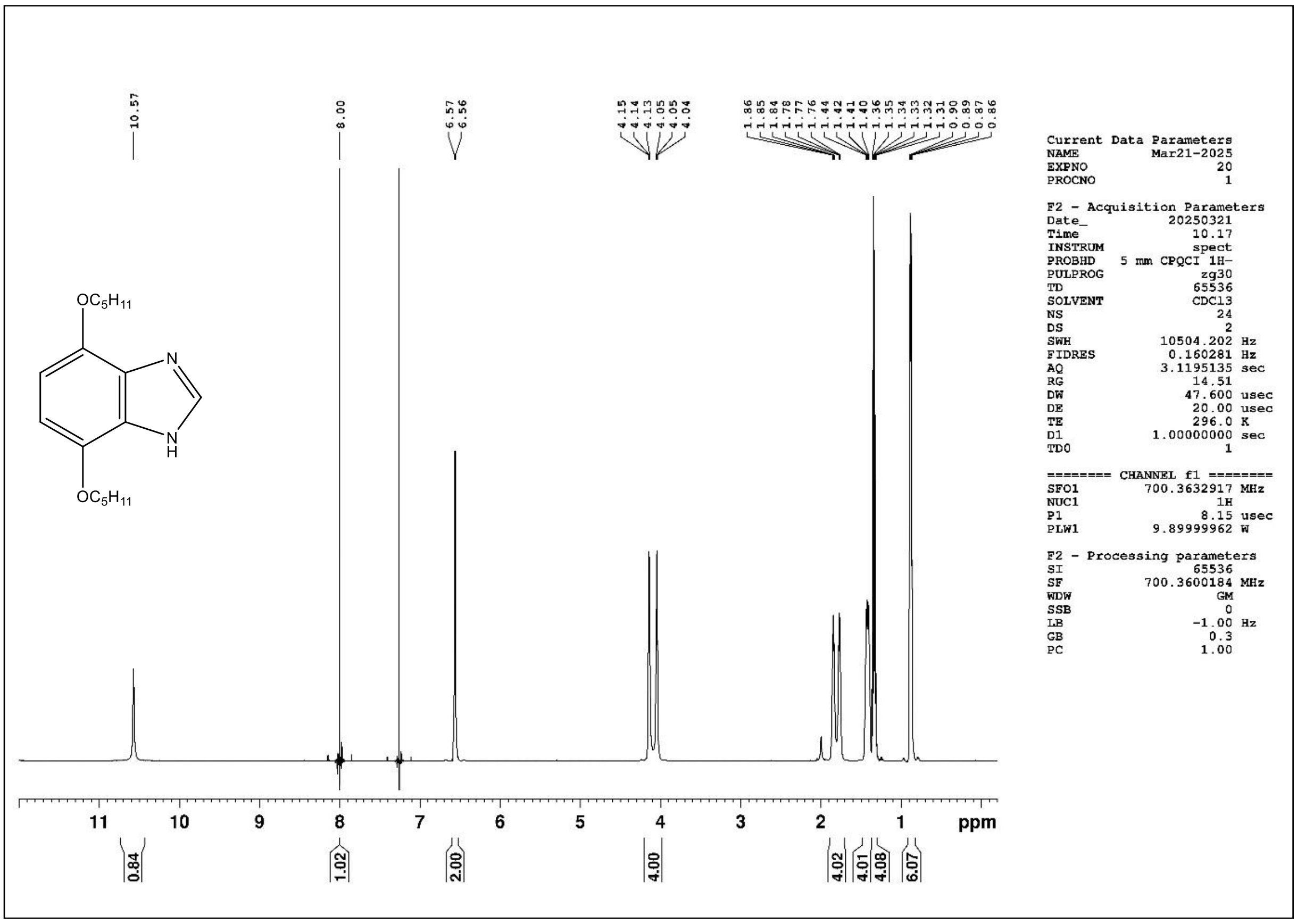

Figure S74. ^{1}H NMR of **L-Bi(5)**.

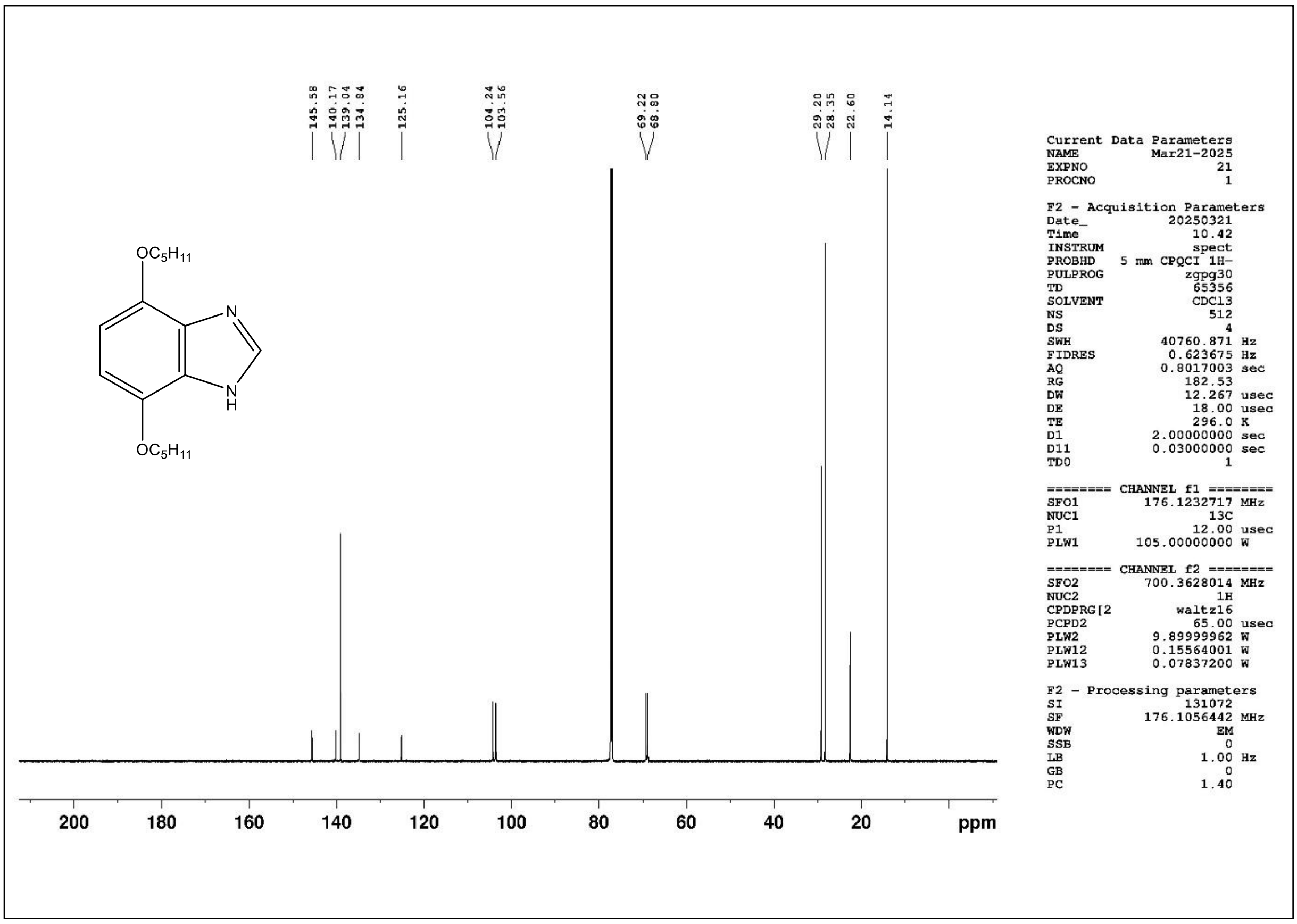

Figure S75. ^{13}C NMR of **L-Bi(5)**.

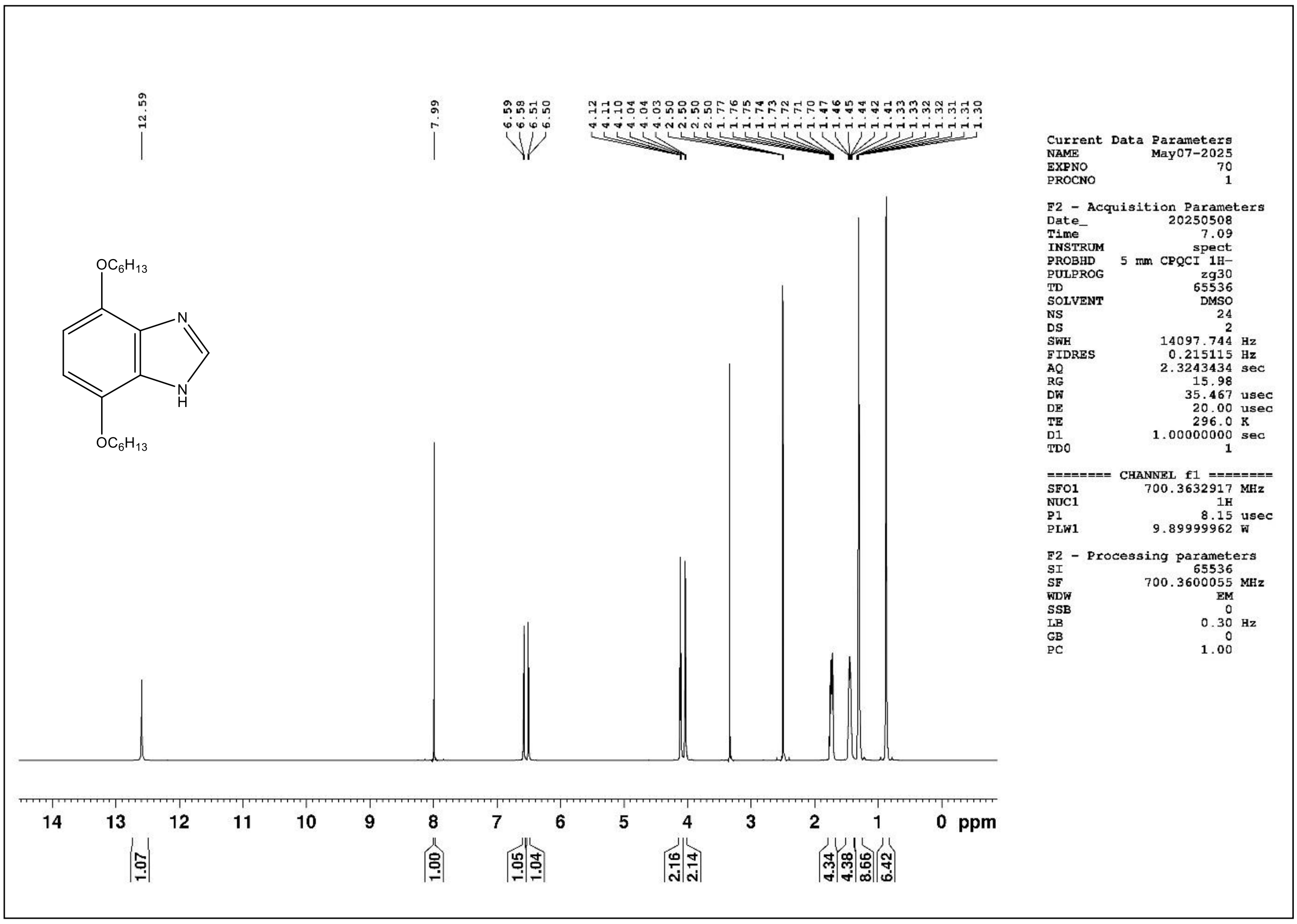

Figure S76. ^{1}H NMR of **L-Bi(6)**.

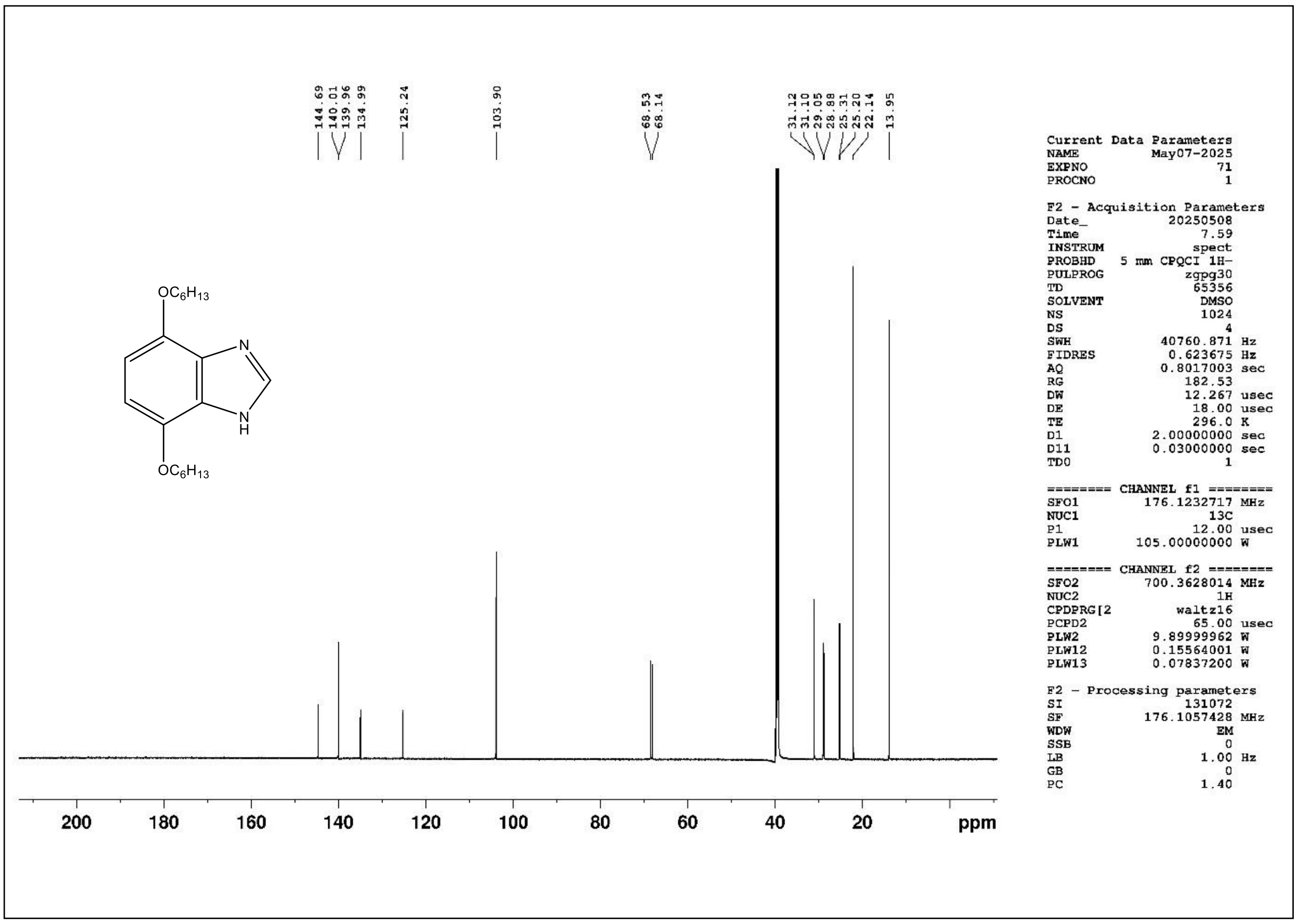

Figure S77. ^{13}C NMR of **L-Bi(6)**.

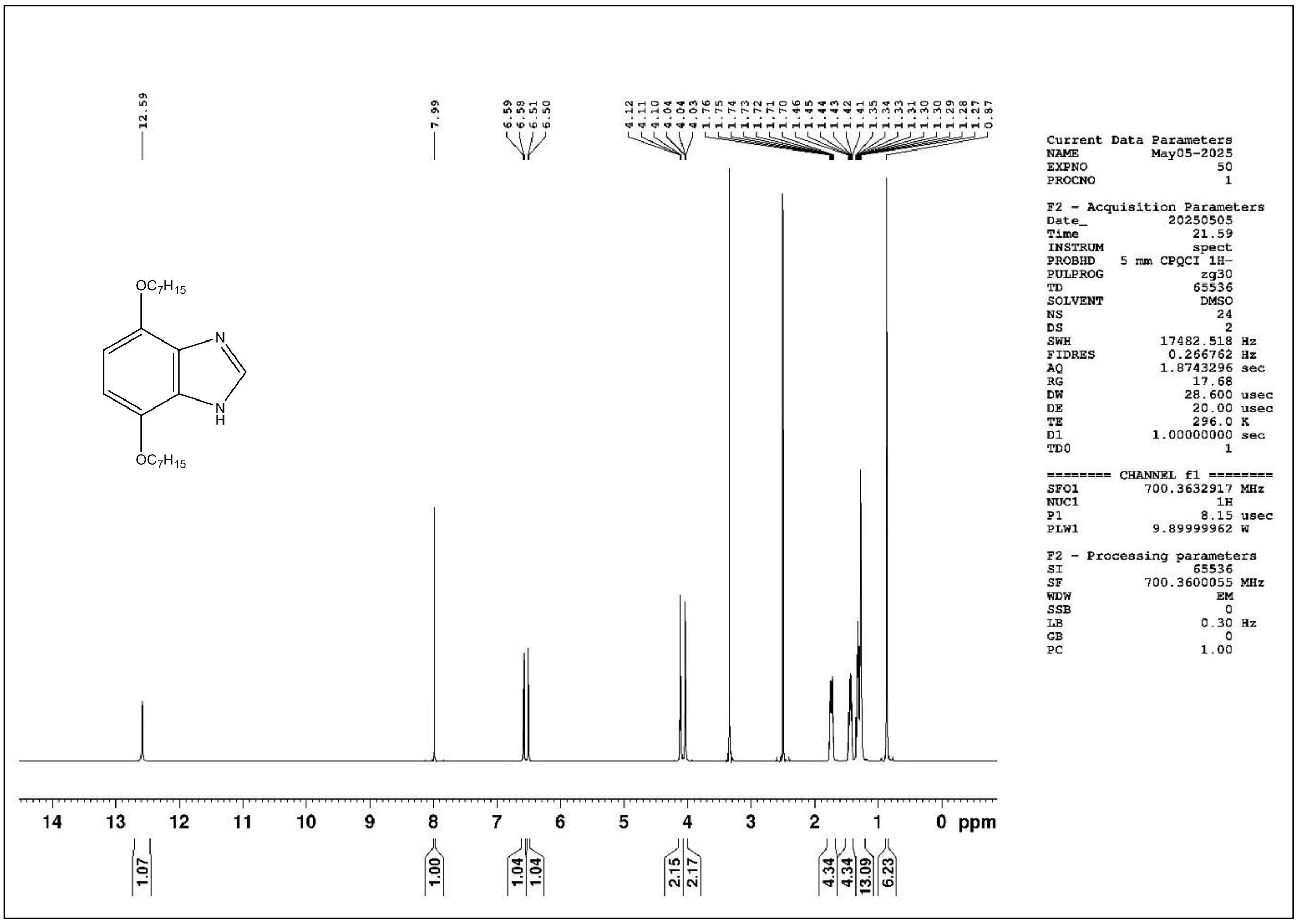

Figure S78. ^{1}H NMR of **L-Bi(7)**.

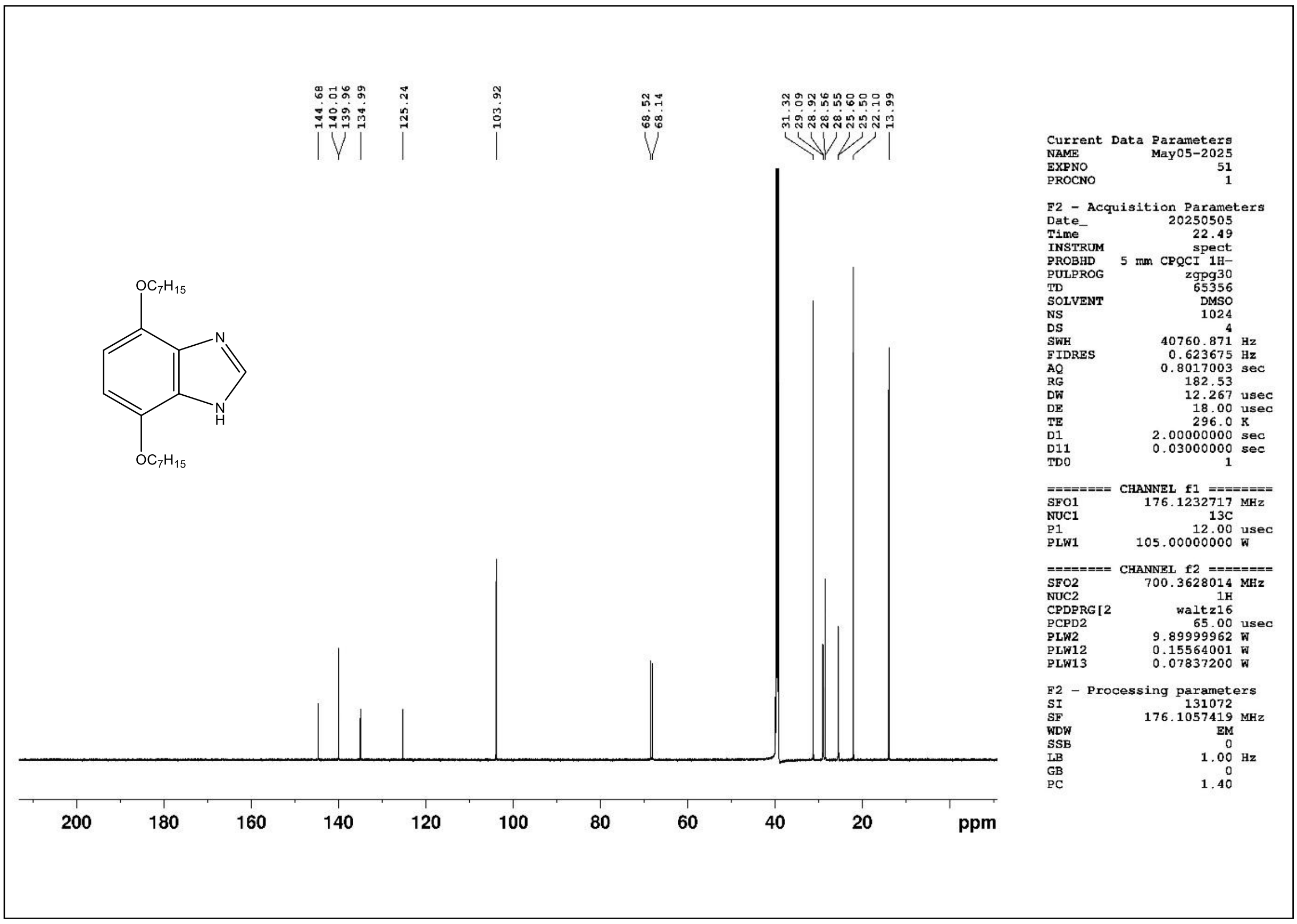

Figure S79. ^{13}C NMR of **L-Bi(7)**.

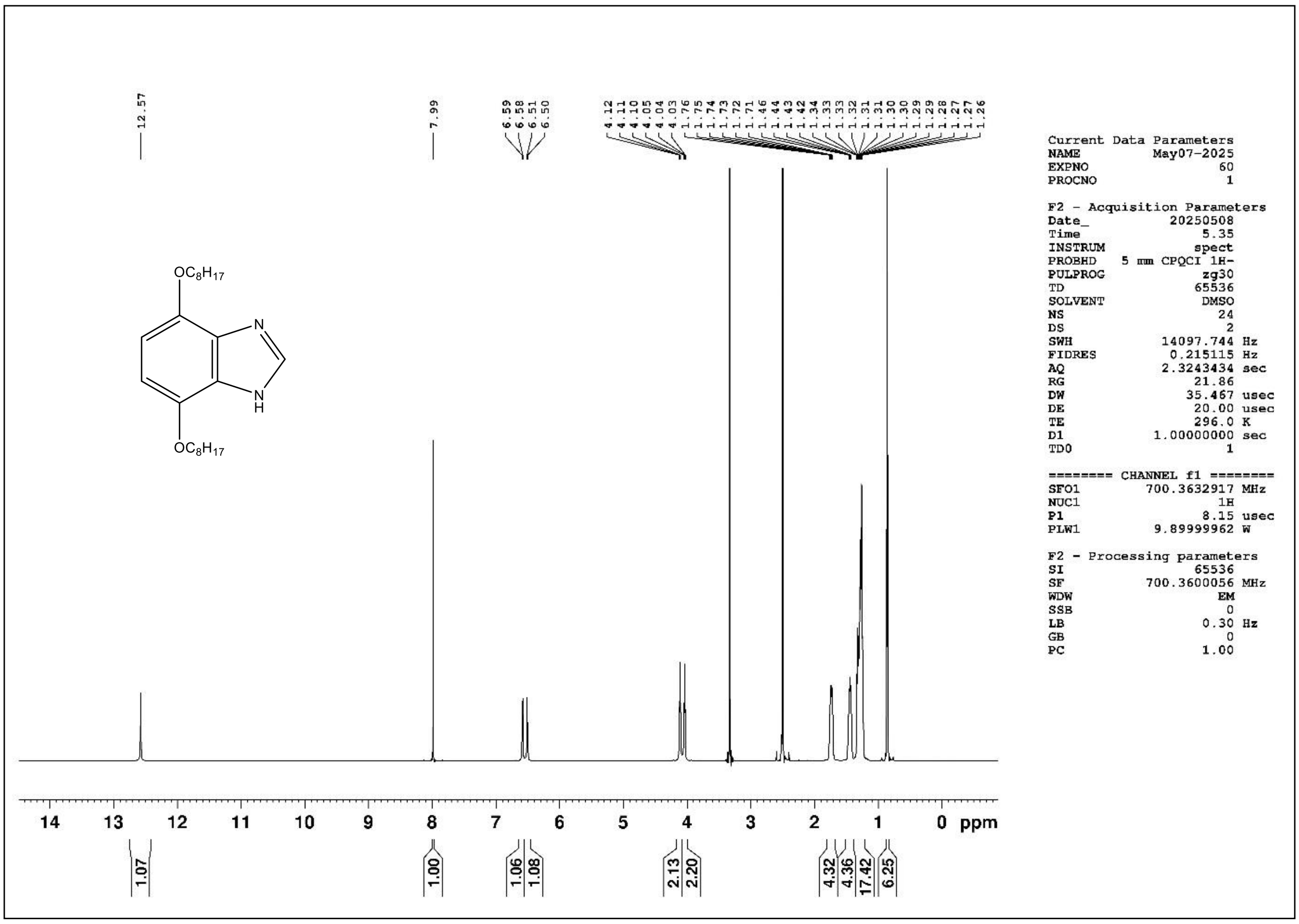

Figure S80. ^{1}H NMR of **L-Bi(8)**.

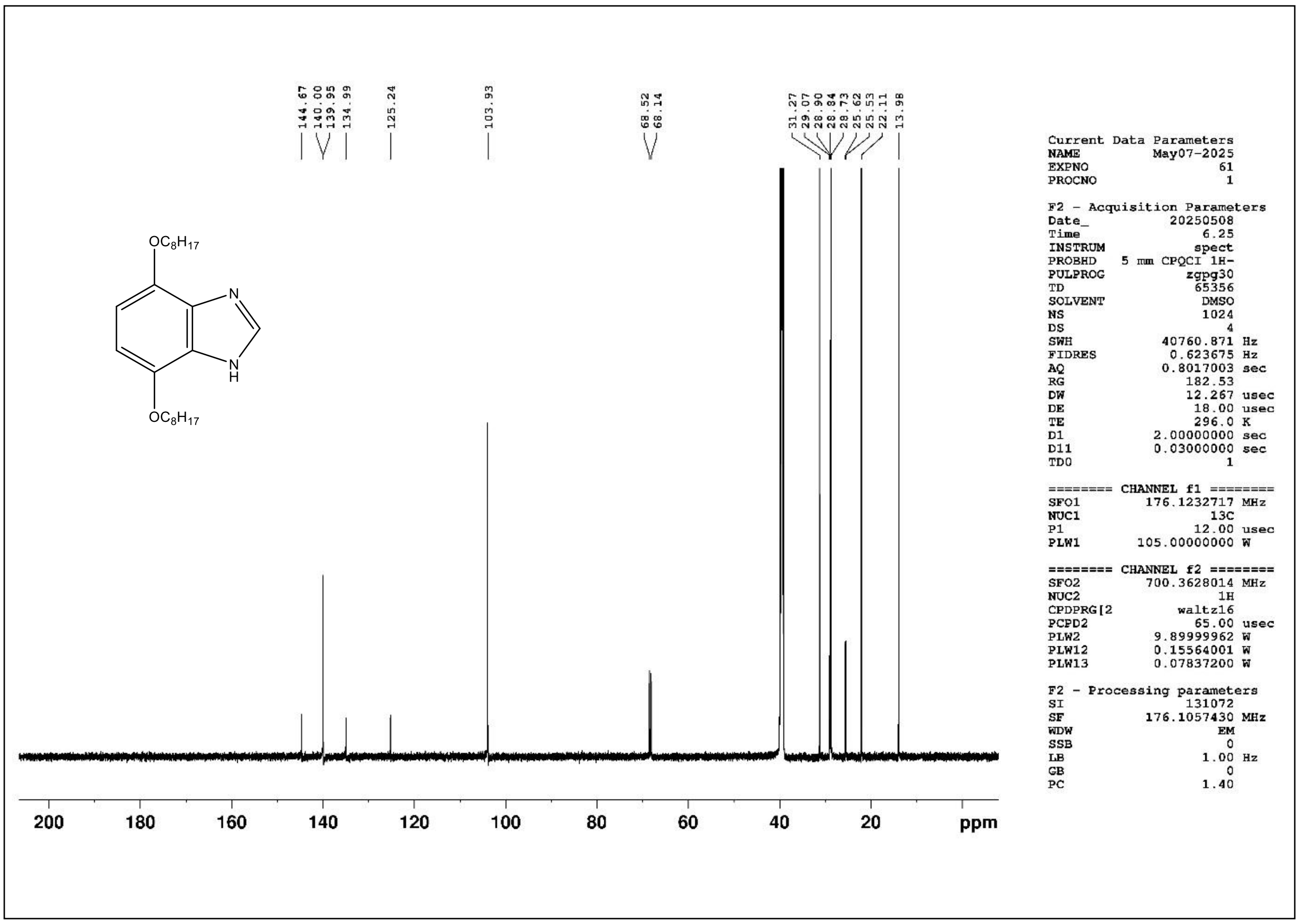

Figure S81. ^{13}C NMR of **L-Bi(8)**.

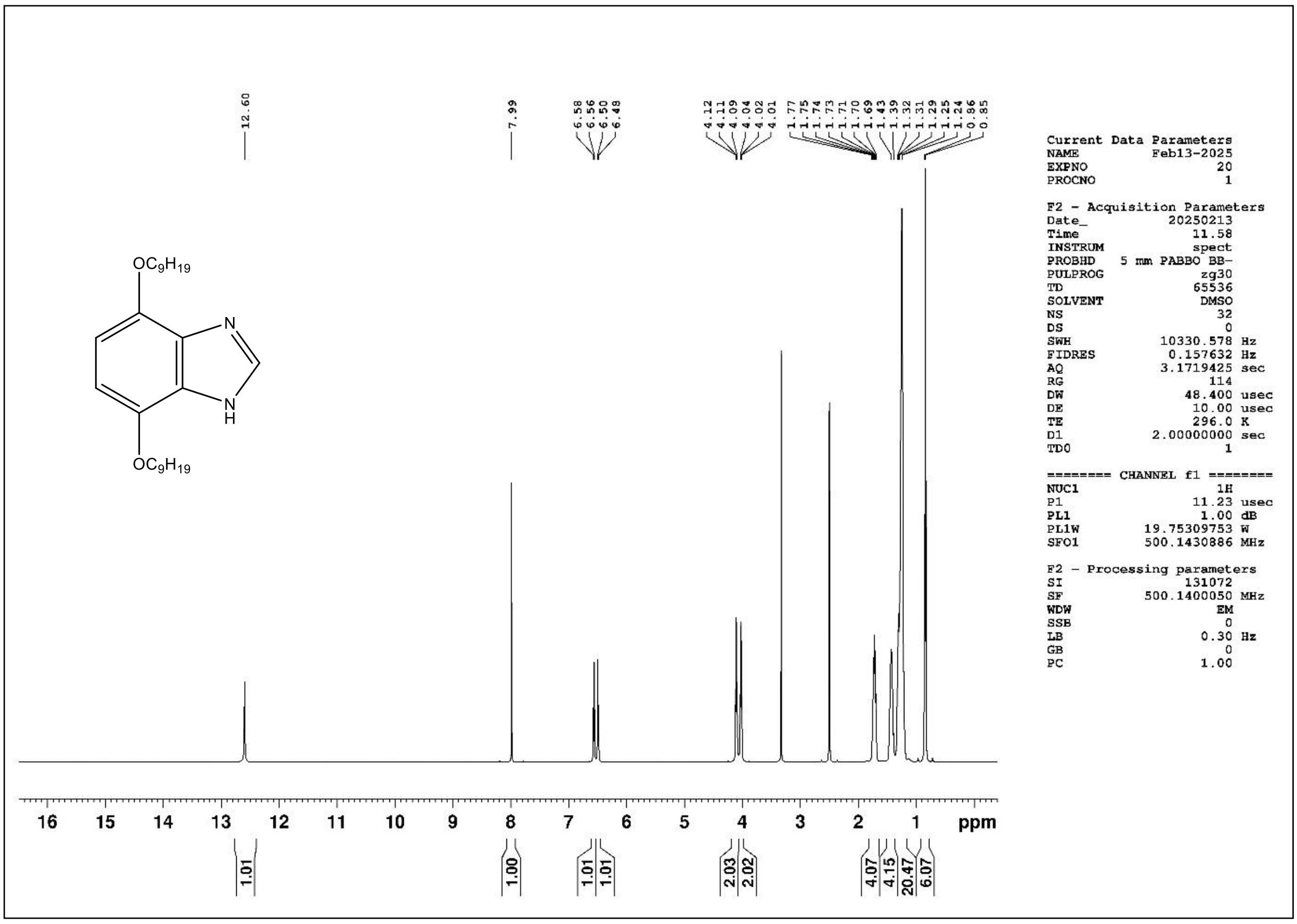

Figure S82. ^{1}H NMR of **L-Bi(9)**.

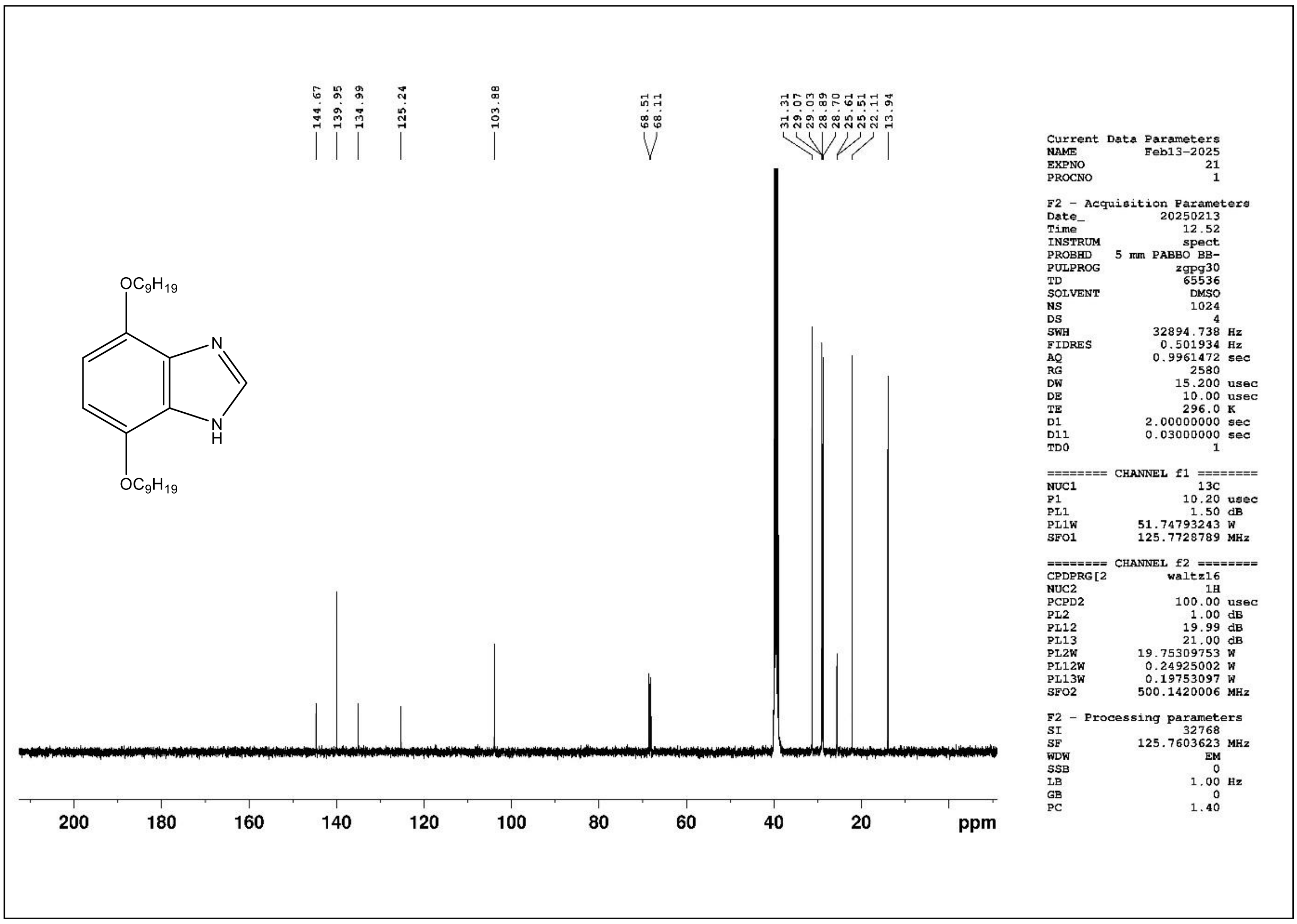

Figure S83. ^{13}C NMR of **L-Bi(9)**.

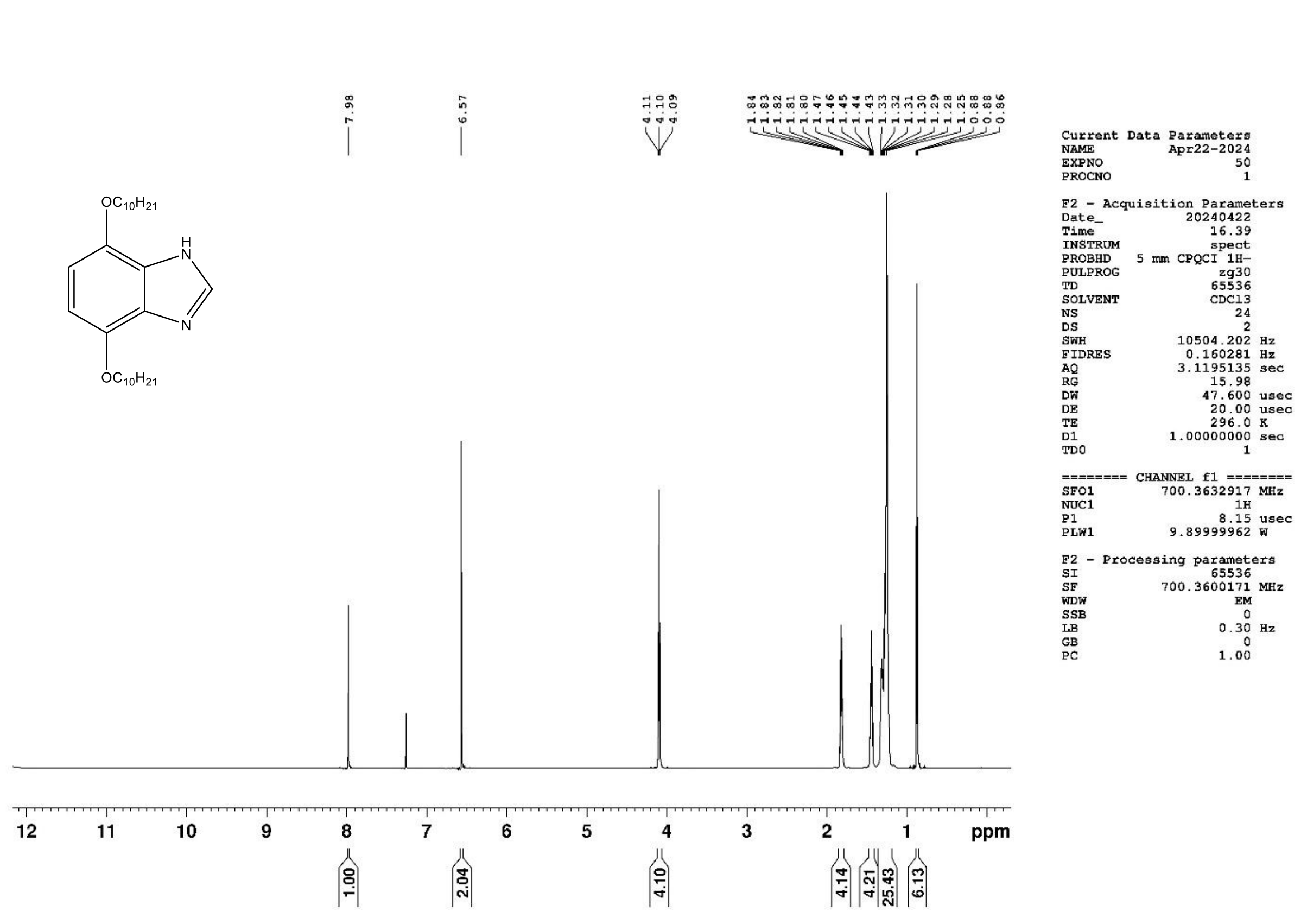

Figure S84. ^{1}H NMR of **L-Bi(10)**.

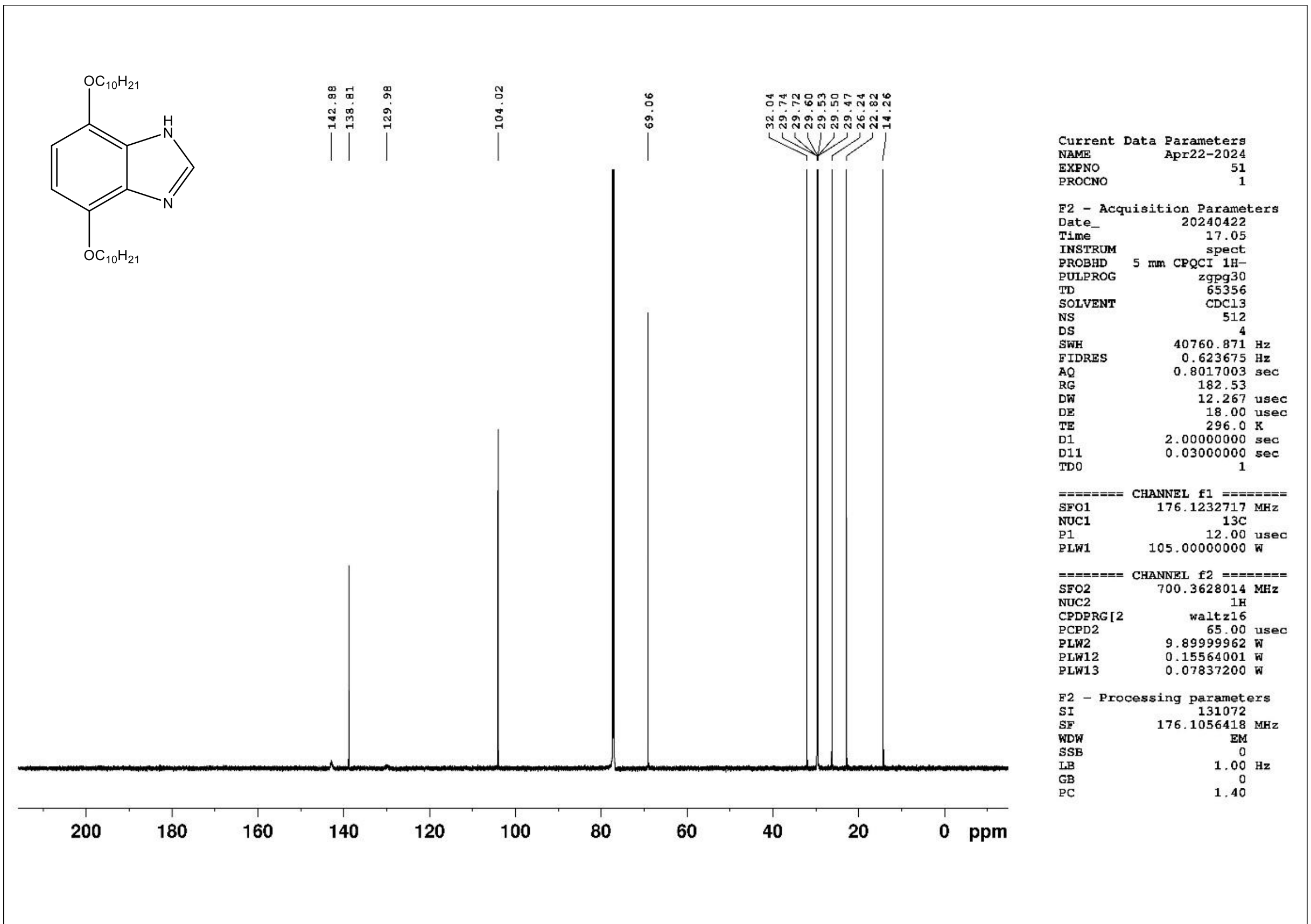

Figure S85. ^{13}C NMR of **L-Bi(10)**.

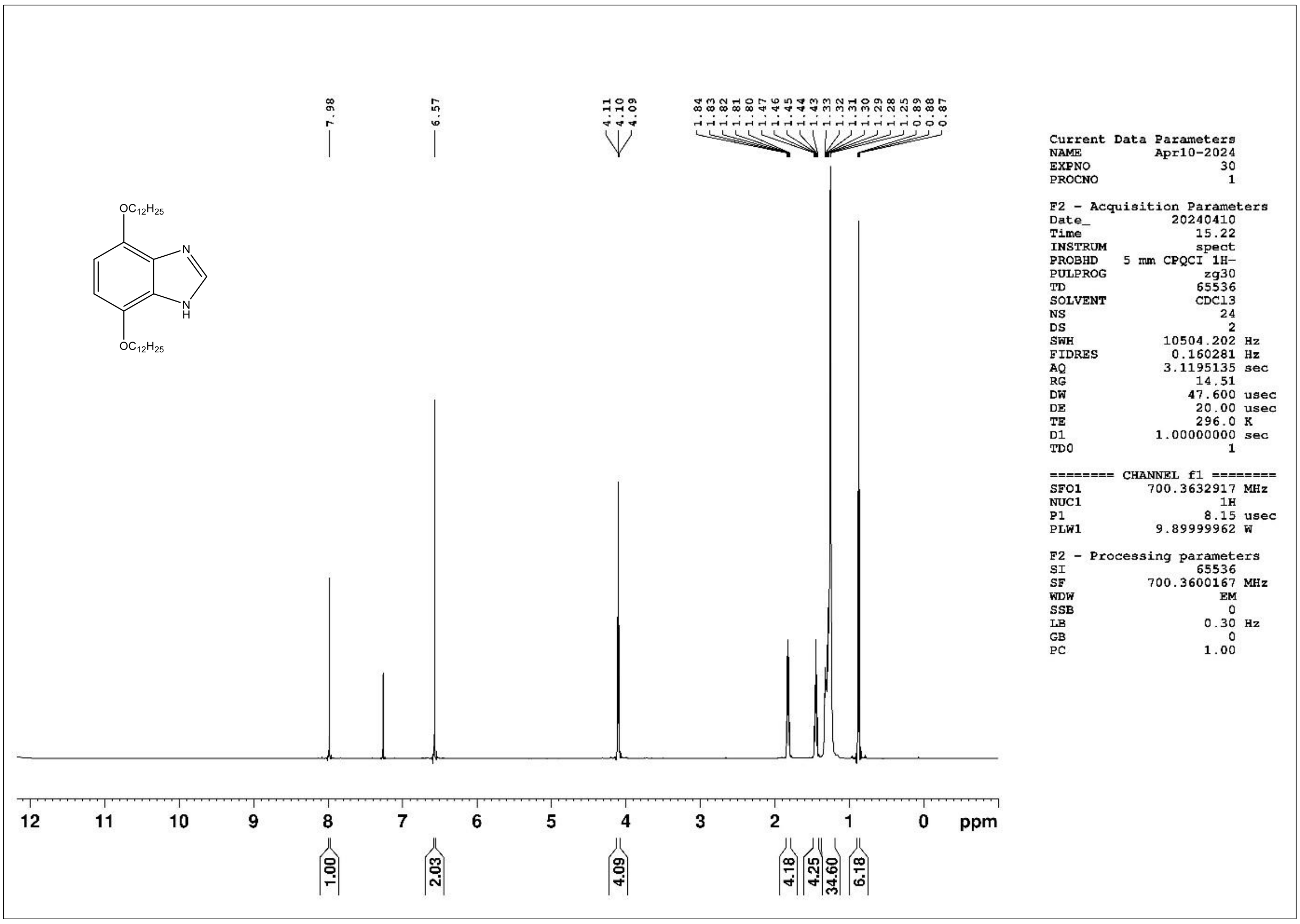

Figure S86. ^{1}H NMR of **L-Bi(12)**.

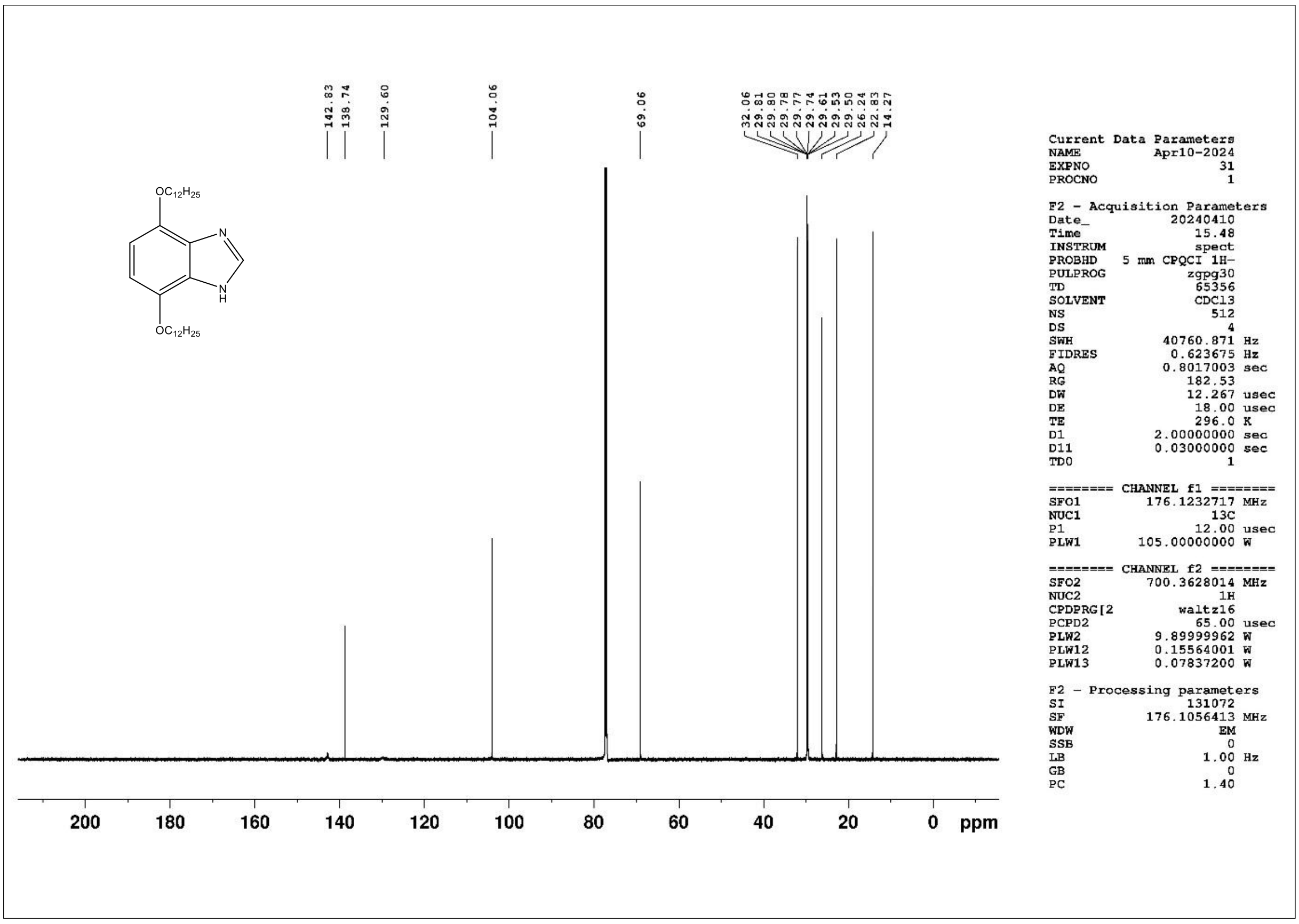

Figure S87. ^{13}C NMR of **L-Bi(12)**.

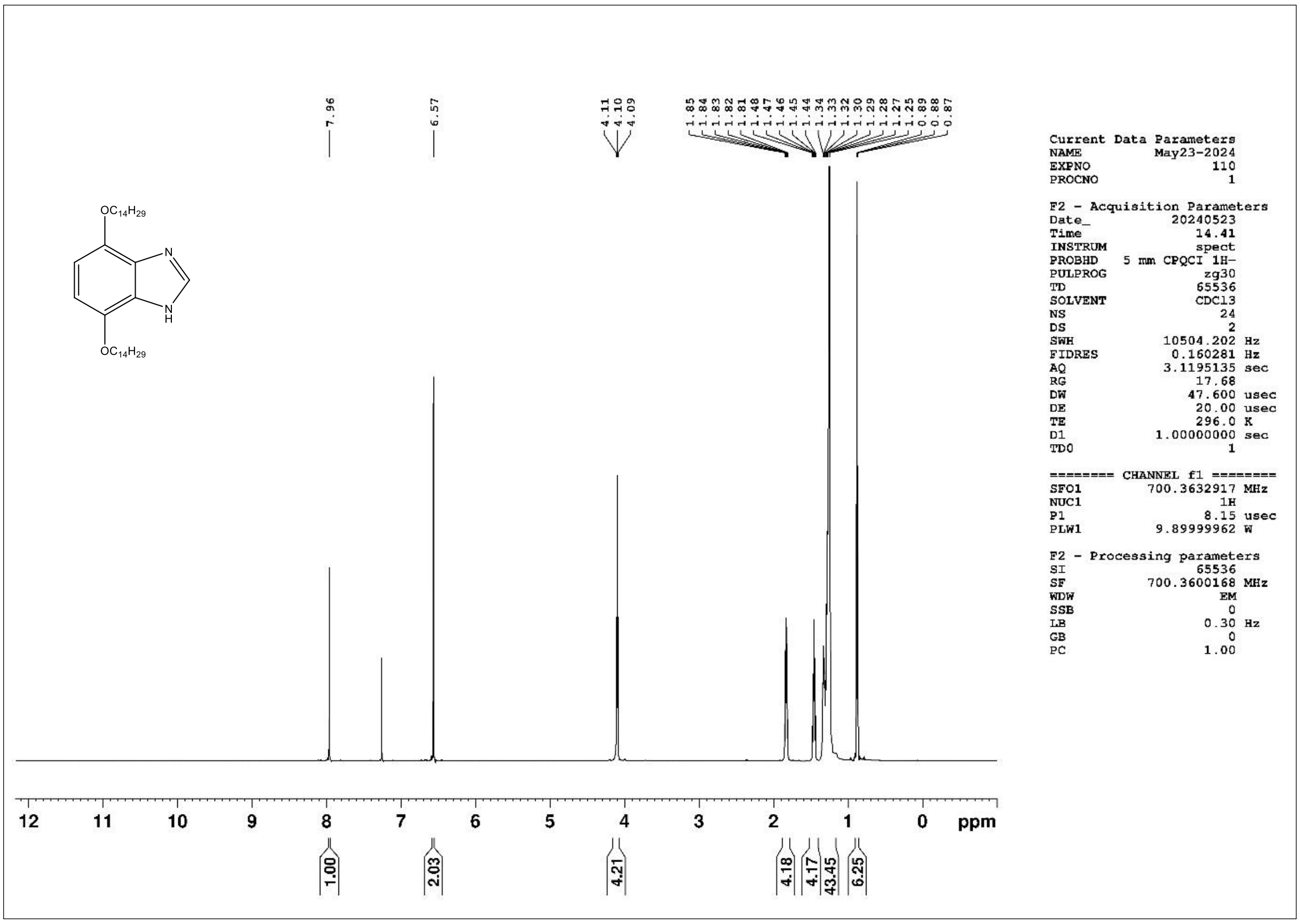

Figure S88. ^{1}H NMR of **L-Bi(14)**.

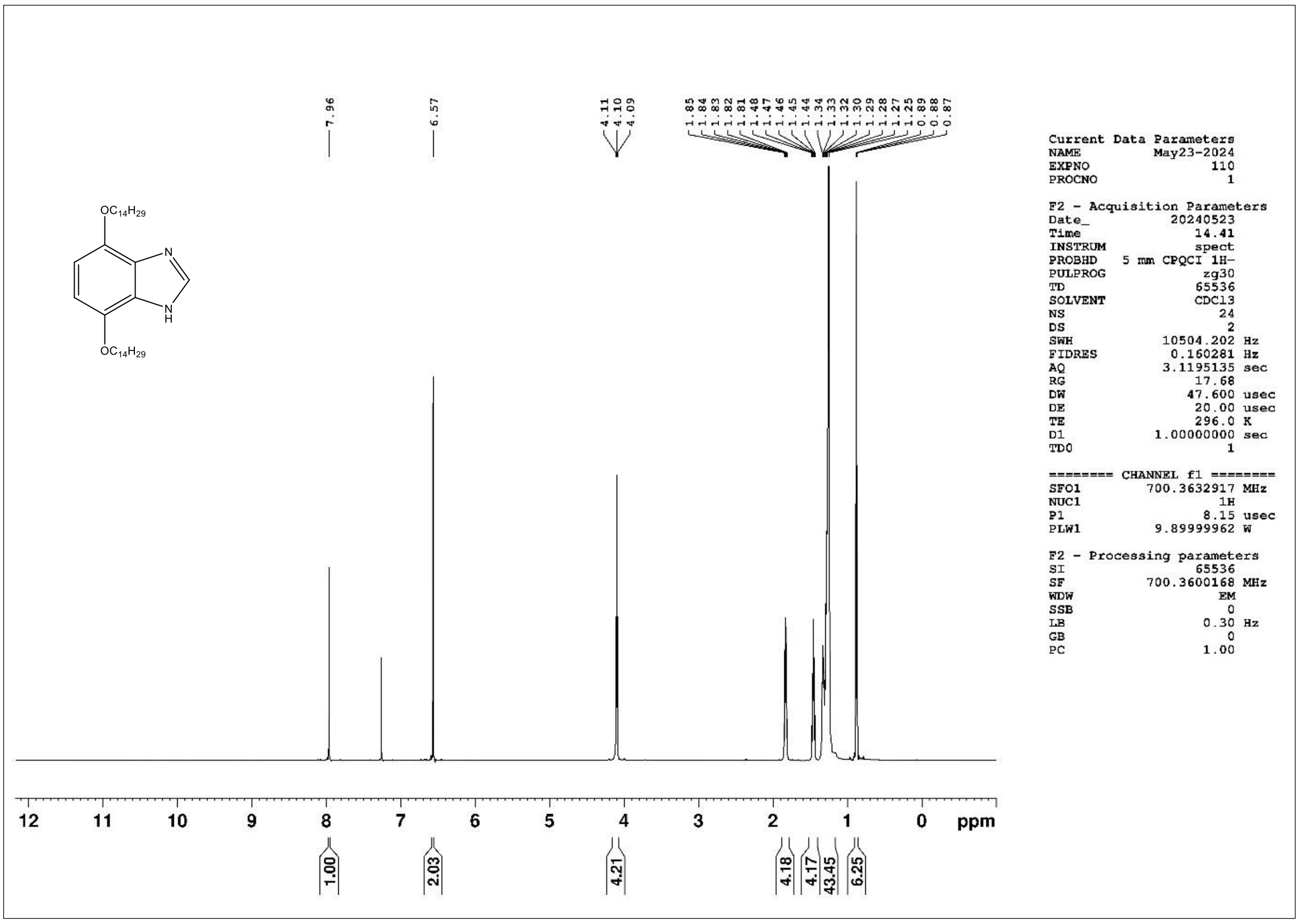

Figure S89. ^{13}C NMR of **L-Bi(14)**.

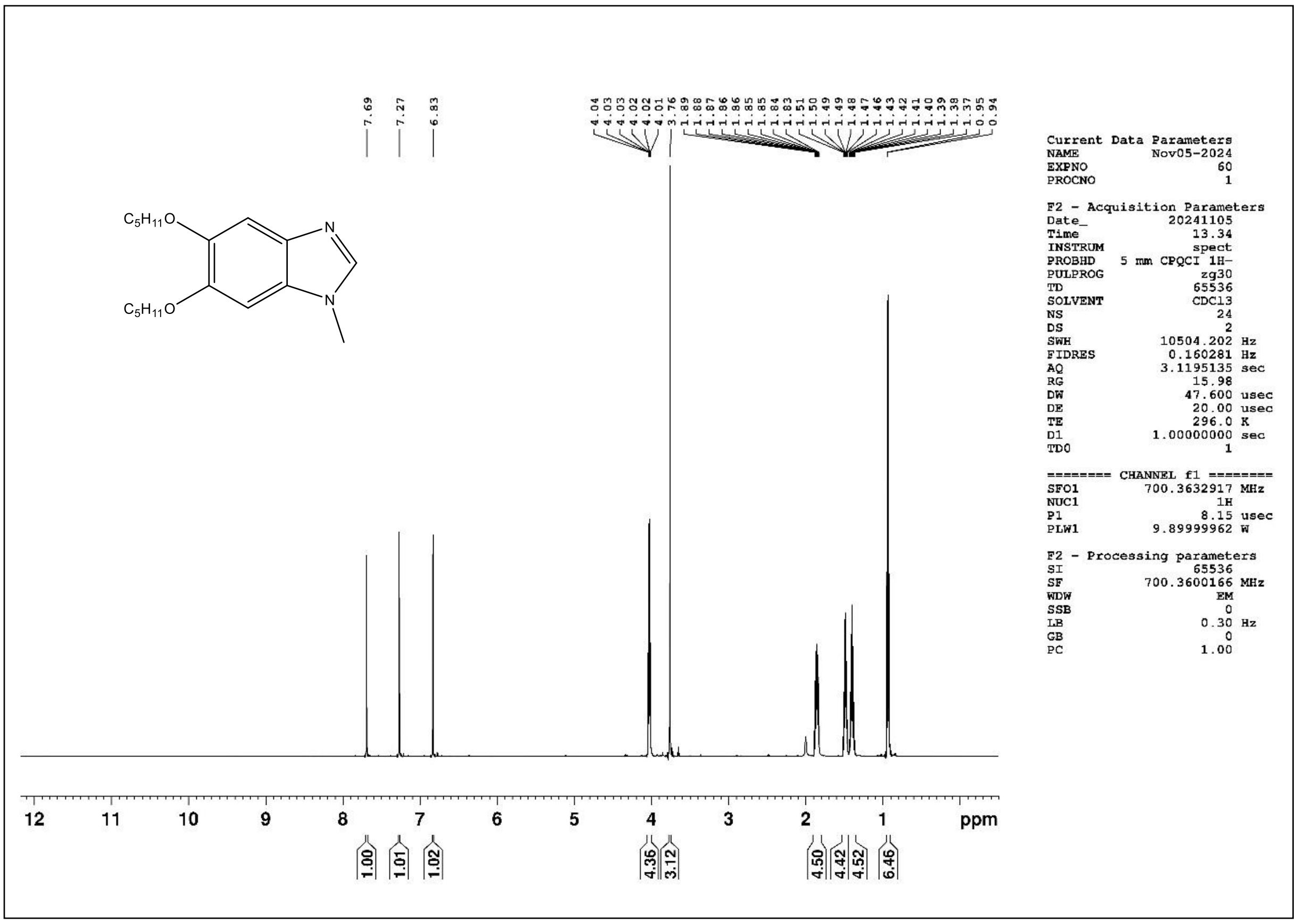

Figure S90. ^{1}H NMR of **U-MeBi(5)**.

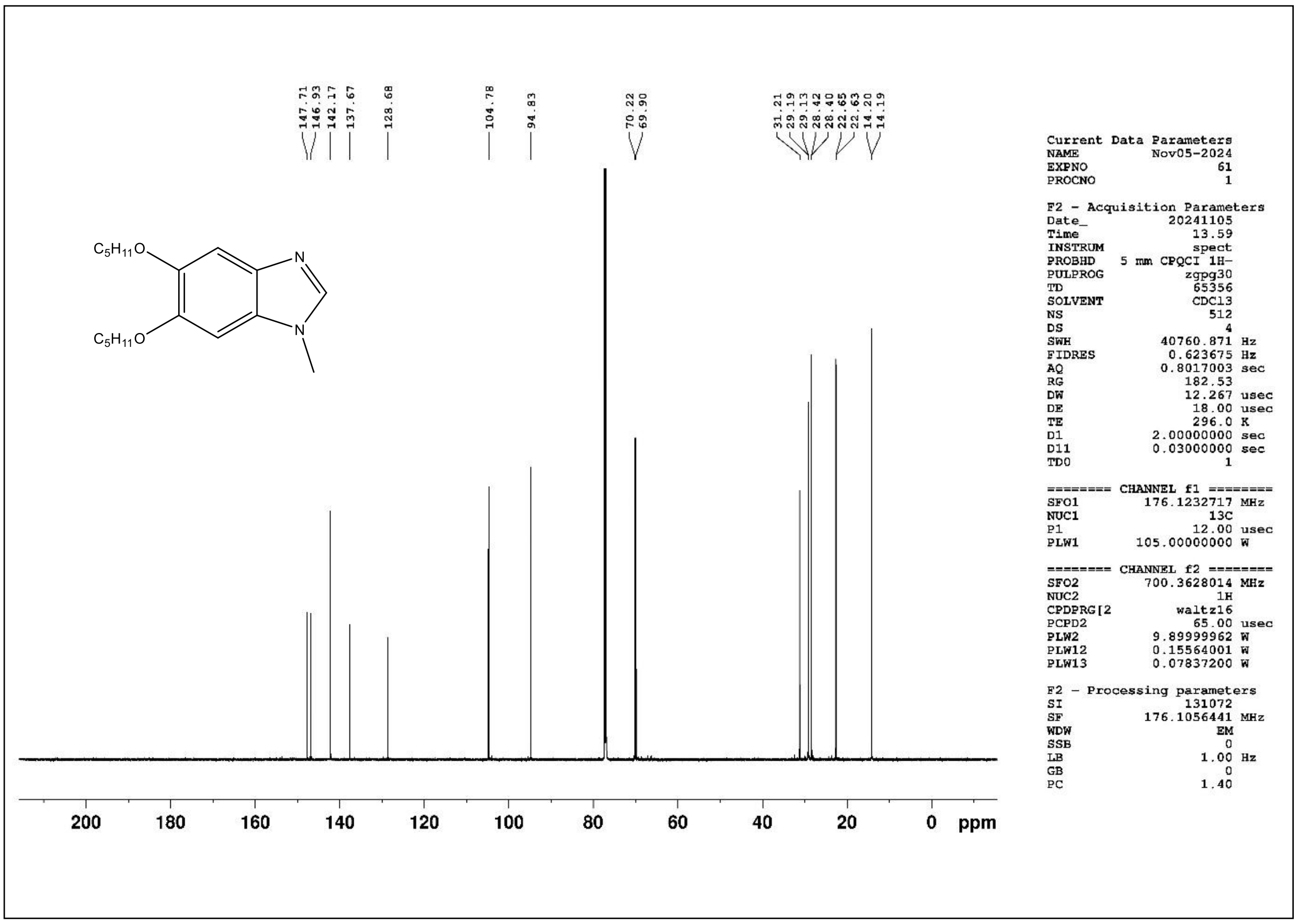

Figure S91. ^{13}C NMR of **U-MeBi(5)**.

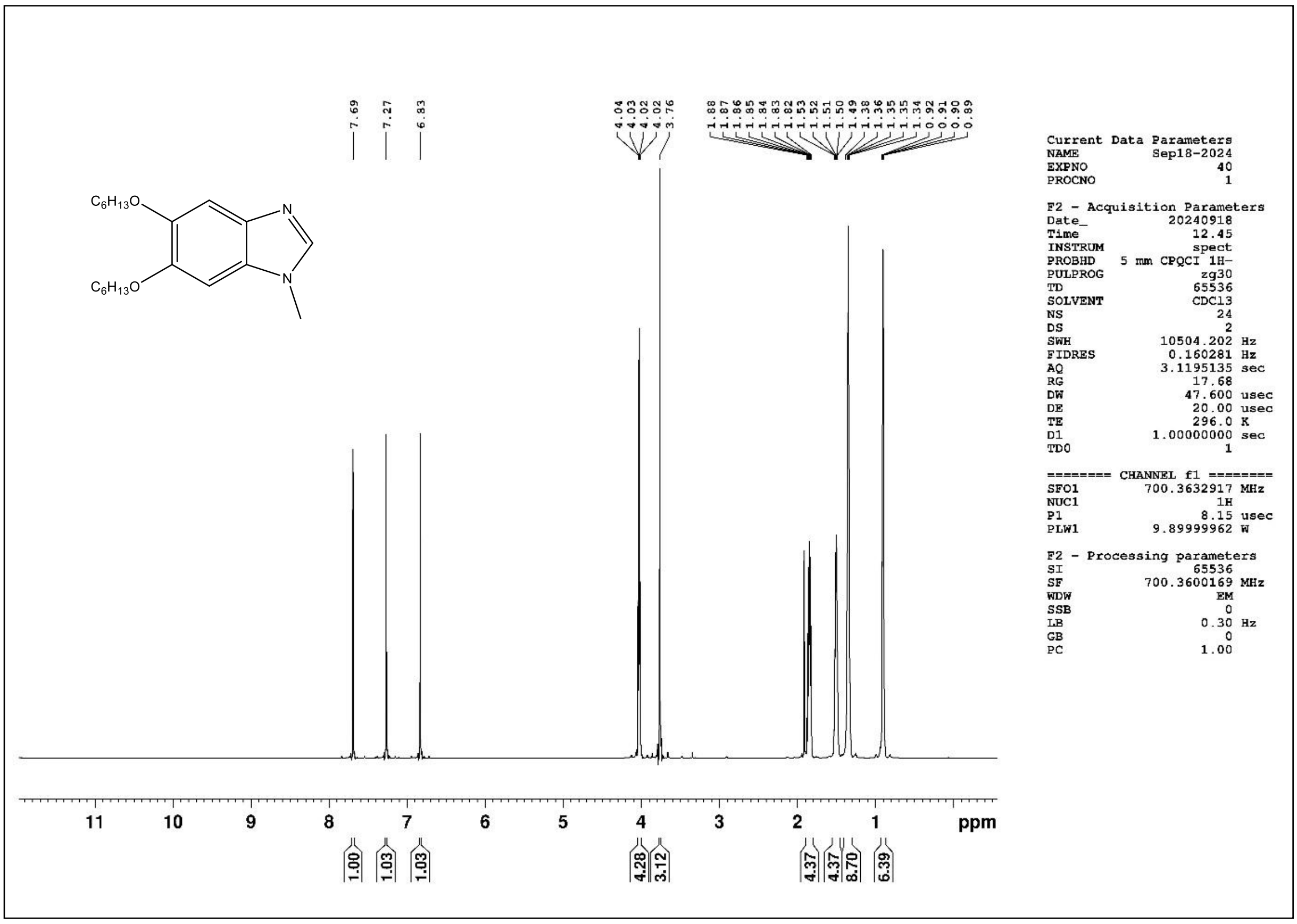

Figure S92. ^{1}H NMR of **U-MeBi(6)**.

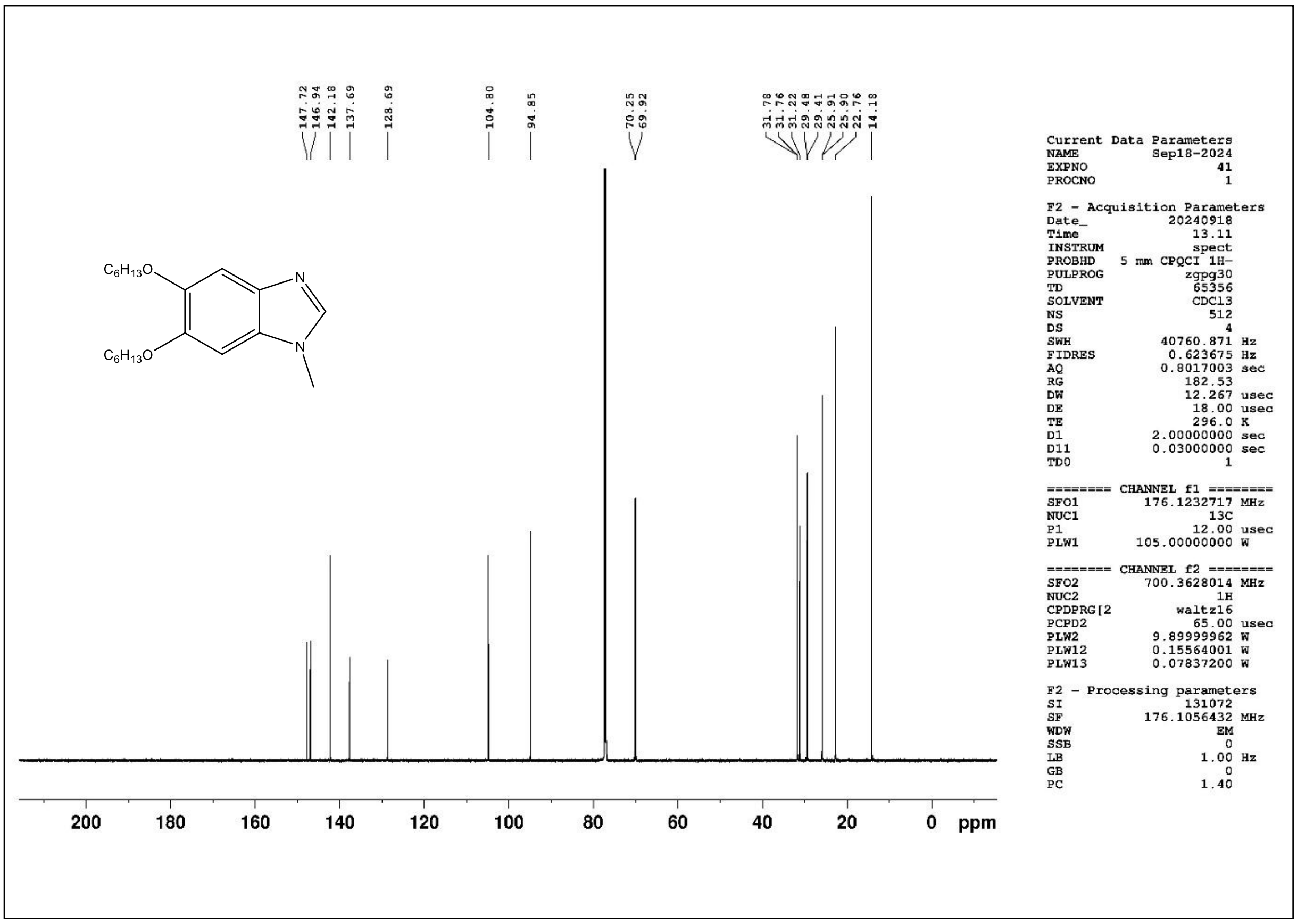

Figure S93. ^{13}C NMR of **U-MeBi(6)**.

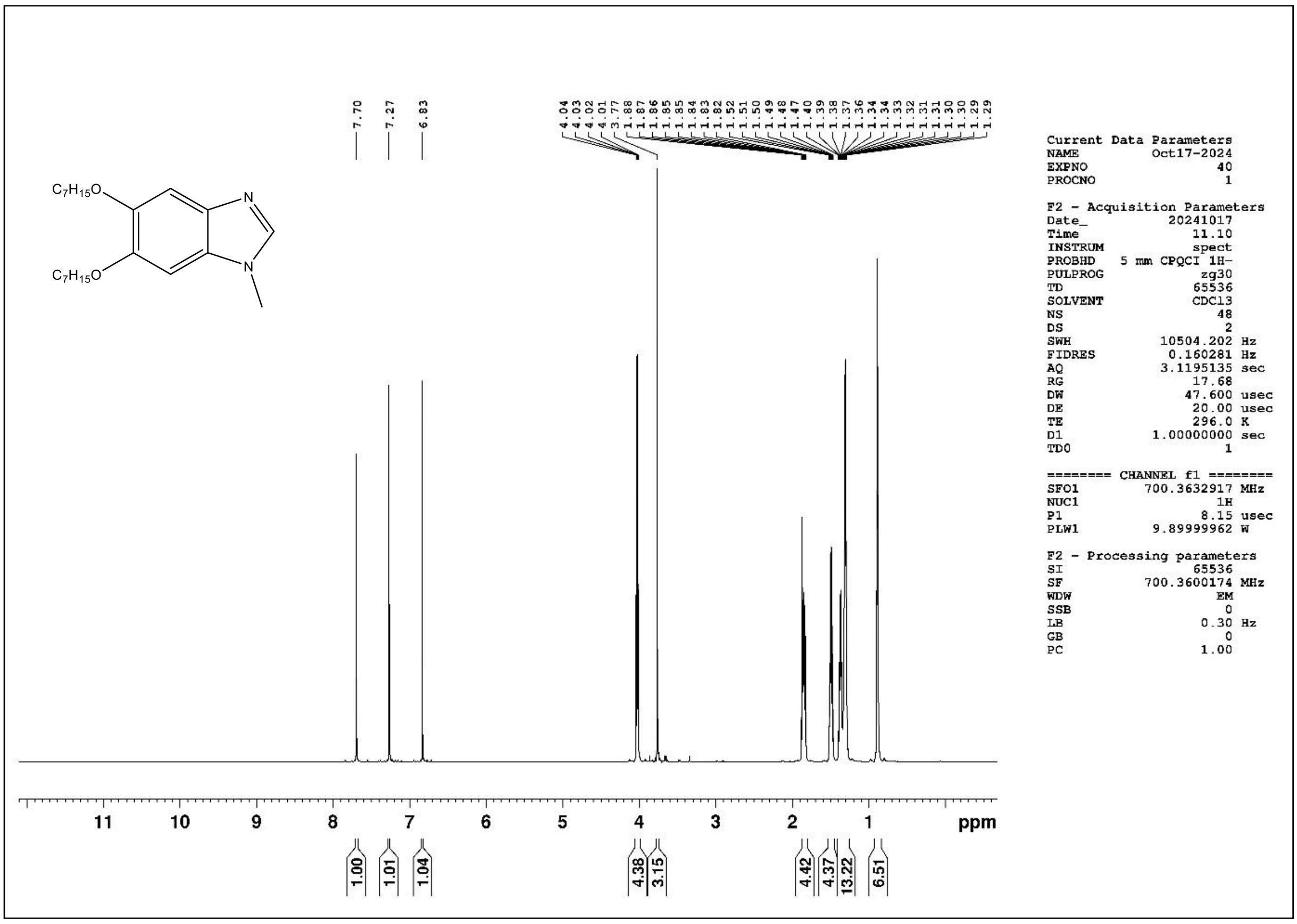

Figure S94. ^{1}H NMR of **U-MeBi(7)**.

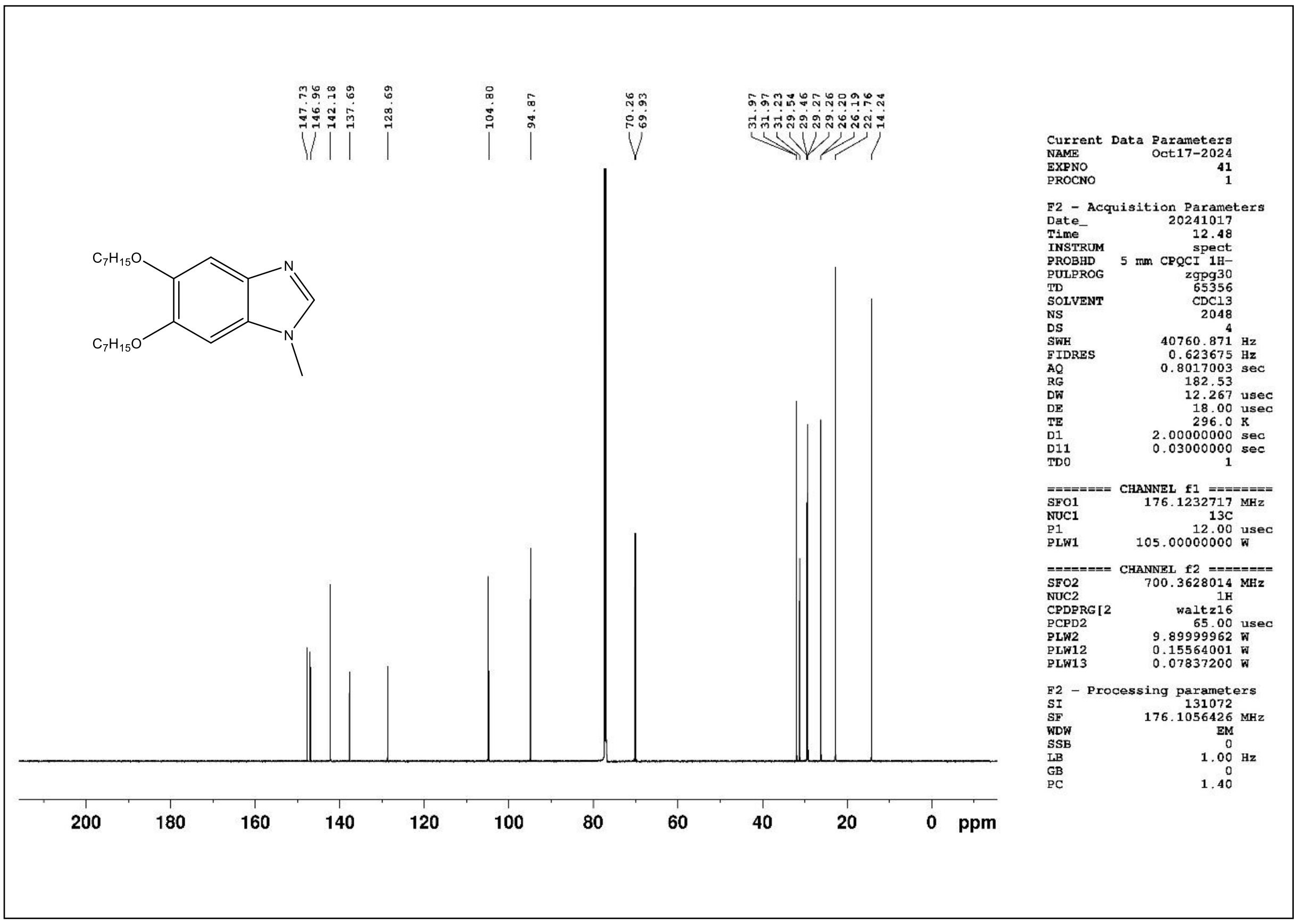

Figure S95. ^{13}C NMR of **U-MeBi(7)**.

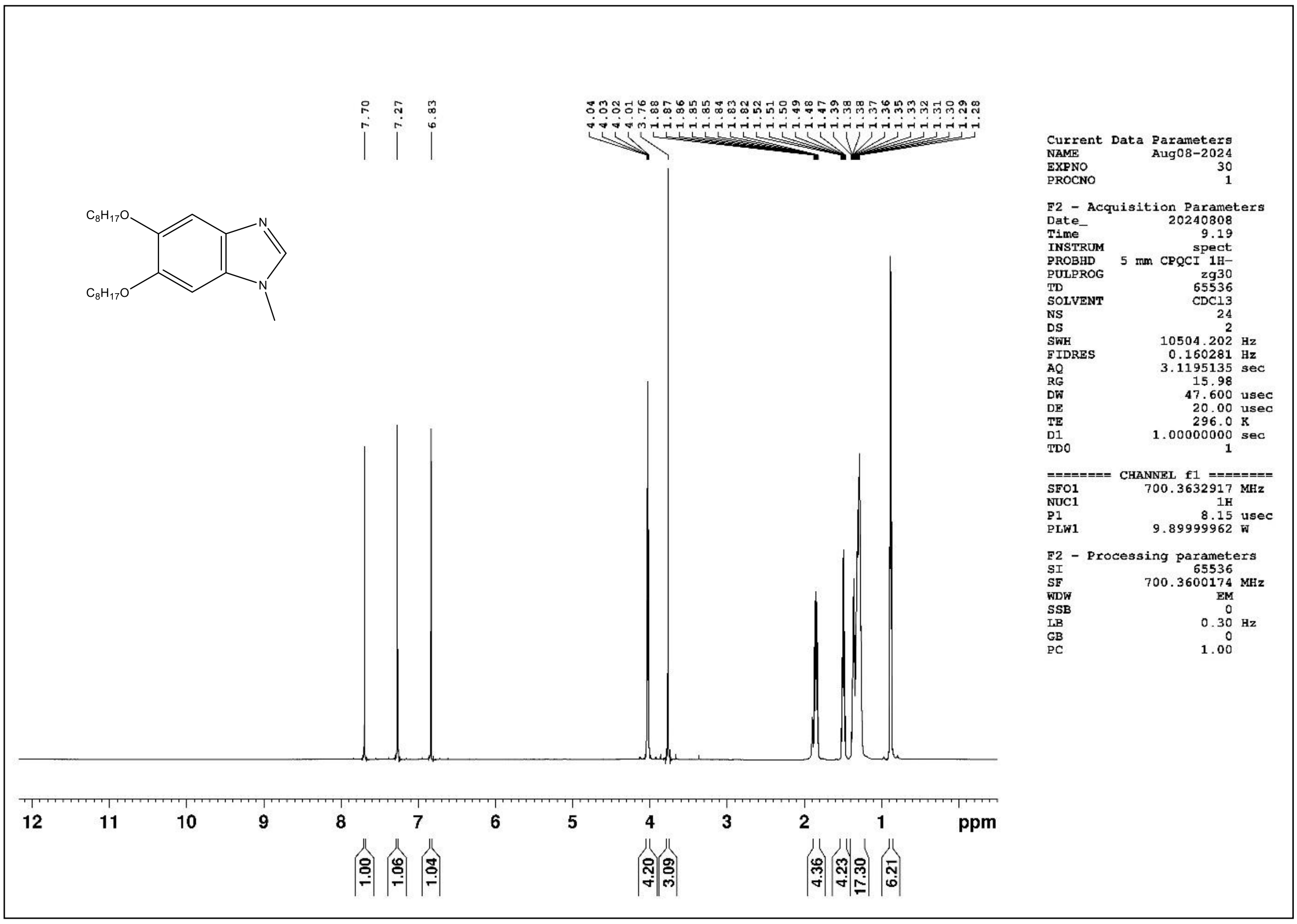

Figure S96. ^{1}H NMR of **U-MeBi(8)**.

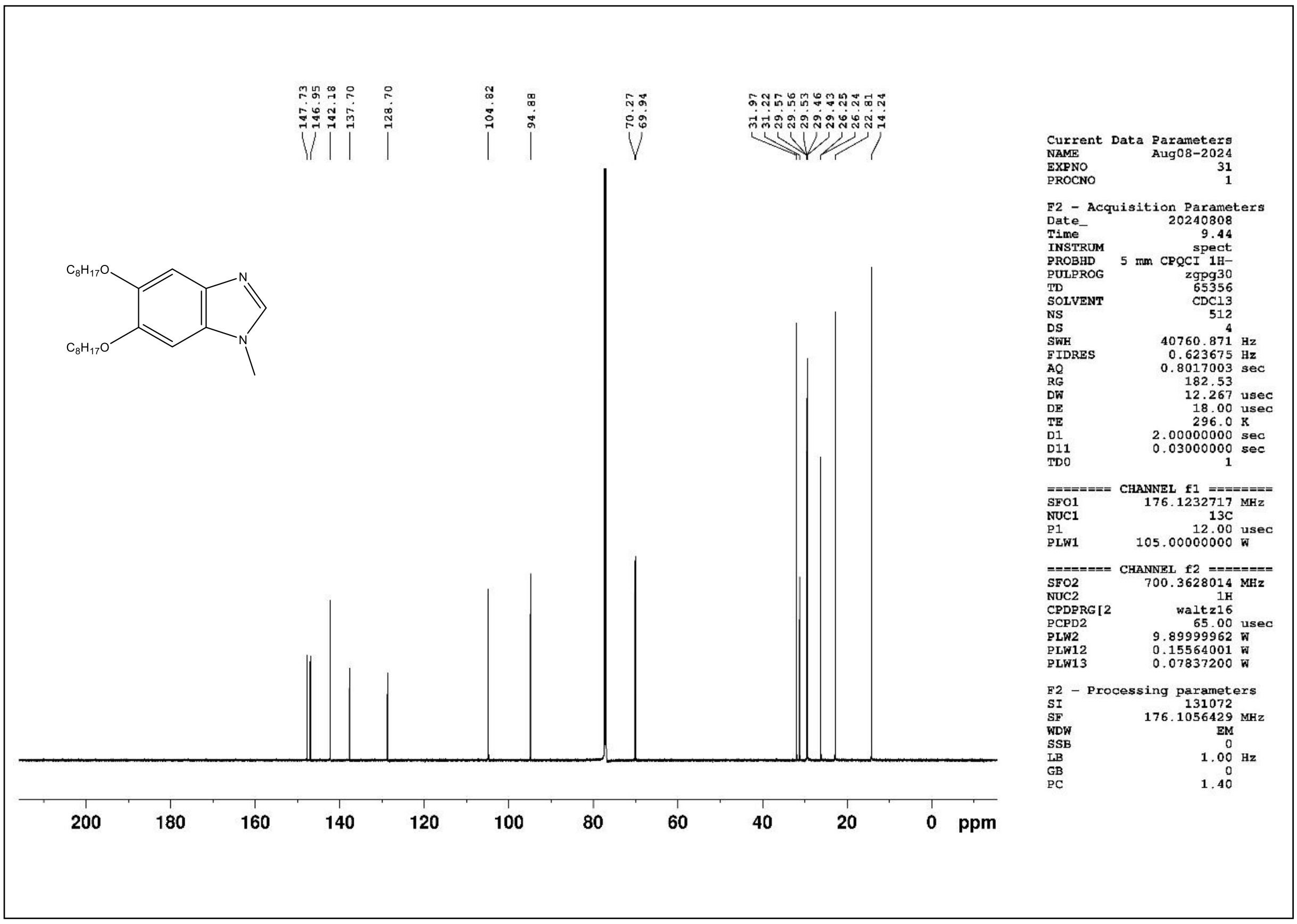

Figure S97. ^{13}C NMR of **U-MeBi(8)**.

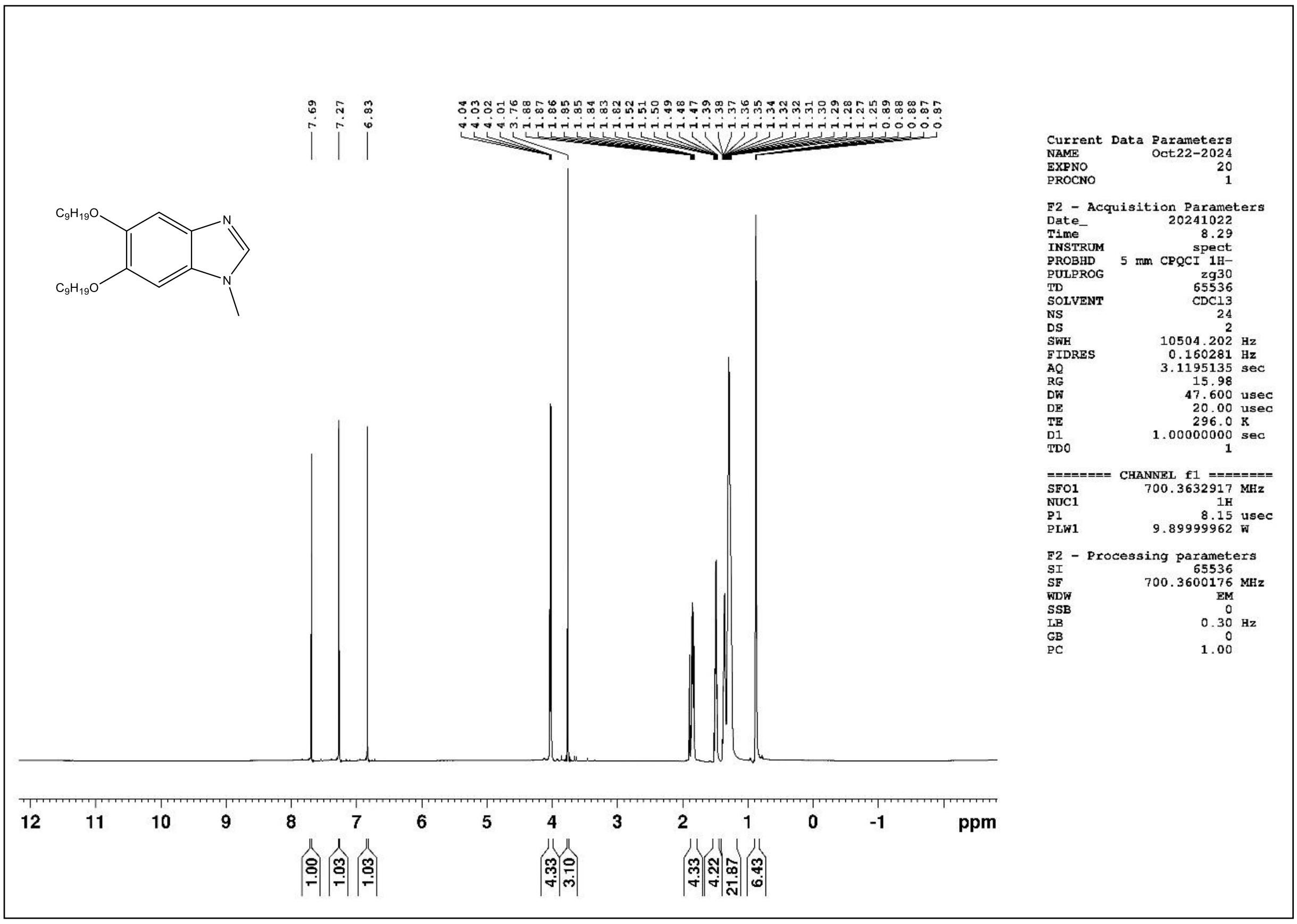

Figure S98. ^{1}H NMR of **U-MeBi(9)**.

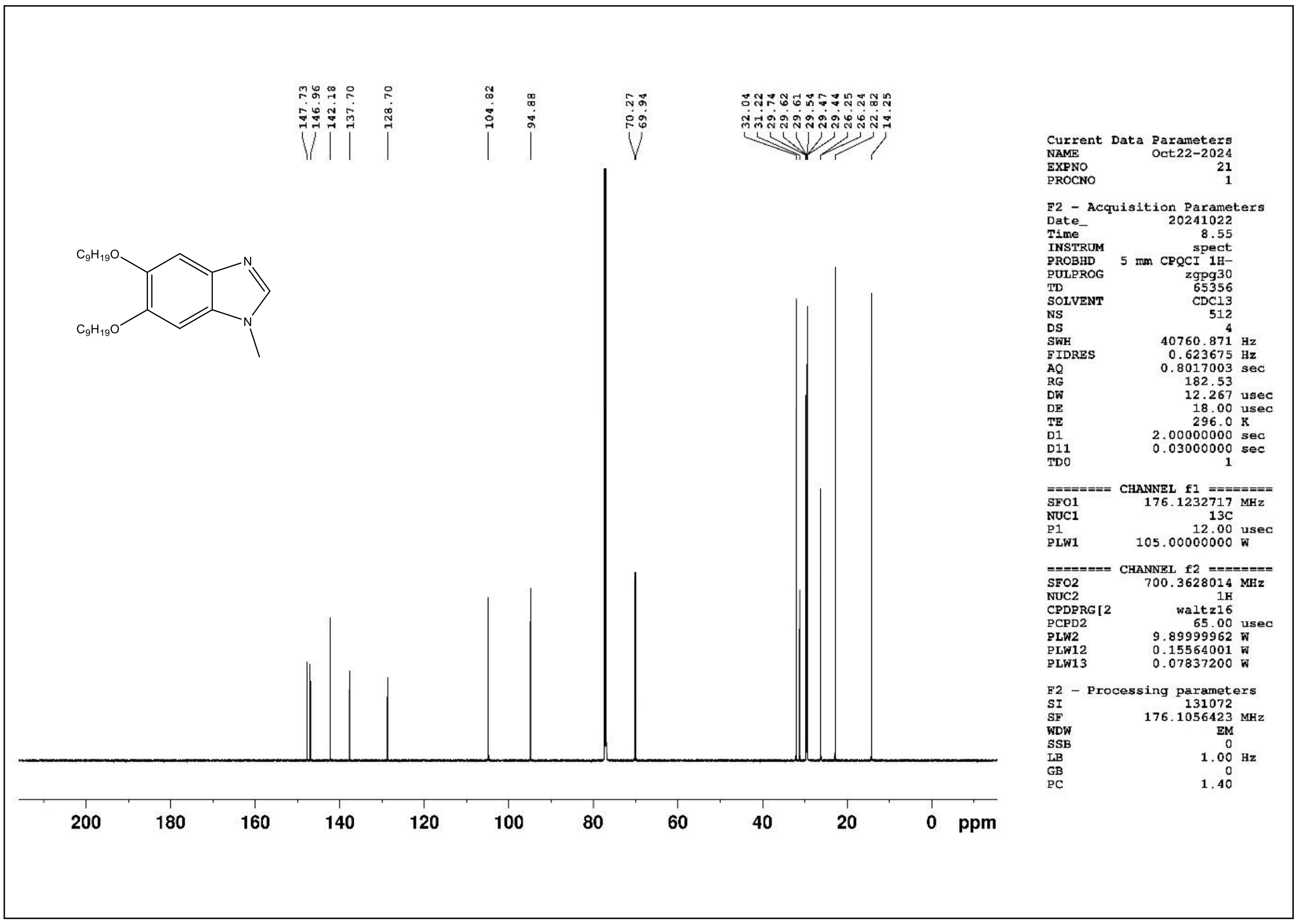

Figure S99. ^{13}C NMR of **U-MeBi(9)**.

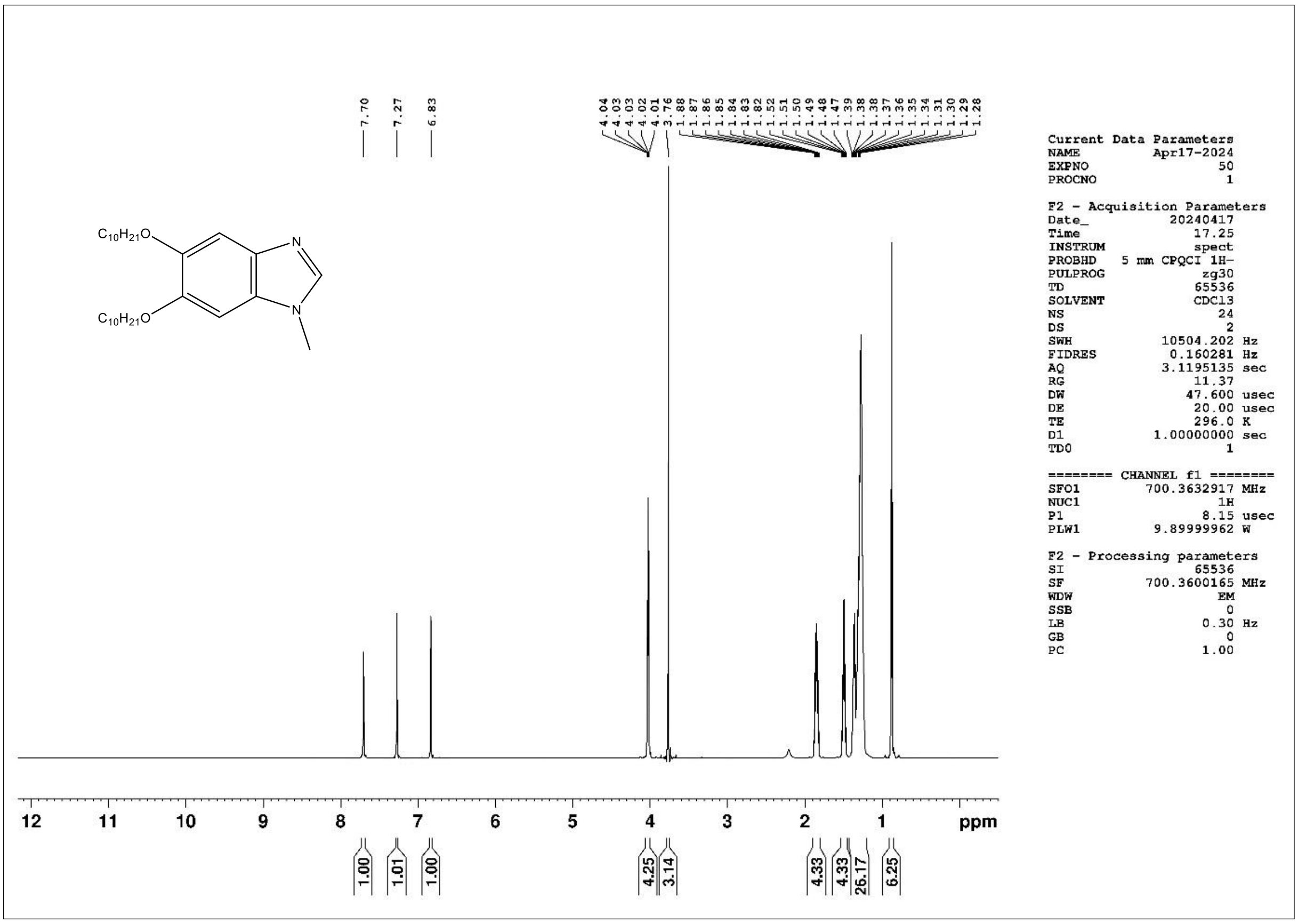

Figure S100. ^{1}H NMR of **U-MeBi(10)**.

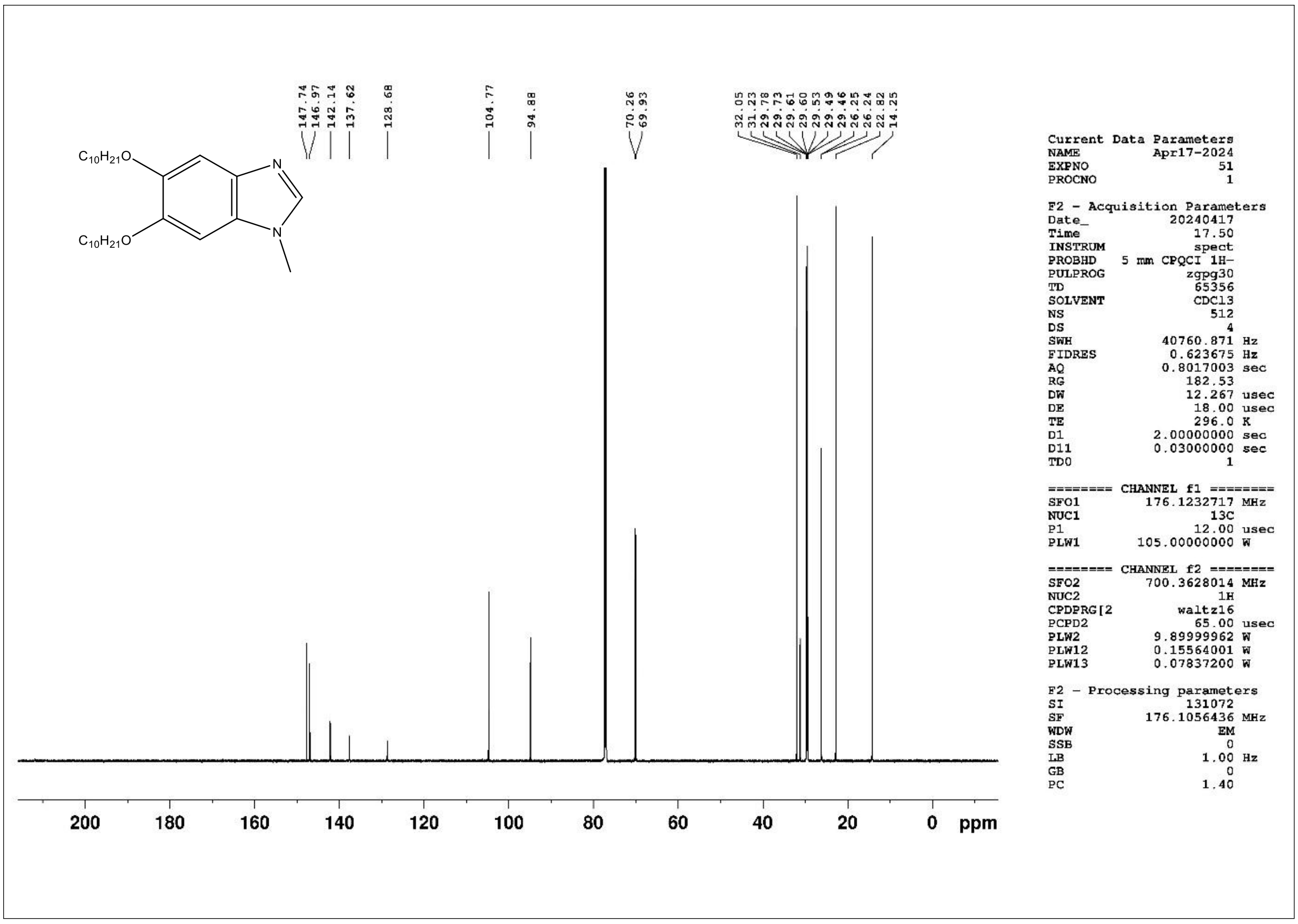

Figure S101. ^{13}C NMR of **U-MeBi(10)**.

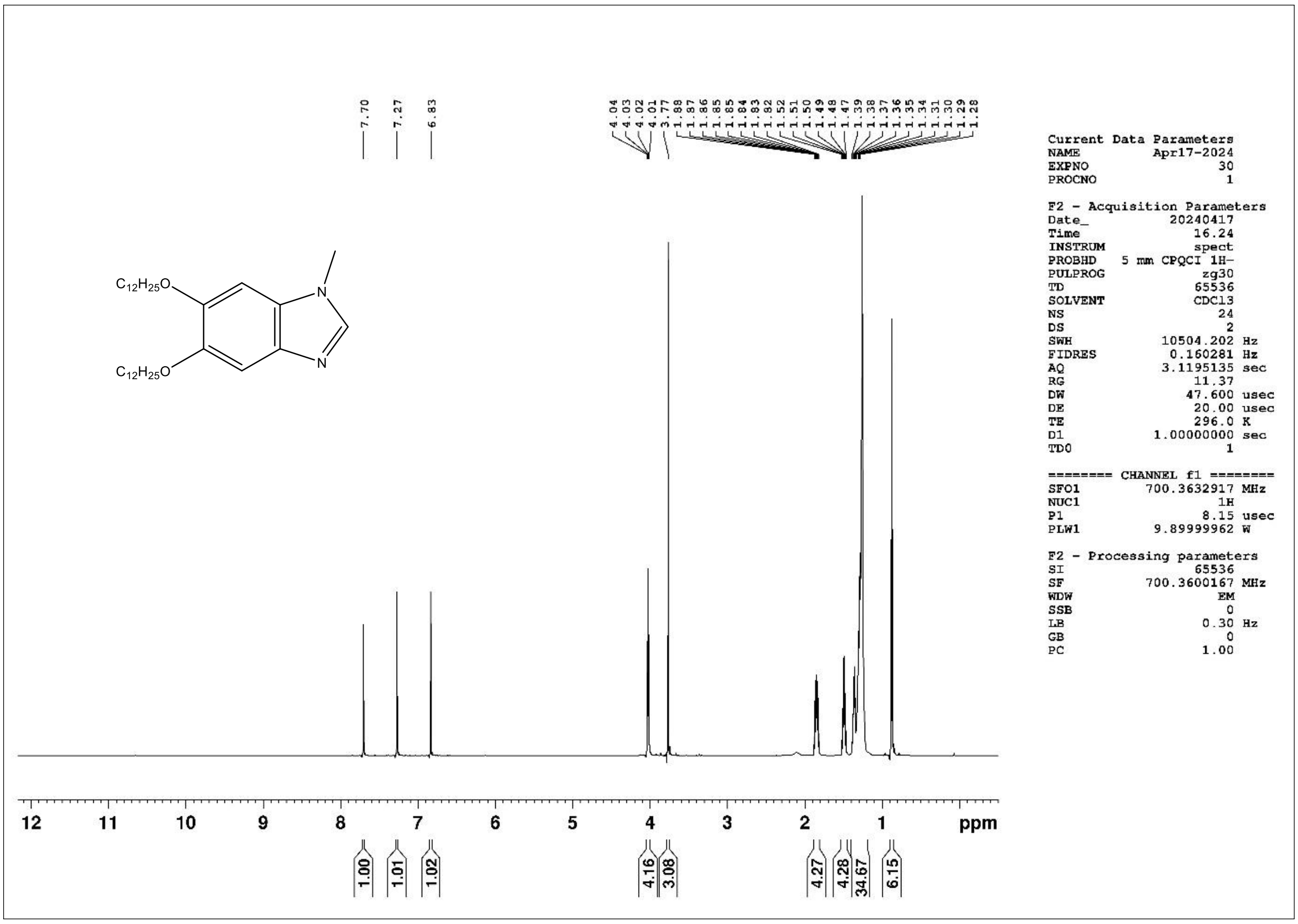

Figure S102. ^{1}H NMR of **U-MeBi(12)**.

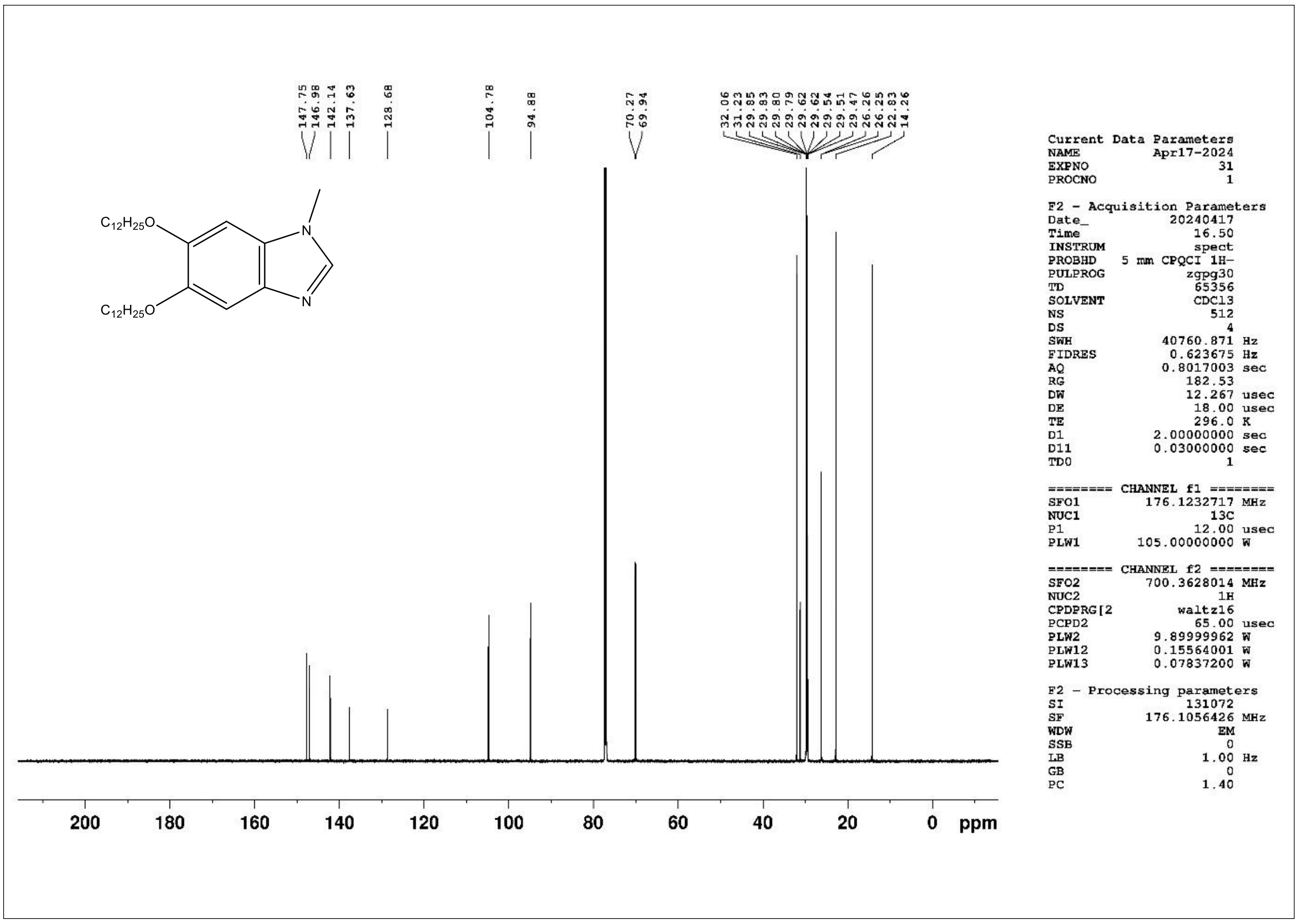

Figure S103. ^{13}C NMR of **U-MeBi(12)**.

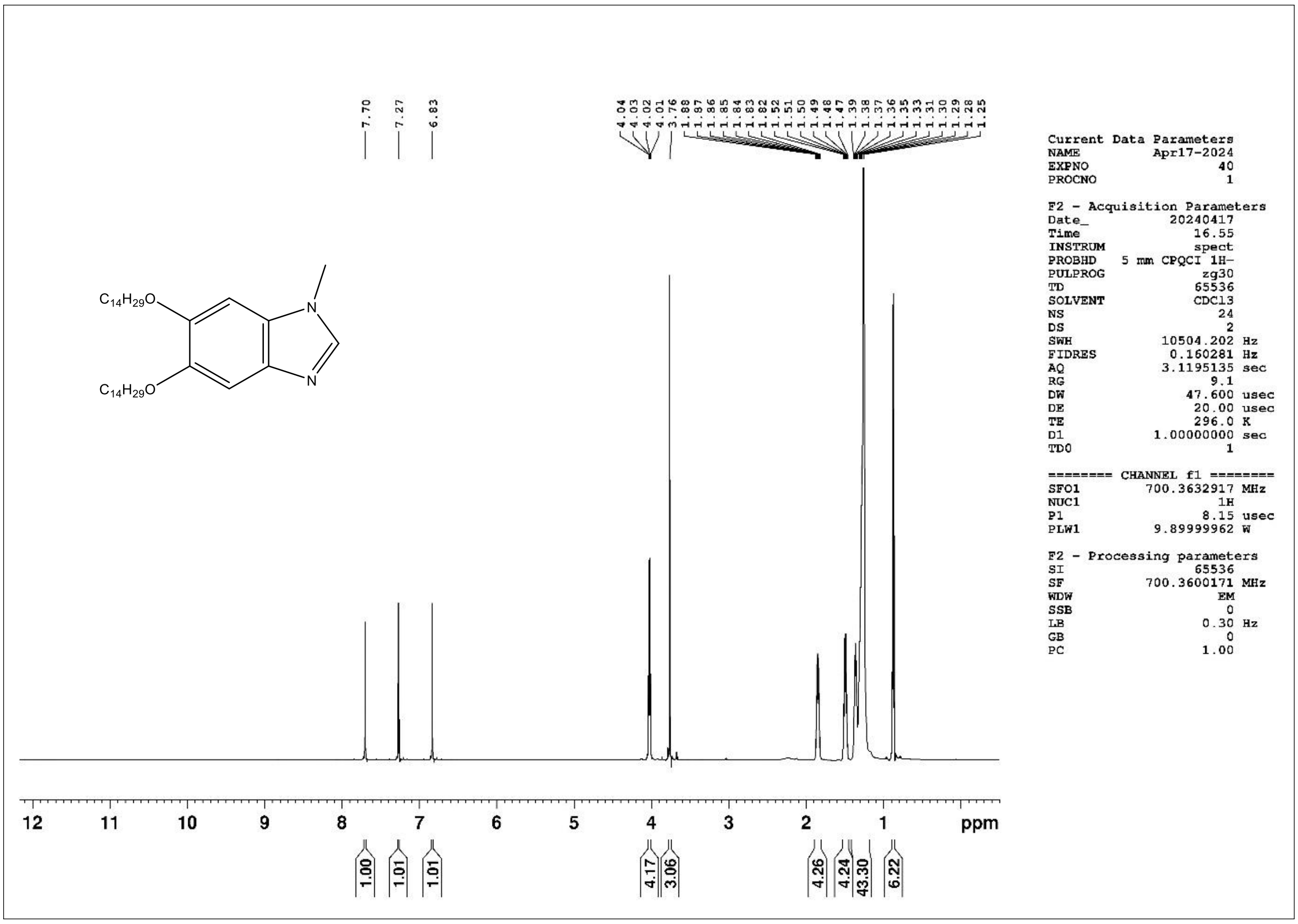

Figure S104. ^{1}H NMR of **U-MeBi(14)**.

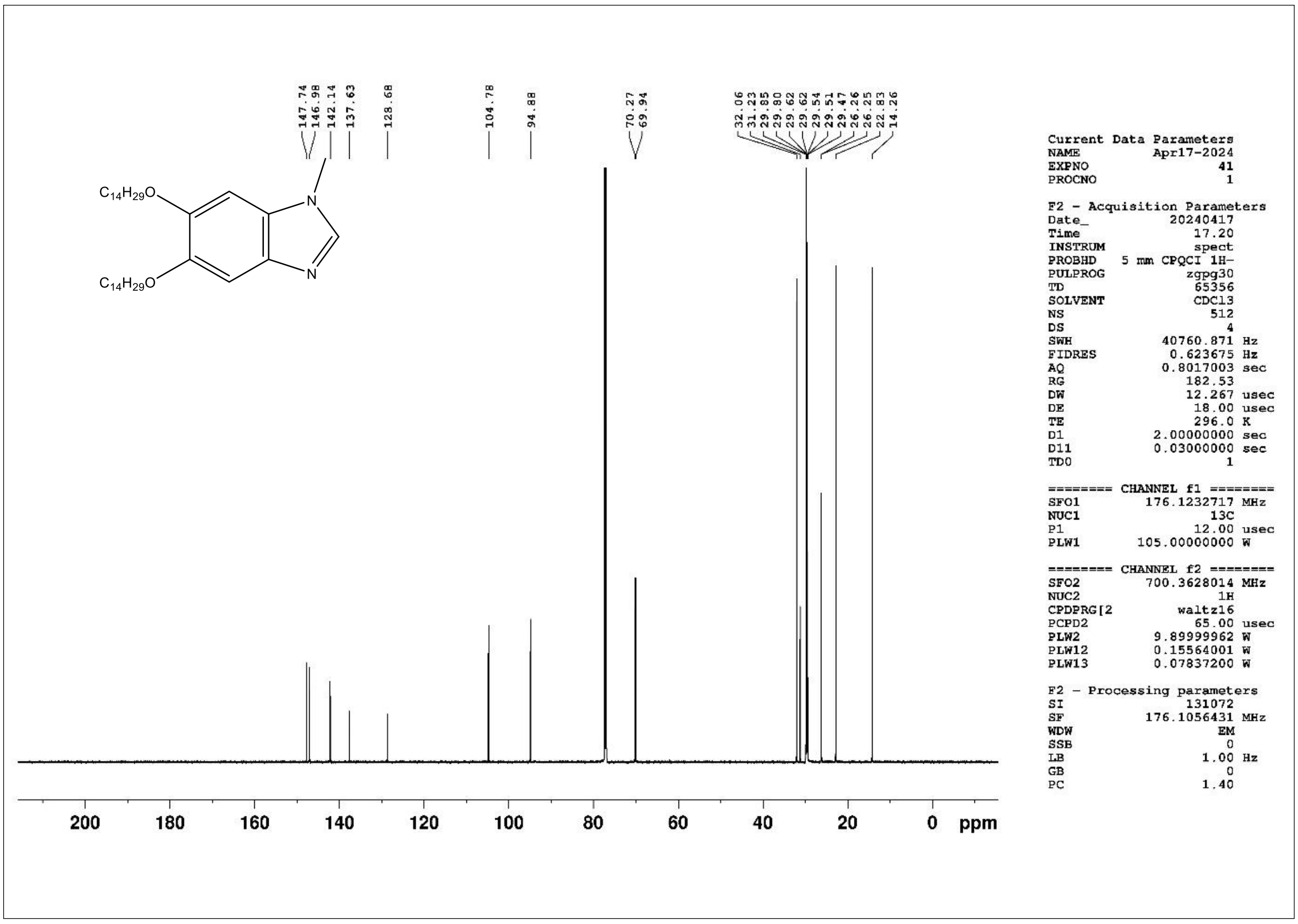

Figure S105. ^{13}C NMR of **U-MeBi(14)**.

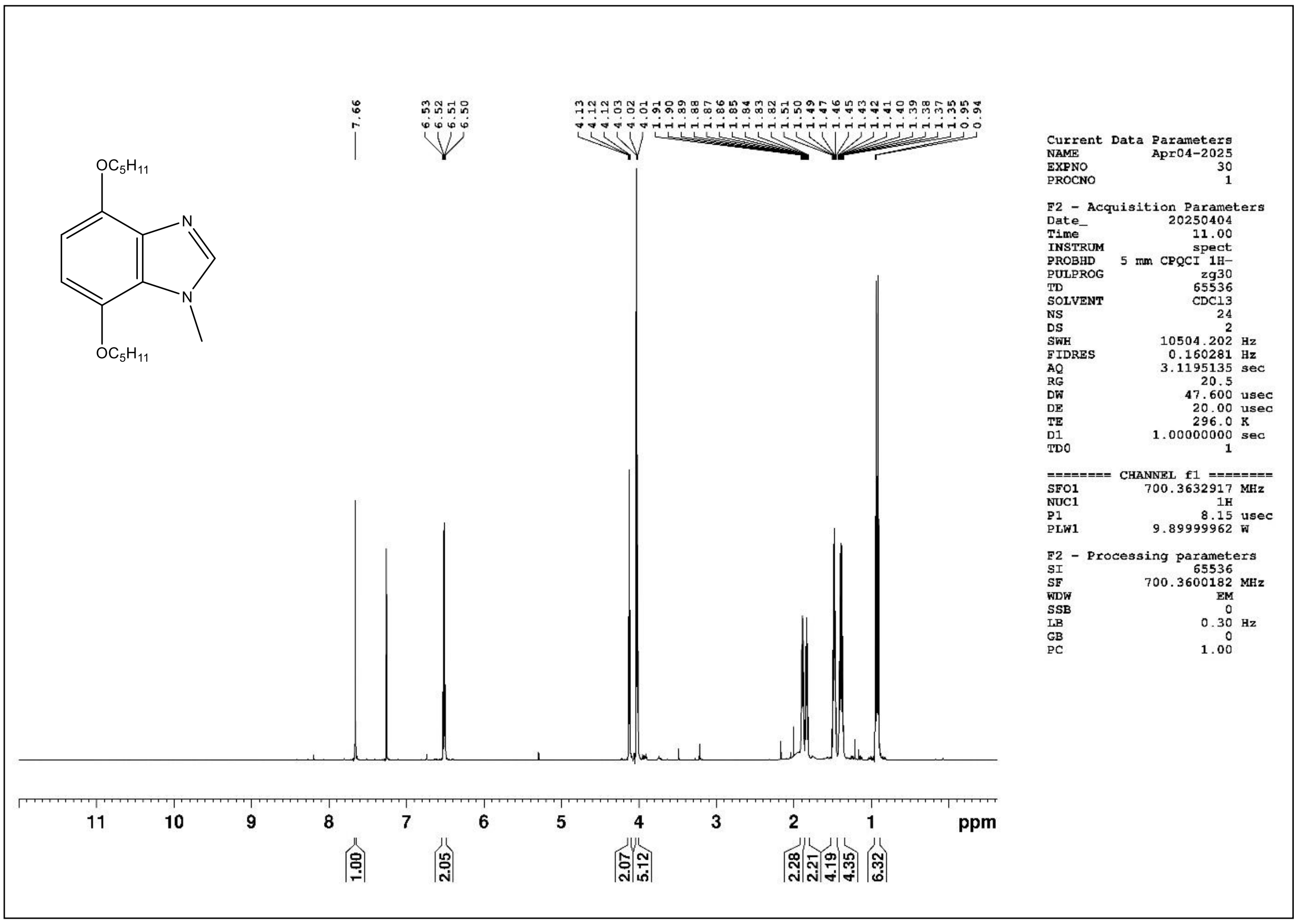

Figure S106. ^{1}H NMR of **L-MeBi(5)**.

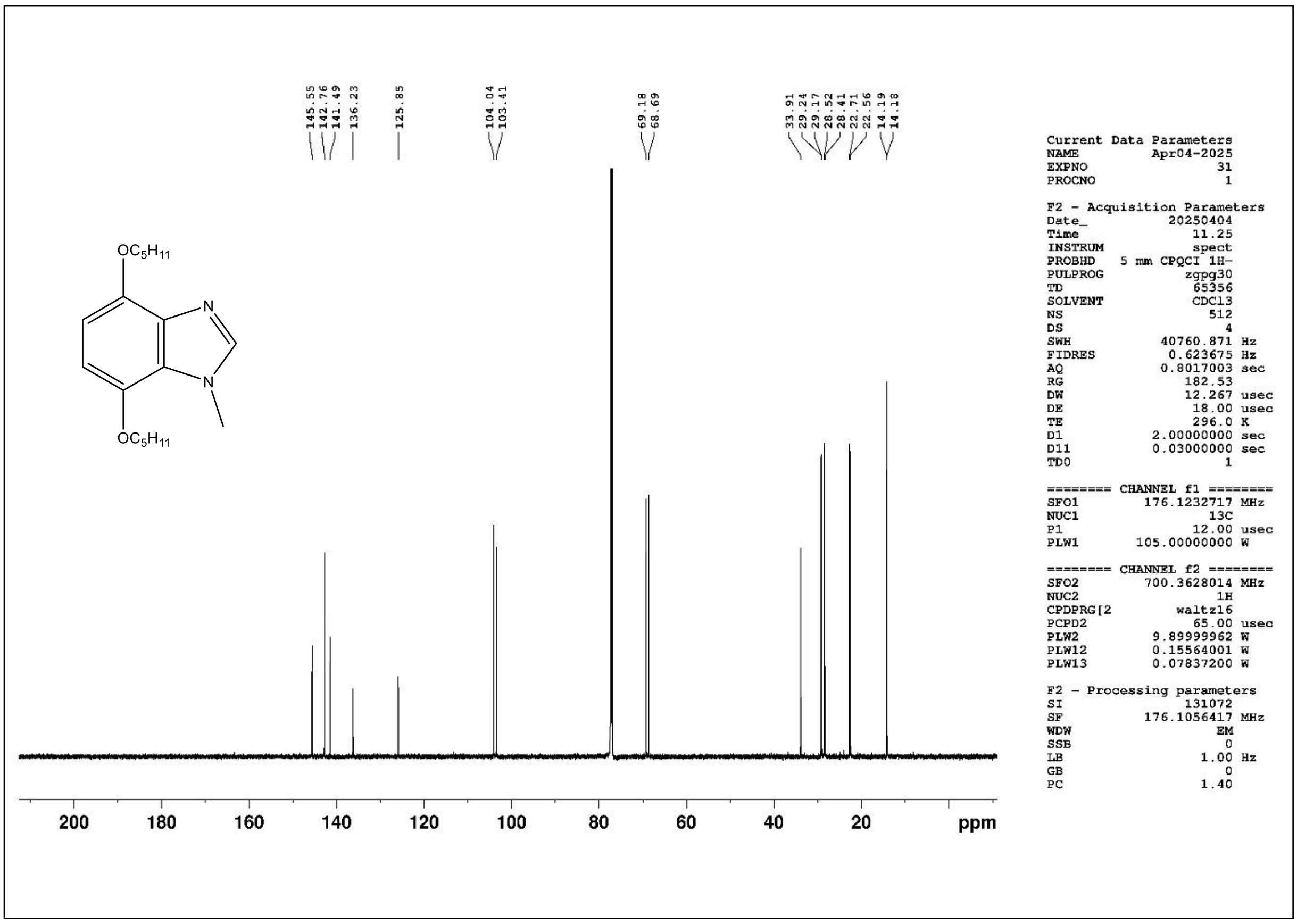

Figure S107. ^{13}C NMR of **L-MeBi(5)**.

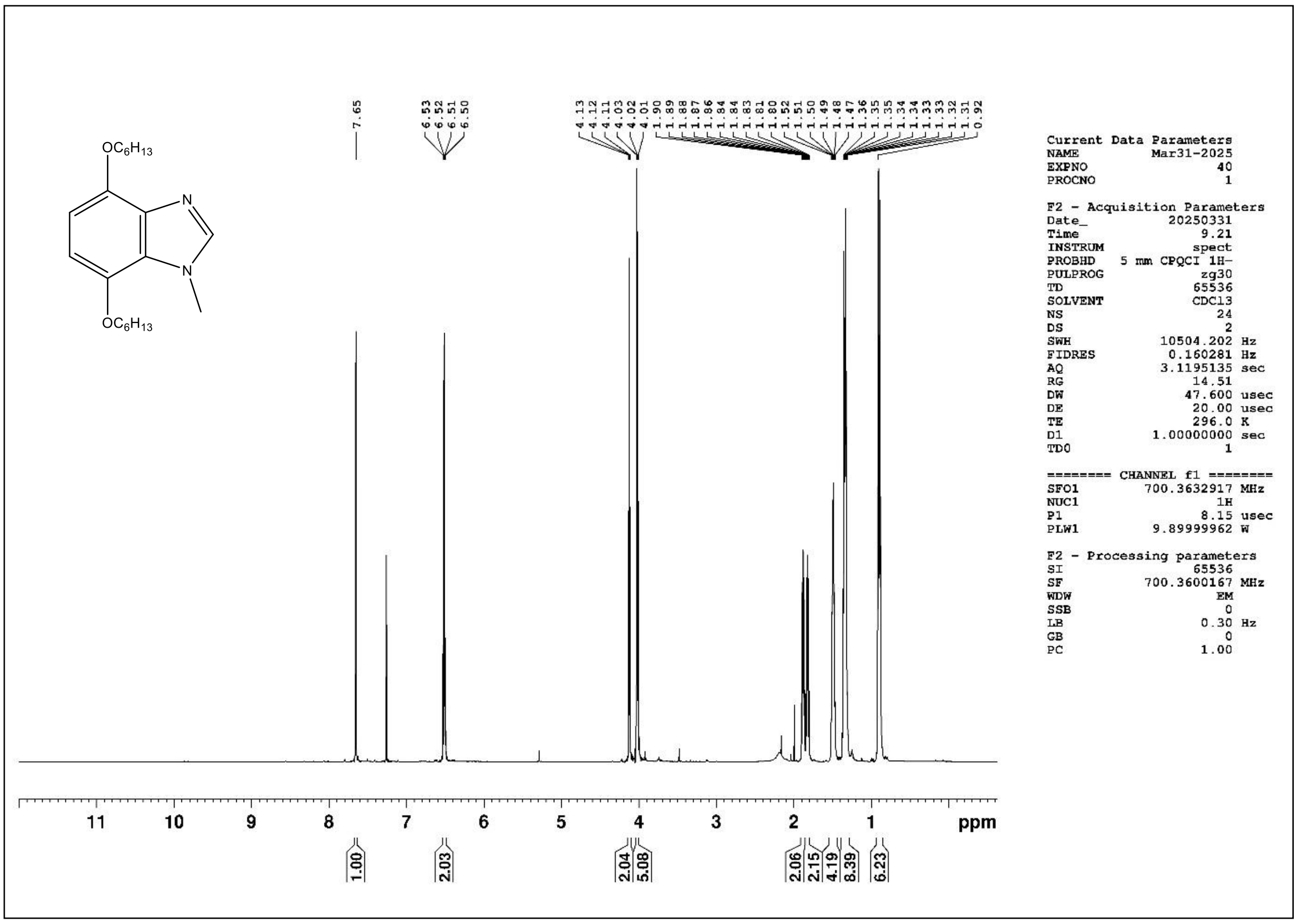

Figure S108. ^{1}H NMR of **L-MeBi(6)**.

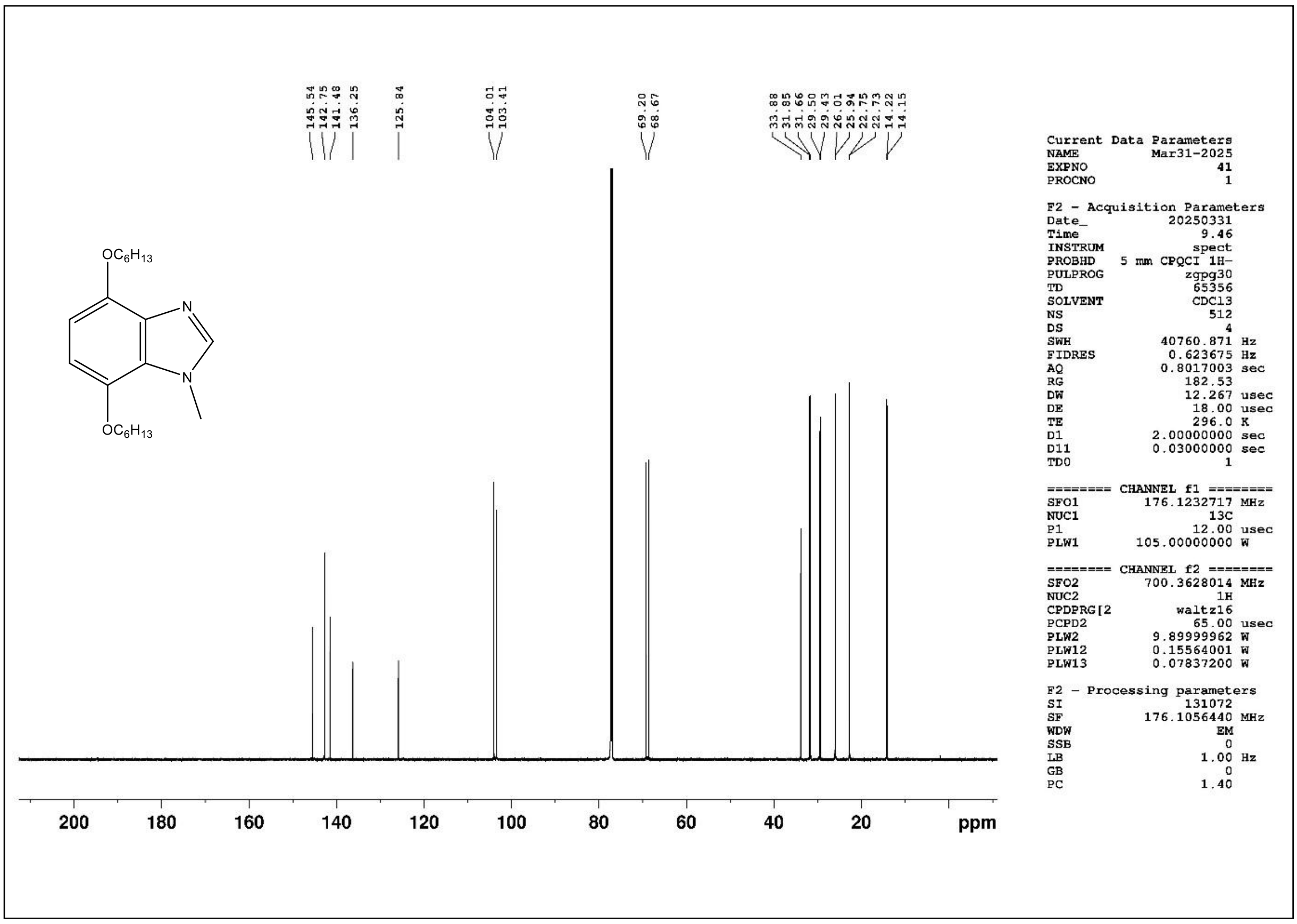

Figure S109. ^{13}C NMR of **L-MeBi(6)**.

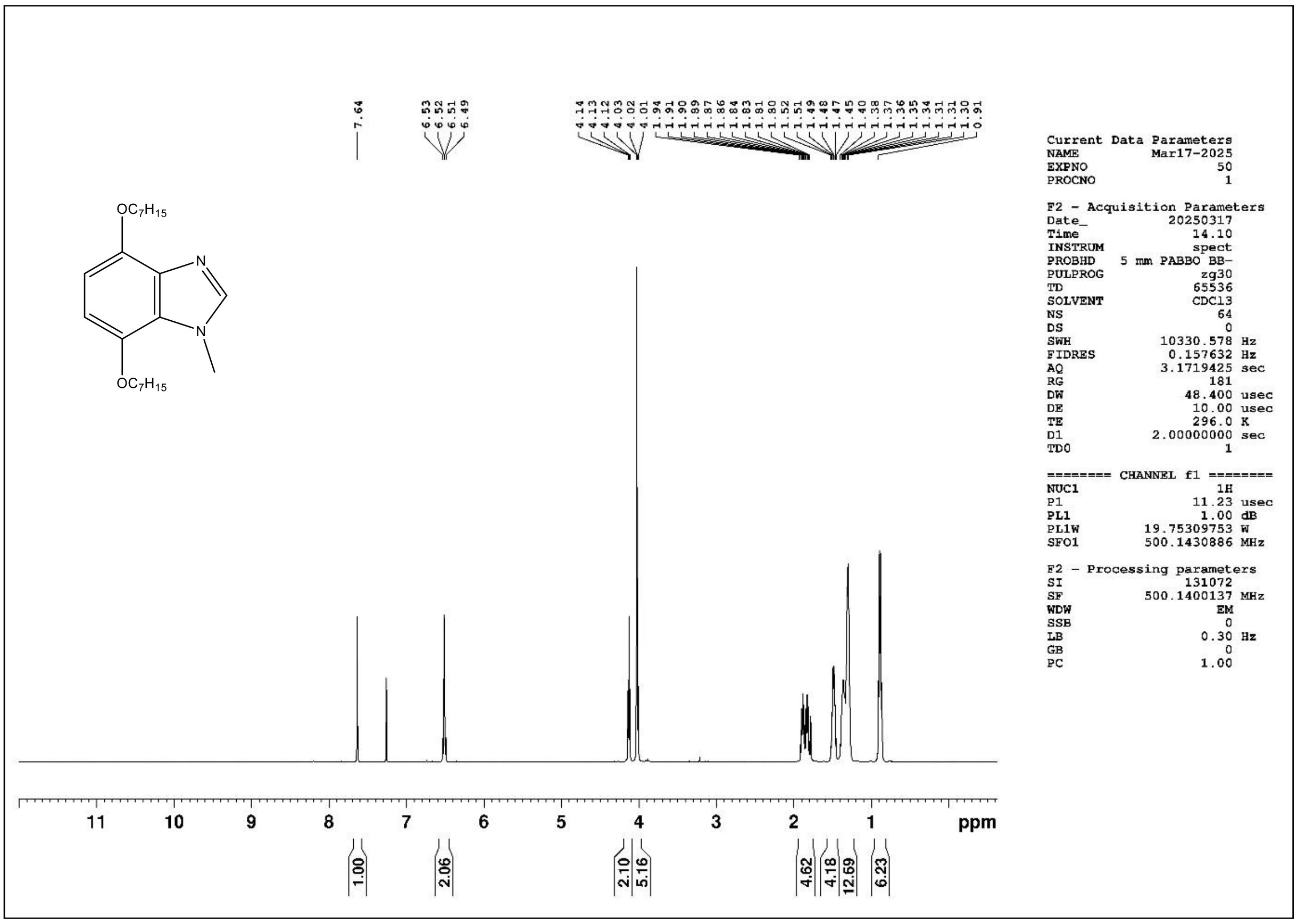

Figure S110. ^{1}H NMR of **L-MeBi(7)**.

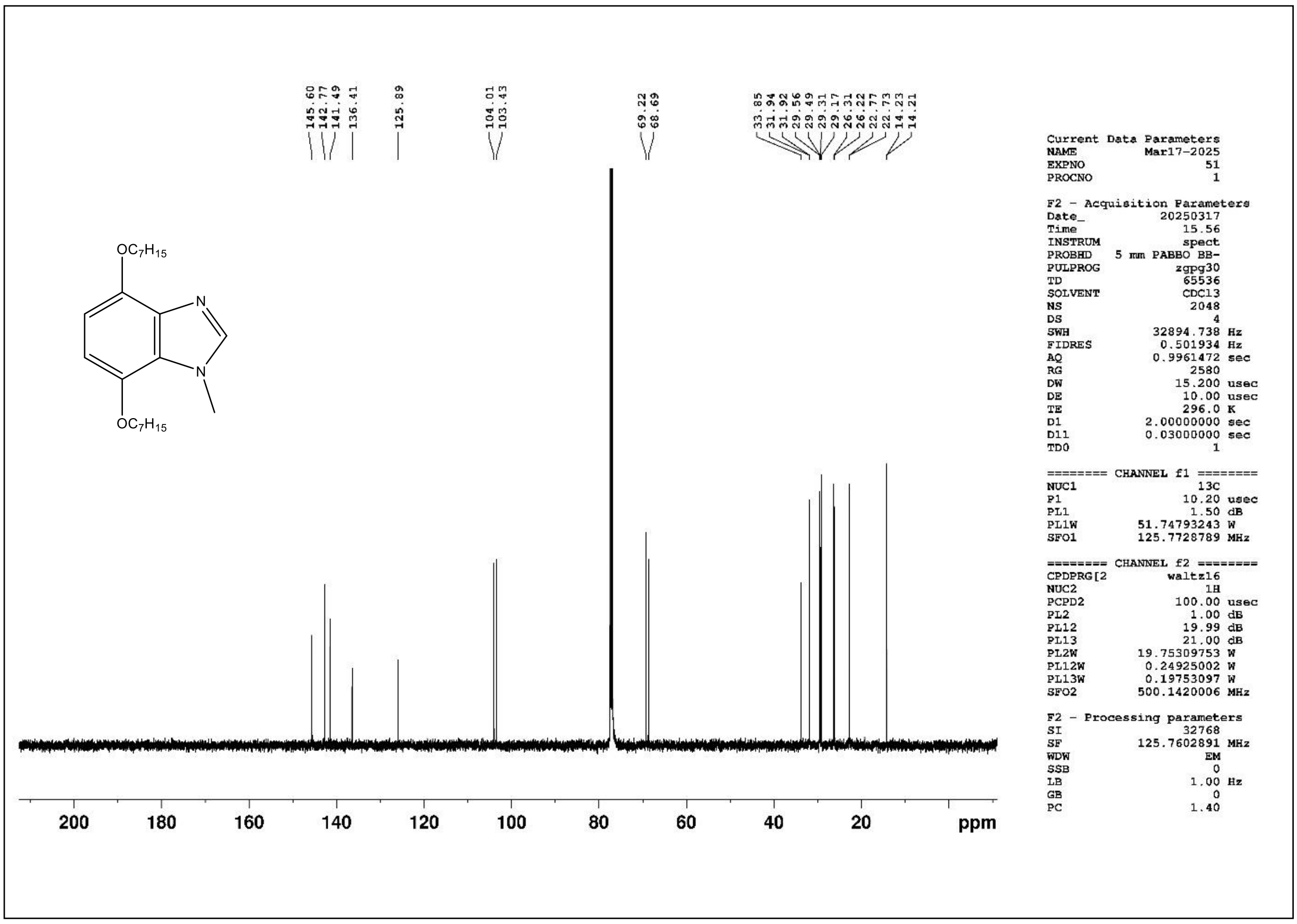

Figure S111. ^{13}C NMR of **L-MeBi(7)**.

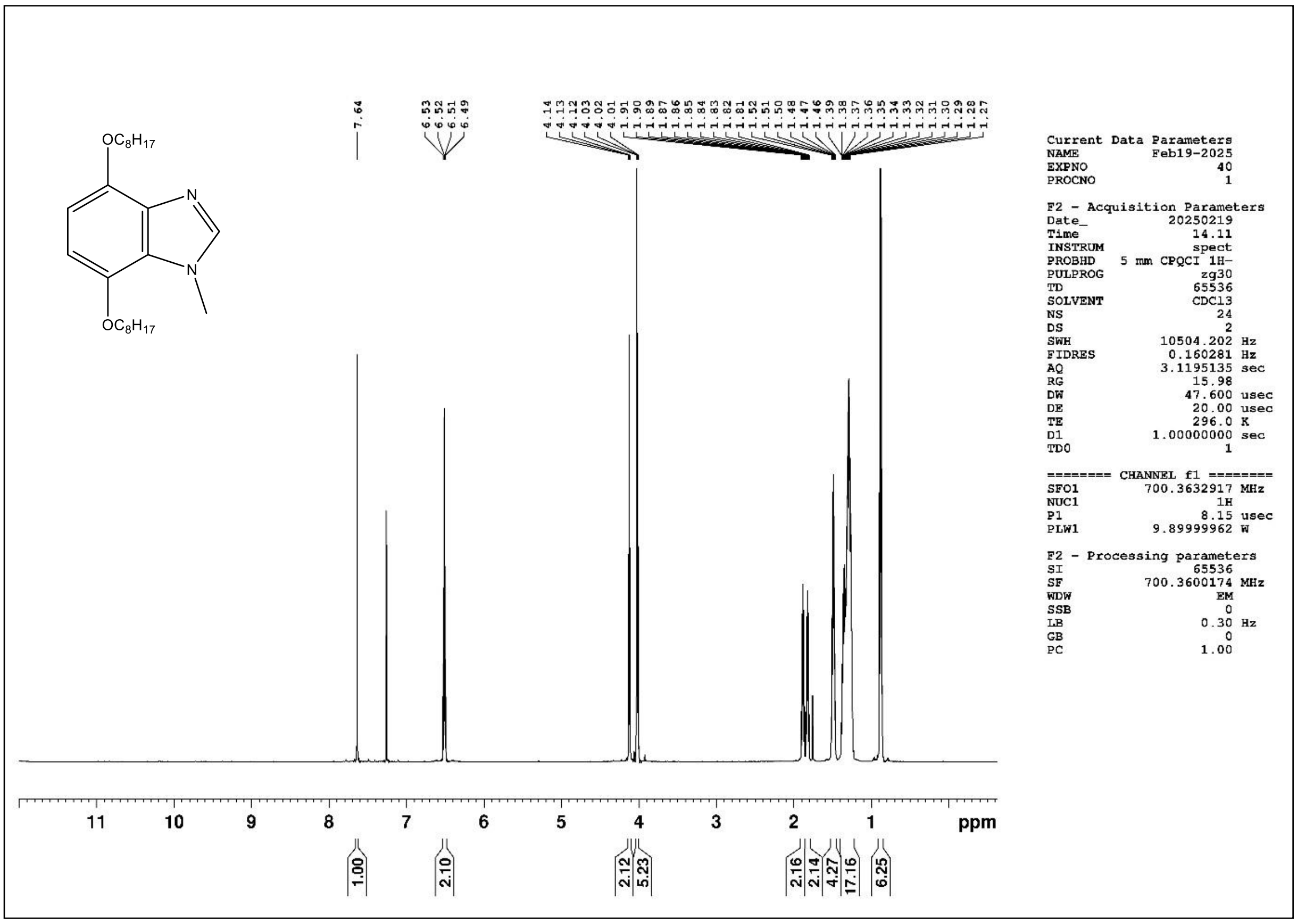

Figure S112. ^{1}H NMR of **L-MeBi(8)**.

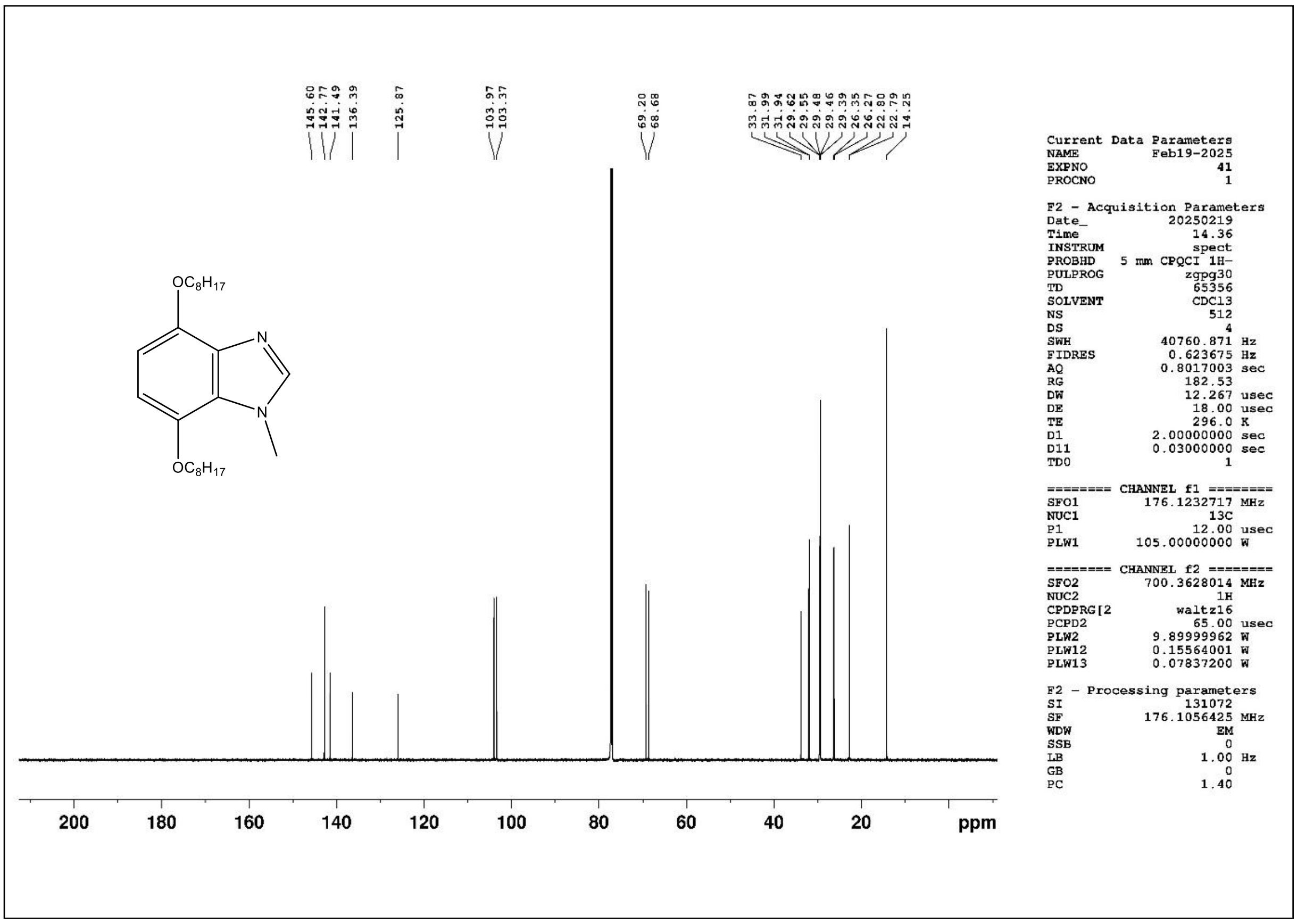

Figure S113.. ^{13}C NMR of **L-MeBi(8)**.

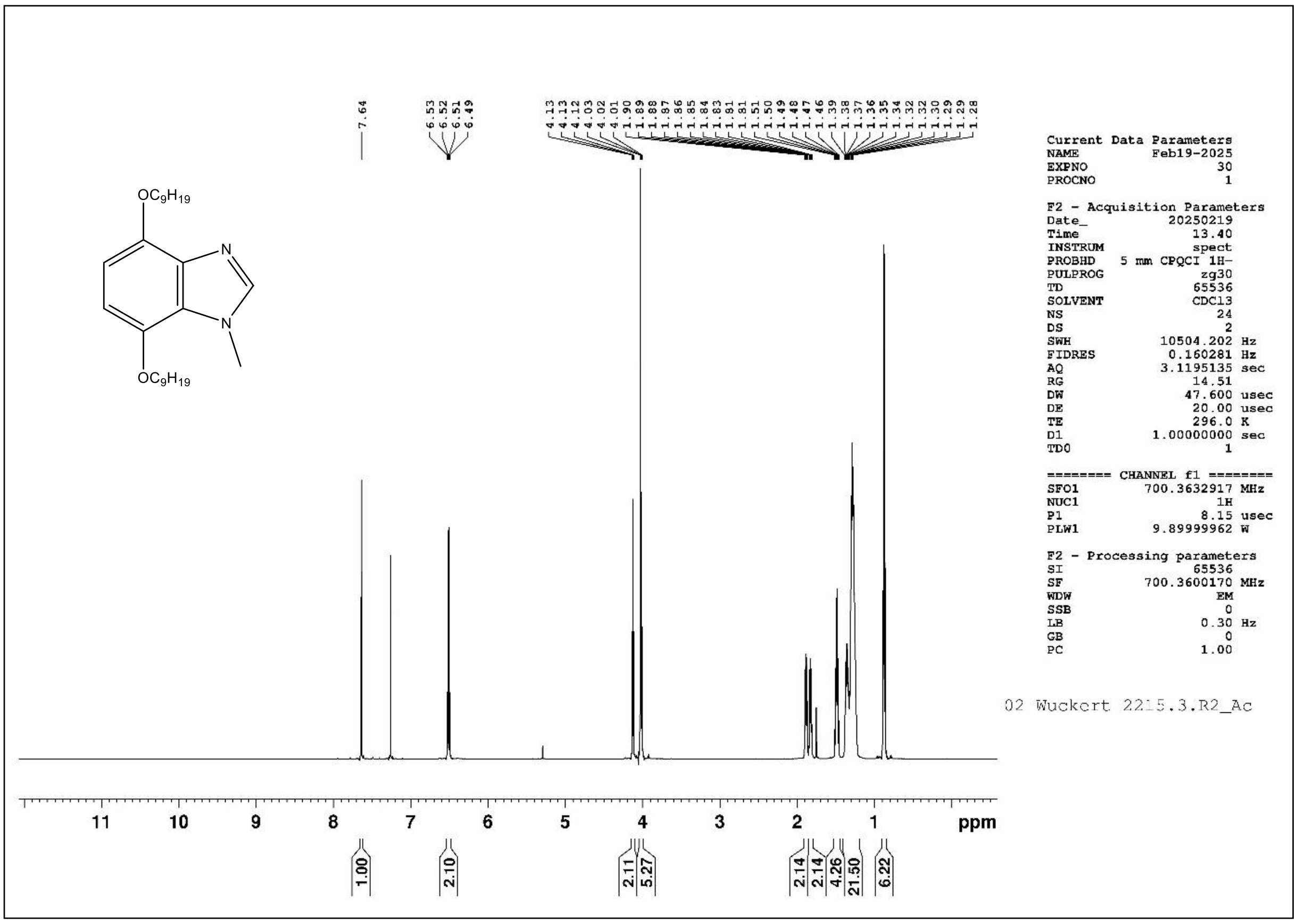

Figure S114. ^{1}H NMR of **L-MeBi(9)**.

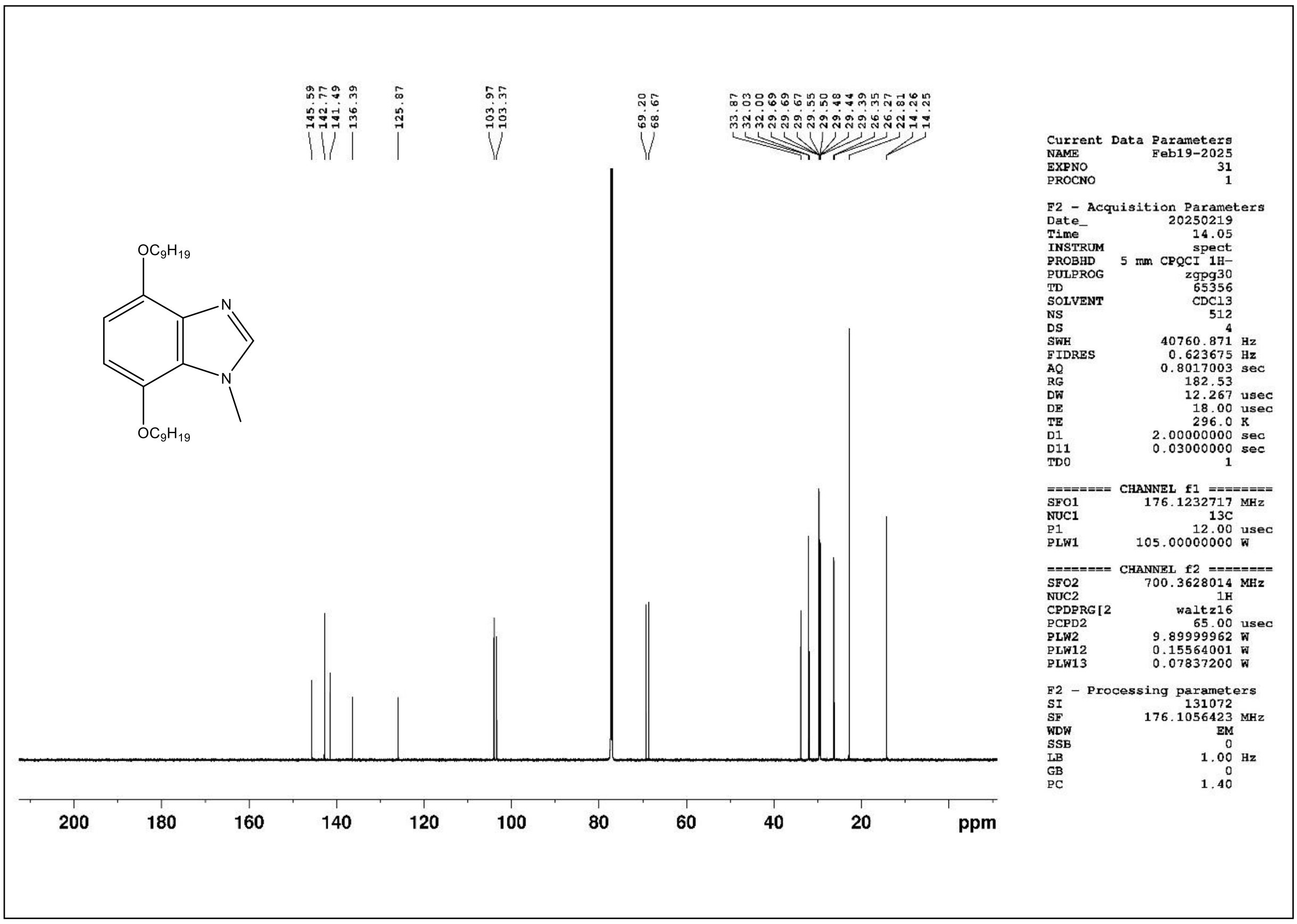

Figure S115. ^{13}C NMR of **L-MeBi(9)**.

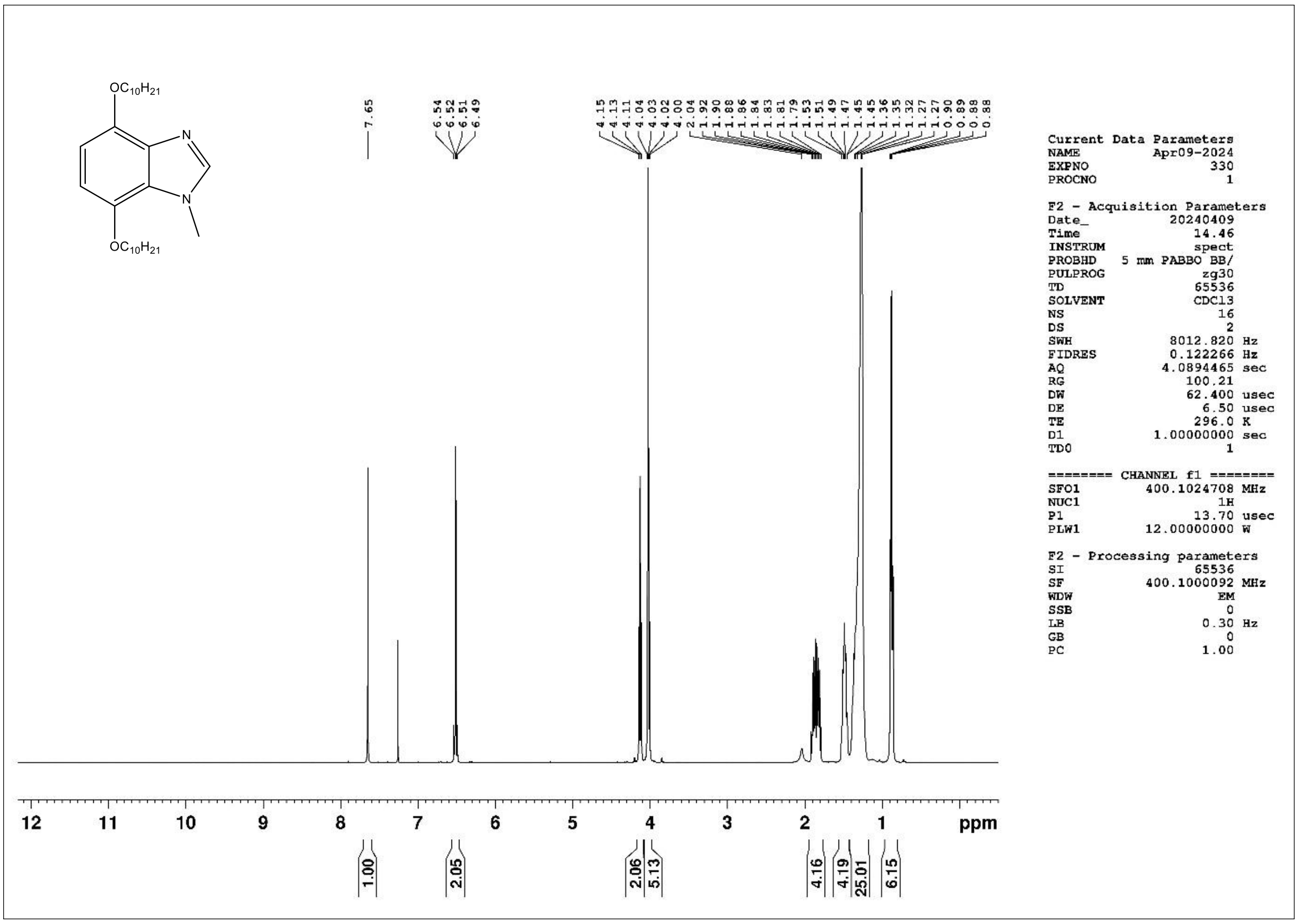

Figure S116. ^{1}H NMR of **L-MeBi(10)**.

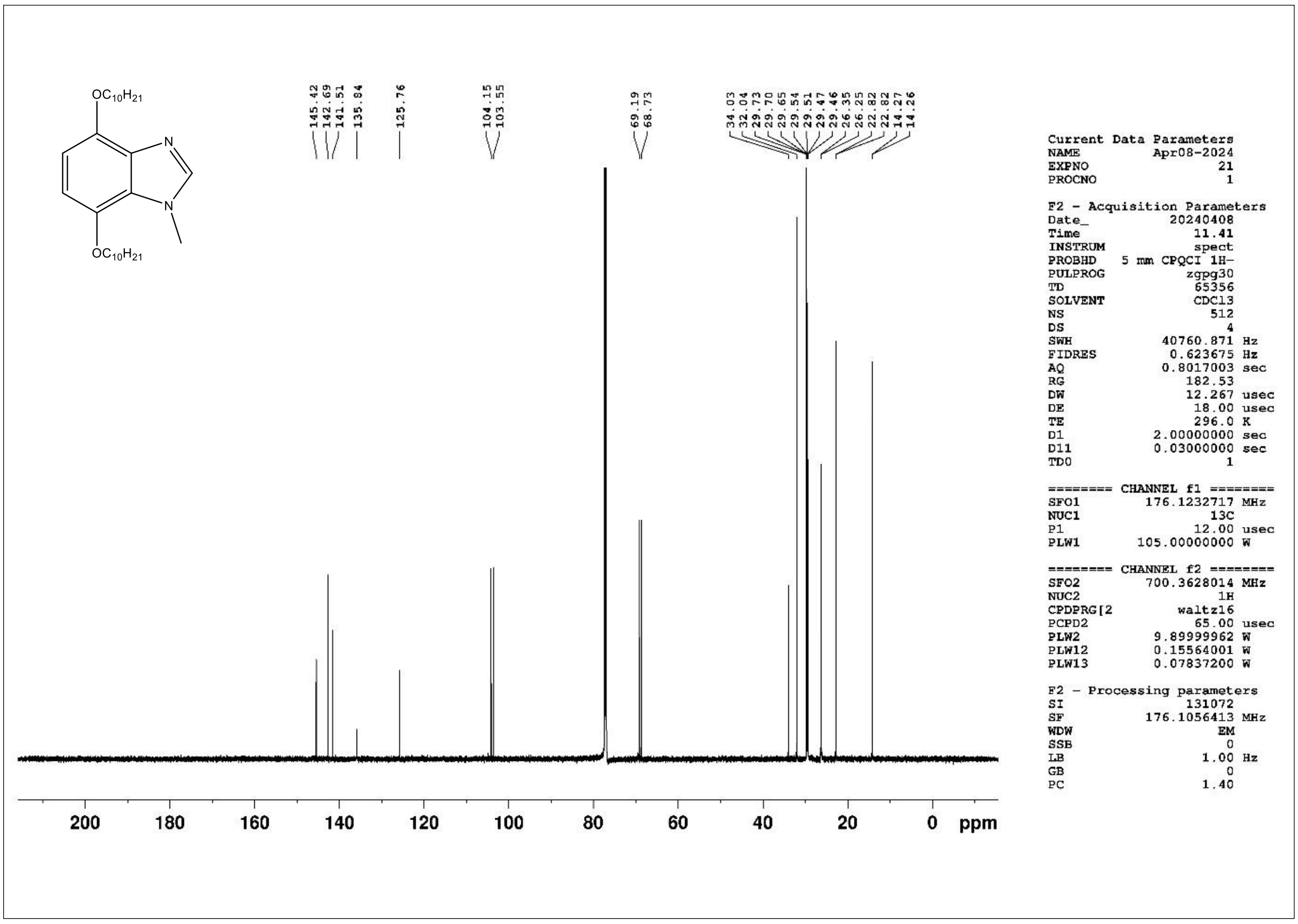

Figure S117. ^{13}C NMR of **L-MeBi(10)**.

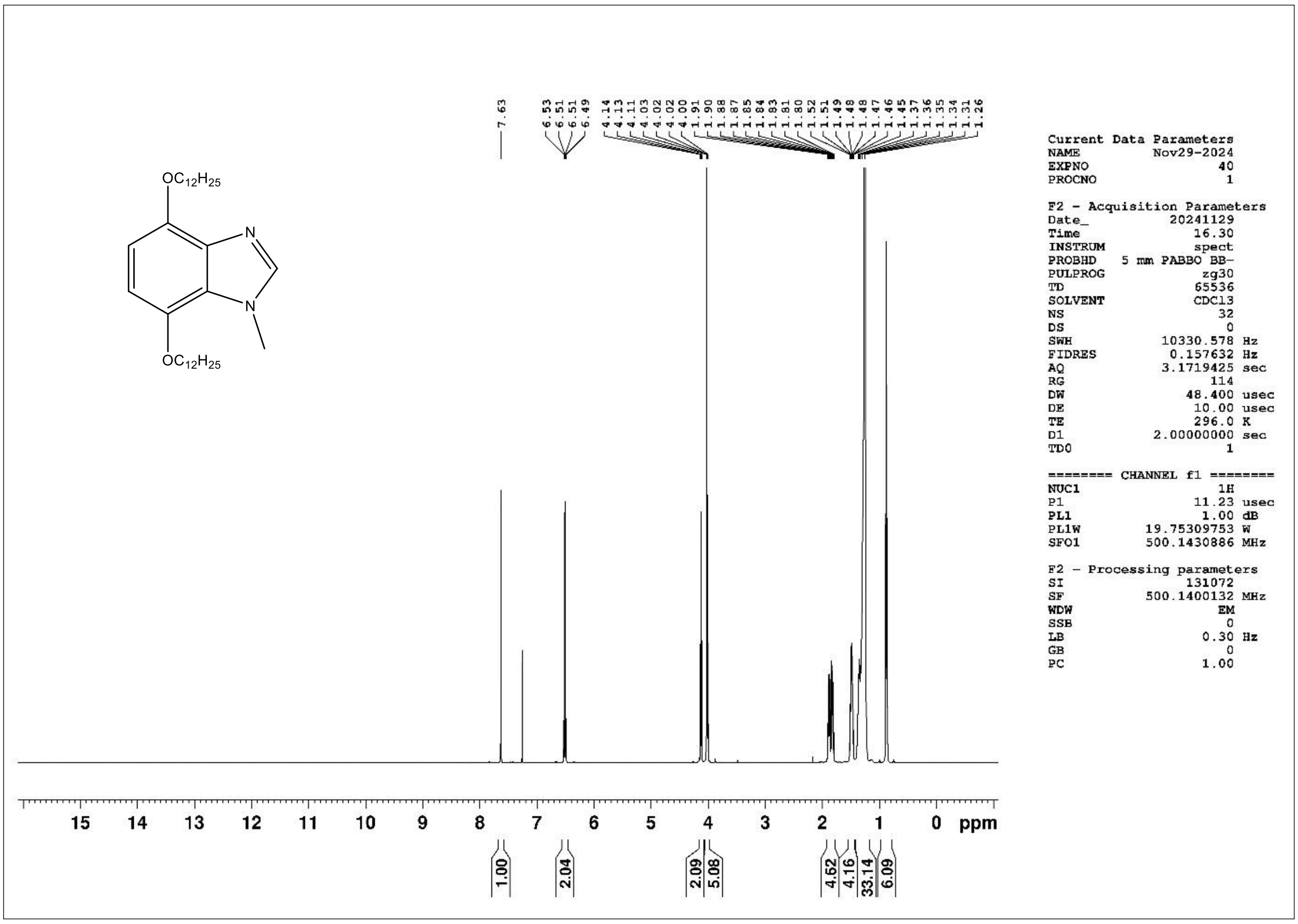

Figure S118. ^{1}H NMR of **L-MeBi(12)**.

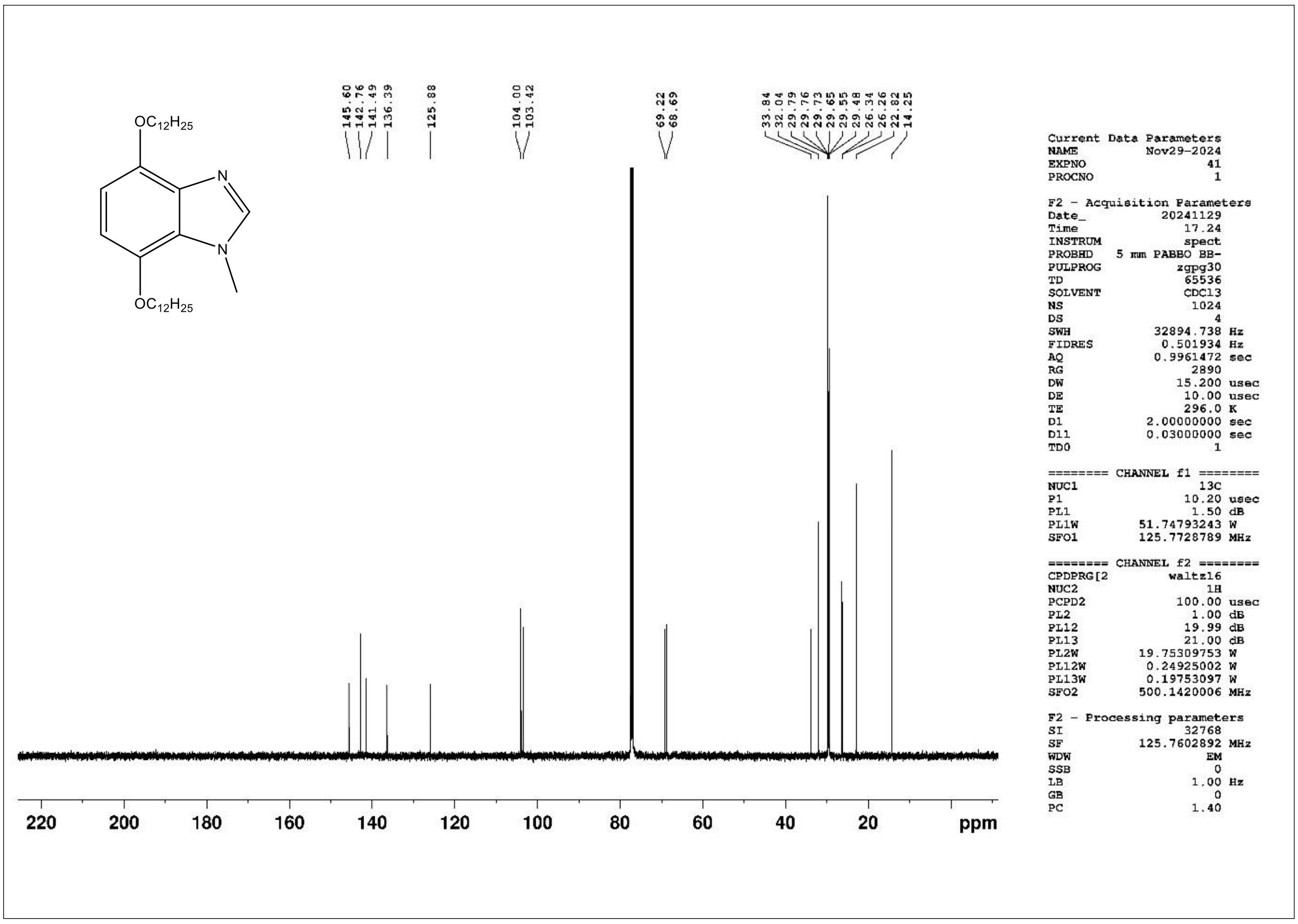

Figure S119. ^{13}C NMR of **L-MeBi(12)**.

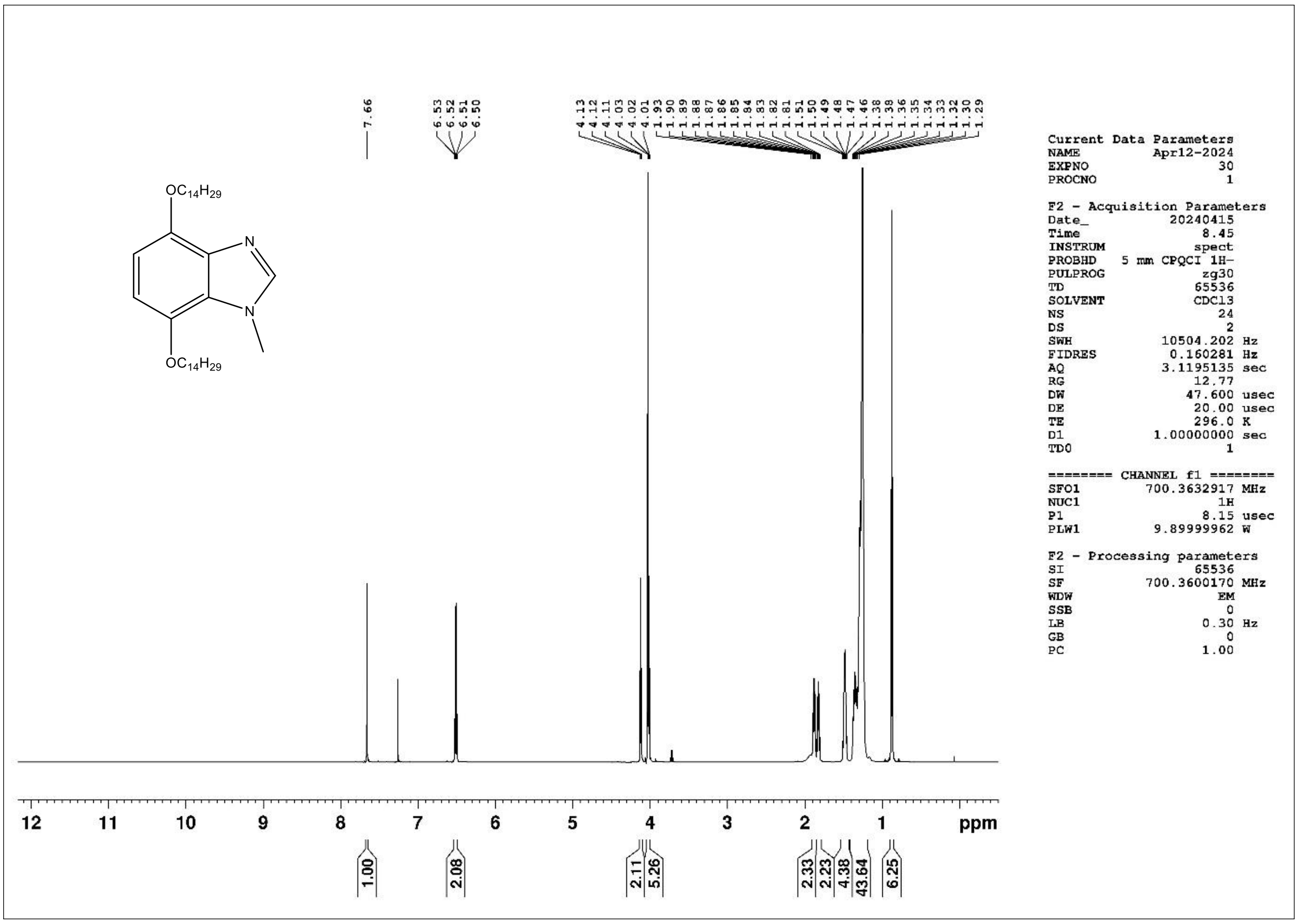

Figure S120. ^{1}H NMR of **L-MeBi(14)**.

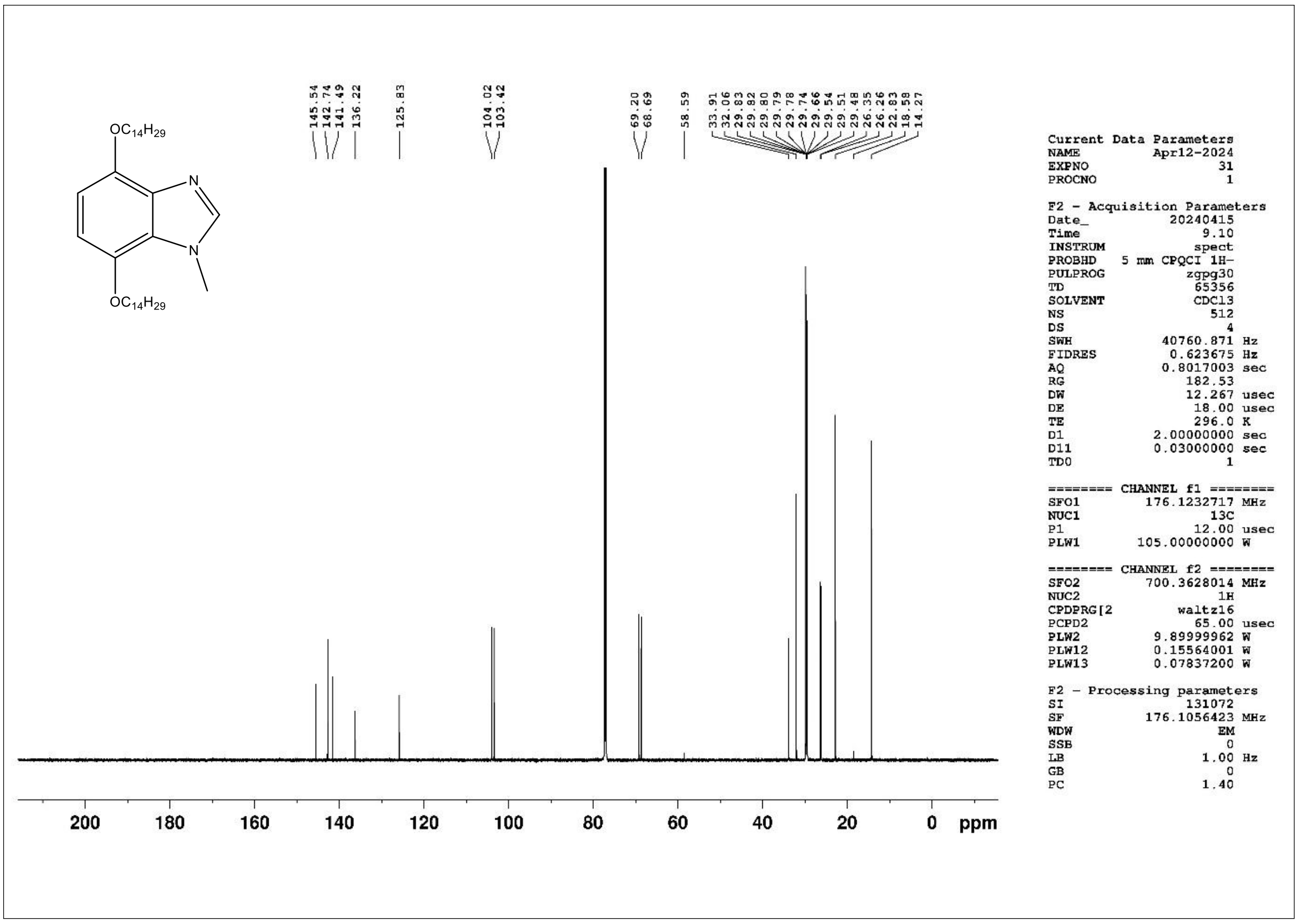

Figure S121. ^{13}C NMR of **L-MeBi(14)**.

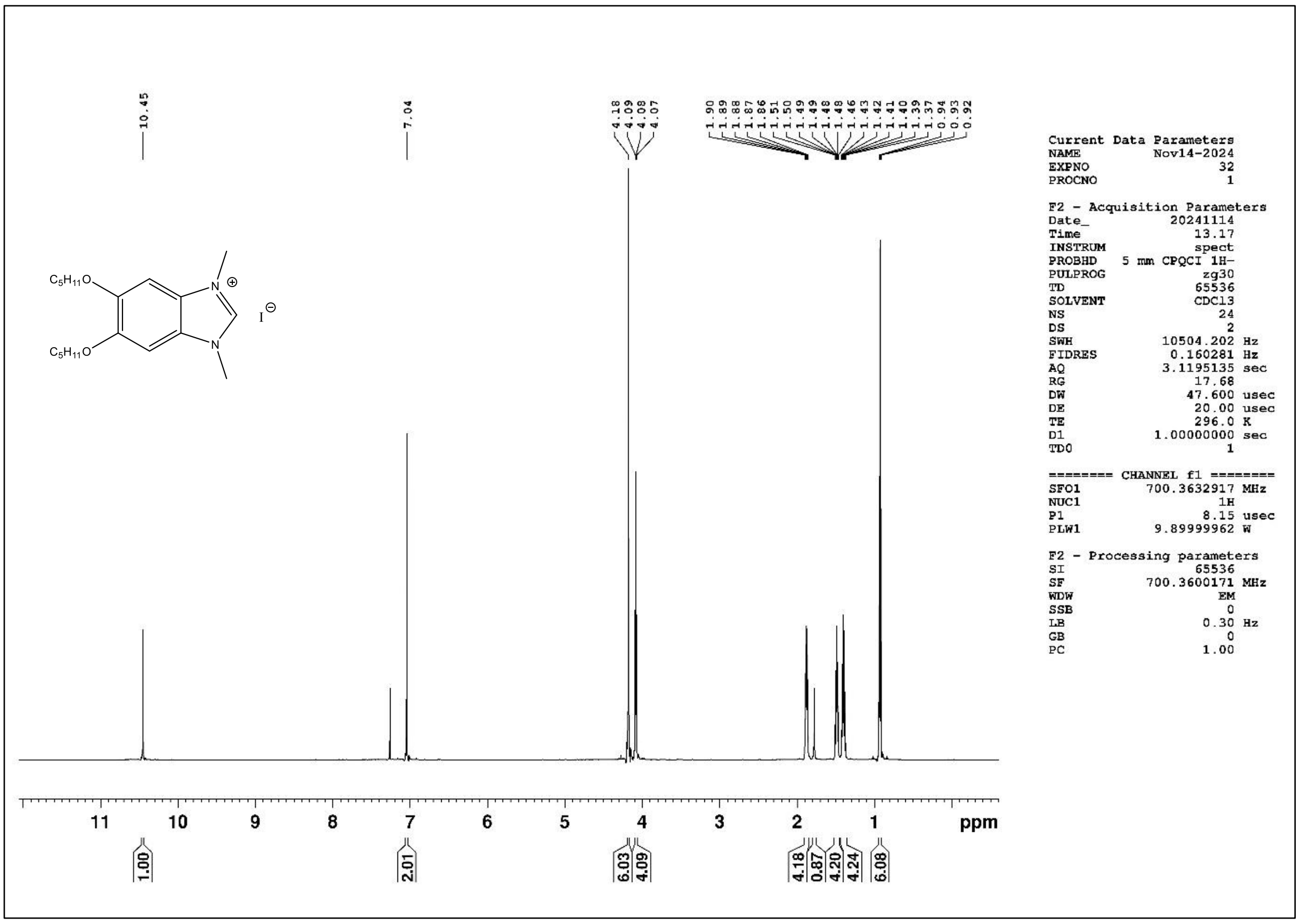

Figure S122. ^{1}H NMR of **U-Me$_2$Bi(5)I**.

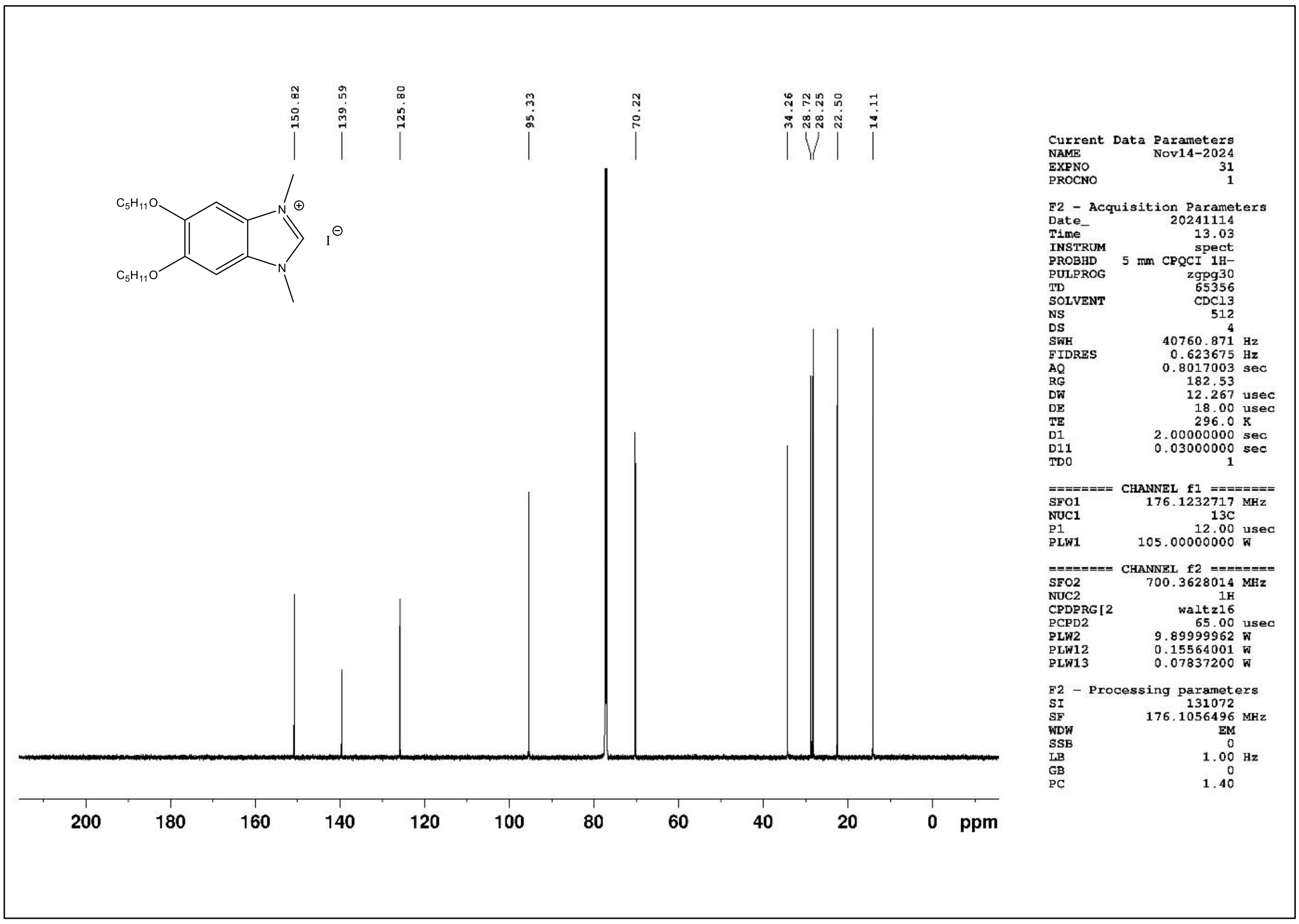

Figure S123. ^{13}C NMR of **U-Me$_2$Bi(5)I**.

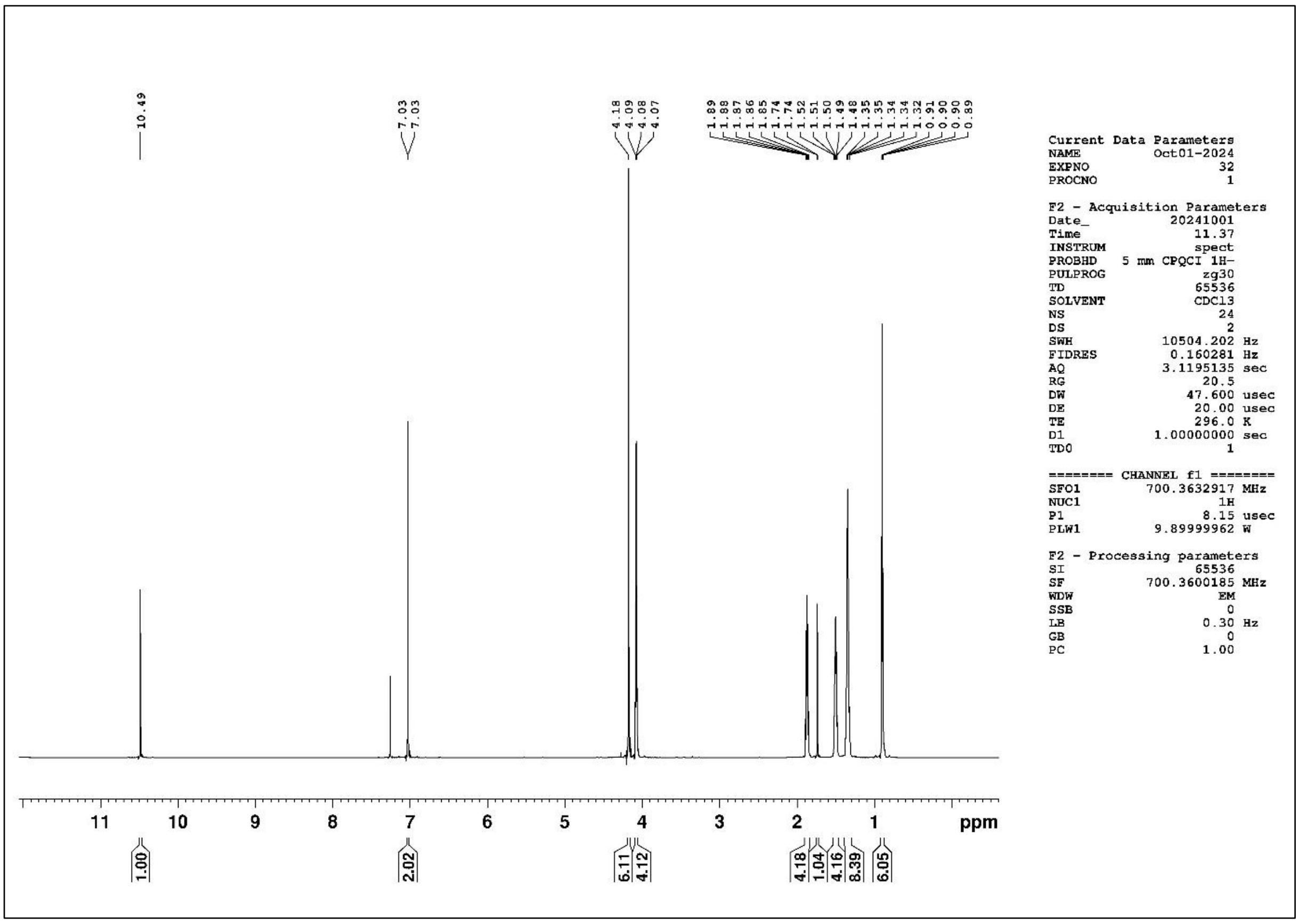

Figure S124. ^{1}H NMR of **U-Me$_2$Bi(6)I**.

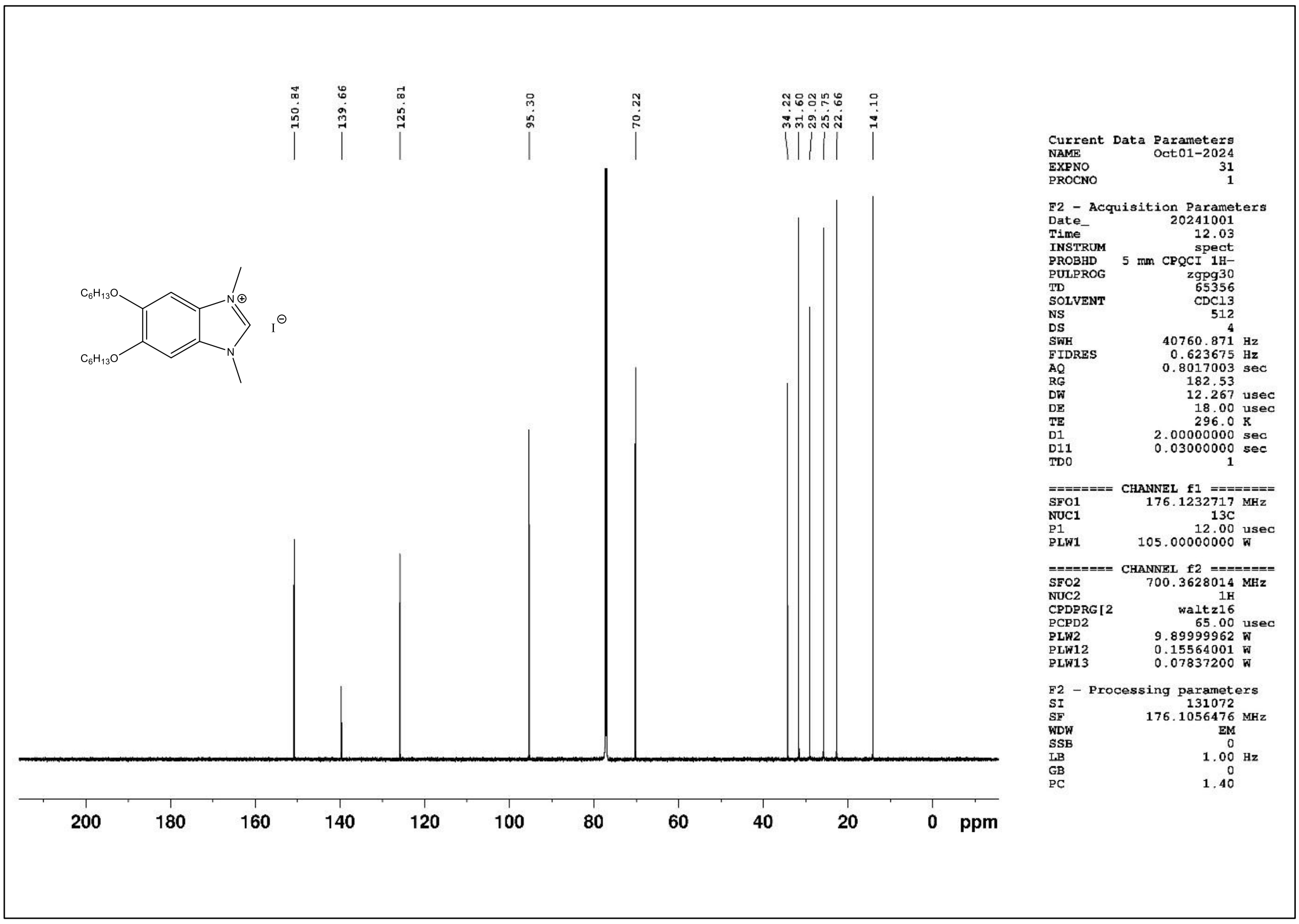

Figure S125. ^{13}C NMR of **U-Me$_2$Bi(6)I**.

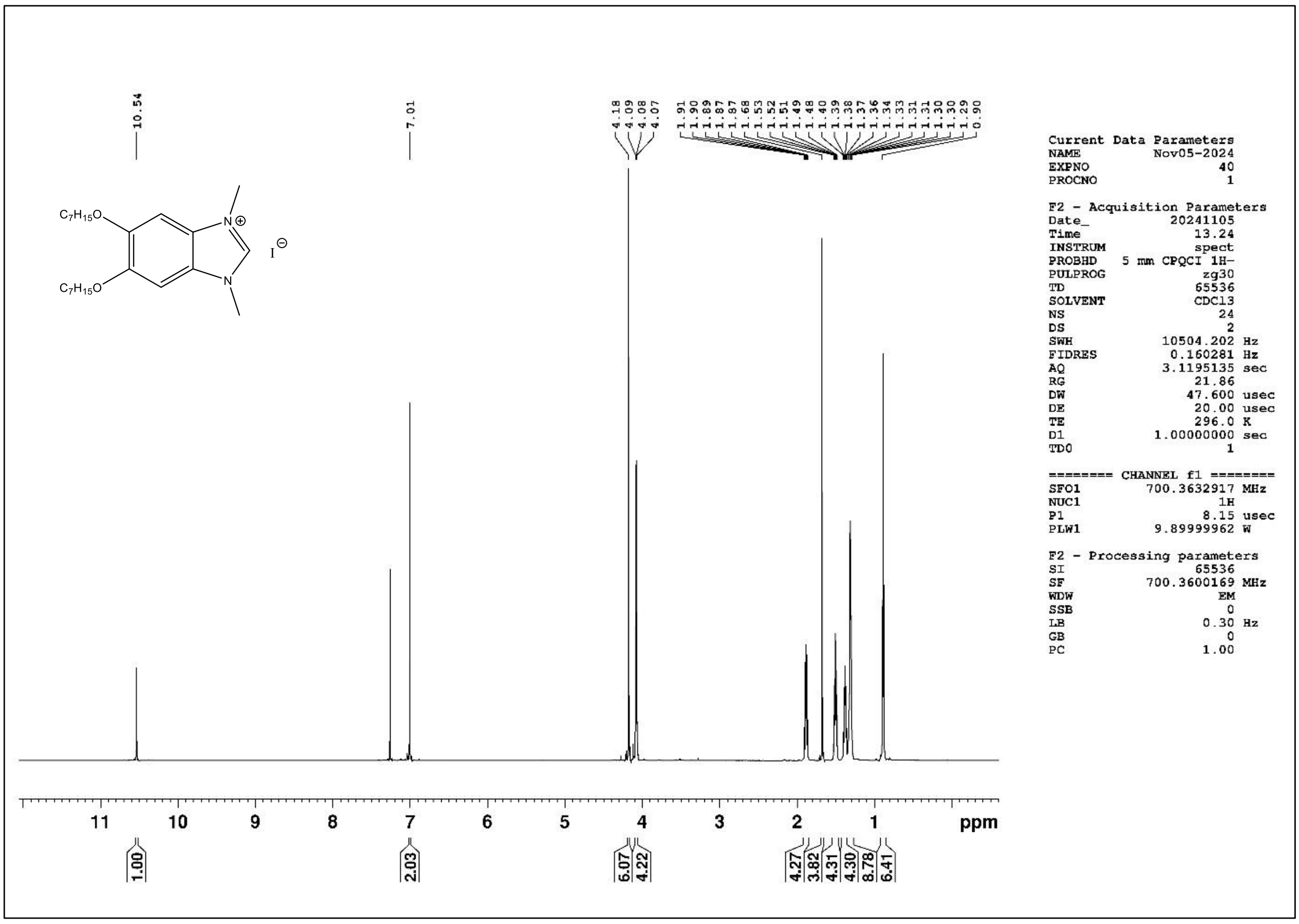

Figure S126. ^{1}H NMR of **U-Me$_2$Bi(7)I**.

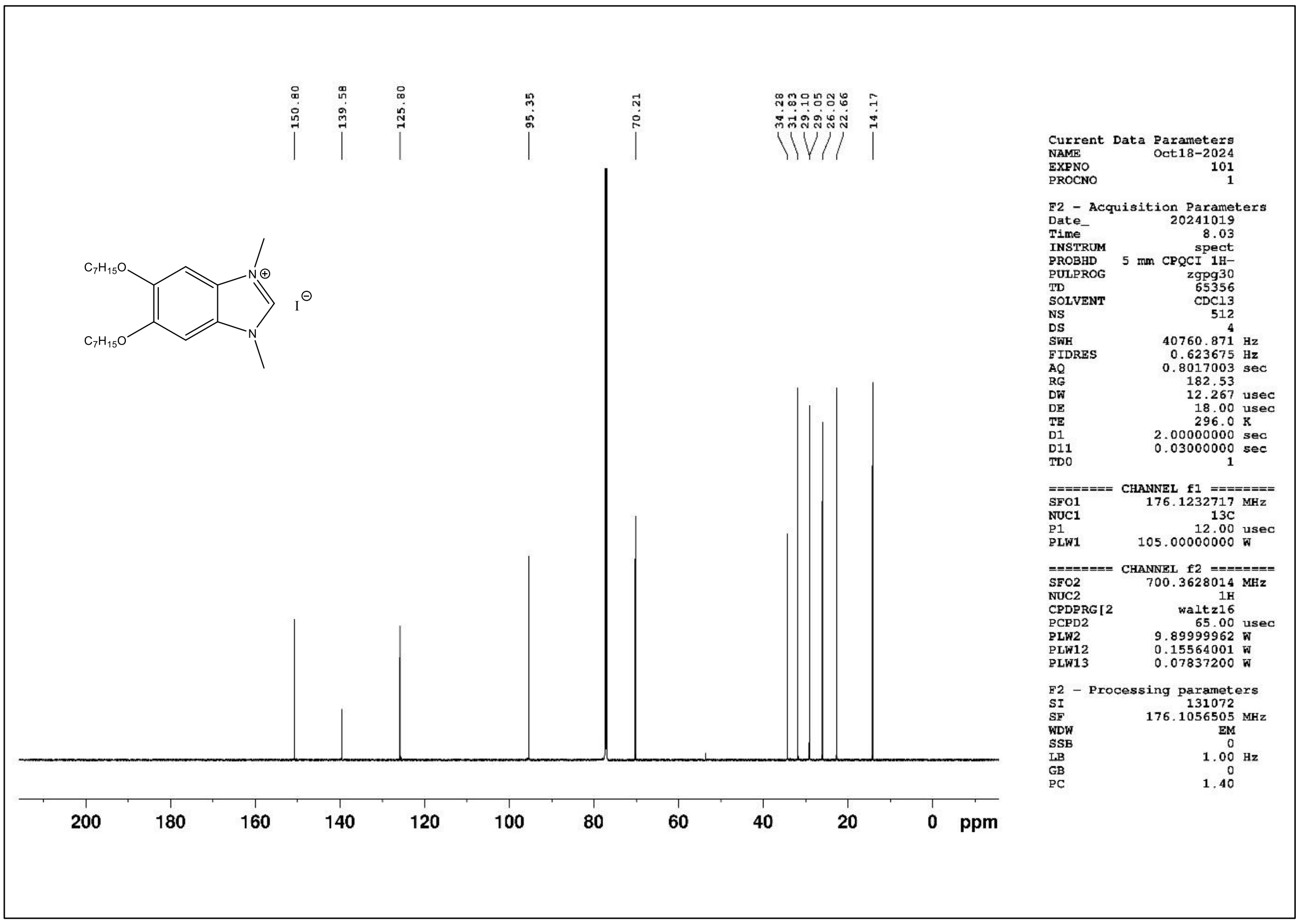

Figure S127. ^{13}C NMR of **U-Me$_2$Bi(7)I**.

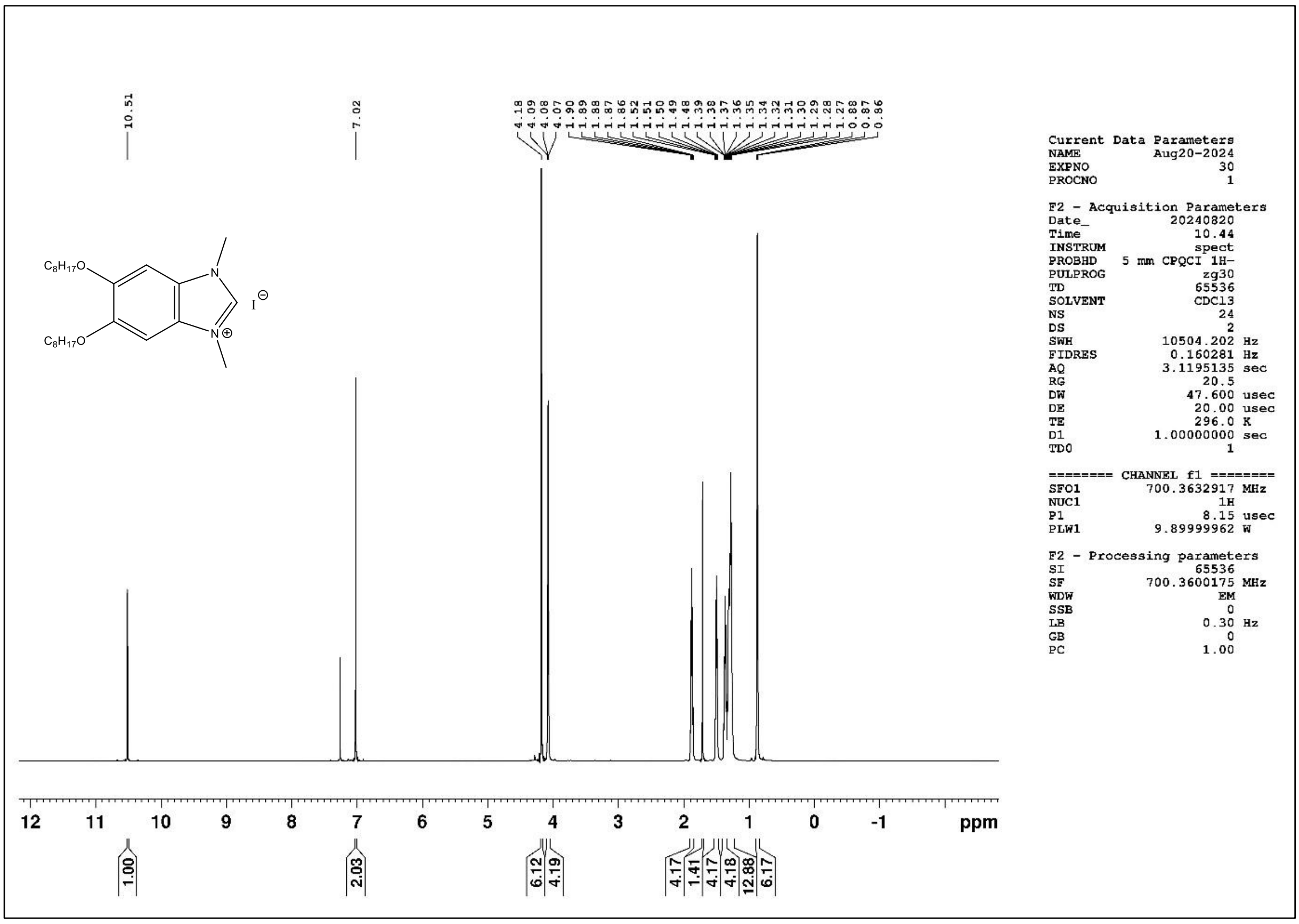

Figure S128. ^{1}H NMR of **U-Me$_2$Bi(8)I**.

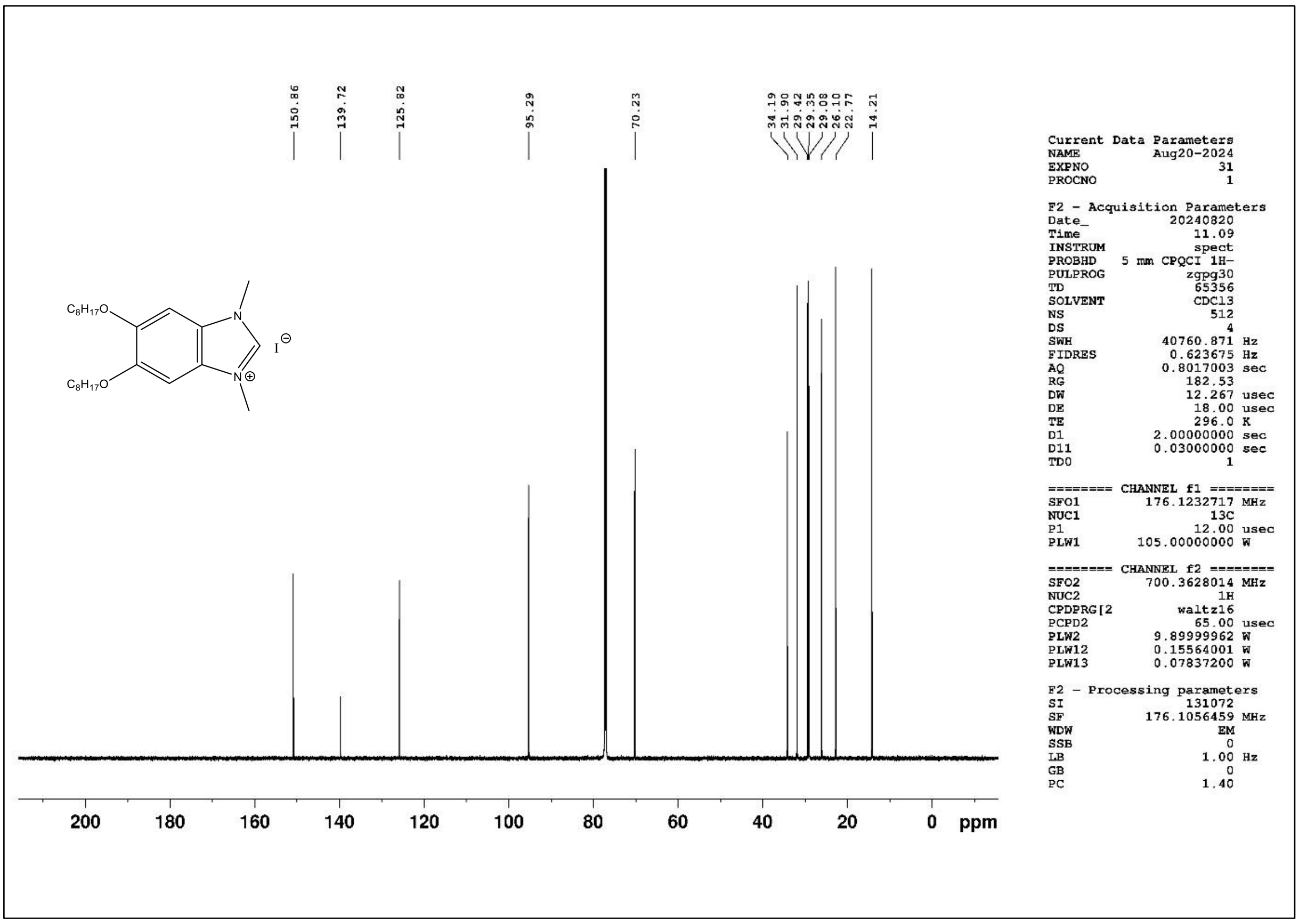

Figure S129. ^{13}C NMR of **U-Me$_2$Bi(8)I**.

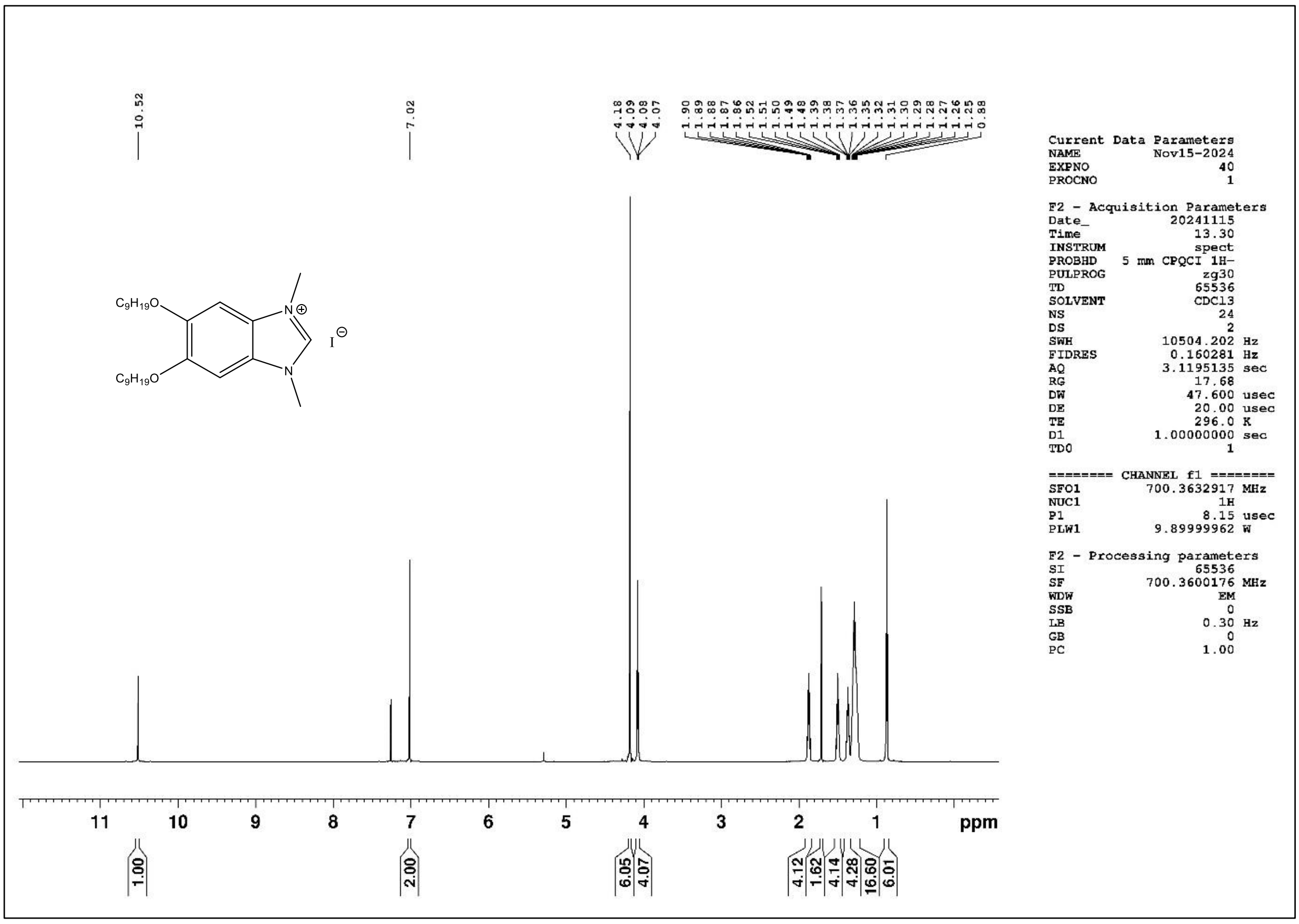

Figure S130. ^{1}H NMR of **U-Me$_2$Bi(9)I**.

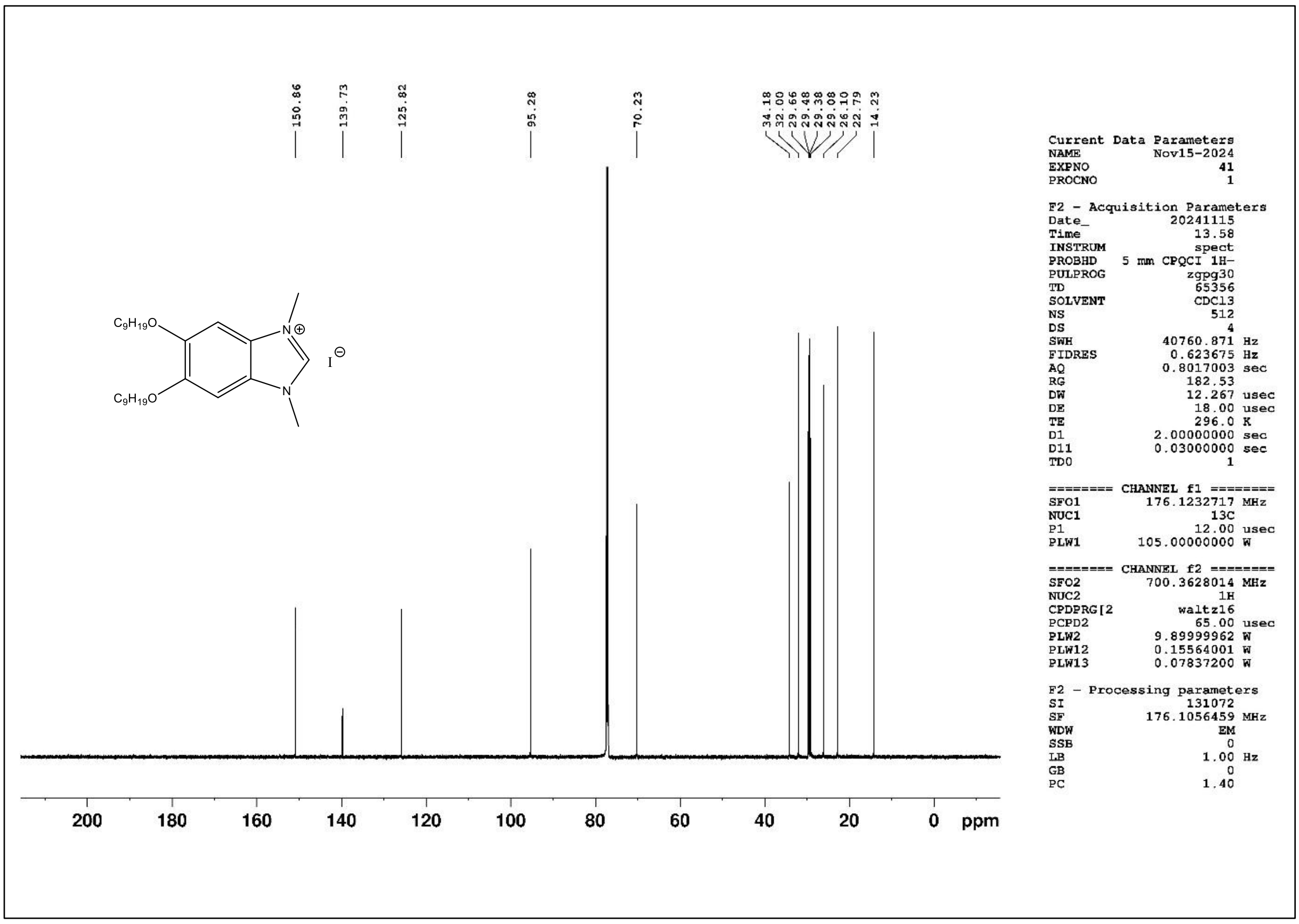

Figure S131.. ^{13}C NMR of **U-Me$_2$Bi(9)I**.

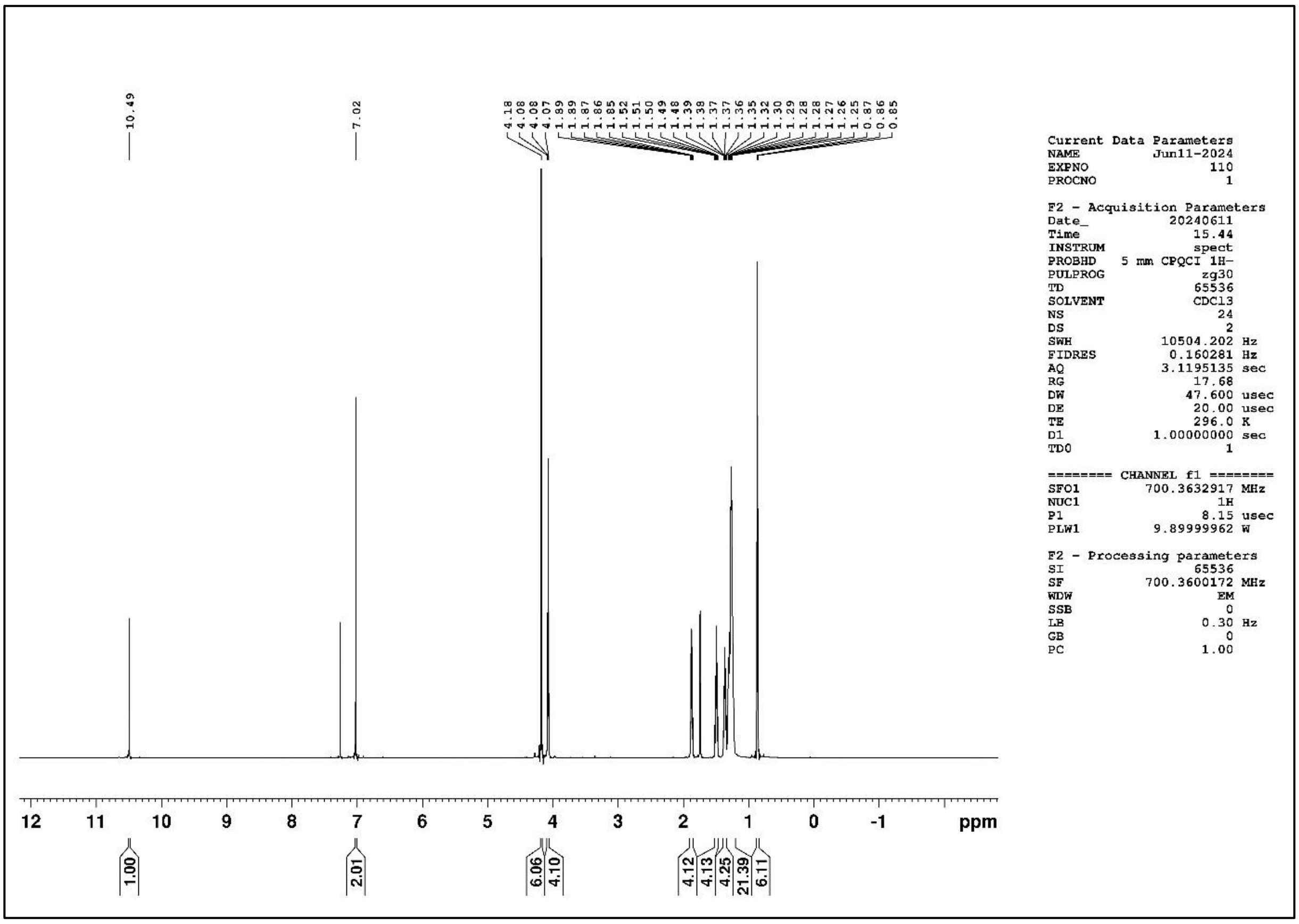

Figure S132. ^{1}H NMR of **U-Me$_2$Bi(10)I**.

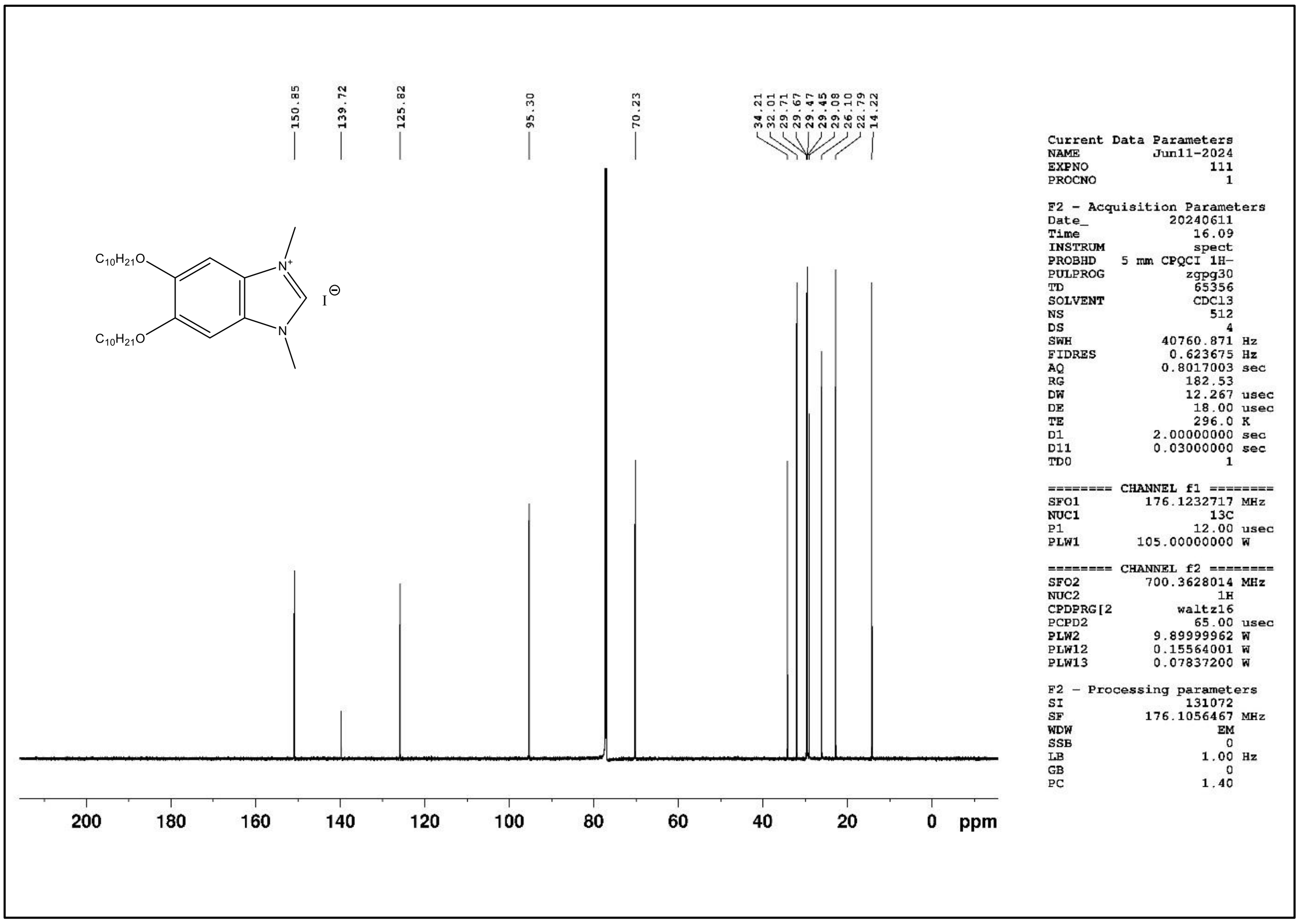

Figure S133. ^{13}C NMR of **U-Me$_2$Bi(10)I**.

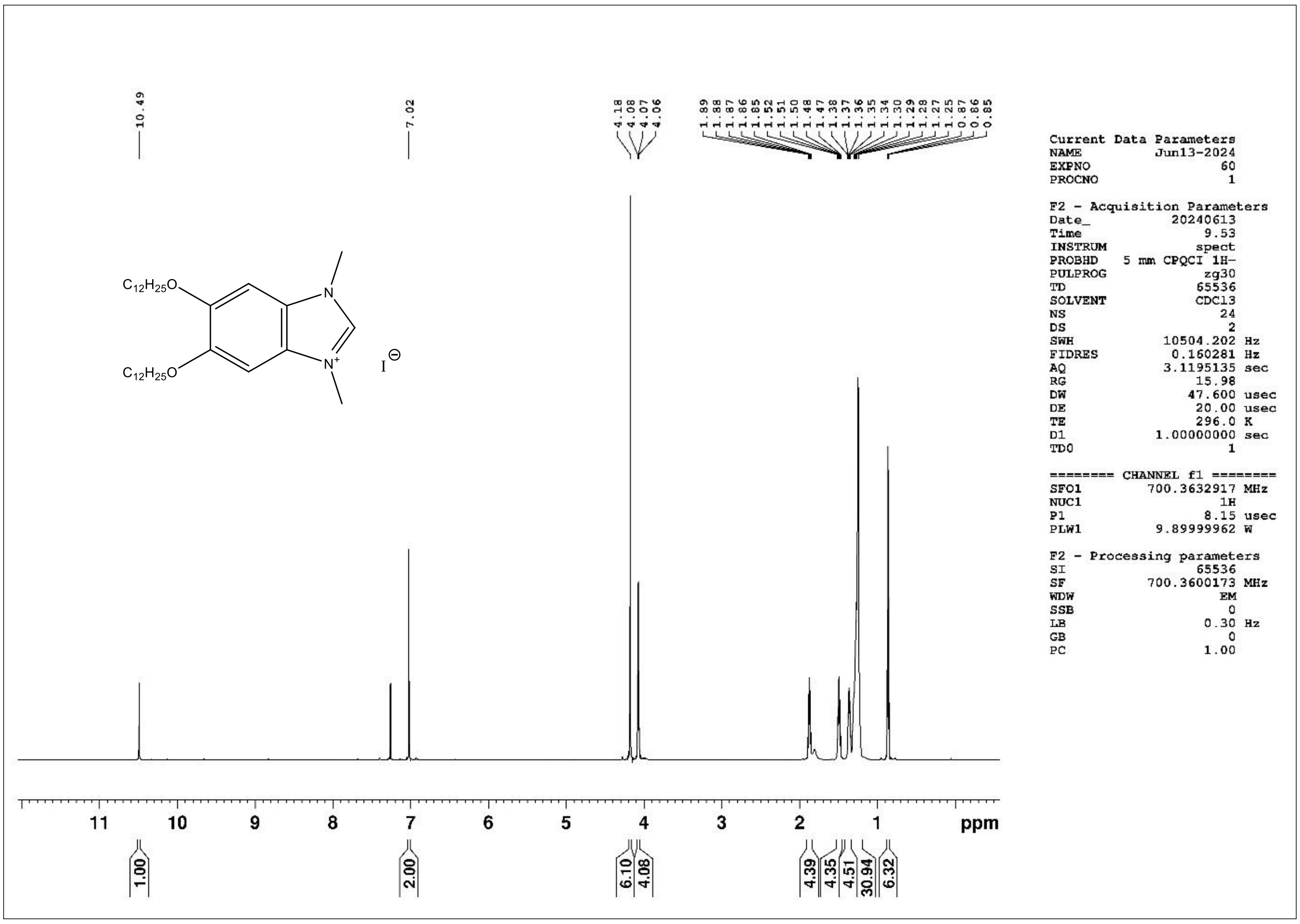

Figure S134. ^{1}H NMR of **U-Me$_2$Bi(12)I**..

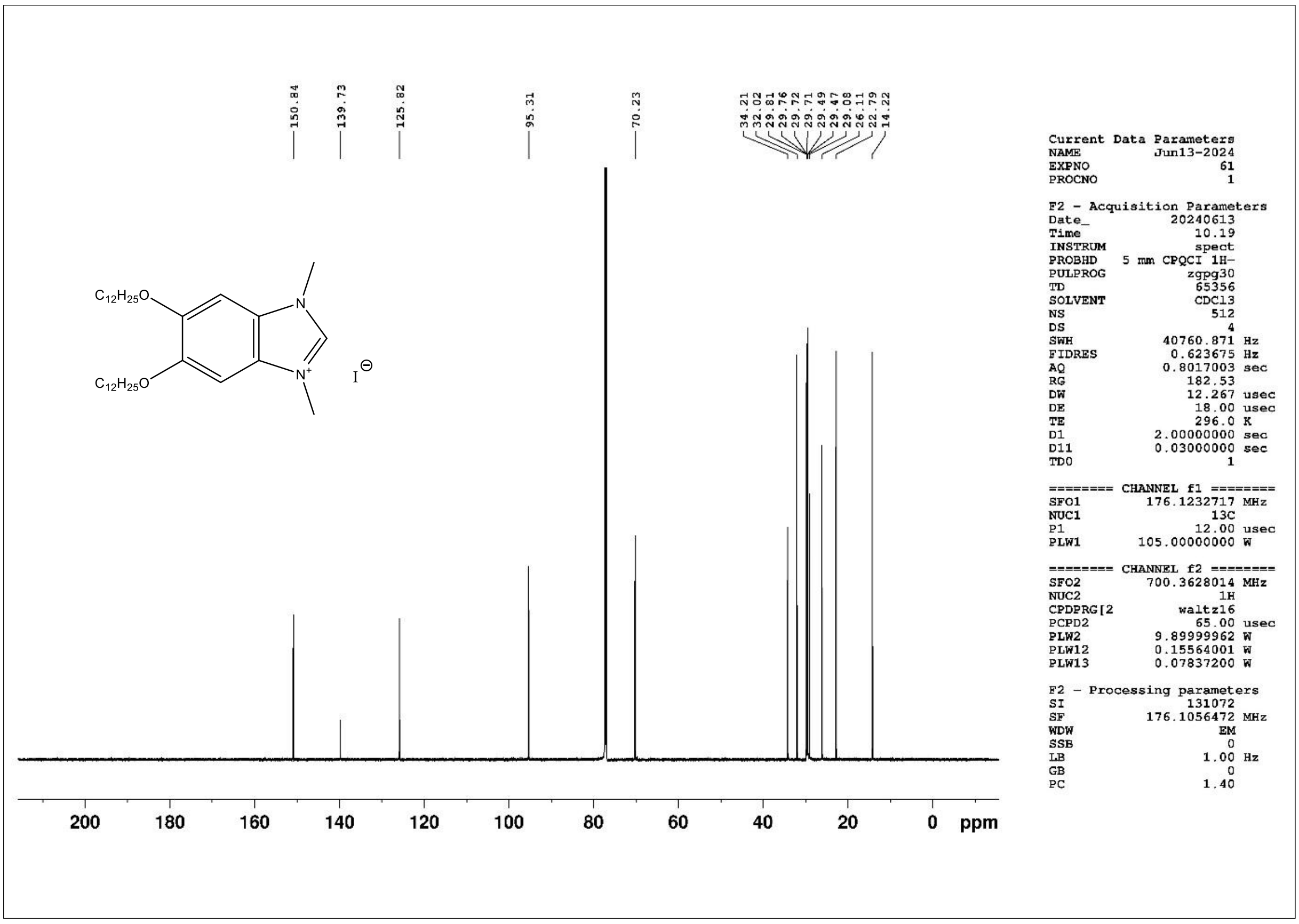

Figure S135. ^{13}C NMR of **U-Me$_2$Bi(12)I**.

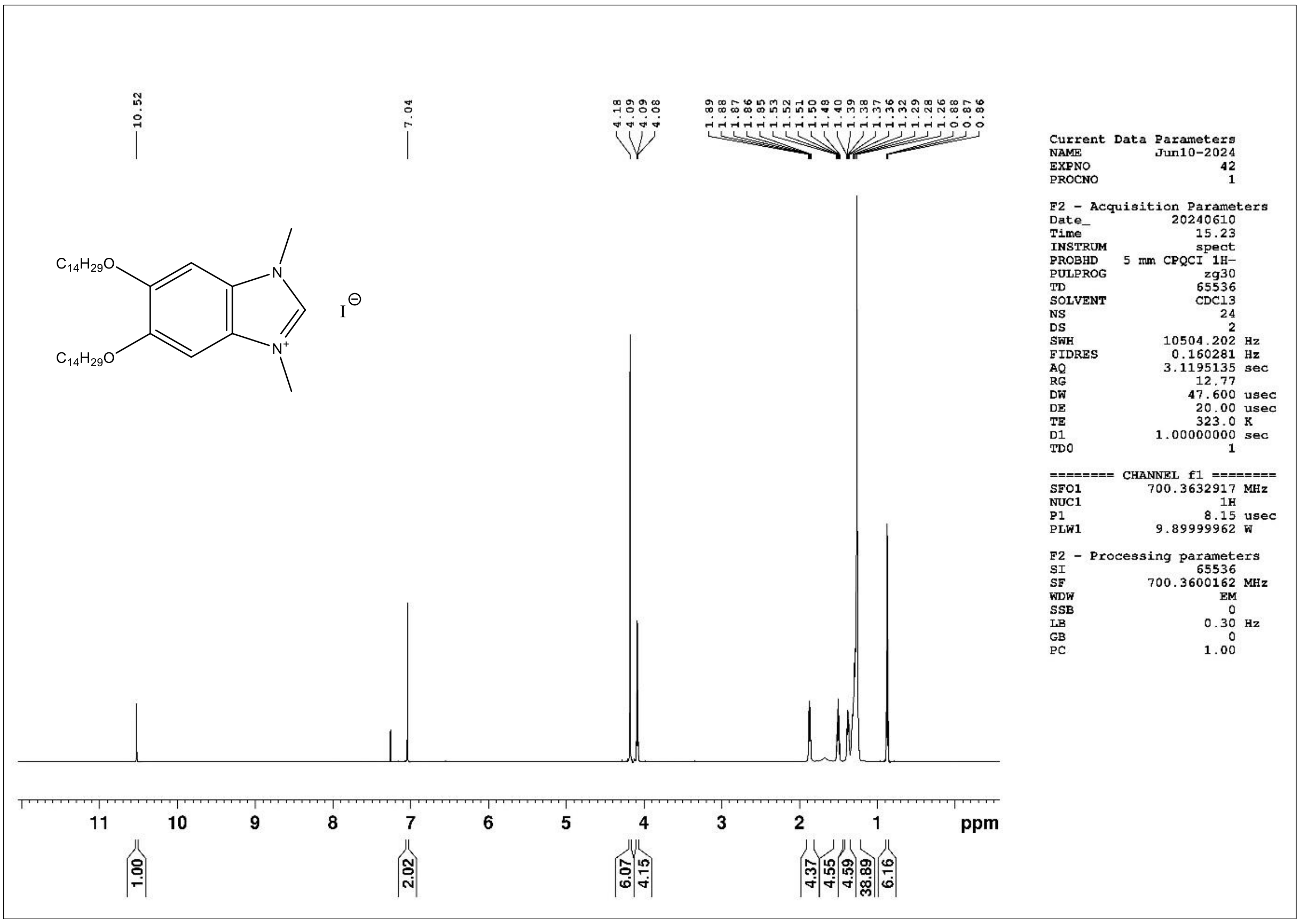

Figure S136. ^{1}H NMR of **U-Me$_2$Bi(14)I**.

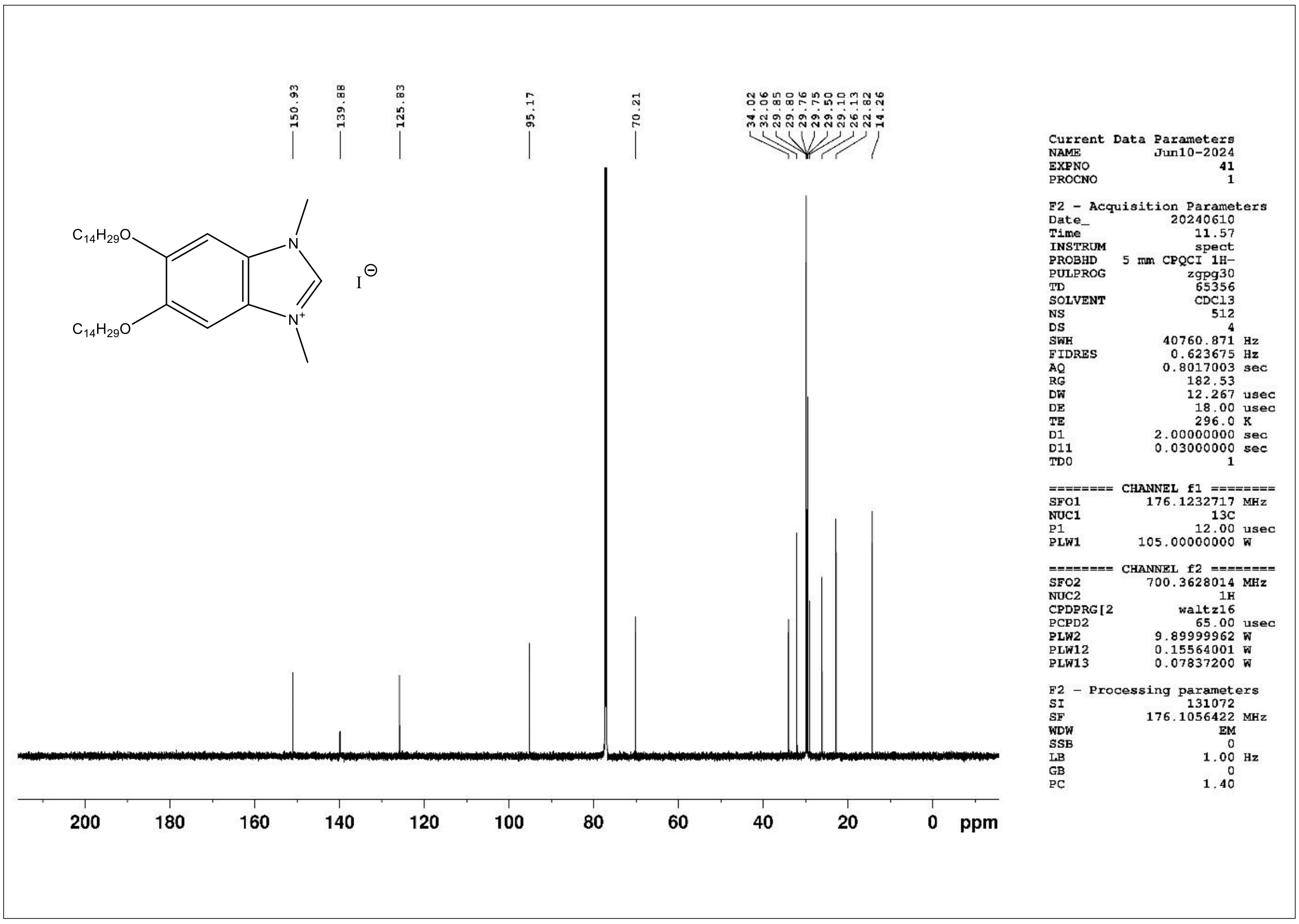

Figure S137. ^{13}C NMR of **U-Me$_2$Bi(14)I**.

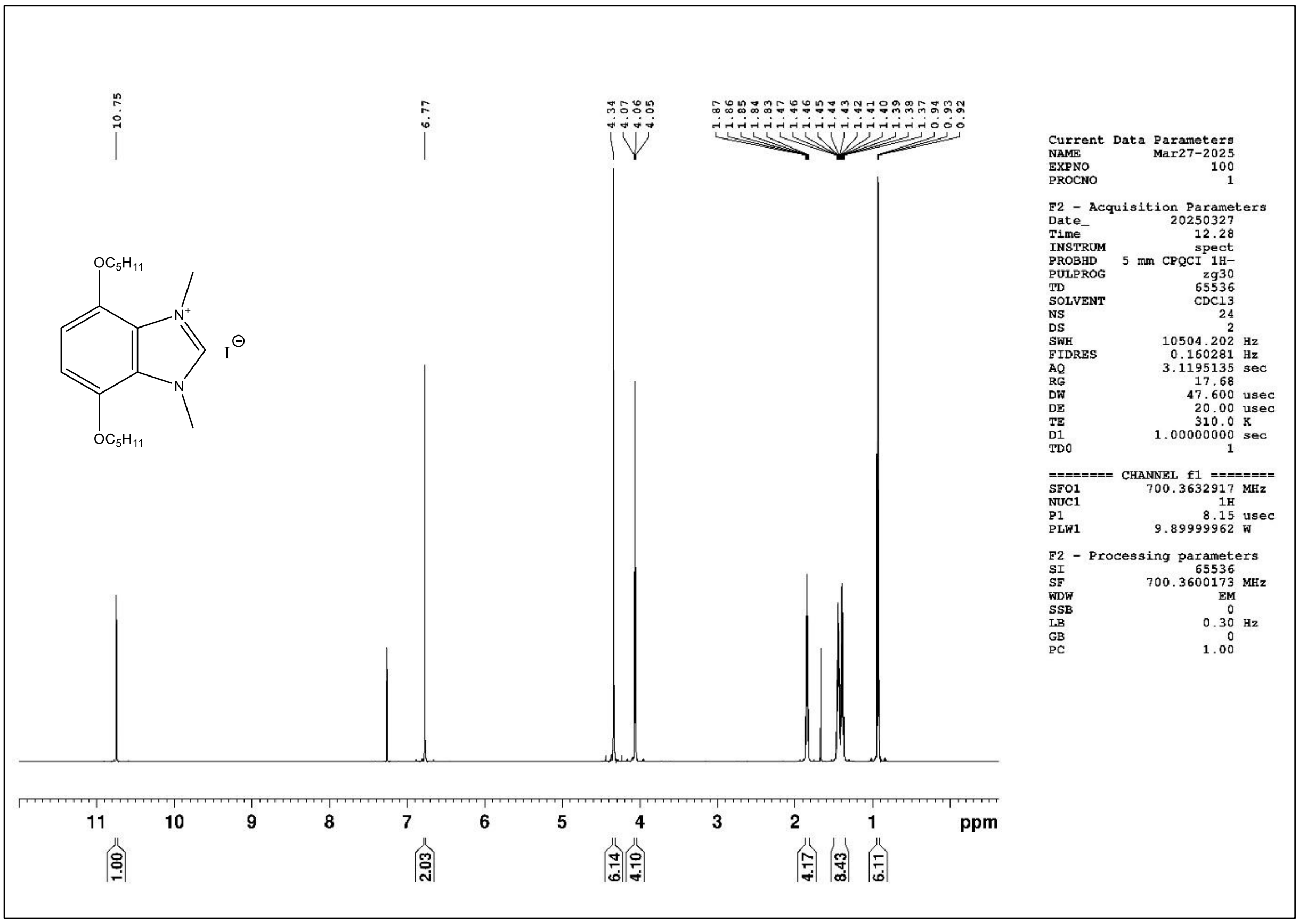

Figure S138. ^{1}H NMR of **L-Me$_2$Bi(5)I**.

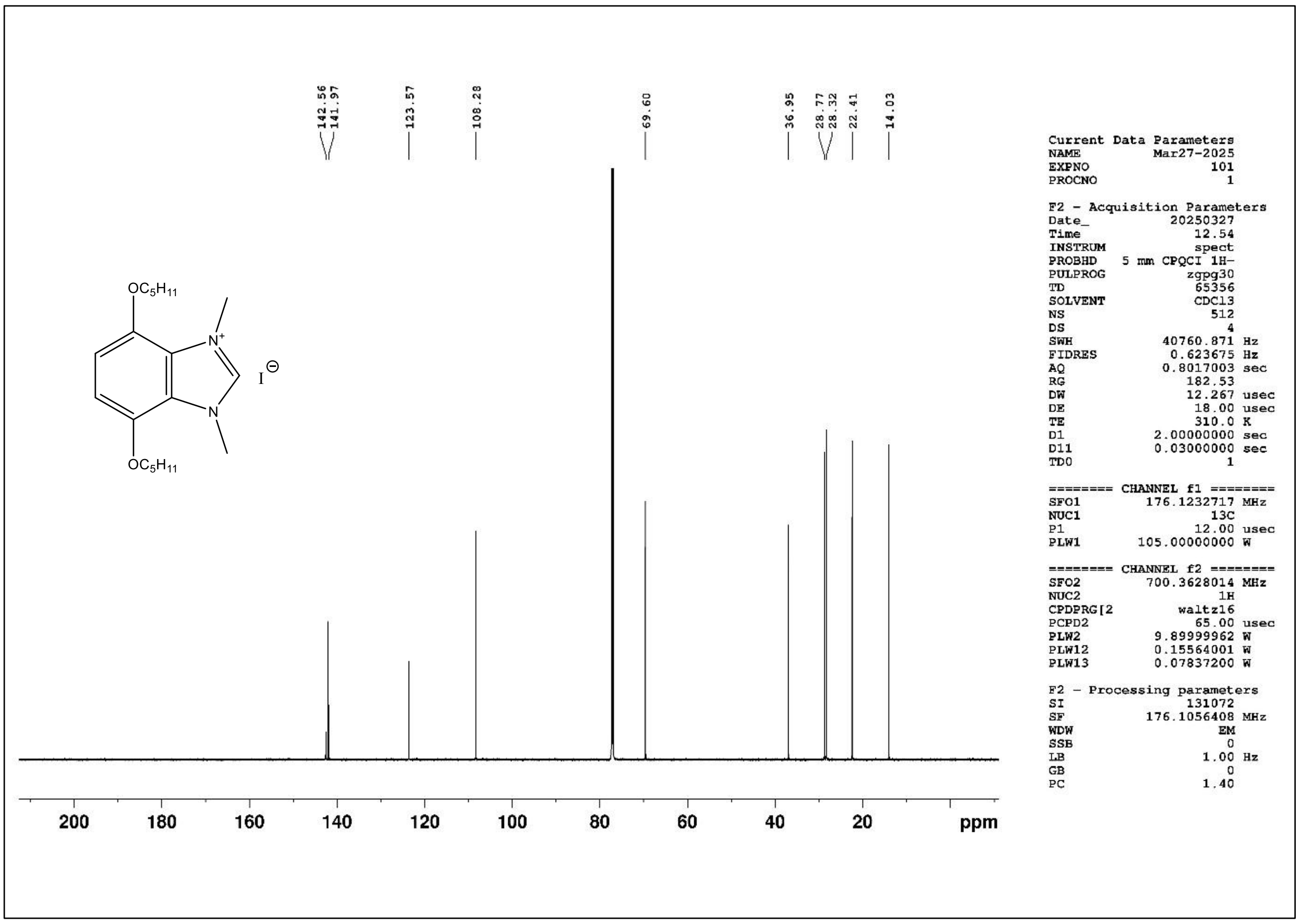

Figure S139. ^{13}C NMR of **L-Me$_2$Bi(5)I**.

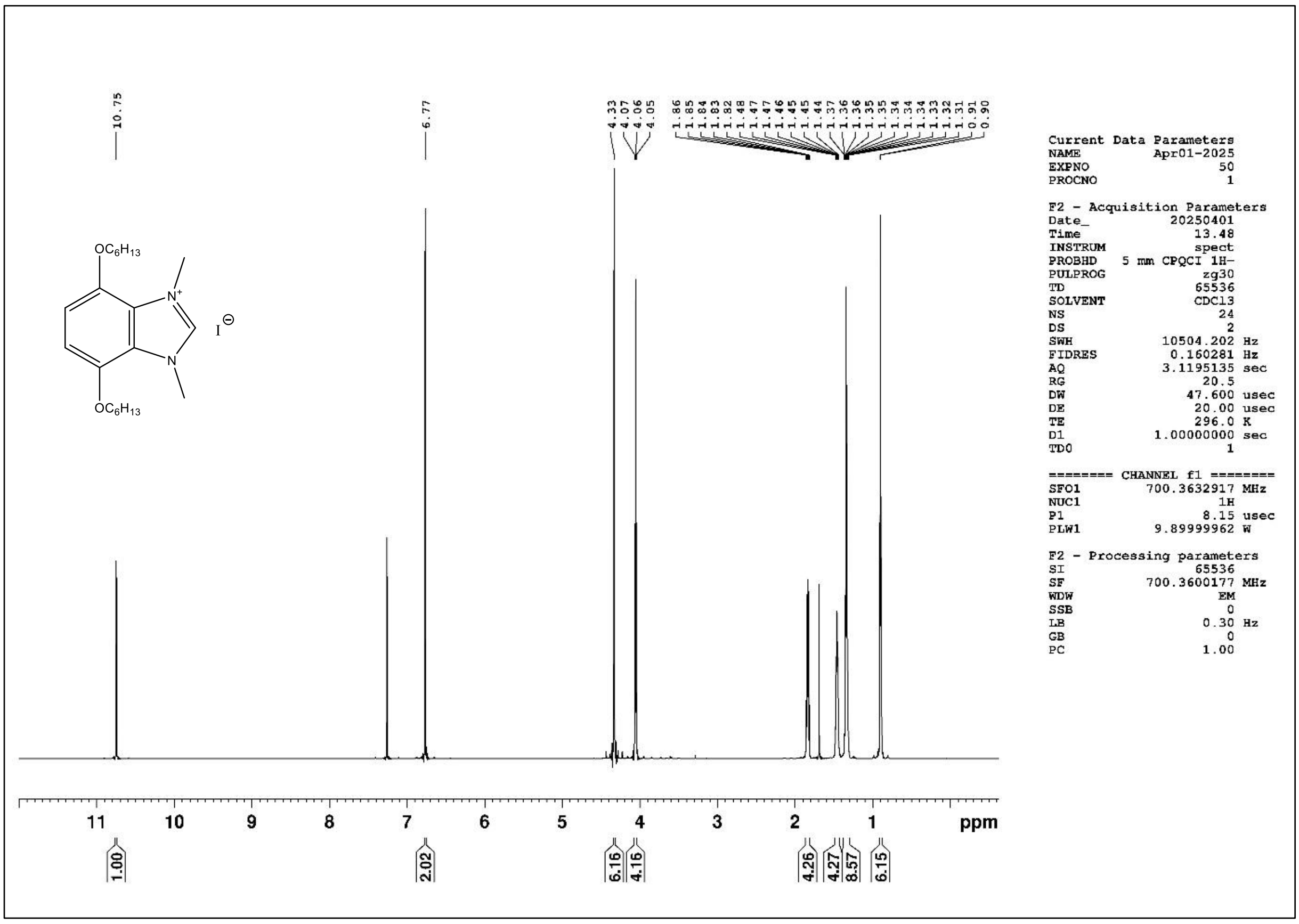

Figure S140. ^{1}H NMR of **L-Me$_2$Bi(6)I**.

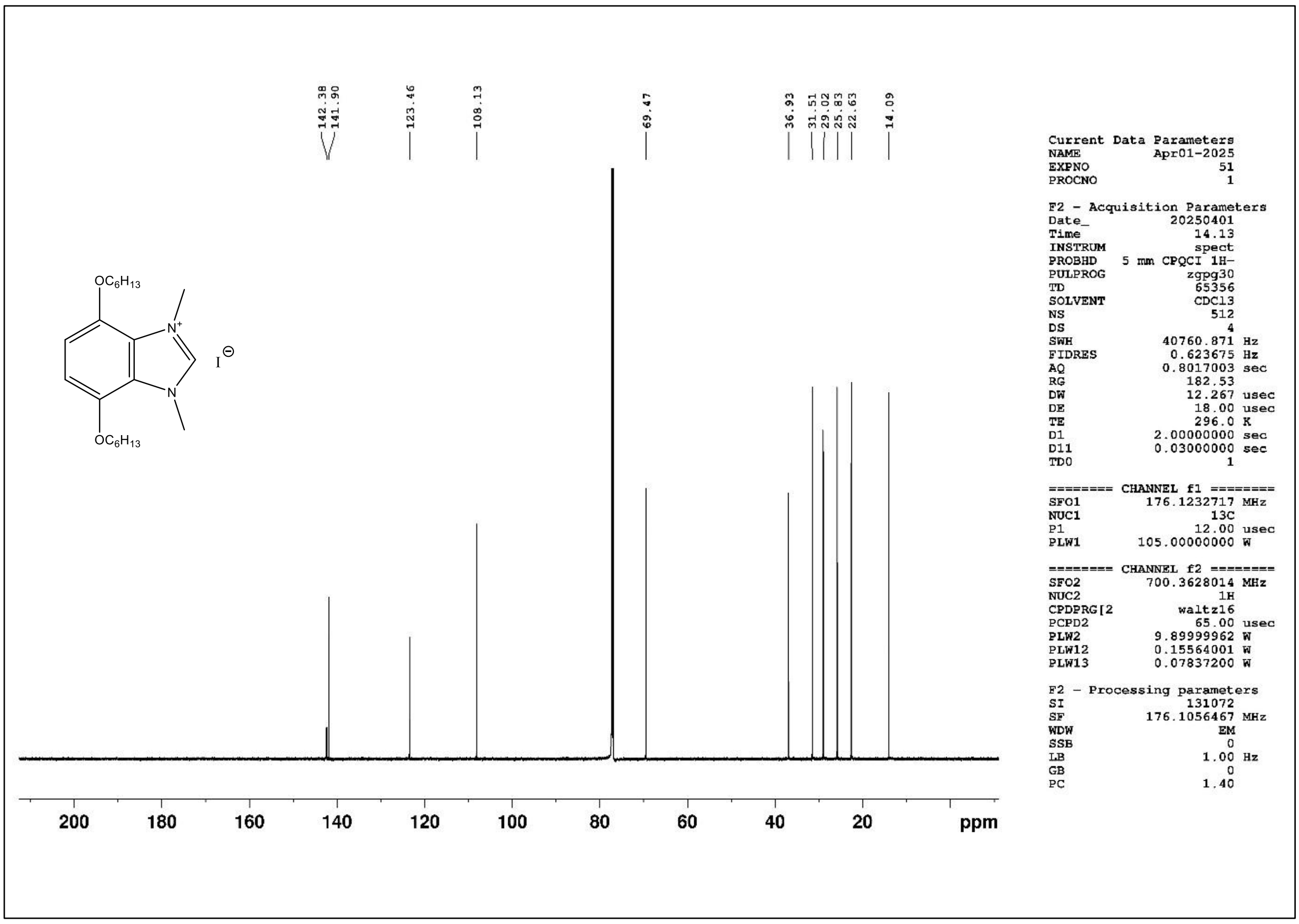

Figure S141. ^{13}C NMR of **L-Me$_2$Bi(6)I**.

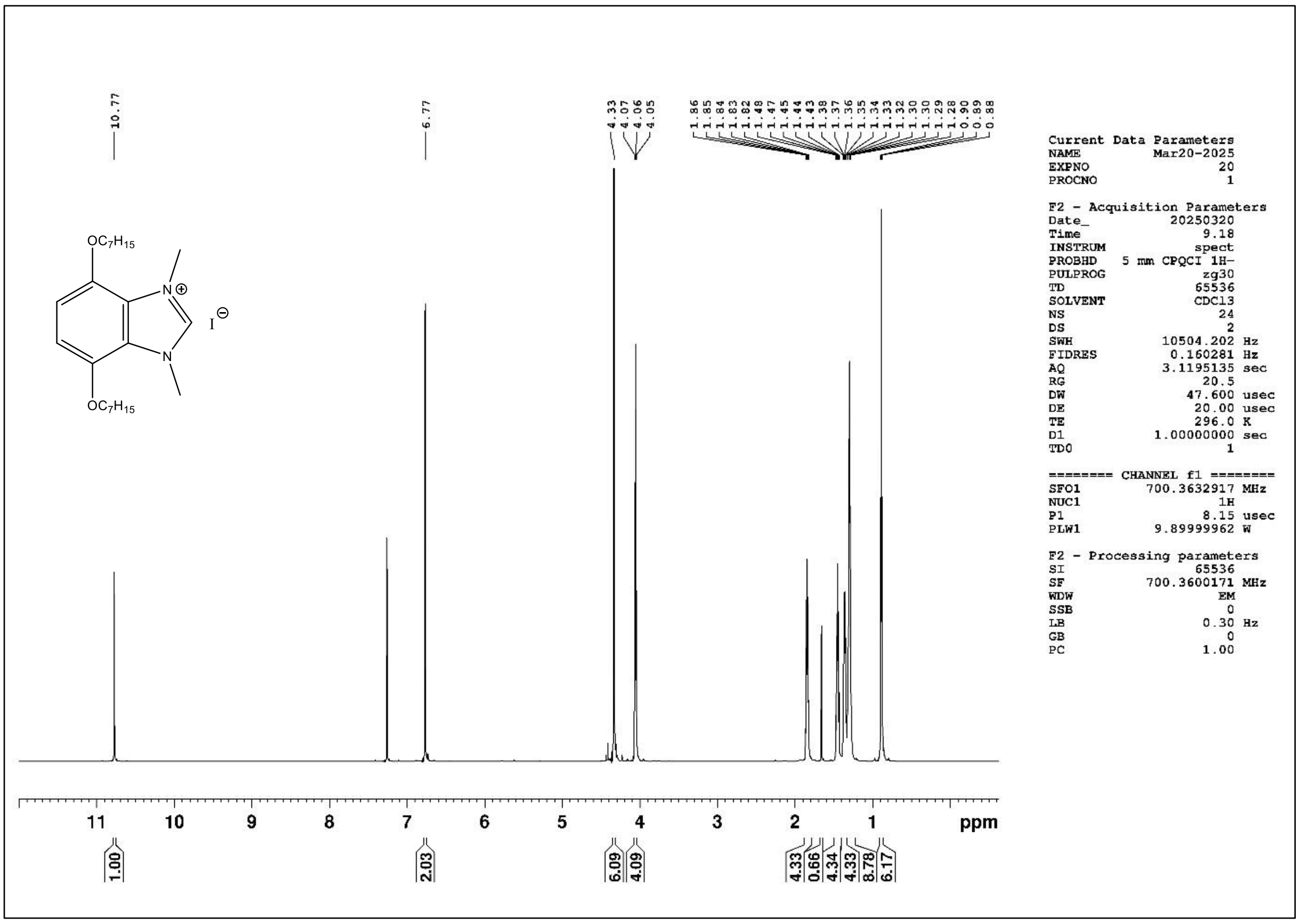

Figure S142. ^{1}H NMR of **L-Me$_2$Bi(7)I**.

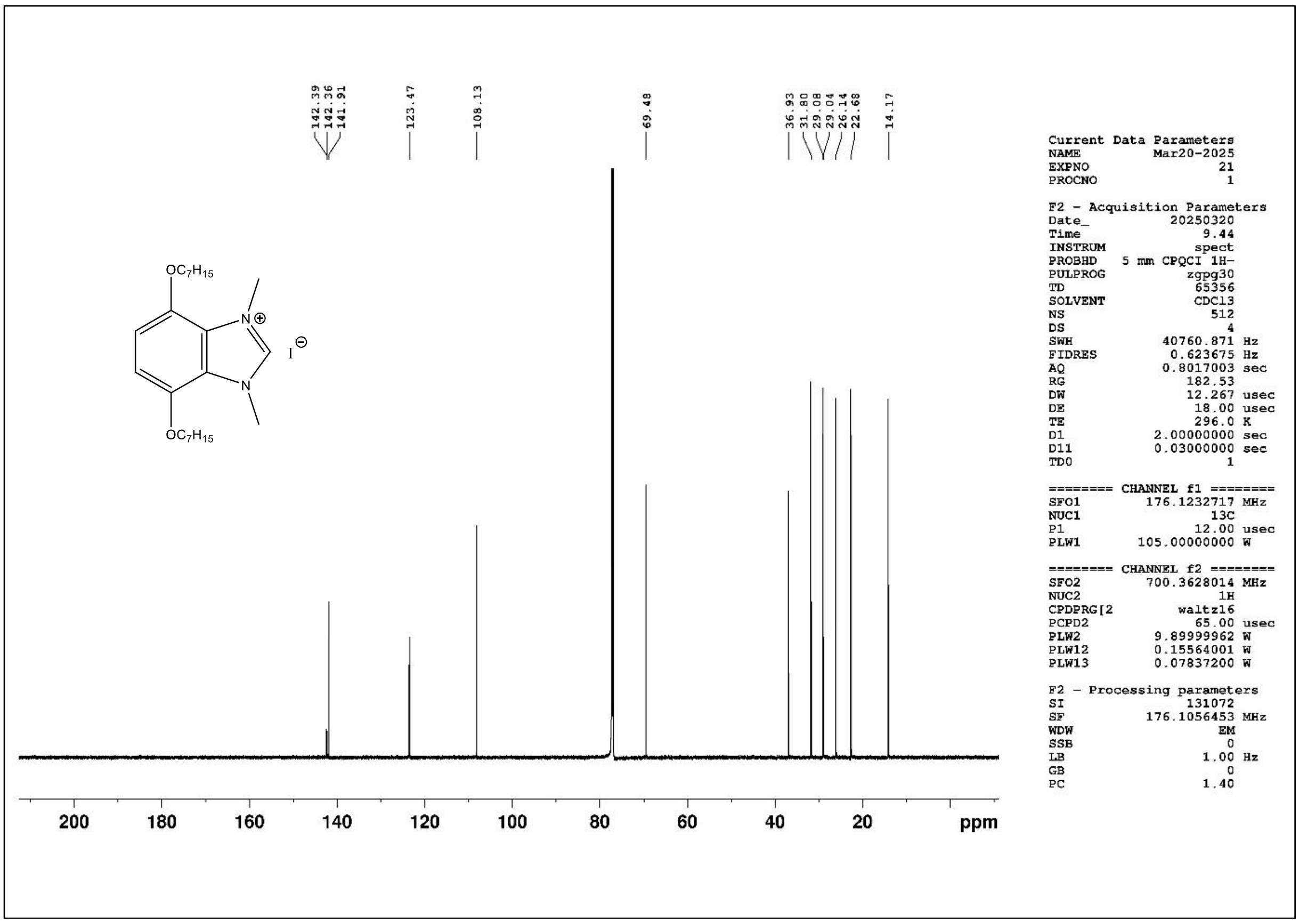

Figure S143. ^{13}C NMR of **L-Me$_2$Bi(7)I**.

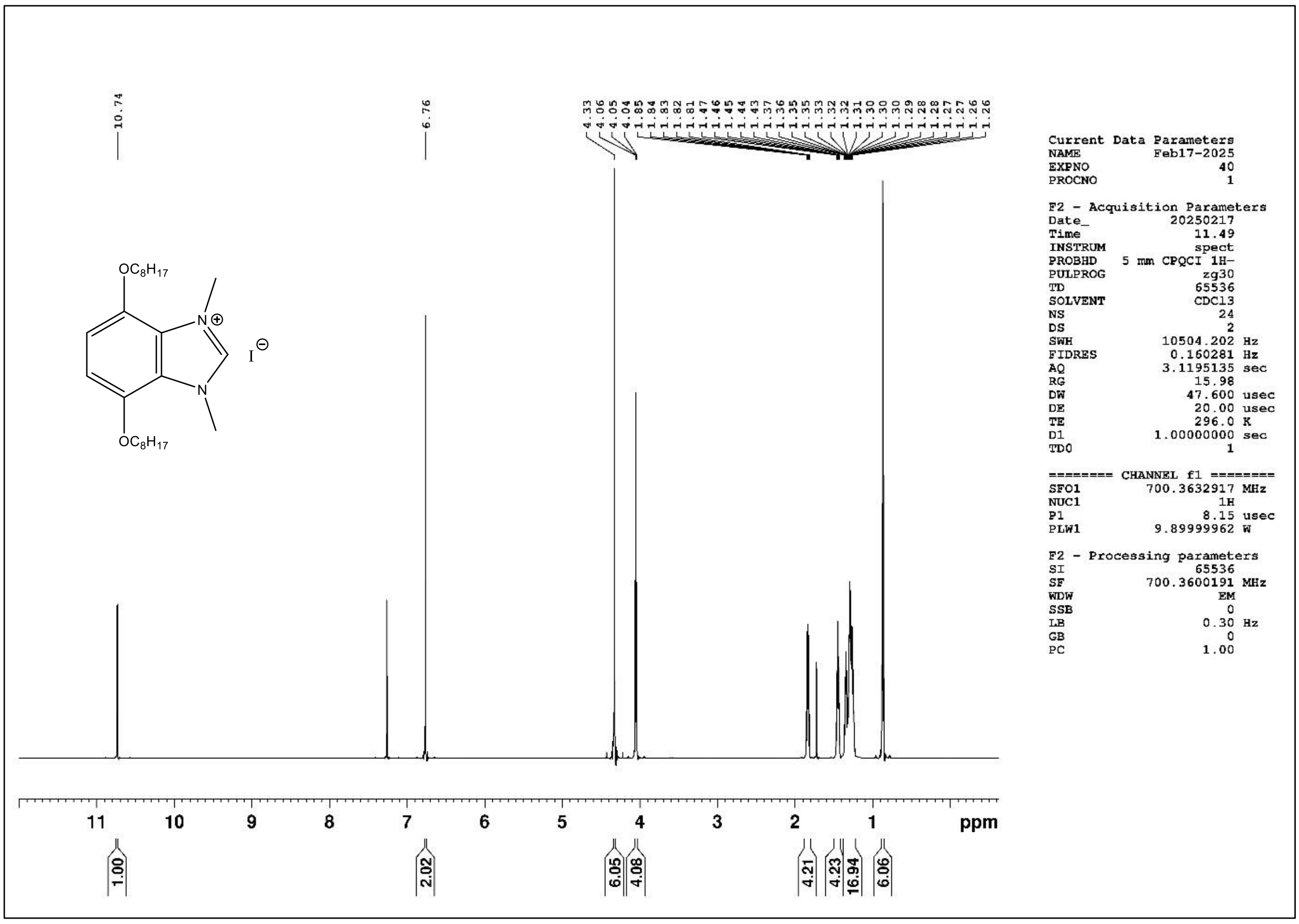

Figure S144. ^{1}H NMR of **L-Me$_2$Bi(8)I**.

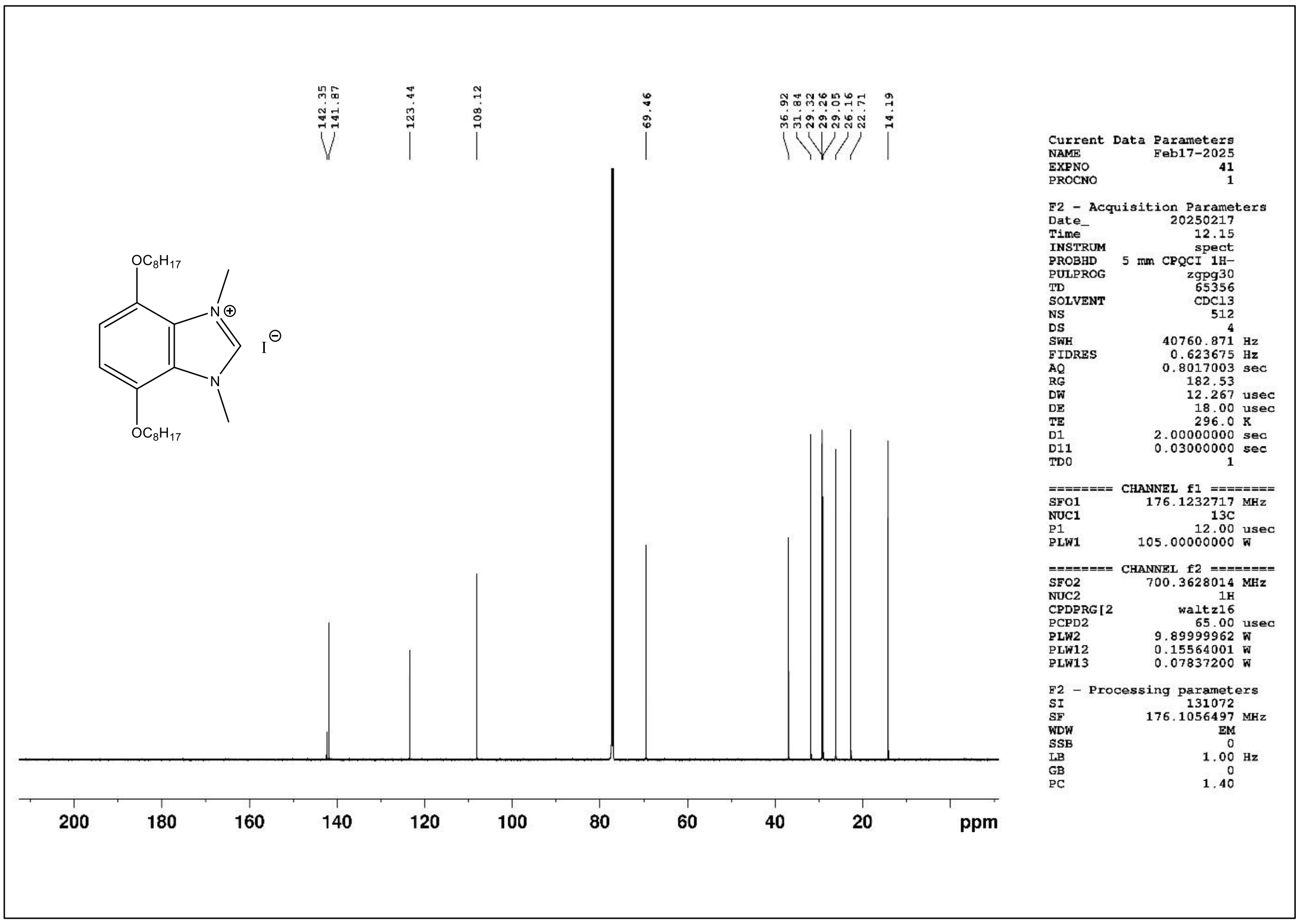

Figure S145. ^{13}C NMR of **L-Me$_2$Bi(8)I**.

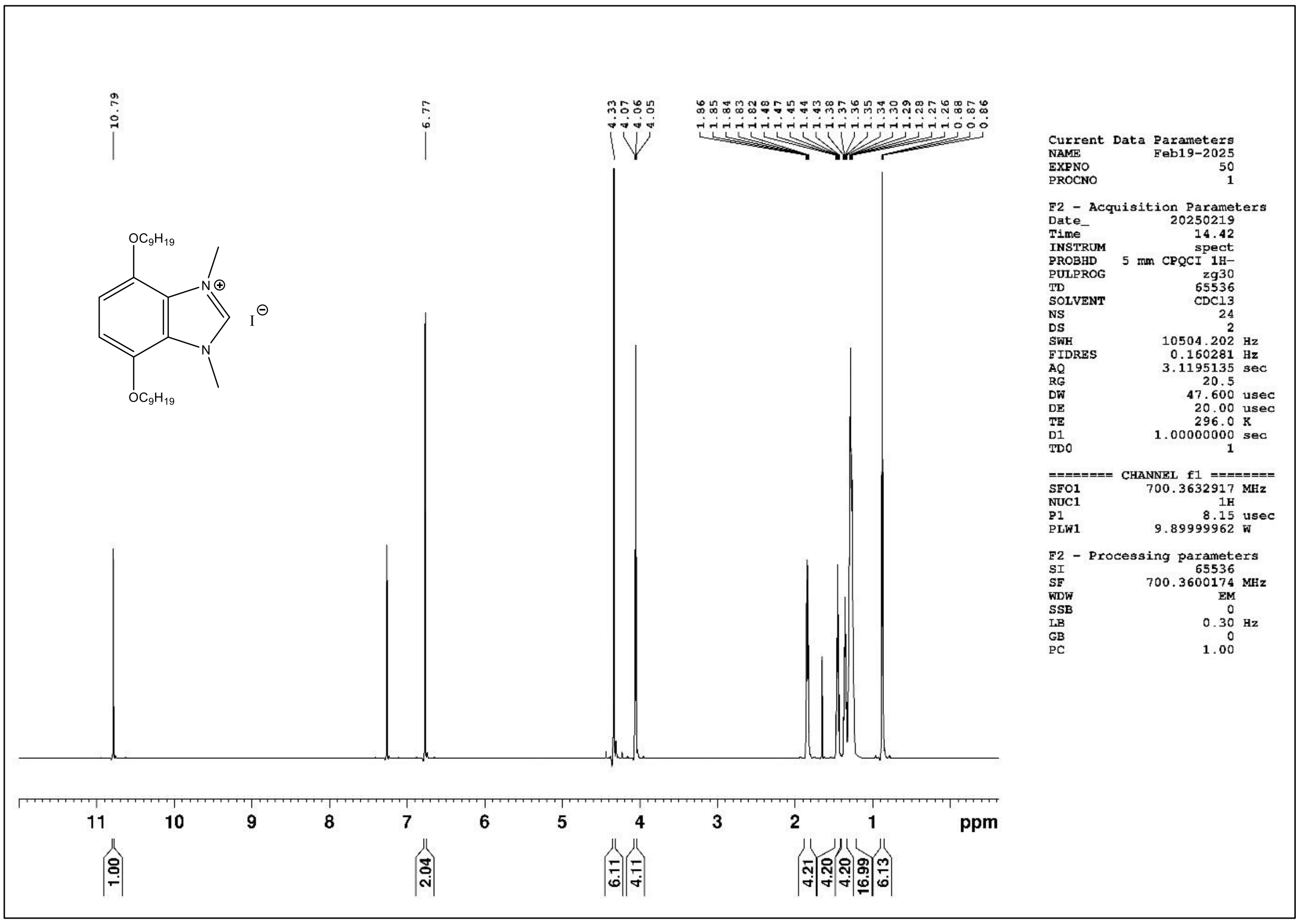

Figure S146. ^{1}H NMR of **L-Me$_2$Bi(9)I**.

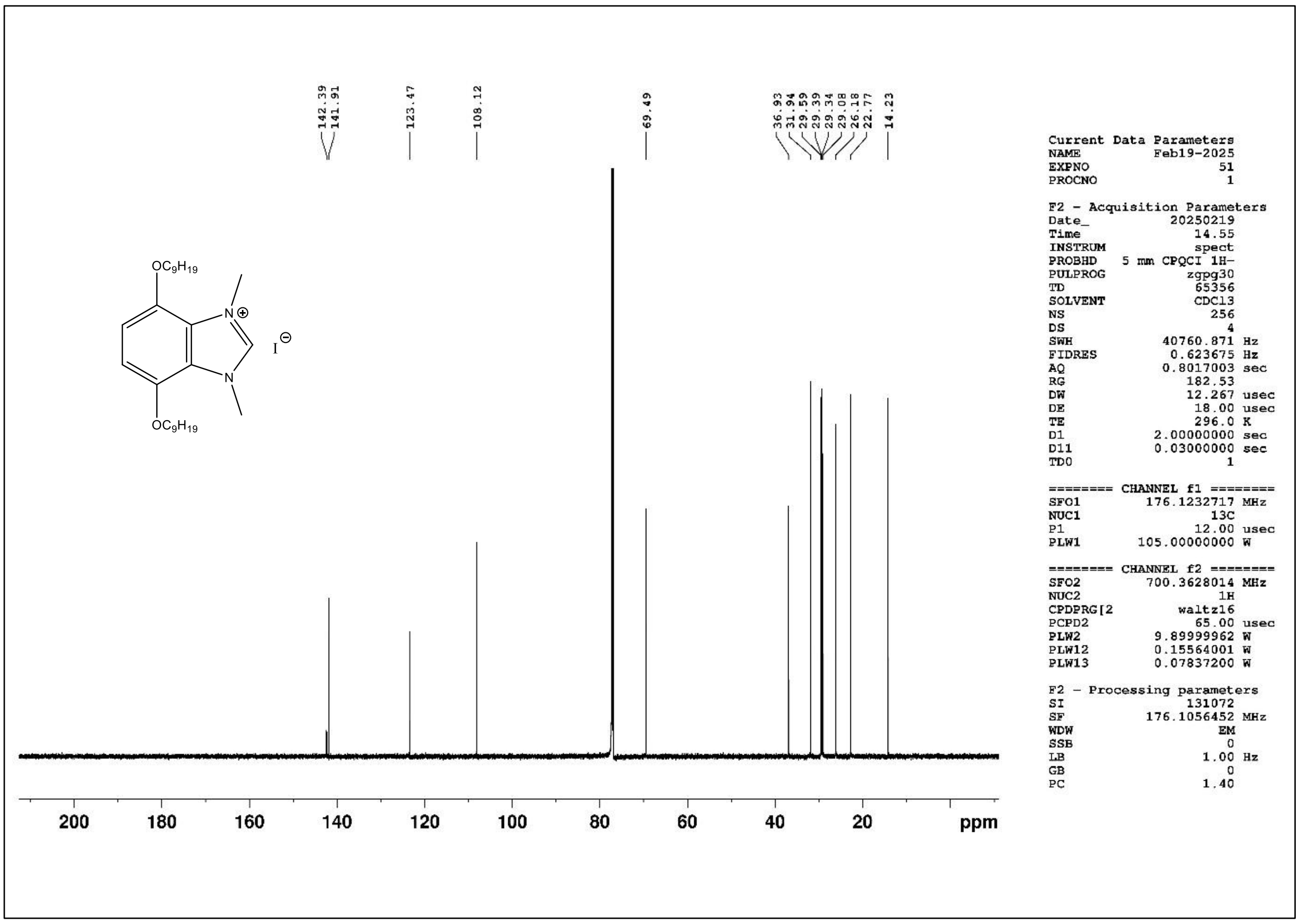

Figure S147. ^{13}C NMR of **L-Me$_2$Bi(9)I**.

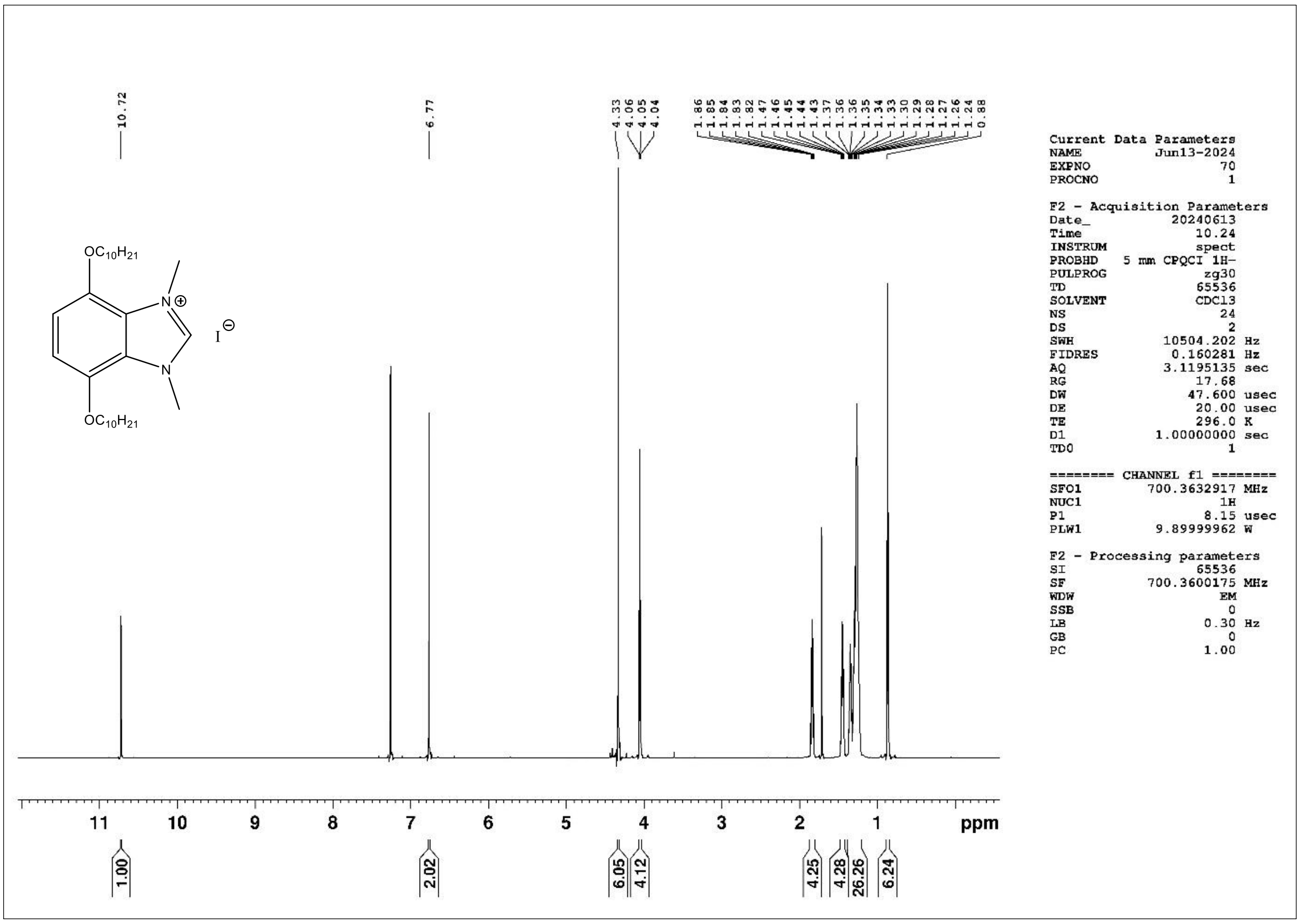

Figure S148. ^{1}H NMR of **L-Me$_2$Bi(10)I**.

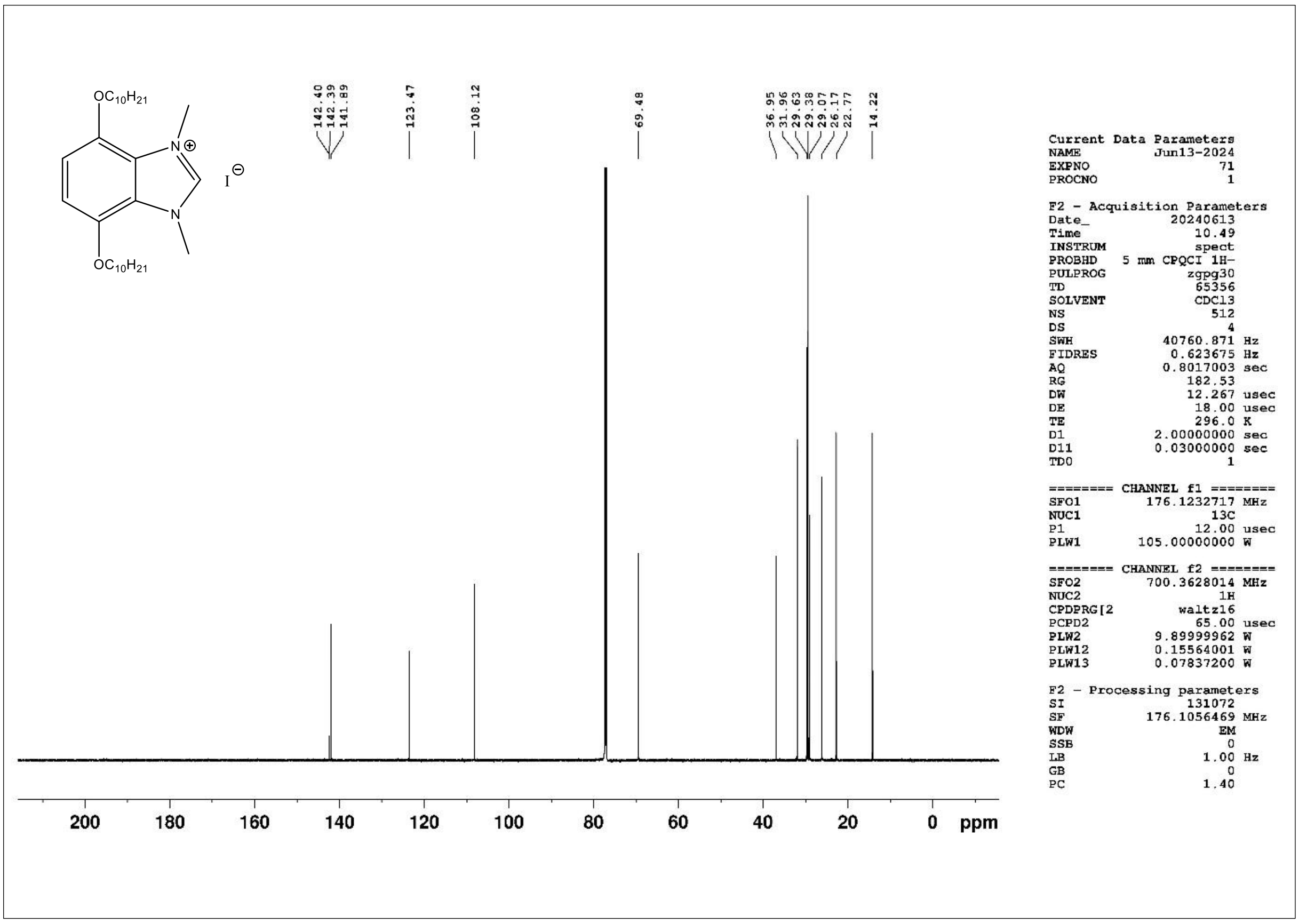

Figure S149. ^{13}C NMR of **L-Me$_2$Bi(10)I**.

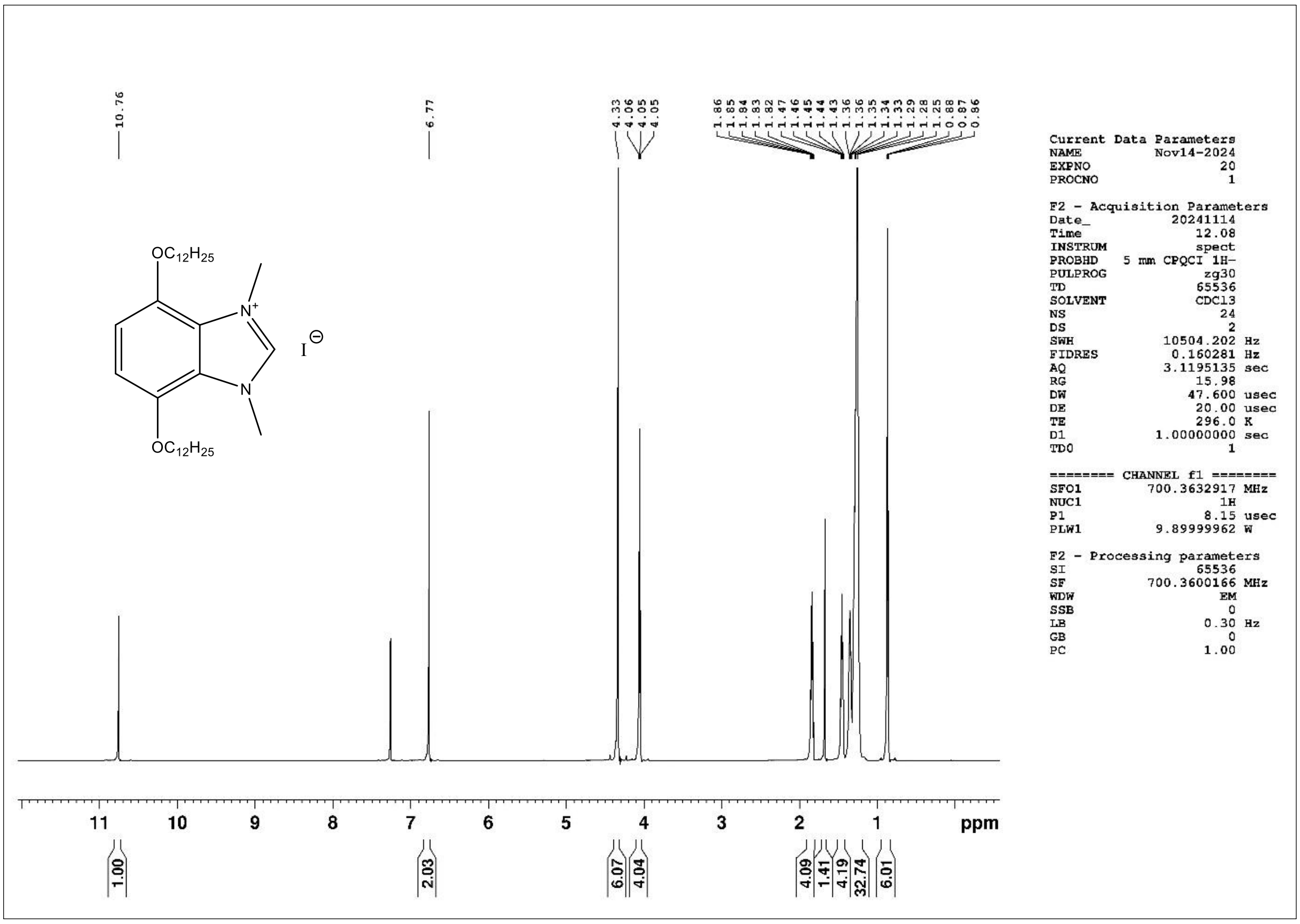

Figure S150. ^{1}H NMR of **L-Me$_2$Bi(12)I**.

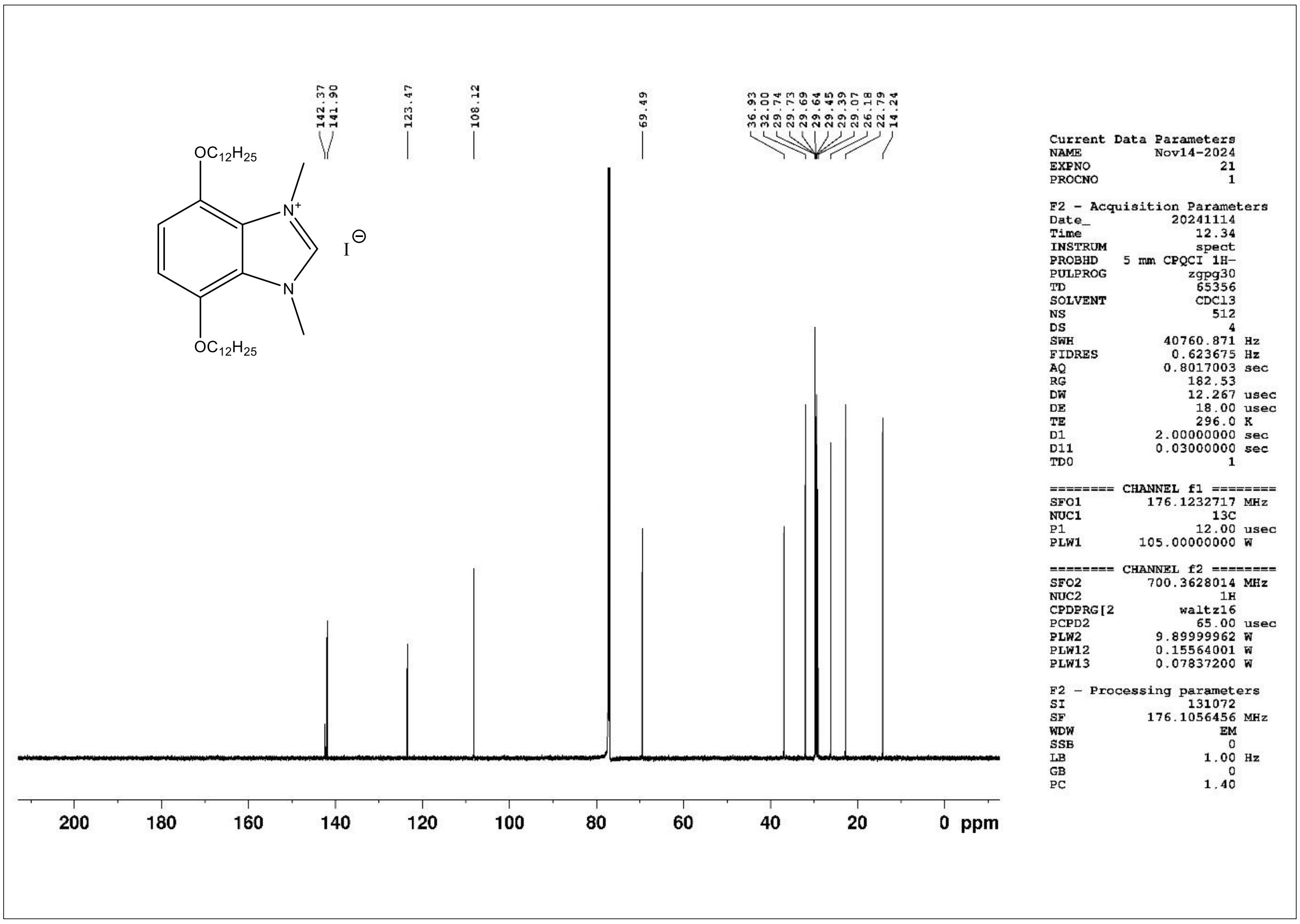

Figure S151. ^{13}C NMR of **L-Me$_2$Bi(12)I**.

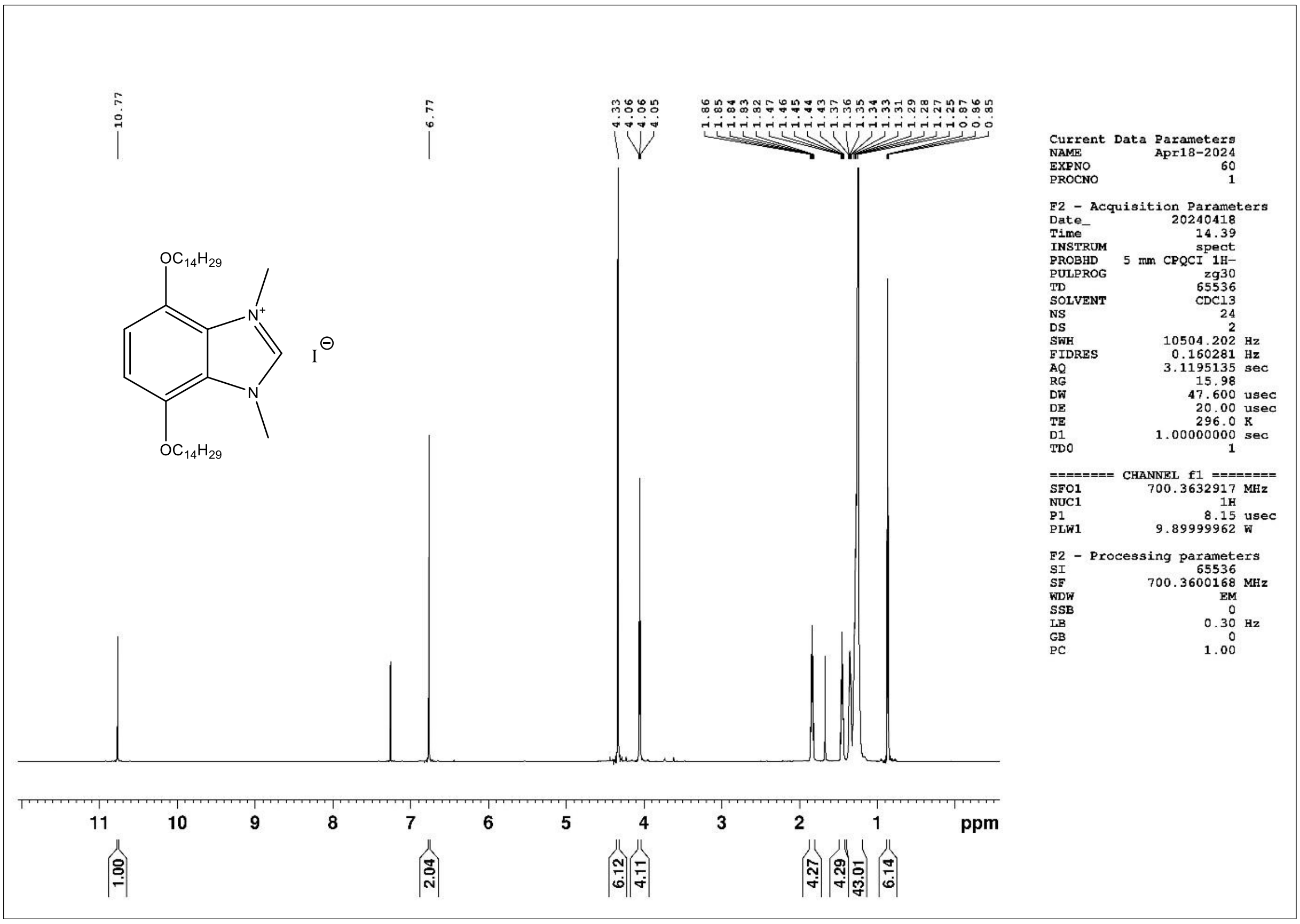

Figure S152. ^{1}H NMR of **L-Me$_2$Bi(14)I**.

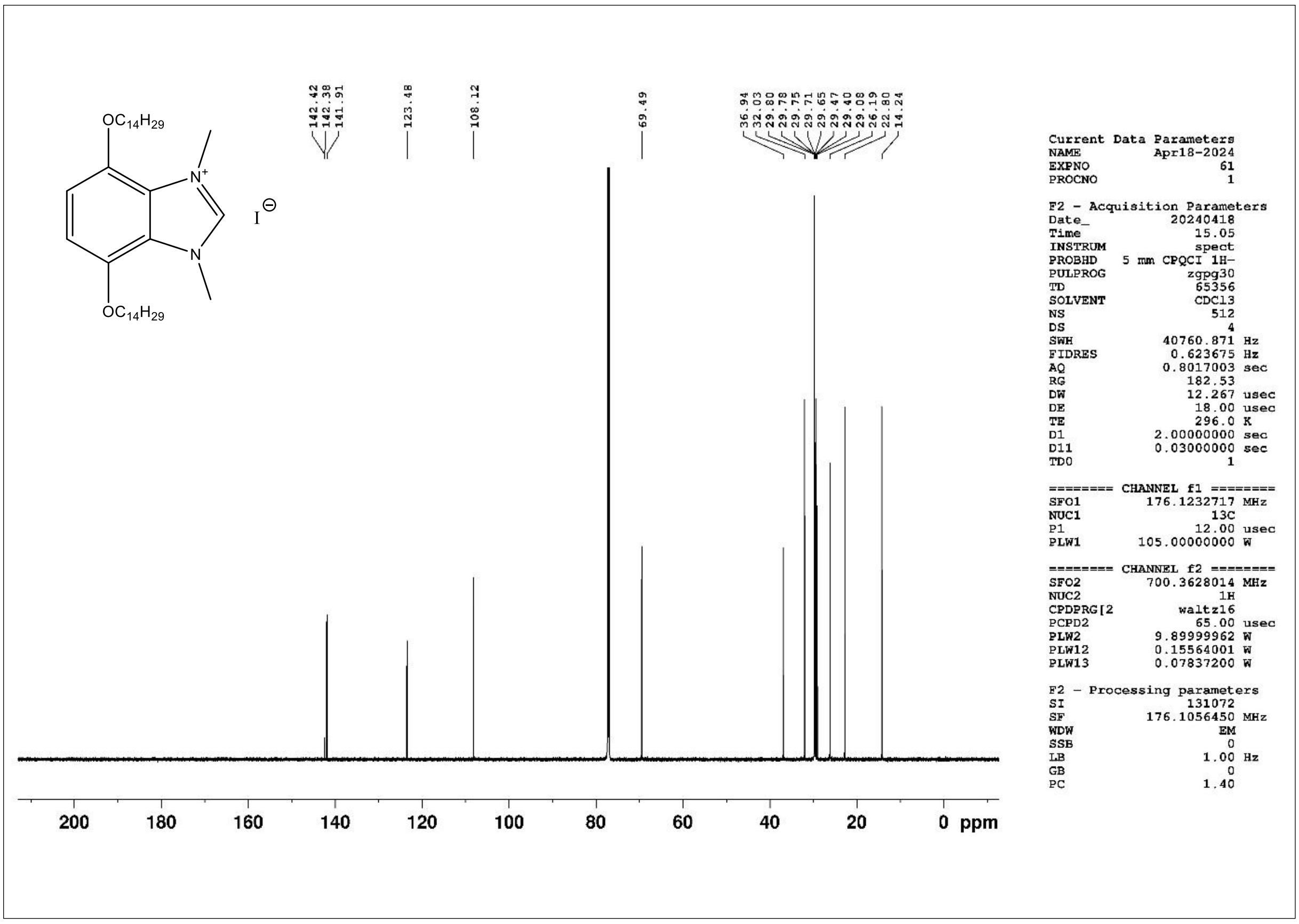

Figure S153. ^{13}C NMR of **L-Me$_2$Bi(14)I**.

5. SC-XRD

Suitable single crystals for crystal structure analysis of the compounds **L-Me$_2$Bi(9)I**, **U-Me$_2$Bi(5)I** and **U-Bi(6)** were obtained (Figure S154). Crystals of **L-Bi(12)** could likewise be obtained. However, the data quality of this measurement was only sufficient to provide an indication of the overall structural features.

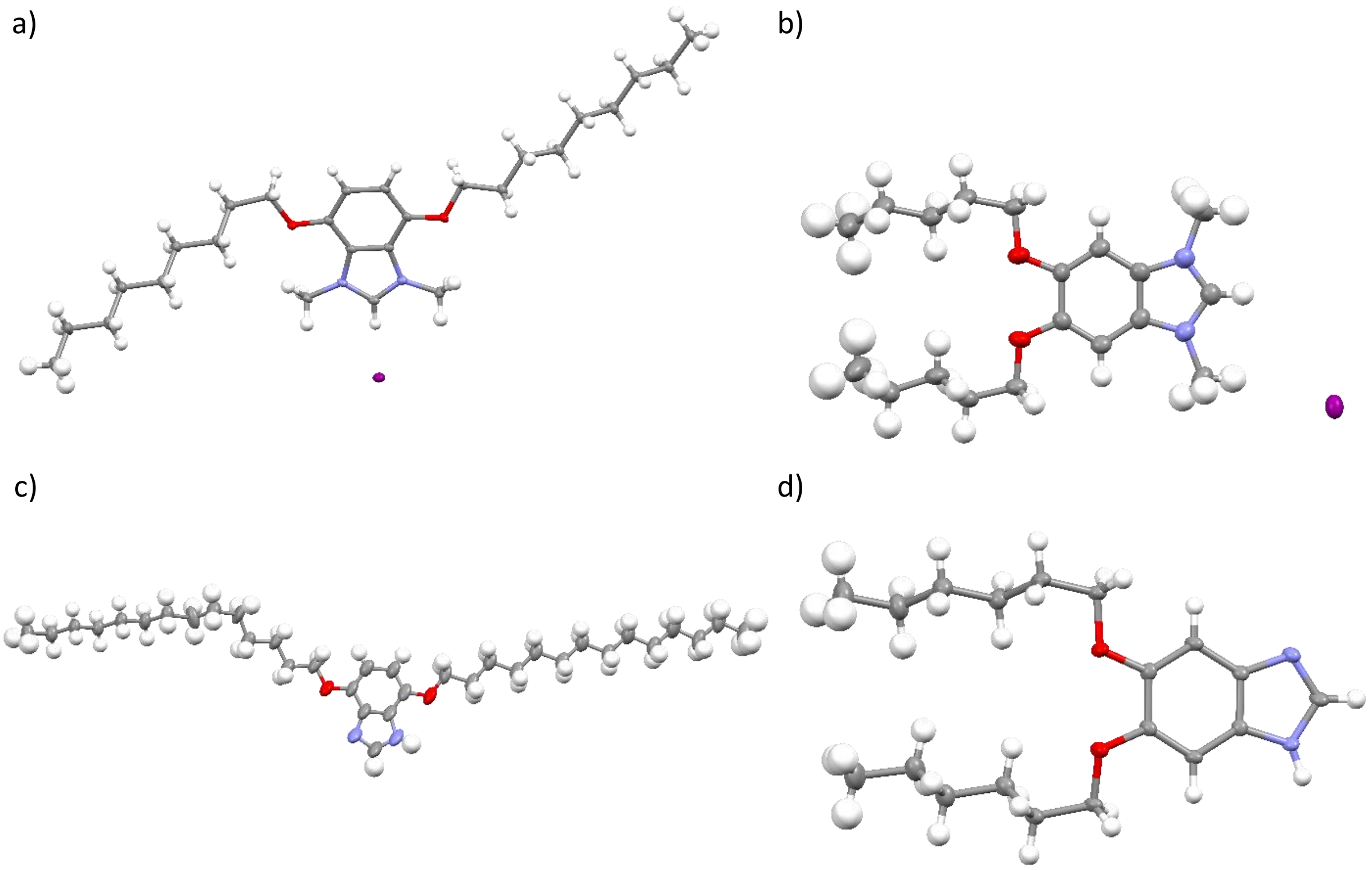

Figure S154. Illustration of the solid state structures of a) **L-Me$_2$Bi(9)I**, b) **U-Me$_2$Bi(5)I** (only one possible position of the disordered iodide), c) **L-Bi(14)** and d) **U-Bi(6)**. Displacement ellipsoids at 50% probability level; colour code: O red, C grey, N blue; I purple; H white.

The solid-state structures of the neutral compound **U-Bi(6)** are constructed from corrugated layers of molecules interconnected by hydrogen bonding (Figure S155). The distance between donor N and acceptor N is 2.83 Å. The benzimidazole units forming these hydrogen bonds are oriented at an angle of 43.3° to each other, creating a slightly staircase-like pattern. Within these layers, adjacent molecules are alternately rotated by 180°, resulting in a head-to-head arrangement of the benzimidazole units. The co-aligned benzimidazole units within each layer are oriented parallel to one another. Figure S156 shows that these layers pack in a nearly perfect lamellar organization in the case of **U-Bi(6)**.

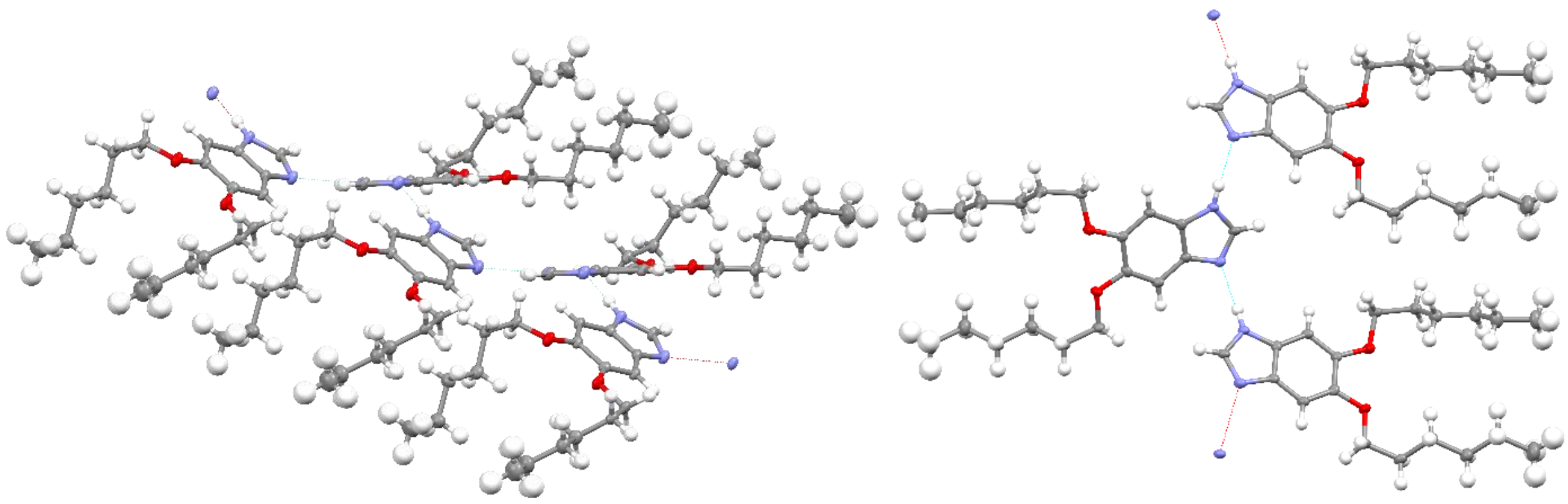

Figure S155. Illustration of the hydrogen bonds (dashed bonds) in a layer of **U-Bi(6)**. Displacement ellipsoids at 50% probability level; hydrogen atoms omitted; color code: O red, C grey, N blue; H white.

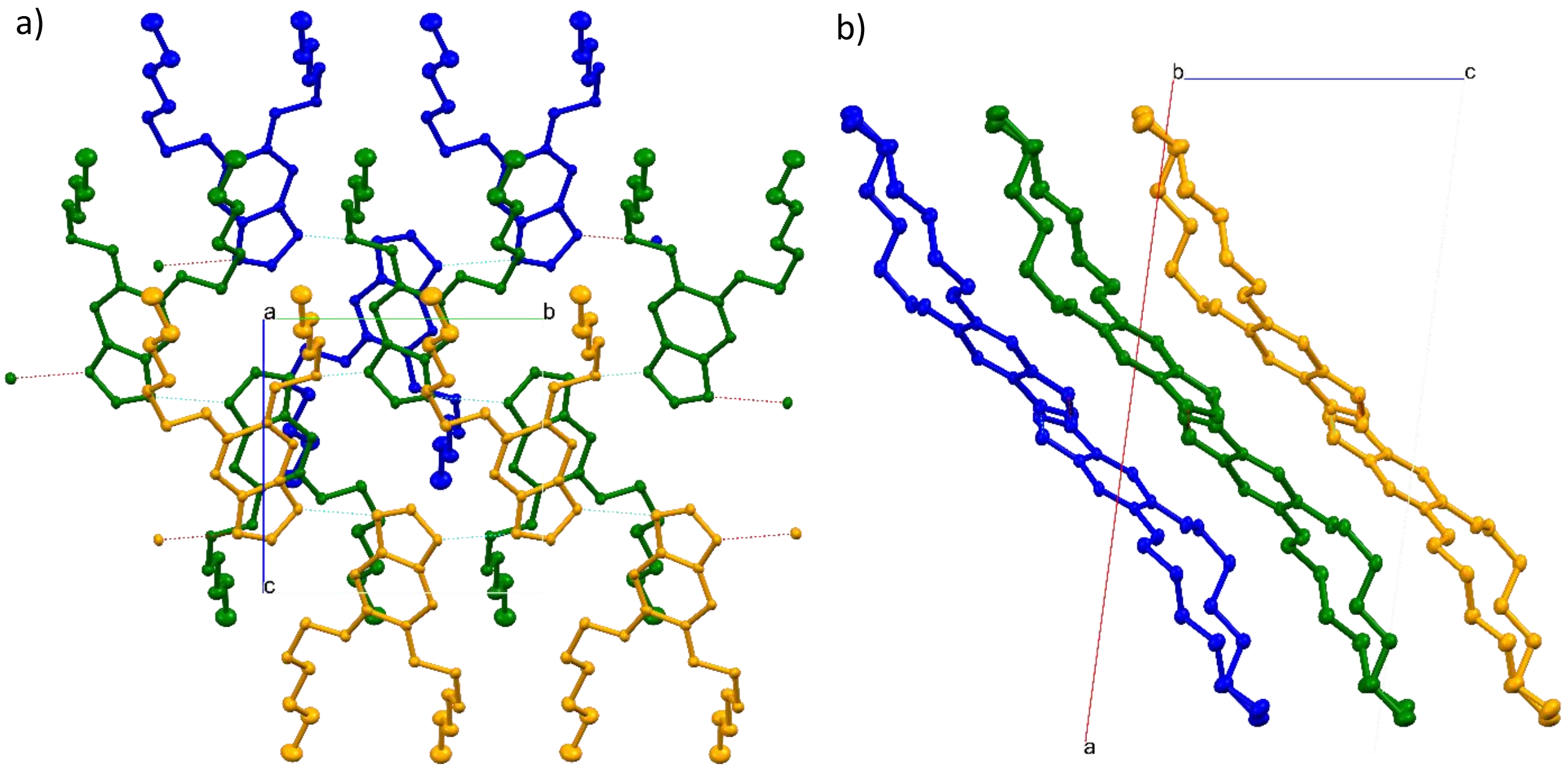

Figure S156. Illustration of the packing of **U-Bi(6)** a) view along the *a*-axis and b) view along the *b*-axis. Molecules within the same layer that are connected by hydrogen bonds are shown in the same colour for clarity. Displacement ellipsoids at 50% probability level.

a)
a)

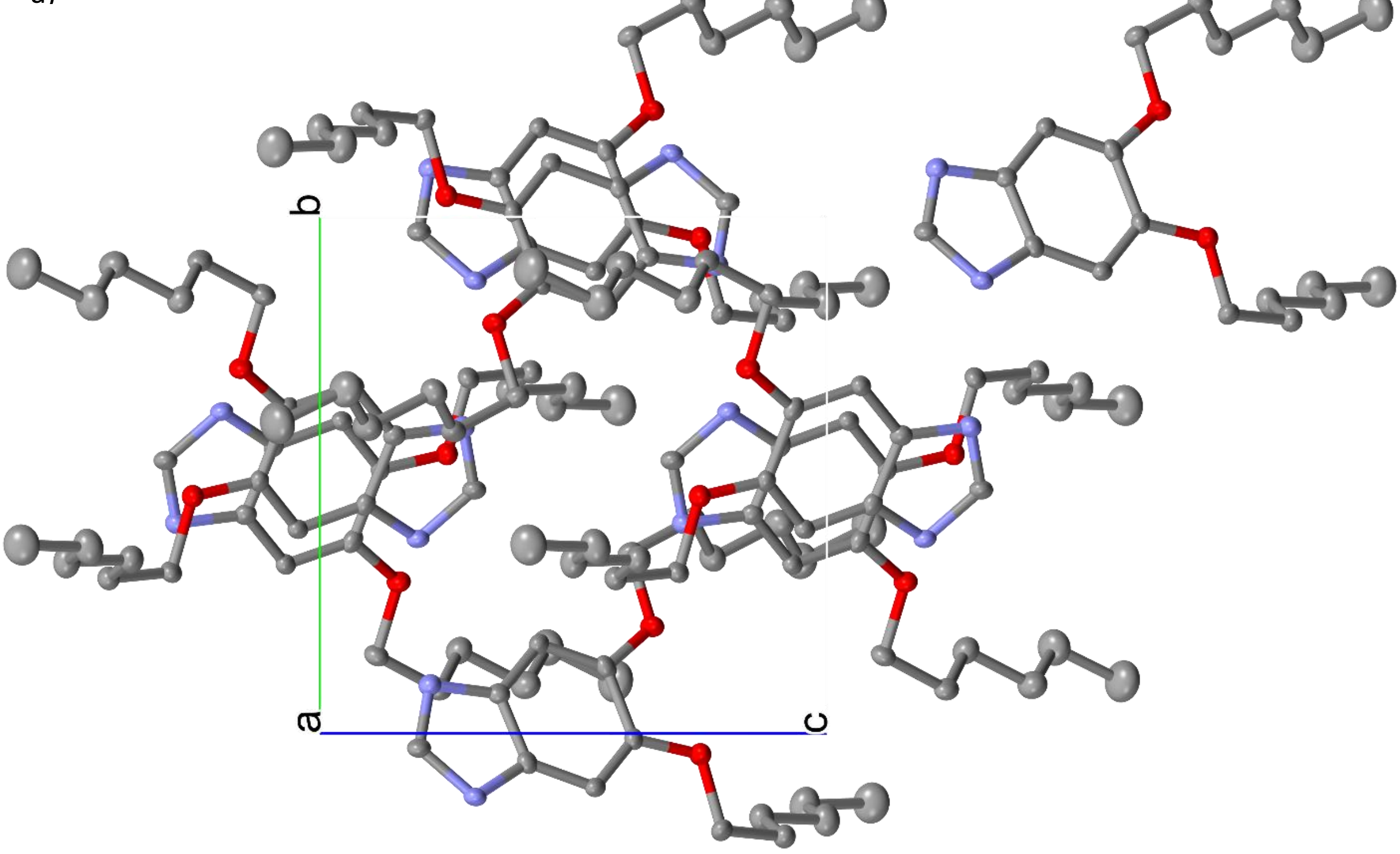

b)
b)

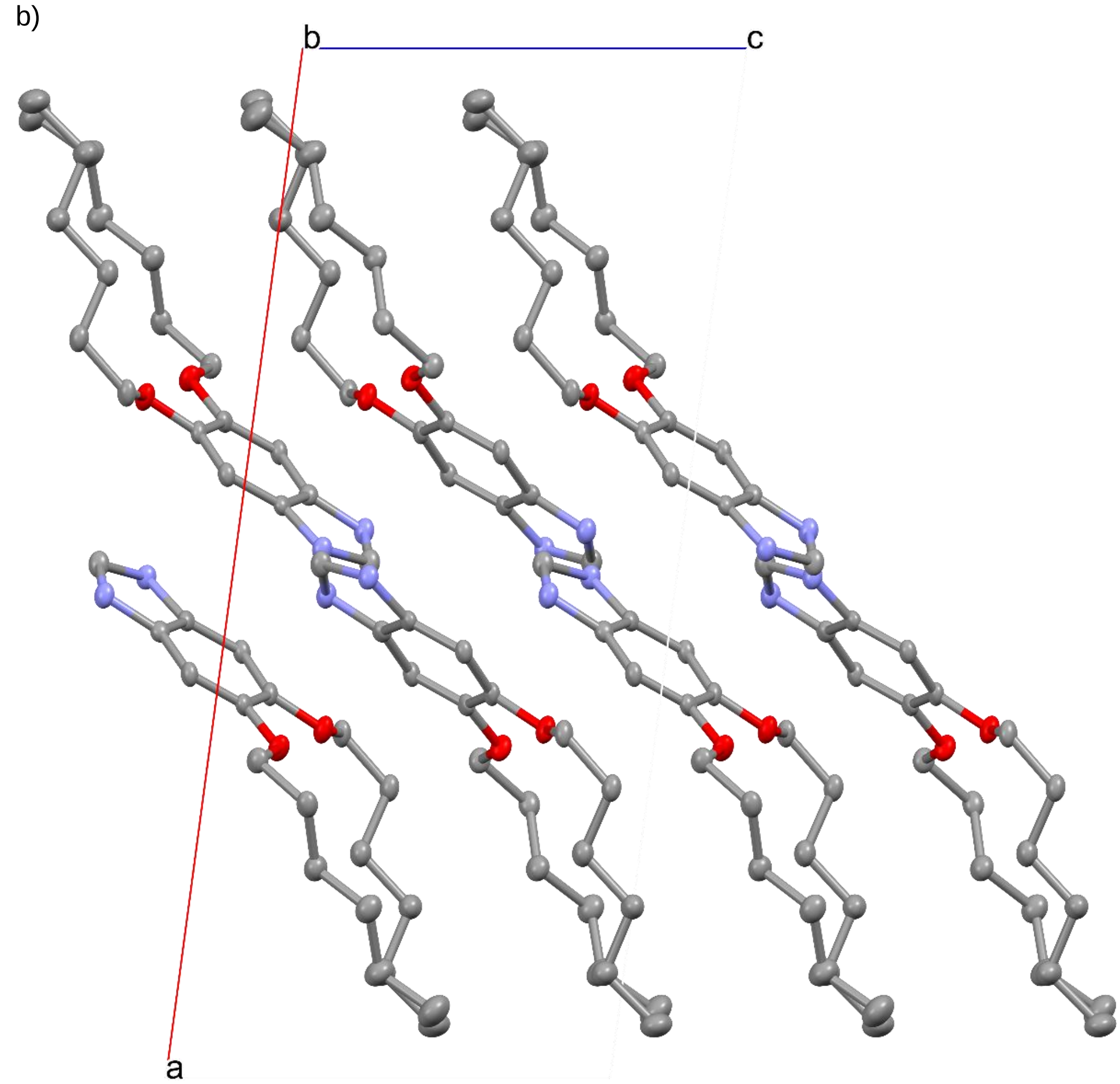

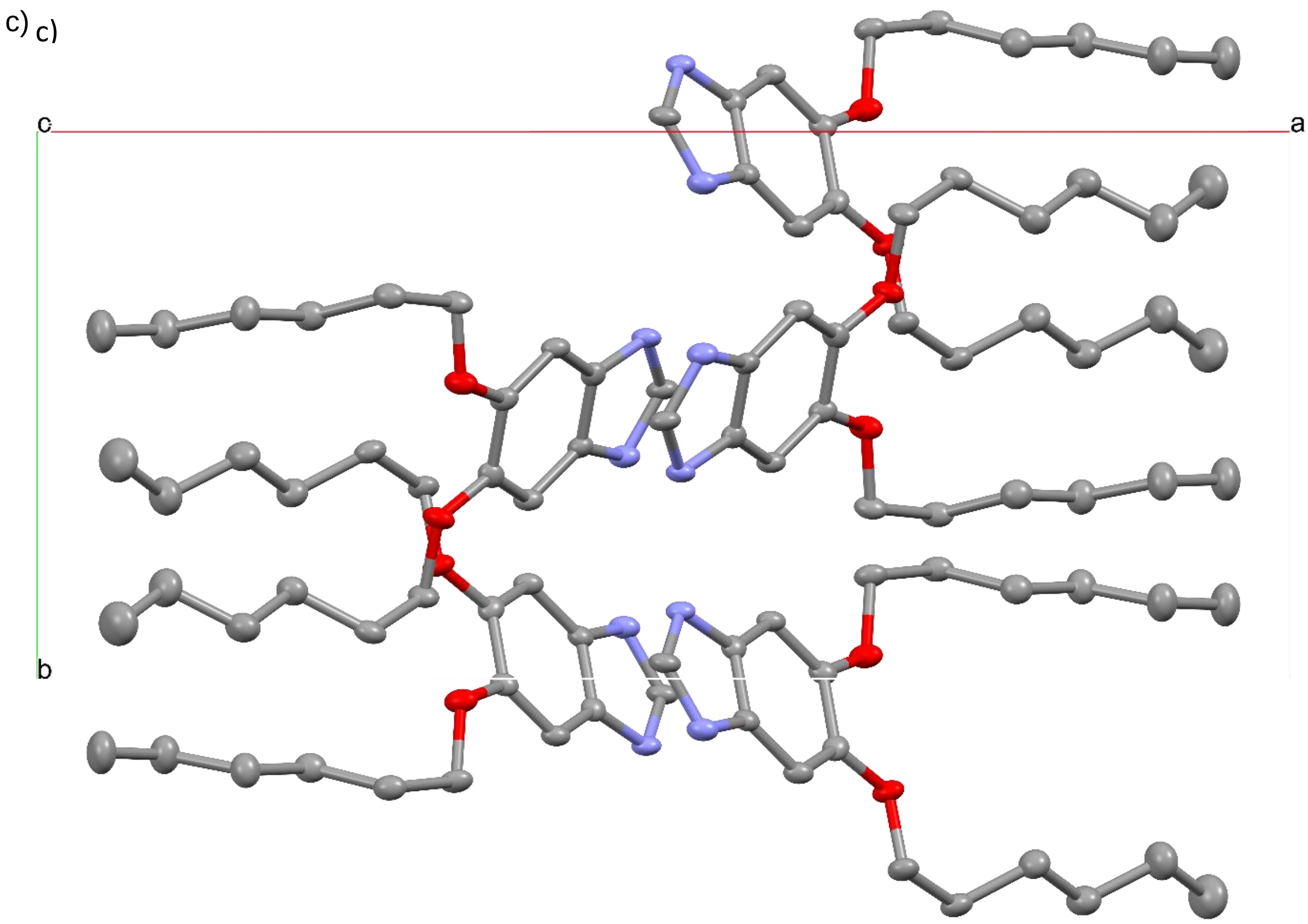

Figure S157. Illustration of the packing of **U-Bi(6)** in the unit cell. View along the a) *a*-axis b) *b*-axis and c) *c*-axis. Displacement ellipsoids at 50% probability level; hydrogen atoms omitted; colour code: O red, C grey, N blue.

Due to the low resolution at high angles and the severe disorder within the crystal, the observations described below should be regarded only as an indication of the solid state structure.

Figure 159c revealed a butterfly-shaped conformation of the **L-Bi(14)**. In this neutral compound, layers of molecules connected by hydrogen bonds are also formed. However, owing to the non-coplanar orientation of the side chains relative to the benzimidazole ring, these layers can no longer arrange in a perfectly lamellar structure, resulting instead in the formation of a more tube-like architecture.

a)

b)

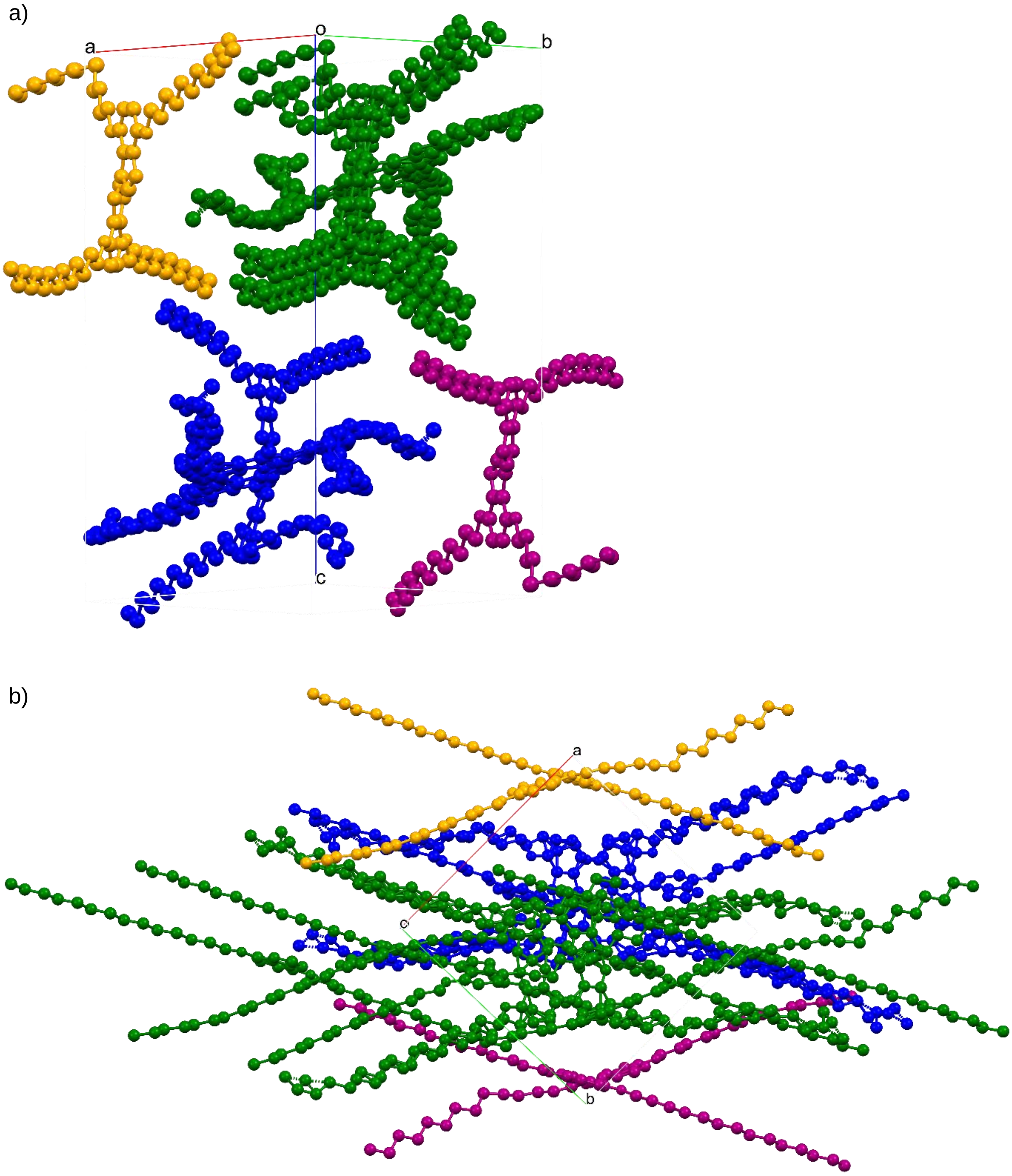

Figure S158. Illustration of the packing of **L-Bi(14)** a) view along the layer of hydrogen bonded molecules b) view along the *c*-axis. Molecules within the same layer that are connected by hydrogen bonds are shown in the same colour for clarity. Originally coloured packing figures are shown below.

a)

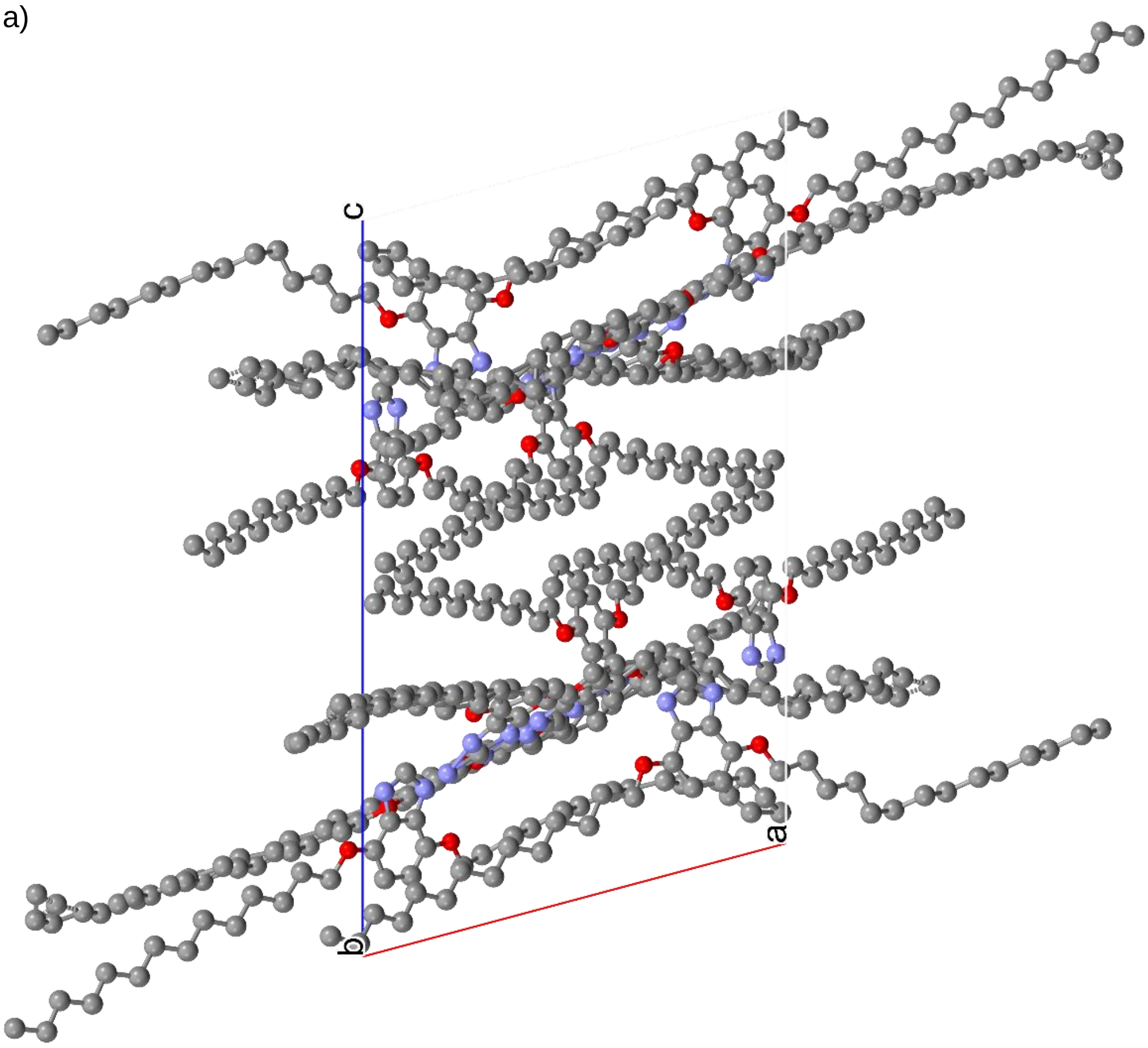

b)

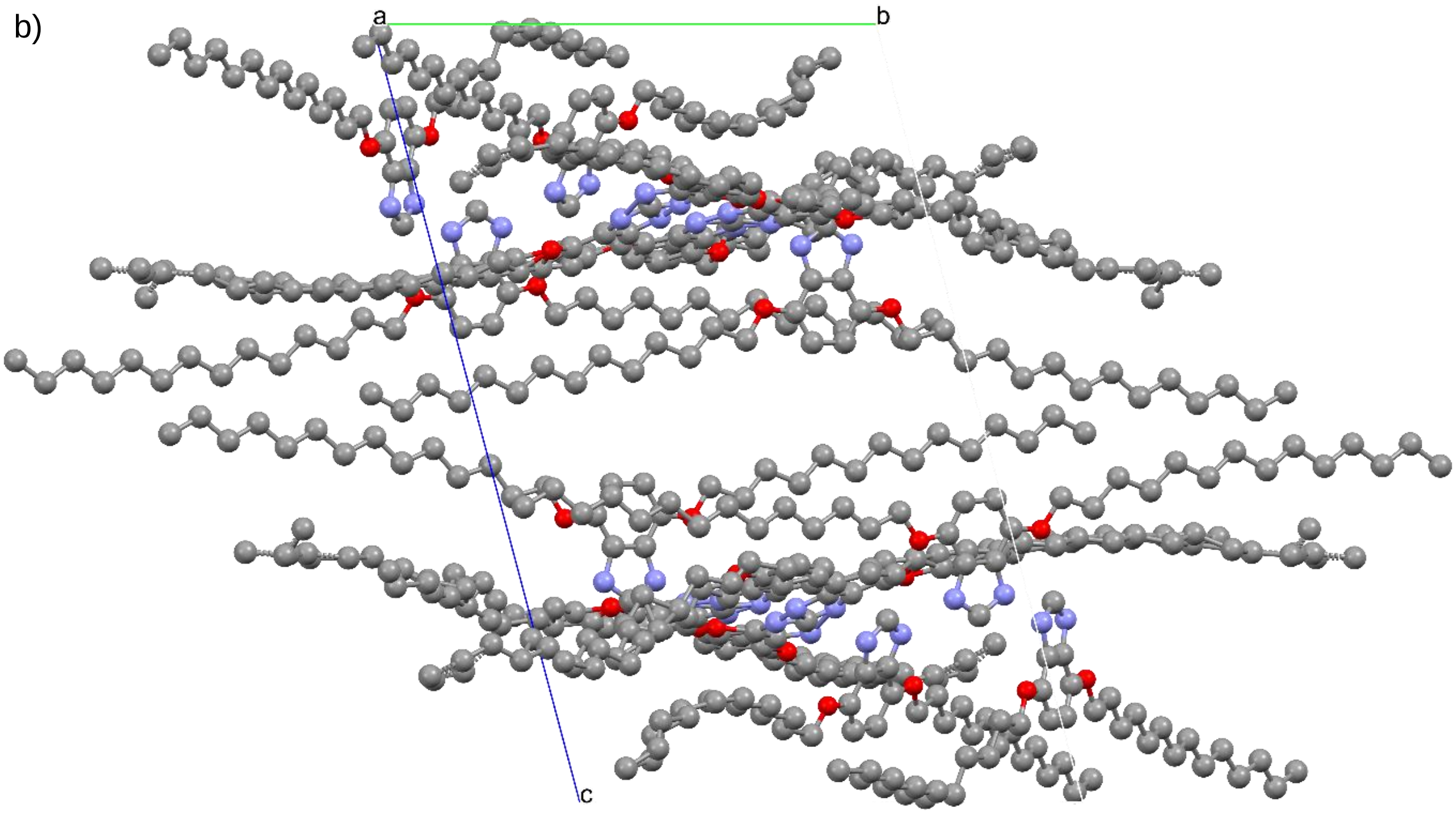

c)

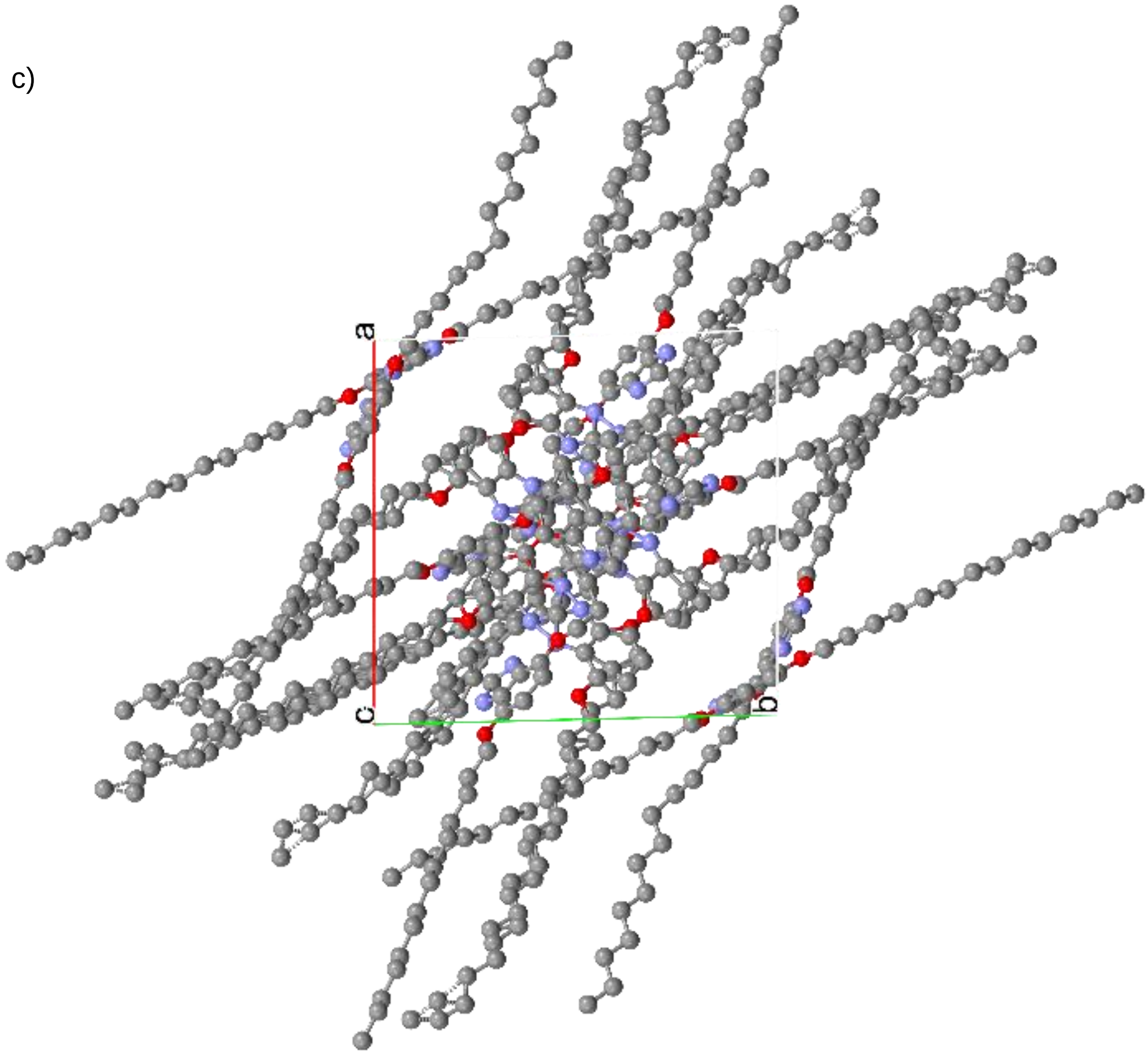

Figure S159. Illustration of the packing of **L-Bi(14)** in the unit cell. View along the a) *a*-axis b) *b*-axis and c) *c*-axis. For better visualization only shown as ball and stick; hydrogen atoms omitted; colour code: O red, C grey, N blue.

In contrast to the neutral benzimidazoles, which form a lamellar structure in the solid state through hydrogen bonding between the imine and amine nitrogens, this is no longer possible in the ionic form. In this case, the former donor-acceptor positions are occupied by methyl groups.

Inspection of the ionic structures of **L-Me$_2$Bi(9)I** and **U-Me$_2$Bi(5)I** reveals that in both, the linear and U-shaped derivatives, arrange in layered assemblies. When viewed along the *b*- and *c*-axis, respectively (Figure S160), the benzimidazole units are aligned parallel to each other but exhibit a slight lateral offset with a distance of 3.42 Å between them. In the **L-Me$_2$Bi(9)I** derivative, the benzimidazole moieties are rotated by 180° from one layer to the next, whereas in the **U-Me$_2$Bi(5)I** structure they maintain the same orientation throughout.

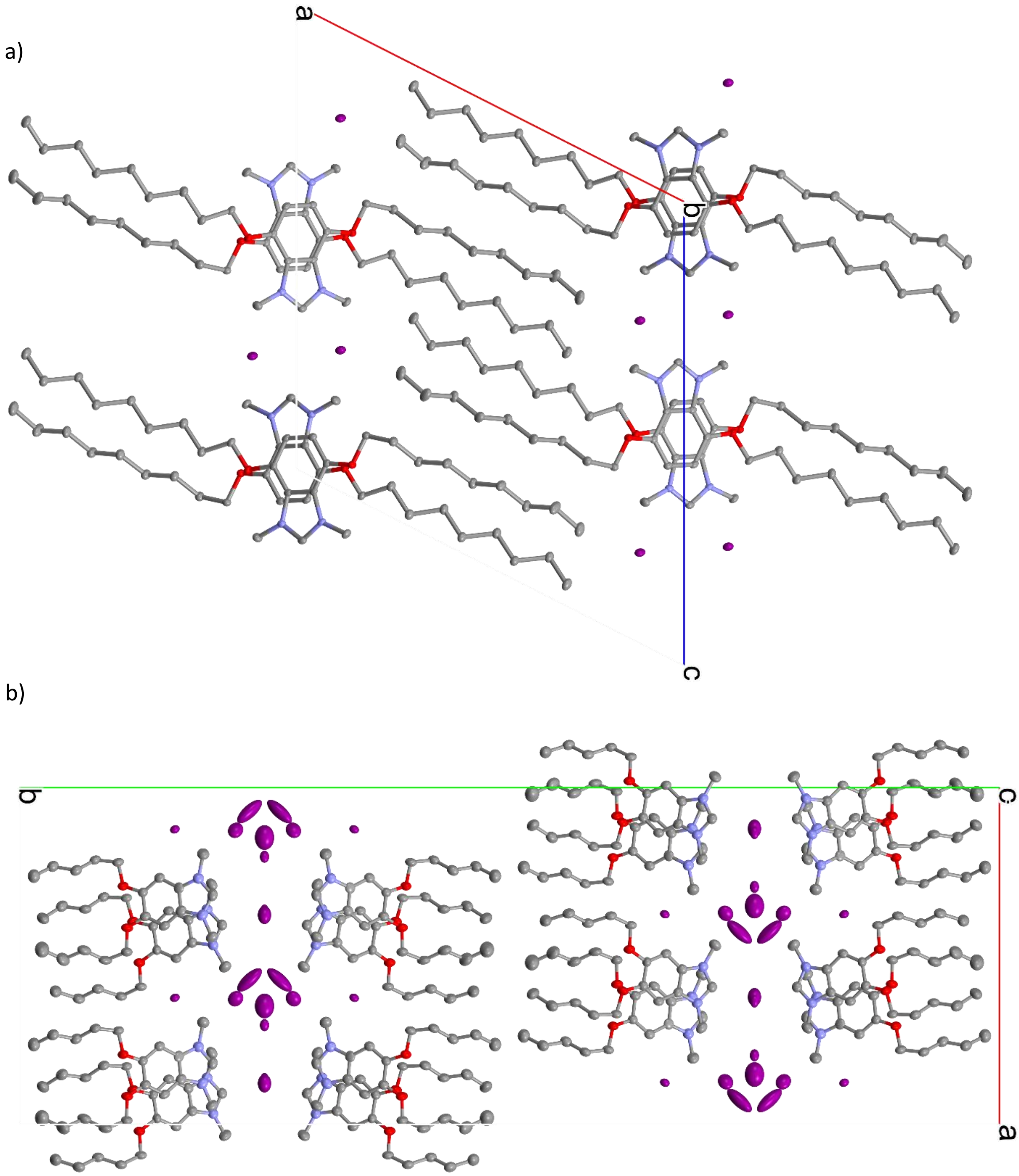

Figure S160. Illustration of the packing of a) **L-Me$_2$Bi(9)I** view along the *b*-axis and b) **U-Me$_2$Bi(5)I** view along the *c*-axis. Displacement ellipsoids at 50% probability level; hydrogen atoms omitted; colour code: O red, C grey, N blue; I purple.

When viewed perpendicular to the benzimidazole layers (Figure S161, along the *c*- and *a*-axis, respectively), the linear derivative **L-Me$_2$Bi(9)I** shows adjacent layers rotated by approximately 90° relative to each other. Within these layers, the benzimidazole moieties are arranged alternately with their imidazole and benzene faces oriented toward one another, while the iodide ions occupy the interlayer spaces. Overall, the molecules adopt a herringbone-type arrangement in the crystal, with overlapping side chains build up from interdigitating alkoxy groups; notably, the side chains are not coplanar with the respective benzimidazole core.

In contrast, the U-shaped derivative **U-Me$_2$Bi(5)I** forms a structure consisting of well-defined layers. The molecular layers comprise parallel-arranged side chains and benzimidazole units that are slightly tilted upward or downward. The individual molecular layers either stack directly via their side chains without an intervening separation layer or are separated by a layer of iodide ions between the benzimidazole units; the iodide ions are highly disordered.

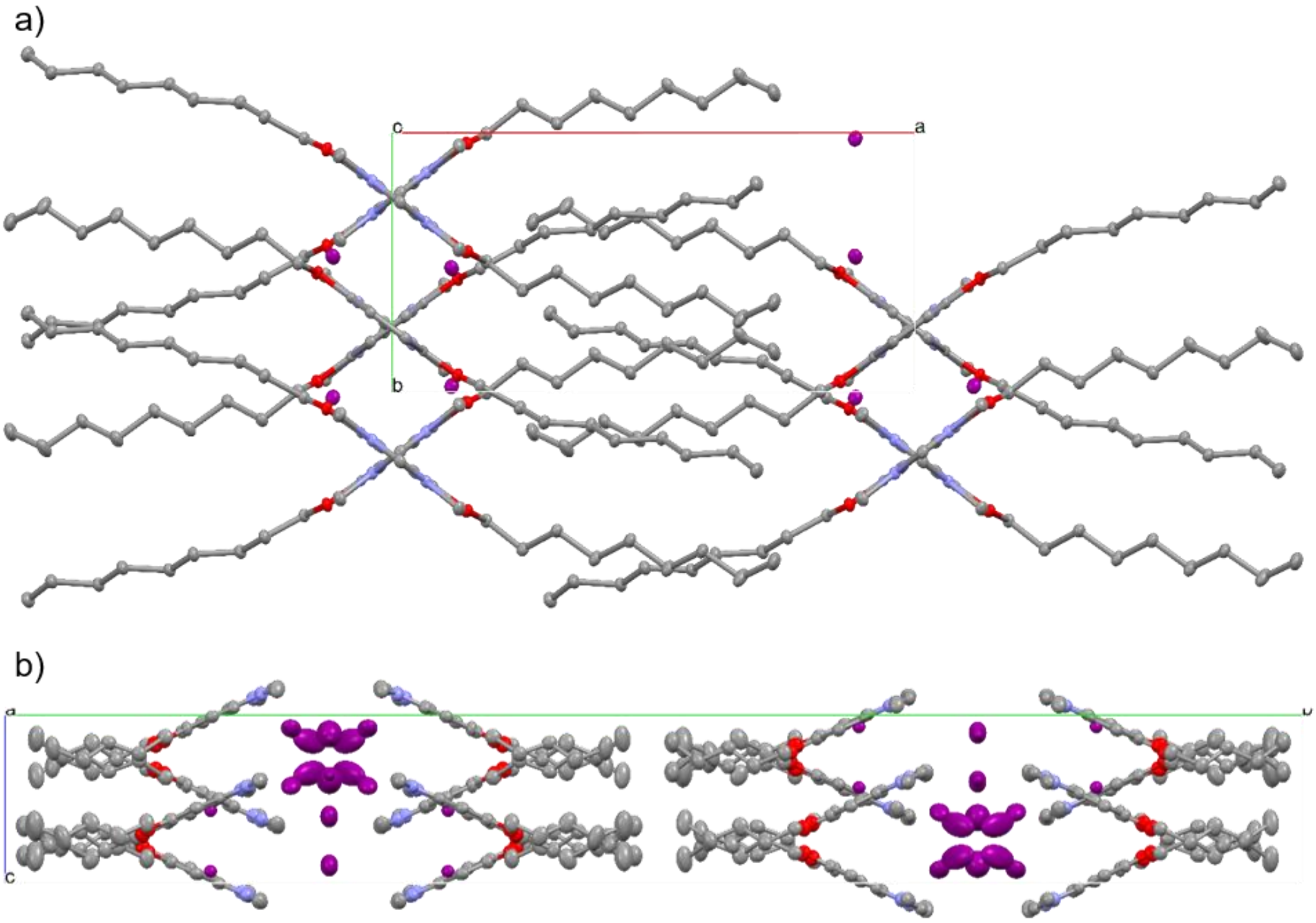

Figure S161. Illustration of the packing of a) **L-Me$_2$Bi(9)I** view along the *c*-axis and b) **U-Me$_2$Bi(5)I** view along the *a*-axis. Displacement ellipsoids at 50% probability level; hydrogen atoms omitted; colour code: O red, C grey, N blue; I purple. Further data are listed in the ESI, chapter 5.

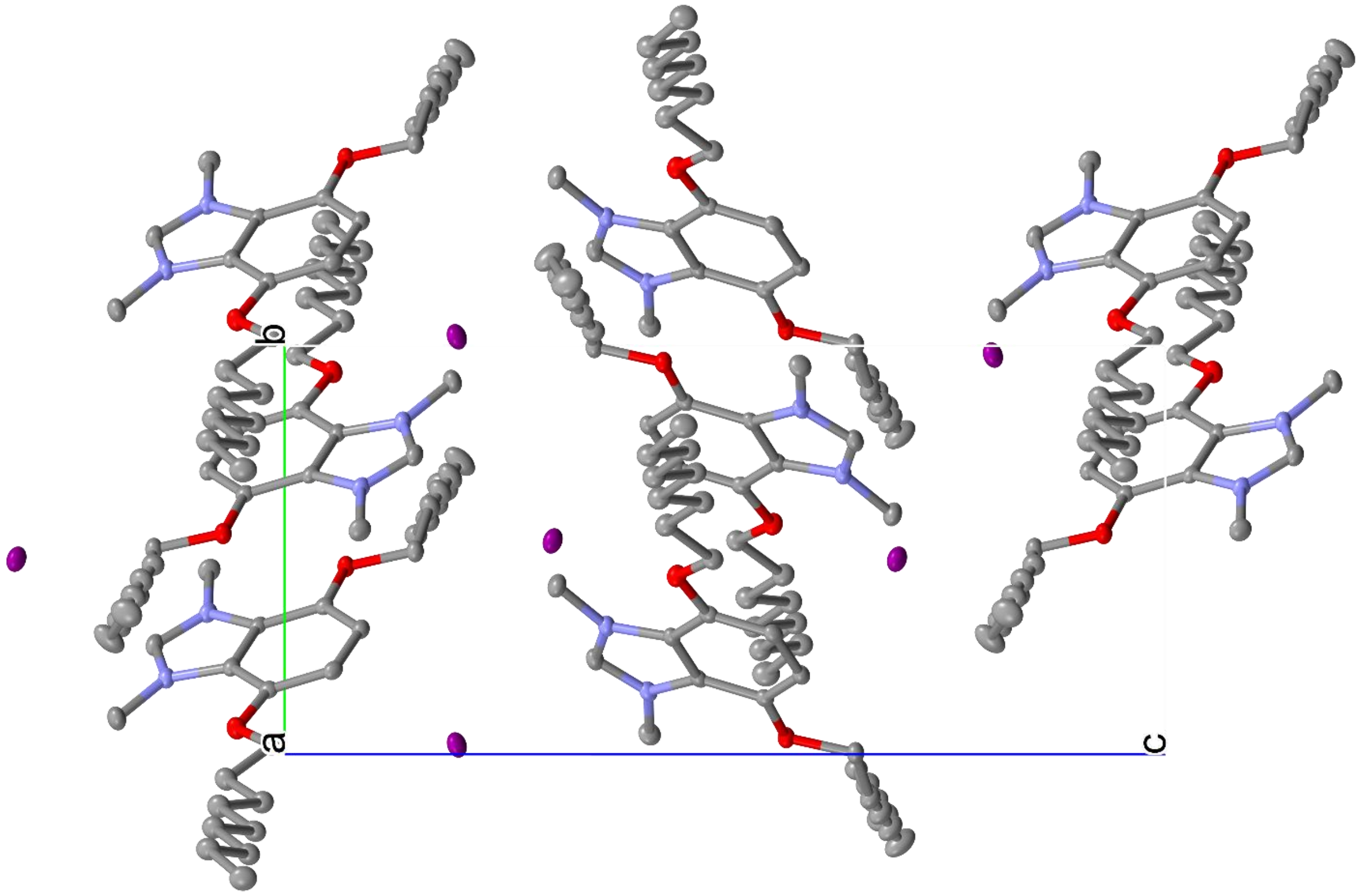

Figure S162. Illustration of the packing of **L-Me$_2$Bi(9)I** in the unit cell. View along the *a*-axis. Displacement ellipsoids at 50% probability level; hydrogen atoms omitted; colour code: I purple, O red, C grey, N blue.

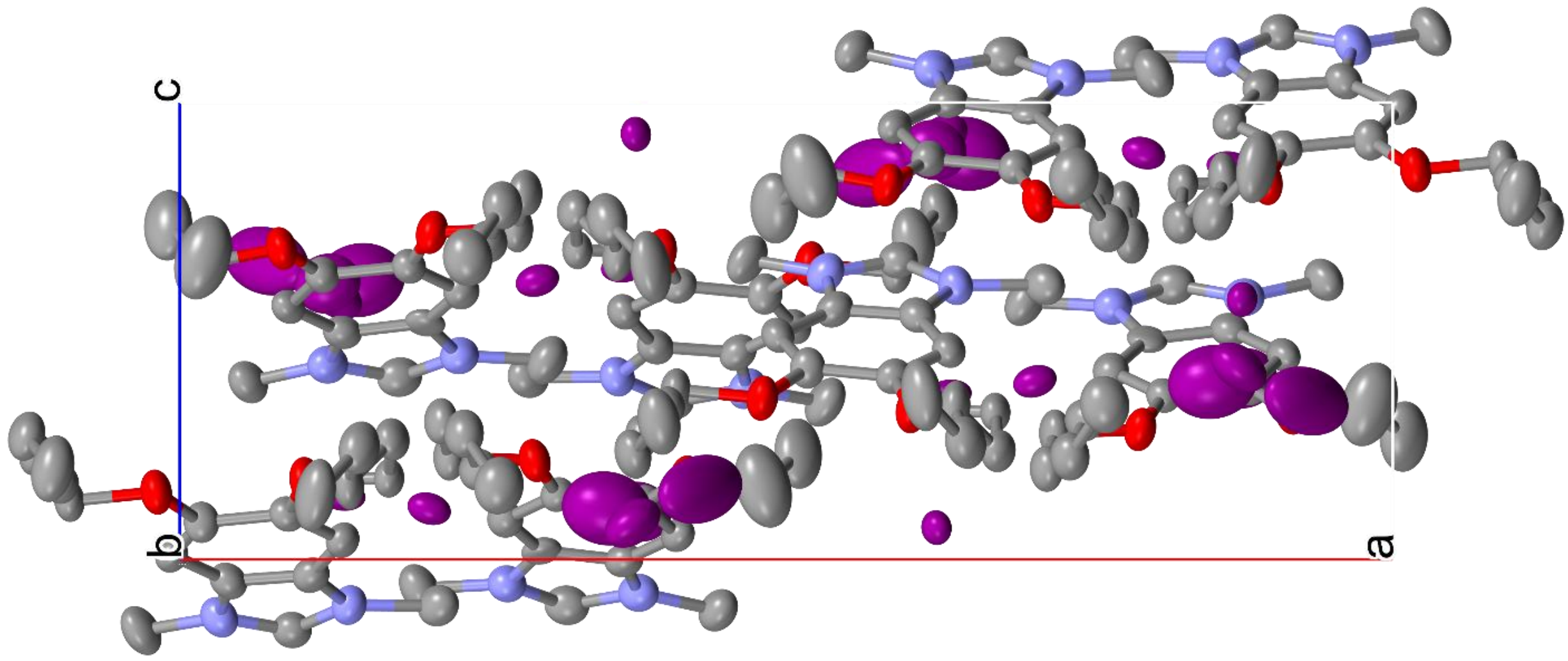

Figure S163. Illustration of the packing of **U-Me$_2$Bi(5)I** in the unit cell. View along the *b*-axis. Displacement ellipsoids at 50% probability level; hydrogen atoms omitted; colour code: I purple, O red, C grey, N blue.

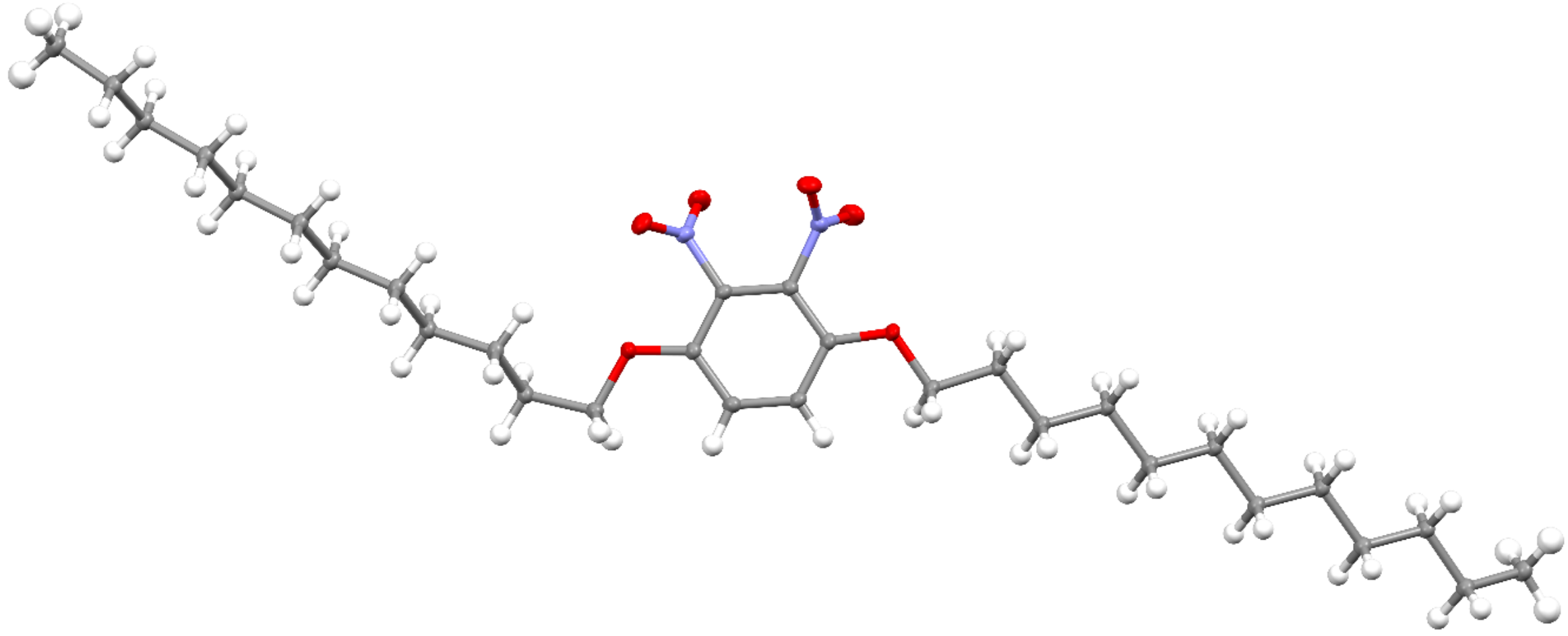

Figure S164. Illustration of the solid state structure of **L-Nitro(12).** Displacement ellipsoids at 50% probability level; colour code: O red, C grey, N blue; H white.

Table S5: Crystallographic data for **L-Bi(14)**, **U-Bi(6), L-Nitro(12)**.

Identification code	**L-Bi(14)**	**U-Bi(6)**	**L-Nitro(12)**
CCDC numcalcd	2532065	2532063	2532062
Empirical formula	$C_{34.96}H_{61.88}N_2O_2$	$C_{19}H_{30}N_2O_2$	$C_{30}H_{52}N_2O_6$
Formula weight	542.24	318.45	536.73
Temperature/K	160.00	100(2) K	100.00
Crystal system	triclinic	monoclinic	monoclinic
Space group	P-1	$P2_1/c$	$P2_1/c$
a/Å	18.1840(7)	21.683(3)	20.5857(19)
b/Å	19.0749(7)	9.3758(13)	8.4078(8)
c/Å	31.6206(11)	9.2803(13)	17.3325(17)
α/°	74.171(2)	90	90
β/°	74.076(2)	97.573(4)	93.302(5)
γ/°	84.364(2)	90	90
Volume/Å3	10143.8(7)	1870.2(5)	2994.9(5)
Z	12	4	4
ρcalcg/cm3	1.065	1.131	1.190

μ/mm-1	0.064	0.073	0.082
F(000)	3620.0	696	1176.0
Crystal size/mm3	0.42 × 0.37 × 0.18	0.420 x 0.380 x 0.150	0.598 × 0.382 × 0.328
Radiation	MoKα (λ = 0.71073)	MoKα (λ = 0.71073)	MoKα (λ = 0.71073)
2Θ range for data collection/°	1.386 to 53.032	1.895 to 25.413	3.964 to 61.41
Index ranges	-22 ≤ h ≤ 22, -23 ≤ k ≤ 23, -39 ≤ l ≤ 39	-26<=h<=25, -11<=k<=8, -11<=l<=10	-29 ≤ h ≤ 29, -7 ≤ k ≤ 11, -24 ≤ l ≤ 24
Reflections collected	145131	10890	64142
Independent reflections	41744 [Rint = 0.0575, Rsigma = 0.0872]	3417 [R(int) = 0.0482]	9242 [R_{int} = 0.0272, R_{sigma} = 0.0196]
Data/restraints/parameters	41744/5739/2811	3417 / 0 / 210	9242/0/346
Goodness-of-fit on F2	1.543	1.038	1.031
Final R indexes [I>=2σ (I)]	R1 = 0.1186, wR2 = 0.2773	R1 = 0.0764, wR2 = 0.1749	R_1 = 0.0383, wR_2 = 0.1006
Final R indexes [all data]	R1 = 0.2783, wR2 = 0.3471	R1 = 0.1093, wR2 = 0.1841	R_1 = 0.0532, wR_2 = 0.1138
Largest diff. peak/hole / e Å-3	0.60/-0.44	0.389/-0.291	0.41/-0.29

Table S6: Crystallographic data for **L-Me_2Bi(9)I** and ***p*-Me_2Bi(5)I**.

Identification code	**L-Me_2Bi(9)I**	**U-Me_2Bi(5)I**
CCDC number	2532061	2532066
Empirical formula	$C_{27}H_{47}N_2O_2I$	$C_{19}H_{31}I_{0.96}N_2O_2$
Formula weight	558.56	441.68
Temperature/K	100.00	172(30)
Crystal system	monoclinic	orthorhombic

Space group	$P2_1/c$	Pnma
a/Å	18.8541(5)	20.0203(7)
b/Å	8.3250(3)	58.245(6)
c/Å	20.1234(5)	7.5327(2)
α/°	90	90
β/°	116.8890(10)	90
γ/°	90	90
Volume/Å3	2817.08(15)	8783.8(10)
Z	4	16
ρcalcg/cm3	1.317	1.336
μ/mm-1	1.161	1.418
F(000)	1168.0	3617.0
Crystal size/mm3	0.392 × 0.319 × 0.08	0.203 × 0.052 × 0.031
Radiation	MoKα (λ = 0.71073)	Mo Kα (λ = 0.71073)
2Θ range for data collection/°	4.064 to 61.222	4.068 to 52
Index ranges	-24 ≤ h ≤ 27, -11 ≤ k ≤ 10, -28 ≤ l ≤ 20	-24 ≤ h ≤ 24, -71 ≤ k ≤ 71, -9 ≤ l ≤ 8
Reflections collected	38135	51263
Independent reflections	8559 [R_{int} = 0.0251, R_{sigma} = 0.0248]	8729 [R_{int} = 0.0785, R_{sigma} = 0.0488]

Data/restraints/parameters	8559/0/293	8729/79/478
Goodness-of-fit on F2	1.019	1.234
Final R indexes [I>=2σ (I)]	R_1 = 0.0243, wR_2 = 0.0538	R_1 = 0.1140, wR_2 = 0.2645
Final R indexes [all data]	R_1 = 0.0350, wR_2 = 0.0581	R_1 = 0.1217, wR_2 = 0.2682
Largest diff. peak/hole / e Å-3	0.62/-0.50	1.79/-2.11

6. Literature

(1) SAINT. *Bruker AXS GmbH Karlsr. Ger.* **2016**.

(2) Sheldrick, G. M. SADABS-Bruker Nonius Scaling and Absorption Correction. *Bruker AXS Inc Madison WI USA* **2012**.

(3) Krause, L.; Herbst-Irmer, R.; Sheldrick, G. M.; Stalke, D. Comparison of Silver and Molybdenum Microfocus X-Ray Sources for Single-Crystal Structure Determination. *Appl. Crystallogr.* **2015**, *48* (1), 3–10.

(4) Sheldrick, G. M. *Acta Cryst* **2015**, *A71*, 3–8.

(5) Sheldrick, G. M. *SHELXL-20xx Univ. Gött. Bruker AXS GmbH Karlsr. Ger.* **2012**.

(6) Robinson, W.; Sheldrick, G. M.; Isaaks, N. W. Crystallographic Computing 4. In *Crystallographic Computing 4*; IUCr and Oxford University Press: Oxford, UK; Vol. Ch. 22.

(7) Sheldrick, G. M. *Acta Cryst.* 2008, pp 112–122.

(8) Dolomanov, O. V.; Bourhis, L. J.; Gildea, R. J.; Howard, J. A.; Puschmann, H. OLEX2: A Complete Structure Solution, Refinement and Analysis Program. *Appl. Crystallogr.* **2009**, *42* (2), 339–341.

(9) Forget, S.; Veber, M.; Strzelecka, H. New Pathways Towards Long-Chain Substituted Dibenzotetraaza [14] Annulenes. *Mol. Cryst. Liq. Cryst. Sci. Technol. Sect. Mol. Cryst. Liq. Cryst.* **1995**, *258* (1), 263–275.

(10) Antonisse, M. M.; Snellink-Ruël, B. H.; Yigit, I.; Engbersen, J. F.; Reinhoudt, D. N. Neutral Anion Receptors: Synthesis and Evaluation as Sensing Molecules in Chemically Modified Field Effect Transistors. *J. Org. Chem.* **1997**, *62* (26), 9034–9038.

(11) Shopsowitz, K.; Lelj, F.; MacLachlan, M. J. Regioselectivity in the Nitration of Dialkoxybenzenes. *J. Org. Chem.* **2011**, *76* (5), 1285–1294.

(12) Baker, M. V.; Brown, D. H.; Heath, C. H.; Skelton, B. W.; White, A. H.; Williams, C. C. Azolium-Linked Cyclophanes: Effects of Structure, Solvent, and Counteranions on Solution Conformation Behavior. *J. Org. Chem.* **2008**, *73* (23), 9340–9352.

(13) Baker, M. V.; Brown, D. H.; Simpson, P. V.; Skelton, B. W.; White, A. H. *Synthesis and Characterisation of (Alkoxybenzimidazolin-2-ylidene) Palladium Complexes: The Effect of Ancillary Ligands on the Behaviour of Precatalysts*; Wiley Online Library, 2009.

(14) Judele, R.; Laschat, S.; Baro, A.; Nimtz, M. Gallic Esters of 4, 5-Dinitrocatechol as Potential Building Blocks for Thermotropic Liquid Crystals. *Tetrahedron* **2006**, *62* (41), 9681–9687.

(15) Ong, C. W.; Liao, S.-C.; Chang, T. H.; Hsu, H.-F. In Situ Synthesis of Hexakis (Alkoxy) Diquinoxalino [2, 3-a: 2 ‘, 3 ‘-c] Phenazines: Mesogenic Phase Transition of the Electron-Deficient Discotic Compounds. *J. Org. Chem.* **2004**, *69* (9), 3181–3185.

(16) Sessler, J. L.; Callaway, W. B.; Dudek, S. P.; Date, R. W.; Bruce, D. W. Synthesis and Characterization of a Discotic Uranium-Containing Liquid Crystal. *Inorg. Chem.* **2004**, *43* (21), 6650–6653.

(17) Yoo, K.; Kim, S.; Han, N.; Kim, G. E.; Shin, M. J.; Shin, J. S.; Kim, M. Stepwise Blue-Red-Yellow Color Change of a Polydiacetylene Sensor through Internal and External Transitions. *Dyes Pigments* **2018**, *149*, 242–245.

(18) *Perkin Elmer Chem 3D pro, 15.1.0.144.*
